# A Sequence and Structure Based Method to Predict Putative Substrates, Functions and Regulatory Networks of Endo Proteases

**DOI:** 10.1371/journal.pone.0005700

**Published:** 2009-05-27

**Authors:** Prasanna Venkatraman, Satish Balakrishnan, Shashidhar Rao, Yogesh Hooda, Suyog Pol

**Affiliations:** 1 Advanced Centre for Treatment, Research and Education in Cancer, Navi Mumbai, Maharashtra, India; 2 Schrodinger, New York, New York, United States of America; Griffith University, Australia

## Abstract

**Background:**

Proteases play a central role in cellular homeostasis and are responsible for the spatio- temporal regulation of function. Many putative proteases have been recently identified through genomic approaches, leading to a surge in global profiling attempts to characterize their function. Through such efforts and others it has become evident that many proteases play non-traditional roles. Accordingly, the number and the variety of the substrate repertoire of proteases are expected to be much larger than previously assumed. In line with such global profiling attempts, we present here a method for the prediction of natural substrates of endo proteases (human proteases used as an example) by employing short peptide sequences as specificity determinants.

**Methodology/Principal Findings:**

Our method incorporates specificity determinants unique to individual enzymes and physiologically relevant dual filters namely, solvent accessible surface area-a parameter dependent on protein three-dimensional structure and subcellular localization. By incorporating such hitherto unused principles in prediction methods, a novel ligand docking strategy to mimic substrate binding at the active site of the enzyme, and GO functions, we identify and perform subjective validation on putative substrates of matriptase and highlight new functions of the enzyme. Using relative solvent accessibility to rank order we show how new protease regulatory networks and enzyme cascades can be created.

**Conclusion:**

We believe that our physiologically relevant computational approach would be a very useful complementary method in the current day attempts to profile proteases (endo proteases in particular) and their substrates. In addition, by using functional annotations, we have demonstrated how normal and unknown functions of a protease can be envisaged. We have developed a network which can be integrated to create a proteolytic world. This network can in turn be extended to integrate other regulatory networks to build a system wide knowledge of the proteome.

## Introduction

Proteases can activate or truncate functions of proteins, unfold a cascade of events, trigger development or differentiation and cause cell death [1], [2]. Regulation of proteolysis is therefore vital to cellular homeostasis. Due to such intricate involvement of proteases in a variety of cellular functions, it is not surprising that aberrant changes in regulation of their function is associated with many malignancies [2]. While a large number of proteases have been identified over the years, only a few corresponding natural substrates have been recognized. Therefore, the general role of these proteases under normal conditions is not as obvious as their role for example, in tumor invasion [3]–[5]. Several different methods are presently employed to bridge the existing gap between information pertaining to natural substrates and the normal physiological function of proteases [6]–[13]. While one would expect computational approaches to be an integral part of such investigation, very few *in silico* methods are currently available for the prediction of natural substrates of proteases. CaSPredictor for the prediction of caspase substrates [14], GraBCas for Granzyme B and caspase substrates [15] are notable among them. These tools are classifiers designed for high accuracy and are based on known natural substrates which act as training sets. Such techniques are therefore restricted to few well studied enzymes such as caspases, trypsin and granzymes and cannot be extended to other proteases. CutDB is a curated database that currently documents proteases from all organisms, along with their experimentally identified and predicted substrates [16]. Newer approaches and programs have been designed catering to protease families. Prediction of Protease Specificity, PoPS [17] is a server which tries to predict natural substrates by finding matches for a potential enzyme active site. It provides an environment to the user to model substrate specificity using available information. Since the output is user dependent, results are prone to be erroneous.

As a result we felt that there is a definite requirement to find prediction methods for large scale global profiling of natural substrates of the human proteases and decided to test the potential of minimal sequence motifs for this purpose. Minimal motif of three amino acids is apparently sufficient to confer functional specificity in proteins [18]. In our method we have used two basic principles inherent to proteolysis namely, (a) sequence specific information [19] or the qualitative criterion, and (b) a three dimensional structure (3D) related quantitative criterion, called relative solvent accessibility. Using this method we report here *in silico* identification of substrates of serine proteases from the Protein Data Bank (PDB) [20] and a database of protein disorder, DisProt [21]. Using relative solvent accessibility and subcellular distribution as filters we perform subjective validation of potential substrates of matriptase and assign new functions to this enzyme. Furthermore, using relative solvent accessibility as a criterion, we project a novel method to build proteolytic cascades and regulatory networks. In addition we have projected the use of sequence specific information to identify putative substrates of majority of human endo proteases from the entire human proteome. It is anticipated that the position specific information on substrate specificity, structural (of the enzyme and substrate) and localization information will increase the accuracy of the prediction methods and eliminate the false positive ones from the ensemble.

## Results

To identify a minimal specificity motif, which can be used to provide as accurate and robust predictive method as possible, we considered several features of enzyme catalysis. They are 1) specificity determinants unique to each protease type, 2) discriminative power of different enzymes within the catalytic type, 3) accessibility of the cleavage site and 4) localization. While the primary site specificity has been extensively used in some of the other predictive methods, no one has arrived at a general formula capable of addressing proteases of an entire catalytic type as a whole. No one explicitly considers the tertiary structure information or protein localization, both of which are of considerable importance *in vivo*.

### I. Guideline 1

#### a) Cleavability based on sequence specificity–a qualitative criterion

Cleavage sites on proteases are short and span a contiguous stretch of residues of the type P4P3P2P1-P1′P2′P3′P4′ with the scissile bond between the P1-P1′ residues. Proteases can also cleave short peptides of two or more residues. MEROPS, the peptidase database [22], is a manually curated information resource for peptidases, which enlist all such experimentally observed cleavage sequences. We call such short peptide sequences as the ‘artificial peptide substrates’. In many instances such short peptide sequences have been used either in the design of inhibitors or in position scanning approaches to optimize the sequence specificity [9], [23], [24]. Some investigators have made best use of such information by identifying disallowed amino acids to discriminate between proteases of similar specificity [25]. Therefore, these short sequences harbor valuable information. Keeping these in mind we simply asked whether such minimal sequences can be used to link MEROPS, with PDB, DISPROT and the human proteome databases. We call this approach: ‘Prediction of Natural Substrates from Artificial Substrate of Proteases’ (**PNSAS**).

In order to retrieve sequences that can be used for the prediction, we cataloged the number and type of short peptide substrates in MEROPS (**Table 1**) wherein peptides of varying length (one-where a single amino acid is linked to a fluorophore to those which are eight amino acids long) are reported. Predictions based on very short sequence will tend to be more non-specific and those with large number of residues more restrictive. To obtain a balance between specificity and versatility, we chose to study tripeptide sequences. As seen below, they represent an optimum number for large scale positive identification. Moreover, our preliminary screening against the PDB database wherein we derive the structural information vital to our method indicated that a reasonable number of hits amenable for analysis will be obtained using tripeptides. Most often in such short peptide substrates, the P1′ position is designed to have a fluorophore (of varying sizes) to enable activity measurements. However proteases, for example, caspases show clear discrimination for amino acids at P1′ position incorporation of which should help in the prediction methods [26].

**Table 1:** Summary of the number and type of peptide substrates in MEROPS database.

Catalytic Type	Length Of the Peptide Substrates								Total number of substrates
	1	2	3	4	5	6	7	8	
**Serine**	55	120	102	43	15	37	38	600	1010
**Aspartate**	0	4	4	3	7	2	17	272	309
**Cysteine**	21	41	22	25	11	9	17	633	779
**Threonine**	5	9	9	6	0	0	0	3	32
**Metallo**	6	120	42	19	74	38	56	633	988

Peptides one to seven amino acids long were derived from short peptide substrates (synthetic/artificial). Peptide of length ‘one’ indicates a single amino acid followed by a fluorophore and the fluorophore itself is not counted. Peptides with eight amino acid residues were derived from natural protein substrates.

In addition to the artificial peptide substrates, MEROPS also documents experimentally identified cleavage sequences from natural substrates. These are recorded as octapeptide sequences in MEROPS with the 4^th^ and 5^th^ residue corresponding to the P1 and P1′ positions. These longer octapeptide sequences would be more specific, but also restrictive. By virtue of being more specific these can be used against a large database like the entire human proteome. However, the limitations of such relatively long sequences are apparent when additional filters need to be incorporated based on the 3D structure of the protein. PDB is a much smaller data base and as will be seen below provides limited output with the octamers.

From the MEROPS data base, we downloaded all the uniprot sequences of those proteins reported to be cleaved by the four major types of proteases namely, metallo, cysteine, aspartate and serine proteases. We extracted all the octapeptide cleavage sequences from these natural substrates and made a query set which we call the NQSS (Naturally derived Query Sequence Set; **Table S1**). We developed a method to extract pattern matches within a dataset when a query is placed (which in this instance is a contiguous stretch of eight amino acids). To verify applicability of the method, we concentrated on serine proteases and chose two proteases for which a significant number of substrates have been identified: furin and thrombin. We split the substrates into a training and test set. The octapeptide cleavage sequences (16/31 for furin and 55/109 for thrombin), not surprisingly, fetched 100% hits from the training set. We then used 964 uniprot sequences that correspond to all the natural serine protease substrates reported in MEROPS. From this data set 48 and 68% of substrates were correctly identified by the furin and thrombin training sets, respectively (**Table 2**). More than one hit (perfect match) i.e., alternative cleavage sites within the same protein are not counted here but the results are tabulated separately (**Table S2**). We also derived shorter sequence motifs of four and three amino acids long (P3P2P1P1′ and P3P2P1) and repeated the exercise to see if we can increase the coverage of the known substrates. As clearly seen from Table 2, tripeptide query sequence set (QSS) fetched 85 and 98% of the already identified substrates of furin and thrombin respectively, while the tetrapeptide QSS fetched 56 and 79% of the substrates respectively (**Table 2**). Also to be noted is the fact that in going from the octapeptide to the tripeptide QSS, the number of hits obtained increases almost exponentially. A tripeptide motif therefore has the best representation of the cleavage sequences, offers the advantage of extensive coverage and greater flexibility in identifying new substrates. The caveat is that large number of false positive identifications is inevitable.

**Table 2:** Identification of reported substrates of furin and thrombin using Query Sequence Set.

Peptide Length	Furin			Thrombin		
	Octa peptide	Tetra peptide	Tri peptide	Octa peptide	Tetra peptide	Tri peptide
Total Known natural substrates	27	27	27	98	98	98
Training Set	16	16	16	55	55	55
Number of proteins identified from entire 964 uniprot sequences of natural substrates of human serine proteases*	19	127	714	73	348	840
Known substrates-correct identification	13/27	15/27	23/27	67/98	77/98	96/98
Percentage correct identification	48%	55.6%	85%	68%	78.6%	98%
Putative novel substrates identified	6	112	691	6	271	744

Furin and thrombin cleavage sequences from the natural substrates were extracted and a training set of different types, i.e. octa, tetra and tri peptides were created. The terapeptide sequence indicates P3P2P1P1′ and the tripeptide sequence indicates P3P2P1 residues. The ability of these query sequences to retrieve known/reported substrates was analyzed as described under methods.

* Note that for the sake of clarity the number of proteins and not the number of cleavage sites are reported here.

Our simple algorithm looks only for a perfect match for an experimentally derived peptide sequence and does not allow any flexibility/mismatches. We deliberately refrain from introducing any variable in the sequence, as our aim is to find exact matches for already observed cleavages. As an illustration of a highly specific query set, we used the octapeptide cleavage sequences of serine, metallo, cysteine and aspartic proteases and identified matches from the entire human proteome **(** **Table S3** **)**. Identified proteins are regarded as the most highly likely substrates of these proteases provided they pass through the biologically relevant filters described below.

#### b) Differential specificity between enzymes of the same family

While the active sites of enzymes of an entire protease type show common preference for residues at the scissile bond (P1- P1′), with a few exceptions, individual enzymes from within the same catalytic type may have unique preferences at other positions [9]. Short synthetic peptides (called artificial here) are often designed to measure activity or optimize binding. We assembled all the short tripeptide sequences listed in MEROPS (although not every one of them would be an optimized sequence) for the enzymes of the serine protease type and short listed those that confirmed to the following kind: P3P2P1. Residue at P1′ was ignored. Using these peptides, a library was created and referred to as ‘Artificial Query Sequence Set’ (**Table S1**). This is a representative set and the guiding principles have been derived from experimental determination. Unique and additional specificities have been reported for furin (RxR/KR) [27] and β-tryptase (PRNR) [28]. Therefore, their query sequences were designed accordingly to include the tetrapeptides. In the case of furin, we could not find in MEROPS, peptide substrates that confirmed to the P3P2P1 type. It is to be emphasized that accuracy of our method is strongly dependent on experimental determination of position specific information and will improve when rigorously optimized sequences are available for a protease, including information about those that are disallowed in some positions between closely related proteases [25]. A right combination of amino acids at the appropriate position which takes into account the specificity dictated by an enzyme active site constitutes a qualitative criteria, called ‘cleavability’.

To obtain an idea about the representation of amino acid type within the artificial peptide substrates of serine proteases, we tabulated the observed cleavage sequences and compared the amino acids present at the scissile bond (data not shown). Majority of the proteases of the serine catalytic type harbor Arg/Lys and so do the artificial short peptide substrates. In granzyme B, a preference for Asp, an oppositely charged residue was observed which was also reflected in the artificial peptide query set. As mentioned before, the P1′ position seems to be flexible and it is generally utilized to add a fluorophore which varies in size and type. Although Ser seems to dominate the P1′ position in most of the natural cleavage sequences, with 35/63 proteases having at least one cleavage sequence with P1′ serine, other types of amino acids were also observed reflecting flexibility at this position (data not shown). In some instances, protease specific information was also evident as in the case of thrombin where P2 is predominantly occupied by a proline residue. Representation of amino acids present in the natural substrates, increases the level of confidence in a predictive approach using short peptides of the kind described here. The artificial QSS containing the P3P2P1 residues (**Table S1**) was used to query the human proteome database for exact matches (**Table S4**). We have also used the tripeptide QSS of serine proteases (part of the NQSS; for example see tripeptide query sequence of furin and thrombin in the training set –Table S1) derived from the naturally identified substrates and scanned the human proteome for exact matches which would be made available on our website. Matches for the tetrapeptide query P3P2P1P1′ would be a subset of this data.

### II. Guideline 2

#### Accessibility - a quantitative criterion

**Figure 1:**
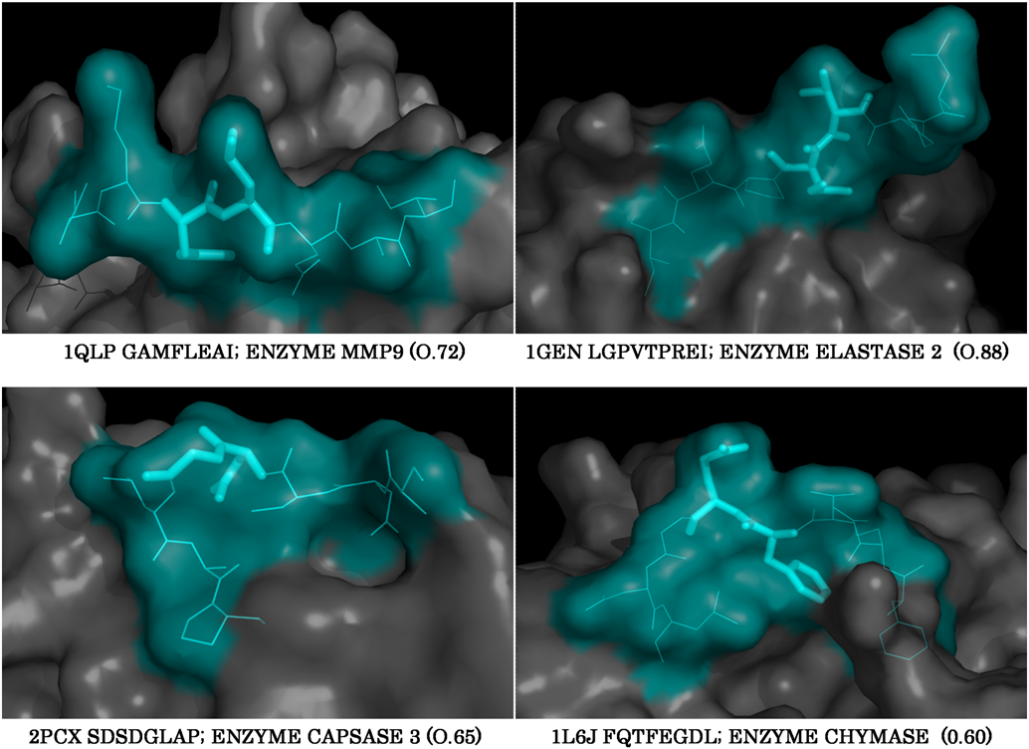
The three dimensional context of the cleavage sequences in natural substrates. a) Structures of the known protein substrates of proteases with their octapeptide cleavage sequences are depicted in cyan. Amino acids at the P1 and P1′ positions are represented as sticks. Protein structure is represented as a surface. The rSASA values for the P3P2P1P1′ sequence is shown in parenthesis.

For a ‘cleavable’ sequence within the protein (as identified above) to be cut by the corresponding protease, the cleavage site must either be surface exposed or present in flexible/disordered region (accessible) in the context of the folded 3D structure of the protein. To obtain such structural information, we queried octapeptide cleavage sequence (NQSS) against human proteins derived from PDB (**Table S5**). To ‘quantify’ accessibility, we calculated Solvent Accessible Surface Area or SASA [29]. To attribute the contribution of SASA to accessibility, we calculated relative values (rSASA) by considering the highest SASA value as 1 (**Table S5**). For validation we excluded those hits which were not reported in MEROPS (potential novel substrates). Majority of the cleavage sites (90.7%) had an rSASA value >0.4. Structures of some of these proteins are shown in **Figure 1**. As noted before, NQSS contains all the octapeptide cleavage sequences for the four major protease type: metallo, cysteine, serine and aspartate. Our ability to fetch back the proteins documented in MEROPS from PDB illustrates the applicability and the reliability of the method. Also, one would expect this approach to be useful to at least a vast majority of endo proteases in general and the exceptions are discussed below.

We also queried the PDB with the short artificial peptide QSS of serine proteases and classified the PDB hits into those with rSASA ≥0.4 (most likely substrates) and those <0.4 (**Table S6**). For comparative purposes, the SASA values of the octapeptide cleavage sequences within the known natural substrates were recalculated using only the P3P2P1P1′ residues (**Table S5**). Based on this calculation, 69% of substrates had rSASA values ≥0.4 and 87% of the substrates ≥0.3. One may use the lower (≥0.3 instead of ≥0.4) cutoff to include more likely substrates. If two different proteases have a cleavable sequence on the same protein or the same protease has different cleavable sites on the same protein, the site with more accessibility (≥0.4) would be considered as a more likely candidate than those with rSASA below 0.4. Many cleavage sites were present in regions with no distinct electron density which are considered as disordered regions, or regions with high degree of flexibility. Since SASA cannot be calculated for such regions, an arbitrary value of 2 was assigned as a quantitative measure. However, when such disordered regions were present in the very beginning or end of the PDB structure they were in general ignored for the following two reasons: 1. processing at the very termini may have less biological sense unless it inactivates the enzyme; 2. the PDB structure may have a partial sequence in which case the site may not be disordered or accessible in the full length protein.

Apart from their lack of electron density in crystal structures, disordered regions have been identified in proteins using other experimental strategies and are documented in DisProt, a database of protein disorder [21]. Such regions span short stretches or run through the entire length of the protein. These regions were scanned for exact matches (**Table S6**) using artificial QSS listed in **Table S1**. Our analysis of the known natural protein substrates indicated that cleavage sites are often present in disordered regions of proteins. Therefore, we believe that proteins containing sequence matches within the disordered regions are very likely candidate substrates. Disordered or flexible regions are often suspected to be proteolytic targets [30] and we provide a platform to test this by identifying a potential enzyme-substrate pair. Due to the small size of this database as well as the PDB, matches were not observed for many enzymes.

### III. Identification of putative substrates of matriptase

In order to validate our method we undertook subjective analysis of the substrates of matriptase, an epithelial membrane bound serine protease also found in extracellular environment. Although its normal functions are yet to be clearly elucidated, it is presumed to be involved in adhesion, growth, proliferation and differentiation [31]. It is also implicated in a wide variety of cancers involving the epithelium, particularly in tumor invasion and angiogenesis [32], [33].

Position scanning approaches and the power of phage display have been used to identify the sequence preference of matriptase [34]. These studies identified two consensus sequences-one with the P4-P1′ positions occupied by R/KXSRA and the other with XR/KSRA where X is a non-basic residue. The artificial peptide substrates in MEROPS [35] belong to the type where P3 is a non-basic residue and P1 is always an R (except for AFK). Although Ala has been identified as the preferred P1′ residue proposed cleavage sequences in natural substrates of matriptase contain Val, Ile, Gly or Ser indicating that P1′ may accept a variety of amino acids [31], [32]. The phage library selection indicates that either P3 or P4 could be basic, but not both. However, activation sites of matriptase on profilaggrin have been mapped to RKRR-G [32] and that of VEGRF 2 to RRVR-K [31]. This is in variance with the projections from phage library.

**Figure 2:**
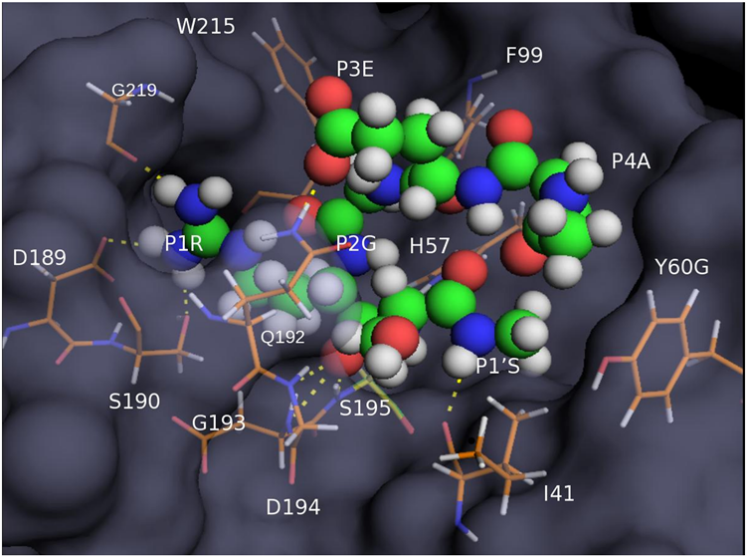
Structure of matriptase docked with a model peptide substrate. A) AEGRS (spheres) was docked to the matriptase structure (2GV6; light blue) using various components of Mastero (Schrödinger) as described under Text S1. Residues that are 4 Å distance from the ligand are shown as sticks. Polar interactions of the ligand with active site residues are indicated as dashes (yellow).

To obtain structural insights about the binding pocket in matriptase, we have docked a common scaffold EGRS with Arg (REGRS) and Ala (AEGRS) at the P4 position. Both peptides fitted well within the binding pocket. The pentapeptide AEGRS (GlideScore of-9.035 kcal/mole) seems to bind tighter than REGRS (GlideScore of −6.730 kcal/mole), as can be seen from its compact positioning in the matriptase cavity **(** **Figure 2** **)**. The guanidinium group of P1 Arg is set deep into the S1 pocket of the protein and is hydrogen bonded to Ser190 and Gly219. These interacting residues and the P1 Arg are held in position within the pocket made of Cys191, Val213, Gly216, Trp215 and Phe99. A salt bridge between P1 Arg and Asp189 reinforces the enzyme substrate interaction. Asp184 and Gly193 interact with the carbonyl carbon of the scissile bond which is covalently bound to the catalytic serine (Ser 195). Ser at the P1′ position is held within the binding pocket via long range van der Waals and electrostatic interactions with the His57 side chain and Ile41 backbone carbonyl oxygen. The P3 glutamate side chain carboxylate is hydrogen bonded to the side chain amide of Gln192 and P4 Ala is in energetically stable hydrophobic contact with Ile60 isobutyl side chain. In addition, interactions between the carboxylate moiety of the P3 Glu side chain of the ligand and the phenolic side chain of Tyr146 can be potentially mediated by a water molecule. When P4 is an Arg, additional hydrogen bonding interactions are made by the P4 side chain guanidinium group with Ile60 and Cys58 backbone carbonyl oxygen. Nevertheless, a lower binding affinity is predicted for this sequence, possibly due to the assessment of energetic penalty for the solvent exposure of the trimethylene chain formed by C^β^, C^γ^ and C^δ^ atoms in the side chain of the P1 Arg. Additional docking studies showed that the pocket holding the P1′ residue was able to accommodate multiple amino acids (data not shown). Also P3 could be changed to Gln with no drastic change in the binding geometry as the glutamine side chain amide is able to engage the side chain of Gln192 (similar to P3 Glu). Non-specific peptides like AAADS (GlideScore of 0.23 kcal/mole) demonstrate considerably reduced binding to the matriptase active site with GlideScore values being nearly 10 kcal/mole higher than the specific sequence AEGRS.

The above results indicate that proteases can indeed discriminate between short peptide sequences and the active site is in fact adopted to specifically bind closely related peptides. Therefore, even if the number of hits using these small sequences is going to be huge, position optimized sequence information can indeed be used *in silico* as a first line of screening to map protease cleavage sites in a high throughput manner.

### Analysis of human proteome results

Due to the results obtained by docking studies and the fact that even within the small set of natural substrates of matriptase identified so far, the rules of phage display library are in variance, we have used, P3P2P1 as QSS to screen for matches with the human proteome (**Table S4**). Some of the experimentally identified natural substrates of matriptase are matriptase itself, profilaggrin, pro-uPA, MMP3, laminin, collagen type IV, fibronectin, gelatin, pro HGF, VEGF2 and PAR-2 [31]–[33], [36]. Although activation of many of the above proteins by matriptase has been demonstrated, the exact *in vivo* processing is unclear and most of the substrates are referred to as ‘putative’. The P2 and P3 residues in these proteins (with the exception of matriptase) are different from those present in QSS. The expected cleavage site of matriptase harbors QAR, a motif present in our query set, but the P1′ position is occupied by Val. We identified matches with urokinase plasminogen activator preproprotein, laminin β-3 precursor, filaggrin and collagen from the human proteome indicating that there are alternative cleavage sites than those proposed earlier. Some of these protein substrates are homologous (collagen) or mature forms (filaggrin) of previously identified substrates. It will be interesting to see which one of these cleavage sites would be the preferred *in vivo* and how proteolysis is halted without cleavage at other sites. Accessibility of the site and/or the exosite specific preferences [37], tissue specificity, subcellular localization and topology would play a decisive role in this instance. To what extent relative rates of cleavages would help in differential susceptibility remains to be seen.

As mentioned before, the exact physiological role of matriptase in health and malignancy still remains to be clarified. The protease however is speculated to be involved in protease activation, epithelial and keratinocyte differentiation, receptor activation, growth factor stimulation, cell adhesion and matrix degradation [31], [36]. Epidermal growth factor receptor pathway substrate 8-like protein, fibroblast growth factor, many of the G protein coupled receptors and proteins, spermatogenesis associated homolog and keratin may be the candidate substrates under such conditions. Matriptase is associated with epithelial cancer, their metastasis, invasion and angiogenesis. Cancer-related proteins like epithelial cell transforming sequence 2 oncogene protein, FAT tumor suppressor 2 precursor, proto-oncogene tyrosine-protein kinase FGR, serologically defined colon cancer antigen, and angiotensin II receptor-associated protein are candidate substrates of matriptase predicted by our method.

### Analysis of PDB results

**Figure 3:**
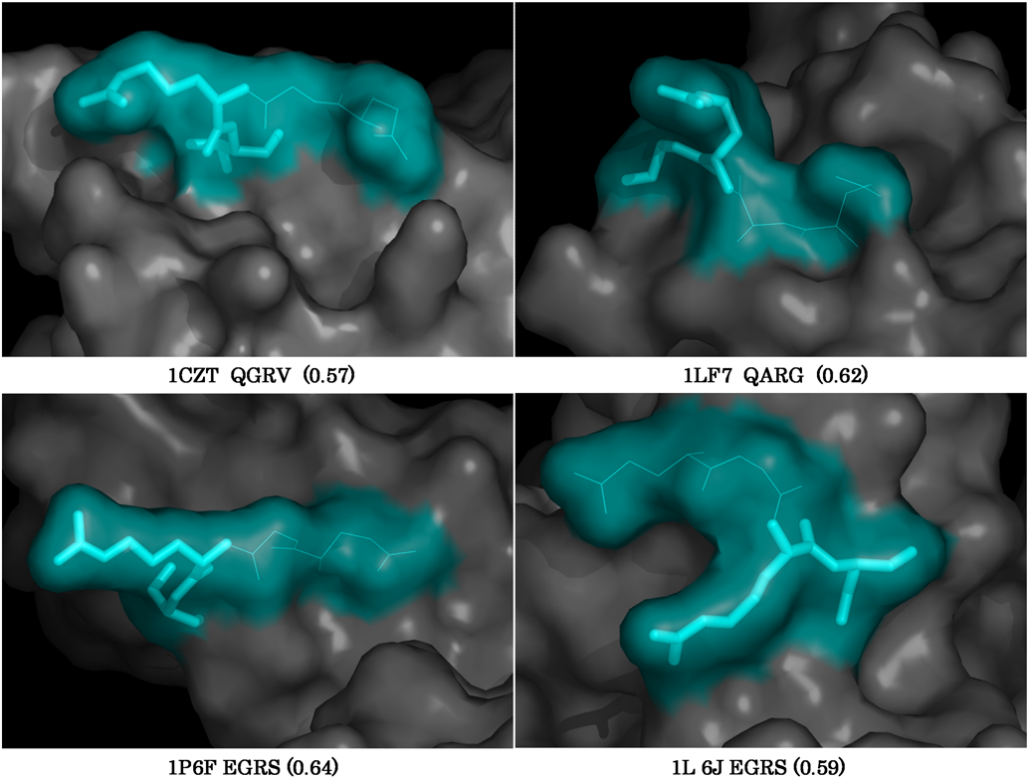
Three dimensional context of the cleavage sequences in putative substrates of matriptase. Structures of the putative substrates of matriptase with their tetrapeptide cleavage sequences are depicted in the format described in Figure 1.

We used matriptase QSS containing P3P2P1 residues to fetch matches from the PDB. Besides addressing similar questions as with the human proteome, structural information in PDB permits one to ask a more physiologically relevant question, i.e., is the identified site accessible or not? Proteins with matches to matriptase query set were rank ordered according to their rSASA values (**Table S7**). Out of 772 human proteins that were short-listed from PDB, we found 269 hits for matriptase query sequences (35.0%). After imposing rSASA filter (0.4), the number of hits was reduced to 100 (12.95%). **Figure 3** shows structures of some of these proteins emphasizing the accessibility of the cleavage sequence.

Not all proteins with accessible sites will be cleaved by a protease. Therefore yet another filter was created to restrict false positive hits. The filter was set as co-localization, a prerequisite that the protease and its potential substrate should be present in the same subcellular compartment (**Table S8**). Proteins localized to the membrane/extracellular region and an rSASA ≥0.4 were short-listed. This further reduced the number of likely candidates to 39 (5%). Matriptase is found either bound to the membrane with the catalytic site facing the extracellular milieu or is secreted into the extracellular environment. Those substrates that were membrane bound were further scrutinized to identify the topological location of the cleavage site. It turns out that in many such proteins which were characterized as membrane bound/extracellular, the actual cleavage site is present in the cytoplasmic region which is less likely to be cleaved by matriptase. Thus by following these stringent criteria the number of potential substrates was reduced to 16 (2%).

During this exercise we found several discrepancies about the information pertaining to subcellular localization, identification of the topology of a membrane protein and mapping of the cleavage sequence. In many instances, the subcellular localization was unclear. Localization is referred to as membrane, integral membrane or extracellular under the GO terms in PDB. We referred to uniprot data to identify the subcellular localization. Even in uniprot there are varying annotations- sometimes the localization is inferred by electronic annotation or it is referenced to ‘traceable to an author’. It is very difficult under such conditions to unequivocally assign the subcellular loci to the protein and the topology could be assigned only upon further reference to other databases or via comparison with the homologous sequences with relevant information. We illustrate this by two examples. Two of the Ephrin receptors 2 and 3 were found to have the cleavage sequence for matriptase. While the Ephrin receptor 3 details are clearly available in the uniprot to map the cleavage sequence, those of Ephrin receptor 2 is not. Ephrin receptor 3 has a mutation within the cleavage sequence in the recombinant protein. It is a LGR in PDB, while the uniprot natural sequence is LSR. The sequence lies within the kinase domain and therefore would face the cytoplasmic side. The cleavage sequence in Ephrin receptor 2 is by analogy on the extracellular face of the membrane and therefore could be a putative substrate. Yet another interesting example is the Mast/stem cell growth factor receptor (2EC8). We identified a cleavage sequence AFK in this protein which is topologically located in the extracellular region. The PDB structure however did not have the uniprot reference to it. We independently queried the protein in uniprot and found that the cleavage site is located in the potential extracellular domain. Interestingly, mutations in this protein leading to overactive kinase is associated with gastrointestinal stromal tumors [38]. It would be interesting to see the role of matriptase, if any, on the proteolytic processing of this protein, resultant activation of the kinase and its effect on such tumors.

We also found examples where the cleavage sites confirm to all stringent criteria but are present within the functional domain of a protein, for example, those of MMP9 and FGF23. Such cleavages would inactivate the protein. Inactivation of MMP9 by matriptase would be contradictory to the known role of matriptase in tumor invasion [39]. At present the relationship between matriptase activity and MMP9 inactivation is unclear, although it is possible that matriptase may inactivate MMP9 under normal conditions to attenuate a physiological function from stepping out of regulation, for example, in bone resorption and development [3]. It is possible that there are differences in proteolytic patterns between normal and pathological conditions. However, any such reasoning as mentioned here is highly speculative and needs to be treated with extreme caution.

Careful analysis of the structure and accessibility of the cleavage sequence has an immense impact in deciding for or against a possible cleavage site especially if (a) such an additional site has not been previously reported or (b) the cleavage of which may lead to inactivation of the enzyme. Vascular endothelial growth factor receptor 2, an integral membrane protein involved in angiogenesis has recently been identified as a potential substrate of matriptase [31]. Our search picked an alternative cleavage site GRG (rSASA 0.48; **Figure 3**) in this protein from the PDB. However, topologically the site was found to be located on the cytoplasmic side of the membrane and therefore is unlikely to be cleaved by matriptase. Similarly, in the case of urokinase plasminogen activator, a secretary protein, we identified a cleavage sequence EGR with P1′ Cys. This cleavage sequence is present within the kinase domain and cleavage at this site is likely to inactivate the protein. Careful look at the structure indicates that this Cys residue is involved in a disulfide linkage. It will be interesting to find if the presence of such a disulfide bond would prevent cleavage at this site.

**Figure 4:**
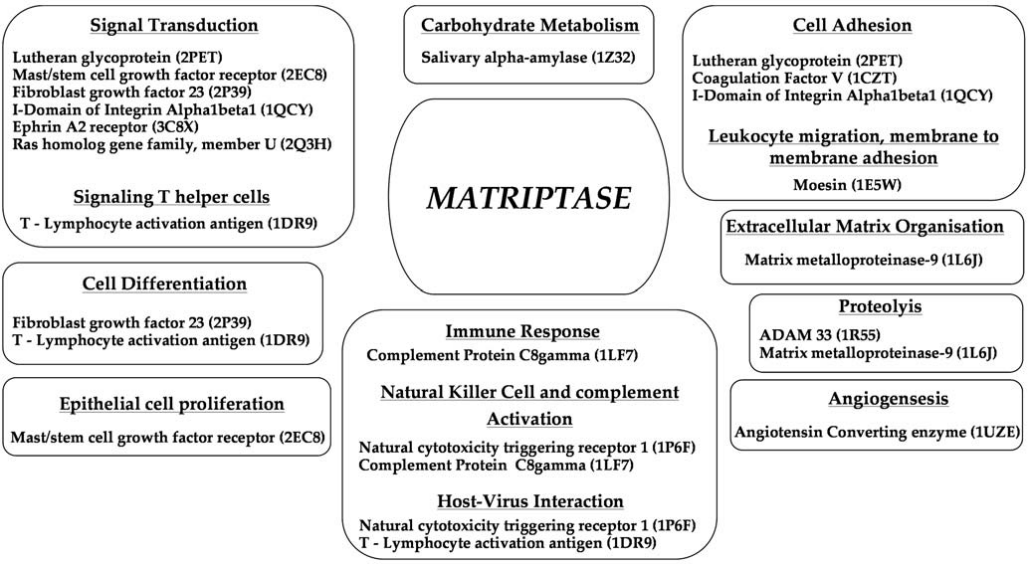
Assignment of functions to matriptase. Potential substrates of matriptase with rSASA ≥0.4 and with subcellular localization similar to matriptase were grouped based on their function.

After such stringent evaluation of the structure and localization, we set about deriving functional information about matriptase. The final 16 potential substrates were grouped based on their GO function [40]. The results (**Figure 4**) show that our method has identified proteins involved in cell adhesion, matrix/membrane organization, cell proliferation and differentiation as potential substrates. Matriptase is presumably involved in these functions [31], [32], [35]. However, only one or two substrates associated with such functions have been reported to posses the putative cleavage sequence for matriptase. We have identified more potential substrates of matriptase in these functional categories. Furthermore, by grouping several potential substrates, role played by a protease can be discerned or new functions assigned far more confidently than when dealing with isolated substrates. By such an analogy, we have identified regulation of carbohydrate metabolism and immune response as novel functions of matriptase. Although matriptase has been shown to be present in immune cells [41] and predicted to have some role in thymic homeostasis [42], its general role as modulator of immune response is not well documented. When more proteins involved in a common biological role harbor an accessible cleavage site for the protease and share the subcellular loci (and cell type and tissue distribution), then, it is reasonable to assume a role for the protease in modulating such a biological function.

Global prediction methods often over represent positive candidates which fail the acid test of *in vivo* relevance. Subjective validation of the kind described here may be used as an index of *in vivo* relevance. While absolute correlation should await experimental validation, we have devised means to restrict false positive identifications. We believe that protein conformation is an extremely critical parameter in this regard since this criterion can be used to eliminate those proteins with inaccessible sites. However a cleavable sequence may be exposed due to any of the following modifications: (a) exosite binding [37], [43], (b) post-translational changes, (c) binding of allosteric effectors, (d) unfolding by chaperones and (e) cleavage of a well accessible alternate site by the same or a different protease. Alternatively, a cleavable sequence may not be accessible because of protein-protein interactions, steric hindrance due to the presence of a disulfide bond or amino acid modification to name a few. A combination of three parameters-sequence specificity, 3D structural information and experimental data would be extremely valuable in more precise positive identification of an enzyme-substrate pair. As more and more structures are determined by structural consortiums worldwide, our ability to use this information in the identification of novel substrates of proteases in general will become more reliable and such information can be very vital in eliminating experimental artifacts and to extrapolate *in vitro* observations to normal physiological conditions.

### IV Proteolytic network

Exposure of a previously inaccessible cleavage site by the action of a protease raises interesting possibilities in functional regulation and creating a reaction cascade. For example, an inaccessible site in the protein could be exposed by the action of the same protease or a different protease acting elsewhere which could impart new function or help in terminating the function. We thought that such enzyme substrate pairs could be linked via the criterion of relative accessibility to build novel networks. Webs emanating from such a network can connect the proteolytic world and other protein regulatory networks like signal transduction, development, differentiation and apoptosis.

**Figure 5:**
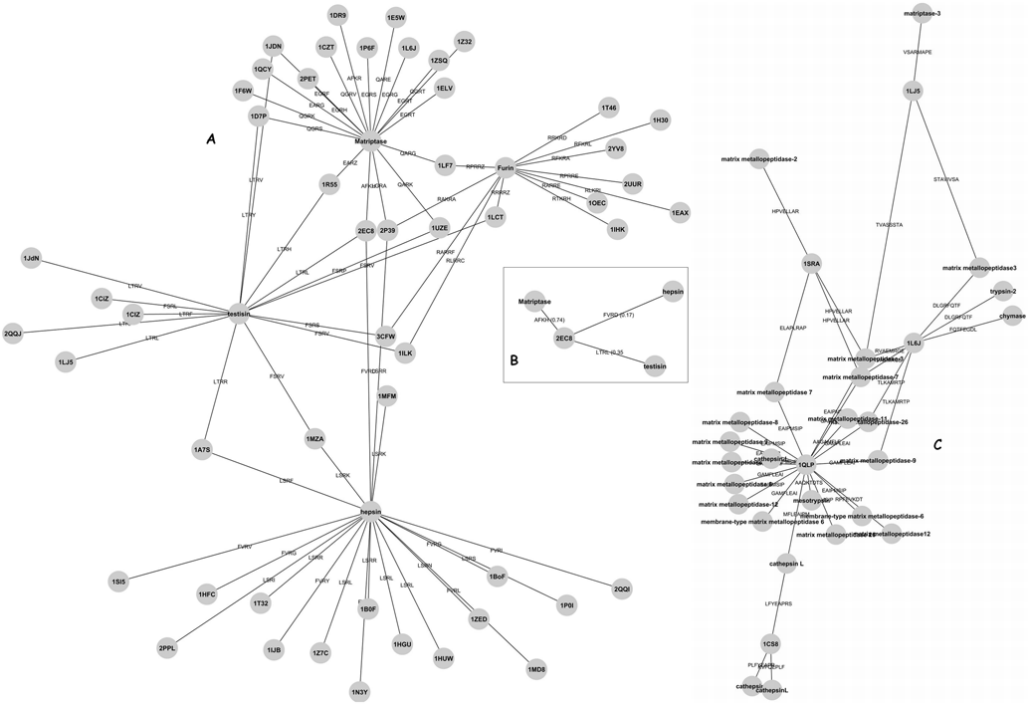
A proteolytic network based on rSASA values. A) Network was built using the program Cytoscape by linking substrates of furin, hepsin, matriptase and testisin derived from the PDB database. Inset B shows one substrate which is differentially accessible to the three enzymes. C) A network was similarly built using some of the proteases and their experimentally identified natural substrates.

In order to provide such novel insights we built a network of proteases and substrates derived from PDB. Here we highlight a small network formed by substrates of matriptase and furin which share the same subcellular loci as the enzyme. We added two other extracellular enzymes hepsin and testisin, into the network (**Table S9**) and only those putative substrates common to furin and matriptase were included (**Figure 5A**). Due to paucity of structural information, the network is not well developed and has limited nodes. One example is highlighted in **Figure 5** **(inset B)**. Mast/stem cell growth factor receptor (2EC8) is best accessible to matriptase (rSASA 0.74). Hepsin (a type II transmembrane protease) and testisin (a GPI anchored serine protease) also have cleavage sites on 2EC8 with rSASA values of 0.35 and 0.17 respectively. All three cleavage sites are in the extracellular domain. Hypothetically, if all these enzymes were to cleave 2EC8, then cleavage by testisin may require prior cleavage by matriptase. We also built network from natural substrates used earlier to determine rSASA values (**Table S5**). This network links metallo, cysteine and serine protease families (**Figure 5C**). It is anticipated that over the time when more structures are solved, these networks will be fully appreciated and novel information would be derived.

## Discussion

We believe that our method would be a very useful complementary approach in the current day attempts at global profiling of proteases and their substrates. The power of the method lies in the use of short peptide motifs which on one hand are big enough to provide specificity and on the other hand, small enough to cover a broad spectrum of proteins and most importantly the use of physiologically relevant filters namely, accessibility in terms of folded structure of a protein and subcellular localization.

We have chosen to use the artificial peptide substrates for each protease to create a subset of query sequence to demonstrate how the method in combination of physiologically relevant filters can actually throw out the possible number of false positive hits. We have illustrated this clearly using the example of matriptase, in which case only ∼2% of the original hits turn out to be potential substrates. Again as illustrated before, additional sites within the same protein for example in VGF1, VGF2 or uroplasminogen activator are present within the cytoplasmic side of the membrane and will not be cleaved by matriptase. Despite such filters, some of the candidates may never be cleaved by the cognate protease. Such false positive identifications however, are common to any such global profiling attempts including experimental approaches.

In addition, there could be many potential substrates that are not identified by our method. Our query data set represents 86% of the total tripeptide substrates indicating potentially high sample coverage. Nevertheless, alternative specificity determinants represented by amino acid occupancy for example at P2P1P1′, is not covered.

Proteases are also known to recognize additional binding sites called the ‘exosite’ [37], [43]. This additional specificity determinant could play a very important role in discriminating between different cleavage sites in the same protein, or between substrates and is not dealt with in the current method. Such discrimination could be an important determinant in cases where overlapping specificities are possible among enzymes of the same catalytic type [35]. Proteases also cleave substrates present in cellular compartments other than their own based on the physiological demand or during malignancy [44]–[46]. By demanding colocalization, our method misses out on such substrates. In order to account for the possibility that an authentic substrate could actually be in a different compartment, we must know the pattern of distribution of the protease in different compartments, in various tissues and their regulated expression. Until substantial information becomes available, a prerequisite in terms of subcellular co-localization (and tissue distribution) and topology in the case of membrane proteins ensures reliability of the prediction method. Sequence specificity and surface accessibility are relatively broad criteria that can include many such non-obvious or unconventional substrates.

We also imagine the use of an additional filter based on structural criteria. Whether all the different target sequences identified for a protease or the same sequence present on different substrates would fit in the active site of the enzyme? While a predetermined geometry could actually help in surface complementarity, a sequence of different topology could very well be induced to fit the active site. It will be useful to come up with a method that can predict whether a particular cleavable sequence in the intact protein would fit in the enzyme active site. Those sequences that cannot fit into the active site then can be excluded as an unlikely substrate. Our attempt at modeling various peptide sequences at the active site of matriptase and grading them based on Glide score is a small step in this direction.

The accuracy of the prediction method largely depends on the sequence information available to us. When stringent specificity information becomes available for as many enzymes as possible, accuracy of the prediction will also increase. We believe that rigorous determination of position specific information within each catalytic type, family and for each protease is necessary in this regard. As an illustrative example, we looked at two structures one of granzyme B and the other of matriptase, both of which belong to the S1 family. Granzyme B has a clear preference for Asp at the P1 position while matriptase shows a preference for Arg/Lys. When the matriptase and granzyme B structures were superposed, the overall fold of the two proteins was grossly similar. However, the active site differences in the two structures are considerable both in terms of the loop conformation of the residues (Gly216 to Gly226) as well as the sequence of the active site residues. For example, a crucial difference is in Asp189 being replaced by Thr in granzyme B. This alone will cut down the interaction energy of a matriptase substrate considerably within the granzyme B active site. In addition, there are other differences between the two structures - Gly226 in matriptase is replaced by an Arg residue - a huge difference that will also contribute significantly to the steric hindrance of the ligand in the active site. Also, Cys191 is replaced by Phe, resulting in a breakage of a key disulfide bond which probably is responsible for a lot of changes in the loop conformations around the active site in granzyme B. These observations, together with our docking results with matriptase and the various short peptide sequences illustrate how structural information at high resolution can help in understanding enzyme specificity.

Although we have used our method to extract substrates of serine proteases that belong to human, we have demonstrated the suitability of our method to enzymes of three other catalytic types as well. From the PDB, we have been able to identify natural substrates harboring the octapeptide cleavage sequences reported in MEROPS. Substrates of enzymes of all major catalytic type are represented (**Table S5**) indicating that the method in principle is applicable to endo proteases in general. In most instances, the substrates for the human proteases would be the cognate human proteins only, as in the case of the enzymes of the digestive system, while in other instances this may include those of pathogenic organisms [47]. It is quite likely that such sequence specific information and accessibility in the context of the folded structure of a protein could be important in determining cleavage of human proteins by viral endo proteases under certain conditions [48]–[50]. Although the approach described here is useful for endo proteases of any species or catalytic type, exceptions could be compartmentalized enzymes such as the ATP dependent proteases like proteasomes which are presumed to unfold a protein prior to degradation. Any 3D structural information is expected to be destroyed well before the polypeptide reaches the catalytic chamber and therefore surface accessibility may be irrelevant in such cases. Sequence specific information may still be useful as selective inhibitors have been designed for the different catalytic sites within the proteolytic chamber [51]. Cleavage specificity may also be dependent on many other factors.

Our method is probably not applicable to the following class of enzymes: exopeptidase, amino and carboxy peptidases, oligopeptidases, tripeptidyl-peptidase and dipeptidases. Method is also not applicable to enzymes involved in antigen processing and presentation like those of endoplasmic reticulum associated amino peptidase (ERAP) or thimmet oligo peptidase which are so far known to cleave peptide products (which in general lack any defined structure) generated upstream by the proteasome. We had also made note of the following: our method is probably irrelevant for many of the endo proteases present in the lysosomes. While the structural information may not be as relevant as for say cytosolic enzymes due to the acidic and denaturing environment milieu of the lysosomes, the dependence on sequence specificity is unclear. Even though we have used all the octapeptide cleavage sequences from MEROPS, identification of substrates for lysosomal enzymes like cathepsins may be treated as illustrative examples only.

**Figure 6:**
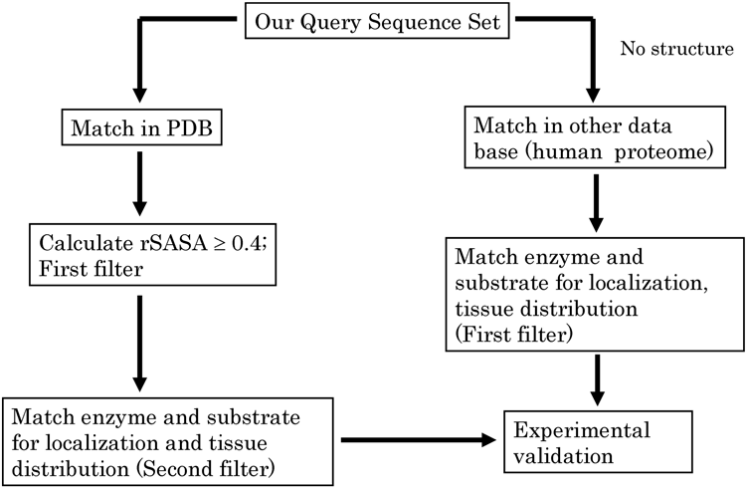
Flow chart of the method. A schematic of our method for *in silico* screening of natural substrates of proteases is presented. If no structure is available, colocalization can be used as the filter to identify putative substrates especially with the octapeptide as the QSS. Eventually however experimental validation is inevitable.

In essence, we believe that we have come up with a simple method to identify natural substrates of proteases using short sequence motifs for initial screening (**Figure 6**). We identify accessibility and subcellular localization as determinants of physiological relevance. Using three dimensional structure of the protein as a guide, we demonstrate novel network which can integrate the world of proteolysis and other regulatory networks. The basic principles suggested here can be extended to the study of other endo proteases from human and other organisms as well. We plan to deposit our results at our website, expand the approach to other proteases and provide a server for search options.

## Methods

Perl scripts were used for extraction (flat file/xml format) and analysis of the data from databases. These programs were run on Linux machine and/or Windows machine with ActivePerl installed (version 5.8.8). The data and the results produced by the scripts were stored in the CSV format files for statistical analysis.

### Analysis of cleavage site specificity

All the entries belonging to Homo sapiens from MEROPS Release 7.70 (22^nd^ January 2008) were extracted and analyzed. The cleavage site sequences reported for natural substrates of various proteases in the MEROPS database were extracted and propensities for each amino acid at every position (P4′-P4) were calculated.

### Extraction of cleavage sequences from MEROPS

Cleavage sequences reported in protein substrates and small peptide substrates reported for aspartate, cysteine, metallo and serine proteases in the MEROPS database were extracted.

### Validation of method

In order to validate our method, we considered two proteases; furin and thrombin. In MEROPS, 27 and 98 known substrates are reported for furin and thrombin respectively. We divided the known substrates into two sets i.e. a training set and a test set with 16 and 55 octapeptides randomly chosen to represent the training set for furin and thrombin respectively. These octapeptides were then scanned against the test set which comprised of the uniprot sequences of all serine protease substrates reported in MEROPS. We then used a SQL query to extract P3P2P1P1′ (tetrapeptide query set) and P3P2P1 peptides (tripeptide query set) from the octapeptides. These were again matched with the test set.

### Extraction of potential substrates

The human proteome data (NCBI refseq down loaded on Sep01 2008) and proteins with short stretches of disordered regions from DisProt were downloaded to a local database. A structural database of 772 proteins was constructed by placing a query on the website www.rcsb.org (on 21^st^ May 2008) for single chain proteins with >100 amino acids that belong to the taxonomy class Homo sapiens. Structures with <3 Å resolution and those with >95% homology were not considered. Even though single chain criterion was used, some of the proteins turned out to be part of a protein complex. They were retained in the analysis.

### Surface accessibility calculations

SASA values were calculated for each potential substrate using the POPS (Parameter OPtimized Surfaces) algorithm. Relative SASA (rSASA) was calculated based on the formula given by [52].

### Modeling of substrate binding to matriptase

The protein structure in the PDB entry 2GV6 was prepared using the protein preparation wizard in the Schrödinger software graphical user interface Maestro (version 8.5). Preliminary models of AEGRS with the terminal capping groups of ACE (acetamide - N terminus) and NME (N-methyl - C terminus) were built with random conformations using the “Builder” tool in Maestro (v8.5) (MAESTRO: A Graphical User Interface for Schrödinger Suite of products (v8.5) developed and marketed by Schrödinger LLC., 120 W. 45th Street, New York NY 10036). After further modifications detailed in the supplemental section, the tetrahedral carbon was marked for covalent bonding to O^γ^ atom of Ser195. Subsequently, the conformations of the pentapeptide ligand and covalently linked Serine residue were varied in the energy optimization process as described in the supplemental section (**Text S1**).

### Compartmentalization of protease-substrate

The cellular localization for each protease and substrates (proteins in the structural database) were gathered from GO terms [40] at the PDB site and verified again at http://www.uniprot.org/.

### Construction of Biological Network

Biological network was constructed in Cytoscape [53] for visualizing the interaction between a protease and its substrates. Proteases and their substrates serve as the nodes. Our network is based on the rSASA values. If the rSASA value is high, the substrate has more chance of being cleaved by a protease and hence such substrates are positioned close to the protease, whereas those that have low rSASA value are positioned far away from the protease.

All snapshots in this study were created using PYMOL (DeLano, W.L., The PyMOL Molecular Graphics System (2002) DeLano Scientific, San Carlos, CA USA).

## Supporting Information

Text S1 Modelling Substrate Binding to Matriptase
(0.05 MB DOC)

Table S1 Query Sequence Set derived from MEROPS
(0.31 MB PDF)

Table S2 Natural Substrates of Furin and Thrombin identified using octapeptide training set
(0.54 MB PDF)

Table S3 Putative Substrates of Aspartate,Cysteine and Serine catalytic type of proteases obtained by matches with Human proteome
(0.48 MB PDF)

Table S4 Putative Substrates of Human Proteome Queried with Tripeptide (P3P2P1) Motif
(4.20 MB PDF)

Table S5 Relative Solvent Accessibility (rSASA) Values of Natural Substrates of Proteases
(0.07 MB PDF)

Table S6 Potential Substrates of Serine Proteases from the PDB and DisProt
(0.38 MB PDF)

Table S7 Putative Substrates of Matriptase from PDB Rank Ordered According to rSASA
(0.08 MB PDF)

Table S8 Putative Substrates of Matriptase (rSASA >0.4) Filtered for Membrane/Extracellular Localization.
(0.06 MB PDF)

Table S9 Substrates of Furin, Hepsin, Matriptase and Testisin used in Building the Network
(0.03 MB PDF)
