# Supplementary material for: A Sequence and Structure Based Method to Predict Putative Substrates, Functions and Regulatory Networks of Endo Proteases
Source: PLoS One. 2009 May 27;4(5):e5700. doi: 10.1371/journal.pone.0005700 (PMC2683571; doi:10.1371/journal.pone.0005700)
Supplement: Text S1 — Modelling Substrate Binding to Matriptase (0.05 MB DOC) [file pone.0005700.s001.doc]

**Supplemental Method (Molecular Docking studies)**

The protein structure in the Protein Data Bank entry 2GV6 was prepared using the protein preparation wizard in the Schrodinger software graphical user interface Maestro (version 8.5). Bond orders were assigned to the ligand in the crystal structure while maintaining cationic states of its amidine and guanidine moieties. Hydrogen atoms were added to the protein-ligand complex consistent with physiologic pH (7.0). Thus, the Arg and Lys side chains were cationic while the Glu and Asp side chain carboxylates were anionic. Protein assignment was carried out to optimize the interactions between the side chains and the ligand atoms, as well the interactions between various side chains. Specifically, the protonation and flip states of the imidazole rings of the histidine residues were adjusted together with the side chain amides of Asn and Gln residues and the hydroxyl and S-H orientations to optimize such interactions. X-ray water molecules within 5 Angstroms of the bound ligand were retained during protein preparation, the last step of which was energy minimization of the entire complex. The minimization was terminated when the root mean square deviation of the heavy atoms in the energy minimized structure relative to the starting (X-ray) coordinates exceeded 0.3 Angstrom. This ensures that the integrity of the X-ray structure is preserved in further modeling studies while eliminating potential stereochemical short contacts that may exist in the protein structure. Furthermore, this process also ensures that the hydrogen atoms are placed in optimized geometries. The protein thus prepared was used for covalent docking of pentameric sequences as described below.

Preliminary models of the sequences AEGRS, PEGRS, FEGRS, KEGRS, YEGRS, REGRS, AEGRW and REGRW were built with random conformations using the "Builder" tool in Maestro (v8.5) (MAESTRO: A Graphical User Interface for Schrodinger Suite of products (v8.5) Developed and marketed by Schrodinger LLC., 120 W. 45^th^ Street, New York NY 10036) using the terminal capping groups of ACE (acetamide - N terminus) and NME (N-methyl - C terminus). This was followed by the modification of the carbonyl group of the 4th amino acid (Arg) to a CO- moiety in which the carbon is of tetrahedral type and the oxygen is anionic, to create an oxyanion. This tetrahedral carbon was marked for covalent bonding to the O^γ^ atom of Ser195 by uniquely identifying it via a SMARTS pattern not matched by any other carbon atom in the system. Upon the formation of the covalent bond, the conformations of the pentapeptide ligand and covalently linked Serine residue were varied in the energy optimization process, carried out using the Schrodinger protein modeling and refinement package PRIME. Here OPLS2005 force field with implicit solvation and a distance-dependent dielectric of 2R is employed. The exploration of the conformational space of the ligand and Ser195 is done using a procedure similar to that employed in loop refinement in PRIME [1]. In addition to the conformations, stereochemistry of the covalent linkage is also explored. In other words, both R and S configurations of the covalent linkage are explored. It turned out that in all the covalent dockings obtained in this investigation, the R configuration was found to be preferred over the S configuration. All the docked complexes were graphically visualized using the MAESTRO graphical user interface.

With a view to get a qualitative assessment of the binding of various pentapeptide ligands, the covalently docked ligand complexes were subjected to the following treatment to obtain a measure of the “docking score”. In each complex, the Ser195 was mutated to Gly195 after the bond between the O-gamma atom and the scissile carbonyl carbon was broken. Hydrogen was added to the latter atom to complete the valence structure of the ligand. This was followed by the generation of energy grids using the GLIDE protocol previously described [2-4] Herein, the docked ligand was used to define the energy grid boundaries with default options. These grids were employed to score the ligand “in place” with the XP scoring function. It should be noted that given the computational protocol followed for the generation of the covalent complexes, the XP GlideScore values can be used only in a qualitative sense to make judgments on the binding of various ligands.

1. Jacobson, M.P., et al., *A hierarchical approach to all-atom protein loop prediction.* Proteins, 2004. **55**(2): p. 351-67.

2. Friesner, R.A., et al., *Glide: a new approach for rapid, accurate docking and scoring. 1. Method and assessment of docking accuracy.* J Med Chem, 2004. **47**(7): p. 1739-49.

3. Friesner, R.A., et al., *Extra precision glide: docking and scoring incorporating a model of hydrophobic enclosure for protein-ligand complexes.* J Med Chem, 2006. **49**(21): p. 6177-96.

4. Halgren, T.A., et al., *Glide: a new approach for rapid, accurate docking and scoring. 2. Enrichment factors in database screening.* J Med Chem, 2004. **47**(7): p. 1750-9.
