# Supplementary material for: A Sequence and Structure Based Method to Predict Putative Substrates, Functions and Regulatory Networks of Endo Proteases
Source: PLoS One. 2009 May 27;4(5):e5700. doi: 10.1371/journal.pone.0005700 (PMC2683571; doi:10.1371/journal.pone.0005700)
Supplement: Table S1 — Query Sequence Set derived from MEROPS (0.31 MB PDF) [file pone.0005700.s002.pdf]

S1- Query Sequence Set derived from MEROPS

Artificial Query Sequence Set

P3P2P1

tryptase gamma 1	KWR	GPR				
chymase	FPF					
coagulation factor IXa	EGR					
coagulation factor XIa	EAR					
coagulation factor XIIa	QGR					
complement component C	LGR					
DESC1 peptidase	EGR	FVR				
elastase-1	APA	AAA				
elastase-2	AAA	AAV				
granzyme A	GPR					
granzyme B	AAD					
granzyme H	AAM	AAF	AAY	FLF		
hepsin	LSR	FVR				
kallikrein 1	PFR	VLR				
kallikrein-related peptida	PFR					
kallikrein-related peptida	VPR					
kallikrein-related peptida	ARR	PFR				
kallikrein-related peptida	KVY	RPY				
kallikrein-related peptida	VLK	VLR	PFR	VPR		
kallikrein-related peptida	QAR	FSR	VPR	GPR	GPK	
kallikrein-related peptida	RPY					
marapsin	VGR	GPR				
matriptase	EAR	EGR	QAR	QGR	LGR	AFK
matriptase-3	FVR					
mesotrypsin	GPR					
myeloblastin	APV					
u-plasminogen activator	LGR					
plasma kallikrein	QRR	PFR				
plasmin	GPK					
prostasin	GPR					
protein C (activated)	EPR					
testisin	LTR	FSR				
thrombin	VPR	GPR	FVR			
t-plasminogen activator	LGR					
tripeptidyl-peptidase I	AAF	GPM	FPA			
tripeptidyl-peptidase II	AAF					
trypsin-2	GPR					
tryptase beta	RNR	KAK	AKR			

Furin
RAKR
RCKR
RDKR
REKR
RFKR
RGKR
RHKR
RIKR
RKKR
RLKR
RMKR
RNKR
RPKR
RQKR
RRKR
RSKR
RTKR
RVKR
RWKR
RYKR
RARR
RCRR
RDRR
RERR
RFRR
RGRR
RHRR
RIRR
RKRR
RLRR
RMRR
RNRR
RPRR
RQRR
RRRR
RSRR
RTRR
RVRR
RWRR
RYRR

Tryptase beta
PAKR
PRNR
PKAK
PRNK

S1 Query Sequence Set (QSS)

Natural Query Sequence Set

Training Set

Furin

Octapeptide	Tetrapeptide	Tripeptide
IIRRSLPA	IRRS	IRR
RAKRFASL	AKRF	AKR
VFRRDAHK	FRRD	FRR
RSRRAATS	SRRA	SRR
RPKRYNSG	PKRY	PKR
RQKRSINL	QKRS	QKR
RENRCQCA	ENRC	ENR
RSKRCSCS	SKRC	SKR
AKQRAKRD	KQRA	KQR
RKRRSLGD	KRRS	KRR
RRRRQLDP	RRRQ	RRR
IRKRANSF	RKRA	RKR
VKKRSVSE	KKRS	KKR
HLKRDTED	LKRD	LKR
RGRRSLGS	GRRS	GRR
RKPRCGNP	KPRC	KPR

Thrombin

Octapeptide	Tetrapeptide	Tripeptide
SILRLAKA	ILRL	ILR
PHIRGNVG	HIRG	HIR
GRPRHQGV	RPRH	RPR
FGLRFYAY	GLRF	GLR
GHARLVHV	HARL	HAR
IAGRSLNP	AGRS	AGR
VSPRASAS	SPRA	SPR
MIVRRAIK	IVRR	IVR
MRQRALST	RQRA	RQR
PNARSQPS	NARS	NAR
SGQRGSSK	GQRG	GQR
AVARLSQR	VARL	VAR
KRGRAPQV	RGRA	RGR
PQGRIVGG	QGRI	QGR
IQIRSVAK	QIRS	QIR
IEPRSFSQ	EPRS	EPR
IKPRIVGG	KPRI	KPR
KAPRVATS	APRV	APR
KPKDSSVD	PKDS	PKD
PLPRKAPL	LPRK	LPR
GKLRSPFL	KLRS	KLR
PALRSKLQ	ALRS	ALR
GGVRGPRV	GVRG	GVR
GDIRGPRI	DIRG	DIR
FSARGHRP	SARG	SAR
NTGRSRGF	TGRS	TGR
GTARRYIG	TARR	TAR
VQPRAQKI	QPRA	QPR
SRLRAYLL	RLRA	RLR
AHPRIISA	HPRI	HPR
MVPRAVYL	VPRA	VPR
FRPKHTRI	RPKH	RPK
AFPRVKPA	FPRV	FPR
SDPRGFGH	DPRG	DPR
VKNRSVYI	KNRS	KNR
AQNRSYSK	QNRS	QNR
PSSRSPST	SSRS	SSR
KAVRALKN	AVRA	AVR
QWARLLQT	WARL	WAR

S1 Query Sequence Set (QSS)

AEPKMHKT	EPKM	EPK
AMARALVQ	MARA	MAR
AFSRGLLK	FSRG	FSR
ATPRLAST	TPRL	TPR
FNPRTFGS	NPRT	NPR
AVTRAKQI	VTRA	VTR
GSFRAGLF	SFRA	SFR
IDGRIVEG	DGRI	DGR
INVRKGGI	NVRK	NVR
ITVRGHNC	TVRG	TVR
KMLRGKPA	MLRG	MLR
PDLRSCVN	DLRS	DLE
PRSFLLRN	RSFL	RSF
QLGRIMLK	LGRI	LGR
VIPRSGGS	IPRS	IPR
YSNRSAAY	SNRS	SNR

S1 Query Sequence Set (QSS)

Furin

Octapeptide	Tetrapeptide	Tripeptide
RKKREITE	KKRE	KKR
RRKRYAIQ	RKRY	RKR
RRKRYAEH	RKRY	RKR
RKRRSVNP	KRRS	KRR
RRKRAIMS	RKRA	RKR
RQKRFVLS	QKRF	QKR
RHRRALDT	HRRA	HRR
RAKRSPKH	AKRS	AKR
AKRRAKRD	KRRA	KRR
RSKRALEN	SKRA	SKR
RKRRSTNE	KRRS	KRR
RSKRSLSC	SKRS	SKR

Thrombin

Octapeptide	Tetrapeptide	Tripeptide
LNARGLTS	NARG	NAR
KAVRALKN	AVRA	AVR
PNARSQPS	NARS	NAR
YGLRSKSK	GLRS	GLR
ITGRSRGF	TGRS	TGR
IFARGQSV	FARG	FAR
QSARARLS	SARA	SAR
TIPRAAIN	IPRA	IPR
LDPRSFLL	DPRS	DPR
SKGRSLIG	KGRS	KGR
PAPRGYPG	APRG	APR
MTPRSEGS	TPRS	TPR
MTPRSGGS	TPRS	TPR
MTPRSRGS	TPRS	TPR
LKLRTMLI	KLRT	KLR
QWARLLQT	WARL	WAR
AVARLSQR	VARL	VAR
QLGRIMLK	LGRI	LGR
YSNRSAAY	SNRS	SNR
LKPRVGKA	KPRV	KPR
YIPRILFL	IPRI	IPR
QGVRGYPT	GVRG	GVR
QGVRGYPT	GVRG	GVR
AEPRTVVS	EPRT	EPR
AVARLSQR	VARL	VAR
MIVRRAIK	IVRR	IVR
LRPRFKII	RPRF	RPR
VDPRLIDG	DPRL	DPR
GRPRHQGV	RPRH	RPR
GRPRHQGV	RPRH	RPR
GRPRHQGV	RPRH	RPR
GRPRHQGV	RPRH	RPR
GRPRHQGV	RPRH	RPR
GRPRHQGV	RPRH	RPR
MIVRRAIK	IVRR	IVR
MIVRRAIK	IVRR	IVR
LSPRTFHP	SPRT	SPR
WYLRSNTG	YLRS	YLR
QGVRGYPT	GVRG	GVR
LIVRSATK	IVRS	IVR
ARPRHPAE	RPRH	RPR
LSPRGVHI	SPRG	SPR
QSPRSFQK	SPRS	SPR

Octapeptide Cleavage Sequences

Serine Protease

thrombin	VSPRASAS
granzyme B	VAPDRLRF
kallikrein-related peptidase 2	IQSRIVGG
granzyme B	LGNDSRDM
mesotrypsin	AAQKTDTS
granzyme B	IGLDSSSI
chymase	RETYGEMA
granzyme B	VTPDQSMV
complement component activated C1s	SLGRKIQI
plasma kallikrein	TFHKAEYR
coagulation factor XIIa	TSTRIVGG
thrombin	VDPRLIDG
HtrA2 peptidase	VPRAVFVD
HtrA2 peptidase	VFVDLEPT
HtrA2 peptidase	HSDCAFMV
HtrA2 peptidase	VAEITNAC
HtrA2 peptidase	PEQLITGK
HtrA2 peptidase	GEGMEEGE
HtrA2 peptidase	VDLTEFQT
HtrA2 peptidase	FSETGAGK
HtrA2 peptidase	FVHWYVGE
HtrA2 peptidase	AFMVDNEA
HtrA2 peptidase	PKDVNAAI
HtrA2 peptidase	GFKVGINY
HtrA2 peptidase	PRAVFVDL
HtrA2 peptidase	AVFVDLEP
plasmin	KKPRCGVP
plasmin	DVGEYNVF
plasmin	GILKENAA
HtrA2 peptidase	PTLLTEAP
HtrA2 peptidase	TLLTEAPL
HtrA2 peptidase	SGLVKAGF
HtrA2 peptidase	LCYVALDF
elastase-2	HQGVMVGM
granzyme B	VGPDFGKK
granzyme B	DEVDNKVK
HtrA2 peptidase	WEVISDEH
HtrA2 peptidase	GFQLTHSL
HtrA2 peptidase	THSLGGGT
HtrA2 peptidase	TTCLRFPG
HtrA2 peptidase	LRKLAVNM

Metallo Protease

matrix metallopeptidase-12	PPLKLMHS
matrix metallopeptidase-12	PPLKLMHS
matrix metallopeptidase-9	GPPGVVGP
matrix metallopeptidase-3	GPPGLKGL
matrix metallopeptidase-9	GPPGIVIG
matrix metallopeptidase-7	GAMFLEAI
matrix metallopeptidase-8	GAMFLEAI
matrix metallopeptidase-8	EAIPMSIP
matrix metallopeptidase-9	GAMFLEAI
matrix metallopeptidase-9	EAIPMSIP
matrix metallopeptidase-11	AAGAMFLE
matrix metallopeptidase-7	EAIPMSIP
matrix metallopeptidase-12	RPFEVKDT
matrix metallopeptidase-12	GAMFLEAI
matrix metallopeptidase-12	EAIPMSIP
membrane-type matrix metallopeptidase-6	GAMFLEAI
membrane-type matrix metallopeptidase-6	EAIPMSIP
matrix metallopeptidase-26	GAMFLEAI
matrix metallopeptidase-26	EAIPMSIP
matrix metallopeptidase-3	EAIPMSIP
matrix metallopeptidase-3	DAASLKGL
matrix metallopeptidase-7	MLEDEASG
matrix metallopeptidase-7	HLRELHLD
membrane-type matrix metallopeptidase-1	AFTRANHL
matrix metallopeptidase-2	HPVELLAR
matrix metallopeptidase-2	TVAEVTEV
matrix metallopeptidase-3	HPVELLAR
matrix metallopeptidase-3	TVAEVTEV
matrix metallopeptidase-7	HPVELLAR
matrix metallopeptidase-7	ELAPLRAP
matrix metallopeptidase-9	NPVQVEVG
matrix metallopeptidase-13	NPVQVEVG
matrix metallopeptidase-13	GANPVQVE
matrix metallopeptidase-13	GPQGLAGQ
matrix metallopeptidase-2	GPQGLAGQ
matrix metallopeptidase-7	GGAQLGVM
matrix metallopeptidase-9	NFAAQMAG
matrix metallopeptidase-9	GHRGFTGL
matrix metallopeptidase-9	GARGIQGP
matrix metallopeptidase-9	GPQGARGF
matrix metallopeptidase-9	GPAGEEGK

HtrA2 peptidase	FPRLHFFM
HtrA2 peptidase	GHYTEGAE
HtrA2 peptidase	GNNWAKGH
HtrA2 peptidase	NHLVSATM
HtrA2 peptidase	FQLTHSLG
granzyme A	TAAKKNDK
chymase	FQTFEGDL
trypsin-2	DLGRFQTF
kallikrein-related peptidase 6	LRQRESSQ
chymotrypsin C	SLYQLENY
elastase-2	SHLVEALY
elastase-2	LVEALYLV
cathepsin G	GSHLVEAL
elastase-1	GERGFFYT
chymotrypsin C	ERGFFYTP
chymotrypsin C	RGFFYTPK
granzyme B	IEADFRLN
cathepsin G	EYEYLDYD
plasmin	RRKRIVGG
plasmin	ATNRRSFP
plasmin	RRFKLSDL
plasmin	LCCKACQG
plasmin	RRIKSLLP
plasmin	VGGKRAQL
plasmin	CLAKKYTH
plasmin	LAKKYTHL
plasmin	PDLKRIVI
plasmin	CQQKSLEC
elastase-2	GRAVTYSR
cathepsin G	AVTYSRSR
cathepsin G	RSRYLECI
u-plasminogen activator	NSGRAVTY
u-plasminogen activator	TYSRSRYL
granzyme B	ISSDRDLL
cathepsin G	VHFFKNIV
cathepsin G	LSRFSWGA
HtrA2 peptidase	TLKLTTPT
HtrA2 peptidase	TTPTYGDL
HtrA2 peptidase	GLKMASTF
coagulation factor XIIa	SMTRVVGG
plasma kallikrein	SLTRNGPL
plasma kallikrein	CGQRLRKS

matrix metallopeptidase-9	GARGLTGR
matrix metallopeptidase-9	GAPGLRGL
matrix metallopeptidase-9	GPPGLQGM
matrix metallopeptidase-9	GPTGVTGP
matrix metallopeptidase-9	GPKGARGD
matrix metallopeptidase-9	GQRGIVGL
matrix metallopeptidase-9	QLGVMQGP
matrix metallopeptidase-3	NFAAQMAG
matrix metallopeptidase-3	FAAQMAGG
matrix metallopeptidase-13	DIKDIVGP
matrix metallopeptidase-13	QLGVMQGP
membrane-type matrix metallopeptidase-1	AQLGVMQG
matrix metallopeptidase-2	GPKGQKGE
matrix metallopeptidase-13	GIQSLYGP
matrix metallopeptidase-3	DVGEYNVF
matrix metallopeptidase-13	GPSGLLAH
membrane-type matrix metallopeptidase-1	DVGEYNVF
membrane-type matrix metallopeptidase-1	NLAGILKE
matrix metallopeptidase-3	KPQQFFGL
matrix metallopeptidase-8	GPQGLLGA
matrix metallopeptidase-8	GPQGLLGA
matrix metallopeptidase-2	GPQGLLGA
matrix metallopeptidase-9	GPQGARGF
matrix metallopeptidase-3	QPDSVSIP
matrix metallopeptidase-3	QPDSVSIP
matrix metallopeptidase-3	DAASLKGL
matrix metallopeptidase-7	MLEDEASG
matrix metallopeptidase-7	HLRELHLD
matrix metallopeptidase-9	TLKAMRTP
matrix metallopeptidase-3	DLGRFQTF
matrix metallopeptidase-3	RVAEMRGE
matrix metallopeptidase-7	RVAEMRGE
matrix metallopeptidase-26	TLKAMRTP
matrix metallopeptidase-7	GAMFLEAI
matrix metallopeptidase-8	GAMFLEAI
matrix metallopeptidase-8	EAIPMSIP
matrix metallopeptidase-9	GAMFLEAI
matrix metallopeptidase-9	EAIPMSIP
matrix metallopeptidase-11	AAGAMFLE
matrix metallopeptidase-7	EAIPMSIP
matrix metallopeptidase-12	RPFEVKDT
matrix metallopeptidase-12	GAMFLEAI

kallikrein-related peptidase 3	QCLRYKKP
kallikrein-related peptidase 3	QCLMLNPP
granzyme B	VDPDYWEK
plasmin	SQRKHSKR
plasmin	KHSKRHIH
elastase-2	PNVILAPS
elastase-2	PTVIQVPS
elastase-2	MESTLLTT
plasmin	GNGKGYRG
granzyme B	IETDSGVD
chymase	KMLFVEPI
granzyme B	VSKDDSII
coagulation factor XIa	KLTRAETV
coagulation factor XIa	DFTRVVGG
u-plasminogen activator	SSSRGPYH
granzyme B	VDVDSGIE
kallikrein-related peptidase 2	TDVRAAVY
kallikrein-related peptidase 3	AAVYQPQP
kallikrein-related peptidase 3	VSVYTVKD
kallikrein-related peptidase 3	ITIYAVEE
kallikrein-related peptidase 2	GFRRTTPP
HtrA2 peptidase	VRLLSGDT
HtrA2 peptidase	PFALQNTI
HtrA2 peptidase	LQNTITSG
HtrA2 peptidase	GFLVFHSF
HtrA2 peptidase	LQDVYKIG
kallikrein-related peptidase 3	QSTNREQD
kallikrein-related peptidase 3	VQKDVSQS
kallikrein-related peptidase 3	VQKDVSQR
kallikrein-related peptidase 3	GHYQNVVE
kallikrein-related peptidase 3	SKVQTSLC
kallikrein-related peptidase 3	IPSQEQEH
kallikrein-related peptidase 3	QQLLHNKQ

matrix metallopeptidase-12	EAIPMSIP
membrane-type matrix metallopeptidase-6	GAMFLEAI
membrane-type matrix metallopeptidase-6	EAIPMSIP
matrix metallopeptidase-26	GAMFLEAI
matrix metallopeptidase-26	EAIPMSIP
matrix metallopeptidase-3	EAIPMSIP
matrix metallopeptidase-3	KPQQFFGL
matrix metallopeptidase-13	GPQGLAGQ
matrix metallopeptidase-2	GPQGLAGQ
matrix metallopeptidase-7	GGAQLGVM
matrix metallopeptidase-9	NFAAQMAG
matrix metallopeptidase-9	GHRGFTGL
matrix metallopeptidase-9	GARGIQGP
matrix metallopeptidase-9	GPQGARGF
matrix metallopeptidase-9	GPAGEEGK
matrix metallopeptidase-9	GARGLTGR
matrix metallopeptidase-9	GAPGLRGL
matrix metallopeptidase-9	GPPGLQGM
matrix metallopeptidase-9	GPTGVTGP
matrix metallopeptidase-9	GPKGARGD
matrix metallopeptidase-9	GQRGIVGL
matrix metallopeptidase-9	QLGVMQGP
matrix metallopeptidase-3	NFAAQMAG
matrix metallopeptidase-3	FAAQMAGG
matrix metallopeptidase-13	DIKDIVGP
matrix metallopeptidase-13	QLGVMQGP
membrane-type matrix metallopeptidase-1	AQLGVMQG
matrix metallopeptidase-2	GPKGQKGE
matrix metallopeptidase-9	LVEALYLV
matrix metallopeptidase-9	SLYQLENY
matrix metallopeptidase-9	GERGFFYT
matrix metallopeptidase-9	VNQHLCGS
matrix metallopeptidase-9	EALYLVCG

kallikrein-related peptidase 3	SSQYSNTE
kallikrein-related peptidase 3	LLVYNKNQ
kallikrein-related peptidase 3	SSIYSQTE
kallikrein-related peptidase 3	RSIYSQTE
kallikrein-related peptidase 3	GQHYSGQK
kallikrein-related peptidase 3	SIQYTYHV
kallikrein-related peptidase 3	SQQYDLNA
kallikrein-related peptidase 2	KAHRGTQN
kallikrein-related peptidase 2	EERRLHYG
kallikrein-related peptidase 2	VSQRSIYS
kallikrein-related peptidase 3	ISYQSSST
kallikrein-related peptidase 3	RLHYGENG
kallikrein-related peptidase 3	QSSYVLQT
complement component C2a	QLGRLHMK
enteropeptidase	DDDKIVGG
complement component activated C1s	GLQRALEI
complement factor I	STGRNGFK
complement factor I	HRGRTLEI
granzyme B	VEQDMFAH
chymase	PSQFNFVP
HtrA2 peptidase	HQRVKYTK
kallikrein-related peptidase 15	ILSRIVGG
elastase-2	LGMISLMK
elastase-2	SSRIGEIK
kallikrein 1	SPFRSSRI
kallikrein 1	ISLMKRPP
kallikrein-related peptidase 2	SLMKRPPG
thrombin	VVPRGVNL
granzyme B	IEADSESQ
plasmin	DVAQFVLT

matrix metallopeptidase-3	LVEALYLV
matrix metallopeptidase-7	LVEALYLV
matrix metallopeptidase-3	DAASLKGL
matrix metallopeptidase-7	MLEDEASG
matrix metallopeptidase-7	HLRELHLD
matrix metallopeptidase-3	MAASLKRP
matrix metallopeptidase-3	MAASLKRP
membrane-type matrix metallopeptidase-1	RRKRYAIQ
membrane-type matrix metallopeptidase-1	PQPRTTSR
membrane-type matrix metallopeptidase-1	FCIQNYTP
matrix metallopeptidase-3	MAASLKRP
matrix metallopeptidase-3	MAASLKRP
matrix metallopeptidase-13	TCVAYVAT
matrix metallopeptidase-13	TCVAYVAT
membrane-type matrix metallopeptidase-1	TCVAYVAT
matrix metallopeptidase-26	SKPNMIDA
matrix metallopeptidase-9	LSRFSWGA
matrix metallopeptidase-3	PPAPVQAK
matrix metallopeptidase-12	VVGGLVAL
matrix metallopeptidase-13	VVGGLVAL
matrix metallopeptidase-12	GLVALRGA
matrix metallopeptidase-12	KEHKYKAE
matrix metallopeptidase-13	EPKKVKDH
matrix metallopeptidase-3	HLGGAKQV
matrix metallopeptidase-13	TCVAYVAT
matrix metallopeptidase-13	TCVAYVAT
membrane-type matrix metallopeptidase-1	TCVAYVAT
matrix metallopeptidase-26	SKPNMIDA
matrix metallopeptidase-12	VPPNVILA
matrix metallopeptidase-2	GPGGAWAA

chymase	QFVLTEGN
chymase	PSVWAAVP
elastase-2	RGSVILTV
plasmin	GEARGSVI
HtrA2 peptidase	LFQTSHTL
HtrA2 peptidase	LLHVAALG
cathepsin G	GLARSNLD
cathepsin G	RSNLDEDI
HtrA2 peptidase	IDKIAFTG
corin	TAPRSLRR
trypsin-2	ETMRKPRC
elastase-2	PETAMSTV
elastase-2	ATTANYDD
elastase-2	LGPVTPEI
HtrA2 peptidase	LTQQVFDA
HtrA2 peptidase	LDRISVYY
HtrA2 peptidase	AILVDLEP
thrombin	FSARGHRP
elastase-2	PMAVVQSV
elastase-2	PVPVYAFS
complement factor I	LPSRSSKI
complement factor I	SLLRSEET
coagulation factor XIIa	IKPRIVGG
coagulation factor XIIa	PMKRLTLG
HtrA2 peptidase	ILPIKFPH
HtrA2 peptidase	TYSLGSAL
thrombin	FGLRFYAY
plasmin	KGYRSQRG
granzyme B	LGPDESKQ
elastase-2	LEKIFSED
elastase-2	VTTVGFMP
elastase-2	GKLTTVFL
elastase-2	IGKVDGTS
elastase-2	TSHVTGKG
elastase-2	GKGVTVET
elastase-2	VETVFSVD
elastase-2	LTTVFLPI
cathepsin G	GRSLIGKV
cathepsin G	ETVFSVDE
cathepsin G	VDEFSASV
myeloblastin	FSVDEFSA
myeloblastin	LTGKLTTV
myeloblastin	TVETVFSV

matrix metallopeptidase-3	AAEVISNA
matrix metallopeptidase-2	GPKGQKGD
matrix metallopeptidase-9	GPKGQKGD
matrix metallopeptidase-20	MGGWLHHQ
matrix metallopeptidase-7	GAMFLEAI
matrix metallopeptidase-8	GAMFLEAI
matrix metallopeptidase-8	EAIPMSIP
matrix metallopeptidase-9	GAMFLEAI
matrix metallopeptidase-9	EAIPMSIP
matrix metallopeptidase-11	AAGAMFLE
matrix metallopeptidase-7	EAIPMSIP
matrix metallopeptidase-12	RPFEVKDT
matrix metallopeptidase-12	GAMFLEAI
matrix metallopeptidase-12	EAIPMSIP
membrane-type matrix metallopeptidase-6	GAMFLEAI
membrane-type matrix metallopeptidase-6	EAIPMSIP
matrix metallopeptidase-26	GAMFLEAI
matrix metallopeptidase-26	EAIPMSIP
matrix metallopeptidase-3	EAIPMSIP
matrix metallopeptidase-2	NSFGLRFG
membrane-type matrix metallopeptidase-1	NSFGLRFG
membrane-type matrix metallopeptidase-3	NSFGLRFG
membrane-type matrix metallopeptidase-1	AFTRANHL
membrane-type matrix metallopeptidase-3	RVEAMLND
matrix metallopeptidase-26	SPVAVSQS
matrix metallopeptidase-3	PFSPLVAT
matrix metallopeptidase-26	SPVAVSQS
matrix metallopeptidase-3	PFSPLVAT
matrix metallopeptidase-26	SPVAVSQS
matrix metallopeptidase-3	PFSPLVAT
matrix metallopeptidase-26	SPVAVSQS
matrix metallopeptidase-2	GPKGQKGE
matrix metallopeptidase-9	GPPGVVGP
matrix metallopeptidase-3	PFSPLVAT
matrix metallopeptidase-26	SPVAVSQS
matrix metallopeptidase-3	PFSPLVAT
matrix metallopeptidase-26	SPVAVSQS
matrix metallopeptidase-9	GPGGVVGP
matrix metallopeptidase-3	ESGDYKAT
matrix metallopeptidase-3	PFSPLVAT
matrix metallopeptidase-26	SPVAVSQS
matrix metallopeptidase-9	LPVNVTDY
matrix metallopeptidase-12	EGFSLSSS

myeloblastin	KLTTVFLP
myeloblastin	GVTVETVF
myeloblastin	VFSVDEFS
thrombin	SKGRSLIG
plasmin	RSSKGRSL
elastase-2	ATNATLDP
elastase-2	QLPAFISE
elastase-2	YRLVSINK
cathepsin G	LDPRSFLL
cathepsin G	PRSFLLRN
cathepsin G	YEPFWEDE
cathepsin G	LTEYRLVS
myeloblastin	SEDASGYL
myeloblastin	LRNPNDKY
myeloblastin	KYEPFWED
thrombin	ARTRARRP
plasmin	TEYRLVSI
plasmin	PESKATNA
plasmin	SINKSSPL
matriptase-3	VSARMAPE
coagulation factor Xa	AFWKTDAS
cathepsin G	PFDLLDFN
coagulation factor IXa	NLTRIVGG
plasmin	THEKGRQS
plasmin	PKAKSHAP
granzyme B	ICPDMYIN
coagulation factor XIIa	PQGRIVGG
chymase	VVPYGLGS
cathepsin G	FCHLDIIW
cathepsin G	CVYFCHLD
cathepsin G	DIIWVNTP
thrombin	YGLRSKSK
granzyme B	VTPDPERW
kallikrein-related peptidase 6	AEFRHDSG
kallikrein-related peptidase 6	HHQKLVFF
kallikrein-related peptidase 6	SEVKMDAE
HtrA1 peptidase	MQQNGYEN
HtrA1 peptidase	VKMDAEFR
HtrA1 peptidase	VHHQKLVF
HtrA1 peptidase	GYEVHHQK
myeloblastin	KRFALLGD
matriptase-3	AMSRMSLS

matrix metallopeptidase-2	GPKGQKGE
matrix metallopeptidase-9	GPPGMPGV
matrix metallopeptidase-9	GPPGVVGP
matrix metallopeptidase-2	GPKGQKGE
matrix metallopeptidase-3	QPDAINAP
matrix metallopeptidase-3	QPDAINAP
membrane-type matrix metallopeptidase-1	GEYRTNPE
membrane-type matrix metallopeptidase-1	SSERSSTS
matrix metallopeptidase-7	GAMFLEAI
matrix metallopeptidase-8	GAMFLEAI
matrix metallopeptidase-8	EAIPMSIP
matrix metallopeptidase-9	GAMFLEAI
matrix metallopeptidase-9	EAIPMSIP
matrix metallopeptidase-11	AAGAMFLE
matrix metallopeptidase-7	EAIPMSIP
matrix metallopeptidase-12	RPFEVKDT
matrix metallopeptidase-12	GAMFLEAI
matrix metallopeptidase-12	EAIPMSIP
membrane-type matrix metallopeptidase-6	GAMFLEAI
membrane-type matrix metallopeptidase-6	EAIPMSIP
matrix metallopeptidase-26	GAMFLEAI
matrix metallopeptidase-26	EAIPMSIP
matrix metallopeptidase-3	EAIPMSIP
matrix metallopeptidase-2	QLGTYNVI
matrix metallopeptidase-2	CINYEAFV
matrix metallopeptidase-3	DVAQFVLT
matrix metallopeptidase-1	PVQPIGPQ
matrix metallopeptidase-1	DVAQFVLT
matrix metallopeptidase-1	VAQFVLTE
matrix metallopeptidase-1	DAETLKVM
matrix metallopeptidase-7	DVAQFVLT
matrix metallopeptidase-9	GPPGVVGP
matrix metallopeptidase-3	QPVGINTS
matrix metallopeptidase-2	QPVGINTS
matrix metallopeptidase-13	QPVGINTS
membrane-type matrix metallopeptidase-1	QPVGINTS
matrix metallopeptidase-3	QPVGINTS
matrix metallopeptidase-2	GPKGQKGE
matrix metallopeptidase-2	GPKGQKGE
matrix metallopeptidase-9	GPPGMPGV
matrix metallopeptidase-7	GAMFLEAI
matrix metallopeptidase-8	GAMFLEAI

HtrA2 peptidase	LLLVANAG
thrombin	GGVRGPRV
kallikrein-related peptidase 3	RRLNSGEK
kallikrein-related peptidase 3	GHYQNVVD
kallikrein-related peptidase 3	SKLQTSLH
kallikrein-related peptidase 3	SQNQVTIH
kallikrein-related peptidase 3	IPSQAQEY
kallikrein-related peptidase 3	MSYQSSST
kallikrein-related peptidase 3	SKQHLGGS
kallikrein-related peptidase 3	RLNYGGKS
kallikrein-related peptidase 2	TEKRLWVH
kallikrein-related peptidase 2	HQDRLQHG
kallikrein-related peptidase 2	PSSRTEER
kallikrein-related peptidase 2	NQVRIPSQ
kallikrein-related peptidase 2	EERRLNYG
kallikrein-related peptidase 2	QKGRYKQE
kallikrein-related peptidase 2	GHFHMIVI
kallikrein-related peptidase 3	HPAHQDRL
kallikrein-related peptidase 3	RQLHHGEK
kallikrein-related peptidase 3	SIQHTYHV
kallikrein-related peptidase 3	GQHYFGQK
granzyme B	VSWDSGGS
granzyme B	VGPDGLVY
complement component activated C1r	EKQRIIGG
elastase-2	RFKIIGGE
cathepsin G	PRFKIIGG
thrombin	LRPRFKII

matrix metallopeptidase-8	EAIPMSIP
matrix metallopeptidase-9	GAMFLEAI
matrix metallopeptidase-9	EAIPMSIP
matrix metallopeptidase-11	AAGAMFLE
matrix metallopeptidase-7	EAIPMSIP
matrix metallopeptidase-12	RPFEVKDT
matrix metallopeptidase-12	GAMFLEAI
matrix metallopeptidase-12	EAIPMSIP
membrane-type matrix metallopeptidase-6	GAMFLEAI
membrane-type matrix metallopeptidase-6	EAIPMSIP
matrix metallopeptidase-26	GAMFLEAI
matrix metallopeptidase-26	EAIPMSIP
matrix metallopeptidase-3	EAIPMSIP
matrix metallopeptidase-3	RAIHIQAE
matrix metallopeptidase-2	RAIHIQAE
matrix metallopeptidase-2	GPHLLVEA
matrix metallopeptidase-10	RAIHIQAE
matrix metallopeptidase-10	GPHLLVEA
matrix metallopeptidase-7	GPHLLVEA
matrix metallopeptidase-9	RAIHIQAE
matrix metallopeptidase-3	RAIHIQAE
matrix metallopeptidase-9	RAIHIQAE
membrane-type matrix metallopeptidase-3	GPLGIAGI
membrane-type matrix metallopeptidase-3	GIAGITGA
matrix metallopeptidase-8	IPENFFGV
matrix metallopeptidase-8	TSEDLVVQ
matrix metallopeptidase-8	TEGEARGS

u-plasminogen activator	VSNKYFSN
plasmin	ADGKKPSS
epitheliasin	RQSRIVGG
matriptase	RKGKAGAA
kallikrein-related peptidase 3	SQRYKVDY
thrombin	SRLRAYLL
thrombin	LSPRGVHI
plasmin	AVSRLRAY
plasmin	DSQRYKVD
plasmin	HPLHSKII
plasmin	QRYKVDYE
plasmin	NHLKFLNV
plasmin	GFYKKKQC
elastase-2	IIIIKKGH
elastase-2	PRGVHIPN
cathepsin G	FHPLHSKI
cathepsin G	HLKFLNVL
matriptase-3	FTFRSARL
myeloblastin	YLPTGPRR
granzyme B	IQADSGPI
HtrA2 peptidase	PSQISYPA
granzyme B	IEPDTDAP
thrombin	VQPRAQKI
chymase	LKSLSQQI
chymase	DRVYIHPF
elastase-2	FHLVIHNE
cathepsin G	IHPFHLVI
complement component activated C1r	QRQRIIGG
cathepsin G	DTDLYDYY
coagulation factor XIa	KQLRVVNG
plasma kallikrein	DLHRHIFW
granzyme B	VDSDEEEE
coagulation factor Xa	FNPRTFGS
coagulation factor Xa	IDGRIVEG
thrombin	MTPRSEGS
thrombin	PDLRSCVN
elastase-2	RSSASVPK
myeloblastin	GKDCRDGF
granzyme B	LEEDAEMK
matriptase-3	IAGRSLNP
elastase-2	AVVIAGRS

matrix metallopeptidase-1	IPENFFGV
matrix metallopeptidase-9	IPENFFGV
matrix metallopeptidase-7	IPENFFGV
matrix metallopeptidase-7	TSEDLVVQ
matrix metallopeptidase-3	IPENFFGV
matrix metallopeptidase-3	ARGSVILT
matrix metallopeptidase-13	IPENFFGV
matrix metallopeptidase-13	TSEDLVVQ
membrane-type matrix metallopeptidase-1	IPENFFGV
membrane-type matrix metallopeptidase-1	TSEDLVVQ
matrix metallopeptidase-20	IPENFFGV
matrix metallopeptidase-19	IPENFFGV
matrix metallopeptidase-3	DSGGFMLT
matrix metallopeptidase-3	DSGGFMLT
matrix metallopeptidase-8	SSNPIQPT
matrix metallopeptidase-2	DVANYNFF
matrix metallopeptidase-13	ERPIRNSV
matrix metallopeptidase-8	IPENFFGV
matrix metallopeptidase-8	TSEDLVVQ
matrix metallopeptidase-8	TEGEARGS
matrix metallopeptidase-1	IPENFFGV
matrix metallopeptidase-9	IPENFFGV
matrix metallopeptidase-7	IPENFFGV
matrix metallopeptidase-7	TSEDLVVQ
matrix metallopeptidase-3	IPENFFGV
matrix metallopeptidase-3	ARGSVILT
matrix metallopeptidase-13	IPENFFGV
matrix metallopeptidase-13	TSEDLVVQ
membrane-type matrix metallopeptidase-1	IPENFFGV
membrane-type matrix metallopeptidase-1	TSEDLVVQ
matrix metallopeptidase-20	IPENFFGV
matrix metallopeptidase-19	IPENFFGV
matrix metallopeptidase-2	GPGGAWAA
matrix metallopeptidase-7	GAMFLEAI
matrix metallopeptidase-8	GAMFLEAI
matrix metallopeptidase-8	EAIPMSIP
matrix metallopeptidase-9	GAMFLEAI
matrix metallopeptidase-9	EAIPMSIP
matrix metallopeptidase-11	AAGAMFLE
matrix metallopeptidase-7	EAIPMSIP
matrix metallopeptidase-12	RPFEVKDT

cathepsin G	PVSLSYRC
HtrA2 peptidase	GNNCVFAP
cathepsin G	RMKLTLKG
cathepsin G	EAVKKLTV
granzyme B	VCTDKPTT
granzyme B	ITPDPNLS
chymase	RVGFYESD
chymase	VGFYESDV
elastase-1	LRVGFYES
elastase-1	GLRVGFYE
thrombin	GHARLVHV
complement factor D	QQKRKIVL
thrombin	AHPRIISA
thrombin	MVPRAVYL
thrombin	FRPKHTRI
plasmin	PQFRIKGG
HtrA2 peptidase	AMNVAYTD
kallikrein-related peptidase 3	SGTEASVV
u-plasminogen activator	CPGRVVGG
plasmin	LFEKKVYL
thrombin	VSPRASAS
thrombin	QSPRSFQK
thrombin	IQIRSVAK
thrombin	IEPRSFSQ
protein C (activated)	PQLRMKNN
protein C (activated)	VDQRGNQI
chymase	TAPYGLGN
elastase-2	VIPANMDK
elastase-2	PLVIVGLS
elastase-2	RRGIQRAA
elastase-2	ALGIRSFR
elastase-2	VTGIRLLS
elastase-2	WRLTSSEM
cathepsin G	SLLLQEFN
cathepsin G	YNTFSERR
cathepsin G	YVPYDDPY
coagulation factor VIIa	LSPRTFHP
coagulation factor VIIa	LIQRNLSP

matrix metallopeptidase-12	GAMFLEAI
matrix metallopeptidase-12	EAIPMSIP
membrane-type matrix metallopeptidase-6	GAMFLEAI
membrane-type matrix metallopeptidase-6	EAIPMSIP
matrix metallopeptidase-26	GAMFLEAI
matrix metallopeptidase-26	EAIPMSIP
matrix metallopeptidase-3	EAIPMSIP
membrane-type matrix metallopeptidase-6	LAQAVRSS
membrane-type matrix metallopeptidase-4	SPLAQAVR
membrane-type matrix metallopeptidase-4	PLAQAVRS
membrane-type matrix metallopeptidase-1	SPLAQAVR
membrane-type matrix metallopeptidase-1	LISPLAQA
membrane-type matrix metallopeptidase-4	PLAQAVRS
matrix metallopeptidase-20	PNLPLPAQ
matrix metallopeptidase-20	LPLPAQQP
matrix metallopeptidase-20	MGGWLHHQ
matrix metallopeptidase-2	DVANYNFF
matrix metallopeptidase-2	GPGGAWAA
matrix metallopeptidase-26	KPEGIDSR
matrix metallopeptidase-9	MDIAIHHP
matrix metallopeptidase-9	SPSRLFDQ
matrix metallopeptidase-9	FFGEHLLE
matrix metallopeptidase-9	SPEELKVK
matrix metallopeptidase-9	DVIEVHGK
matrix metallopeptidase-9	GEHLLESD
matrix metallopeptidase-9	LSPFYLRP
matrix metallopeptidase-9	PFFPFHSP
matrix metallopeptidase-9	LLESDLFP
matrix metallopeptidase-9	APSWFDTG
matrix metallopeptidase-9	VIEVHGKH
matrix metallopeptidase-9	KPAVTAAP
matrix metallopeptidase-20	MGGWLHHQ
membrane-type matrix metallopeptidase-1	GYFGDPLA
matrix metallopeptidase-7	GAMFLEAI
matrix metallopeptidase-8	GAMFLEAI
matrix metallopeptidase-8	EAIPMSIP
matrix metallopeptidase-9	GAMFLEAI
matrix metallopeptidase-9	EAIPMSIP

coagulation factor VIIa	MATRKMHD
coagulation factor VIIa	LGIRSFRN
thrombin	WYLRSNNG
protein C (activated)	KKTRNLKK
protein C (activated)	LDRRGIQR
protein C (activated)	RLKKSQFL
elastase-2	VKITLLSA
PIM1 peptidase	RMEAMGEW
PIM1 peptidase	SIDLKGWL
PIM1 peptidase	NQVLSQTQ
nucleoporin 145	VSHFSKYG
EGF-like module containing mucin-like hormone rece	CTHLSSFA
PIDD auto-processing protein unit 1	VPHFSWFL
PIDD auto-processing protein unit 1	VTHFSWYW
furin	RGRRSLGS
furin	VFRRDAHK
furin	HLKRDTED
furin	IRKRANSF
proprotein convertase 1	KTRREAED
proprotein convertase 1	LQKRGIVE
furin	RRKRYAIQ
furin	RHRRALDT
furin	RPKRYNSG
furin	RKRRSVNP
proprotein convertase 2	VAKKSVPH
furin	RQKRSINL
furin	RSKRSLSC
site-1 peptidase	RGLTSLAD
furin	RAKRSPKH
proprotein convertase 5	RPRRTKKT
furin	RKRRSTNE
furin	RQKRFVLS
furin	RRKRYAEH
site-1 peptidase	RSVLSFES
furin	RKPRCGNP
furin	IIRRSLPA
site-1 peptidase	RHLLGFSA
proprotein convertase 1	KDKRYGGF
proprotein convertase 1	EGKRSYSM

matrix metallopeptidase-11	AAGAMFLE
matrix metallopeptidase-7	EAIPMSIP
matrix metallopeptidase-12	RPFEVKDT
matrix metallopeptidase-12	GAMFLEAI
matrix metallopeptidase-12	EAIPMSIP
membrane-type matrix metallopeptidase-6	GAMFLEAI
membrane-type matrix metallopeptidase-6	EAIPMSIP
matrix metallopeptidase-26	GAMFLEAI
matrix metallopeptidase-26	EAIPMSIP
matrix metallopeptidase-3	EAIPMSIP
matrix metallopeptidase-20	MGGWLHHQ
matrix metallopeptidase-2	GPKGQKGE
matrix metallopeptidase-2	GPGGAWAA
matrix metallopeptidase-7	GAMFLEAI
matrix metallopeptidase-8	GAMFLEAI
matrix metallopeptidase-8	EAIPMSIP
matrix metallopeptidase-9	GAMFLEAI
matrix metallopeptidase-9	EAIPMSIP
matrix metallopeptidase-11	AAGAMFLE
matrix metallopeptidase-7	EAIPMSIP
matrix metallopeptidase-12	RPFEVKDT
matrix metallopeptidase-12	GAMFLEAI
matrix metallopeptidase-12	EAIPMSIP
membrane-type matrix metallopeptidase-6	GAMFLEAI
membrane-type matrix metallopeptidase-6	EAIPMSIP
matrix metallopeptidase-26	GAMFLEAI
matrix metallopeptidase-26	EAIPMSIP
matrix metallopeptidase-3	EAIPMSIP
matrix metallopeptidase-26	KALHVTNI
matrix metallopeptidase-11	KALHVTNI
membrane-type matrix metallopeptidase-1	GEYRTNPE
membrane-type matrix metallopeptidase-1	SSERSSTS
matrix metallopeptidase-3	TVASSSTA
matrix metallopeptidase-3	STAVIVSA
matrix metallopeptidase-2	GIQGLKGD
matrix metallopeptidase-2	GPLGARGI
matrix metallopeptidase-9	GPLGARGI
matrix metallopeptidase-3	GPLGARGI
matrix metallopeptidase-9	PAAAVLRE

proprotein convertase 2	GKKRRPVK
proprotein convertase 2	ENPRKYVM
proprotein convertase 1	RERKAGCK
proprotein convertase 7	KIRRSSEE
proprotein convertase 7	RSKRCSCS
proprotein convertase 7	RSKRALEN
furin	RENRCQCA
furin	RAPRSPKM
proprotein convertase 2	RRIRPKLK
proprotein convertase 1	LYKRYGGF
proprotein convertase 2	NQKRYGGF
furin	RSRRAATS
furin	VKKRSVSE
proprotein convertase 1	QHKRQHPG
proprotein convertase 1	VPKRQHPG
proprotein convertase 1	PEKRQHPG
proprotein convertase 1	EEKRQHPG
proprotein convertase 1	LSKRQHPG
furin	RAKRFASL
proprotein convertase 1	RGRRDFPE
proprotein convertase 2	EDKRHSQG
proprotein convertase 2	NTKRNRNN
site-1 peptidase	QKLLSIDL
site-1 peptidase	RRLLRAIP
site-1 peptidase	RSLKYAES
site-1 peptidase	RKVFRSLK
proprotein convertase 2	GGKRDAEN
proprotein convertase 1	VSKRYGGF
proprotein convertase 1	GLKRSPQL
proprotein convertase 1	YQKRYGGF
proprotein convertase 1	FLKRFAEA
proprotein convertase 1	MEKRYGGF
proprotein convertase 1	FMKKDAEE
prolyl oligopeptidase	RIRPKLKW

matrix metallopeptidase-3	VVYGLRSK
matrix metallopeptidase-7	VAQDLNAP
matrix metallopeptidase-7	RADDILAS
matrix metallopeptidase-9	TARPWRAD
matrix metallopeptidase-3	PPAHSHRD
matrix metallopeptidase-3	HHSSYVHL
matrix metallopeptidase-13	APHHSSYV
matrix metallopeptidase-13	HHSSYVHL
matrix metallopeptidase-20	HHSSYVHL
membrane-type matrix metallopeptidase-1	HHSSYVHL
membrane-type matrix metallopeptidase-1	GYFGDPLA
matrix metallopeptidase-7	PPLKLMHS
matrix metallopeptidase-9	PPLKLMHS
matrix metallopeptidase-9	NKGAIIGL
matrix metallopeptidase-9	AIIGLMVG
matrix metallopeptidase-9	IIGLMVGG
matrix metallopeptidase-9	LVFFAEDV
matrix metallopeptidase-9	FAEDVGSN
membrane-type matrix metallopeptidase-3	RVEAMLND
membrane-type matrix metallopeptidase-3	VLANMISE
membrane-type matrix metallopeptidase-3	EVHHQKLV
membrane-type matrix metallopeptidase-4	EVHHQKLV
membrane-type matrix metallopeptidase-4	EVHHQKLV
membrane-type matrix metallopeptidase-4	HHQKLVFF
matrix metallopeptidase-3	APRELKEQ
matrix metallopeptidase-3	SIAMSRMS
matrix metallopeptidase-3	FLQSLKGF
matrix metallopeptidase-2	CINYEAFV
matrix metallopeptidase-3	VVYGLRSK
matrix metallopeptidase-7	VAQDLNAP
matrix metallopeptidase-12	MLGEFVSE
matrix metallopeptidase-12	HTEKLVTS
matrix metallopeptidase-13	GDKELRTG
membrane-type matrix metallopeptidase-1	HTEKLVTS
membrane-type matrix metallopeptidase-1	DSHSLTTN
matrix metallopeptidase-8	TAWTADSG
matrix metallopeptidase-12	TAWTADSG
matrix metallopeptidase-13	TAWTADSG
matrix metallopeptidase-26	GDKELRTG

Cysteine Protease

cathepsin K	AGARGSDG
cathepsin K	GLPGFKGI
cathepsin S	QWLGAPVP
cathepsin H	QWLGAPVP
cathepsin S	CPVTYGQC
cathepsin S	EEVMSLMS
cathepsin S	SLMSSLRV
cathepsin K	YIPEWEGR
cathepsin K	LYPEEILD
cathepsin K	VPLSHSRS
dipeptidyl-peptidase I	NSQEKYSN
dipeptidyl-peptidase I	LTAEIQQK
dipeptidyl-peptidase I	LKNSQEKY
cathepsin K	EGPQGVRG
cathepsin K	MGPSGPRG
cathepsin K	QPPQEKAH
cathepsin K	DFSFLPQP
cathepsin K	GLPGMKGH
cathepsin H	ALQARPGP
cathepsin H	SEQAIPQA
cathepsin H	QCIHSPDL
cathepsin H	LLTLVPRG
cathepsin H	RCSMDDSA
cathepsin H	ALQARPGP
cathepsin H	SEQAIPQA
cathepsin H	QCIHSPDL
cathepsin H	LLTLVPRG
cathepsin H	RCSMDDSA
legumain	LYENKPRR
legumain	MNTNDLEE
legumain	FFKNIVTP
legumain	MNTNDLEE
legumain	FFKNIVTP
legumain	FFKNIVTP

matrix metallopeptidase-26	HTEKLVTS
matrix metallopeptidase-9	NKGAIIGL
matrix metallopeptidase-9	AIIGLMVG
matrix metallopeptidase-9	IIGLMVGG
matrix metallopeptidase-9	LVFFAEDV
matrix metallopeptidase-9	FAEDVGSN
membrane-type matrix metallopeptidase-3	RVEAMLND
membrane-type matrix metallopeptidase-3	VLANMISE
membrane-type matrix metallopeptidase-3	EVHHQKLV
membrane-type matrix metallopeptidase-4	EVHHQKLV
membrane-type matrix metallopeptidase-4	EVHHQKLV
membrane-type matrix metallopeptidase-4	HHQKLVFF
matrix metallopeptidase-7	ALAAYRLE
matrix metallopeptidase-3	ALAAYRLE
matrix metallopeptidase-7	RANCLVQT
matrix metallopeptidase-13	RANCLVQT
membrane-type matrix metallopeptidase-1	ALAAYRLE
matrix metallopeptidase-3	VAPPIHQG
matrix metallopeptidase-3	EPTTIIRQ
matrix metallopeptidase-3	AQPDALNV
matrix metallopeptidase-3	QPDALNVP
matrix metallopeptidase-13	GIQSLYGP
matrix metallopeptidase-3	DTLEVMRK
matrix metallopeptidase-7	PPLKLMHS
matrix metallopeptidase-9	PPLKLMHS
matrix metallopeptidase-9	NKGAIIGL
matrix metallopeptidase-9	AIIGLMVG
matrix metallopeptidase-9	IIGLMVGG
matrix metallopeptidase-9	LVFFAEDV
matrix metallopeptidase-9	FAEDVGSN
membrane-type matrix metallopeptidase-3	RVEAMLND
membrane-type matrix metallopeptidase-3	VLANMISE
membrane-type matrix metallopeptidase-3	EVHHQKLV
membrane-type matrix metallopeptidase-4	EVHHQKLV
membrane-type matrix metallopeptidase-4	EVHHQKLV
membrane-type matrix metallopeptidase-4	HHQKLVFF

legumain	FFKNIVTP
legumain	FFKNIVTP
caspase-6	VEMDAAPG
caspase-6	VEMDAAPG
caspase-3	DLLDDGEI
caspase-3	DVLDVLNE
caspase-3	SYNDFGNY
caspase-3	GSYDSYNN
caspase-3	SYNDFGNY
caspase-1	ALDDLIDT
caspase-1	LSSDFTCG
caspase-1	ALADSLGK
caspase-3	LSSDFTCG
caspase-3	DQTDGLGL
caspase-3	DGPDGPEE
caspase-1	AVQDNPAM
caspase-1	WFKDSVGV
caspase-1	FEDDAIKK
caspase-3	GQVDVPLY
caspase-3	DENDGPGE
caspase-3	DENDGPGE
caspase-8	DENDGPGE
caspase-3	DGLDGPTY
caspase-3	DITDFQAK
caspase-3	EQEDSSSA
caspase-3	DITDSSAA
caspase-3	DITDSSAA
caspase-3	IETDAMIK
caspase-3	DEVDGMAG
caspase-3	DLVDSQIR
caspase-8	ILRDKDNT
caspase-3	YVPDSPAL
caspase-1	YVPDSPAL
caspase-3	DQTDGLGL
caspase-3	YVPDSPAL
caspase-1	YVPDSPAL
caspase-3	DYLDLAAS
caspase-3	VSVDAFKI
caspase-3	DDEDDDVD
caspase-3	DEDDDVDT
caspase-3	EERDGSLN
caspase-1	ELPDGQVI
caspase-3	ELPDGQVI
caspase-3	YVPDSPAL

matrix metallopeptidase-12	MLGEFVSE
matrix metallopeptidase-12	HTEKLVTS
matrix metallopeptidase-13	GDKELRTG
membrane-type matrix metallopeptidase-1	HTEKLVTS
membrane-type matrix metallopeptidase-1	DSHSLTTN
matrix metallopeptidase-8	TAWTADSG
matrix metallopeptidase-12	TAWTADSG
matrix metallopeptidase-13	TAWTADSG
matrix metallopeptidase-26	GDKELRTG
matrix metallopeptidase-26	HTEKLVTS
matrix metallopeptidase-3	VVYGLRSK
matrix metallopeptidase-7	VAQDLNAP
matrix metallopeptidase-7	GAMFLEAI
matrix metallopeptidase-8	GAMFLEAI
matrix metallopeptidase-8	EAIPMSIP
matrix metallopeptidase-9	GAMFLEAI
matrix metallopeptidase-9	EAIPMSIP
matrix metallopeptidase-11	AAGAMFLE
matrix metallopeptidase-7	EAIPMSIP
matrix metallopeptidase-12	RPFEVKDT
matrix metallopeptidase-12	GAMFLEAI
matrix metallopeptidase-12	EAIPMSIP
membrane-type matrix metallopeptidase-6	GAMFLEAI
membrane-type matrix metallopeptidase-6	EAIPMSIP
matrix metallopeptidase-26	GAMFLEAI
matrix metallopeptidase-26	EAIPMSIP
matrix metallopeptidase-3	EAIPMSIP
matrix metallopeptidase-2	GPPGKLGP
matrix metallopeptidase-2	GPKGQKGD
matrix metallopeptidase-9	GPKGQKGD
membrane-type matrix metallopeptidase-1	GLRGLQGP
matrix metallopeptidase-3	PPEELKFQ
matrix metallopeptidase-7	PPEELKFQ
matrix metallopeptidase-2	CINYEAFV
matrix metallopeptidase-9	LVEALYLV
matrix metallopeptidase-9	GERGFFYT
matrix metallopeptidase-9	VNQHLCGS
matrix metallopeptidase-9	EALYLVCG
matrix metallopeptidase-3	LVEALYLV
matrix metallopeptidase-7	LVEALYLV
matrix metallopeptidase-7	RADDILAS
matrix metallopeptidase-9	TARPWRAD
matrix metallopeptidase-3	PPAHSHRD
matrix metallopeptidase-3	HHSSYVHL

caspase-1	YVPDSPAL
caspase-3	FIQDRAGR
caspase-1	DRHDGTSN
caspase-3	DRHDGTSN
caspase-6	DRHDGTSN
caspase-3	YVPDSPAL
caspase-1	YVPDSPAL
caspase-1	ALDDLIDT
caspase-1	LSSDFTCG
caspase-1	ALADSLGK
caspase-3	LSSDFTCG
caspase-3	YVPDSPAL
caspase-1	YVPDSPAL
caspase-3	DEVDNKVK
caspase-3	DLLDDGEI
caspase-3	DVLDVLNE
caspase-3	DNIDNLSP
caspase-3	MELDGPKG
caspase-3	SYNDFGNY
caspase-3	SVTDSVMG
caspase-3	DEDDSAAP
caspase-3	DYLDLAAS
caspase-3	VSVDAFKI
caspase-3	DQTDGLGL
caspase-1	ALDDLIDT
caspase-1	LSSDFTCG
caspase-1	ALADSLGK
caspase-3	LSSDFTCG
caspase-3	DLLDDGEI
caspase-3	DVLDVLNE
caspase-3	DETDSKTA
caspase-3	DSLDSVEA
caspase-1	DETDSKTA
caspase-4	DETDSKTA
caspase-8	ILRDKDNT
caspase-3	QSVDGKAP
caspase-3	DHVDGQIL
caspase-7	DHVDGQIL
caspase-1	DRHDGTSN
caspase-3	DRHDGTSN
caspase-6	DRHDGTSN
caspase-3	DDADYKPK

matrix metallopeptidase-13	APHHSSYV
matrix metallopeptidase-13	HHSSYVHL
matrix metallopeptidase-20	HHSSYVHL
membrane-type matrix metallopeptidase-1	HHSSYVHL
matrix metallopeptidase-7	GAMFLEAI
matrix metallopeptidase-8	GAMFLEAI
matrix metallopeptidase-8	EAIPMSIP
matrix metallopeptidase-9	GAMFLEAI
matrix metallopeptidase-9	EAIPMSIP
matrix metallopeptidase-11	AAGAMFLE
matrix metallopeptidase-7	EAIPMSIP
matrix metallopeptidase-12	RPFEVKDT
matrix metallopeptidase-12	GAMFLEAI
matrix metallopeptidase-12	EAIPMSIP
membrane-type matrix metallopeptidase-6	GAMFLEAI
membrane-type matrix metallopeptidase-6	EAIPMSIP
matrix metallopeptidase-26	GAMFLEAI
matrix metallopeptidase-26	EAIPMSIP
matrix metallopeptidase-3	EAIPMSIP
matrix metallopeptidase-9	MSYNLLGF
matrix metallopeptidase-9	NVYHQINH
matrix metallopeptidase-3	APGNASES
matrix metallopeptidase-7	AVSRLRAY
matrix metallopeptidase-1	HPLHSKII
matrix metallopeptidase-2	SRLRAYLL
matrix metallopeptidase-2	HPLHSKII
matrix metallopeptidase-2	VSRLRAYL
matrix metallopeptidase-2	FHPLHSKI
matrix metallopeptidase-2	CINYEAFV
matrix metallopeptidase-2	GPQGIAGQ
membrane-type matrix metallopeptidase-1	GPQGIAGQ
matrix metallopeptidase-9	GPAGEEGK
matrix metallopeptidase-9	HPKNIQSL
matrix metallopeptidase-9	VIATLKDG
matrix metallopeptidase-9	LSRFSWGA
matrix metallopeptidase-13	GIQSLYGP
matrix metallopeptidase-3	DVGHFRTF
matrix metallopeptidase-3	DTLEVMRK
membrane-type matrix metallopeptidase-1	LSLPVENR
membrane-type matrix metallopeptidase-4	LPVENRLY
membrane-type matrix metallopeptidase-4	VENRLYTY
matrix metallopeptidase-3	LPVENRLY

caspase-3	EEEDGKLK
caspase-6	PEDDGYFV
caspase-6	EEEDGKLK
caspase-3	DNIDNLSP
caspase-3	DFPDWWQV
caspase-1	ALDDLIDT
caspase-1	LSSDFTCG
caspase-1	ALADSLGK
caspase-3	LSSDFTCG
caspase-3	DLRDDKDT
caspase-3	MELDGPKG
caspase-3	SVTDSVMG
caspase-3	DNIDNLSP
caspase-3	DDVDTKKQ
caspase-3	DDEDDDVD
caspase-3	DEDDDVDT
caspase-1	ALDDLIDT
caspase-1	LSSDFTCG
caspase-1	ALADSLGK
caspase-3	LSSDFTCG
caspase-3	DEVDNKVK
caspase-1	DRHDGTSN
caspase-3	DRHDGTSN
caspase-6	DRHDGTSN
caspase-3	PHLDGPPS
caspase-3	DEDDDDEE
caspase-3	DVPDGPLL
caspase-3	DSPDGQYE
caspase-8	ILRDKDNT
caspase-3	DSTDSPAS
caspase-3	DETDHSSK
caspase-3	DVTDIAPS
caspase-3	DAGDVGAA
caspase-3	YVPDSPAL
caspase-1	YVPDSPAL
caspase-1	ALDDLIDT
caspase-1	LSSDFTCG
caspase-1	ALADSLGK
caspase-3	LSSDFTCG
caspase-3	DVTDIAPS
caspase-8	AAVDGPMD
caspase-3	DVTDIAPS

membrane-type matrix metallopeptidase-1	LPVENRLY
membrane-type matrix metallopeptidase-4	VENRLYTY
membrane-type matrix metallopeptidase-4	LPVENRLY
matrix metallopeptidase-7	ALEDLRQG
matrix metallopeptidase-12	ALEDLRQG
matrix metallopeptidase-12	RLAEYHAK
matrix metallopeptidase-12	KVEPLRAE
matrix metallopeptidase-3	RLAEYHAK
matrix metallopeptidase-2	CINYEAFV
matrix metallopeptidase-3	TVAEKTKE
matrix metallopeptidase-3	GVATVAEK
membrane-type matrix metallopeptidase-1	GEYRTNPE
membrane-type matrix metallopeptidase-1	SSERSSTS
matrix metallopeptidase-7	AAQNLYEK
membrane-type matrix metallopeptidase-1	AAQNLYEK
matrix metallopeptidase-2	CINYEAFV
matrix metallopeptidase-9	QSVNVKSP
matrix metallopeptidase-9	VATELRCQ
matrix metallopeptidase-2	IITGLTIL
matrix metallopeptidase-2	ITGLTILN
matrix metallopeptidase-9	ITGLTILN
matrix metallopeptidase-12	ITGLTILN
matrix metallopeptidase-13	IITGLTIL
matrix metallopeptidase-9	GHRGFTGL
matrix metallopeptidase-9	GPPGLQGM
matrix metallopeptidase-9	LSRFSWGA
membrane-type matrix metallopeptidase-1	TKRDLALS
matrix metallopeptidase-2	GPKGQKGD
matrix metallopeptidase-9	GPKGQKGD
matrix metallopeptidase-3	IAGRSLNP
matrix metallopeptidase-9	GPGGVVGP
matrix metallopeptidase-9	GPPGVVGP
matrix metallopeptidase-9	GPGGVVGP
matrix metallopeptidase-7	MLEDEASG
matrix metallopeptidase-9	KPVSLSYR
matrix metallopeptidase-2	GPKGQKGE
matrix metallopeptidase-7	GAMFLEAI
matrix metallopeptidase-8	GAMFLEAI
matrix metallopeptidase-8	EAIPMSIP
matrix metallopeptidase-9	GAMFLEAI
matrix metallopeptidase-9	EAIPMSIP
matrix metallopeptidase-11	AAGAMFLE

caspase-3	DDVDIPTP
caspase-3	MDIDGVSC
caspase-7	MDIDGVSC
caspase-6	VDVDSGIE
caspase-3	DLADYGGY
caspase-3	DDVDIPTP
caspase-9	MIEDAIRS
caspase-3	DELDAAVA
caspase-3	VEVDPMLT
caspase-3	DVTDIAPS
caspase-3	FIQDRAGR
caspase-1	ALDDLIDT
caspase-1	LSSDFTCG
caspase-1	ALADSLGK
caspase-3	LSSDFTCG
caspase-3	SYNDFGNY
caspase-3	DQIDDTVE
caspase-3	DLYDCVEN
caspase-3	DDVDTKKQ
caspase-3	DDEDDDVD
caspase-3	DEDDDVDT
caspase-3	DLLDDGEI
caspase-3	DVLDVLNE
caspase-3	DSLDGRSL
caspase-7	DSLDGRSL
caspase-2	DSLDGRSL
caspase-8	DSLDGRSL
caspase-1	ALDDLIDT
caspase-1	LSSDFTCG
caspase-1	ALADSLGK
caspase-3	LSSDFTCG
caspase-3	DDVDTKKQ
caspase-3	DDEDDDVD
caspase-3	DEDDDVDT
caspase-3	MDIDGVSC
caspase-7	MDIDGVSC
caspase-6	VDVDSGIE
caspase-1	ELPDGQVI
caspase-3	ELPDGQVI
caspase-3	SVTDSVMG
caspase-8	ILRDKDNT

matrix metallopeptidase-7	EAIPMSIP
matrix metallopeptidase-12	RPFEVKDT
matrix metallopeptidase-12	GAMFLEAI
matrix metallopeptidase-12	EAIPMSIP
membrane-type matrix metallopeptidase-6	GAMFLEAI
membrane-type matrix metallopeptidase-6	EAIPMSIP
matrix metallopeptidase-26	GAMFLEAI
matrix metallopeptidase-26	EAIPMSIP
matrix metallopeptidase-3	EAIPMSIP
membrane-type matrix metallopeptidase-1	MDETMKEL
membrane-type matrix metallopeptidase-1	EQVAEVRA
membrane-type matrix metallopeptidase-1	DPFRLQCT
matrix metallopeptidase-3	DVAEYSLF
matrix metallopeptidase-3	KPQQFFGL
membrane-type matrix metallopeptidase-1	GEYRTNPE
membrane-type matrix metallopeptidase-1	SSERSSTS
matrix metallopeptidase-2	GHARLVHV
matrix metallopeptidase-2	GPEGLRVG
matrix metallopeptidase-3	GPEGLRVG
matrix metallopeptidase-3	RVGFYESD
matrix metallopeptidase-7	MLEDEASG
matrix metallopeptidase-3	KPQQFFGL
membrane-type matrix metallopeptidase-1	VLPRSAKE
matrix metallopeptidase-9	LPRSAKEL
matrix metallopeptidase-9	KPVSLSYR
matrix metallopeptidase-3	PPAPVQAK
matrix metallopeptidase-3	CAAPSFDC
matrix metallopeptidase-9	VAPPPVVL
matrix metallopeptidase-7	APPPVVLL
matrix metallopeptidase-3	APGNASES
matrix metallopeptidase-7	AVSRLRAY
matrix metallopeptidase-1	HPLHSKII
matrix metallopeptidase-2	SRLRAYLL
matrix metallopeptidase-2	HPLHSKII
matrix metallopeptidase-2	VSRLRAYL
matrix metallopeptidase-2	FHPLHSKI
matrix metallopeptidase-9	GPVSAVLT
matrix metallopeptidase-3	DAASLKGL
matrix metallopeptidase-7	MLEDEASG
matrix metallopeptidase-7	HLRELHLD
matrix metallopeptidase-9	KPVSLSYR

caspase-3	DLFDLTSG
caspase-3	SSYDGSDR
caspase-7	SSYDGSDR
caspase-3	SSYDGSDR
caspase-7	SSYDGSDR
caspase-3	SSYDGSDR
caspase-7	SSYDGSDR
caspase-3	SSYDGSDR
caspase-7	SSYDGSDR
caspase-3	SVTDSVMG
caspase-3	DDEDDDVD
caspase-3	DEDDDVDT
caspase-8	ILRDKDNT
caspase-3	DSSDSELE
caspase-3	TQFDAAHP
caspase-3	SYLDSGIH
caspase-3	YPVDGLPD
caspase-3	DLMDGLPP
caspase-3	ADIDGQYA
caspase-3	DYPDSSVS
caspase-3	DLRDDKDT
caspase-3	DLKDHMRE
caspase-3	CYADVYRD
caspase-3	VYRDGTGV
caspase-3	RKLDNTKF
caspase-3	DLRDDPST
caspase-7	DLRDDPST
caspase-3	VLGDGVQL
caspase-3	TQFDAAHP
caspase-3	SYLDSGIH
caspase-3	YPVDGLPD
caspase-3	DLMDGLPP
caspase-3	ADIDGQYA
caspase-3	GSSDPLIQ
caspase-3	DMVDSPQL
caspase-1	DMVDSPQL
caspase-7	GSSDPLIQ
caspase-7	DMVDSPQL
caspase-6	DMVDSPQL
caspase-8	GSSDPLIQ
caspase-8	DMVDSPQL
caspase-3	DYADGAIS
caspase-3	DLKDHMRE
caspase-3	CYADVYRD
caspase-3	VYRDGTGV
caspase-3	RKLDNTKF

matrix metallopeptidase-3	TVAEKTKE
matrix metallopeptidase-3	GVATVAEK
matrix metallopeptidase-26	QMHALLHQ
matrix metallopeptidase-26	SPLLTQET
matrix metallopeptidase-26	QLLQQFHR
matrix metallopeptidase-1	LLSALVET
matrix metallopeptidase-3	LLSALVET
matrix metallopeptidase-13	LLSALVET
matrix metallopeptidase-7	EPGDIKDI
matrix metallopeptidase-7	EPGDIKDI
matrix metallopeptidase-9	IVENLLAN
matrix metallopeptidase-3	AVAQKTVE
matrix metallopeptidase-3	AVAQKTVE
procollagen C-peptidase	QLLDDGNG
procollagen C-peptidase	TPQSQDPN
adamalysin-19	RPLESNAV
meprin beta subunit	MYPRGNHW
procollagen C-peptidase	FMLEDEAS
procollagen I N-peptidase	NFAAQMAG
procollagen C-peptidase	YMRADQAA
procollagen C-peptidase	FYRADQPR
meprin beta subunit	MYPRGNHW
procollagen C-peptidase	VAVGDSTG
procollagen C-peptidase	FMLEDEAS
procollagen I N-peptidase	NFAAQMAG
procollagen C-peptidase	YMRADQAA
meprin beta subunit	LVEALYLV
adamalysin-19	LVEALYLV
procollagen C-peptidase	FMLEDEAS
ADAM17 peptidase	AGQRLATA
ADAM17 peptidase	NSARSEGP
ADAM17 peptidase	VAAAVVSH
ADAM17 peptidase	DLLAVVAA
ADAM17 peptidase	RVEQVVKP
ADAM17 peptidase	EYDNEPQI
ADAM8 peptidase	KEASSTFS
procollagen C-peptidase	PMQADGPR
procollagen C-peptidase	RSYSDRGE
mammalian tolloid-like 1 protein	PMQADGPR
mammalian tolloid-like 1 protein	RSYSDRGE
ADAM17 peptidase	VAAAVVSH
ADAM17 peptidase	DLLAVVAA
ADAMTS4 peptidase	EAAEARRG
procollagen C-peptidase	GMVGDDPY
mammalian tolloid-like 1 protein	GMVGDDPY
ADAMTS13 peptidase	NLVYMVTG

caspase-3	FIQDRAGR
caspase-3	EEIDHAER
caspase-1	EEIDHAER
caspase-7	EEIDHAER
caspase-3	DSLDGRSL
caspase-7	DSLDGRSL
caspase-2	DSLDGRSL
caspase-8	DSLDGRSL
caspase-3	DEDDDDEE
caspase-3	DELDSKLN
caspase-3	AEPDYGAL
caspase-7	AEPDYGAL
caspase-3	DLPDMKET
caspase-3	DSYDSFGE
caspase-8	ILRDKDNT
caspase-6	VEMDAAPG
caspase-6	VEMDAAPG
caspase-3	SDSDGLAP
caspase-3	TFSDLWKL
caspase-3	DLRDDPST
caspase-7	DLRDDPST
caspase-3	DSSDSELE
caspase-3	LQTDGNRS
caspase-3	IEADSESQ
caspase-8	LQTDGNRS
caspase-8	IEADSESQ
caspase-3	LISDTYLA
caspase-3	DLPDCEAD
caspase-3	DEEDLQRA
caspase-6	IVLDGTDN
caspase-2	DLNDGTQA
caspase-3	DSVDLASC
caspase-3	DEEDILSH
caspase-3	DLNDGTQA
caspase-3	IVLDGTDN
caspase-3	DLLDDGEI
caspase-3	DVLDVLNE
caspase-3	QSVDGKAP
caspase-8	ILRDKDNT
caspase-3	TQFDAAHP
caspase-3	SYLDSGIH
caspase-3	YPVDGLPD
caspase-3	DLMDGLPP
caspase-3	ADIDGQYA
caspase-3	DDVDTKKQ
caspase-3	DDEDDDVD
caspase-3	DEDDDVDT
caspase-3	LHTDSRKD

ADAM8 peptidase	KEASSTFS
meprin beta subunit	GPWLEEEE
meprin beta subunit	PWLEEEEE
meprin beta subunit	WLEEEEEA
meprin beta subunit	LEEEEEAY
meprin beta subunit	NPGEDAPA
meprin beta subunit	APAEDMAR
adamalysin-19	RPLESNAV
ADAM10 peptidase	SFEMQKGD
procollagen C-peptidase	DVQRDDSS
mammalian tolloid-like 1 protein	DVQRDDSS
procollagen C-peptidase	PYYGDEPM
ADAMTS4 peptidase	KEEEGLGS
ADAMTS4 peptidase	TAQEAGEG
ADAMTS4 peptidase	TEGEARGS
ADAMTS1 peptidase	KEEEGLGS
ADAMTS5 peptidase	TEGEARGS
ADAMTS4 peptidase	IPENFFGV
ADAMTS1 peptidase	SELEGRGT
ADAMTS1 peptidase	ISQELGQR
ADAMTS1 peptidase	TEGEARGS
ADAMTS5 peptidase	TEGEARGS
ADAMTS4 peptidase	KEEEGLGS
ADAMTS4 peptidase	TAQEAGEG
ADAMTS4 peptidase	TEGEARGS
ADAMTS1 peptidase	KEEEGLGS
ADAMTS5 peptidase	TEGEARGS
ADAMTS4 peptidase	IPENFFGV
ADAMTS1 peptidase	SELEGRGT
ADAMTS1 peptidase	ISQELGQR
ADAMTS1 peptidase	TEGEARGS
ADAMTS5 peptidase	TEGEARGS
ADAM17 peptidase	LAQAVRSS
ADAM17 peptidase	LAQAVRSS
ADAM10 peptidase	LAQAVRSS
ADAM8 peptidase	SPLAQAVR
ADAM8 peptidase	LAQAVRSS
ADAM9 peptidase	SPLAQAVR
ADAM9 peptidase	SPLAQAVR
ADAM9 peptidase	AVRSSSRT
ADAM10 peptidase	LAQAVRSS
ADAM17 peptidase	LAQAVRSS
ADAM8 peptidase	SPLAQAVR
ADAM8 peptidase	LAQAVRSS
ADAM33 peptidase	GSQHIRAE
procollagen C-peptidase	SYAADTAG
procollagen C-peptidase	CYSGDENP
procollagen C-peptidase	CYSGDENP

caspase-1	LHTDSRKD
caspase-1	ELPDGQVI
caspase-3	ELPDGQVI
caspase-1	DEPDSPPV
caspase-3	DEPDSPPV
caspase-7	DEPDSPPV
caspase-3	AEPDYGAL
caspase-7	AEPDYGAL
caspase-3	DLRDDPST
caspase-7	DLRDDPST
caspase-3	DVVDNQTE
caspase-3	TEVDAASV
caspase-3	TETDAFYK
caspase-3	VDFDDIHR
caspase-3	EEIDHAER
caspase-1	EEIDHAER
caspase-7	EEIDHAER
caspase-3	LQTDGNRS
caspase-3	IEADSESQ
caspase-8	LQTDGNRS
caspase-8	IEADSESQ
caspase-1	ELPDGQVI
caspase-3	ELPDGQVI
caspase-3	DDVDTKKQ
caspase-3	DDEDDDVD
caspase-3	DEDDDVDT
caspase-1	HLADSPAV
caspase-3	HLADSPAV
caspase-3	SSLDAREV
caspase-3	SDSDGLAP
caspase-3	TFSDLWKL
caspase-3	EEIDHAER
caspase-1	EEIDHAER
caspase-7	EEIDHAER
caspase-3	SEVDGNRM
caspase-3	LVFDNQLT
caspase-3	EEIDHAER
caspase-1	EEIDHAER
caspase-7	EEIDHAER
caspase-3	PHLDGPPS
caspase-3	DMEDFGLV
caspase-3	DQLDNIVD
caspase-3	LISDTYLA
caspase-3	DLPDCEAD
caspase-3	DEEDLQRA
caspase-3	DEDDDDEE
caspase-3	DESDFGPL
caspase-3	DMVDSPQL
caspase-1	DMVDSPQL

ADAM17 peptidase	HHQKLVFF
ADAM8 peptidase	EVHHQKLV
ADAM9 peptidase	EVHHQKLV
ADAM10 peptidase	HHQKLVFF
ADAM9 peptidase	EVHHQKLV
ADAM33 peptidase	EVHHQKLV
ADAM33 peptidase	GSQHIRAE
ADAM8 peptidase	PVAASSLR
ADAM9 peptidase	PPVAASSL
ADAM9 peptidase	VAASSLRN
adamalysin-19	PVAASSLR
ADAM17 peptidase	PVAASSLR
ADAM33 peptidase	PVAASSLR
ADAM17 peptidase	HHQKLVFF
ADAM8 peptidase	EVHHQKLV
ADAM9 peptidase	EVHHQKLV
ADAM10 peptidase	HHQKLVFF
ADAM9 peptidase	EVHHQKLV
ADAM33 peptidase	EVHHQKLV
ADAM17 peptidase	HHQKLVFF
ADAM8 peptidase	EVHHQKLV
ADAM9 peptidase	EVHHQKLV
ADAM10 peptidase	HHQKLVFF
ADAM9 peptidase	EVHHQKLV
ADAM33 peptidase	EVHHQKLV
meprin beta subunit	LVEALYLV
adamalysin-19	LVEALYLV
ADAM9 peptidase	GAVHLPQP
procollagen I N-peptidase	NFAPQLSY
procollagen C-peptidase	YYRADDAN
procollagen C-peptidase	YYRADDAN
procollagen C-peptidase	DFQGDALQ
ADAM10 peptidase	KKGKGLGK
ADAM17 peptidase	LSLPVENR
ADAM9 peptidase	PPAASSLR
ADAM9 peptidase	PPAASSLR
ADAMTS4 peptidase	SFRKFRYG
ADAMTS4 peptidase	SALTFREE
procollagen C-peptidase	GMQSDDPE
procollagen C-peptidase	EFTEDQAA
ADAM17 peptidase	RVEQVVKP
ADAM17 peptidase	EYDNEPQI
meprin beta subunit	HSQGTFTS
meprin beta subunit	TFTSDYSK
meprin beta subunit	SKYLDSRR
meprin beta subunit	DSRRAQDF
meprin beta subunit	RRAQDFVQ
meprin beta subunit	MYPRGNHW
ADAM17 peptidase	AGQRLATA

caspase-7	DMVDSPQL
caspase-6	DMVDSPQL
caspase-8	DMVDSPQL
caspase-1	LVVDNGSG
caspase-1	ELPDGQVI
caspase-3	ELPDGQVI
caspase-3	PHLDGPPS
caspase-3	DDEDDDVD
caspase-3	DEDDDVDT
caspase-3	DEIDHAEM
caspase-1	DEIDHAEM
caspase-3	TLTDSSML
caspase-3	STTDLTVP
caspase-3	SSYDGSDR
caspase-7	STTDLTVP
caspase-7	SSYDGSDR
caspase-6	TLTDSSML
caspase-3	SSYDGSDR
caspase-7	SSYDGSDR
caspase-3	KLTDCVVM
caspase-3	VMRDPASK
caspase-3	AEVDAAMA
caspase-3	TLTDSSML
caspase-3	STTDLTVP
caspase-3	SSYDGSDR
caspase-7	STTDLTVP
caspase-7	SSYDGSDR
caspase-6	TLTDSSML
caspase-3	SSYDGSDR
caspase-7	SSYDGSDR
caspase-3	DSVDFSLA
caspase-7	DSVDFSLA
caspase-6	IDVDVSKP
caspase-9	IDVDVSKP
caspase-3	TLTDSSML
caspase-3	STTDLTVP
caspase-3	SSYDGSDR
caspase-7	STTDLTVP
caspase-7	SSYDGSDR

ADAM17 peptidase	NSARSEGP
procollagen C-peptidase	GMQSDDPE
procollagen C-peptidase	FMLEDEAS
procollagen C-peptidase	FYESDVMG
ADAMTS4 peptidase	SDVMGRGH
ADAMTS5 peptidase	SDVMGRGH
procollagen C-peptidase	FMMNDEEA
mammalian tolloid-like 1 protein	FMMNDEEA
procollagen C-peptidase	FMLEDEAS
procollagen C-peptidase	FMLEDEAS
ADAM8 peptidase	FAQAQQQL
endothelin-converting enzyme 1	DIIWINTP
Kell blood-group protein	DIIWINTP
endothelin-converting enzyme 2	DIIWINTP
neprilysin	KPQQFFGL
neprilysin	PQQFFGLM
endothelin-converting enzyme 1	KPQQFFGL
endothelin-converting enzyme 1	PQQFFGLM
endothelin-converting enzyme 1	DIIWINTP
Kell blood-group protein	DIIWINTP
endothelin-converting enzyme 2	DIIWINTP
neprilysin	KPQQFFGL
neprilysin	PQQFFGLM
endothelin-converting enzyme 1	KPQQFFGL
endothelin-converting enzyme 1	PQQFFGLM
neprilysin	GSHLVEAL
neprilysin	LVEALYLV
neprilysin	GERGFFYT
neprilysin	ERGFFYTP
neprilysin	RGFFYTPK
PHEX peptidase	DHLSDTST
PHEX peptidase	DHLSDTST
neprilysin	FGGRMDRI
neprilysin	RMDRIGAQ
neprilysin	SLRRSSCF
neprilysin	RSSCFGGR
neprilysin	DRIGAQSG
neprilysin	AQSGLGCN
endothelin-converting enzyme 1	DIIWVNTP

caspase-6	TLTDSSML
caspase-3	SSYDGSDR
caspase-7	SSYDGSDR
caspase-3	DFPDWWQV
caspase-3	SDSDGLAP
caspase-3	TFSDLWKL
caspase-3	ESVDYRAT
caspase-3	DEVDRDAP
caspase-3	TLTDSSML
caspase-3	STTDLTVP
caspase-3	SSYDGSDR
caspase-7	STTDLTVP
caspase-7	SSYDGSDR
caspase-6	TLTDSSML
caspase-3	SSYDGSDR
caspase-7	SSYDGSDR
caspase-3	VEVDAPKS
caspase-7	EVDAPKSQ
caspase-6	EVDAPKSQ
caspase-8	TEKDSMIE
caspase-3	TEKDSMIE
caspase-3	DEQDGASA
caspase-3	VDFDDIHR
caspase-8	LQLDCVAV
caspase-3	EEADSMKS
caspase-3	ESVDKSAG
caspase-3	DLRDDKDT
caspase-3	PHLDGPPS
caspase-3	DEVDRDAP
caspase-3	YPPDYYGY
caspase-3	DYYDDYYG

neprilysin-2	DIIWVNTP
Kell blood-group protein	DIIWVNTP
endothelin-converting enzyme 1	DIIWVNTP
Kell blood-group protein	DIIWVNTP
endothelin-converting enzyme 1	CHLDIIWV
endothelin-converting enzyme 1	YGLGSPRS
endothelin-converting enzyme 1	TPEHVVPY
endothelin-converting enzyme 1	HLDIIWVN
endothelin-converting enzyme 1	CVYFCHLD
endothelin-converting enzyme 1	SCSSLMDK
endothelin-converting enzyme 1	ECVYFCHL
endothelin-converting enzyme 2	DIIWVNTP
neprilysin	QKLVFFAE
endothelin-converting enzyme 1	HQKLVFFA
endothelin-converting enzyme 1	HHQKLVFF
endothelin-converting enzyme 1	KLVFFAED
neprilysin	QKLVFFAE
endothelin-converting enzyme 1	HQKLVFFA
endothelin-converting enzyme 1	HHQKLVFF
endothelin-converting enzyme 1	KLVFFAED
neprilysin	QKLVFFAE
endothelin-converting enzyme 1	HQKLVFFA
endothelin-converting enzyme 1	HHQKLVFF
endothelin-converting enzyme 1	KLVFFAED
neprilysin	GSHLVEAL
neprilysin	LVEALYLV
neprilysin	GERGFFYT
neprilysin	ERGFFYTP
neprilysin	RGFFYTPK
neprilysin	DYMGWMDF
neprilysin	VASEFRKK

caspase-3	DYHDYRGG
caspase-3	RAIDALRE
caspase-3	KESDLSHV
caspase-3	DMTDSDCR
caspase-1	LESDYFGK
caspase-3	EEADSMKS
caspase-3	ESVDKSAG
caspase-3	LISDTYLA
caspase-3	DLPDCEAD
caspase-3	DEEDLQRA
caspase-3	DSYDSFGE
caspase-3	DMVDSPQL
caspase-1	DMVDSPQL
caspase-7	DMVDSPQL
caspase-6	DMVDSPQL
caspase-8	DMVDSPQL
caspase-3	PHLDGPPS
caspase-3	SDSDGLAP
caspase-3	TFSDLWKL
caspase-3	NPQDSVGS
caspase-3	SELDRLLL
caspase-3	FPADEANS
caspase-3	SLLDELES
caspase-3	SQLDSMLG
caspase-3	DDLDALLA
caspase-3	KLTDCVVM
caspase-3	VMRDPASK
caspase-3	AEVDAAMA
caspase-3	EHIDHHIQ
caspase-3	EHIDHHIQ
caspase-3	EHIDHHIQ
caspase-3	DDNDSERN
caspase-7	DSPDSVDP

neprilysin	FRKKWNKW
neprilysin	KWNKWALS
neprilysin	WNKWALSR
neprilysin	NKWALSRG
neprilysin	KPQQFFGL
neprilysin	PQQFFGLM
endothelin-converting enzyme 1	KPQQFFGL
endothelin-converting enzyme 1	PQQFFGLM
endothelin-converting enzyme 1	DIIWINTP
Kell blood-group protein	DIIWINTP
endothelin-converting enzyme 2	DIIWINTP
neprilysin	KPQQFFGL
neprilysin	PQQFFGLM
endothelin-converting enzyme 1	KPQQFFGL
endothelin-converting enzyme 1	PQQFFGLM
PHEX peptidase	DHLSDTST
endothelin-converting enzyme 1	DIIWVNTP
neprilysin-2	DIIWVNTP
Kell blood-group protein	DIIWVNTP
endothelin-converting enzyme 1	DIIWVNTP
Kell blood-group protein	DIIWVNTP
endothelin-converting enzyme 1	CHLDIIWV
endothelin-converting enzyme 1	HLDIIWVN
endothelin-converting enzyme 1	CVYFCHLD
endothelin-converting enzyme 1	ECVYFCHL
endothelin-converting enzyme 2	DIIWVNTP
endothelin-converting enzyme 1	DIIWINTP
Kell blood-group protein	DIIWINTP
endothelin-converting enzyme 2	DIIWINTP
PHEX peptidase	DHLSDTST
nardilysin	YILKRDSY
insulysin	HLVEALYL
mitochondrial processing peptidase beta-subunit	LRRSFSTS

caspase-3	DSPDSVDP
caspase-3	DEIDHAEM
caspase-1	DEIDHAEM
caspase-3	VDFDDIHR
caspase-3	EECDAAEG
caspase-7	EECDAAEG
caspase-8	EECDAAEG
caspase-3	LHTDSRKD
caspase-1	LHTDSRKD
caspase-7	SEVDGNDS
caspase-3	CSTDSPLP
caspase-2	CSTDSPLP
caspase-2	SEVDGNDS
caspase-2	ESPDGPGQ
caspase-1	YVHDAPVR
caspase-1	FEADGPKQ
caspase-3	SDSDGLAP
caspase-3	NPQDSVGS
caspase-3	SELDRLLL
caspase-3	FPADEANS
caspase-3	SLLDELES
caspase-3	SQLDSMLG
caspase-3	DDLDALLA
caspase-3	LISDTYLA
caspase-3	DLPDCEAD
caspase-3	DEEDLQRA
caspase-3	VEVDPMLT
caspase-3	DELDYHRG
caspase-3	EGEDDRDS
caspase-3	DEQDGASA
caspase-8	VETDSEEQ
caspase-3	DTRDNVYY
caspase-3	DESDSEGS
caspase-3	EEIDHAER
caspase-1	EEIDHAER
caspase-7	EEIDHAER
caspase-3	SELDASKT
caspase-1	ELPDGQVI
caspase-3	ELPDGQVI
caspase-3	SDSDGLAP
caspase-3	DEEDMDDV
caspase-3	DMDDVVDA
caspase-8	VETDSEEQ
caspase-3	SELDRLLL
caspase-3	FPADEANS

mitochondrial processing peptidase beta-subunit	LRRSFSTS
insulysin	GRMDRIGA
insulysin	RSSCFGGR
insulysin	YTRLRKQM
mitochondrial processing peptidase beta-subunit	PRRASSNQ
mitochondrial processing peptidase beta-subunit	GRRGLRTD
insulysin	YTRLRKQM
mitochondrial processing peptidase beta-subunit	MVRNFRCG
insulysin	LVTLFKNA
insulysin	VTLFKNAI
nardilysin	PRERKAGC
insulysin	VHHQKLVF
insulysin	YEVHHQKL
insulysin	EVHHQKLV
insulysin	HQKLVFFA
insulysin	HHQKLVFF
insulysin	GSNKGAII
insulysin	DAEFRHDS
insulysin	KLVFFAED
insulysin	QKLVFFAE
insulysin	EVHHQKLV
insulysin	LVTLFKNA
insulysin	VTLFKNAI
insulysin	VHHQKLVF
insulysin	YEVHHQKL
insulysin	EVHHQKLV
insulysin	HQKLVFFA
insulysin	HHQKLVFF
insulysin	GSNKGAII
insulysin	DAEFRHDS
insulysin	KLVFFAED
insulysin	QKLVFFAE
insulysin	EVHHQKLV
insulysin	VHHQKLVF
insulysin	YEVHHQKL
insulysin	EVHHQKLV
insulysin	HQKLVFFA
insulysin	HHQKLVFF
insulysin	GSNKGAII
insulysin	DAEFRHDS
insulysin	KLVFFAED
insulysin	QKLVFFAE
insulysin	EVHHQKLV
insulysin	GFLRRIRP
eupitrilysin	KVVTRSQE

caspase-3	SLLDELES
caspase-3	SQLDSMLG
caspase-7	EDLDGKGS
caspase-8	EDLDGKGS
caspase-9	EDLDGKGS
caspase-10	EDLDGKGS
caspase-3	EDLDGKGS
caspase-6	VKMDAEFR
caspase-6	VEVDAAVT
caspase-3	VEVDAAVT
caspase-3	YVPDSPAL
caspase-1	YVPDSPAL
caspase-1	SEPDSPVF
caspase-6	TEEDGVPS
caspase-8	TEEDGVPS
caspase-9	TEEDGVPS
caspase-3	YVPDSPAL
caspase-1	YVPDSPAL
caspase-3	DMVDSPQL
caspase-1	DMVDSPQL
caspase-7	DMVDSPQL
caspase-6	DMVDSPQL
caspase-8	DMVDSPQL
caspase-6	IQFDSEKG
caspase-3	YVPDSPAL
caspase-1	YVPDSPAL
caspase-3	YVPDSPAL
caspase-1	YVPDSPAL
caspase-1	SEPDSPVF
caspase-3	SDSDGLAP
caspase-3	VDFDDIHR
caspase-6	VKMDAEFR
caspase-6	VEVDAAVT
caspase-3	VEVDAAVT
caspase-7	DQTDSWNH
caspase-6	VSWDSGGS
caspase-3	DQTDSWNH
caspase-8	VETDSEEQ
caspase-3	YVPDSPAL
caspase-1	YVPDSPAL

insulysin	HLVEALYL
mitochondrial processing peptidase beta-subunit	PRRASSNQ
mitochondrial processing peptidase beta-subunit	GRRGLRTD
angiotensin-converting enzyme peptidase unit 2	VVHFFKNI
angiotensin-converting enzyme peptidase unit 2	VVHFFKNI
angiotensin-converting enzyme peptidase unit 2	VVHFFKNI
angiotensin-converting enzyme peptidase unit 2	VVHFFKNI
angiotensin-converting enzyme peptidase unit 2	VVHFFKNI
thimet oligopeptidase	NKPRRPYI
thimet oligopeptidase	PPGFSPFR
mitochondrial intermediate peptidase	PPGFSPFR
thimet oligopeptidase	EVKMDAEF
thimet oligopeptidase	EVKMDAEF
thimet oligopeptidase	EVKMDAEF
thimet oligopeptidase	PPGFSPFR
mitochondrial intermediate peptidase	PPGFSPFR
pappalysin-1	YERFNFDG
pappalysin-1	GGKMKVNG
pappalysin-1	TQSKFVGG
pappalysin-1	LTQSKFVG
pappalysin-2	LTQSKFVG
S2P peptidase	RILLCVLT
S2P peptidase	LTFLCLSF

Aspartate Protease

gastricsin	FGDLSVTY
gastricsin	LGEFLRTH
cathepsin D	FQEAYRRF
cathepsin D	SHCLLVTL
cathepsin D	LVTLAAHL
cathepsin D	STVLTSKY
cathepsin D	AEALERMF
cathepsin D	LERMFLSF
cathepsin D	LDKFLASV
cathepsin D	ERMFLSFP
cathepsin D	FLSFPTTK
cathepsin D	ASVSTVLT
cathepsin D	LLVTLAAH
pepsin A	GSHLVEAL

caspase-6	VKMDAEFR
caspase-6	VEVDAAVT
caspase-3	VEVDAAVT
caspase-3	YVPDSPAL
caspase-1	YVPDSPAL
caspase-3	YPPDYYGY
caspase-3	GYEDYYDY
caspase-3	DYYDYYGY
caspase-3	MMPDGTLG
caspase-1	ELPDGQVI
caspase-3	ELPDGQVI
caspase-3	DEQDGASA
caspase-3	DIVDRGST
caspase-3	DRVDENNP
caspase-3	SSTDSAAS
caspase-3	AEVDGDDD
caspase-3	DGDDDAEE
caspase-3	DIVDRGST
caspase-3	DRVDENNP
caspase-3	DDSDAATF
caspase-1	AVQDNPAM
caspase-3	ESVDGQVV
caspase-7	ESVDGQVV
caspase-6	VEMDAAPG
caspase-6	VEMDAAPG
caspase-3	PHLDGPPS
caspase-7	LTEDHLDL
caspase-7	DHLDLNNA
caspase-3	TEIDGRSI
caspase-3	AMEDGEID
caspase-3	GEIDGNKV
caspase-3	TVADGLKK
caspase-3	EEMDFRSG
caspase-3	ECVDSERR
caspase-8	VETDSEEQ
caspase-3	DESDSEGS
caspase-3	PAPDAPLK
caspase-3	YWIDGSNR
caspase-3	YWIDGSNR
caspase-3	DSVDAKPD
caspase-7	DSVDAKPD
caspase-3	SSTDSAAS
caspase-3	SVTDSVMG
caspase-3	TVADGLKK
caspase-3	EEMDFRSG
caspase-3	ECVDSERR
caspase-8	VETDSEEQ
caspase-6	VEIDNGKQ
caspase-3	DGVDLKTQ

pepsin A	ERGFFYTP
gastricsin	HLVEALYL
gastricsin	LVEALYLV
memapsin-2	ASNLSVNY
memapsin-1	GLALALEP
cathepsin D	LGRLLVVY
cathepsin D	GRLLVVYP
cathepsin D	TQRFFESF
cathepsin D	FESFGDLS
cathepsin D	FIVGFTRQ
cathepsin D	ANPKQTWV
cathepsin D	HPKFIVGF
cathepsin D	KQTWVKYI
cathepsin D	WVKYIVRL
cathepsin D	SGGKMKVN
cathepsin D	ITKLNAEN
cathepsin D	AGKKYFID
cathepsin D	FIDFVARE
memapsin-2	GTQHGIRL
memapsin-2	SSNFAVGA
memapsin-2	GLAYAEIA
memapsin-2	EFEFEAHR
memapsin-2	GEDFTSVV
memapsin-2	TIFLQVVD
chymosin	HPSFIAIP
cathepsin D	TAEEKAAV
cathepsin D	RPFLVVIF
cathepsin D	AIKFFSAQ
cathepsin D	IKFFSAQT
cathepsin D	PKELWVQQ
cathepsin D	KFLASLLE
cathepsin D	TTELFSPV
cathepsin D	DGHFLREP
cathepsin D	FSHFIRSG
cathepsin D	VVIATVIV
cathepsin D	IIGLMVGG
cathepsin D	VITLVMLK
cathepsin D	KLVFFAED
cathepsin D	LVFFAEDV
cathepsin D	TYKFFEQM
cathepsin D	VIATVIVI
cathepsin D	IVITLVML
memapsin-2	EVKMDAEF
cathepsin D	LGDFFRKS
cathepsin D	IKDFLRNL
cathepsin D	GNFKSQLQ
cathepsin D	WGTFEEVS
cathepsin D	LGEFVSET
cathepsin D	GMELIVSQ

caspase-3	EEIDHAER
caspase-1	EEIDHAER
caspase-7	EEIDHAER
caspase-3	PHLDGPPS
caspase-3	DLLDDGEI
caspase-3	DVLDVLNE
caspase-3	DHVDLSLS
caspase-3	SMTDFYHS
caspase-3	DSVDAKPD
caspase-7	DSVDAKPD
caspase-6	IQADSGPI
caspase-6	NDTDANPR
caspase-3	DRLDRART
caspase-3	DLLDAFKE
caspase-3	DGLDGPTY
caspase-3	EEIDHAER
caspase-1	EEIDHAER
caspase-7	EEIDHAER
caspase-3	DCRDGYYE
caspase-3	YPPDYYGY
caspase-3	DYYDDYYG
caspase-3	DYHDYRGG
caspase-3	DHVDLSLS
caspase-3	SMTDFYHS
caspase-3	DHVDGQIL
caspase-7	DHVDGQIL
caspase-8	LEVDGPAM
caspase-3	DETDSKTA
caspase-3	DSLDSVEA
caspase-1	DETDSKTA
caspase-4	DETDSKTA
caspase-3	DQLDAISS
caspase-9	PEPDATPF
caspase-3	TVADGLKK
caspase-3	EEMDFRSG
caspase-3	ECVDSERR
caspase-3	DEQDGASA
caspase-6	VEIDNGKQ
caspase-3	AVVDGCGK
caspase-3	DLLDDGEI
caspase-3	DVLDVLNE
caspase-3	DQTDGLGL
caspase-3	VEVDAPKS
caspase-7	EVDAPKSQ
caspase-6	EVDAPKSQ
caspase-3	DSVDAKPD
caspase-7	DSVDAKPD
caspase-6	IQADSGPI
caspase-6	NDTDANPR

cathepsin D	YPVWSGLP
cathepsin D	NEIYPVWS
cathepsin D	RLRAYLLP
cathepsin D	LKFLNVLS
cathepsin D	SQRYKVDY
cathepsin D	KVDYESQS
cathepsin D	TMTLSKST
cathepsin D	NYFLDVEL
cathepsin D	ALDFAVGE
cathepsin D	FQIYAVPW
napsin A	KLVLPVLP
renin	PFHLVIHN
cathepsin D	VQAAYQKV
cathepsin D	VTALWGKV
cathepsin D	KGTFATLS
cathepsin D	TALWGKVN
pepsin A	PWILTSLH
chymosin	RVGFYESD
chymosin	VRKYFPET
chymosin	TETVRKYF
cathepsin D	KDVLDSVL
cathepsin D	VEDLESVG
cathepsin D	LLKEAQLP
cathepsin D	VVLLPDVE
cathepsin D	DVVLFEKK
cathepsin D	LLSALVET
cathepsin D	ITLLSALV
cathepsin D	LSALVETR
presenilin 1	LTFAVYVR
presenilin 1	SLLALALI
presenilin 1	AAFLVTLL
presenilin 1	FLVTLLIY
presenilin 1	AFLVTLLI
presenilin 1	LLFLRRRA
presenilin 1	GGVVIATV
presenilin 1	YVAAAAFv
presenilin 1	VGCGVLLS
presenilin 1	GALLVLQL

caspase-3	EEADSMKS
caspase-3	ESVDKSAG
caspase-3	DINDGHCG
caspase-3	DRLDRART
caspase-3	DLLDAFKE
caspase-3	ESVDYRAT
caspase-3	DGVDLKTQ
caspase-3	EETDGIAY
caspase-3	DGMDGIKI
caspase-7	EETDGIAY
caspase-7	DSEDLKPV
caspase-6	EETDGIAY
caspase-8	EETDGIAY
caspase-8	LEVDGPAM
caspase-1	ELPDGQVI
caspase-3	ELPDGQVI
caspase-3	VDFDDIHR
caspase-3	DAGDVGAA
caspase-3	DQLDAISS
caspase-9	PEPDATPF
caspase-3	VDFDDIHR
caspase-3	DLKDHMRE
caspase-3	GWADERGG
caspase-3	VDFDDIHR
caspase-3	DFPDWWQV
caspase-3	DITDCPRT
caspase-3	DEVDSLMC
caspase-6	VEIDNGKQ
caspase-3	DVTDYKGE
caspase-1	HLADSPAV
caspase-3	HLADSPAV
caspase-3	SSLDAREV
caspase-3	DSVDAKPD
caspase-7	DSVDAKPD
caspase-6	IQADSGPI
caspase-6	NDTDANPR
caspase-3	DYPDSSVS
caspase-3	DMVDSPQL
caspase-1	DMVDSPQL
caspase-7	DMVDSPQL
caspase-6	DMVDSPQL
caspase-8	DMVDSPQL
caspase-3	DRLDRART
caspase-3	DLLDAFKE
caspase-3	YPPDYYGY
caspase-3	DYYDDYYG
caspase-3	DYHDYRGG
caspase-3	RAIDALRE
caspase-3	KESDLSHV

caspase-3	DKTDISSG
caspase-3	TEVDFNKS
caspase-3	EERDGSLN
caspase-3	DEVDSKRL
caspase-3	ETVDTSEM
caspase-3	DEVDNKVK
caspase-3	DDVDTKKQ
caspase-3	DDEDDDVD
caspase-3	DEDDDVDT
caspase-3	VEVDPMLT
caspase-3	DVTDYKGE
caspase-3	DETDSSSA
caspase-3	EERDGSLN
caspase-3	NKTDPRSM
caspase-3	EGEDDRDS
caspase-3	GEDDRDSA
caspase-3	DDRDSANG
caspase-6	TEEDGVPS
caspase-8	TEEDGVPS
caspase-9	TEEDGVPS
caspase-1	LVVDNGSG
caspase-1	ELPDGQVI
caspase-3	ELPDGQVI
caspase-3	DRLDRART
caspase-3	DLLDAFKE
caspase-1	AVQDNPAM
caspase-3	DVVDNQTE
caspase-3	TEVDAASV
caspase-1	ELPDGQVI
caspase-3	ELPDGQVI
caspase-3	ATHDGPQS
caspase-3	DFLDNERH
caspase-3	LGTDSDSS
caspase-3	TDSDSSPQ
caspase-3	EETDGIAY
caspase-3	DGMDGIKI
caspase-7	EETDGIAY
caspase-7	DSEDLKPV
caspase-6	EETDGIAY
caspase-8	EETDGIAY
caspase-3	DQTDGLGL
caspase-3	DRHDSGLD
caspase-3	DEVDSKRL
caspase-3	DTYDALHM
caspase-3	GLLDPKLC
caspase-3	YLLDGILF
caspase-3	DCRDGFYE
caspase-6	VFTDLASV
caspase-3	DETDSSSA

caspase-3	YPPDYYGY
caspase-3	DYYDDYYG
caspase-3	DYHDYRGG
caspase-3	DETDGNLP
caspase-3	NKTDPRSM
caspase-3	EGEDDRDS
caspase-3	GEDDRDSA
caspase-3	DDRDSANG
caspase-3	GSSDPLIQ
caspase-3	DMVDSPQL
caspase-1	DMVDSPQL
caspase-7	GSSDPLIQ
caspase-7	DMVDSPQL
caspase-6	DMVDSPQL
caspase-8	GSSDPLIQ
caspase-8	DMVDSPQL
caspase-3	DGFDGATA
caspase-7	DGFDGATA
caspase-3	DLRDDKDT
caspase-3	DLFDLTSG
caspase-3	DEDDDDEE
caspase-3	EVPDGAVK
caspase-3	DAQDGNQP
caspase-3	WEIDNNPK
caspase-7	EVPDGAVK
caspase-7	DAQDGNQP
caspase-7	WEIDNNPK
caspase-3	DGFDGATA
caspase-7	DGFDGATA
caspase-3	PHLDGPPS
caspase-3	DIPDGLFL
caspase-1	AVQDNPAM
caspase-1	WFKDSVGV
caspase-1	FEDDAIKK
caspase-3	DRLDRART
caspase-3	DLLDAFKE
caspase-3	FIQDRAGR
caspase-3	DGFDGATA
caspase-7	DGFDGATA
caspase-1	ALDDLIDT
caspase-1	LSSDFTCG
caspase-1	ALADSLGK
caspase-3	LSSDFTCG
caspase-3	ATHDGPQS
caspase-3	DFLDNERH
caspase-3	LGTDSDSS
caspase-3	TDSDSSPQ
caspase-3	DETDSGAG
caspase-3	DAVDTGIS

caspase-3	LEVDCYRA
caspase-6	LEVDCYRA
caspase-8	LEVDCYRA
caspase-3	SALDGDQM
caspase-3	DEDDDDEE
caspase-3	EETDGIAY
caspase-3	DGMDGIKI
caspase-7	EETDGIAY
caspase-7	DSEDLKPV
caspase-6	EETDGIAY
caspase-8	EETDGIAY
caspase-3	DEMDSGTM
caspase-3	TMTDGANT
caspase-6	VEMDSLSE
caspase-3	DESDSEGS
caspase-6	VEMDAAPG
caspase-6	VEMDAAPG
caspase-3	EVPDGAVK
caspase-3	WEIDNNPK
caspase-7	EVPDGAVK
caspase-7	WEIDNNPK
caspase-3	NKTDPRSM
caspase-3	EGEDDRDS
caspase-3	GEDDRDSA
caspase-3	DDRDSANG
caspase-3	DIVDRGST
caspase-3	DRVDENNP
caspase-3	DDSDAATF
caspase-3	DESDFGPL
caspase-3	DETDSGAG
caspase-3	DAVDTGIS
caspase-3	DEVDGVDE
caspase-1	DEVDGVDE
caspase-3	DLRDDKDT
caspase-3	DTYDALHM
caspase-3	GLLDPKLC
caspase-3	YLLDGILF
caspase-1	ELPDGQVI
caspase-3	ELPDGQVI
caspase-1	NMQDSQGV
caspase-1	AVQDNPAM
caspase-1	WFKDSVGV
caspase-1	FEDDAIKK
caspase-3	SYNDFGNY
caspase-1	ALDDLIDT
caspase-1	LSSDFTCG
caspase-1	ALADSLGK
caspase-3	LSSDFTCG
caspase-3	EVPDGAVK

caspase-3	DAQDGNQP
caspase-3	WEIDNNPK
caspase-7	EVPDGAVK
caspase-7	DAQDGNQP
caspase-7	WEIDNNPK
caspase-3	AETDGQAS
caspase-7	AETDGQAS
caspase-8	AETDGQAS
caspase-6	VEMDSLSE
caspase-3	NKTDPRSM
caspase-3	EGEDDRDS
caspase-3	GEDDRDSA
caspase-3	DDRDSANG
caspase-3	DIVDRGST
caspase-3	DRVDENNP
caspase-3	SYNDFGNY
caspase-3	DEMDEKSE
caspase-3	DEIDVVPE
caspase-3	MDVDNSKN
caspase-3	SYNDFGNY
caspase-3	DELDSHTM
caspase-7	DQTDSWNH
caspase-6	VSWDSGGS
caspase-3	DQTDSWNH
caspase-3	SALDGDQM
caspase-3	LSVDRGFG
caspase-6	VEMDAAPG
caspase-6	VEMDAAPG
caspase-3	AETDGQAS
caspase-7	AETDGQAS
caspase-8	AETDGQAS
caspase-3	YVPDSPAL
caspase-1	YVPDSPAL
caspase-3	VASDGVVA
caspase-3	EAVDGECP
caspase-1	ALADSLGK
caspase-3	DQTDGLGL
caspase-3	DQTDGLGL
caspase-8	ILRDKDNT
caspase-3	DVPDCKKT
caspase-7	DVPDCKKT
caspase-6	DVPDCKKT
caspase-3	DAGDVGAA
caspase-3	DQTDGLGL
caspase-3	EEVDLNAG
caspase-3	EQEDSSSA
ubiquitin-specific peptidase 4	LRGGIIEP

ubiquitin-specific peptidase 4	LRGGIIEP
ubiquitin-specific peptidase 4	LRGGMQIF
ubiquitin-specific peptidase 15	LRGGMQIF
ubiquitin-specific peptidase 5	LRGGMQIF
ubiquitin-specific peptidase 4	LRGGIIEP
ubiquitin-specific peptidase 4	LRGGIIEP
ubiquitin-specific peptidase 4	LRGGMQIF
ubiquitin-specific peptidase 15	LRGGMQIF
ubiquitin-specific peptidase 5	LRGGMQIF
calpain-2	LILQLCIG
calpain-2	RVNKLILQ
calpain-2	AKLAKDRE
calpain-2	EGLGSHER
calpain-1	EKSTASWA
calpain-1	TASWAERF
calpain-1	EKSTASWA
calpain-1	TASWAERF
calpain-1	LLLQGFIQ
calpain-2	LILQLCIG
calpain-2	RVNKLILQ
calpain-1	QEVYGMMP
calpain-1	HLVEALYL
calpain-2	PDLKNVKS
calpain-2	KNVKSKIG
calpain-2	LILQLCIG
calpain-2	RVNKLILQ
calpain-1	VTPRTPPP
calpain-1	KNIVTPRT
calpain-2	VTPRTPPP
calpain-2	KNIVTPRT
calpain-1	HQTQAGKP
calpain-1	LLLQGFIQ
calpain-2	EKLKSQWN
calpain-2	TTVMNPKF
calpain-2	NPLFKSAT
calpain-2	LILQLCIG
calpain-2	RVNKLILQ
calpain-1	MKVNGAPR
calpain-1	AEIEAIQE
calpain-1	QKHFAKIR

calpain-2	EKLKSQWN
calpain-2	TTVMNPKF
calpain-2	NPLFKSAT
calpain-2	QQRNRENL
calpain-2	LRKGRDPP
calpain-2	ENLLRKGR
calpain-2	AVVRTPPK
calpain-2	AGLKESPL
calpain-2	PDLKNVKS
calpain-2	KNVKSKIG
calpain-2	ENLKHQPG
calpain-2	VEVKSEKL
calpain-2	FEVMEDHA
calpain-2	QIVYKPVD
calpain-2	EIVYKSPV
calpain-1	LLLQGFIQ
calpain-1	RELRRGQI
calpain-1	RGLNRIQT
calpain-1	SPFRSSRI
calpain-1	ISLMKRPP
calpain-2	SPFRSSRI
calpain-2	ISLMKRPP
calpain-1	SRLGRIEA
calpain-1	WEGYDELQ
calpain-1	SHSSSQVS
calpain-1	SALTASVK
calpain-2	SHSSSQVS
calpain-2	SALTASVK
calpain-1	QALHSQPA
calpain-1	RELRRGQI
calpain-1	RGLNRIQT
calpain-1	SRLGRIEA
calpain-1	WEGYDELQ
calpain-2	WHLADSPA
calpain-1	EGTESEME
calpain-1	RELRRGQI
calpain-1	RGLNRIQT
calpain-1	RELRRGQI
calpain-1	RGLNRIQT
calpain-2	ENLAKEKE
calpain-2	SNIRANFE
calpain-2	AYQKTVPV
calpain-2	VTSKTSNI
calpain-2	ENLAKEKE
calpain-2	SNIRANFE
calpain-2	AYQKTVPV
calpain-2	VTSKTSNI
calpain-1	KWDTANNP
calpain-1	EERARAKW

calpain-1	TSTFTNIT
calpain-1	KWDTANNP
calpain-1	NPLYKEAT
calpain-2	AVVRTPPK
calpain-2	PDLKNVKS
calpain-2	KNVKSKIG
calpain-2	ENLKHQPG
calpain-2	VEVKSEKL
calpain-2	FEVMEDHA
calpain-2	QIVYKPVD
calpain-2	EIVYKSPV
calpain-1	RGLNRIQT
calpain-2	VRLRSSVP
calpain-2	YSLGSALR
calpain-2	YVTTSTRT
calpain-2	TRTYSLGS
calpain-1	ETVFSVDE
calpain-2	VLTGKLTT
calpain-2	VDGTSHVT
calpain-2	VETVFSVD
calpain-2	AVVRTPPK
calpain-2	AGLKESPL
calpain-2	PDLKNVKS
calpain-2	KNVKSKIG
calpain-2	ENLKHQPG
calpain-2	VEVKSEKL
calpain-2	FEVMEDHA
calpain-2	QIVYKPVD
calpain-2	EIVYKSPV
calpain-1	QALHSQPA
calpain-1	PESKATNA
calpain-1	SINKSSPL
calpain-2	PDLKNVKS
calpain-2	KNVKSKIG
calpain-1	RGLNRIQT
calpain-2	MAEQHGAP
calpain-2	AEQHGAPE
calpain-1	RELGLGRH
calpain-1	TGVSAQVQ
calpain-1	FWKTFTSC
calpain-1	RELRRGQI
calpain-1	RGLNRIQT
calpain-1	KPNGIFKG
calpain-1	RRLLQERE
calpain-1	DDTFLPVP
calpain-1	NSTFDSPA
calpain-1	QSCPIKED
calpain-1	LWIPEGEK
calpain-1	PSTSRTPL

calpain-1	KRCFFGAS
calpain-1	GPSSSPIP
calpain-2	KRCFFGAS
calpain-2	GPSSSPIP
calpain-2	AVVRTPPK
calpain-2	PDLKNVKS
calpain-2	KNVKSKIG
calpain-2	ENLKHQPG
calpain-2	VEVKSEKL
calpain-2	FEVMEDHA
calpain-2	QIVYKPVD
calpain-2	EIVYKSPV
calpain-2	APQYTYAQ
calpain-2	APQYTYAQ
calpain-1	LSTFAQPP
calpain-1	SEDRKQPS
calpain-1	SEDRKQPS
calpain-1	EKAKLGPA
calpain-1	AGNKVISP
calpain-1	AGNKVISP
calpain-2	EKAKLGPA
calpain-2	TVCGTPGY
calpain-1	GFLRRIRP
calpain-1	HLVEALYL
calpain-1	SPFRSSRI
calpain-1	ISLMKRPP
calpain-2	SPFRSSRI
calpain-2	ISLMKRPP
calpain-1	RELRRGQI
calpain-1	RGLNRIQT
calpain-1	GSLFSVPS
calpain-1	RELRRGQI
calpain-1	RGLNRIQT
calpain-1	QEVYGMMP
calpain-1	GSLFSVPS
calpain-2	DQLDAISS
calpain-2	RPEIRKPE
calpain-1	VTPRTPPP
calpain-1	KNIVTPRT
calpain-2	VTPRTPPP
calpain-2	KNIVTPRT
calpain-2	GGSGTSSR
calpain-1	PTTKMAQT
calpain-2	DQLDAISS
calpain-2	RPEIRKPE
calpain-2	LILQLCIG
calpain-2	RVNKLILQ
calpain-2	LILQLCIG
calpain-2	RVNKLILQ

calpain-2	WHLADSPA
calpain-1	EGTESEME
calpain-2	AVVRTPPK
calpain-2	AGLKESPL
calpain-2	PDLKNVKS
calpain-2	KNVKSKIG
calpain-2	ENLKHQPG
calpain-2	VEVKSEKL
calpain-2	FEVMEDHA
calpain-2	QIVYKPVD
calpain-2	EIVYKSPV
calpain-1	EKSAATWD
calpain-1	VTPRTPPP
calpain-1	KNIVTPRT
calpain-2	VTPRTPPP
calpain-2	KNIVTPRT
calpain-2	PDLKNVKS
calpain-2	KNVKSKIG
calpain-1	EKSAATWD
calpain-1	VTPRTPPP
calpain-1	KNIVTPRT
calpain-2	VTPRTPPP
calpain-2	KNIVTPRT
calpain-2	AVVRTPPK
calpain-2	AGLKESPL
calpain-2	PDLKNVKS
calpain-2	KNVKSKIG
calpain-2	ENLKHQPG
calpain-2	VEVKSEKL
calpain-2	FEVMEDHA
calpain-2	QIVYKPVD
calpain-2	EIVYKSPV
calpain-3	ISVDRPVK
calpain-1	LLLQGFIQ
calpain-2	LILQLCIG
calpain-2	RVNKLILQ
calpain-1	KKLTVNPG
calpain-2	VELNTQAL
calpain-2	SVLKGAKP
calpain-2	PEVTGEPV
calpain-2	LILQLCIG
calpain-2	RVNKLILQ
calpain-2	GGSGTSSR
calpain-2	PDLKNVKS
calpain-2	KNVKSKIG
calpain-3	ISVDRPVK
calpain-1	WSYGLRPG
calpain-1	QSLGSFVH
calpain-1	KKLTQSKF

calpain-1	AENTAHPR
calpain-1	WSYGLRPG
calpain-1	VTPRTPPP
calpain-1	KNIVTPRT
calpain-2	VTPRTPPP
calpain-2	KNIVTPRT
Sonic hedgehog protein	KSGGCFPG
Indian hedgehog protein	KTGGCFPA
SENP1 peptidase	QTGGHSTV
SENP1 peptidase	QTGGHSTV
SENP1 peptidase	QTGGHSTV
SENP8 peptidase	LRGGGGLR
separase	EIMRTIPE
separase	EILRGSDG
separase	ELLRLDSS
separase	EIMREGSA
separase	EPSRLQES
autophagin-1	ETFGMKLS
