# Supplementary material for: A Sequence and Structure Based Method to Predict Putative Substrates, Functions and Regulatory Networks of Endo Proteases
Source: PLoS One. 2009 May 27;4(5):e5700. doi: 10.1371/journal.pone.0005700 (PMC2683571; doi:10.1371/journal.pone.0005700)
Supplement: Table S2 — Natural Substrates of Furin and Thrombin identified using octapeptide training set (0.54 MB PDF) [file pone.0005700.s003.pdf]

Table S2 - Natural Substrates of Furin and Thrombin identified using octapeptide training set.

Furin

Protein Name and Accession Number	Cleavage Sequence		
tr|A6NBZ8|A6NBZ8|HUMAN Putative uncharacterized protein ALB OS=Homo sapiens GN=ALB PE=4 SV=2 *	VFRRDAHK		
tr|B2RBS8|B2RBS8|HUMAN cDNA| FLJ95666| highly similar to Homo sapiens albumin (ALB)| mRNA OS=Homo sapiens PE=2 SV=1 *	VFRRDAHK		
sp|O60494|CUBN|HUMAN Cubilin OS=Homo sapiens GN=CUBN PE=1 SV=3	RQKRSINL		
sp|O75173|ATS4|HUMAN A disintegrin and metalloproteinase with thrombospondin motifs 4 OS=Homo sapiens GN=ADAMTS4 PE=1 SV=2	RAKRFASL		
sp|P00740|FA9|HUMAN Coagulation factor IX OS=Homo sapiens GN=F9 PE=1 SV=2	RPKRYNSG		
sp|P01127|PDGFB|HUMAN Platelet-derived growth factor subunit B OS=Homo sapiens GN=PDGFB PE=1 SV=1	RGRRSLGS		
sp|P01270|PTHY|HUMAN Parathyroid hormone OS=Homo sapiens GN=PTH PE=1 SV=1	VKKRSVSE		
sp|P02768|ALBU|HUMAN Serum albumin OS=Homo sapiens GN=ALB PE=1 SV=2 *	VFRRDAHK		
sp|P04070|PROC|HUMAN Vitamin K-dependent protein C OS=Homo sapiens GN=PROC PE=1 SV=1	HLKRDTED	IRKRANSF	
sp|P05305|EDN1|HUMAN Endothelin-1 OS=Homo sapiens GN=EDN1 PE=1 SV=1	RENRCQCA	RSKRCSCS	
sp|P06213|INSR|HUMAN Insulin receptor OS=Homo sapiens GN=INSR PE=1 SV=2	RKRRSLGD		
sp|P08253|MMP2|HUMAN 72 kDa type IV collagenase OS=Homo sapiens GN=MMP2 PE=1 SV=2	RKPRCGNP		
sp|P13497|BMP1|HUMAN Bone morphogenetic protein 1 OS=Homo sapiens GN=BMP1 PE=1 SV=2	RSRRAATS		
sp|P22388|EDN1|RAT Endothelin-1 OS=Rattus norvegicus GN=Edn1 PE=1 SV=2 *	RSKRCSCS		
sp|P23377|FURIN|RAT Furin OS=Rattus norvegicus GN=Furin PE=1 SV=1	AKQRAKRD		
sp|P26006|ITA3|HUMAN Integrin alpha-3 OS=Homo sapiens GN=ITGA3 PE=1 SV=3	RRRRQLDP		
sp|P49767|VEGFC|HUMAN Vascular endothelial growth factor C OS=Homo sapiens GN=VEGFC PE=1 SV=1	IIRRSLPA		
tr|Q56G89|Q56G89|HUMAN Serum albumin OS=Homo sapiens PE=2 SV=1 *	VFRRDAHK		
sp|Q95ND7|FA9|PANTR Coagulation factor IX OS=Pan troglodytes GN=F9 PE=2 SV=1 *	RPKRYNSG		

Thrombin

Protein Name and Accession Number	Cleavage Sequence		
tr|A0JLU0|A0JLU0|HUMAN TCOF1 protein (Fragment) OS=Homo sapiens GN=TCOF1 PE=2 SV=1	ATPRLAST		
tr|A6NBZ8|A6NBZ8|HUMAN Putative uncharacterized protein ALB OS=Homo sapiens GN=ALB PE=4 SV=2	AVARLSQR		
tr|A8K7E6|A8K7E6|HUMAN cDNA FLJ77695| highly similar to Homo sapiens chaperonin containing TCP1| subunit 7 (eta) (CCT7)|mRNA OS=Homo sapiens GN=CCT7 PE=2 SV=1	MIVRRAIK		
tr|A8MWI8|A8MWI8|HUMAN cDNA FLJ54832| highly similar to T-complex protein 1 subunit eta OS=Homo sapiens GN=CCT7 PE=2 SV=1	MIVRRAIK		
tr|B2R5W2|B2R5W2|HUMAN cDNA| FLJ92657| highly similar to Homo sapiens heterogeneous nuclear ribonucleoprotein C (C1/C2) (HNRPC)| transcript variant 2| mRNA OS=Homo sapiens PE=2 SV=1	SGQRGSSK		
tr|B2RBS8|B2RBS8|HUMAN cDNA| FLJ95666| highly similar to Homo sapiens albumin (ALB)| mRNA OS=Homo sapiens PE=2 SV=1	AVARLSQR		
sp|O43488|ARK72|HUMAN Aflatoxin B1 aldehyde reductase member 2 OS=Homo sapiens GN=AKR7A2 PE=1 SV=3	FGLRFYAY		
sp|P00451|FA8|HUMAN Coagulation factor VIII OS=Homo sapiens GN=F8 PE=1 SV=1	IEPRSFSQ	IQIRSVAK	
sp|P00558|PGK1|HUMAN Phosphoglycerate kinase 1 OS=Homo sapiens GN=PGK1 PE=1 SV=3	AVTRAKQI	FNPRTFGS	IDGRIVEG
sp|P00735|THRB|BOVIN Prothrombin OS=Bos taurus GN=F2 PE=1 SV=2	VIPRSGGS		
sp|P01008|ANT3|HUMAN Antithrombin-III OS=Homo sapiens GN=SERPINC1 PE=1 SV=1	IAGRSLNP		
sp|P01023|A2MG|HUMAN Alpha-2-macroglobulin OS=Homo sapiens GN=A2M PE=1 SV=1	GHARLVHV		
sp|P02671|FIBA|HUMAN Fibrinogen alpha chain OS=Homo sapiens GN=FGA PE=1 SV=2	GGVRGPRV		
sp|P02675|FIBB|HUMAN Fibrinogen beta chain OS=Homo sapiens GN=FGB PE=1 SV=2	FSARGHRP		
sp|P02768|ALBU|HUMAN Serum albumin OS=Homo sapiens GN=ALB PE=1 SV=2 *	AVARLSQR		
sp|P03951|FA11|HUMAN Coagulation factor XI OS=Homo sapiens GN=F11 PE=1 SV=1	IKPRIVGG		

sp|P05388|RLA0|HUMAN 60S acidic ribosomal protein P0 OS=Homo sapiens GN=RPLP0 PE=1 SV=1	PHIRGNVG		
sp|P05455|LA|HUMAN Lupus La protein OS=Homo sapiens GN=SSB PE=1 SV=2	VKNRSVYI		
sp|P06399|FIBA|RAT Fibrinogen alpha chain OS=Rattus norvegicus GN=Fga PE=1 SV=3	GDIRGPRI		
sp|P07224|PROS|BOVIN Vitamin K-dependent protein S OS=Bos taurus GN=PROS1 PE=1 SV=1	GSFRAGLF	PDLRSCVN	
sp|P08709|FA7|HUMAN Coagulation factor VII OS=Homo sapiens GN=F7 PE=1 SV=1	PQGRIVGG		
sp|P14174|MIF|HUMAN Macrophage migration inhibitory factor OS=Homo sapiens GN=MIF PE=1 SV=4	AQNRSYSK		
sp|P17844|DDX5|HUMAN Probable ATP-dependent RNA helicase DDX5 OS=Homo sapiens GN=DDX5 PE=1 SV=1	ITVRGHNC		
sp|P17936|IBP3|HUMAN Insulin-like growth factor-binding protein 3 OS=Homo sapiens GN=IGFBP3 PE=1 SV=2	SRLRAYLL		
sp|P18292|THRB|RAT Prothrombin OS=Rattus norvegicus GN=F2 PE=1 SV=1	IDGRIVEG		
sp|P19221|THRB|MOUSE Prothrombin OS=Mus musculus GN=F2 PE=1 SV=1	IDGRIVEG		
sp|P24593|IBP5|HUMAN Insulin-like growth factor-binding protein 5 OS=Homo sapiens GN=IGFBP5 PE=1 SV=1	AHPRIISA	FRPKHTRI	MVPRAVYL
sp|P25116|PAR1|HUMAN Proteinase-activated receptor 1 OS=Homo sapiens GN=F2R PE=1 SV=2	PRSFLLRN		
sp|P27816|MAP4|HUMAN Microtubule-associated protein 4 OS=Homo sapiens GN=MAP4 PE=1 SV=2	PSSRSPST		
sp|P50502|F10A1|HUMAN Hsc70-interacting protein OS=Homo sapiens GN=ST13 PE=1 SV=2	VQPRAQKI		
sp|P50991|TCPD|HUMAN T-complex protein 1 subunit delta OS=Homo sapiens GN=CCT4 PE=1 SV=4	INVRKGGI		
sp|P52597|HNRPF|HUMAN Heterogeneous nuclear ribonucleoprotein F OS=Homo sapiens GN=HNRNPF PE=1 SV=3	GTARRYIG		
sp|P60709|ACTB|HUMAN Actin| cytoplasmic 1 OS=Homo sapiens GN=ACTB PE=1 SV=1	GRPRHQGV		
sp|P60710|ACTB|MOUSE Actin| cytoplasmic 1 OS=Mus musculus GN=Actb PE=1 SV=1 *****	GRPRHQGV		
sp|P62304|RUXE|HUMAN Small nuclear ribonucleoprotein E OS=Homo sapiens GN=SNRPE PE=1 SV=1	QLGRIMLK		
sp|P62736|ACTA|HUMAN Actin| aortic smooth muscle OS=Homo sapiens GN=ACTA2 PE=1 SV=1	GRPRHQGV		
sp|P63220|RS21|HUMAN 40S ribosomal protein S21 OS=Homo sapiens GN=RPS21 PE=1 SV=1	SILRLAKA		
sp|P63261|ACTG|HUMAN Actin| cytoplasmic 2 OS=Homo sapiens GN=ACTG1 PE=1 SV=1	GRPRHQGV		

sp|P63267|ACTH|HUMAN Actin| gamma-enteric smooth muscle OS=Homo sapiens GN=ACTG2 PE=1 SV=1	GRPRHQGV		
tr|Q56G89|Q56G89|HUMAN Serum albumin OS=Homo sapiens PE=2 SV=1	AVARLSQR		
sp|P68032|ACTC|HUMAN Actin| alpha cardiac muscle 1 OS=Homo sapiens GN=ACTC1 PE=1 SV=1	GRPRHQGV		
sp|P68133|ACTS|HUMAN Actin| alpha skeletal muscle OS=Homo sapiens GN=ACTA1 PE=1 SV=1	GRPRHQGV		
sp|P70670|NACAM|MOUSE Nascent polypeptide-associated complex subunit alpha| muscle-specific form OS=Mus musculus GN=Naca PE=1 SV=1	KAVRALKN		
sp|P85800|VARI|AMBVA Variegin OS=Amblyomma variegatum PE=1 SV=1	AEPKMHKT		
sp|Q04760|LGUL|HUMAN Lactoylglutathione lyase OS=Homo sapiens GN=GLO1 PE=1 SV=4	SDPRGFGH		
sp|Q10713|MPPA|HUMAN Mitochondrial-processing peptidase subunit alpha OS=Homo sapiens GN=PMPCA PE=1 SV=2	KMLRGKPA		
sp|Q12905|ILF2|HUMAN Interleukin enhancer-binding factor 2 OS=Homo sapiens GN=ILF2 PE=1 SV=2	AFPRVKPA		
sp|Q13177|PAK2|HUMAN Serine/threonine-protein kinase PAK 2 OS=Homo sapiens GN=PAK2 PE=1 SV=3	QWARLLQT		
sp|Q13765|NACA|HUMAN Nascent polypeptide-associated complex subunit alpha OS=Homo sapiens GN=NACA PE=1 SV=1	KAVRALKN		
sp|Q15003|CND2|HUMAN Condensin complex subunit 2 OS=Homo sapiens GN=NCAPH PE=1 SV=3	PLPRKAPL		
tr|B2RDL0|B2RDL0|HUMAN cDNA| FLJ96663| Homo sapiens DEAD (Asp-Glu-Ala-Asp) box polypeptide 21 (DDX21)|mRNA (DEAD (Asp-Glu-Ala-Asp) box polypeptide 21| isoform CRA|c) OS=Homo sapiens GN=DDX21 PE=2 SV=1	KRGRAPQV		
tr|Q3ZCU9|Q3ZCU9|HUMAN STIP1 protein OS=Homo sapiens GN=STIP1 PE=2 SV=1	YSNRSAAY		
tr|Q53F64|Q53F64|HUMAN Heterogeneous nuclear ribonucleoprotein AB isoform a variant (Fragment) OS=Homo sapiens PE=2 SV=1	NTGRSRGF		
tr|Q53HV2|Q53HV2|HUMAN Chaperonin containing TCP1| subunit 7 (Eta) variant (Fragment) OS=Homo sapiens PE=2 SV=1	MIVRRAIK		
tr|Q59FH4|Q59FH4|HUMAN Drebrin-like protein variant (Fragment) OS=Homo sapiens PE=2 SV=1	GKLRSPFL		
tr|Q59G96|Q59G96|HUMAN Dynamin 2 isoform 4 variant (Fragment) OS=Homo sapiens PE=2 SV=1	PALRSKLQ		
sp|P31948|STIP1|HUMAN Stress-induced-phosphoprotein 1 OS=Homo sapiens GN=STIP1 PE=1 SV=1 *	YSNRSAAY		
sp|Q5VYJ4|RUEL1|HUMAN Putative small nuclear ribonucleoprotein polypeptide E-like protein 1 OS=Homo sapiens GN=SNRPEL1 PE=5 SV=1	QLGRIMLK		

sp|Q62930|CO9|RAT Complement component C9 OS=Rattus norvegicus GN=C9 PE=2 SV=1	KPKDSSVD		
tr|Q6IBT3|Q6IBT3|HUMAN CCT7 protein OS=Homo sapiens GN=CCT7 PE=2 SV=1	MIVRRAIK		
tr|Q6ZS99|Q6ZS99|HUMAN cDNA FLJ45706 fis| clone FEBRA2028457| highly similar to Nucleolin OS=Homo sapiens PE=2 SV=1	PNARSQPS		
tr|Q7Z7J6|Q7Z7J6|HUMAN Actin alpha 1 skeletal muscle protein OS=Homo sapiens GN=ACTA1 PE=2 SV=1	GRPRHQGV		
tr|Q86Y04|Q86Y04|HUMAN Microtubule-associated protein (Fragment) OS=Homo sapiens GN=MAP4 PE=1 SV=1	PSSRSPST		
tr|Q8N7G1|Q8N7G1|HUMAN cDNA FLJ25678 fis| clone TST04067| highly similar to PURINE NUCLEOSIDE PHOSPHORYLASE (EC 2.4.2.1) OS=Homo sapiens PE=2 SV=1	MRQRALST		
sp|Q96CT7|CC124|HUMAN Coiled-coil domain-containing protein 124 OS=Homo sapiens GN=CCDC124 PE=1 SV=1	KAPRVATS		
sp|Q96IY4|CBPB2|HUMAN Carboxypeptidase B2 OS=Homo sapiens GN=CPB2 PE=1 SV=1	VSPRASAS		
sp|Q99848|EBP2|HUMAN Probable rRNA-processing protein EBP2 OS=Homo sapiens GN=EBNA1BP2 PE=1 SV=2	AFSRGLLK		
tr|Q9BQ02|Q9BQ02|HUMAN NCL protein OS=Homo sapiens PE=2 SV=1 *	PNARSQPS		
sp|Q9H009|NACA2|HUMAN Nascent polypeptide-associated complex subunit alpha-2 OS=Homo sapiens GN=NACA2 PE=1 SV=1	KAVRALKN		
sp|Q9JIK5|DDX21|MOUSE Nucleolar RNA helicase 2 OS=Mus musculus GN=Ddx21 PE=1 SV=2 *	KRGRAPQV		
sp|Q9NX46|ARHL2|HUMAN Poly(ADP-ribose) glycohydrolase ARH3 OS=Homo sapiens GN=ADPRHL2 PE=1 SV=1	AMARALVQ		
tr|Q9Y6B5|Q9Y6B5|HUMAN PAK2 OS=Homo sapiens PE=2 SV=1	QWARLLQT		
sp|Q9Z2X1|HNRPF|MOUSE Heterogeneous nuclear ribonucleoprotein F OS=Mus musculus GN=Hnrnpf PE=1 SV=3 *	GTARRYIG		

Entries marked with star are putative substrates of Furin or Thrombin while all others are reported natural substrates.
