# Supplementary material for: A Sequence and Structure Based Method to Predict Putative Substrates, Functions and Regulatory Networks of Endo Proteases
Source: PLoS One. 2009 May 27;4(5):e5700. doi: 10.1371/journal.pone.0005700 (PMC2683571; doi:10.1371/journal.pone.0005700)
Supplement: Table S3 — Putative Substrates of Aspartate,Cysteine and Serine catalytic type of proteases obtained by matches with Human proteome (0.48 MB PDF) [file pone.0005700.s004.pdf]

Table S3- Putative Substrates of Aspartate,Cysteine, Metallo and Serine catalytic type of proteases obtained by matches with Human proteome

Aspartate Proteases

Name of the Protein	Enzyme	Cleavage Sequence
gi|4505757|ref|NP_002621.1| progastricsin (pepsinogen C)	gastricsin	FGDLSVTY
gi|4505757|ref|NP_002621.1| progastricsin (pepsinogen C)	gastricsin	LGEFLRTH
gi|40316933|ref|NP_954642.1| osteocalcin preproprotein	cathepsin D	FQEAYRRF
gi|40316933|ref|NP_954642.1| osteocalcin preproprotein	cathepsin E	FQEAYRRF
gi|4504345|ref|NP_000508.1| alpha 2 globin	cathepsin D	SHCLLVTL
gi|4504345|ref|NP_000508.1| alpha 2 globin	cathepsin D	LVTLAAHL
gi|4504345|ref|NP_000508.1| alpha 2 globin	cathepsin D	STVLTSKY
gi|4504345|ref|NP_000508.1| alpha 2 globin	cathepsin D	AEALERMF
gi|4504345|ref|NP_000508.1| alpha 2 globin	cathepsin D	LERMFLSF
gi|4504345|ref|NP_000508.1| alpha 2 globin	cathepsin D	LDKFLASV
gi|4504345|ref|NP_000508.1| alpha 2 globin	cathepsin D	ERMFLSFP
gi|4504345|ref|NP_000508.1| alpha 2 globin	cathepsin D	FLSFPTTK
gi|4504345|ref|NP_000508.1| alpha 2 globin	cathepsin D	ASVSTVLT
gi|4504345|ref|NP_000508.1| alpha 2 globin	cathepsin D	LLVTLAAH
gi|4504345|ref|NP_000508.1| alpha 2 globin	cathepsin E	SHCLLVTL
gi|4504345|ref|NP_000508.1| alpha 2 globin	cathepsin E	LVTLAAHL
gi|4504345|ref|NP_000508.1| alpha 2 globin	cathepsin E	STVLTSKY
gi|4504345|ref|NP_000508.1| alpha 2 globin	cathepsin E	AEALERMF
gi|4504345|ref|NP_000508.1| alpha 2 globin	cathepsin E	LERMFLSF
gi|4504345|ref|NP_000508.1| alpha 2 globin	cathepsin E	LDKFLASV
gi|4504345|ref|NP_000508.1| alpha 2 globin	cathepsin E	ERMFLSFP
gi|4504345|ref|NP_000508.1| alpha 2 globin	cathepsin E	FLSFPTTK
gi|4504345|ref|NP_000508.1| alpha 2 globin	cathepsin E	ASVSTVLT
gi|4504345|ref|NP_000508.1| alpha 2 globin	cathepsin E	LLVTLAAH
gi|4557671|ref|NP_000198.1| proinsulin precursor	pepsin A	GSHLVEAL
gi|4557671|ref|NP_000198.1| proinsulin precursor	pepsin A	ERGFFYTP
gi|4557671|ref|NP_000198.1| proinsulin precursor	gastricsin	HLVEALYL
gi|4557671|ref|NP_000198.1| proinsulin precursor	gastricsin	LVEALYLV
gi|4557671|ref|NP_000198.1| proinsulin precursor	gastricsin	ERGFFYTP
gi|4557671|ref|NP_000198.1| proinsulin precursor	cathepsin D	ERGFFYTP
gi|4557671|ref|NP_000198.1| proinsulin precursor	cathepsin E	ERGFFYTP
gi|4506879|ref|NP_002997.1| selectin P ligand	memapsin-2	ASNLSVNY
gi|21040360|ref|NP_620476.1| beta-site APP-cleaving enzyme 2 isoform C preproprotein	memapsin-1	GLALALEP
gi|4504351|ref|NP_000510.1| delta globin	cathepsin D	LGRLLVVY
gi|4504351|ref|NP_000510.1| delta globin	cathepsin D	LGRLLVVY
gi|4504351|ref|NP_000510.1| delta globin	cathepsin D	GRLLVVYP

gi|4504351|ref|NP_000510.1| delta globin	cathepsin D	GRLLVVYP
gi|4504351|ref|NP_000510.1| delta globin	cathepsin D	TQRFFESF
gi|4504351|ref|NP_000510.1| delta globin	cathepsin D	TQRFFESF
gi|4504351|ref|NP_000510.1| delta globin	cathepsin D	FESFGDLS
gi|4504351|ref|NP_000510.1| delta globin	cathepsin D	FESFGDLS
gi|4504351|ref|NP_000510.1| delta globin	cathepsin E	LGRLLVVY
gi|4504351|ref|NP_000510.1| delta globin	cathepsin E	LGRLLVVY
gi|4504351|ref|NP_000510.1| delta globin	cathepsin E	GRLLVVYP
gi|4504351|ref|NP_000510.1| delta globin	cathepsin E	GRLLVVYP
gi|4504351|ref|NP_000510.1| delta globin	cathepsin E	TQRFFESF
gi|4504351|ref|NP_000510.1| delta globin	cathepsin E	TQRFFESF
gi|4504351|ref|NP_000510.1| delta globin	cathepsin E	FESFGDLS
gi|4504351|ref|NP_000510.1| delta globin	cathepsin E	FESFGDLS
gi|4759076|ref|NP_004582.1| chemokine (C-C motif) ligand 20 isoform 1	cathepsin D	FIVGFTRQ
gi|4759076|ref|NP_004582.1| chemokine (C-C motif) ligand 20 isoform 1	cathepsin D	ANPKQTWV
gi|4759076|ref|NP_004582.1| chemokine (C-C motif) ligand 20 isoform 1	cathepsin D	HPKFIVGF
gi|4759076|ref|NP_004582.1| chemokine (C-C motif) ligand 20 isoform 1	cathepsin D	KQTWVKYI
gi|4759076|ref|NP_004582.1| chemokine (C-C motif) ligand 20 isoform 1	cathepsin D	WVKYIVRL
gi|4759076|ref|NP_004582.1| chemokine (C-C motif) ligand 20 isoform 1	cathepsin E	FIVGFTRQ
gi|4759076|ref|NP_004582.1| chemokine (C-C motif) ligand 20 isoform 1	cathepsin E	ANPKQTWV
gi|4759076|ref|NP_004582.1| chemokine (C-C motif) ligand 20 isoform 1	cathepsin E	HPKFIVGF
gi|4759076|ref|NP_004582.1| chemokine (C-C motif) ligand 20 isoform 1	cathepsin E	KQTWVKYI
gi|4759076|ref|NP_004582.1| chemokine (C-C motif) ligand 20 isoform 1	cathepsin E	WVKYIVRL
gi|4885397|ref|NP_005323.1| zeta globin	cathepsin D	SHCLLVTL
gi|4885397|ref|NP_005323.1| zeta globin	cathepsin E	SHCLLVTL
gi|194239688|ref|NP_001123518.1| chemokine (C-C motif) ligand 20 isoform 2	cathepsin D	FIVGFTRQ
gi|194239688|ref|NP_001123518.1| chemokine (C-C motif) ligand 20 isoform 2	cathepsin D	ANPKQTWV
gi|194239688|ref|NP_001123518.1| chemokine (C-C motif) ligand 20 isoform 2	cathepsin D	HPKFIVGF
gi|194239688|ref|NP_001123518.1| chemokine (C-C motif) ligand 20 isoform 2	cathepsin D	KQTWVKYI
gi|194239688|ref|NP_001123518.1| chemokine (C-C motif) ligand 20 isoform 2	cathepsin D	WVKYIVRL
gi|194239688|ref|NP_001123518.1| chemokine (C-C motif) ligand 20 isoform 2	cathepsin E	FIVGFTRQ
gi|194239688|ref|NP_001123518.1| chemokine (C-C motif) ligand 20 isoform 2	cathepsin E	ANPKQTWV

gi|194239688|ref|NP_001123518.1| chemokine (C-C motif) ligand 20 isoform 2	cathepsin E	HPKFIVGF
gi|194239688|ref|NP_001123518.1| chemokine (C-C motif) ligand 20 isoform 2	cathepsin E	KQTWVKYI
gi|194239688|ref|NP_001123518.1| chemokine (C-C motif) ligand 20 isoform 2	cathepsin E	WVKYIVRL
gi|62243290|ref|NP_001543.2| insulin-like growth factor binding protein 4 precursor	cathepsin D	SGGKMKVN
gi|62243290|ref|NP_001543.2| insulin-like growth factor binding protein 4 precursor	cathepsin E	SGGKMKVN
gi|156231037|ref|NP_001095886.1| kininogen 1 isoform 1	cathepsin D	ITKLNAEN
gi|156231037|ref|NP_001095886.1| kininogen 1 isoform 1	cathepsin D	AGKKYFID
gi|156231037|ref|NP_001095886.1| kininogen 1 isoform 1	cathepsin D	FIDFVARE
gi|156231037|ref|NP_001095886.1| kininogen 1 isoform 1	cathepsin E	ITKLNAEN
gi|156231037|ref|NP_001095886.1| kininogen 1 isoform 1	cathepsin E	AGKKYFID
gi|156231037|ref|NP_001095886.1| kininogen 1 isoform 1	cathepsin E	FIDFVARE
gi|6912266|ref|NP_036236.1| beta-site APP-cleaving enzyme 1 isoform A preproprotein	memapsin-2	GTQHGIRL
gi|6912266|ref|NP_036236.1| beta-site APP-cleaving enzyme 1 isoform A preproprotein	memapsin-2	SSNFAVGA
gi|6912266|ref|NP_036236.1| beta-site APP-cleaving enzyme 1 isoform A preproprotein	memapsin-2	GLAYAEIA
gi|93587332|ref|NP_001035241.1| voltage-gated sodium channel beta-3 subunit precursor	memapsin-2	EFEFEAHR
gi|93587332|ref|NP_001035241.1| voltage-gated sodium channel beta-3 subunit precursor	memapsin-2	GEDFTSVV
gi|28372555|ref|NP_777594.1| sodium channel voltage-gated type IV beta	memapsin-2	TIFLQVVD
gi|21040368|ref|NP_620429.1| beta-site APP-cleaving enzyme 1 isoform D preproprotein	memapsin-2	GTQHGIRL
gi|21040368|ref|NP_620429.1| beta-site APP-cleaving enzyme 1 isoform D preproprotein	memapsin-2	SSNFAVGA
gi|148491103|ref|NP_005203.2| casein kappa	chymosin	HPSFIAIP
gi|4885393|ref|NP_005321.1| epsilon globin	cathepsin D	TAEEKAAV
gi|4885393|ref|NP_005321.1| epsilon globin	cathepsin D	LGRLLVVY
gi|4885393|ref|NP_005321.1| epsilon globin	cathepsin D	LGRLLVVY
gi|4885393|ref|NP_005321.1| epsilon globin	cathepsin D	GRLLVVYP
gi|4885393|ref|NP_005321.1| epsilon globin	cathepsin D	GRLLVVYP
gi|4885393|ref|NP_005321.1| epsilon globin	cathepsin E	TAEEKAAV
gi|4885393|ref|NP_005321.1| epsilon globin	cathepsin E	LGRLLVVY
gi|4885393|ref|NP_005321.1| epsilon globin	cathepsin E	LGRLLVVY
gi|4885393|ref|NP_005321.1| epsilon globin	cathepsin E	GRLLVVYP
gi|4885393|ref|NP_005321.1| epsilon globin	cathepsin E	GRLLVVYP
gi|21361302|ref|NP_006206.2| serine (or cysteine) proteinase inhibitor clade A (alpha-1 antiproteinase antitrypsin) member 4	cathepsin D	RPFLVVIF
gi|21361302|ref|NP_006206.2| serine (or cysteine) proteinase inhibitor clade A (alpha-1 antiproteinase antitrypsin) member 4	cathepsin D	AIKFFSAQ

gi|21361302|ref|NP_006206.2| serine (or cysteine) proteinase inhibitor clade A (alpha-1 antiproteinase antitrypsin) member 4	cathepsin D	IKFFSAQT
gi|21361302|ref|NP_006206.2| serine (or cysteine) proteinase inhibitor clade A (alpha-1 antiproteinase antitrypsin) member 4	cathepsin E	RPFLVVIF
gi|21361302|ref|NP_006206.2| serine (or cysteine) proteinase inhibitor clade A (alpha-1 antiproteinase antitrypsin) member 4	cathepsin E	AIKFFSAQ
gi|21361302|ref|NP_006206.2| serine (or cysteine) proteinase inhibitor clade A (alpha-1 antiproteinase antitrypsin) member 4	cathepsin E	IKFFSAQT
gi|4504347|ref|NP_000549.1| alpha 1 globin	cathepsin D	SHCLLVTL
gi|4504347|ref|NP_000549.1| alpha 1 globin	cathepsin D	LVTLAAHL
gi|4504347|ref|NP_000549.1| alpha 1 globin	cathepsin D	STVLTSKY
gi|4504347|ref|NP_000549.1| alpha 1 globin	cathepsin D	AEALERMF
gi|4504347|ref|NP_000549.1| alpha 1 globin	cathepsin D	LERMFLSF
gi|4504347|ref|NP_000549.1| alpha 1 globin	cathepsin D	LDKFLASV
gi|4504347|ref|NP_000549.1| alpha 1 globin	cathepsin D	ERMFLSFP
gi|4504347|ref|NP_000549.1| alpha 1 globin	cathepsin D	FLSFPTTK
gi|4504347|ref|NP_000549.1| alpha 1 globin	cathepsin D	ASVSTVLT
gi|4504347|ref|NP_000549.1| alpha 1 globin	cathepsin D	LLVTLAAH
gi|4504347|ref|NP_000549.1| alpha 1 globin	cathepsin E	SHCLLVTL
gi|4504347|ref|NP_000549.1| alpha 1 globin	cathepsin E	LVTLAAHL
gi|4504347|ref|NP_000549.1| alpha 1 globin	cathepsin E	STVLTSKY
gi|4504347|ref|NP_000549.1| alpha 1 globin	cathepsin E	AEALERMF
gi|4504347|ref|NP_000549.1| alpha 1 globin	cathepsin E	LERMFLSF
gi|4504347|ref|NP_000549.1| alpha 1 globin	cathepsin E	LDKFLASV
gi|4504347|ref|NP_000549.1| alpha 1 globin	cathepsin E	ERMFLSFP
gi|4504347|ref|NP_000549.1| alpha 1 globin	cathepsin E	FLSFPTTK
gi|4504347|ref|NP_000549.1| alpha 1 globin	cathepsin E	ASVSTVLT
gi|4504347|ref|NP_000549.1| alpha 1 globin	cathepsin E	LLVTLAAH
gi|28302131|ref|NP_000550.2| A-gamma globin	cathepsin D	LGRLLVVY
gi|28302131|ref|NP_000550.2| A-gamma globin	cathepsin D	LGRLLVVY
gi|28302131|ref|NP_000550.2| A-gamma globin	cathepsin D	GRLLVVYP
gi|28302131|ref|NP_000550.2| A-gamma globin	cathepsin D	GRLLVVYP
gi|28302131|ref|NP_000550.2| A-gamma globin	cathepsin E	LGRLLVVY
gi|28302131|ref|NP_000550.2| A-gamma globin	cathepsin E	LGRLLVVY
gi|28302131|ref|NP_000550.2| A-gamma globin	cathepsin E	GRLLVVYP
gi|28302131|ref|NP_000550.2| A-gamma globin	cathepsin E	GRLLVVYP
gi|4506835|ref|NP_002980.1| small inducible cytokine A21 precursor	cathepsin D	PKELWVQQ
gi|4506835|ref|NP_002980.1| small inducible cytokine A21 precursor	cathepsin E	PKELWVQQ
gi|55770862|ref|NP_003226.4| thyroglobulin	cathepsin D	KFLASLLE
gi|55770862|ref|NP_003226.4| thyroglobulin	cathepsin D	TTELFSPV
gi|55770862|ref|NP_003226.4| thyroglobulin	cathepsin D	DGHFLREP
gi|55770862|ref|NP_003226.4| thyroglobulin	cathepsin D	FSHFIRSG
gi|55770862|ref|NP_003226.4| thyroglobulin	cathepsin E	KFLASLLE

gi|55770862|ref|NP_003226.4| thyroglobulin	cathepsin E	TTELFSPV
gi|55770862|ref|NP_003226.4| thyroglobulin	cathepsin E	DGHFLREP
gi|55770862|ref|NP_003226.4| thyroglobulin	cathepsin E	FSHFIRSG
gi|4502167|ref|NP_000475.1| amyloid beta A4 protein precursor isoform a	cathepsin D	VVIATVIV
gi|4502167|ref|NP_000475.1| amyloid beta A4 protein precursor isoform a	cathepsin D	IIGLMVGG
gi|4502167|ref|NP_000475.1| amyloid beta A4 protein precursor isoform a	cathepsin D	VITLVMLK
gi|4502167|ref|NP_000475.1| amyloid beta A4 protein precursor isoform a	cathepsin D	KLVFFAED
gi|4502167|ref|NP_000475.1| amyloid beta A4 protein precursor isoform a	cathepsin D	LVFFAEDV
gi|4502167|ref|NP_000475.1| amyloid beta A4 protein precursor isoform a	cathepsin D	TYKFFEQM
gi|4502167|ref|NP_000475.1| amyloid beta A4 protein precursor isoform a	cathepsin D	VIATVIVI
gi|4502167|ref|NP_000475.1| amyloid beta A4 protein precursor isoform a	cathepsin D	IVITLVML
gi|4502167|ref|NP_000475.1| amyloid beta A4 protein precursor isoform a	cathepsin E	VVIATVIV
gi|4502167|ref|NP_000475.1| amyloid beta A4 protein precursor isoform a	cathepsin E	IIGLMVGG
gi|4502167|ref|NP_000475.1| amyloid beta A4 protein precursor isoform a	cathepsin E	VITLVMLK
gi|4502167|ref|NP_000475.1| amyloid beta A4 protein precursor isoform a	cathepsin E	KLVFFAED
gi|4502167|ref|NP_000475.1| amyloid beta A4 protein precursor isoform a	cathepsin E	LVFFAEDV
gi|4502167|ref|NP_000475.1| amyloid beta A4 protein precursor isoform a	cathepsin E	TYKFFEQM
gi|4502167|ref|NP_000475.1| amyloid beta A4 protein precursor isoform a	cathepsin E	VIATVIVI
gi|4502167|ref|NP_000475.1| amyloid beta A4 protein precursor isoform a	cathepsin E	IVITLVML
gi|4502167|ref|NP_000475.1| amyloid beta A4 protein precursor isoform a	memapsin-2	EVKMDAEF
gi|4502167|ref|NP_000475.1| amyloid beta A4 protein precursor isoform a	memapsin-1	KLVFFAED
gi|4502167|ref|NP_000475.1| amyloid beta A4 protein precursor isoform a	memapsin-1	LVFFAEDV
gi|39753970|ref|NP_004336.2| cathelicidin antimicrobial peptide	cathepsin D	LGDFFRKS
gi|39753970|ref|NP_004336.2| cathelicidin antimicrobial peptide	cathepsin D	IKDFLRNL
gi|39753970|ref|NP_004336.2| cathelicidin antimicrobial peptide	cathepsin E	LGDFFRKS
gi|39753970|ref|NP_004336.2| cathelicidin antimicrobial peptide	cathepsin E	IKDFLRNL

gi|19923395|ref|NP_036237.2| beta-site APP-cleaving enzyme 2 isoform A preproprotein	memapsin-1	GLALALEP
gi|4503689|ref|NP_000499.1| fibrinogen alpha polypeptide isoform alpha-E preproprotein	cathepsin D	GNFKSQLQ
gi|4503689|ref|NP_000499.1| fibrinogen alpha polypeptide isoform alpha-E preproprotein	cathepsin D	WGTFEEVS
gi|4503689|ref|NP_000499.1| fibrinogen alpha polypeptide isoform alpha-E preproprotein	cathepsin D	LGEFVSET
gi|4503689|ref|NP_000499.1| fibrinogen alpha polypeptide isoform alpha-E preproprotein	cathepsin E	GNFKSQLQ
gi|4503689|ref|NP_000499.1| fibrinogen alpha polypeptide isoform alpha-E preproprotein	cathepsin E	WGTFEEVS
gi|4503689|ref|NP_000499.1| fibrinogen alpha polypeptide isoform alpha-E preproprotein	cathepsin E	LGEFVSET
gi|41406055|ref|NP_958816.1| amyloid beta A4 protein precursor isoform b	cathepsin D	VVIATVIV
gi|41406055|ref|NP_958816.1| amyloid beta A4 protein precursor isoform b	cathepsin D	IIGLMVGG
gi|41406055|ref|NP_958816.1| amyloid beta A4 protein precursor isoform b	cathepsin D	VITLVMLK
gi|41406055|ref|NP_958816.1| amyloid beta A4 protein precursor isoform b	cathepsin D	KLVFFAED
gi|41406055|ref|NP_958816.1| amyloid beta A4 protein precursor isoform b	cathepsin D	LVFFAEDV
gi|41406055|ref|NP_958816.1| amyloid beta A4 protein precursor isoform b	cathepsin D	TYKFFEQM
gi|41406055|ref|NP_958816.1| amyloid beta A4 protein precursor isoform b	cathepsin D	VIATVIVI
gi|41406055|ref|NP_958816.1| amyloid beta A4 protein precursor isoform b	cathepsin D	IVITLVML
gi|41406055|ref|NP_958816.1| amyloid beta A4 protein precursor isoform b	cathepsin E	VVIATVIV
gi|41406055|ref|NP_958816.1| amyloid beta A4 protein precursor isoform b	cathepsin E	IIGLMVGG
gi|41406055|ref|NP_958816.1| amyloid beta A4 protein precursor isoform b	cathepsin E	VITLVMLK
gi|41406055|ref|NP_958816.1| amyloid beta A4 protein precursor isoform b	cathepsin E	KLVFFAED
gi|41406055|ref|NP_958816.1| amyloid beta A4 protein precursor isoform b	cathepsin E	LVFFAEDV
gi|41406055|ref|NP_958816.1| amyloid beta A4 protein precursor isoform b	cathepsin E	TYKFFEQM
gi|41406055|ref|NP_958816.1| amyloid beta A4 protein precursor isoform b	cathepsin E	VIATVIVI
gi|41406055|ref|NP_958816.1| amyloid beta A4 protein precursor isoform b	cathepsin E	IVITLVML
gi|41406055|ref|NP_958816.1| amyloid beta A4 protein precursor isoform b	memapsin-2	EVKMDAEF

gi|41406055|ref|NP_958816.1| amyloid beta A4 protein precursor isoform b	memapsin-1	KLVFFAED
gi|41406055|ref|NP_958816.1| amyloid beta A4 protein precursor isoform b	memapsin-1	LVFFAEDV
gi|4506105|ref|NP_000939.1| prolactin	cathepsin D	GMELIVSQ
gi|4506105|ref|NP_000939.1| prolactin	cathepsin D	YPVWSGLP
gi|4506105|ref|NP_000939.1| prolactin	cathepsin D	NEIYPVWS
gi|4506105|ref|NP_000939.1| prolactin	cathepsin E	GMELIVSQ
gi|4506105|ref|NP_000939.1| prolactin	cathepsin E	YPVWSGLP
gi|4506105|ref|NP_000939.1| prolactin	cathepsin E	NEIYPVWS
gi|41406057|ref|NP_958817.1| amyloid beta A4 protein precursor isoform c	cathepsin D	VVIATVIV
gi|41406057|ref|NP_958817.1| amyloid beta A4 protein precursor isoform c	cathepsin D	IIGLMVGG
gi|41406057|ref|NP_958817.1| amyloid beta A4 protein precursor isoform c	cathepsin D	VITLVMLK
gi|41406057|ref|NP_958817.1| amyloid beta A4 protein precursor isoform c	cathepsin D	KLVFFAED
gi|41406057|ref|NP_958817.1| amyloid beta A4 protein precursor isoform c	cathepsin D	LVFFAEDV
gi|41406057|ref|NP_958817.1| amyloid beta A4 protein precursor isoform c	cathepsin D	TYKFFEQM
gi|41406057|ref|NP_958817.1| amyloid beta A4 protein precursor isoform c	cathepsin D	VIATVIVI
gi|41406057|ref|NP_958817.1| amyloid beta A4 protein precursor isoform c	cathepsin D	IVITLVML
gi|41406057|ref|NP_958817.1| amyloid beta A4 protein precursor isoform c	cathepsin E	VVIATVIV
gi|41406057|ref|NP_958817.1| amyloid beta A4 protein precursor isoform c	cathepsin E	IIGLMVGG
gi|41406057|ref|NP_958817.1| amyloid beta A4 protein precursor isoform c	cathepsin E	VITLVMLK
gi|41406057|ref|NP_958817.1| amyloid beta A4 protein precursor isoform c	cathepsin E	KLVFFAED
gi|41406057|ref|NP_958817.1| amyloid beta A4 protein precursor isoform c	cathepsin E	LVFFAEDV
gi|41406057|ref|NP_958817.1| amyloid beta A4 protein precursor isoform c	cathepsin E	TYKFFEQM
gi|41406057|ref|NP_958817.1| amyloid beta A4 protein precursor isoform c	cathepsin E	VIATVIVI
gi|41406057|ref|NP_958817.1| amyloid beta A4 protein precursor isoform c	cathepsin E	IVITLVML
gi|41406057|ref|NP_958817.1| amyloid beta A4 protein precursor isoform c	memapsin-2	EVKMDAEF
gi|41406057|ref|NP_958817.1| amyloid beta A4 protein precursor isoform c	memapsin-1	KLVFFAED
gi|41406057|ref|NP_958817.1| amyloid beta A4 protein precursor isoform c	memapsin-1	LVFFAEDV

gi|9055238|ref|NP_060870.1| voltage-gated sodium channel beta-3 subunit precursor	memapsin-2	EFEFEAHR
gi|9055238|ref|NP_060870.1| voltage-gated sodium channel beta-3 subunit precursor	memapsin-2	GEDFTSVV
gi|11761629|ref|NP_068657.1| fibrinogen alpha polypeptide isoform alpha preproprotein	cathepsin D	GNFKSQLQ
gi|11761629|ref|NP_068657.1| fibrinogen alpha polypeptide isoform alpha preproprotein	cathepsin D	WGTFEEVS
gi|11761629|ref|NP_068657.1| fibrinogen alpha polypeptide isoform alpha preproprotein	cathepsin D	LGEFVSET
gi|11761629|ref|NP_068657.1| fibrinogen alpha polypeptide isoform alpha preproprotein	cathepsin E	GNFKSQLQ
gi|11761629|ref|NP_068657.1| fibrinogen alpha polypeptide isoform alpha preproprotein	cathepsin E	WGTFEEVS
gi|11761629|ref|NP_068657.1| fibrinogen alpha polypeptide isoform alpha preproprotein	cathepsin E	LGEFVSET
gi|109148522|ref|NP_001035835.1| insulin- insulin-like growth factor 2	pepsin A	GSHLVEAL
gi|109148522|ref|NP_001035835.1| insulin- insulin-like growth factor 2	pepsin A	ERGFFYTP
gi|109148522|ref|NP_001035835.1| insulin- insulin-like growth factor 2	gastricsin	HLVEALYL
gi|109148522|ref|NP_001035835.1| insulin- insulin-like growth factor 2	gastricsin	LVEALYLV
gi|109148522|ref|NP_001035835.1| insulin- insulin-like growth factor 2	gastricsin	ERGFFYTP
gi|109148522|ref|NP_001035835.1| insulin- insulin-like growth factor 2	cathepsin D	ERGFFYTP
gi|109148522|ref|NP_001035835.1| insulin- insulin-like growth factor 2	cathepsin E	ERGFFYTP
gi|4504893|ref|NP_000884.1| kininogen 1 isoform 2	cathepsin D	ITKLNAEN
gi|4504893|ref|NP_000884.1| kininogen 1 isoform 2	cathepsin D	AGKKYFID
gi|4504893|ref|NP_000884.1| kininogen 1 isoform 2	cathepsin D	FIDFVARE
gi|4504893|ref|NP_000884.1| kininogen 1 isoform 2	cathepsin E	ITKLNAEN
gi|4504893|ref|NP_000884.1| kininogen 1 isoform 2	cathepsin E	AGKKYFID
gi|4504893|ref|NP_000884.1| kininogen 1 isoform 2	cathepsin E	FIDFVARE
gi|62243248|ref|NP_001013416.1| insulin-like growth factor binding protein 3 isoform a precursor	cathepsin D	RLRAYLLP
gi|62243248|ref|NP_001013416.1| insulin-like growth factor binding protein 3 isoform a precursor	cathepsin D	LKFLNVLS
gi|62243248|ref|NP_001013416.1| insulin-like growth factor binding protein 3 isoform a precursor	cathepsin D	SQRYKVDY
gi|62243248|ref|NP_001013416.1| insulin-like growth factor binding protein 3 isoform a precursor	cathepsin D	KVDYESQS
gi|62243248|ref|NP_001013416.1| insulin-like growth factor binding protein 3 isoform a precursor	cathepsin E	RLRAYLLP
gi|62243248|ref|NP_001013416.1| insulin-like growth factor binding protein 3 isoform a precursor	cathepsin E	LKFLNVLS

gi|62243248|ref|NP_001013416.1| insulin-like growth factor binding protein 3 isoform a precursor	cathepsin E	SQRYKVDY
gi|62243248|ref|NP_001013416.1| insulin-like growth factor binding protein 3 isoform a precursor	cathepsin E	KVDYESQS
gi|4503107|ref|NP_000090.1| cystatin C precursor	cathepsin D	TMTLSKST
gi|4503107|ref|NP_000090.1| cystatin C precursor	cathepsin D	NYFLDVEL
gi|4503107|ref|NP_000090.1| cystatin C precursor	cathepsin D	ALDFAVGE
gi|4503107|ref|NP_000090.1| cystatin C precursor	cathepsin D	FQIYAVPW
gi|4503107|ref|NP_000090.1| cystatin C precursor	cathepsin E	TMTLSKST
gi|4503107|ref|NP_000090.1| cystatin C precursor	cathepsin E	NYFLDVEL
gi|4503107|ref|NP_000090.1| cystatin C precursor	cathepsin E	ALDFAVGE
gi|4503107|ref|NP_000090.1| cystatin C precursor	cathepsin E	FQIYAVPW
gi|21040366|ref|NP_620428.1| beta-site APP-cleaving enzyme 1 isoform B preproprotein	memapsin-2	GTQHGIRL
gi|21040366|ref|NP_620428.1| beta-site APP-cleaving enzyme 1 isoform B preproprotein	memapsin-2	SSNFAVGA
gi|21040366|ref|NP_620428.1| beta-site APP-cleaving enzyme 1 isoform B preproprotein	memapsin-2	GLAYAEIA
gi|6715607|ref|NP_000175.1| G-gamma globin	cathepsin D	LGRLLVVY
gi|6715607|ref|NP_000175.1| G-gamma globin	cathepsin D	LGRLLVVY
gi|6715607|ref|NP_000175.1| G-gamma globin	cathepsin D	GRLLVVYP
gi|6715607|ref|NP_000175.1| G-gamma globin	cathepsin D	GRLLVVYP
gi|6715607|ref|NP_000175.1| G-gamma globin	cathepsin E	LGRLLVVY
gi|6715607|ref|NP_000175.1| G-gamma globin	cathepsin E	LGRLLVVY
gi|6715607|ref|NP_000175.1| G-gamma globin	cathepsin E	GRLLVVYP
gi|6715607|ref|NP_000175.1| G-gamma globin	cathepsin E	GRLLVVYP
gi|33943099|ref|NP_000533.2| surfactant pulmonary-associated protein B	napsin A	KLVLPVLP
gi|4557287|ref|NP_000020.1| angiotensinogen preproprotein	renin	PFHLVIHN
gi|4504349|ref|NP_000509.1| beta globin	cathepsin D	VQAAYQKV
gi|4504349|ref|NP_000509.1| beta globin	cathepsin D	VTALWGKV
gi|4504349|ref|NP_000509.1| beta globin	cathepsin D	LGRLLVVY
gi|4504349|ref|NP_000509.1| beta globin	cathepsin D	LGRLLVVY
gi|4504349|ref|NP_000509.1| beta globin	cathepsin D	GRLLVVYP
gi|4504349|ref|NP_000509.1| beta globin	cathepsin D	GRLLVVYP
gi|4504349|ref|NP_000509.1| beta globin	cathepsin D	TQRFFESF
gi|4504349|ref|NP_000509.1| beta globin	cathepsin D	TQRFFESF
gi|4504349|ref|NP_000509.1| beta globin	cathepsin D	FESFGDLS
gi|4504349|ref|NP_000509.1| beta globin	cathepsin D	FESFGDLS
gi|4504349|ref|NP_000509.1| beta globin	cathepsin D	KGTFATLS
gi|4504349|ref|NP_000509.1| beta globin	cathepsin D	TALWGKVN
gi|4504349|ref|NP_000509.1| beta globin	cathepsin E	VQAAYQKV
gi|4504349|ref|NP_000509.1| beta globin	cathepsin E	VTALWGKV
gi|4504349|ref|NP_000509.1| beta globin	cathepsin E	LGRLLVVY
gi|4504349|ref|NP_000509.1| beta globin	cathepsin E	LGRLLVVY
gi|4504349|ref|NP_000509.1| beta globin	cathepsin E	GRLLVVYP
gi|4504349|ref|NP_000509.1| beta globin	cathepsin E	GRLLVVYP
gi|4504349|ref|NP_000509.1| beta globin	cathepsin E	TQRFFESF

gi|4504349|ref|NP_000509.1| beta globin	cathepsin E	TQRFFESF
gi|4504349|ref|NP_000509.1| beta globin	cathepsin E	FESFGDLS
gi|4504349|ref|NP_000509.1| beta globin	cathepsin E	FESFGDLS
gi|4504349|ref|NP_000509.1| beta globin	cathepsin E	KGTFATLS
gi|4504349|ref|NP_000509.1| beta globin	cathepsin E	TALWGKVN
gi|21040364|ref|NP_620427.1| beta-site APP-cleaving enzyme 1 isoform C preproprotein	memapsin-2	GTQHGIRL
gi|21040364|ref|NP_620427.1| beta-site APP-cleaving enzyme 1 isoform C preproprotein	memapsin-2	SSNFAVGA
gi|148536853|ref|NP_004362.2| coatomer protein complex subunit alpha isoform 2	pepsin A	PWILTSLH
gi|21040362|ref|NP_620477.1| beta-site APP-cleaving enzyme 2 isoform B preproprotein	memapsin-1	GLALALEP
gi|148536855|ref|NP_001091868.1| coatomer protein complex subunit alpha isoform 1	pepsin A	PWILTSLH
gi|38504667|ref|NP_942140.1| surfactant pulmonary-associated protein B	napsin A	KLVLPVLP
gi|66932947|ref|NP_000005.2| alpha-2-macroglobulin precursor	chymosin	RVGFYESD
gi|66932947|ref|NP_000005.2| alpha-2-macroglobulin precursor	chymosin	VRKYFPET
gi|66932947|ref|NP_000005.2| alpha-2-macroglobulin precursor	chymosin	TETVRKYF
gi|16751921|ref|NP_444513.1| dermcidin preproprotein	cathepsin D	KDVLDSVL
gi|16751921|ref|NP_444513.1| dermcidin preproprotein	cathepsin D	VEDLESVG
gi|16751921|ref|NP_444513.1| dermcidin preproprotein	cathepsin E	KDVLDSVL
gi|16751921|ref|NP_444513.1| dermcidin preproprotein	cathepsin E	VEDLESVG
gi|4505881|ref|NP_000292.1| plasminogen	cathepsin D	LLKEAQLP
gi|4505881|ref|NP_000292.1| plasminogen	cathepsin D	VVLLPDVE
gi|4505881|ref|NP_000292.1| plasminogen	cathepsin D	DVVLFEKK
gi|4505881|ref|NP_000292.1| plasminogen	cathepsin E	LLKEAQLP
gi|4505881|ref|NP_000292.1| plasminogen	cathepsin E	VVLLPDVE
gi|4505881|ref|NP_000292.1| plasminogen	cathepsin E	DVVLFEKK
gi|62243068|ref|NP_000589.2| insulin-like growth factor binding protein 3 isoform b precursor	cathepsin D	RLRAYLLP
gi|62243068|ref|NP_000589.2| insulin-like growth factor binding protein 3 isoform b precursor	cathepsin D	LKFLNVLS
gi|62243068|ref|NP_000589.2| insulin-like growth factor binding protein 3 isoform b precursor	cathepsin D	SQRYKVDY
gi|62243068|ref|NP_000589.2| insulin-like growth factor binding protein 3 isoform b precursor	cathepsin D	KVDYESQS
gi|62243068|ref|NP_000589.2| insulin-like growth factor binding protein 3 isoform b precursor	cathepsin E	RLRAYLLP
gi|62243068|ref|NP_000589.2| insulin-like growth factor binding protein 3 isoform b precursor	cathepsin E	LKFLNVLS
gi|62243068|ref|NP_000589.2| insulin-like growth factor binding protein 3 isoform b precursor	cathepsin E	SQRYKVDY
gi|62243068|ref|NP_000589.2| insulin-like growth factor binding protein 3 isoform b precursor	cathepsin E	KVDYESQS

gi|50659080|ref|NP_001076.2| serpin peptidase inhibitor clade A member 3 precursor	cathepsin D	LLSALVET
gi|50659080|ref|NP_001076.2| serpin peptidase inhibitor clade A member 3 precursor	cathepsin D	ITLLSALV
gi|50659080|ref|NP_001076.2| serpin peptidase inhibitor clade A member 3 precursor	cathepsin D	LSALVETR
gi|50659080|ref|NP_001076.2| serpin peptidase inhibitor clade A member 3 precursor	cathepsin E	LLSALVET
gi|50659080|ref|NP_001076.2| serpin peptidase inhibitor clade A member 3 precursor	cathepsin E	ITLLSALV
gi|50659080|ref|NP_001076.2| serpin peptidase inhibitor clade A member 3 precursor	cathepsin E	LSALVETR
gi|4885215|ref|NP_005226.1| v-erb-a erythroblastic leukemia viral oncogene homolog 4 isoform JM-a/CVT-1 precursor	presenilin 1	LTFAVYVR
gi|48255935|ref|NP_000601.3| CD44 antigen isoform 1 precursor	presenilin 1	SLLALALI
gi|110825958|ref|NP_001036064.1| v-erb-a erythroblastic leukemia viral oncogene homolog 4 isoform JM-a/CVT-2 precursor	presenilin 1	LTFAVYVR
gi|187960059|ref|NP_055469.3| syndecan 3	presenilin 1	AAFLVTLL
gi|187960059|ref|NP_055469.3| syndecan 3	presenilin 1	FLVTLLIY
gi|187960059|ref|NP_055469.3| syndecan 3	presenilin 1	AFLVTLLI
gi|48255937|ref|NP_001001389.1| CD44 antigen isoform 2 precursor	presenilin 1	SLLALALI
gi|4757960|ref|NP_004351.1| cadherin 1 type 1 preproprotein	presenilin 1	LLFLRRRA
gi|4502167|ref|NP_000475.1| amyloid beta A4 protein precursor isoform a	presenilin 1	VVIATVIV
gi|4502167|ref|NP_000475.1| amyloid beta A4 protein precursor isoform a	presenilin 1	GGVVIATV
gi|41406055|ref|NP_958816.1| amyloid beta A4 protein precursor isoform b	presenilin 1	VVIATVIV
gi|41406055|ref|NP_958816.1| amyloid beta A4 protein precursor isoform b	presenilin 1	GGVVIATV
gi|41406057|ref|NP_958817.1| amyloid beta A4 protein precursor isoform c	presenilin 1	VVIATVIV
gi|41406057|ref|NP_958817.1| amyloid beta A4 protein precursor isoform c	presenilin 1	GGVVIATV
gi|148833508|ref|NP_060087.3| notch1 preproprotein	presenilin 1	YVAAAAFV
gi|148833508|ref|NP_060087.3| notch1 preproprotein	presenilin 1	VGCGVLLS
gi|148833508|ref|NP_060087.3| notch1 preproprotein	presenilin 1	VGCGVLLS
gi|48255939|ref|NP_001001390.1| CD44 antigen isoform 3 precursor	presenilin 1	SLLALALI
gi|55770876|ref|NP_004548.3| notch4 preproprotein	presenilin 1	GALLVLQL
gi|48255941|ref|NP_001001391.1| CD44 antigen isoform 4 precursor	presenilin 1	SLLALALI

Cysteine Proteases

gi|48762934|ref|NP_000080.2| alpha 2 type I collagen	cathepsin K	AGARGSDG
gi|48762934|ref|NP_000080.2| alpha 2 type I collagen	cathepsin K	GLPGFKGI
gi|40316933|ref|NP_954642.1| osteocalcin preproprotein	cathepsin S	QWLGAPVP
gi|40316933|ref|NP_954642.1| osteocalcin preproprotein	cathepsin H	QWLGAPVP
gi|4507065|ref|NP_003055.1| secretory leukocyte peptidase inhibitor precursor	cathepsin S	CPVTYGQC
gi|23110962|ref|NP_004070.3| cathepsin S preproprotein	cathepsin S	EEVMSLMS
gi|23110962|ref|NP_004070.3| cathepsin S preproprotein	cathepsin S	SLMSSLRV
gi|4503151|ref|NP_000387.1| cathepsin K preproprotein	cathepsin K	YIPEWEGR
gi|4503151|ref|NP_000387.1| cathepsin K preproprotein	cathepsin K	LYPEEILD
gi|4503151|ref|NP_000387.1| cathepsin K preproprotein	cathepsin K	VPLSHSRS
gi|189083844|ref|NP_001805.3| cathepsin C isoform a preproprotein	dipeptidyl-peptidase I	NSQEKYSN
gi|189083844|ref|NP_001805.3| cathepsin C isoform a preproprotein	dipeptidyl-peptidase I	LTAEIQQK
gi|189083844|ref|NP_001805.3| cathepsin C isoform a preproprotein	dipeptidyl-peptidase I	LKNSQEKY
gi|110349772|ref|NP_000079.2| alpha 1 type I collagen preproprotein	cathepsin K	EGPQGVRG
gi|110349772|ref|NP_000079.2| alpha 1 type I collagen preproprotein	cathepsin K	MGPSGPRG
gi|110349772|ref|NP_000079.2| alpha 1 type I collagen preproprotein	cathepsin K	QPPQEKAH
gi|110349772|ref|NP_000079.2| alpha 1 type I collagen preproprotein	cathepsin K	DFSFLPQP
gi|110349772|ref|NP_000079.2| alpha 1 type I collagen preproprotein	cathepsin K	GLPGMKGH
gi|33943099|ref|NP_000533.2| surfactant pulmonary-associated protein B	cathepsin H	ALQARPGP
gi|33943099|ref|NP_000533.2| surfactant pulmonary-associated protein B	cathepsin H	SEQAIPQA
gi|33943099|ref|NP_000533.2| surfactant pulmonary-associated protein B	cathepsin H	QCIHSPDL
gi|33943099|ref|NP_000533.2| surfactant pulmonary-associated protein B	cathepsin H	LLTLVPRG
gi|33943099|ref|NP_000533.2| surfactant pulmonary-associated protein B	cathepsin H	RCSMDDSA
gi|38504667|ref|NP_942140.1| surfactant pulmonary-associated protein B	cathepsin H	ALQARPGP
gi|38504667|ref|NP_942140.1| surfactant pulmonary-associated protein B	cathepsin H	SEQAIPQA
gi|38504667|ref|NP_942140.1| surfactant pulmonary-associated protein B	cathepsin H	QCIHSPDL
gi|38504667|ref|NP_942140.1| surfactant pulmonary-associated protein B	cathepsin H	LLTLVPRG

gi|38504667|ref|NP_942140.1| surfactant pulmonary-associated protein B	cathepsin H	RCSMDDSA
gi|5453816|ref|NP_006174.1| neurotensin/neuromedin N preproprotein	legumain	LYENKPRR
gi|56682962|ref|NP_005597.3| legumain preproprotein	legumain	MNTNDLEE
gi|68509940|ref|NP_001020272.1| Golli-mbp isoform 1	legumain	FFKNIVTP
gi|56682964|ref|NP_001008530.1| legumain preproprotein	legumain	MNTNDLEE
gi|68509928|ref|NP_001020261.1| myelin basic protein isoform 3	legumain	FFKNIVTP
gi|4505123|ref|NP_002376.1| myelin basic protein isoform 2	legumain	FFKNIVTP
gi|68509930|ref|NP_001020252.1| myelin basic protein isoform 1	legumain	FFKNIVTP
gi|68509932|ref|NP_001020263.1| myelin basic protein isoform 4	legumain	FFKNIVTP
gi|15431310|ref|NP_000517.2| keratin 14	caspase-6	VEMDAAPG
gi|15431310|ref|NP_000517.2| keratin 14	caspase-6	VEMDAAPG
gi|6552305|ref|NP_009228.1| breast cancer 1 early onset isoform BRCA1-delta2-10	caspase-3	DLLDDGEI
gi|6552305|ref|NP_009228.1| breast cancer 1 early onset isoform BRCA1-delta2-10	caspase-3	DVLDVLNE
gi|88953883|ref|XP_933794.1| PREDICTED: similar to heterogeneous nuclear ribonucleoprotein A1	caspase-3	SYNDFGNY
gi|14043070|ref|NP_112420.1| heterogeneous nuclear ribonucleoprotein A1 isoform b	caspase-3	GSYDSYNN
gi|14043070|ref|NP_112420.1| heterogeneous nuclear ribonucleoprotein A1 isoform b	caspase-3	SYNDFGNY
gi|109637757|ref|NP_775086.1| calpastatin isoform e	caspase-1	ALDDLIDT
gi|109637757|ref|NP_775086.1| calpastatin isoform e	caspase-1	LSSDFTCG
gi|109637757|ref|NP_775086.1| calpastatin isoform e	caspase-1	ALADSLGK
gi|109637757|ref|NP_775086.1| calpastatin isoform e	caspase-3	LSSDFTCG
gi|189083774|ref|NP_001121135.1| gelsolin isoform b	caspase-3	DQTDGLGL
gi|29568103|ref|NP_003080.2| U1 small nuclear ribonucleoprotein 70 kDa	caspase-3	DGPDGPEE
gi|15431330|ref|NP_150635.1| caspase 1 isoform gamma precursor	caspase-1	AVQDNPAM
gi|15431330|ref|NP_150635.1| caspase 1 isoform gamma precursor	caspase-1	WFKDSVGV
gi|15431330|ref|NP_150635.1| caspase 1 isoform gamma precursor	caspase-1	FEDDAIKK
gi|114520609|ref|NP_006145.2| neural precursor cell expressed developmentally down-regulated 4 isoform 1	caspase-3	GQVDVPLY
gi|4758128|ref|NP_004725.1| doublecortin and CaM kinase-like 1	caspase-3	DENDGPGE
gi|4758128|ref|NP_004725.1| doublecortin and CaM kinase-like 1	caspase-3	DENDGPGE
gi|4758128|ref|NP_004725.1| doublecortin and CaM kinase-like 1	caspase-8	DENDGPGE

gi|30410794|ref|NP_005780.2| proteasome activator subunit 3 isoform 1	caspase-3	DGLDGPTY
gi|24430146|ref|NP_005115.2| nucleoporin 153kDa	caspase-3	DITDFQAK
gi|14670388|ref|NP_116784.1| BCL2-associated agonist of cell death	caspase-3	EQEDSSSA
gi|32261318|ref|NP_734465.2| unc-5 homolog B	caspase-3	DITDSSAA
gi|32261318|ref|NP_734465.2| unc-5 homolog B	caspase-3	DITDSSAA
gi|32528306|ref|NP_002904.3| replication factor C large subunit	caspase-3	IETDAMIK
gi|32528306|ref|NP_002904.3| replication factor C large subunit	caspase-3	DEVDGMAG
gi|32528306|ref|NP_002904.3| replication factor C large subunit	caspase-3	DLVDSQIR
gi|41322912|ref|NP_958780.1| plectin 1 isoform 2	caspase-8	ILRDKDNT
gi|169160761|ref|XP_001713794.1| PREDICTED: hypothetical protein isoform 1	caspase-3	YVPDSPAL
gi|169160761|ref|XP_001713794.1| PREDICTED: hypothetical protein isoform 1	caspase-1	YVPDSPAL
gi|189083780|ref|NP_001121138.1| gelsolin isoform c	caspase-3	DQTDGLGL
gi|169160765|ref|XP_001713796.1| PREDICTED: hypothetical protein isoform 3	caspase-3	YVPDSPAL
gi|169160765|ref|XP_001713796.1| PREDICTED: hypothetical protein isoform 3	caspase-1	YVPDSPAL
gi|10862703|ref|NP_066124.1| ret proto-oncogene isoform a	caspase-3	DYLDLAAS
gi|10862703|ref|NP_066124.1| ret proto-oncogene isoform a	caspase-3	VSVDAFKI
gi|169204588|ref|XP_001723453.1| PREDICTED: similar to prothymosin alpha	caspase-3	DDEDDDVD
gi|169204588|ref|XP_001723453.1| PREDICTED: similar to prothymosin alpha	caspase-3	DEDDDVDT
gi|4503823|ref|NP_002028.1| protein-tyrosine kinase fyn isoform a	caspase-3	EERDGSLN
gi|4501881|ref|NP_001091.1| actin alpha 1 skeletal muscle	caspase-1	ELPDGQVI
gi|4501881|ref|NP_001091.1| actin alpha 1 skeletal muscle	caspase-3	ELPDGQVI
gi|169160767|ref|XP_001713797.1| PREDICTED: hypothetical protein isoform 4	caspase-3	YVPDSPAL
gi|169160767|ref|XP_001713797.1| PREDICTED: hypothetical protein isoform 4	caspase-1	YVPDSPAL
gi|4757838|ref|NP_004315.1| BCL2-associated X protein isoform beta	caspase-3	FIQDRAGR
gi|4507441|ref|NP_003211.1| transcription factor AP-2 alpha isoform a	caspase-1	DRHDGTSN
gi|4507441|ref|NP_003211.1| transcription factor AP-2 alpha isoform a	caspase-3	DRHDGTSN
gi|4507441|ref|NP_003211.1| transcription factor AP-2 alpha isoform a	caspase-6	DRHDGTSN

gi|169160769|ref|XP_001713798.1| PREDICTED: hypothetical protein isoform 5	caspase-3	YVPDSPAL
gi|169160769|ref|XP_001713798.1| PREDICTED: hypothetical protein isoform 5	caspase-1	YVPDSPAL
gi|109637759|ref|NP_001035905.1| calpastatin isoform f	caspase-1	ALDDLIDT
gi|109637759|ref|NP_001035905.1| calpastatin isoform f	caspase-1	LSSDFTCG
gi|109637759|ref|NP_001035905.1| calpastatin isoform f	caspase-1	ALADSLGK
gi|109637759|ref|NP_001035905.1| calpastatin isoform f	caspase-3	LSSDFTCG
gi|169160771|ref|XP_001713799.1| PREDICTED: hypothetical protein isoform 6	caspase-3	YVPDSPAL
gi|169160771|ref|XP_001713799.1| PREDICTED: hypothetical protein isoform 6	caspase-1	YVPDSPAL
gi|113430845|ref|XP_001129414.1| PREDICTED: similar to DNA dependent protein kinase catalytic subunit	caspase-3	DEVDNKVK
gi|6552303|ref|NP_009227.1| breast cancer 1 early onset isoform 1	caspase-3	DLLDDGEI
gi|6552303|ref|NP_009227.1| breast cancer 1 early onset isoform 1	caspase-3	DVLDVLNE
gi|53759122|ref|NP_000029.2| adenomatous polyposis coli	caspase-3	DNIDNLSP
gi|21536301|ref|NP_644671.1| signal transducer and activator of transcription 1 isoform beta	caspase-3	MELDGPKG
gi|58761496|ref|NP_001011724.1| heterogeneous nuclear ribonucleoprotein A1-like	caspase-3	SYNDFGNY
gi|32483359|ref|NP_863651.1| apoptotic peptidase activating factor 1 isoform c	caspase-3	SVTDSVMG
gi|21322252|ref|NP_000035.2| androgen receptor isoform 1	caspase-3	DEDDSAAP
gi|10862701|ref|NP_065681.1| ret proto-oncogene isoform c	caspase-3	DYLDLAAS
gi|10862701|ref|NP_065681.1| ret proto-oncogene isoform c	caspase-3	VSVDAFKI
gi|189083782|ref|NP_001121139.1| gelsolin isoform c	caspase-3	DQTDGLGL
gi|109637761|ref|NP_001035906.1| calpastatin isoform g	caspase-1	ALDDLIDT
gi|109637761|ref|NP_001035906.1| calpastatin isoform g	caspase-1	LSSDFTCG
gi|109637761|ref|NP_001035906.1| calpastatin isoform g	caspase-1	ALADSLGK
gi|109637761|ref|NP_001035906.1| calpastatin isoform g	caspase-3	LSSDFTCG
gi|6552311|ref|NP_009231.1| breast cancer 1 early onset isoform BRCA1-delta14-18	caspase-3	DLLDDGEI
gi|6552311|ref|NP_009231.1| breast cancer 1 early onset isoform BRCA1-delta14-18	caspase-3	DVLDVLNE
gi|154759259|ref|NP_003118.2| spectrin alpha non-erythrocytic 1 (alpha-fodrin) isoform 2	caspase-3	DETDSKTA
gi|154759259|ref|NP_003118.2| spectrin alpha non-erythrocytic 1 (alpha-fodrin) isoform 2	caspase-3	DSLDSVEA
gi|154759259|ref|NP_003118.2| spectrin alpha non-erythrocytic 1 (alpha-fodrin) isoform 2	caspase-1	DETDSKTA
gi|154759259|ref|NP_003118.2| spectrin alpha non-erythrocytic 1 (alpha-fodrin) isoform 2	caspase-4	DETDSKTA

gi|41322908|ref|NP_958781.1| plectin 1 isoform 3	caspase-8	ILRDKDNT
gi|83641885|ref|NP_001032726.1| basic transcription factor 3 isoform A	caspase-3	QSVDGKAP
gi|11545910|ref|NP_071444.1| livin inhibitor of apoptosis isoform beta	caspase-3	DHVDGQIL
gi|11545910|ref|NP_071444.1| livin inhibitor of apoptosis isoform beta	caspase-7	DHVDGQIL
gi|73760407|ref|NP_001027451.1| transcription factor AP-2 alpha isoform b	caspase-1	DRHDGTSN
gi|73760407|ref|NP_001027451.1| transcription factor AP-2 alpha isoform b	caspase-3	DRHDGTSN
gi|73760407|ref|NP_001027451.1| transcription factor AP-2 alpha isoform b	caspase-6	DRHDGTSN
gi|11225260|ref|NP_003277.1| DNA topoisomerase I	caspase-3	DDADYKPK
gi|11225260|ref|NP_003277.1| DNA topoisomerase I	caspase-3	EEEDGKLK
gi|11225260|ref|NP_003277.1| DNA topoisomerase I	caspase-6	PEDDGYFV
gi|11225260|ref|NP_003277.1| DNA topoisomerase I	caspase-6	EEEDGKLK
gi|189011566|ref|NP_001120982.1| adenomatous polyposis coli	caspase-3	DNIDNLSP
gi|169216975|ref|XP_001714644.1| PREDICTED: hypothetical protein	caspase-3	DFPDWWQV
gi|109637763|ref|NP_001035907.1| calpastatin isoform h	caspase-1	ALDDLIDT
gi|109637763|ref|NP_001035907.1| calpastatin isoform h	caspase-1	LSSDFTCG
gi|109637763|ref|NP_001035907.1| calpastatin isoform h	caspase-1	ALADSLGK
gi|109637763|ref|NP_001035907.1| calpastatin isoform h	caspase-3	LSSDFTCG
gi|38505165|ref|NP_942126.1| ras-related C3 botulinum toxin substrate 1 isoform Rac1c	caspase-3	DLRDDKDT
gi|6274552|ref|NP_009330.1| signal transducer and activator of transcription 1 isoform alpha	caspase-3	MELDGPKG
gi|7108333|ref|NP_037361.1| apoptotic peptidase activating factor 1 isoform a	caspase-3	SVTDSVMG
gi|189011568|ref|NP_001120983.1| adenomatous polyposis coli	caspase-3	DNIDNLSP
gi|169217548|ref|XP_001722951.1| PREDICTED: hypothetical protein	caspase-3	DDVDTKKQ
gi|169217548|ref|XP_001722951.1| PREDICTED: hypothetical protein	caspase-3	DDEDDDVD
gi|169217548|ref|XP_001722951.1| PREDICTED: hypothetical protein	caspase-3	DEDDDVDT
gi|109637765|ref|NP_001035908.1| calpastatin isoform i	caspase-1	ALDDLIDT
gi|109637765|ref|NP_001035908.1| calpastatin isoform i	caspase-1	LSSDFTCG
gi|109637765|ref|NP_001035908.1| calpastatin isoform i	caspase-1	ALADSLGK
gi|109637765|ref|NP_001035908.1| calpastatin isoform i	caspase-3	LSSDFTCG
gi|126032350|ref|NP_001075109.1| protein kinase DNA-activated catalytic polypeptide isoform 2	caspase-3	DEVDNKVK
gi|109389358|ref|NP_001035890.1| transcription factor AP-2 alpha isoform c	caspase-1	DRHDGTSN
gi|109389358|ref|NP_001035890.1| transcription factor AP-2 alpha isoform c	caspase-3	DRHDGTSN

gi|109389358|ref|NP_001035890.1| transcription factor AP-2 alpha isoform c	caspase-6	DRHDGTSN
gi|169217905|ref|XP_001717440.1| PREDICTED: similar to PML	caspase-3	PHLDGPPS
gi|6912480|ref|NP_036449.1| leucine zipper down-regulated in cancer 1	caspase-3	DEDDDDEE
gi|44662836|ref|NP_055382.2| breast cancer anti-estrogen resistance 1	caspase-3	DVPDGPLL
gi|44662836|ref|NP_055382.2| breast cancer anti-estrogen resistance 1	caspase-3	DSPDGQYE
gi|41322916|ref|NP_958782.1| plectin 1 isoform 6	caspase-8	ILRDKDNT
gi|157738639|ref|NP_116254.4| tensin 4	caspase-3	DSTDSPAS
gi|115583670|ref|NP_003244.2| T-cell lymphoma invasion and metastasis 1	caspase-3	DETDHSSK
gi|12408647|ref|NP_071801.1| X-ray repair cross complementing protein 4 isoform 2	caspase-3	DVTDIAPS
gi|169209154|ref|XP_001715722.1| PREDICTED: similar to hCG32587	caspase-3	DAGDVGAA
gi|148763345|ref|NP_277071.2| cell division cycle 2-like 2 isoform 4	caspase-3	YVPDSPAL
gi|148763345|ref|NP_277071.2| cell division cycle 2-like 2 isoform 4	caspase-1	YVPDSPAL
gi|109637767|ref|NP_001035909.1| calpastatin isoform j	caspase-1	ALDDLIDT
gi|109637767|ref|NP_001035909.1| calpastatin isoform j	caspase-1	LSSDFTCG
gi|109637767|ref|NP_001035909.1| calpastatin isoform j	caspase-1	ALADSLGK
gi|109637767|ref|NP_001035909.1| calpastatin isoform j	caspase-3	LSSDFTCG
gi|4507945|ref|NP_003392.1| X-ray repair cross complementing protein 4 isoform 1	caspase-3	DVTDIAPS
gi|32171186|ref|NP_005736.3| B-cell receptor-associated protein 31	caspase-8	AAVDGPMD
gi|12408649|ref|NP_072044.1| X-ray repair cross complementing protein 4 isoform 1	caspase-3	DVTDIAPS
gi|6005810|ref|NP_009112.1| mitogen-activated protein kinase kinase kinase kinase 1 isoform 2	caspase-3	DDVDIPTP
gi|157739864|ref|NP_001099032.1| ubiquitination factor E4B isoform 1	caspase-3	MDIDGVSC
gi|157739864|ref|NP_001099032.1| ubiquitination factor E4B isoform 1	caspase-7	MDIDGVSC
gi|157739864|ref|NP_001099032.1| ubiquitination factor E4B isoform 1	caspase-6	VDVDSGIE
gi|83281438|ref|NP_003749.2| eukaryotic translation initiation factor 3 subunit 1 alpha 35kDa	caspase-3	DLADYGGY
gi|110611908|ref|NP_001036065.1| mitogen-activated protein kinase kinase kinase kinase 1 isoform 1	caspase-3	DDVDIPTP
gi|4506401|ref|NP_002871.1| v-raf-1 murine leukemia viral oncogene homolog 1	caspase-9	MIEDAIRS
gi|23943920|ref|NP_077734.1| cytosolic phospholipase A2 group IVA	caspase-3	DELDAAVA

gi|4502147|ref|NP_001633.1| amyloid beta (A4) precursor-like protein 2	caspase-3	VEVDPMLT
gi|196162702|ref|NP_001124494.1| X-ray repair cross complementing protein 4 isoform 3	caspase-3	DVTDIAPS
gi|20631973|ref|NP_620120.1| BCL2-associated X protein isoform sigma	caspase-3	FIQDRAGR
gi|109637769|ref|NP_001035910.1| calpastatin isoform k	caspase-1	ALDDLIDT
gi|109637769|ref|NP_001035910.1| calpastatin isoform k	caspase-1	LSSDFTCG
gi|109637769|ref|NP_001035910.1| calpastatin isoform k	caspase-1	ALADSLGK
gi|109637769|ref|NP_001035910.1| calpastatin isoform k	caspase-3	LSSDFTCG
gi|88958985|ref|XP_941883.1| PREDICTED: similar to heterogeneous nuclear ribonucleoprotein A1	caspase-3	SYNDFGNY
gi|7108367|ref|NP_005419.2| vav 1 guanine nucleotide exchange factor	caspase-3	DQIDDTVE
gi|7108367|ref|NP_005419.2| vav 1 guanine nucleotide exchange factor	caspase-3	DLYDCVEN
gi|151101404|ref|NP_002814.3| prothymosin alpha isoform 2	caspase-3	DDVDTKKQ
gi|151101404|ref|NP_002814.3| prothymosin alpha isoform 2	caspase-3	DDEDDDVD
gi|151101404|ref|NP_002814.3| prothymosin alpha isoform 2	caspase-3	DEDDDVDT
gi|6552309|ref|NP_009230.1| breast cancer 1 early onset isoform BRCA1-delta14-17	caspase-3	DLLDDGEI
gi|6552309|ref|NP_009230.1| breast cancer 1 early onset isoform BRCA1-delta14-17	caspase-3	DVLDVLNE
gi|55750041|ref|NP_001931.2| atrophin-1	caspase-3	DSLDGRSL
gi|55750041|ref|NP_001931.2| atrophin-1	caspase-7	DSLDGRSL
gi|55750041|ref|NP_001931.2| atrophin-1	caspase-2	DSLDGRSL
gi|55750041|ref|NP_001931.2| atrophin-1	caspase-8	DSLDGRSL
gi|109637771|ref|NP_001035911.1| calpastatin isoform l	caspase-1	ALDDLIDT
gi|109637771|ref|NP_001035911.1| calpastatin isoform l	caspase-1	LSSDFTCG
gi|109637771|ref|NP_001035911.1| calpastatin isoform l	caspase-1	ALADSLGK
gi|109637771|ref|NP_001035911.1| calpastatin isoform l	caspase-3	LSSDFTCG
gi|151101407|ref|NP_001092755.1| prothymosin alpha isoform 1	caspase-3	DDVDTKKQ
gi|151101407|ref|NP_001092755.1| prothymosin alpha isoform 1	caspase-3	DDEDDDVD
gi|151101407|ref|NP_001092755.1| prothymosin alpha isoform 1	caspase-3	DEDDDVDT
gi|38327034|ref|NP_006039.2| ubiquitination factor E4B isoform 2	caspase-3	MDIDGVSC
gi|38327034|ref|NP_006039.2| ubiquitination factor E4B isoform 2	caspase-7	MDIDGVSC
gi|38327034|ref|NP_006039.2| ubiquitination factor E4B isoform 2	caspase-6	VDVDSGIE
gi|4501889|ref|NP_001606.1| actin gamma 2 propeptide	caspase-1	ELPDGQVI

gi|4501889|ref|NP_001606.1| actin gamma 2 propeptide	caspase-3	ELPDGQVI
gi|32483361|ref|NP_863658.1| apoptotic peptidase activating factor 1 isoform d	caspase-3	SVTDSVMG
gi|41322919|ref|NP_958784.1| plectin 1 isoform 8	caspase-8	ILRDKDNT
gi|22027653|ref|NP_663782.1| adaptor-related protein complex 1 beta 1 subunit isoform b	caspase-3	DLFDLTSG
gi|19923215|ref|NP_002388.2| myocyte enhancer factor 2C isoform 1	caspase-3	SSYDGSDR
gi|19923215|ref|NP_002388.2| myocyte enhancer factor 2C isoform 1	caspase-7	SSYDGSDR
gi|19923215|ref|NP_002388.2| myocyte enhancer factor 2C isoform 1	caspase-3	SSYDGSDR
gi|19923215|ref|NP_002388.2| myocyte enhancer factor 2C isoform 1	caspase-7	SSYDGSDR
gi|196114945|ref|NP_001124477.1| myocyte enhancer factor 2C isoform 2	caspase-3	SSYDGSDR
gi|196114945|ref|NP_001124477.1| myocyte enhancer factor 2C isoform 2	caspase-7	SSYDGSDR
gi|196114945|ref|NP_001124477.1| myocyte enhancer factor 2C isoform 2	caspase-3	SSYDGSDR
gi|196114945|ref|NP_001124477.1| myocyte enhancer factor 2C isoform 2	caspase-7	SSYDGSDR
gi|32483363|ref|NP_863659.1| apoptotic peptidase activating factor 1 isoform e	caspase-3	SVTDSVMG
gi|89036005|ref|XP_950171.1| PREDICTED: similar to prothymosin alpha isoform 2	caspase-3	DDEDDDVD
gi|89036005|ref|XP_950171.1| PREDICTED: similar to prothymosin alpha isoform 2	caspase-3	DEDDDVDT
gi|41322914|ref|NP_958785.1| plectin 1 isoform 10	caspase-8	ILRDKDNT
gi|7305199|ref|NP_038462.1| Kv channel interacting protein 3 isoform 1	caspase-3	DSSDSELE
gi|4503131|ref|NP_001895.1| catenin (cadherin-associated protein) beta 1 88kDa	caspase-3	TQFDAAHP
gi|4503131|ref|NP_001895.1| catenin (cadherin-associated protein) beta 1 88kDa	caspase-3	SYLDSGIH
gi|4503131|ref|NP_001895.1| catenin (cadherin-associated protein) beta 1 88kDa	caspase-3	YPVDGLPD
gi|4503131|ref|NP_001895.1| catenin (cadherin-associated protein) beta 1 88kDa	caspase-3	DLMDGLPP
gi|4503131|ref|NP_001895.1| catenin (cadherin-associated protein) beta 1 88kDa	caspase-3	ADIDGQYA
gi|71902540|ref|NP_000042.3| ataxia telangiectasia mutated protein isoform 1	caspase-3	DYPDSSVS
gi|9845509|ref|NP_061485.1| ras-related C3 botulinum toxin substrate 1 isoform Rac1b	caspase-3	DLRDDKDT
gi|5902076|ref|NP_008855.1| splicing factor arginine/serine-rich 1 isoform 1	caspase-3	DLKDHMRE

gi|5902076|ref|NP_008855.1| splicing factor arginine/serine-rich 1 isoform 1	caspase-3	CYADVYRD
gi|5902076|ref|NP_008855.1| splicing factor arginine/serine-rich 1 isoform 1	caspase-3	VYRDGTGV
gi|5902076|ref|NP_008855.1| splicing factor arginine/serine-rich 1 isoform 1	caspase-3	RKLDNTKF
gi|4757952|ref|NP_001782.1| cell division cycle 42 isoform 1	caspase-3	DLRDDPST
gi|4757952|ref|NP_001782.1| cell division cycle 42 isoform 1	caspase-7	DLRDDPST
gi|4758258|ref|NP_004086.1| eukaryotic translation initiation factor 4E binding protein 1	caspase-3	VLGDGVQL
gi|148233338|ref|NP_001091679.1| catenin (cadherin-associated protein) beta 1 88kDa	caspase-3	TQFDAAHP
gi|148233338|ref|NP_001091679.1| catenin (cadherin-associated protein) beta 1 88kDa	caspase-3	SYLDSGIH
gi|148233338|ref|NP_001091679.1| catenin (cadherin-associated protein) beta 1 88kDa	caspase-3	YPVDGLPD
gi|148233338|ref|NP_001091679.1| catenin (cadherin-associated protein) beta 1 88kDa	caspase-3	DLMDGLPP
gi|148233338|ref|NP_001091679.1| catenin (cadherin-associated protein) beta 1 88kDa	caspase-3	ADIDGQYA
gi|82534351|ref|NP_058519.2| microtubule-associated protein tau isoform 1	caspase-3	GSSDPLIQ
gi|82534351|ref|NP_058519.2| microtubule-associated protein tau isoform 1	caspase-3	DMVDSPQL
gi|82534351|ref|NP_058519.2| microtubule-associated protein tau isoform 1	caspase-1	DMVDSPQL
gi|82534351|ref|NP_058519.2| microtubule-associated protein tau isoform 1	caspase-7	GSSDPLIQ
gi|82534351|ref|NP_058519.2| microtubule-associated protein tau isoform 1	caspase-7	DMVDSPQL
gi|82534351|ref|NP_058519.2| microtubule-associated protein tau isoform 1	caspase-6	DMVDSPQL
gi|82534351|ref|NP_058519.2| microtubule-associated protein tau isoform 1	caspase-8	GSSDPLIQ
gi|82534351|ref|NP_058519.2| microtubule-associated protein tau isoform 1	caspase-8	DMVDSPQL
gi|119964718|ref|NP_001935.2| desmoglein 3 preproprotein	caspase-3	DYADGAIS
gi|118582269|ref|NP_001071634.1| splicing factor arginine/serine-rich 1 isoform 2	caspase-3	DLKDHMRE
gi|118582269|ref|NP_001071634.1| splicing factor arginine/serine-rich 1 isoform 2	caspase-3	CYADVYRD
gi|118582269|ref|NP_001071634.1| splicing factor arginine/serine-rich 1 isoform 2	caspase-3	VYRDGTGV
gi|118582269|ref|NP_001071634.1| splicing factor arginine/serine-rich 1 isoform 2	caspase-3	RKLDNTKF

gi|20631967|ref|NP_620119.1| BCL2-associated X protein isoform epsilon	caspase-3	FIQDRAGR
gi|48255955|ref|NP_001001344.1| plasma membrane calcium ATPase 3 isoform 3b	caspase-3	EEIDHAER
gi|48255955|ref|NP_001001344.1| plasma membrane calcium ATPase 3 isoform 3b	caspase-1	EEIDHAER
gi|48255955|ref|NP_001001344.1| plasma membrane calcium ATPase 3 isoform 3b	caspase-7	EEIDHAER
gi|55750053|ref|NP_001007027.1| atrophin-1	caspase-3	DSLDGRSL
gi|55750053|ref|NP_001007027.1| atrophin-1	caspase-7	DSLDGRSL
gi|55750053|ref|NP_001007027.1| atrophin-1	caspase-2	DSLDGRSL
gi|55750053|ref|NP_001007027.1| atrophin-1	caspase-8	DSLDGRSL
gi|42740903|ref|NP_872307.2| MTERF domain containing 2	caspase-3	DEDDDDEE
gi|56676393|ref|NP_001166.3| Rho GDP dissociation inhibitor (GDI) beta	caspase-3	DELDSKLN
gi|33598946|ref|NP_877963.1| phospholipase C gamma 1 isoform b	caspase-3	AEPDYGAL
gi|33598946|ref|NP_877963.1| phospholipase C gamma 1 isoform b	caspase-7	AEPDYGAL
gi|4506103|ref|NP_002750.1| eukaryotic translation initiation factor 2-alpha kinase 2	caspase-3	DLPDMKET
gi|156105681|ref|NP_036618.2| presenilin 2 isoform 2	caspase-3	DSYDSFGE
gi|41322923|ref|NP_958786.1| plectin 1 isoform 11	caspase-8	ILRDKDNT
gi|4557699|ref|NP_000214.1| keratin 12	caspase-6	VEMDAAPG
gi|4557699|ref|NP_000214.1| keratin 12	caspase-6	VEMDAAPG
gi|120407068|ref|NP_000537.3| tumor protein p53 isoform a	caspase-3	SDSDGLAP
gi|120407068|ref|NP_000537.3| tumor protein p53 isoform a	caspase-3	TFSDLWKL
gi|89903012|ref|NP_001034891.1| cell division cycle 42 isoform 1	caspase-3	DLRDDPST
gi|89903012|ref|NP_001034891.1| cell division cycle 42 isoform 1	caspase-7	DLRDDPST
gi|78190495|ref|NP_001030086.1| Kv channel interacting protein 3 isoform 2	caspase-3	DSSDSELE
gi|4557361|ref|NP_001187.1| BH3 interacting domain death agonist isoform 2	caspase-3	LQTDGNRS
gi|4557361|ref|NP_001187.1| BH3 interacting domain death agonist isoform 2	caspase-3	IEADSESQ
gi|4557361|ref|NP_001187.1| BH3 interacting domain death agonist isoform 2	caspase-8	LQTDGNRS
gi|4557361|ref|NP_001187.1| BH3 interacting domain death agonist isoform 2	caspase-8	IEADSESQ
gi|13518019|ref|NP_004984.2| ataxin 3 isoform 1	caspase-3	LISDTYLA
gi|13518019|ref|NP_004984.2| ataxin 3 isoform 1	caspase-3	DLPDCEAD
gi|13518019|ref|NP_004984.2| ataxin 3 isoform 1	caspase-3	DEEDLQRA
gi|90903231|ref|NP_002102.4| huntingtin	caspase-6	IVLDGTDN
gi|90903231|ref|NP_002102.4| huntingtin	caspase-2	DLNDGTQA

gi|90903231|ref|NP_002102.4| huntingtin	caspase-3	DSVDLASC
gi|90903231|ref|NP_002102.4| huntingtin	caspase-3	DEEDILSH
gi|90903231|ref|NP_002102.4| huntingtin	caspase-3	DLNDGTQA
gi|90903231|ref|NP_002102.4| huntingtin	caspase-3	IVLDGTDN
gi|6552315|ref|NP_009233.1| breast cancer 1 early onset isoform BRCA1-delta9-10	caspase-3	DLLDDGEI
gi|6552315|ref|NP_009233.1| breast cancer 1 early onset isoform BRCA1-delta9-10	caspase-3	DVLDVLNE
gi|20070130|ref|NP_001198.2| basic transcription factor 3 isoform B	caspase-3	QSVDGKAP
gi|41322910|ref|NP_958783.1| plectin 1 isoform 7	caspase-8	ILRDKDNT
gi|148227672|ref|NP_001091680.1| catenin (cadherin-associated protein) beta 1 88kDa	caspase-3	TQFDAAHP
gi|148227672|ref|NP_001091680.1| catenin (cadherin-associated protein) beta 1 88kDa	caspase-3	SYLDSGIH
gi|148227672|ref|NP_001091680.1| catenin (cadherin-associated protein) beta 1 88kDa	caspase-3	YPVDGLPD
gi|148227672|ref|NP_001091680.1| catenin (cadherin-associated protein) beta 1 88kDa	caspase-3	DLMDGLPP
gi|148227672|ref|NP_001091680.1| catenin (cadherin-associated protein) beta 1 88kDa	caspase-3	ADIDGQYA
gi|113420837|ref|XP_001126659.1| PREDICTED: hypothetical protein	caspase-3	DDVDTKKQ
gi|113420837|ref|XP_001126659.1| PREDICTED: hypothetical protein	caspase-3	DDEDDDVD
gi|113420837|ref|XP_001126659.1| PREDICTED: hypothetical protein	caspase-3	DEDDDVDT
gi|169790969|ref|NP_004553.2| parkin isoform 1	caspase-3	LHTDSRKD
gi|169790969|ref|NP_004553.2| parkin isoform 1	caspase-1	LHTDSRKD
gi|88953571|ref|XP_933678.1| PREDICTED: similar to protein expressed in prostate ovary testis and placenta 2 isoform 2	caspase-1	ELPDGQVI
gi|88953571|ref|XP_933678.1| PREDICTED: similar to protein expressed in prostate ovary testis and placenta 2 isoform 2	caspase-3	ELPDGQVI
gi|27477113|ref|NP_004590.2| sterol regulatory element-binding transcription factor 2	caspase-1	DEPDSPPV
gi|27477113|ref|NP_004590.2| sterol regulatory element-binding transcription factor 2	caspase-3	DEPDSPPV
gi|27477113|ref|NP_004590.2| sterol regulatory element-binding transcription factor 2	caspase-7	DEPDSPPV
gi|33598948|ref|NP_002651.2| phospholipase C gamma 1 isoform a	caspase-3	AEPDYGAL
gi|33598948|ref|NP_002651.2| phospholipase C gamma 1 isoform a	caspase-7	AEPDYGAL
gi|16357472|ref|NP_426359.1| cell division cycle 42 isoform 2	caspase-3	DLRDDPST
gi|16357472|ref|NP_426359.1| cell division cycle 42 isoform 2	caspase-7	DLRDDPST

gi|14916483|ref|NP_001217.2| caspase 6 isoform alpha preproprotein	caspase-3	DVVDNQTE
gi|14916483|ref|NP_001217.2| caspase 6 isoform alpha preproprotein	caspase-3	TEVDAASV
gi|14916483|ref|NP_001217.2| caspase 6 isoform alpha preproprotein	caspase-3	TETDAFYK
gi|48255877|ref|NP_000355.2| troponin T type 2 cardiac isoform 1	caspase-3	VDFDDIHR
gi|48255945|ref|NP_001673.2| plasma membrane calcium ATPase 1 isoform 1b	caspase-3	EEIDHAER
gi|48255945|ref|NP_001673.2| plasma membrane calcium ATPase 1 isoform 1b	caspase-1	EEIDHAER
gi|48255945|ref|NP_001673.2| plasma membrane calcium ATPase 1 isoform 1b	caspase-7	EEIDHAER
gi|37574726|ref|NP_932070.1| BH3 interacting domain death agonist isoform 1	caspase-3	LQTDGNRS
gi|37574726|ref|NP_932070.1| BH3 interacting domain death agonist isoform 1	caspase-3	IEADSESQ
gi|37574726|ref|NP_932070.1| BH3 interacting domain death agonist isoform 1	caspase-8	LQTDGNRS
gi|37574726|ref|NP_932070.1| BH3 interacting domain death agonist isoform 1	caspase-8	IEADSESQ
gi|113413200|ref|XP_934799.2| PREDICTED: similar to protein expressed in prostate ovary testis and placenta 2	caspase-1	ELPDGQVI
gi|113413200|ref|XP_934799.2| PREDICTED: similar to protein expressed in prostate ovary testis and placenta 2	caspase-3	ELPDGQVI
gi|113420848|ref|XP_001127088.1| PREDICTED: hypothetical protein	caspase-3	DDVDTKKQ
gi|113420848|ref|XP_001127088.1| PREDICTED: hypothetical protein	caspase-3	DDEDDDVD
gi|113420848|ref|XP_001127088.1| PREDICTED: hypothetical protein	caspase-3	DEDDDVDT
gi|4502381|ref|NP_001182.1| BCL2-like 1 isoform 2	caspase-1	HLADSPAV
gi|4502381|ref|NP_001182.1| BCL2-like 1 isoform 2	caspase-3	HLADSPAV
gi|4502381|ref|NP_001182.1| BCL2-like 1 isoform 2	caspase-3	SSLDAREV
gi|187830777|ref|NP_001119584.1| tumor protein p53 isoform a	caspase-3	SDSDGLAP
gi|187830777|ref|NP_001119584.1| tumor protein p53 isoform a	caspase-3	TFSDLWKL
gi|48255951|ref|NP_001001331.1| plasma membrane calcium ATPase 2 isoform 1	caspase-3	EEIDHAER
gi|48255951|ref|NP_001001331.1| plasma membrane calcium ATPase 2 isoform 1	caspase-1	EEIDHAER
gi|48255951|ref|NP_001001331.1| plasma membrane calcium ATPase 2 isoform 1	caspase-7	EEIDHAER
gi|4502703|ref|NP_001245.1| cell division cycle 6 protein	caspase-3	SEVDGNRM
gi|4502703|ref|NP_001245.1| cell division cycle 6 protein	caspase-3	LVFDNQLT

gi|48255949|ref|NP_001674.2| plasma membrane calcium ATPase 2 isoform 2	caspase-3	EEIDHAER
gi|48255949|ref|NP_001674.2| plasma membrane calcium ATPase 2 isoform 2	caspase-1	EEIDHAER
gi|48255949|ref|NP_001674.2| plasma membrane calcium ATPase 2 isoform 2	caspase-7	EEIDHAER
gi|67089157|ref|NP_150243.2| promyelocytic leukemia protein isoform 2	caspase-3	PHLDGPPS
gi|62198237|ref|NP_006222.2| DNA polymerase epsilon catalytic subunit	caspase-3	DMEDFGLV
gi|62198237|ref|NP_006222.2| DNA polymerase epsilon catalytic subunit	caspase-3	DQLDNIVD
gi|13518013|ref|NP_109376.1| ataxin 3 isoform 2	caspase-3	LISDTYLA
gi|13518013|ref|NP_109376.1| ataxin 3 isoform 2	caspase-3	DLPDCEAD
gi|13518013|ref|NP_109376.1| ataxin 3 isoform 2	caspase-3	DEEDLQRA
gi|86262149|ref|NP_001034449.1| zinc finger and BTB domain containing 7C	caspase-3	DEDDDDEE
gi|31377798|ref|NP_004694.2| rabaptin RAB GTPase binding effector protein 1 isoform 1	caspase-3	DESDFGPL
gi|8400715|ref|NP_058525.1| microtubule-associated protein tau isoform 4	caspase-3	DMVDSPQL
gi|8400715|ref|NP_058525.1| microtubule-associated protein tau isoform 4	caspase-1	DMVDSPQL
gi|8400715|ref|NP_058525.1| microtubule-associated protein tau isoform 4	caspase-7	DMVDSPQL
gi|8400715|ref|NP_058525.1| microtubule-associated protein tau isoform 4	caspase-6	DMVDSPQL
gi|8400715|ref|NP_058525.1| microtubule-associated protein tau isoform 4	caspase-8	DMVDSPQL
gi|4501885|ref|NP_001092.1| beta actin	caspase-1	LVVDNGSG
gi|4501885|ref|NP_001092.1| beta actin	caspase-1	ELPDGQVI
gi|4501885|ref|NP_001092.1| beta actin	caspase-3	ELPDGQVI
gi|67089149|ref|NP_150241.2| promyelocytic leukemia protein isoform 1	caspase-3	PHLDGPPS
gi|89035369|ref|XP_933168.1| PREDICTED: similar to prothymosin alpha isoform 1	caspase-3	DDEDDDVD
gi|89035369|ref|XP_933168.1| PREDICTED: similar to prothymosin alpha isoform 1	caspase-3	DEDDDVDT
gi|48255959|ref|NP_001001396.1| plasma membrane calcium ATPase 4 isoform 4a	caspase-3	DEIDHAEM
gi|48255959|ref|NP_001001396.1| plasma membrane calcium ATPase 4 isoform 4a	caspase-1	DEIDHAEM
gi|195972799|ref|NP_005578.2| myocyte enhancer factor 2A isoform 1	caspase-3	TLTDSSML
gi|195972799|ref|NP_005578.2| myocyte enhancer factor 2A isoform 1	caspase-3	STTDLTVP
gi|195972799|ref|NP_005578.2| myocyte enhancer factor 2A isoform 1	caspase-3	SSYDGSDR

gi|195972799|ref|NP_005578.2| myocyte enhancer factor 2A isoform 1	caspase-7	STTDLTVP
gi|195972799|ref|NP_005578.2| myocyte enhancer factor 2A isoform 1	caspase-7	SSYDGSDR
gi|195972799|ref|NP_005578.2| myocyte enhancer factor 2A isoform 1	caspase-6	TLTDSSML
gi|195972799|ref|NP_005578.2| myocyte enhancer factor 2A isoform 1	caspase-3	SSYDGSDR
gi|195972799|ref|NP_005578.2| myocyte enhancer factor 2A isoform 1	caspase-7	SSYDGSDR
gi|4504447|ref|NP_002128.1| heterogeneous nuclear ribonucleoprotein A2/B1 isoform A2	caspase-3	KLTDCVVM
gi|4504447|ref|NP_002128.1| heterogeneous nuclear ribonucleoprotein A2/B1 isoform A2	caspase-3	VMRDPASK
gi|4504447|ref|NP_002128.1| heterogeneous nuclear ribonucleoprotein A2/B1 isoform A2	caspase-3	AEVDAAMA
gi|195972801|ref|NP_001124398.1| myocyte enhancer factor 2A isoform 2	caspase-3	TLTDSSML
gi|195972801|ref|NP_001124398.1| myocyte enhancer factor 2A isoform 2	caspase-3	STTDLTVP
gi|195972801|ref|NP_001124398.1| myocyte enhancer factor 2A isoform 2	caspase-3	SSYDGSDR
gi|195972801|ref|NP_001124398.1| myocyte enhancer factor 2A isoform 2	caspase-7	STTDLTVP
gi|195972801|ref|NP_001124398.1| myocyte enhancer factor 2A isoform 2	caspase-7	SSYDGSDR
gi|195972801|ref|NP_001124398.1| myocyte enhancer factor 2A isoform 2	caspase-6	TLTDSSML
gi|195972801|ref|NP_001124398.1| myocyte enhancer factor 2A isoform 2	caspase-3	SSYDGSDR
gi|195972801|ref|NP_001124398.1| myocyte enhancer factor 2A isoform 2	caspase-7	SSYDGSDR
gi|62414289|ref|NP_003371.2| vimentin	caspase-3	DSVDFSLA
gi|62414289|ref|NP_003371.2| vimentin	caspase-7	DSVDFSLA
gi|62414289|ref|NP_003371.2| vimentin	caspase-6	IDVDVSKP
gi|62414289|ref|NP_003371.2| vimentin	caspase-9	IDVDVSKP
gi|195972803|ref|NP_001124399.1| myocyte enhancer factor 2A isoform 3	caspase-3	TLTDSSML
gi|195972803|ref|NP_001124399.1| myocyte enhancer factor 2A isoform 3	caspase-3	STTDLTVP
gi|195972803|ref|NP_001124399.1| myocyte enhancer factor 2A isoform 3	caspase-3	SSYDGSDR
gi|195972803|ref|NP_001124399.1| myocyte enhancer factor 2A isoform 3	caspase-7	STTDLTVP
gi|195972803|ref|NP_001124399.1| myocyte enhancer factor 2A isoform 3	caspase-7	SSYDGSDR
gi|195972803|ref|NP_001124399.1| myocyte enhancer factor 2A isoform 3	caspase-6	TLTDSSML

gi|195972803|ref|NP_001124399.1| myocyte enhancer factor 2A isoform 3	caspase-3	SSYDGSDR
gi|195972803|ref|NP_001124399.1| myocyte enhancer factor 2A isoform 3	caspase-7	SSYDGSDR
gi|42544182|ref|NP_975010.1| BMX non-receptor tyrosine kinase	caspase-3	DFPDWWQV
gi|187830823|ref|NP_001119585.1| tumor protein p53 isoform c	caspase-3	SDSDGLAP
gi|187830823|ref|NP_001119585.1| tumor protein p53 isoform c	caspase-3	TFSDLWKL
gi|42741655|ref|NP_000236.2| met proto-oncogene isoform b precursor	caspase-3	ESVDYRAT
gi|154354996|ref|NP_002213.4| inositol 1 4 5-triphosphate receptor type 1 isoform 2	caspase-3	DEVDRDAP
gi|195972805|ref|NP_001124400.1| myocyte enhancer factor 2A isoform 4	caspase-3	TLTDSSML
gi|195972805|ref|NP_001124400.1| myocyte enhancer factor 2A isoform 4	caspase-3	STTDLTVP
gi|195972805|ref|NP_001124400.1| myocyte enhancer factor 2A isoform 4	caspase-3	SSYDGSDR
gi|195972805|ref|NP_001124400.1| myocyte enhancer factor 2A isoform 4	caspase-7	STTDLTVP
gi|195972805|ref|NP_001124400.1| myocyte enhancer factor 2A isoform 4	caspase-7	SSYDGSDR
gi|195972805|ref|NP_001124400.1| myocyte enhancer factor 2A isoform 4	caspase-6	TLTDSSML
gi|195972805|ref|NP_001124400.1| myocyte enhancer factor 2A isoform 4	caspase-3	SSYDGSDR
gi|195972805|ref|NP_001124400.1| myocyte enhancer factor 2A isoform 4	caspase-7	SSYDGSDR
gi|4557888|ref|NP_000215.1| keratin 18	caspase-3	VEVDAPKS
gi|4557888|ref|NP_000215.1| keratin 18	caspase-7	EVDAPKSQ
gi|4557888|ref|NP_000215.1| keratin 18	caspase-6	EVDAPKSQ
gi|4505541|ref|NP_003706.1| USO1 homolog vesicle docking protein	caspase-8	TEKDSMIE
gi|4505541|ref|NP_003706.1| USO1 homolog vesicle docking protein	caspase-3	TEKDSMIE
gi|149158692|ref|NP_004630.3| HLA-B associated transcript-3 isoform a	caspase-3	DEQDGASA
gi|48255879|ref|NP_001001430.1| troponin T type 2 cardiac isoform 2	caspase-3	VDFDDIHR
gi|57242761|ref|NP_003795.2| receptor (TNFRSF)-interacting serine-threonine kinase 1	caspase-8	LQLDCVAV
gi|22027618|ref|NP_663777.1| TNF receptor-associated factor 3 isoform 1	caspase-3	EEADSMKS
gi|22027618|ref|NP_663777.1| TNF receptor-associated factor 3 isoform 1	caspase-3	ESVDKSAG

gi|4826962|ref|NP_005043.1| ras-related C3 botulinum toxin substrate 3 (rho family small GTP binding protein Rac3)	caspase-3	DLRDDKDT
gi|15451779|ref|NP_150252.1| promyelocytic leukemia protein isoform 10	caspase-3	PHLDGPPS
gi|154354998|ref|NP_001093422.1| inositol 1 4 5-triphosphate receptor type 1 isoform 1	caspase-3	DEVDRDAP
gi|5031755|ref|NP_005817.1| heterogeneous nuclear ribonucleoprotein R isoform 2	caspase-3	YPPDYYGY
gi|5031755|ref|NP_005817.1| heterogeneous nuclear ribonucleoprotein R isoform 2	caspase-3	DYYDDYYG
gi|5031755|ref|NP_005817.1| heterogeneous nuclear ribonucleoprotein R isoform 2	caspase-3	DYHDYRGG
gi|5031755|ref|NP_005817.1| heterogeneous nuclear ribonucleoprotein R isoform 2	caspase-3	RAIDALRE
gi|5031755|ref|NP_005817.1| heterogeneous nuclear ribonucleoprotein R isoform 2	caspase-3	KESDLSHV
gi|4504653|ref|NP_001553.1| interleukin 18 proprotein	caspase-3	DMTDSDCR
gi|4504653|ref|NP_001553.1| interleukin 18 proprotein	caspase-1	LESDYFGK
gi|22027620|ref|NP_663778.1| TNF receptor-associated factor 3 isoform 2	caspase-3	EEADSMKS
gi|22027620|ref|NP_663778.1| TNF receptor-associated factor 3 isoform 2	caspase-3	ESVDKSAG
gi|189163493|ref|NP_001121168.1| ataxin 3 isoform 3	caspase-3	LISDTYLA
gi|189163493|ref|NP_001121168.1| ataxin 3 isoform 3	caspase-3	DLPDCEAD
gi|189163493|ref|NP_001121168.1| ataxin 3 isoform 3	caspase-3	DEEDLQRA
gi|156105679|ref|NP_000438.2| presenilin 2 isoform 1	caspase-3	DSYDSFGE
gi|6754638|ref|NP_005901.2| microtubule-associated protein tau isoform 2	caspase-3	DMVDSPQL
gi|6754638|ref|NP_005901.2| microtubule-associated protein tau isoform 2	caspase-1	DMVDSPQL
gi|6754638|ref|NP_005901.2| microtubule-associated protein tau isoform 2	caspase-7	DMVDSPQL
gi|6754638|ref|NP_005901.2| microtubule-associated protein tau isoform 2	caspase-6	DMVDSPQL
gi|6754638|ref|NP_005901.2| microtubule-associated protein tau isoform 2	caspase-8	DMVDSPQL
gi|67089160|ref|NP_150253.2| promyelocytic leukemia protein isoform 11	caspase-3	PHLDGPPS
gi|187830855|ref|NP_001119586.1| tumor protein p53 isoform b	caspase-3	SDSDGLAP
gi|187830855|ref|NP_001119586.1| tumor protein p53 isoform b	caspase-3	TFSDLWKL
gi|170932514|ref|NP_001074324.1| paxillin isoform 1	caspase-3	NPQDSVGS
gi|170932514|ref|NP_001074324.1| paxillin isoform 1	caspase-3	SELDRLLL
gi|170932514|ref|NP_001074324.1| paxillin isoform 1	caspase-3	FPADEANS
gi|170932514|ref|NP_001074324.1| paxillin isoform 1	caspase-3	SLLDELES
gi|170932514|ref|NP_001074324.1| paxillin isoform 1	caspase-3	SQLDSMLG
gi|170932514|ref|NP_001074324.1| paxillin isoform 1	caspase-3	DDLDALLA

gi|14043072|ref|NP_112533.1| heterogeneous nuclear ribonucleoprotein A2/B1 isoform B1	caspase-3	KLTDCVVM
gi|14043072|ref|NP_112533.1| heterogeneous nuclear ribonucleoprotein A2/B1 isoform B1	caspase-3	VMRDPASK
gi|14043072|ref|NP_112533.1| heterogeneous nuclear ribonucleoprotein A2/B1 isoform B1	caspase-3	AEVDAAMA
gi|21361670|ref|NP_054782.2| drebrin-like isoform a	caspase-3	EHIDHHIQ
gi|62198235|ref|NP_001014436.1| drebrin-like isoform b	caspase-3	EHIDHHIQ
gi|171906575|ref|NP_001116428.1| drebrin-like isoform c	caspase-3	EHIDHHIQ
gi|115529463|ref|NP_002733.2| protein kinase D1	caspase-3	DDNDSERN
gi|5453994|ref|NP_006256.1| RAD21 homolog	caspase-7	DSPDSVDP
gi|5453994|ref|NP_006256.1| RAD21 homolog	caspase-3	DSPDSVDP
gi|48255957|ref|NP_001675.3| plasma membrane calcium ATPase 4 isoform 4b	caspase-3	DEIDHAEM
gi|48255957|ref|NP_001675.3| plasma membrane calcium ATPase 4 isoform 4b	caspase-1	DEIDHAEM
gi|48255881|ref|NP_001001431.1| troponin T type 2 cardiac isoform 3	caspase-3	VDFDDIHR
gi|5803227|ref|NP_006817.1| tyrosine 3/tryptophan 5 -monooxygenase activation protein theta polypeptide	caspase-3	EECDAAEG
gi|5803227|ref|NP_006817.1| tyrosine 3/tryptophan 5 -monooxygenase activation protein theta polypeptide	caspase-7	EECDAAEG
gi|5803227|ref|NP_006817.1| tyrosine 3/tryptophan 5 -monooxygenase activation protein theta polypeptide	caspase-8	EECDAAEG
gi|169790971|ref|NP_054642.2| parkin isoform 2	caspase-3	LHTDSRKD
gi|169790971|ref|NP_054642.2| parkin isoform 2	caspase-1	LHTDSRKD
gi|30089940|ref|NP_005886.2| Golgi autoantigen golgin subfamily a 3	caspase-7	SEVDGNDS
gi|30089940|ref|NP_005886.2| Golgi autoantigen golgin subfamily a 3	caspase-3	CSTDSPLP
gi|30089940|ref|NP_005886.2| Golgi autoantigen golgin subfamily a 3	caspase-2	CSTDSPLP
gi|30089940|ref|NP_005886.2| Golgi autoantigen golgin subfamily a 3	caspase-2	SEVDGNDS
gi|30089940|ref|NP_005886.2| Golgi autoantigen golgin subfamily a 3	caspase-2	ESPDGPGQ
gi|10835145|ref|NP_000567.1| interleukin 1 beta proprotein	caspase-1	YVHDAPVR
gi|10835145|ref|NP_000567.1| interleukin 1 beta proprotein	caspase-1	FEADGPKQ
gi|187830894|ref|NP_001119587.1| tumor protein p53 isoform d	caspase-3	SDSDGLAP
gi|170932516|ref|NP_002850.2| paxillin isoform 2	caspase-3	NPQDSVGS
gi|170932516|ref|NP_002850.2| paxillin isoform 2	caspase-3	SELDRLLL
gi|170932516|ref|NP_002850.2| paxillin isoform 2	caspase-3	FPADEANS
gi|170932516|ref|NP_002850.2| paxillin isoform 2	caspase-3	SLLDELES
gi|170932516|ref|NP_002850.2| paxillin isoform 2	caspase-3	SQLDSMLG
gi|170932516|ref|NP_002850.2| paxillin isoform 2	caspase-3	DDLDALLA
gi|189163495|ref|NP_001121169.1| ataxin 3 isoform 4	caspase-3	LISDTYLA

gi|189163495|ref|NP_001121169.1| ataxin 3 isoform 4	caspase-3	DLPDCEAD
gi|189163495|ref|NP_001121169.1| ataxin 3 isoform 4	caspase-3	DEEDLQRA
gi|4885065|ref|NP_005157.1| amyloid precursor-like protein 1 isoform 2 precursor	caspase-3	VEVDPMLT
gi|7662238|ref|NP_055792.1| apoptotic chromatin condensation inducer 1	caspase-3	DELDYHRG
gi|61966711|ref|NP_001013653.1| heterogeneous nuclear ribonucleoprotein C-like 1	caspase-3	EGEDDRDS
gi|18375630|ref|NP_542433.1| HLA-B associated transcript-3 isoform b	caspase-3	DEQDGASA
gi|15718704|ref|NP_001219.2| caspase 8 isoform A precursor	caspase-8	VETDSEEQ
gi|4757960|ref|NP_004351.1| cadherin 1 type 1 preproprotein	caspase-3	DTRDNVYY
gi|109633026|ref|NP_001035917.1| serine/threonine protein kinase MST4 isoform 3	caspase-3	DESDSEGS
gi|48255953|ref|NP_068768.2| plasma membrane calcium ATPase 3 isoform 3a	caspase-3	EEIDHAER
gi|48255953|ref|NP_068768.2| plasma membrane calcium ATPase 3 isoform 3a	caspase-1	EEIDHAER
gi|48255953|ref|NP_068768.2| plasma membrane calcium ATPase 3 isoform 3a	caspase-7	EEIDHAER
gi|109638749|ref|NP_008878.3| signal recognition particle 72kDa	caspase-3	SELDASKT
gi|134133226|ref|NP_001077007.1| protein expressed in prostate ovary testis and placenta 2	caspase-1	ELPDGQVI
gi|134133226|ref|NP_001077007.1| protein expressed in prostate ovary testis and placenta 2	caspase-3	ELPDGQVI
gi|187830901|ref|NP_001119588.1| tumor protein p53 isoform e	caspase-3	SDSDGLAP
gi|29725609|ref|NP_005219.2| epidermal growth factor receptor isoform a precursor	caspase-3	DEEDMDDV
gi|29725609|ref|NP_005219.2| epidermal growth factor receptor isoform a precursor	caspase-3	DMDDVVDA
gi|15718706|ref|NP_203519.1| caspase 8 isoform B precursor	caspase-8	VETDSEEQ
gi|170932518|ref|NP_079433.3| paxillin isoform 3	caspase-3	SELDRLLL
gi|170932518|ref|NP_079433.3| paxillin isoform 3	caspase-3	FPADEANS
gi|170932518|ref|NP_079433.3| paxillin isoform 3	caspase-3	SLLDELES
gi|170932518|ref|NP_079433.3| paxillin isoform 3	caspase-3	SQLDSMLG
gi|4503925|ref|NP_002040.1| GATA binding protein 1	caspase-7	EDLDGKGS
gi|4503925|ref|NP_002040.1| GATA binding protein 1	caspase-8	EDLDGKGS
gi|4503925|ref|NP_002040.1| GATA binding protein 1	caspase-9	EDLDGKGS
gi|4503925|ref|NP_002040.1| GATA binding protein 1	caspase-10	EDLDGKGS
gi|4503925|ref|NP_002040.1| GATA binding protein 1	caspase-3	EDLDGKGS
gi|4502167|ref|NP_000475.1| amyloid beta A4 protein precursor isoform a	caspase-6	VKMDAEFR
gi|4502167|ref|NP_000475.1| amyloid beta A4 protein precursor isoform a	caspase-6	VEVDAAVT

gi|4502167|ref|NP_000475.1| amyloid beta A4 protein precursor isoform a	caspase-3	VEVDAAVT
gi|16332358|ref|NP_277021.1| cell division cycle 2-like 1 (PITSLRE proteins) isoform 2	caspase-3	YVPDSPAL
gi|16332358|ref|NP_277021.1| cell division cycle 2-like 1 (PITSLRE proteins) isoform 2	caspase-1	YVPDSPAL
gi|22547195|ref|NP_004167.3| sterol regulatory element binding transcription factor 1 isoform b	caspase-1	SEPDSPVF
gi|4507693|ref|NP_000359.1| tuberous sclerosis 1 protein isoform 1	caspase-6	TEEDGVPS
gi|4507693|ref|NP_000359.1| tuberous sclerosis 1 protein isoform 1	caspase-8	TEEDGVPS
gi|4507693|ref|NP_000359.1| tuberous sclerosis 1 protein isoform 1	caspase-9	TEEDGVPS
gi|16332360|ref|NP_277022.1| cell division cycle 2-like 1 (PITSLRE proteins) isoform 3	caspase-3	YVPDSPAL
gi|16332360|ref|NP_277022.1| cell division cycle 2-like 1 (PITSLRE proteins) isoform 3	caspase-1	YVPDSPAL
gi|8400711|ref|NP_058518.1| microtubule-associated protein tau isoform 3	caspase-3	DMVDSPQL
gi|8400711|ref|NP_058518.1| microtubule-associated protein tau isoform 3	caspase-1	DMVDSPQL
gi|8400711|ref|NP_058518.1| microtubule-associated protein tau isoform 3	caspase-7	DMVDSPQL
gi|8400711|ref|NP_058518.1| microtubule-associated protein tau isoform 3	caspase-6	DMVDSPQL
gi|8400711|ref|NP_058518.1| microtubule-associated protein tau isoform 3	caspase-8	DMVDSPQL
gi|4502057|ref|NP_000689.1| arachidonate 5-lipoxygenase	caspase-6	IQFDSEKG
gi|16332362|ref|NP_277023.1| cell division cycle 2-like 1 (PITSLRE proteins) isoform 4	caspase-3	YVPDSPAL
gi|16332362|ref|NP_277023.1| cell division cycle 2-like 1 (PITSLRE proteins) isoform 4	caspase-1	YVPDSPAL
gi|16332364|ref|NP_277024.1| cell division cycle 2-like 1 (PITSLRE proteins) isoform 5	caspase-3	YVPDSPAL
gi|16332364|ref|NP_277024.1| cell division cycle 2-like 1 (PITSLRE proteins) isoform 5	caspase-1	YVPDSPAL
gi|52630419|ref|NP_001005291.1| sterol regulatory element binding transcription factor 1 isoform a	caspase-1	SEPDSPVF
gi|187830909|ref|NP_001119589.1| tumor protein p53 isoform f	caspase-3	SDSDGLAP
gi|48255883|ref|NP_001001432.1| troponin T type 2 cardiac isoform 4	caspase-3	VDFDDIHR
gi|41406055|ref|NP_958816.1| amyloid beta A4 protein precursor isoform b	caspase-6	VKMDAEFR
gi|41406055|ref|NP_958816.1| amyloid beta A4 protein precursor isoform b	caspase-6	VEVDAAVT

gi|41406055|ref|NP_958816.1| amyloid beta A4 protein precursor isoform b	caspase-3	VEVDAAVT
gi|24476013|ref|NP_722560.1| PTK2 protein tyrosine kinase 2 isoform a	caspase-7	DQTDSWNH
gi|24476013|ref|NP_722560.1| PTK2 protein tyrosine kinase 2 isoform a	caspase-6	VSWDSGGS
gi|24476013|ref|NP_722560.1| PTK2 protein tyrosine kinase 2 isoform a	caspase-3	DQTDSWNH
gi|15718708|ref|NP_203520.1| caspase 8 isoform C precursor	caspase-8	VETDSEEQ
gi|16332370|ref|NP_277027.1| cell division cycle 2-like 1 (PITSLRE proteins) isoform 8	caspase-3	YVPDSPAL
gi|16332370|ref|NP_277027.1| cell division cycle 2-like 1 (PITSLRE proteins) isoform 8	caspase-1	YVPDSPAL
gi|41406057|ref|NP_958817.1| amyloid beta A4 protein precursor isoform c	caspase-6	VKMDAEFR
gi|41406057|ref|NP_958817.1| amyloid beta A4 protein precursor isoform c	caspase-6	VEVDAAVT
gi|41406057|ref|NP_958817.1| amyloid beta A4 protein precursor isoform c	caspase-3	VEVDAAVT
gi|16332372|ref|NP_277028.1| cell division cycle 2-like 1 (PITSLRE proteins) isoform 9	caspase-3	YVPDSPAL
gi|16332372|ref|NP_277028.1| cell division cycle 2-like 1 (PITSLRE proteins) isoform 9	caspase-1	YVPDSPAL
gi|23397427|ref|NP_006363.3| synaptotagmin binding cytoplasmic RNA interacting protein	caspase-3	YPPDYYGY
gi|23397427|ref|NP_006363.3| synaptotagmin binding cytoplasmic RNA interacting protein	caspase-3	GYEDYYDY
gi|23397427|ref|NP_006363.3| synaptotagmin binding cytoplasmic RNA interacting protein	caspase-3	DYYDYYGY
gi|34932414|ref|NP_031389.3| non-POU domain containing octamer-binding	caspase-3	MMPDGTLG
gi|4885049|ref|NP_005150.1| cardiac muscle alpha actin 1 proprotein	caspase-1	ELPDGQVI
gi|4885049|ref|NP_005150.1| cardiac muscle alpha actin 1 proprotein	caspase-3	ELPDGQVI
gi|18375632|ref|NP_542434.1| HLA-B associated transcript-3 isoform b	caspase-3	DEQDGASA
gi|29826325|ref|NP_789771.1| adducin 1 (alpha) isoform d	caspase-3	DIVDRGST
gi|29826325|ref|NP_789771.1| adducin 1 (alpha) isoform d	caspase-3	DRVDENNP
gi|27262655|ref|NP_004504.3| interleukin 16 isoform 1 precursor	caspase-3	SSTDSAAS
gi|4758256|ref|NP_004085.1| eukaryotic translation initiation factor 2 subunit 1 alpha 35kDa	caspase-3	AEVDGDDD
gi|4758256|ref|NP_004085.1| eukaryotic translation initiation factor 2 subunit 1 alpha 35kDa	caspase-3	DGDDDAEE

gi|29826319|ref|NP_001110.2| adducin 1 (alpha) isoform a	caspase-3	DIVDRGST
gi|29826319|ref|NP_001110.2| adducin 1 (alpha) isoform a	caspase-3	DRVDENNP
gi|29826319|ref|NP_001110.2| adducin 1 (alpha) isoform a	caspase-3	DDSDAATF
gi|62953113|ref|NP_001017534.1| caspase-1 dominant-negative inhibitor pseudo-ICE isoform 1	caspase-1	AVQDNPAM
gi|24430190|ref|NP_002266.2| keratin 15	caspase-3	ESVDGQVV
gi|24430190|ref|NP_002266.2| keratin 15	caspase-7	ESVDGQVV
gi|24430190|ref|NP_002266.2| keratin 15	caspase-6	VEMDAAPG
gi|24430190|ref|NP_002266.2| keratin 15	caspase-6	VEMDAAPG
gi|109637788|ref|NP_150247.2| promyelocytic leukemia protein isoform 5	caspase-3	PHLDGPPS
gi|5174545|ref|NP_005911.1| myocyte enhancer factor 2D	caspase-7	LTEDHLDL
gi|5174545|ref|NP_005911.1| myocyte enhancer factor 2D	caspase-7	DHLDLNNA
gi|55956788|ref|NP_005372.2| nucleolin	caspase-3	TEIDGRSI
gi|55956788|ref|NP_005372.2| nucleolin	caspase-3	AMEDGEID
gi|55956788|ref|NP_005372.2| nucleolin	caspase-3	GEIDGNKV
gi|62241011|ref|NP_005154.2| v-akt murine thymoma viral oncogene homolog 1	caspase-3	TVADGLKK
gi|62241011|ref|NP_005154.2| v-akt murine thymoma viral oncogene homolog 1	caspase-3	EEMDFRSG
gi|62241011|ref|NP_005154.2| v-akt murine thymoma viral oncogene homolog 1	caspase-3	ECVDSERR
gi|122056474|ref|NP_001073593.1| caspase 8 isoform C precursor	caspase-8	VETDSEEQ
gi|109633028|ref|NP_001035918.1| serine/threonine protein kinase MST4 isoform 2	caspase-3	DESDSEGS
gi|4502557|ref|NP_001735.1| calcium/calmodulin-dependent protein kinase IV	caspase-3	PAPDAPLK
gi|4502557|ref|NP_001735.1| calcium/calmodulin-dependent protein kinase IV	caspase-3	YWIDGSNR
gi|4502557|ref|NP_001735.1| calcium/calmodulin-dependent protein kinase IV	caspase-3	YWIDGSNR
gi|15718702|ref|NP_203126.1| caspase 7 isoform beta	caspase-3	DSVDAKPD
gi|15718702|ref|NP_203126.1| caspase 7 isoform beta	caspase-7	DSVDAKPD
gi|148833504|ref|NP_757366.2| interleukin 16 isoform 2	caspase-3	SSTDSAAS
gi|4502123|ref|NP_001151.1| apoptotic peptidase activating factor 1 isoform b	caspase-3	SVTDSVMG
gi|62241013|ref|NP_001014431.1| v-akt murine thymoma viral oncogene homolog 1	caspase-3	TVADGLKK
gi|62241013|ref|NP_001014431.1| v-akt murine thymoma viral oncogene homolog 1	caspase-3	EEMDFRSG
gi|62241013|ref|NP_001014431.1| v-akt murine thymoma viral oncogene homolog 1	caspase-3	ECVDSERR

gi|122056476|ref|NP_001073594.1| caspase 8 isoform G precursor	caspase-8	VETDSEEQ
gi|27436946|ref|NP_733821.1| lamin A/C isoform 1 precursor	caspase-6	VEIDNGKQ
gi|4505055|ref|NP_002341.1| Yamaguchi sarcoma viral (v-yes-1) oncogene homolog isoform A	caspase-3	DGVDLKTQ
gi|48255947|ref|NP_001001323.1| plasma membrane calcium ATPase 1 isoform 1a	caspase-3	EEIDHAER
gi|48255947|ref|NP_001001323.1| plasma membrane calcium ATPase 1 isoform 1a	caspase-1	EEIDHAER
gi|48255947|ref|NP_001001323.1| plasma membrane calcium ATPase 1 isoform 1a	caspase-7	EEIDHAER
gi|15451765|ref|NP_150242.1| promyelocytic leukemia protein isoform 9	caspase-3	PHLDGPPS
gi|6552299|ref|NP_009225.1| breast cancer 1 early onset isoform 1	caspase-3	DLLDDGEI
gi|6552299|ref|NP_009225.1| breast cancer 1 early onset isoform 1	caspase-3	DVLDVLNE
gi|17978495|ref|NP_510867.1| cyclin-dependent kinase inhibitor 1A	caspase-3	DHVDLSLS
gi|17978495|ref|NP_510867.1| cyclin-dependent kinase inhibitor 1A	caspase-3	SMTDFYHS
gi|4502581|ref|NP_001218.1| caspase 7 isoform alpha precursor	caspase-3	DSVDAKPD
gi|4502581|ref|NP_001218.1| caspase 7 isoform alpha precursor	caspase-7	DSVDAKPD
gi|4502581|ref|NP_001218.1| caspase 7 isoform alpha precursor	caspase-6	IQADSGPI
gi|4502581|ref|NP_001218.1| caspase 7 isoform alpha precursor	caspase-6	NDTDANPR
gi|38201623|ref|NP_937884.1| eukaryotic translation initiation factor 4 gamma 1 isoform 1	caspase-3	DRLDRART
gi|38201623|ref|NP_937884.1| eukaryotic translation initiation factor 4 gamma 1 isoform 1	caspase-3	DLLDAFKE
gi|30410796|ref|NP_789839.1| proteasome activator subunit 3 isoform 2	caspase-3	DGLDGPTY
gi|169165336|ref|XP_001714623.1| PREDICTED: similar to ATPase Ca++ transporting plasma membrane 2	caspase-3	EEIDHAER
gi|169165336|ref|XP_001714623.1| PREDICTED: similar to ATPase Ca++ transporting plasma membrane 2	caspase-1	EEIDHAER
gi|169165336|ref|XP_001714623.1| PREDICTED: similar to ATPase Ca++ transporting plasma membrane 2	caspase-7	EEIDHAER
gi|4506473|ref|NP_002899.1| v-rel reticuloendotheliosis viral oncogene homolog	caspase-3	DCRDGYYE
gi|156151392|ref|NP_001095867.1| heterogeneous nuclear ribonucleoprotein R isoform 4	caspase-3	YPPDYYGY
gi|156151392|ref|NP_001095867.1| heterogeneous nuclear ribonucleoprotein R isoform 4	caspase-3	DYYDDYYG

gi|156151392|ref|NP_001095867.1| heterogeneous nuclear ribonucleoprotein R isoform 4	caspase-3	DYHDYRGG
gi|11386203|ref|NP_000380.1| cyclin-dependent kinase inhibitor 1A	caspase-3	DHVDLSLS
gi|11386203|ref|NP_000380.1| cyclin-dependent kinase inhibitor 1A	caspase-3	SMTDFYHS
gi|21536421|ref|NP_647478.1| livin inhibitor of apoptosis isoform alpha	caspase-3	DHVDGQIL
gi|21536421|ref|NP_647478.1| livin inhibitor of apoptosis isoform alpha	caspase-7	DHVDGQIL
gi|187608571|ref|NP_003870.4| CASP8 and FADD-like apoptosis regulator isoform 1	caspase-8	LEVDGPAM
gi|194595509|ref|NP_001123910.1| spectrin alpha non-erythrocytic 1 (alpha-fodrin) isoform 1	caspase-3	DETDSKTA
gi|194595509|ref|NP_001123910.1| spectrin alpha non-erythrocytic 1 (alpha-fodrin) isoform 1	caspase-3	DSLDSVEA
gi|194595509|ref|NP_001123910.1| spectrin alpha non-erythrocytic 1 (alpha-fodrin) isoform 1	caspase-1	DETDSKTA
gi|194595509|ref|NP_001123910.1| spectrin alpha non-erythrocytic 1 (alpha-fodrin) isoform 1	caspase-4	DETDSKTA
gi|14790124|ref|NP_001220.2| caspase 9 isoform alpha preproprotein	caspase-3	DQLDAISS
gi|14790124|ref|NP_001220.2| caspase 9 isoform alpha preproprotein	caspase-9	PEPDATPF
gi|62241015|ref|NP_001014432.1| v-akt murine thymoma viral oncogene homolog 1	caspase-3	TVADGLKK
gi|62241015|ref|NP_001014432.1| v-akt murine thymoma viral oncogene homolog 1	caspase-3	EEMDFRSG
gi|62241015|ref|NP_001014432.1| v-akt murine thymoma viral oncogene homolog 1	caspase-3	ECVDSERR
gi|149158696|ref|NP_001092004.1| HLA-B associated transcript-3 isoform b	caspase-3	DEQDGASA
gi|5031875|ref|NP_005563.1| lamin A/C isoform 2	caspase-6	VEIDNGKQ
gi|4557625|ref|NP_001489.1| glutamate-cysteine ligase catalytic subunit	caspase-3	AVVDGCGK
gi|6552301|ref|NP_009226.1| breast cancer 1 early onset isoform 1	caspase-3	DLLDDGEI
gi|6552301|ref|NP_009226.1| breast cancer 1 early onset isoform 1	caspase-3	DVLDVLNE
gi|38044288|ref|NP_937895.1| gelsolin isoform b	caspase-3	DQTDGLGL
gi|40354195|ref|NP_954657.1| keratin 18	caspase-3	VEVDAPKS
gi|40354195|ref|NP_954657.1| keratin 18	caspase-7	EVDAPKSQ
gi|40354195|ref|NP_954657.1| keratin 18	caspase-6	EVDAPKSQ
gi|15718700|ref|NP_203125.1| caspase 7 isoform alpha	caspase-3	DSVDAKPD
gi|15718700|ref|NP_203125.1| caspase 7 isoform alpha	caspase-7	DSVDAKPD
gi|15718700|ref|NP_203125.1| caspase 7 isoform alpha	caspase-6	IQADSGPI
gi|15718700|ref|NP_203125.1| caspase 7 isoform alpha	caspase-6	NDTDANPR
gi|22027616|ref|NP_003291.2| TNF receptor-associated factor 3 isoform 1	caspase-3	EEADSMKS

gi|22027616|ref|NP_003291.2| TNF receptor-associated factor 3 isoform 1	caspase-3	ESVDKSAG
gi|4758476|ref|NP_004801.1| GRB2-related adaptor protein 2	caspase-3	DINDGHCG
gi|38201625|ref|NP_937885.1| eukaryotic translation initiation factor 4 gamma 1 isoform 3	caspase-3	DRLDRART
gi|38201625|ref|NP_937885.1| eukaryotic translation initiation factor 4 gamma 1 isoform 3	caspase-3	DLLDAFKE
gi|188595716|ref|NP_001120972.1| met proto-oncogene isoform a precursor	caspase-3	ESVDYRAT
gi|162287326|ref|NP_001104567.1| Yamaguchi sarcoma viral (v-yes-1) oncogene homolog isoform B	caspase-3	DGVDLKTQ
gi|52486327|ref|NP_002735.3| protein kinase C zeta isoform 1	caspase-3	EETDGIAY
gi|52486327|ref|NP_002735.3| protein kinase C zeta isoform 1	caspase-3	DGMDGIKI
gi|52486327|ref|NP_002735.3| protein kinase C zeta isoform 1	caspase-7	EETDGIAY
gi|52486327|ref|NP_002735.3| protein kinase C zeta isoform 1	caspase-7	DSEDLKPV
gi|52486327|ref|NP_002735.3| protein kinase C zeta isoform 1	caspase-6	EETDGIAY
gi|52486327|ref|NP_002735.3| protein kinase C zeta isoform 1	caspase-8	EETDGIAY
gi|187608577|ref|NP_001120655.1| CASP8 and FADD-like apoptosis regulator isoform 1	caspase-8	LEVDGPAM
gi|4501887|ref|NP_001605.1| actin gamma 1 propeptide	caspase-1	ELPDGQVI
gi|4501887|ref|NP_001605.1| actin gamma 1 propeptide	caspase-3	ELPDGQVI
gi|187173288|ref|NP_003274.3| troponin T1 skeletal slow isoform a	caspase-3	VDFDDIHR
gi|72198189|ref|NP_000624.2| B-cell lymphoma protein 2 alpha isoform	caspase-3	DAGDVGAA
gi|14790128|ref|NP_127463.1| caspase 9 isoform beta preproprotein	caspase-3	DQLDAISS
gi|14790128|ref|NP_127463.1| caspase 9 isoform beta preproprotein	caspase-9	PEPDATPF
gi|187173290|ref|NP_001119604.1| troponin T1 skeletal slow isoform b	caspase-3	VDFDDIHR
gi|4506903|ref|NP_003760.1| splicing factor arginine/serine-rich 9	caspase-3	DLKDHMRE
gi|4506903|ref|NP_003760.1| splicing factor arginine/serine-rich 9	caspase-3	GWADERGG
gi|187173292|ref|NP_001119605.1| troponin T1 skeletal slow isoform c	caspase-3	VDFDDIHR
gi|4502435|ref|NP_001712.1| BMX non-receptor tyrosine kinase	caspase-3	DFPDWWQV
gi|5453974|ref|NP_006247.1| protein kinase N2	caspase-3	DITDCPRT

gi|5453974|ref|NP_006247.1| protein kinase N2	caspase-3	DEVDSLMC
gi|27436948|ref|NP_733822.1| lamin A/C isoform 3	caspase-6	VEIDNGKQ
gi|52486194|ref|NP_003551.2| phospholipase A2 group VI isoform a	caspase-3	DVTDYKGE
gi|20336335|ref|NP_612815.1| BCL2-like 1 isoform 1	caspase-1	HLADSPAV
gi|20336335|ref|NP_612815.1| BCL2-like 1 isoform 1	caspase-3	HLADSPAV
gi|20336335|ref|NP_612815.1| BCL2-like 1 isoform 1	caspase-3	SSLDAREV
gi|15718698|ref|NP_203124.1| caspase 7 isoform delta	caspase-3	DSVDAKPD
gi|15718698|ref|NP_203124.1| caspase 7 isoform delta	caspase-7	DSVDAKPD
gi|15718698|ref|NP_203124.1| caspase 7 isoform delta	caspase-6	IQADSGPI
gi|15718698|ref|NP_203124.1| caspase 7 isoform delta	caspase-6	NDTDANPR
gi|169218268|ref|XP_945884.2| PREDICTED: similar to mutated in ataxia telangiectasia partial	caspase-3	DYPDSSVS
gi|178557736|ref|NP_001116539.1| microtubule-associated protein tau isoform 5	caspase-3	DMVDSPQL
gi|178557736|ref|NP_001116539.1| microtubule-associated protein tau isoform 5	caspase-1	DMVDSPQL
gi|178557736|ref|NP_001116539.1| microtubule-associated protein tau isoform 5	caspase-7	DMVDSPQL
gi|178557736|ref|NP_001116539.1| microtubule-associated protein tau isoform 5	caspase-6	DMVDSPQL
gi|178557736|ref|NP_001116539.1| microtubule-associated protein tau isoform 5	caspase-8	DMVDSPQL
gi|38201627|ref|NP_937887.1| eukaryotic translation initiation factor 4 gamma 1 isoform 2	caspase-3	DRLDRART
gi|38201627|ref|NP_937887.1| eukaryotic translation initiation factor 4 gamma 1 isoform 2	caspase-3	DLLDAFKE
gi|156151394|ref|NP_001095868.1| heterogeneous nuclear ribonucleoprotein R isoform 1	caspase-3	YPPDYYGY
gi|156151394|ref|NP_001095868.1| heterogeneous nuclear ribonucleoprotein R isoform 1	caspase-3	DYYDDYYG
gi|156151394|ref|NP_001095868.1| heterogeneous nuclear ribonucleoprotein R isoform 1	caspase-3	DYHDYRGG
gi|156151394|ref|NP_001095868.1| heterogeneous nuclear ribonucleoprotein R isoform 1	caspase-3	RAIDALRE
gi|156151394|ref|NP_001095868.1| heterogeneous nuclear ribonucleoprotein R isoform 1	caspase-3	KESDLSHV
gi|4557757|ref|NP_000240.1| MutL protein homolog 1	caspase-3	DKTDISSG
gi|4557365|ref|NP_000048.1| Bloom syndrome protein	caspase-3	TEVDFNKS
gi|23510362|ref|NP_694592.1| protein-tyrosine kinase fyn isoform b	caspase-3	EERDGSLN
gi|112382250|ref|NP_003119.2| spectrin beta non-erythrocytic 1 isoform 1	caspase-3	DEVDSKRL
gi|112382250|ref|NP_003119.2| spectrin beta non-erythrocytic 1 isoform 1	caspase-3	ETVDTSEM
gi|13654237|ref|NP_008835.5| protein kinase DNA-activated catalytic polypeptide isoform 1	caspase-3	DEVDNKVK
gi|113431257|ref|XP_499153.3| PREDICTED: hypothetical protein	caspase-3	DDVDTKKQ

gi|113431257|ref|XP_499153.3| PREDICTED: hypothetical protein	caspase-3	DDEDDDVD
gi|113431257|ref|XP_499153.3| PREDICTED: hypothetical protein	caspase-3	DEDDDVDT
gi|67782338|ref|NP_001019978.1| amyloid precursor-like protein 1 isoform 1 precursor	caspase-3	VEVDPMLT
gi|52486251|ref|NP_001004426.1| phospholipase A2 group VI isoform b	caspase-3	DVTDYKGE
gi|4503539|ref|NP_001409.1| eukaryotic translation initiation factor 4 gamma 2 isoform 1	caspase-3	DETDSSSA
gi|23510364|ref|NP_694593.1| protein-tyrosine kinase fyn isoform c	caspase-3	EERDGSLN
gi|117189975|ref|NP_112604.2| heterogeneous nuclear ribonucleoprotein C isoform a	caspase-3	NKTDPRSM
gi|117189975|ref|NP_112604.2| heterogeneous nuclear ribonucleoprotein C isoform a	caspase-3	EGEDDRDS
gi|117189975|ref|NP_112604.2| heterogeneous nuclear ribonucleoprotein C isoform a	caspase-3	GEDDRDSA
gi|117189975|ref|NP_112604.2| heterogeneous nuclear ribonucleoprotein C isoform a	caspase-3	DDRDSANG
gi|31543385|ref|NP_005008.2| phosphate cytidylyltransferase 1 choline alpha	caspase-6	TEEDGVPS
gi|31543385|ref|NP_005008.2| phosphate cytidylyltransferase 1 choline alpha	caspase-8	TEEDGVPS
gi|31543385|ref|NP_005008.2| phosphate cytidylyltransferase 1 choline alpha	caspase-9	TEEDGVPS
gi|63055057|ref|NP_001017992.1| actin beta-like 2	caspase-1	LVVDNGSG
gi|63055057|ref|NP_001017992.1| actin beta-like 2	caspase-1	ELPDGQVI
gi|63055057|ref|NP_001017992.1| actin beta-like 2	caspase-3	ELPDGQVI
gi|38201621|ref|NP_886553.2| eukaryotic translation initiation factor 4 gamma 1 isoform 1	caspase-3	DRLDRART
gi|38201621|ref|NP_886553.2| eukaryotic translation initiation factor 4 gamma 1 isoform 1	caspase-3	DLLDAFKE
gi|15431332|ref|NP_150636.1| caspase 1 isoform delta	caspase-1	AVQDNPAM
gi|14916485|ref|NP_116787.1| caspase 6 isoform beta	caspase-3	DVVDNQTE
gi|14916485|ref|NP_116787.1| caspase 6 isoform beta	caspase-3	TEVDAASV
gi|4501883|ref|NP_001604.1| alpha 2 actin	caspase-1	ELPDGQVI
gi|4501883|ref|NP_001604.1| alpha 2 actin	caspase-3	ELPDGQVI
gi|47132589|ref|NP_002732.3| protein kinase N1 isoform 2	caspase-3	ATHDGPQS
gi|47132589|ref|NP_002732.3| protein kinase N1 isoform 2	caspase-3	DFLDNERH
gi|47132589|ref|NP_002732.3| protein kinase N1 isoform 2	caspase-3	LGTDSDSS
gi|47132589|ref|NP_002732.3| protein kinase N1 isoform 2	caspase-3	TDSDSSPQ
gi|75709226|ref|NP_001028753.1| protein kinase C zeta isoform 2	caspase-3	EETDGIAY

gi|75709226|ref|NP_001028753.1| protein kinase C zeta isoform 2	caspase-3	DGMDGIKI
gi|75709226|ref|NP_001028753.1| protein kinase C zeta isoform 2	caspase-7	EETDGIAY
gi|75709226|ref|NP_001028753.1| protein kinase C zeta isoform 2	caspase-7	DSEDLKPV
gi|75709226|ref|NP_001028753.1| protein kinase C zeta isoform 2	caspase-6	EETDGIAY
gi|75709226|ref|NP_001028753.1| protein kinase C zeta isoform 2	caspase-8	EETDGIAY
gi|4504165|ref|NP_000168.1| gelsolin isoform a precursor	caspase-3	DQTDGLGL
gi|10092619|ref|NP_065390.1| nuclear factor of kappa light polypeptide gene enhancer in B-cells inhibitor alpha	caspase-3	DRHDSGLD
gi|112382252|ref|NP_842565.2| spectrin beta non-erythrocytic 1 isoform 2	caspase-3	DEVDSKRL
gi|37595565|ref|NP_932170.1| T-cell receptor zeta chain isoform 1 precursor	caspase-3	DTYDALHM
gi|37595565|ref|NP_932170.1| T-cell receptor zeta chain isoform 1 precursor	caspase-3	GLLDPKLC
gi|37595565|ref|NP_932170.1| T-cell receptor zeta chain isoform 1 precursor	caspase-3	YLLDGILF
gi|46430499|ref|NP_068810.2| v-rel reticuloendotheliosis viral oncogene homolog A	caspase-3	DCRDGFYE
gi|46430499|ref|NP_068810.2| v-rel reticuloendotheliosis viral oncogene homolog A	caspase-6	VFTDLASV
gi|110630018|ref|NP_001036024.1| eukaryotic translation initiation factor 4 gamma 2 isoform 2	caspase-3	DETDSSSA
gi|156151396|ref|NP_001095869.1| heterogeneous nuclear ribonucleoprotein R isoform 3	caspase-3	YPPDYYGY
gi|156151396|ref|NP_001095869.1| heterogeneous nuclear ribonucleoprotein R isoform 3	caspase-3	DYYDDYYG
gi|156151396|ref|NP_001095869.1| heterogeneous nuclear ribonucleoprotein R isoform 3	caspase-3	DYHDYRGG
gi|4885583|ref|NP_005397.1| Rho-associated coiled-coil containing protein kinase 1	caspase-3	DETDGNLP
gi|117190174|ref|NP_004491.2| heterogeneous nuclear ribonucleoprotein C isoform b	caspase-3	NKTDPRSM
gi|117190174|ref|NP_004491.2| heterogeneous nuclear ribonucleoprotein C isoform b	caspase-3	EGEDDRDS
gi|117190174|ref|NP_004491.2| heterogeneous nuclear ribonucleoprotein C isoform b	caspase-3	GEDDRDSA
gi|117190174|ref|NP_004491.2| heterogeneous nuclear ribonucleoprotein C isoform b	caspase-3	DDRDSANG
gi|178557734|ref|NP_001116538.1| microtubule-associated protein tau isoform 6	caspase-3	GSSDPLIQ
gi|178557734|ref|NP_001116538.1| microtubule-associated protein tau isoform 6	caspase-3	DMVDSPQL

gi|178557734|ref|NP_001116538.1| microtubule-associated protein tau isoform 6	caspase-1	DMVDSPQL
gi|178557734|ref|NP_001116538.1| microtubule-associated protein tau isoform 6	caspase-7	GSSDPLIQ
gi|178557734|ref|NP_001116538.1| microtubule-associated protein tau isoform 6	caspase-7	DMVDSPQL
gi|178557734|ref|NP_001116538.1| microtubule-associated protein tau isoform 6	caspase-6	DMVDSPQL
gi|178557734|ref|NP_001116538.1| microtubule-associated protein tau isoform 6	caspase-8	GSSDPLIQ
gi|178557734|ref|NP_001116538.1| microtubule-associated protein tau isoform 6	caspase-8	DMVDSPQL
gi|6715568|ref|NP_000935.1| protein phosphatase 3 catalytic subunit alpha isoform isoform 1	caspase-3	DGFDGATA
gi|6715568|ref|NP_000935.1| protein phosphatase 3 catalytic subunit alpha isoform isoform 1	caspase-7	DGFDGATA
gi|4506381|ref|NP_002863.1| ras-related C3 botulinum toxin substrate 2	caspase-3	DLRDDKDT
gi|22027651|ref|NP_001118.2| adaptor-related protein complex 1 beta 1 subunit isoform a	caspase-3	DLFDLTSG
gi|37595537|ref|NP_079402.2| ring finger protein 34 isoform 2	caspase-3	DEDDDDEE
gi|19923653|ref|NP_150091.2| PC4 and SFRS1 interacting protein 1 isoform 2	caspase-3	EVPDGAVK
gi|19923653|ref|NP_150091.2| PC4 and SFRS1 interacting protein 1 isoform 2	caspase-3	DAQDGNQP
gi|19923653|ref|NP_150091.2| PC4 and SFRS1 interacting protein 1 isoform 2	caspase-3	WEIDNNPK
gi|19923653|ref|NP_150091.2| PC4 and SFRS1 interacting protein 1 isoform 2	caspase-7	EVPDGAVK
gi|19923653|ref|NP_150091.2| PC4 and SFRS1 interacting protein 1 isoform 2	caspase-7	DAQDGNQP
gi|19923653|ref|NP_150091.2| PC4 and SFRS1 interacting protein 1 isoform 2	caspase-7	WEIDNNPK
gi|194688147|ref|NP_001124163.1| protein phosphatase 3 catalytic subunit alpha isoform isoform 2	caspase-3	DGFDGATA
gi|194688147|ref|NP_001124163.1| protein phosphatase 3 catalytic subunit alpha isoform isoform 2	caspase-7	DGFDGATA
gi|4505903|ref|NP_002666.1| promyelocytic leukemia protein isoform 6	caspase-3	PHLDGPPS
gi|4505815|ref|NP_003548.1| phosphatidylinositol-4-phosphate 5-kinase type I alpha	caspase-3	DIPDGLFL
gi|4502573|ref|NP_001214.1| caspase 1 isoform beta precursor	caspase-1	AVQDNPAM
gi|4502573|ref|NP_001214.1| caspase 1 isoform beta precursor	caspase-1	WFKDSVGV
gi|4502573|ref|NP_001214.1| caspase 1 isoform beta precursor	caspase-1	FEDDAIKK

gi|38201619|ref|NP_004944.2| eukaryotic translation initiation factor 4 gamma 1 isoform 4	caspase-3	DRLDRART
gi|38201619|ref|NP_004944.2| eukaryotic translation initiation factor 4 gamma 1 isoform 4	caspase-3	DLLDAFKE
gi|20631958|ref|NP_620116.1| BCL2-associated X protein isoform alpha	caspase-3	FIQDRAGR
gi|194688149|ref|NP_001124164.1| protein phosphatase 3 catalytic subunit alpha isoform isoform 3	caspase-3	DGFDGATA
gi|194688149|ref|NP_001124164.1| protein phosphatase 3 catalytic subunit alpha isoform isoform 3	caspase-7	DGFDGATA
gi|109637753|ref|NP_001741.4| calpastatin isoform a	caspase-1	ALDDLIDT
gi|109637753|ref|NP_001741.4| calpastatin isoform a	caspase-1	LSSDFTCG
gi|109637753|ref|NP_001741.4| calpastatin isoform a	caspase-1	ALADSLGK
gi|109637753|ref|NP_001741.4| calpastatin isoform a	caspase-3	LSSDFTCG
gi|47132591|ref|NP_998725.1| protein kinase N1 isoform 1	caspase-3	ATHDGPQS
gi|47132591|ref|NP_998725.1| protein kinase N1 isoform 1	caspase-3	DFLDNERH
gi|47132591|ref|NP_998725.1| protein kinase N1 isoform 1	caspase-3	LGTDSDSS
gi|47132591|ref|NP_998725.1| protein kinase N1 isoform 1	caspase-3	TDSDSSPQ
gi|4758148|ref|NP_004392.1| DNA fragmentation factor 45kDa alpha polypeptide isoform 1	caspase-3	DETDSGAG
gi|4758148|ref|NP_004392.1| DNA fragmentation factor 45kDa alpha polypeptide isoform 1	caspase-3	DAVDTGIS
gi|5032193|ref|NP_005649.1| TNF receptor-associated factor 1	caspase-3	LEVDCYRA
gi|5032193|ref|NP_005649.1| TNF receptor-associated factor 1	caspase-6	LEVDCYRA
gi|5032193|ref|NP_005649.1| TNF receptor-associated factor 1	caspase-8	LEVDCYRA
gi|74136883|ref|NP_114032.2| heterogeneous nuclear ribonucleoprotein U isoform a	caspase-3	SALDGDQM
gi|37595539|ref|NP_919247.1| ring finger protein 34 isoform 1	caspase-3	DEDDDDEE
gi|75709228|ref|NP_001028754.1| protein kinase C zeta isoform 2	caspase-3	EETDGIAY
gi|75709228|ref|NP_001028754.1| protein kinase C zeta isoform 2	caspase-3	DGMDGIKI
gi|75709228|ref|NP_001028754.1| protein kinase C zeta isoform 2	caspase-7	EETDGIAY
gi|75709228|ref|NP_001028754.1| protein kinase C zeta isoform 2	caspase-7	DSEDLKPV
gi|75709228|ref|NP_001028754.1| protein kinase C zeta isoform 2	caspase-6	EETDGIAY
gi|75709228|ref|NP_001028754.1| protein kinase C zeta isoform 2	caspase-8	EETDGIAY
gi|5454096|ref|NP_006273.1| serine/threonine kinase 4	caspase-3	DEMDSGTM

gi|5454096|ref|NP_006273.1| serine/threonine kinase 4	caspase-3	TMTDGANT
gi|4506791|ref|NP_002962.1| special AT-rich sequence binding protein 1	caspase-6	VEMDSLSE
gi|15011880|ref|NP_057626.2| serine/threonine protein kinase MST4 isoform 1	caspase-3	DESDSEGS
gi|24430192|ref|NP_005548.2| keratin 16	caspase-6	VEMDAAPG
gi|24430192|ref|NP_005548.2| keratin 16	caspase-6	VEMDAAPG
gi|190014586|ref|NP_066967.3| PC4 and SFRS1 interacting protein 1 isoform 1	caspase-3	EVPDGAVK
gi|190014586|ref|NP_066967.3| PC4 and SFRS1 interacting protein 1 isoform 1	caspase-3	WEIDNNPK
gi|190014586|ref|NP_066967.3| PC4 and SFRS1 interacting protein 1 isoform 1	caspase-7	EVPDGAVK
gi|190014586|ref|NP_066967.3| PC4 and SFRS1 interacting protein 1 isoform 1	caspase-7	WEIDNNPK
gi|117190192|ref|NP_001070910.1| heterogeneous nuclear ribonucleoprotein C isoform a	caspase-3	NKTDPRSM
gi|117190192|ref|NP_001070910.1| heterogeneous nuclear ribonucleoprotein C isoform a	caspase-3	EGEDDRDS
gi|117190192|ref|NP_001070910.1| heterogeneous nuclear ribonucleoprotein C isoform a	caspase-3	GEDDRDSA
gi|117190192|ref|NP_001070910.1| heterogeneous nuclear ribonucleoprotein C isoform a	caspase-3	DDRDSANG
gi|29826321|ref|NP_054908.2| adducin 1 (alpha) isoform b	caspase-3	DIVDRGST
gi|29826321|ref|NP_054908.2| adducin 1 (alpha) isoform b	caspase-3	DRVDENNP
gi|29826321|ref|NP_054908.2| adducin 1 (alpha) isoform b	caspase-3	DDSDAATF
gi|134152674|ref|NP_001077054.1| rabaptin RAB GTPase binding effector protein 1 isoform 2	caspase-3	DESDFGPL
gi|47132600|ref|NP_998731.1| DNA fragmentation factor 45kDa alpha polypeptide isoform 2	caspase-3	DETDSGAG
gi|47132600|ref|NP_998731.1| DNA fragmentation factor 45kDa alpha polypeptide isoform 2	caspase-3	DAVDTGIS
gi|156523968|ref|NP_001609.2| poly (ADP-ribose) polymerase family member 1	caspase-3	DEVDGVDE
gi|156523968|ref|NP_001609.2| poly (ADP-ribose) polymerase family member 1	caspase-1	DEVDGVDE
gi|9845511|ref|NP_008839.2| ras-related C3 botulinum toxin substrate 1 isoform Rac1	caspase-3	DLRDDKDT
gi|4557431|ref|NP_000725.1| T-cell receptor zeta chain isoform 2 precursor	caspase-3	DTYDALHM
gi|4557431|ref|NP_000725.1| T-cell receptor zeta chain isoform 2 precursor	caspase-3	GLLDPKLC
gi|4557431|ref|NP_000725.1| T-cell receptor zeta chain isoform 2 precursor	caspase-3	YLLDGILF
gi|153791352|ref|NP_001093241.1| prostate ovary testis expressed protein on chromosome 2	caspase-1	ELPDGQVI

gi|153791352|ref|NP_001093241.1| prostate ovary testis expressed protein on chromosome 2	caspase-3	ELPDGQVI
gi|15431328|ref|NP_150634.1| caspase 1 isoform alpha precursor	caspase-1	NMQDSQGV
gi|15431328|ref|NP_150634.1| caspase 1 isoform alpha precursor	caspase-1	AVQDNPAM
gi|15431328|ref|NP_150634.1| caspase 1 isoform alpha precursor	caspase-1	WFKDSVGV
gi|15431328|ref|NP_150634.1| caspase 1 isoform alpha precursor	caspase-1	FEDDAIKK
gi|169164476|ref|XP_001724226.1| PREDICTED: similar to heterogeneous nuclear ribonucleoprotein A1	caspase-3	SYNDFGNY
gi|27765085|ref|NP_775083.1| calpastatin isoform b	caspase-1	ALDDLIDT
gi|27765085|ref|NP_775083.1| calpastatin isoform b	caspase-1	LSSDFTCG
gi|27765085|ref|NP_775083.1| calpastatin isoform b	caspase-1	ALADSLGK
gi|27765085|ref|NP_775083.1| calpastatin isoform b	caspase-3	LSSDFTCG
gi|190014588|ref|NP_001121689.1| PC4 and SFRS1 interacting protein 1 isoform 2	caspase-3	EVPDGAVK
gi|190014588|ref|NP_001121689.1| PC4 and SFRS1 interacting protein 1 isoform 2	caspase-3	DAQDGNQP
gi|190014588|ref|NP_001121689.1| PC4 and SFRS1 interacting protein 1 isoform 2	caspase-3	WEIDNNPK
gi|190014588|ref|NP_001121689.1| PC4 and SFRS1 interacting protein 1 isoform 2	caspase-7	EVPDGAVK
gi|190014588|ref|NP_001121689.1| PC4 and SFRS1 interacting protein 1 isoform 2	caspase-7	DAQDGNQP
gi|190014588|ref|NP_001121689.1| PC4 and SFRS1 interacting protein 1 isoform 2	caspase-7	WEIDNNPK
gi|20070158|ref|NP_003567.2| serine/threonine kinase 24 isoform a	caspase-3	AETDGQAS
gi|20070158|ref|NP_003567.2| serine/threonine kinase 24 isoform a	caspase-7	AETDGQAS
gi|20070158|ref|NP_003567.2| serine/threonine kinase 24 isoform a	caspase-8	AETDGQAS
gi|196114999|ref|NP_001124482.1| special AT-rich sequence binding protein 1	caspase-6	VEMDSLSE
gi|117190254|ref|NP_001070911.1| heterogeneous nuclear ribonucleoprotein C isoform b	caspase-3	NKTDPRSM
gi|117190254|ref|NP_001070911.1| heterogeneous nuclear ribonucleoprotein C isoform b	caspase-3	EGEDDRDS
gi|117190254|ref|NP_001070911.1| heterogeneous nuclear ribonucleoprotein C isoform b	caspase-3	GEDDRDSA
gi|117190254|ref|NP_001070911.1| heterogeneous nuclear ribonucleoprotein C isoform b	caspase-3	DDRDSANG
gi|29826323|ref|NP_054909.2| adducin 1 (alpha) isoform c	caspase-3	DIVDRGST
gi|29826323|ref|NP_054909.2| adducin 1 (alpha) isoform c	caspase-3	DRVDENNP

gi|4504445|ref|NP_002127.1| heterogeneous nuclear ribonucleoprotein A1 isoform a	caspase-3	SYNDFGNY
gi|115430252|ref|NP_037365.2| serine threonine kinase 39 (STE20/SPS1 homolog yeast)	caspase-3	DEMDEKSE
gi|70610136|ref|NP_003622.2| poly (ADP-ribose) glycohydrolase	caspase-3	DEIDVVPE
gi|70610136|ref|NP_003622.2| poly (ADP-ribose) glycohydrolase	caspase-3	MDVDNSKN
gi|58761498|ref|NP_001011725.1| heterogeneous nuclear ribonucleoprotein A1-like	caspase-3	SYNDFGNY
gi|103471999|ref|NP_006272.2| serine/threonine kinase 3 (STE20 homolog yeast)	caspase-3	DELDSHTM
gi|27886593|ref|NP_005598.3| PTK2 protein tyrosine kinase 2 isoform b	caspase-7	DQTDSWNH
gi|27886593|ref|NP_005598.3| PTK2 protein tyrosine kinase 2 isoform b	caspase-6	VSWDSGGS
gi|27886593|ref|NP_005598.3| PTK2 protein tyrosine kinase 2 isoform b	caspase-3	DQTDSWNH
gi|14141161|ref|NP_004492.2| heterogeneous nuclear ribonucleoprotein U isoform b	caspase-3	SALDGDQM
gi|110431348|ref|NP_005206.2| deleted in colorectal carcinoma	caspase-3	LSVDRGFG
gi|4557701|ref|NP_000413.1| keratin 17	caspase-6	VEMDAAPG
gi|4557701|ref|NP_000413.1| keratin 17	caspase-6	VEMDAAPG
gi|110349738|ref|NP_001027467.2| serine/threonine kinase 24 isoform b	caspase-3	AETDGQAS
gi|110349738|ref|NP_001027467.2| serine/threonine kinase 24 isoform b	caspase-7	AETDGQAS
gi|110349738|ref|NP_001027467.2| serine/threonine kinase 24 isoform b	caspase-8	AETDGQAS
gi|148763347|ref|NP_076916.2| cell division cycle 2-like 2 isoform 1	caspase-3	YVPDSPAL
gi|148763347|ref|NP_076916.2| cell division cycle 2-like 2 isoform 1	caspase-1	YVPDSPAL
gi|18105007|ref|NP_004332.2| carbamoylphosphate synthetase 2/aspartate transcarbamylase/dihydroorotase	caspase-3	VASDGVVA
gi|18105007|ref|NP_004332.2| carbamoylphosphate synthetase 2/aspartate transcarbamylase/dihydroorotase	caspase-3	EAVDGECP
gi|27765087|ref|NP_775084.1| calpastatin isoform c	caspase-1	ALADSLGK
gi|189083772|ref|NP_001121134.1| gelsolin isoform b	caspase-3	DQTDGLGL
gi|189083776|ref|NP_001121136.1| gelsolin isoform b	caspase-3	DQTDGLGL
gi|47607492|ref|NP_000436.2| plectin 1 isoform 1	caspase-8	ILRDKDNT
gi|89993689|ref|NP_002383.2| mouse double minute 2 homolog isoform MDM2	caspase-3	DVPDCKKT
gi|89993689|ref|NP_002383.2| mouse double minute 2 homolog isoform MDM2	caspase-7	DVPDCKKT
gi|89993689|ref|NP_002383.2| mouse double minute 2 homolog isoform MDM2	caspase-6	DVPDCKKT

gi|72198346|ref|NP_000648.2| B-cell lymphoma protein 2 beta isoform	caspase-3	DAGDVGAA
gi|189083778|ref|NP_001121137.1| gelsolin isoform b	caspase-3	DQTDGLGL
gi|100913206|ref|NP_001348.2| DEAH (Asp-Glu-Ala-His) box polypeptide 9	caspase-3	EEVDLNAG
gi|10835069|ref|NP_004313.1| BCL2-associated agonist of cell death	caspase-3	EQEDSSSA
gi|77539055|ref|NP_001029102.1| ubiquitin and ribosomal protein L40 precursor	ubiquitin-specific peptidase 4	LRGGIIEP
gi|77539055|ref|NP_001029102.1| ubiquitin and ribosomal protein L40 precursor	ubiquitin-specific peptidase 4	LRGGIIEP
gi|67191208|ref|NP_066289.2| ubiquitin C	ubiquitin-specific peptidase 4	LRGGMQIF
gi|67191208|ref|NP_066289.2| ubiquitin C	ubiquitin-specific peptidase 15	LRGGMQIF
gi|67191208|ref|NP_066289.2| ubiquitin C	ubiquitin-specific peptidase 5	LRGGMQIF
gi|4507761|ref|NP_003324.1| ubiquitin and ribosomal protein L40 precursor	ubiquitin-specific peptidase 4	LRGGIIEP
gi|4507761|ref|NP_003324.1| ubiquitin and ribosomal protein L40 precursor	ubiquitin-specific peptidase 4	LRGGIIEP
gi|11024714|ref|NP_061828.1| ubiquitin B precursor	ubiquitin-specific peptidase 4	LRGGMQIF
gi|11024714|ref|NP_061828.1| ubiquitin B precursor	ubiquitin-specific peptidase 15	LRGGMQIF
gi|11024714|ref|NP_061828.1| ubiquitin B precursor	ubiquitin-specific peptidase 5	LRGGMQIF
gi|32967254|ref|NP_861546.1| neurofibromin 2 isoform 2	calpain-2	LILQLCIG
gi|32967254|ref|NP_861546.1| neurofibromin 2 isoform 2	calpain-2	RVNKLILQ
gi|157389005|ref|NP_001739.2| calpain 2 large subunit	calpain-2	AKLAKDRE
gi|157389005|ref|NP_001739.2| calpain 2 large subunit	calpain-2	EGLGSHER
gi|67782319|ref|NP_000338.3| spectrin beta isoform b	calpain-1	EKSTASWA
gi|67782319|ref|NP_000338.3| spectrin beta isoform b	calpain-1	TASWAERF
gi|67782321|ref|NP_001020029.1| spectrin beta isoform a	calpain-1	EKSTASWA
gi|67782321|ref|NP_001020029.1| spectrin beta isoform a	calpain-1	TASWAERF
gi|4757838|ref|NP_004315.1| BCL2-associated X protein isoform beta	calpain-1	LLLQGFIQ
gi|32967514|ref|NP_861969.1| neurofibromin 2 isoform 7	calpain-2	LILQLCIG
gi|32967514|ref|NP_861969.1| neurofibromin 2 isoform 7	calpain-2	RVNKLILQ
gi|154759259|ref|NP_003118.2| spectrin alpha non-erythrocytic 1 (alpha-fodrin) isoform 2	calpain-1	QEVYGMMP
gi|4557671|ref|NP_000198.1| proinsulin precursor	calpain-1	HLVEALYL
gi|87578394|ref|NP_001034627.1| microtubule-associated protein 2 isoform 5	calpain-2	PDLKNVKS
gi|87578394|ref|NP_001034627.1| microtubule-associated protein 2 isoform 5	calpain-2	KNVKSKIG
gi|32967260|ref|NP_861966.1| neurofibromin 2 isoform 5	calpain-2	LILQLCIG

gi|32967260|ref|NP_861966.1| neurofibromin 2 isoform 5	calpain-2	RVNKLILQ
gi|68509940|ref|NP_001020272.1| Golli-mbp isoform 1	calpain-1	VTPRTPPP
gi|68509940|ref|NP_001020272.1| Golli-mbp isoform 1	calpain-1	KNIVTPRT
gi|68509940|ref|NP_001020272.1| Golli-mbp isoform 1	calpain-2	VTPRTPPP
gi|68509940|ref|NP_001020272.1| Golli-mbp isoform 1	calpain-2	KNIVTPRT
gi|4503063|ref|NP_000487.1| crystallin beta B2	calpain-1	HQTQAGKP
gi|20631973|ref|NP_620120.1| BCL2-associated X protein isoform sigma	calpain-1	LLLQGFIQ
gi|89191865|ref|NP_000202.2| integrin beta 2 precursor	calpain-2	EKLKSQWN
gi|89191865|ref|NP_000202.2| integrin beta 2 precursor	calpain-2	TTVMNPKF
gi|89191865|ref|NP_000202.2| integrin beta 2 precursor	calpain-2	NPLFKSAT
gi|32967264|ref|NP_861968.1| neurofibromin 2 isoform 7	calpain-2	LILQLCIG
gi|32967264|ref|NP_861968.1| neurofibromin 2 isoform 7	calpain-2	RVNKLILQ
gi|62243290|ref|NP_001543.2| insulin-like growth factor binding protein 4 precursor	calpain-1	MKVNGAPR
gi|62243290|ref|NP_001543.2| insulin-like growth factor binding protein 4 precursor	calpain-1	AEIEAIQE
gi|62243290|ref|NP_001543.2| insulin-like growth factor binding protein 4 precursor	calpain-1	QKHFAKIR
gi|188595677|ref|NP_001120963.1| integrin beta 2 precursor	calpain-2	EKLKSQWN
gi|188595677|ref|NP_001120963.1| integrin beta 2 precursor	calpain-2	TTVMNPKF
gi|188595677|ref|NP_001120963.1| integrin beta 2 precursor	calpain-2	NPLFKSAT
gi|4502739|ref|NP_003927.1| cyclin-dependent kinase 5 regulatory subunit 2	calpain-2	QQRNRENL
gi|4502739|ref|NP_003927.1| cyclin-dependent kinase 5 regulatory subunit 2	calpain-2	LRKGRDPP
gi|4502739|ref|NP_003927.1| cyclin-dependent kinase 5 regulatory subunit 2	calpain-2	ENLLRKGR
gi|82534351|ref|NP_058519.2| microtubule-associated protein tau isoform 1	calpain-2	AVVRTPPK
gi|82534351|ref|NP_058519.2| microtubule-associated protein tau isoform 1	calpain-2	AGLKESPL
gi|82534351|ref|NP_058519.2| microtubule-associated protein tau isoform 1	calpain-2	PDLKNVKS
gi|82534351|ref|NP_058519.2| microtubule-associated protein tau isoform 1	calpain-2	KNVKSKIG
gi|82534351|ref|NP_058519.2| microtubule-associated protein tau isoform 1	calpain-2	ENLKHQPG
gi|82534351|ref|NP_058519.2| microtubule-associated protein tau isoform 1	calpain-2	VEVKSEKL
gi|82534351|ref|NP_058519.2| microtubule-associated protein tau isoform 1	calpain-2	FEVMEDHA
gi|82534351|ref|NP_058519.2| microtubule-associated protein tau isoform 1	calpain-2	QIVYKPVD
gi|82534351|ref|NP_058519.2| microtubule-associated protein tau isoform 1	calpain-2	EIVYKSPV

gi|20631967|ref|NP_620119.1| BCL2-associated X protein isoform epsilon	calpain-1	LLLQGFIQ
gi|48255955|ref|NP_001001344.1| plasma membrane calcium ATPase 3 isoform 3b	calpain-1	RELRRGQI
gi|48255955|ref|NP_001001344.1| plasma membrane calcium ATPase 3 isoform 3b	calpain-1	RGLNRIQT
gi|156231037|ref|NP_001095886.1| kininogen 1 isoform 1	calpain-1	SPFRSSRI
gi|156231037|ref|NP_001095886.1| kininogen 1 isoform 1	calpain-1	ISLMKRPP
gi|156231037|ref|NP_001095886.1| kininogen 1 isoform 1	calpain-2	SPFRSSRI
gi|156231037|ref|NP_001095886.1| kininogen 1 isoform 1	calpain-2	ISLMKRPP
gi|4557361|ref|NP_001187.1| BH3 interacting domain death agonist isoform 2	calpain-1	SRLGRIEA
gi|4557361|ref|NP_001187.1| BH3 interacting domain death agonist isoform 2	calpain-1	WEGYDELQ
gi|90903231|ref|NP_002102.4| huntingtin	calpain-1	SHSSSQVS
gi|90903231|ref|NP_002102.4| huntingtin	calpain-1	SALTASVK
gi|90903231|ref|NP_002102.4| huntingtin	calpain-2	SHSSSQVS
gi|90903231|ref|NP_002102.4| huntingtin	calpain-2	SALTASVK
gi|12083581|ref|NP_056007.1| phosphoinositide-specific phospholipase C beta 1 isoform a	calpain-1	QALHSQPA
gi|48255945|ref|NP_001673.2| plasma membrane calcium ATPase 1 isoform 1b	calpain-1	RELRRGQI
gi|48255945|ref|NP_001673.2| plasma membrane calcium ATPase 1 isoform 1b	calpain-1	RGLNRIQT
gi|37574726|ref|NP_932070.1| BH3 interacting domain death agonist isoform 1	calpain-1	SRLGRIEA
gi|37574726|ref|NP_932070.1| BH3 interacting domain death agonist isoform 1	calpain-1	WEGYDELQ
gi|4502381|ref|NP_001182.1| BCL2-like 1 isoform 2	calpain-2	WHLADSPA
gi|4502381|ref|NP_001182.1| BCL2-like 1 isoform 2	calpain-1	EGTESEME
gi|48255951|ref|NP_001001331.1| plasma membrane calcium ATPase 2 isoform 1	calpain-1	RELRRGQI
gi|48255951|ref|NP_001001331.1| plasma membrane calcium ATPase 2 isoform 1	calpain-1	RGLNRIQT
gi|48255949|ref|NP_001674.2| plasma membrane calcium ATPase 2 isoform 2	calpain-1	RELRRGQI
gi|48255949|ref|NP_001674.2| plasma membrane calcium ATPase 2 isoform 2	calpain-1	RGLNRIQT
gi|20357552|ref|NP_005222.2| cortactin isoform a	calpain-2	ENLAKEKE
gi|20357552|ref|NP_005222.2| cortactin isoform a	calpain-2	SNIRANFE
gi|20357552|ref|NP_005222.2| cortactin isoform a	calpain-2	AYQKTVPV
gi|20357552|ref|NP_005222.2| cortactin isoform a	calpain-2	VTSKTSNI
gi|20357556|ref|NP_612632.1| cortactin isoform b	calpain-2	ENLAKEKE
gi|20357556|ref|NP_612632.1| cortactin isoform b	calpain-2	SNIRANFE
gi|20357556|ref|NP_612632.1| cortactin isoform b	calpain-2	AYQKTVPV
gi|20357556|ref|NP_612632.1| cortactin isoform b	calpain-2	VTSKTSNI
gi|47078292|ref|NP_000203.2| integrin beta chain beta 3 precursor	calpain-1	KWDTANNP

gi|47078292|ref|NP_000203.2| integrin beta chain beta 3 precursor	calpain-1	EERARAKW
gi|47078292|ref|NP_000203.2| integrin beta chain beta 3 precursor	calpain-1	TSTFTNIT
gi|47078292|ref|NP_000203.2| integrin beta chain beta 3 precursor	calpain-1	KWDTANNP
gi|47078292|ref|NP_000203.2| integrin beta chain beta 3 precursor	calpain-1	NPLYKEAT
gi|8400715|ref|NP_058525.1| microtubule-associated protein tau isoform 4	calpain-2	AVVRTPPK
gi|8400715|ref|NP_058525.1| microtubule-associated protein tau isoform 4	calpain-2	PDLKNVKS
gi|8400715|ref|NP_058525.1| microtubule-associated protein tau isoform 4	calpain-2	KNVKSKIG
gi|8400715|ref|NP_058525.1| microtubule-associated protein tau isoform 4	calpain-2	ENLKHQPG
gi|8400715|ref|NP_058525.1| microtubule-associated protein tau isoform 4	calpain-2	VEVKSEKL
gi|8400715|ref|NP_058525.1| microtubule-associated protein tau isoform 4	calpain-2	FEVMEDHA
gi|8400715|ref|NP_058525.1| microtubule-associated protein tau isoform 4	calpain-2	QIVYKPVD
gi|8400715|ref|NP_058525.1| microtubule-associated protein tau isoform 4	calpain-2	EIVYKSPV
gi|48255959|ref|NP_001001396.1| plasma membrane calcium ATPase 4 isoform 4a	calpain-1	RGLNRIQT
gi|62414289|ref|NP_003371.2| vimentin	calpain-2	VRLRSSVP
gi|62414289|ref|NP_003371.2| vimentin	calpain-2	YSLGSALR
gi|62414289|ref|NP_003371.2| vimentin	calpain-2	YVTTSTRT
gi|62414289|ref|NP_003371.2| vimentin	calpain-2	TRTYSLGS
gi|34577052|ref|NP_005233.3| coagulation factor II (thrombin) receptor-like 1 precursor	calpain-1	ETVFSVDE
gi|34577052|ref|NP_005233.3| coagulation factor II (thrombin) receptor-like 1 precursor	calpain-2	VLTGKLTT
gi|34577052|ref|NP_005233.3| coagulation factor II (thrombin) receptor-like 1 precursor	calpain-2	VDGTSHVT
gi|34577052|ref|NP_005233.3| coagulation factor II (thrombin) receptor-like 1 precursor	calpain-2	VETVFSVD
gi|6754638|ref|NP_005901.2| microtubule-associated protein tau isoform 2	calpain-2	AVVRTPPK
gi|6754638|ref|NP_005901.2| microtubule-associated protein tau isoform 2	calpain-2	AGLKESPL
gi|6754638|ref|NP_005901.2| microtubule-associated protein tau isoform 2	calpain-2	PDLKNVKS
gi|6754638|ref|NP_005901.2| microtubule-associated protein tau isoform 2	calpain-2	KNVKSKIG
gi|6754638|ref|NP_005901.2| microtubule-associated protein tau isoform 2	calpain-2	ENLKHQPG

gi|6754638|ref|NP_005901.2| microtubule-associated protein tau isoform 2	calpain-2	VEVKSEKL
gi|6754638|ref|NP_005901.2| microtubule-associated protein tau isoform 2	calpain-2	FEVMEDHA
gi|6754638|ref|NP_005901.2| microtubule-associated protein tau isoform 2	calpain-2	QIVYKPVD
gi|6754638|ref|NP_005901.2| microtubule-associated protein tau isoform 2	calpain-2	EIVYKSPV
gi|33356544|ref|NP_877398.1| phosphoinositide-specific phospholipase C beta 1 isoform b	calpain-1	QALHSQPA
gi|166362740|ref|NP_001983.2| coagulation factor II receptor precursor	calpain-1	PESKATNA
gi|166362740|ref|NP_001983.2| coagulation factor II receptor precursor	calpain-1	SINKSSPL
gi|87578255|ref|NP_114035.2| microtubule-associated protein 2 isoform 4	calpain-2	PDLKNVKS
gi|87578255|ref|NP_114035.2| microtubule-associated protein 2 isoform 4	calpain-2	KNVKSKIG
gi|48255957|ref|NP_001675.3| plasma membrane calcium ATPase 4 isoform 4b	calpain-1	RGLNRIQT
gi|4758074|ref|NP_004067.1| crystallin beta B3	calpain-2	MAEQHGAP
gi|4758074|ref|NP_004067.1| crystallin beta B3	calpain-2	AEQHGAPE
gi|12408656|ref|NP_005177.2| calpain 1 large subunit	calpain-1	RELGLGRH
gi|12408656|ref|NP_005177.2| calpain 1 large subunit	calpain-1	TGVSAQVQ
gi|4507243|ref|NP_001039.1| somatostatin preproprotein	calpain-1	FWKTFTSC
gi|48255953|ref|NP_068768.2| plasma membrane calcium ATPase 3 isoform 3a	calpain-1	RELRRGQI
gi|48255953|ref|NP_068768.2| plasma membrane calcium ATPase 3 isoform 3a	calpain-1	RGLNRIQT
gi|29725609|ref|NP_005219.2| epidermal growth factor receptor isoform a precursor	calpain-1	KPNGIFKG
gi|29725609|ref|NP_005219.2| epidermal growth factor receptor isoform a precursor	calpain-1	RRLLQERE
gi|29725609|ref|NP_005219.2| epidermal growth factor receptor isoform a precursor	calpain-1	DDTFLPVP
gi|29725609|ref|NP_005219.2| epidermal growth factor receptor isoform a precursor	calpain-1	NSTFDSPA
gi|29725609|ref|NP_005219.2| epidermal growth factor receptor isoform a precursor	calpain-1	QSCPIKED
gi|29725609|ref|NP_005219.2| epidermal growth factor receptor isoform a precursor	calpain-1	LWIPEGEK
gi|29725609|ref|NP_005219.2| epidermal growth factor receptor isoform a precursor	calpain-1	PSTSRTPL
gi|13384594|ref|NP_002730.1| protein kinase C gamma	calpain-1	KRCFFGAS
gi|13384594|ref|NP_002730.1| protein kinase C gamma	calpain-1	GPSSSPIP
gi|13384594|ref|NP_002730.1| protein kinase C gamma	calpain-2	KRCFFGAS
gi|13384594|ref|NP_002730.1| protein kinase C gamma	calpain-2	GPSSSPIP

gi|8400711|ref|NP_058518.1| microtubule-associated protein tau isoform 3	calpain-2	AVVRTPPK
gi|8400711|ref|NP_058518.1| microtubule-associated protein tau isoform 3	calpain-2	PDLKNVKS
gi|8400711|ref|NP_058518.1| microtubule-associated protein tau isoform 3	calpain-2	KNVKSKIG
gi|8400711|ref|NP_058518.1| microtubule-associated protein tau isoform 3	calpain-2	ENLKHQPG
gi|8400711|ref|NP_058518.1| microtubule-associated protein tau isoform 3	calpain-2	VEVKSEKL
gi|8400711|ref|NP_058518.1| microtubule-associated protein tau isoform 3	calpain-2	FEVMEDHA
gi|8400711|ref|NP_058518.1| microtubule-associated protein tau isoform 3	calpain-2	QIVYKPVD
gi|8400711|ref|NP_058518.1| microtubule-associated protein tau isoform 3	calpain-2	EIVYKSPV
gi|116063573|ref|NP_001447.2| filamin A alpha isoform 1	calpain-2	APQYTYAQ
gi|160420317|ref|NP_001104026.1| filamin A alpha isoform 2	calpain-2	APQYTYAQ
gi|4502737|ref|NP_003876.1| cyclin-dependent kinase 5 regulatory subunit 1	calpain-1	LSTFAQPP
gi|4506067|ref|NP_002728.1| protein kinase C alpha	calpain-1	SEDRKQPS
gi|4506067|ref|NP_002728.1| protein kinase C alpha	calpain-1	SEDRKQPS
gi|4506067|ref|NP_002728.1| protein kinase C alpha	calpain-1	EKAKLGPA
gi|4506067|ref|NP_002728.1| protein kinase C alpha	calpain-1	AGNKVISP
gi|4506067|ref|NP_002728.1| protein kinase C alpha	calpain-1	AGNKVISP
gi|4506067|ref|NP_002728.1| protein kinase C alpha	calpain-2	EKAKLGPA
gi|4502557|ref|NP_001735.1| calcium/calmodulin-dependent protein kinase IV	calpain-2	TVCGTPGY
gi|13270473|ref|NP_077722.1| beta-neoendorphin-dynorphin preproprotein	calpain-1	GFLRRIRP
gi|109148522|ref|NP_001035835.1| insulin- insulin-like growth factor 2	calpain-1	HLVEALYL
gi|4504893|ref|NP_000884.1| kininogen 1 isoform 2	calpain-1	SPFRSSRI
gi|4504893|ref|NP_000884.1| kininogen 1 isoform 2	calpain-1	ISLMKRPP
gi|4504893|ref|NP_000884.1| kininogen 1 isoform 2	calpain-2	SPFRSSRI
gi|4504893|ref|NP_000884.1| kininogen 1 isoform 2	calpain-2	ISLMKRPP
gi|48255947|ref|NP_001001323.1| plasma membrane calcium ATPase 1 isoform 1a	calpain-1	RELRRGQI
gi|48255947|ref|NP_001001323.1| plasma membrane calcium ATPase 1 isoform 1a	calpain-1	RGLNRIQT
gi|4504125|ref|NP_000824.1| N-methyl-D-aspartate receptor subunit 2A isoform 1 precursor	calpain-1	GSLFSVPS
gi|169165336|ref|XP_001714623.1| PREDICTED: similar to ATPase Ca++ transporting plasma membrane 2	calpain-1	RELRRGQI
gi|169165336|ref|XP_001714623.1| PREDICTED: similar to ATPase Ca++ transporting plasma membrane 2	calpain-1	RGLNRIQT

gi|194595509|ref|NP_001123910.1| spectrin alpha non-erythrocytic 1 (alpha-fodrin) isoform 1	calpain-1	QEVYGMMP
gi|197313636|ref|NP_001127879.1| N-methyl-D-aspartate receptor subunit 2A isoform 1 precursor	calpain-1	GSLFSVPS
gi|14790124|ref|NP_001220.2| caspase 9 isoform alpha preproprotein	calpain-2	DQLDAISS
gi|14790124|ref|NP_001220.2| caspase 9 isoform alpha preproprotein	calpain-2	RPEIRKPE
gi|68509928|ref|NP_001020261.1| myelin basic protein isoform 3	calpain-1	VTPRTPPP
gi|68509928|ref|NP_001020261.1| myelin basic protein isoform 3	calpain-1	KNIVTPRT
gi|68509928|ref|NP_001020261.1| myelin basic protein isoform 3	calpain-2	VTPRTPPP
gi|68509928|ref|NP_001020261.1| myelin basic protein isoform 3	calpain-2	KNIVTPRT
gi|188528901|ref|NP_001120868.1| syntaxin binding protein 2 isoform b	calpain-2	GGSGTSSR
gi|12056461|ref|NP_005199.2| crystallin beta A3	calpain-1	PTTKMAQT
gi|14790128|ref|NP_127463.1| caspase 9 isoform beta preproprotein	calpain-2	DQLDAISS
gi|14790128|ref|NP_127463.1| caspase 9 isoform beta preproprotein	calpain-2	RPEIRKPE
gi|32967262|ref|NP_861967.1| neurofibromin 2 isoform 6	calpain-2	LILQLCIG
gi|32967262|ref|NP_861967.1| neurofibromin 2 isoform 6	calpain-2	RVNKLILQ
gi|4557795|ref|NP_000259.1| neurofibromin 2 isoform 1	calpain-2	LILQLCIG
gi|4557795|ref|NP_000259.1| neurofibromin 2 isoform 1	calpain-2	RVNKLILQ
gi|20336335|ref|NP_612815.1| BCL2-like 1 isoform 1	calpain-2	WHLADSPA
gi|20336335|ref|NP_612815.1| BCL2-like 1 isoform 1	calpain-1	EGTESEME
gi|178557736|ref|NP_001116539.1| microtubule-associated protein tau isoform 5	calpain-2	AVVRTPPK
gi|178557736|ref|NP_001116539.1| microtubule-associated protein tau isoform 5	calpain-2	AGLKESPL
gi|178557736|ref|NP_001116539.1| microtubule-associated protein tau isoform 5	calpain-2	PDLKNVKS
gi|178557736|ref|NP_001116539.1| microtubule-associated protein tau isoform 5	calpain-2	KNVKSKIG
gi|178557736|ref|NP_001116539.1| microtubule-associated protein tau isoform 5	calpain-2	ENLKHQPG
gi|178557736|ref|NP_001116539.1| microtubule-associated protein tau isoform 5	calpain-2	VEVKSEKL
gi|178557736|ref|NP_001116539.1| microtubule-associated protein tau isoform 5	calpain-2	FEVMEDHA
gi|178557736|ref|NP_001116539.1| microtubule-associated protein tau isoform 5	calpain-2	QIVYKPVD
gi|178557736|ref|NP_001116539.1| microtubule-associated protein tau isoform 5	calpain-2	EIVYKSPV
gi|112382250|ref|NP_003119.2| spectrin beta non-erythrocytic 1 isoform 1	calpain-1	EKSAATWD

gi|4505123|ref|NP_002376.1| myelin basic protein isoform 2	calpain-1	VTPRTPPP
gi|4505123|ref|NP_002376.1| myelin basic protein isoform 2	calpain-1	KNIVTPRT
gi|4505123|ref|NP_002376.1| myelin basic protein isoform 2	calpain-2	VTPRTPPP
gi|4505123|ref|NP_002376.1| myelin basic protein isoform 2	calpain-2	KNIVTPRT
gi|87578392|ref|NP_114033.2| microtubule-associated protein 2 isoform 2	calpain-2	PDLKNVKS
gi|87578392|ref|NP_114033.2| microtubule-associated protein 2 isoform 2	calpain-2	KNVKSKIG
gi|112382252|ref|NP_842565.2| spectrin beta non-erythrocytic 1 isoform 2	calpain-1	EKSAATWD
gi|68509930|ref|NP_001020252.1| myelin basic protein isoform 1	calpain-1	VTPRTPPP
gi|68509930|ref|NP_001020252.1| myelin basic protein isoform 1	calpain-1	KNIVTPRT
gi|68509930|ref|NP_001020252.1| myelin basic protein isoform 1	calpain-2	VTPRTPPP
gi|68509930|ref|NP_001020252.1| myelin basic protein isoform 1	calpain-2	KNIVTPRT
gi|178557734|ref|NP_001116538.1| microtubule-associated protein tau isoform 6	calpain-2	AVVRTPPK
gi|178557734|ref|NP_001116538.1| microtubule-associated protein tau isoform 6	calpain-2	AGLKESPL
gi|178557734|ref|NP_001116538.1| microtubule-associated protein tau isoform 6	calpain-2	PDLKNVKS
gi|178557734|ref|NP_001116538.1| microtubule-associated protein tau isoform 6	calpain-2	KNVKSKIG
gi|178557734|ref|NP_001116538.1| microtubule-associated protein tau isoform 6	calpain-2	ENLKHQPG
gi|178557734|ref|NP_001116538.1| microtubule-associated protein tau isoform 6	calpain-2	VEVKSEKL
gi|178557734|ref|NP_001116538.1| microtubule-associated protein tau isoform 6	calpain-2	FEVMEDHA
gi|178557734|ref|NP_001116538.1| microtubule-associated protein tau isoform 6	calpain-2	QIVYKPVD
gi|178557734|ref|NP_001116538.1| microtubule-associated protein tau isoform 6	calpain-2	EIVYKSPV
gi|4557405|ref|NP_000061.1| calpain 3 isoform a	calpain-3	ISVDRPVK
gi|20631958|ref|NP_620116.1| BCL2-associated X protein isoform alpha	calpain-1	LLLQGFIQ
gi|32451486|ref|NP_057502.2| neurofibromin 2 isoform 2	calpain-2	LILQLCIG
gi|32451486|ref|NP_057502.2| neurofibromin 2 isoform 2	calpain-2	RVNKLILQ
gi|156523968|ref|NP_001609.2| poly (ADP-ribose) polymerase family member 1	calpain-1	KKLTVNPG
gi|6912506|ref|NP_036196.1| major intrinsic protein of lens fiber	calpain-2	VELNTQAL

gi|6912506|ref|NP_036196.1| major intrinsic protein of lens fiber	calpain-2	SVLKGAKP
gi|6912506|ref|NP_036196.1| major intrinsic protein of lens fiber	calpain-2	PEVTGEPV
gi|32967266|ref|NP_861970.1| neurofibromin 2 isoform 2	calpain-2	LILQLCIG
gi|32967266|ref|NP_861970.1| neurofibromin 2 isoform 2	calpain-2	RVNKLILQ
gi|188528689|ref|NP_008880.2| syntaxin binding protein 2 isoform a	calpain-2	GGSGTSSR
gi|87578396|ref|NP_002365.3| microtubule-associated protein 2 isoform 1	calpain-2	PDLKNVKS
gi|87578396|ref|NP_002365.3| microtubule-associated protein 2 isoform 1	calpain-2	KNVKSKIG
gi|27765076|ref|NP_775111.1| calpain 3 isoform d	calpain-3	ISVDRPVK
gi|133908610|ref|NP_000816.3| gonadotropin-releasing hormone 1 precursor	calpain-1	WSYGLRPG
gi|10834982|ref|NP_000590.1| insulin-like growth factor binding protein 5	calpain-1	QSLGSFVH
gi|10834982|ref|NP_000590.1| insulin-like growth factor binding protein 5	calpain-1	KKLTQSKF
gi|10834982|ref|NP_000590.1| insulin-like growth factor binding protein 5	calpain-1	AENTAHPR
gi|133908612|ref|NP_001076580.1| gonadotropin-releasing hormone 1 precursor	calpain-1	WSYGLRPG
gi|68509932|ref|NP_001020263.1| myelin basic protein isoform 4	calpain-1	VTPRTPPP
gi|68509932|ref|NP_001020263.1| myelin basic protein isoform 4	calpain-1	KNIVTPRT
gi|68509932|ref|NP_001020263.1| myelin basic protein isoform 4	calpain-2	VTPRTPPP
gi|68509932|ref|NP_001020263.1| myelin basic protein isoform 4	calpain-2	KNIVTPRT
gi|4506939|ref|NP_000184.1| sonic hedgehog preproprotein	Sonic hedgehog protein	KSGGCFPG
gi|119392086|ref|NP_002172.2| Indian hedgehog homolog	Indian hedgehog protein	KTGGCFPA
gi|4507801|ref|NP_003343.1| SMT3 suppressor of mif two 3 homolog 1 isoform a precursor	SENP1 peptidase	QTGGHSTV
gi|54792065|ref|NP_001005781.1| SMT3 suppressor of mif two 3 homolog 1 isoform a precursor	SENP1 peptidase	QTGGHSTV
gi|54792067|ref|NP_001005782.1| SMT3 suppressor of mif two 3 homolog 1 isoform b precursor	SENP1 peptidase	QTGGHSTV
gi|5453760|ref|NP_006147.1| neural precursor cell expressed developmentally down-regulated 8	SENP8 peptidase	LRGGGGLR
gi|134276943|ref|NP_036423.4| extra spindle poles like 1	separase	EIMRTIPE
gi|134276943|ref|NP_036423.4| extra spindle poles like 1	separase	EILRGSDG
gi|134276943|ref|NP_036423.4| extra spindle poles like 1	separase	ELLRLDSS
gi|5453994|ref|NP_006256.1| RAD21 homolog	separase	EIMREGSA
gi|5453994|ref|NP_006256.1| RAD21 homolog	separase	EPSRLQES

gi|148277548|ref|NP_001078950.1| microtubule-associated protein 1 light chain 3 beta 2	autophagin-1	ETFGMKLS
gi|12383056|ref|NP_073729.1| microtubule-associated proteins 1A/1B light chain 3	autophagin-1	ETFGMKLS

Metallo Proteases

gi|5454114|ref|NP_006278.1| tissue factor pathway inhibitor isoform a precursor	matrix metallopeptidase-12	PPLKLMHS
gi|73760409|ref|NP_001027452.1| tissue factor pathway inhibitor isoform b precursor	matrix metallopeptidase-12	PPLKLMHS
gi|89276751|ref|NP_000084.3| alpha 1 type V collagen preproprotein	matrix metallopeptidase-9	GPPGVVGP
gi|148536825|ref|NP_001836.2| alpha 1 type IV collagen preproprotein	matrix metallopeptidase-3	GPPGLKGL
gi|148536825|ref|NP_001836.2| alpha 1 type IV collagen preproprotein	matrix metallopeptidase-9	GPPGIVIG
gi|50363217|ref|NP_000286.3| serine proteinase inhibitor clade A member 1	matrix metallopeptidase-7	GAMFLEAI
gi|50363217|ref|NP_000286.3| serine proteinase inhibitor clade A member 1	matrix metallopeptidase-8	GAMFLEAI
gi|50363217|ref|NP_000286.3| serine proteinase inhibitor clade A member 1	matrix metallopeptidase-8	EAIPMSIP
gi|50363217|ref|NP_000286.3| serine proteinase inhibitor clade A member 1	matrix metallopeptidase-9	GAMFLEAI
gi|50363217|ref|NP_000286.3| serine proteinase inhibitor clade A member 1	matrix metallopeptidase-9	EAIPMSIP
gi|50363217|ref|NP_000286.3| serine proteinase inhibitor clade A member 1	matrix metallopeptidase-11	AAGAMFLE
gi|50363217|ref|NP_000286.3| serine proteinase inhibitor clade A member 1	matrix metallopeptidase-7	EAIPMSIP
gi|50363217|ref|NP_000286.3| serine proteinase inhibitor clade A member 1	matrix metallopeptidase-12	RPFEVKDT
gi|50363217|ref|NP_000286.3| serine proteinase inhibitor clade A member 1	matrix metallopeptidase-12	GAMFLEAI

gi|50363217|ref|NP_000286.3| serine proteinase inhibitor clade A member 1	matrix metallopeptidase-12	EAIPMSIP
gi|50363217|ref|NP_000286.3| serine proteinase inhibitor clade A member 1	membrane-type matrix metallopeptidase-6	GAMFLEAI
gi|50363217|ref|NP_000286.3| serine proteinase inhibitor clade A member 1	membrane-type matrix metallopeptidase-6	EAIPMSIP
gi|50363217|ref|NP_000286.3| serine proteinase inhibitor clade A member 1	matrix metallopeptidase-26	GAMFLEAI
gi|50363217|ref|NP_000286.3| serine proteinase inhibitor clade A member 1	matrix metallopeptidase-26	EAIPMSIP
gi|50363217|ref|NP_000286.3| serine proteinase inhibitor clade A member 1	matrix metallopeptidase-3	EAIPMSIP
gi|19743848|ref|NP_598011.1| decorin isoform b precursor	matrix metallopeptidase-3	DAASLKGL
gi|19743848|ref|NP_598011.1| decorin isoform b precursor	matrix metallopeptidase-7	MLEDEASG
gi|19743848|ref|NP_598011.1| decorin isoform b precursor	matrix metallopeptidase-7	HLRELHLD
gi|39777599|ref|NP_945189.1| transglutaminase 2 isoform b	membrane-type matrix metallopeptidase-1	AFTRANHL
gi|4507171|ref|NP_003109.1| secreted protein acidic cysteine-rich	matrix metallopeptidase-2	HPVELLAR
gi|4507171|ref|NP_003109.1| secreted protein acidic cysteine-rich	matrix metallopeptidase-2	TVAEVTEV
gi|4507171|ref|NP_003109.1| secreted protein acidic cysteine-rich	matrix metallopeptidase-3	HPVELLAR
gi|4507171|ref|NP_003109.1| secreted protein acidic cysteine-rich	matrix metallopeptidase-3	TVAEVTEV
gi|4507171|ref|NP_003109.1| secreted protein acidic cysteine-rich	matrix metallopeptidase-7	HPVELLAR

gi|4507171|ref|NP_003109.1| secreted protein acidic cysteine-rich	matrix metallopeptidase-7	ELAPLRAP
gi|4507171|ref|NP_003109.1| secreted protein acidic cysteine-rich	matrix metallopeptidase-9	NPVQVEVG
gi|4507171|ref|NP_003109.1| secreted protein acidic cysteine-rich	matrix metallopeptidase-13	NPVQVEVG
gi|4507171|ref|NP_003109.1| secreted protein acidic cysteine-rich	matrix metallopeptidase-13	GANPVQVE
gi|111118974|ref|NP_149162.2| collagen type II alpha 1 isoform 2 precursor	matrix metallopeptidase-13	GPQGLAGQ
gi|111118974|ref|NP_149162.2| collagen type II alpha 1 isoform 2 precursor	matrix metallopeptidase-2	GPQGLAGQ
gi|111118974|ref|NP_149162.2| collagen type II alpha 1 isoform 2 precursor	matrix metallopeptidase-7	GGAQLGVM
gi|111118974|ref|NP_149162.2| collagen type II alpha 1 isoform 2 precursor	matrix metallopeptidase-9	NFAAQMAG
gi|111118974|ref|NP_149162.2| collagen type II alpha 1 isoform 2 precursor	matrix metallopeptidase-9	GHRGFTGL
gi|111118974|ref|NP_149162.2| collagen type II alpha 1 isoform 2 precursor	matrix metallopeptidase-9	GARGIQGP
gi|111118974|ref|NP_149162.2| collagen type II alpha 1 isoform 2 precursor	matrix metallopeptidase-9	GPQGARGF
gi|111118974|ref|NP_149162.2| collagen type II alpha 1 isoform 2 precursor	matrix metallopeptidase-9	GPAGEEGK
gi|111118974|ref|NP_149162.2| collagen type II alpha 1 isoform 2 precursor	matrix metallopeptidase-9	GARGLTGR
gi|111118974|ref|NP_149162.2| collagen type II alpha 1 isoform 2 precursor	matrix metallopeptidase-9	GAPGLRGL
gi|111118974|ref|NP_149162.2| collagen type II alpha 1 isoform 2 precursor	matrix metallopeptidase-9	GPPGLQGM
gi|111118974|ref|NP_149162.2| collagen type II alpha 1 isoform 2 precursor	matrix metallopeptidase-9	GPTGVTGP

gi|111118974|ref|NP_149162.2| collagen type II alpha 1 isoform 2 precursor	matrix metallopeptidase-9	GPKGARGD
gi|111118974|ref|NP_149162.2| collagen type II alpha 1 isoform 2 precursor	matrix metallopeptidase-9	GQRGIVGL
gi|111118974|ref|NP_149162.2| collagen type II alpha 1 isoform 2 precursor	matrix metallopeptidase-9	QLGVMQGP
gi|111118974|ref|NP_149162.2| collagen type II alpha 1 isoform 2 precursor	matrix metallopeptidase-3	NFAAQMAG
gi|111118974|ref|NP_149162.2| collagen type II alpha 1 isoform 2 precursor	matrix metallopeptidase-3	FAAQMAGG
gi|111118974|ref|NP_149162.2| collagen type II alpha 1 isoform 2 precursor	matrix metallopeptidase-13	DIKDIVGP
gi|111118974|ref|NP_149162.2| collagen type II alpha 1 isoform 2 precursor	matrix metallopeptidase-13	QLGVMQGP
gi|111118974|ref|NP_149162.2| collagen type II alpha 1 isoform 2 precursor	membrane-type matrix metallopeptidase-1	AQLGVMQG
gi|111118974|ref|NP_149162.2| collagen type II alpha 1 isoform 2 precursor	matrix metallopeptidase-2	GPKGQKGE
gi|4505209|ref|NP_002418.1| matrix metalloproteinase 13 preproprotein	matrix metallopeptidase-13	GIQSLYGP
gi|4505209|ref|NP_002418.1| matrix metalloproteinase 13 preproprotein	matrix metallopeptidase-3	DVGEYNVF
gi|4505209|ref|NP_002418.1| matrix metalloproteinase 13 preproprotein	matrix metallopeptidase-13	GPSGLLAH
gi|4505209|ref|NP_002418.1| matrix metalloproteinase 13 preproprotein	membrane-type matrix metallopeptidase-1	DVGEYNVF
gi|4505209|ref|NP_002418.1| matrix metalloproteinase 13 preproprotein	membrane-type matrix metallopeptidase-1	NLAGILKE
gi|7770077|ref|NP_054703.1| tachykinin 1 isoform gamma precursor	matrix metallopeptidase-3	KPQQFFGL

gi|48762934|ref|NP_000080.2| alpha 2 type I collagen	matrix metallopeptidase-8	GPQGLLGA
gi|48762934|ref|NP_000080.2| alpha 2 type I collagen	matrix metallopeptidase-8	GPQGLLGA
gi|48762934|ref|NP_000080.2| alpha 2 type I collagen	matrix metallopeptidase-2	GPQGLLGA
gi|48762934|ref|NP_000080.2| alpha 2 type I collagen	matrix metallopeptidase-9	GPQGARGF
gi|22538816|ref|NP_005614.2| small inducible cytokine A8 precursor	matrix metallopeptidase-3	QPDSVSIP
gi|22538816|ref|NP_005614.2| small inducible cytokine A8 precursor	matrix metallopeptidase-3	QPDSVSIP
gi|4503271|ref|NP_001911.1| decorin isoform a preproprotein	matrix metallopeptidase-3	DAASLKGL
gi|4503271|ref|NP_001911.1| decorin isoform a preproprotein	matrix metallopeptidase-7	MLEDEASG
gi|4503271|ref|NP_001911.1| decorin isoform a preproprotein	matrix metallopeptidase-7	HLRELHLD
gi|74272287|ref|NP_004985.2| matrix metalloproteinase 9 preproprotein	matrix metallopeptidase-9	TLKAMRTP
gi|74272287|ref|NP_004985.2| matrix metalloproteinase 9 preproprotein	matrix metallopeptidase-3	DLGRFQTF
gi|74272287|ref|NP_004985.2| matrix metalloproteinase 9 preproprotein	matrix metallopeptidase-3	RVAEMRGE
gi|74272287|ref|NP_004985.2| matrix metalloproteinase 9 preproprotein	matrix metallopeptidase-7	RVAEMRGE
gi|74272287|ref|NP_004985.2| matrix metalloproteinase 9 preproprotein	matrix metallopeptidase-26	TLKAMRTP
gi|189163532|ref|NP_001121174.1| serine proteinase inhibitor clade A member 1	matrix metallopeptidase-7	GAMFLEAI
gi|189163532|ref|NP_001121174.1| serine proteinase inhibitor clade A member 1	matrix metallopeptidase-8	GAMFLEAI

gi|189163532|ref|NP_001121174.1| serine proteinase inhibitor clade A member 1	matrix metallopeptidase-8	EAIPMSIP
gi|189163532|ref|NP_001121174.1| serine proteinase inhibitor clade A member 1	matrix metallopeptidase-9	GAMFLEAI
gi|189163532|ref|NP_001121174.1| serine proteinase inhibitor clade A member 1	matrix metallopeptidase-9	EAIPMSIP
gi|189163532|ref|NP_001121174.1| serine proteinase inhibitor clade A member 1	matrix metallopeptidase-11	AAGAMFLE
gi|189163532|ref|NP_001121174.1| serine proteinase inhibitor clade A member 1	matrix metallopeptidase-7	EAIPMSIP
gi|189163532|ref|NP_001121174.1| serine proteinase inhibitor clade A member 1	matrix metallopeptidase-12	RPFEVKDT
gi|189163532|ref|NP_001121174.1| serine proteinase inhibitor clade A member 1	matrix metallopeptidase-12	GAMFLEAI
gi|189163532|ref|NP_001121174.1| serine proteinase inhibitor clade A member 1	matrix metallopeptidase-12	EAIPMSIP
gi|189163532|ref|NP_001121174.1| serine proteinase inhibitor clade A member 1	membrane-type matrix metallopeptidase-6	GAMFLEAI
gi|189163532|ref|NP_001121174.1| serine proteinase inhibitor clade A member 1	membrane-type matrix metallopeptidase-6	EAIPMSIP
gi|189163532|ref|NP_001121174.1| serine proteinase inhibitor clade A member 1	matrix metallopeptidase-26	GAMFLEAI
gi|189163532|ref|NP_001121174.1| serine proteinase inhibitor clade A member 1	matrix metallopeptidase-26	EAIPMSIP
gi|189163532|ref|NP_001121174.1| serine proteinase inhibitor clade A member 1	matrix metallopeptidase-3	EAIPMSIP
gi|7770075|ref|NP_054702.1| tachykinin 1 isoform alpha precursor	matrix metallopeptidase-3	KPQQFFGL
gi|111118976|ref|NP_001835.3| collagen type II alpha 1 isoform 1 precursor	matrix metallopeptidase-13	GPQGLAGQ

gi|111118976|ref|NP_001835.3| collagen type II alpha 1 isoform 1 precursor	matrix metallopeptidase-2	GPQGLAGQ
gi|111118976|ref|NP_001835.3| collagen type II alpha 1 isoform 1 precursor	matrix metallopeptidase-7	GGAQLGVM
gi|111118976|ref|NP_001835.3| collagen type II alpha 1 isoform 1 precursor	matrix metallopeptidase-9	NFAAQMAG
gi|111118976|ref|NP_001835.3| collagen type II alpha 1 isoform 1 precursor	matrix metallopeptidase-9	GHRGFTGL
gi|111118976|ref|NP_001835.3| collagen type II alpha 1 isoform 1 precursor	matrix metallopeptidase-9	GARGIQGP
gi|111118976|ref|NP_001835.3| collagen type II alpha 1 isoform 1 precursor	matrix metallopeptidase-9	GPQGARGF
gi|111118976|ref|NP_001835.3| collagen type II alpha 1 isoform 1 precursor	matrix metallopeptidase-9	GPAGEEGK
gi|111118976|ref|NP_001835.3| collagen type II alpha 1 isoform 1 precursor	matrix metallopeptidase-9	GARGLTGR
gi|111118976|ref|NP_001835.3| collagen type II alpha 1 isoform 1 precursor	matrix metallopeptidase-9	GAPGLRGL
gi|111118976|ref|NP_001835.3| collagen type II alpha 1 isoform 1 precursor	matrix metallopeptidase-9	GPPGLQGM
gi|111118976|ref|NP_001835.3| collagen type II alpha 1 isoform 1 precursor	matrix metallopeptidase-9	GPTGVTGP
gi|111118976|ref|NP_001835.3| collagen type II alpha 1 isoform 1 precursor	matrix metallopeptidase-9	GPKGARGD
gi|111118976|ref|NP_001835.3| collagen type II alpha 1 isoform 1 precursor	matrix metallopeptidase-9	GQRGIVGL
gi|111118976|ref|NP_001835.3| collagen type II alpha 1 isoform 1 precursor	matrix metallopeptidase-9	QLGVMQGP
gi|111118976|ref|NP_001835.3| collagen type II alpha 1 isoform 1 precursor	matrix metallopeptidase-3	NFAAQMAG
gi|111118976|ref|NP_001835.3| collagen type II alpha 1 isoform 1 precursor	matrix metallopeptidase-3	FAAQMAGG

gi|111118976|ref|NP_001835.3| collagen type II alpha 1 isoform 1 precursor	matrix metallopeptidase-13	DIKDIVGP
gi|111118976|ref|NP_001835.3| collagen type II alpha 1 isoform 1 precursor	matrix metallopeptidase-13	QLGVMQGP
gi|111118976|ref|NP_001835.3| collagen type II alpha 1 isoform 1 precursor	membrane-type matrix metallopeptidase-1	AQLGVMQG
gi|111118976|ref|NP_001835.3| collagen type II alpha 1 isoform 1 precursor	matrix metallopeptidase-2	GPKGQKGE
gi|4557671|ref|NP_000198.1| proinsulin precursor	matrix metallopeptidase-9	LVEALYLV
gi|4557671|ref|NP_000198.1| proinsulin precursor	matrix metallopeptidase-9	SLYQLENY
gi|4557671|ref|NP_000198.1| proinsulin precursor	matrix metallopeptidase-9	GERGFFYT
gi|4557671|ref|NP_000198.1| proinsulin precursor	matrix metallopeptidase-9	VNQHLCGS
gi|4557671|ref|NP_000198.1| proinsulin precursor	matrix metallopeptidase-9	EALYLVCG
gi|4557671|ref|NP_000198.1| proinsulin precursor	matrix metallopeptidase-3	LVEALYLV
gi|4557671|ref|NP_000198.1| proinsulin precursor	matrix metallopeptidase-7	LVEALYLV
gi|19743846|ref|NP_598010.1| decorin isoform a preproprotein	matrix metallopeptidase-3	DAASLKGL
gi|19743846|ref|NP_598010.1| decorin isoform a preproprotein	matrix metallopeptidase-7	MLEDEASG
gi|19743846|ref|NP_598010.1| decorin isoform a preproprotein	matrix metallopeptidase-7	HLRELHLD
gi|73486666|ref|NP_001842.3| alpha 1 type IX collagen isoform 1 precursor	matrix metallopeptidase-3	MAASLKRP
gi|73486666|ref|NP_001842.3| alpha 1 type IX collagen isoform 1 precursor	matrix metallopeptidase-3	MAASLKRP

gi|4826834|ref|NP_004986.1| matrix metalloproteinase 14 preproprotein	membrane-type matrix metallopeptidase-1	RRKRYAIQ
gi|4826834|ref|NP_004986.1| matrix metalloproteinase 14 preproprotein	membrane-type matrix metallopeptidase-1	PQPRTTSR
gi|4826834|ref|NP_004986.1| matrix metalloproteinase 14 preproprotein	membrane-type matrix metallopeptidase-1	FCIQNYTP
gi|73486664|ref|NP_511040.2| alpha 1 type IX collagen isoform 2 precursor	matrix metallopeptidase-3	MAASLKRP
gi|73486664|ref|NP_511040.2| alpha 1 type IX collagen isoform 2 precursor	matrix metallopeptidase-3	MAASLKRP
gi|70906437|ref|NP_000500.2| fibrinogen gamma chain isoform gamma-A precursor	matrix metallopeptidase-13	TCVAYVAT
gi|70906437|ref|NP_000500.2| fibrinogen gamma chain isoform gamma-A precursor	matrix metallopeptidase-13	TCVAYVAT
gi|70906437|ref|NP_000500.2| fibrinogen gamma chain isoform gamma-A precursor	membrane-type matrix metallopeptidase-1	TCVAYVAT
gi|70906437|ref|NP_000500.2| fibrinogen gamma chain isoform gamma-A precursor	matrix metallopeptidase-26	SKPNMIDA
gi|68509940|ref|NP_001020272.1| Golli-mbp isoform 1	matrix metallopeptidase-9	LSRFSWGA
gi|51873053|ref|NP_001004019.1| fibulin 2 precursor isoform a	matrix metallopeptidase-3	PPAPVQAK
gi|145275213|ref|NP_000496.2| coagulation factor XII precursor	matrix metallopeptidase-12	VVGGLVAL
gi|145275213|ref|NP_000496.2| coagulation factor XII precursor	matrix metallopeptidase-13	VVGGLVAL
gi|145275213|ref|NP_000496.2| coagulation factor XII precursor	matrix metallopeptidase-12	GLVALRGA
gi|145275213|ref|NP_000496.2| coagulation factor XII precursor	matrix metallopeptidase-12	KEHKYKAE

gi|145275213|ref|NP_000496.2| coagulation factor XII precursor	matrix metallopeptidase-13	EPKKVKDH
gi|70906439|ref|NP_068656.2| fibrinogen gamma chain isoform gamma-B precursor	matrix metallopeptidase-3	HLGGAKQV
gi|70906439|ref|NP_068656.2| fibrinogen gamma chain isoform gamma-B precursor	matrix metallopeptidase-13	TCVAYVAT
gi|70906439|ref|NP_068656.2| fibrinogen gamma chain isoform gamma-B precursor	matrix metallopeptidase-13	TCVAYVAT
gi|70906439|ref|NP_068656.2| fibrinogen gamma chain isoform gamma-B precursor	membrane-type matrix metallopeptidase-1	TCVAYVAT
gi|70906439|ref|NP_068656.2| fibrinogen gamma chain isoform gamma-B precursor	matrix metallopeptidase-26	SKPNMIDA
gi|116292750|ref|NP_005568.2| lipoprotein Lp(a) precursor	matrix metallopeptidase-12	VPPNVILA
gi|188497671|ref|NP_110381.2| serum amyloid A2 isoform a	matrix metallopeptidase-2	GPGGAWAA
gi|188497671|ref|NP_110381.2| serum amyloid A2 isoform a	matrix metallopeptidase-3	AAEVISNA
gi|29725624|ref|NP_775736.2| collagen type XXIII alpha 1	matrix metallopeptidase-2	GPKGQKGD
gi|29725624|ref|NP_775736.2| collagen type XXIII alpha 1	matrix metallopeptidase-9	GPKGQKGD
gi|4502071|ref|NP_001133.1| amelogenin (X chromosome) isoform 1 precursor	matrix metallopeptidase-20	MGGWLHHQ
gi|189163536|ref|NP_001121176.1| serine proteinase inhibitor clade A member 1	matrix metallopeptidase-7	GAMFLEAI
gi|189163536|ref|NP_001121176.1| serine proteinase inhibitor clade A member 1	matrix metallopeptidase-8	GAMFLEAI
gi|189163536|ref|NP_001121176.1| serine proteinase inhibitor clade A member 1	matrix metallopeptidase-8	EAIPMSIP
gi|189163536|ref|NP_001121176.1| serine proteinase inhibitor clade A member 1	matrix metallopeptidase-9	GAMFLEAI

gi|189163536|ref|NP_001121176.1| serine proteinase inhibitor clade A member 1	matrix metallopeptidase-9	EAIPMSIP
gi|189163536|ref|NP_001121176.1| serine proteinase inhibitor clade A member 1	matrix metallopeptidase-11	AAGAMFLE
gi|189163536|ref|NP_001121176.1| serine proteinase inhibitor clade A member 1	matrix metallopeptidase-7	EAIPMSIP
gi|189163536|ref|NP_001121176.1| serine proteinase inhibitor clade A member 1	matrix metallopeptidase-12	RPFEVKDT
gi|189163536|ref|NP_001121176.1| serine proteinase inhibitor clade A member 1	matrix metallopeptidase-12	GAMFLEAI
gi|189163536|ref|NP_001121176.1| serine proteinase inhibitor clade A member 1	matrix metallopeptidase-12	EAIPMSIP
gi|189163536|ref|NP_001121176.1| serine proteinase inhibitor clade A member 1	membrane-type matrix metallopeptidase-6	GAMFLEAI
gi|189163536|ref|NP_001121176.1| serine proteinase inhibitor clade A member 1	membrane-type matrix metallopeptidase-6	EAIPMSIP
gi|189163536|ref|NP_001121176.1| serine proteinase inhibitor clade A member 1	matrix metallopeptidase-26	GAMFLEAI
gi|189163536|ref|NP_001121176.1| serine proteinase inhibitor clade A member 1	matrix metallopeptidase-26	EAIPMSIP
gi|189163536|ref|NP_001121176.1| serine proteinase inhibitor clade A member 1	matrix metallopeptidase-3	EAIPMSIP
gi|116829964|ref|NP_002247.3| KiSS-1 metastasis-suppressor	matrix metallopeptidase-2	NSFGLRFG
gi|116829964|ref|NP_002247.3| KiSS-1 metastasis-suppressor	membrane-type matrix metallopeptidase-1	NSFGLRFG
gi|116829964|ref|NP_002247.3| KiSS-1 metastasis-suppressor	membrane-type matrix metallopeptidase-3	NSFGLRFG

gi|39777597|ref|NP_004604.2| transglutaminase 2 isoform a	membrane-type matrix metallopeptidase-1	AFTRANHL
gi|4502147|ref|NP_001633.1| amyloid beta (A4) precursor-like protein 2	membrane-type matrix metallopeptidase-3	RVEAMLND
gi|47132547|ref|NP_473375.2| fibronectin 1 isoform 7 preproprotein	matrix metallopeptidase-26	SPVAVSQS
gi|47132549|ref|NP_997639.1| fibronectin 1 isoform 6 preproprotein	matrix metallopeptidase-3	PFSPLVAT
gi|47132549|ref|NP_997639.1| fibronectin 1 isoform 6 preproprotein	matrix metallopeptidase-26	SPVAVSQS
gi|47132553|ref|NP_997641.1| fibronectin 1 isoform 5 preproprotein	matrix metallopeptidase-3	PFSPLVAT
gi|47132553|ref|NP_997641.1| fibronectin 1 isoform 5 preproprotein	matrix metallopeptidase-26	SPVAVSQS
gi|47132555|ref|NP_997643.1| fibronectin 1 isoform 4 preproprotein	matrix metallopeptidase-3	PFSPLVAT
gi|47132555|ref|NP_997643.1| fibronectin 1 isoform 4 preproprotein	matrix metallopeptidase-26	SPVAVSQS
gi|4505259|ref|NP_002436.1| macrophage scavenger receptor 1 isoform type 2	matrix metallopeptidase-2	GPKGQKGE
gi|111118970|ref|NP_542411.2| collagen type XI alpha 2 isoform 1 preproprotein	matrix metallopeptidase-9	GPPGVVGP
gi|47132557|ref|NP_997647.1| fibronectin 1 isoform 1 preproprotein	matrix metallopeptidase-3	PFSPLVAT
gi|47132557|ref|NP_997647.1| fibronectin 1 isoform 1 preproprotein	matrix metallopeptidase-26	SPVAVSQS
gi|16933542|ref|NP_002017.1| fibronectin 1 isoform 3 preproprotein	matrix metallopeptidase-3	PFSPLVAT
gi|16933542|ref|NP_002017.1| fibronectin 1 isoform 3 preproprotein	matrix metallopeptidase-26	SPVAVSQS

gi|98985810|ref|NP_542196.2| alpha 1 type XI collagen isoform B preproprotein	matrix metallopeptidase-9	GPGGVVGP
gi|87298828|ref|NP_000482.3| complement component 1 q subcomponent B chain precursor	matrix metallopeptidase-3	ESGDYKAT
gi|47132551|ref|NP_997640.1| fibronectin 1 isoform 2 preproprotein	matrix metallopeptidase-3	PFSPLVAT
gi|47132551|ref|NP_997640.1| fibronectin 1 isoform 2 preproprotein	matrix metallopeptidase-26	SPVAVSQS
gi|24430141|ref|NP_000129.2| fibrillin 1 precursor	matrix metallopeptidase-9	LPVNVTDY
gi|24430141|ref|NP_000129.2| fibrillin 1 precursor	matrix metallopeptidase-12	EGFSLSSS
gi|166235903|ref|NP_001107573.1| complement component 1 q subcomponent C chain precursor	matrix metallopeptidase-2	GPKGQKGE
gi|166235903|ref|NP_001107573.1| complement component 1 q subcomponent C chain precursor	matrix metallopeptidase-9	GPPGMPGV
gi|111118968|ref|NP_542410.2| collagen type XI alpha 2 isoform 3 preproprotein	matrix metallopeptidase-9	GPPGVVGP
gi|20357512|ref|NP_619729.1| macrophage scavenger receptor 1 isoform type 1	matrix metallopeptidase-2	GPKGQKGE
gi|4506841|ref|NP_002973.1| small inducible cytokine A2 precursor	matrix metallopeptidase-3	QPDAINAP
gi|4506841|ref|NP_002973.1| small inducible cytokine A2 precursor	matrix metallopeptidase-3	QPDAINAP
gi|48255935|ref|NP_000601.3| CD44 antigen isoform 1 precursor	membrane-type matrix metallopeptidase-1	GEYRTNPE
gi|48255935|ref|NP_000601.3| CD44 antigen isoform 1 precursor	membrane-type matrix metallopeptidase-1	SSERSSTS
gi|189163528|ref|NP_001121172.1| serine proteinase inhibitor clade A member 1	matrix metallopeptidase-7	GAMFLEAI

gi|189163528|ref|NP_001121172.1| serine proteinase inhibitor clade A member 1	matrix metallopeptidase-8	GAMFLEAI
gi|189163528|ref|NP_001121172.1| serine proteinase inhibitor clade A member 1	matrix metallopeptidase-8	EAIPMSIP
gi|189163528|ref|NP_001121172.1| serine proteinase inhibitor clade A member 1	matrix metallopeptidase-9	GAMFLEAI
gi|189163528|ref|NP_001121172.1| serine proteinase inhibitor clade A member 1	matrix metallopeptidase-9	EAIPMSIP
gi|189163528|ref|NP_001121172.1| serine proteinase inhibitor clade A member 1	matrix metallopeptidase-11	AAGAMFLE
gi|189163528|ref|NP_001121172.1| serine proteinase inhibitor clade A member 1	matrix metallopeptidase-7	EAIPMSIP
gi|189163528|ref|NP_001121172.1| serine proteinase inhibitor clade A member 1	matrix metallopeptidase-12	RPFEVKDT
gi|189163528|ref|NP_001121172.1| serine proteinase inhibitor clade A member 1	matrix metallopeptidase-12	GAMFLEAI
gi|189163528|ref|NP_001121172.1| serine proteinase inhibitor clade A member 1	matrix metallopeptidase-12	EAIPMSIP
gi|189163528|ref|NP_001121172.1| serine proteinase inhibitor clade A member 1	membrane-type matrix metallopeptidase-6	GAMFLEAI
gi|189163528|ref|NP_001121172.1| serine proteinase inhibitor clade A member 1	membrane-type matrix metallopeptidase-6	EAIPMSIP
gi|189163528|ref|NP_001121172.1| serine proteinase inhibitor clade A member 1	matrix metallopeptidase-26	GAMFLEAI
gi|189163528|ref|NP_001121172.1| serine proteinase inhibitor clade A member 1	matrix metallopeptidase-26	EAIPMSIP
gi|189163528|ref|NP_001121172.1| serine proteinase inhibitor clade A member 1	matrix metallopeptidase-3	EAIPMSIP
gi|162809334|ref|NP_002855.2| pregnancy-zone protein	matrix metallopeptidase-2	QLGTYNVI

gi|17986258|ref|NP_066299.2| myosin light chain 6 alkali smooth muscle and non-muscle isoform 1	matrix metallopeptidase-2	CINYEAFV
gi|4505215|ref|NP_002412.1| matrix metalloproteinase 1 preproprotein	matrix metallopeptidase-3	DVAQFVLT
gi|4505215|ref|NP_002412.1| matrix metalloproteinase 1 preproprotein	matrix metallopeptidase-1	PVQPIGPQ
gi|4505215|ref|NP_002412.1| matrix metalloproteinase 1 preproprotein	matrix metallopeptidase-1	DVAQFVLT
gi|4505215|ref|NP_002412.1| matrix metalloproteinase 1 preproprotein	matrix metallopeptidase-1	VAQFVLTE
gi|4505215|ref|NP_002412.1| matrix metalloproteinase 1 preproprotein	matrix metallopeptidase-1	DAETLKVM
gi|4505215|ref|NP_002412.1| matrix metalloproteinase 1 preproprotein	matrix metallopeptidase-7	DVAQFVLT
gi|111118972|ref|NP_542412.2| collagen type XI alpha 2 isoform 2 preproprotein	matrix metallopeptidase-9	GPPGVVGP
gi|13435402|ref|NP_006264.2| chemokine (C-C motif) ligand 7 precursor	matrix metallopeptidase-3	QPVGINTS
gi|13435402|ref|NP_006264.2| chemokine (C-C motif) ligand 7 precursor	matrix metallopeptidase-2	QPVGINTS
gi|13435402|ref|NP_006264.2| chemokine (C-C motif) ligand 7 precursor	matrix metallopeptidase-13	QPVGINTS
gi|13435402|ref|NP_006264.2| chemokine (C-C motif) ligand 7 precursor	membrane-type matrix metallopeptidase-1	QPVGINTS
gi|13435402|ref|NP_006264.2| chemokine (C-C motif) ligand 7 precursor	matrix metallopeptidase-3	QPVGINTS
gi|20357515|ref|NP_619730.1| macrophage scavenger receptor 1 isoform type 3	matrix metallopeptidase-2	GPKGQKGE
gi|56786155|ref|NP_758957.2| complement component 1 q subcomponent C chain precursor	matrix metallopeptidase-2	GPKGQKGE
gi|56786155|ref|NP_758957.2| complement component 1 q subcomponent C chain precursor	matrix metallopeptidase-9	GPPGMPGV

gi|189163534|ref|NP_001121175.1| serine proteinase inhibitor clade A member 1	matrix metallopeptidase-7	GAMFLEAI
gi|189163534|ref|NP_001121175.1| serine proteinase inhibitor clade A member 1	matrix metallopeptidase-8	GAMFLEAI
gi|189163534|ref|NP_001121175.1| serine proteinase inhibitor clade A member 1	matrix metallopeptidase-8	EAIPMSIP
gi|189163534|ref|NP_001121175.1| serine proteinase inhibitor clade A member 1	matrix metallopeptidase-9	GAMFLEAI
gi|189163534|ref|NP_001121175.1| serine proteinase inhibitor clade A member 1	matrix metallopeptidase-9	EAIPMSIP
gi|189163534|ref|NP_001121175.1| serine proteinase inhibitor clade A member 1	matrix metallopeptidase-11	AAGAMFLE
gi|189163534|ref|NP_001121175.1| serine proteinase inhibitor clade A member 1	matrix metallopeptidase-7	EAIPMSIP
gi|189163534|ref|NP_001121175.1| serine proteinase inhibitor clade A member 1	matrix metallopeptidase-12	RPFEVKDT
gi|189163534|ref|NP_001121175.1| serine proteinase inhibitor clade A member 1	matrix metallopeptidase-12	GAMFLEAI
gi|189163534|ref|NP_001121175.1| serine proteinase inhibitor clade A member 1	matrix metallopeptidase-12	EAIPMSIP
gi|189163534|ref|NP_001121175.1| serine proteinase inhibitor clade A member 1	membrane-type matrix metallopeptidase-6	GAMFLEAI
gi|189163534|ref|NP_001121175.1| serine proteinase inhibitor clade A member 1	membrane-type matrix metallopeptidase-6	EAIPMSIP
gi|189163534|ref|NP_001121175.1| serine proteinase inhibitor clade A member 1	matrix metallopeptidase-26	GAMFLEAI
gi|189163534|ref|NP_001121175.1| serine proteinase inhibitor clade A member 1	matrix metallopeptidase-26	EAIPMSIP
gi|189163534|ref|NP_001121175.1| serine proteinase inhibitor clade A member 1	matrix metallopeptidase-3	EAIPMSIP

gi|4503053|ref|NP_001875.1| hyaluronan and proteoglycan link protein 1	matrix metallopeptidase-3	RAIHIQAE
gi|4503053|ref|NP_001875.1| hyaluronan and proteoglycan link protein 1	matrix metallopeptidase-2	RAIHIQAE
gi|4503053|ref|NP_001875.1| hyaluronan and proteoglycan link protein 1	matrix metallopeptidase-2	GPHLLVEA
gi|4503053|ref|NP_001875.1| hyaluronan and proteoglycan link protein 1	matrix metallopeptidase-10	RAIHIQAE
gi|4503053|ref|NP_001875.1| hyaluronan and proteoglycan link protein 1	matrix metallopeptidase-10	GPHLLVEA
gi|4503053|ref|NP_001875.1| hyaluronan and proteoglycan link protein 1	matrix metallopeptidase-7	GPHLLVEA
gi|4503053|ref|NP_001875.1| hyaluronan and proteoglycan link protein 1	matrix metallopeptidase-9	RAIHIQAE
gi|4503053|ref|NP_001875.1| hyaluronan and proteoglycan link protein 1	matrix metallopeptidase-3	RAIHIQAE
gi|4503053|ref|NP_001875.1| hyaluronan and proteoglycan link protein 1	matrix metallopeptidase-9	RAIHIQAE
gi|4502951|ref|NP_000081.1| collagen type III alpha 1 preproprotein	membrane-type matrix metallopeptidase-3	GPLGIAGI
gi|4502951|ref|NP_000081.1| collagen type III alpha 1 preproprotein	membrane-type matrix metallopeptidase-3	GIAGITGA
gi|126090883|ref|NP_001126.2| aggrecan isoform 1 precursor	matrix metallopeptidase-8	IPENFFGV
gi|126090883|ref|NP_001126.2| aggrecan isoform 1 precursor	matrix metallopeptidase-8	TSEDLVVQ
gi|126090883|ref|NP_001126.2| aggrecan isoform 1 precursor	matrix metallopeptidase-8	TEGEARGS
gi|126090883|ref|NP_001126.2| aggrecan isoform 1 precursor	matrix metallopeptidase-1	IPENFFGV

gi|126090883|ref|NP_001126.2| aggrecan isoform 1 precursor	matrix metallopeptidase-9	IPENFFGV
gi|126090883|ref|NP_001126.2| aggrecan isoform 1 precursor	matrix metallopeptidase-7	IPENFFGV
gi|126090883|ref|NP_001126.2| aggrecan isoform 1 precursor	matrix metallopeptidase-7	TSEDLVVQ
gi|126090883|ref|NP_001126.2| aggrecan isoform 1 precursor	matrix metallopeptidase-3	IPENFFGV
gi|126090883|ref|NP_001126.2| aggrecan isoform 1 precursor	matrix metallopeptidase-3	ARGSVILT
gi|126090883|ref|NP_001126.2| aggrecan isoform 1 precursor	matrix metallopeptidase-13	IPENFFGV
gi|126090883|ref|NP_001126.2| aggrecan isoform 1 precursor	matrix metallopeptidase-13	TSEDLVVQ
gi|126090883|ref|NP_001126.2| aggrecan isoform 1 precursor	membrane-type matrix metallopeptidase-1	IPENFFGV
gi|126090883|ref|NP_001126.2| aggrecan isoform 1 precursor	membrane-type matrix metallopeptidase-1	TSEDLVVQ
gi|126090883|ref|NP_001126.2| aggrecan isoform 1 precursor	matrix metallopeptidase-20	IPENFFGV
gi|126090883|ref|NP_001126.2| aggrecan isoform 1 precursor	matrix metallopeptidase-19	IPENFFGV
gi|4505221|ref|NP_002415.1| matrix metalloproteinase 8 preproprotein	matrix metallopeptidase-3	DSGGFMLT
gi|4505221|ref|NP_002415.1| matrix metalloproteinase 8 preproprotein	matrix metallopeptidase-3	DSGGFMLT
gi|4505221|ref|NP_002415.1| matrix metalloproteinase 8 preproprotein	matrix metallopeptidase-8	SSNPIQPT
gi|11342666|ref|NP_004521.1| matrix metalloproteinase 2 isoform a preproprotein	matrix metallopeptidase-2	DVANYNFF

gi|70906435|ref|NP_005132.2| fibrinogen beta chain preproprotein	matrix metallopeptidase-13	ERPIRNSV
gi|126090892|ref|NP_037359.2| aggrecan isoform 2 precursor	matrix metallopeptidase-8	IPENFFGV
gi|126090892|ref|NP_037359.2| aggrecan isoform 2 precursor	matrix metallopeptidase-8	TSEDLVVQ
gi|126090892|ref|NP_037359.2| aggrecan isoform 2 precursor	matrix metallopeptidase-8	TEGEARGS
gi|126090892|ref|NP_037359.2| aggrecan isoform 2 precursor	matrix metallopeptidase-1	IPENFFGV
gi|126090892|ref|NP_037359.2| aggrecan isoform 2 precursor	matrix metallopeptidase-9	IPENFFGV
gi|126090892|ref|NP_037359.2| aggrecan isoform 2 precursor	matrix metallopeptidase-7	IPENFFGV
gi|126090892|ref|NP_037359.2| aggrecan isoform 2 precursor	matrix metallopeptidase-7	TSEDLVVQ
gi|126090892|ref|NP_037359.2| aggrecan isoform 2 precursor	matrix metallopeptidase-3	IPENFFGV
gi|126090892|ref|NP_037359.2| aggrecan isoform 2 precursor	matrix metallopeptidase-3	ARGSVILT
gi|126090892|ref|NP_037359.2| aggrecan isoform 2 precursor	matrix metallopeptidase-13	IPENFFGV
gi|126090892|ref|NP_037359.2| aggrecan isoform 2 precursor	matrix metallopeptidase-13	TSEDLVVQ
gi|126090892|ref|NP_037359.2| aggrecan isoform 2 precursor	membrane-type matrix metallopeptidase-1	IPENFFGV
gi|126090892|ref|NP_037359.2| aggrecan isoform 2 precursor	membrane-type matrix metallopeptidase-1	TSEDLVVQ
gi|126090892|ref|NP_037359.2| aggrecan isoform 2 precursor	matrix metallopeptidase-20	IPENFFGV

gi|126090892|ref|NP_037359.2| aggrecan isoform 2 precursor	matrix metallopeptidase-19	IPENFFGV
gi|40316910|ref|NP_954630.1| serum amyloid A1 preproprotein	matrix metallopeptidase-2	GPGGAWAA
gi|189163538|ref|NP_001121177.1| serine proteinase inhibitor clade A member 1	matrix metallopeptidase-7	GAMFLEAI
gi|189163538|ref|NP_001121177.1| serine proteinase inhibitor clade A member 1	matrix metallopeptidase-8	GAMFLEAI
gi|189163538|ref|NP_001121177.1| serine proteinase inhibitor clade A member 1	matrix metallopeptidase-8	EAIPMSIP
gi|189163538|ref|NP_001121177.1| serine proteinase inhibitor clade A member 1	matrix metallopeptidase-9	GAMFLEAI
gi|189163538|ref|NP_001121177.1| serine proteinase inhibitor clade A member 1	matrix metallopeptidase-9	EAIPMSIP
gi|189163538|ref|NP_001121177.1| serine proteinase inhibitor clade A member 1	matrix metallopeptidase-11	AAGAMFLE
gi|189163538|ref|NP_001121177.1| serine proteinase inhibitor clade A member 1	matrix metallopeptidase-7	EAIPMSIP
gi|189163538|ref|NP_001121177.1| serine proteinase inhibitor clade A member 1	matrix metallopeptidase-12	RPFEVKDT
gi|189163538|ref|NP_001121177.1| serine proteinase inhibitor clade A member 1	matrix metallopeptidase-12	GAMFLEAI
gi|189163538|ref|NP_001121177.1| serine proteinase inhibitor clade A member 1	matrix metallopeptidase-12	EAIPMSIP
gi|189163538|ref|NP_001121177.1| serine proteinase inhibitor clade A member 1	membrane-type matrix metallopeptidase-6	GAMFLEAI
gi|189163538|ref|NP_001121177.1| serine proteinase inhibitor clade A member 1	membrane-type matrix metallopeptidase-6	EAIPMSIP
gi|189163538|ref|NP_001121177.1| serine proteinase inhibitor clade A member 1	matrix metallopeptidase-26	GAMFLEAI

gi|189163538|ref|NP_001121177.1| serine proteinase inhibitor clade A member 1	matrix metallopeptidase-26	EAIPMSIP
gi|189163538|ref|NP_001121177.1| serine proteinase inhibitor clade A member 1	matrix metallopeptidase-3	EAIPMSIP
gi|25952111|ref|NP_000585.2| tumor necrosis factor alpha	membrane-type matrix metallopeptidase-6	LAQAVRSS
gi|25952111|ref|NP_000585.2| tumor necrosis factor alpha	membrane-type matrix metallopeptidase-4	SPLAQAVR
gi|25952111|ref|NP_000585.2| tumor necrosis factor alpha	membrane-type matrix metallopeptidase-4	PLAQAVRS
gi|25952111|ref|NP_000585.2| tumor necrosis factor alpha	membrane-type matrix metallopeptidase-1	SPLAQAVR
gi|25952111|ref|NP_000585.2| tumor necrosis factor alpha	membrane-type matrix metallopeptidase-1	LISPLAQA
gi|25952111|ref|NP_000585.2| tumor necrosis factor alpha	membrane-type matrix metallopeptidase-4	PLAQAVRS
gi|4502073|ref|NP_001134.1| amelogenin Y-linked precursor	matrix metallopeptidase-20	PNLPLPAQ
gi|4502073|ref|NP_001134.1| amelogenin Y-linked precursor	matrix metallopeptidase-20	LPLPAQQP
gi|4502073|ref|NP_001134.1| amelogenin Y-linked precursor	matrix metallopeptidase-20	MGGWLHHQ
gi|189217853|ref|NP_001121363.1| matrix metalloproteinase 2 isoform b	matrix metallopeptidase-2	DVANYNFF
gi|40316912|ref|NP_000322.2| serum amyloid A1 preproprotein	matrix metallopeptidase-2	GPGGAWAA
gi|88853069|ref|NP_000629.3| vitronectin precursor	matrix metallopeptidase-26	KPEGIDSR

gi|4503057|ref|NP_001876.1| crystallin alpha B	matrix metallopeptidase-9	MDIAIHHP
gi|4503057|ref|NP_001876.1| crystallin alpha B	matrix metallopeptidase-9	SPSRLFDQ
gi|4503057|ref|NP_001876.1| crystallin alpha B	matrix metallopeptidase-9	FFGEHLLE
gi|4503057|ref|NP_001876.1| crystallin alpha B	matrix metallopeptidase-9	SPEELKVK
gi|4503057|ref|NP_001876.1| crystallin alpha B	matrix metallopeptidase-9	DVIEVHGK
gi|4503057|ref|NP_001876.1| crystallin alpha B	matrix metallopeptidase-9	GEHLLESD
gi|4503057|ref|NP_001876.1| crystallin alpha B	matrix metallopeptidase-9	LSPFYLRP
gi|4503057|ref|NP_001876.1| crystallin alpha B	matrix metallopeptidase-9	PFFPFHSP
gi|4503057|ref|NP_001876.1| crystallin alpha B	matrix metallopeptidase-9	LLESDLFP
gi|4503057|ref|NP_001876.1| crystallin alpha B	matrix metallopeptidase-9	APSWFDTG
gi|4503057|ref|NP_001876.1| crystallin alpha B	matrix metallopeptidase-9	VIEVHGKH
gi|4503057|ref|NP_001876.1| crystallin alpha B	matrix metallopeptidase-9	KPAVTAAP
gi|33356556|ref|NP_872621.1| amelogenin (X chromosome) isoform 3 precursor	matrix metallopeptidase-20	MGGWLHHQ
gi|157419138|ref|NP_005553.2| laminin gamma 2 isoform a precursor	membrane-type matrix metallopeptidase-1	GYFGDPLA
gi|50363219|ref|NP_001002236.1| serine proteinase inhibitor clade A member 1	matrix metallopeptidase-7	GAMFLEAI
gi|50363219|ref|NP_001002236.1| serine proteinase inhibitor clade A member 1	matrix metallopeptidase-8	GAMFLEAI

gi|50363219|ref|NP_001002236.1| serine proteinase inhibitor clade A member 1	matrix metallopeptidase-8	EAIPMSIP
gi|50363219|ref|NP_001002236.1| serine proteinase inhibitor clade A member 1	matrix metallopeptidase-9	GAMFLEAI
gi|50363219|ref|NP_001002236.1| serine proteinase inhibitor clade A member 1	matrix metallopeptidase-9	EAIPMSIP
gi|50363219|ref|NP_001002236.1| serine proteinase inhibitor clade A member 1	matrix metallopeptidase-11	AAGAMFLE
gi|50363219|ref|NP_001002236.1| serine proteinase inhibitor clade A member 1	matrix metallopeptidase-7	EAIPMSIP
gi|50363219|ref|NP_001002236.1| serine proteinase inhibitor clade A member 1	matrix metallopeptidase-12	RPFEVKDT
gi|50363219|ref|NP_001002236.1| serine proteinase inhibitor clade A member 1	matrix metallopeptidase-12	GAMFLEAI
gi|50363219|ref|NP_001002236.1| serine proteinase inhibitor clade A member 1	matrix metallopeptidase-12	EAIPMSIP
gi|50363219|ref|NP_001002236.1| serine proteinase inhibitor clade A member 1	membrane-type matrix metallopeptidase-6	GAMFLEAI
gi|50363219|ref|NP_001002236.1| serine proteinase inhibitor clade A member 1	membrane-type matrix metallopeptidase-6	EAIPMSIP
gi|50363219|ref|NP_001002236.1| serine proteinase inhibitor clade A member 1	matrix metallopeptidase-26	GAMFLEAI
gi|50363219|ref|NP_001002236.1| serine proteinase inhibitor clade A member 1	matrix metallopeptidase-26	EAIPMSIP
gi|50363219|ref|NP_001002236.1| serine proteinase inhibitor clade A member 1	matrix metallopeptidase-3	EAIPMSIP
gi|33356558|ref|NP_872622.1| amelogenin (X chromosome) isoform 2 precursor	matrix metallopeptidase-20	MGGWLHHQ
gi|116256354|ref|NP_001837.2| alpha 2 type IV collagen preproprotein	matrix metallopeptidase-2	GPKGQKGE

gi|188497687|ref|NP_001120852.1| serum amyloid A2 isoform b	matrix metallopeptidase-2	GPGGAWAA
gi|189163542|ref|NP_001121179.1| serine proteinase inhibitor clade A member 1	matrix metallopeptidase-7	GAMFLEAI
gi|189163542|ref|NP_001121179.1| serine proteinase inhibitor clade A member 1	matrix metallopeptidase-8	GAMFLEAI
gi|189163542|ref|NP_001121179.1| serine proteinase inhibitor clade A member 1	matrix metallopeptidase-8	EAIPMSIP
gi|189163542|ref|NP_001121179.1| serine proteinase inhibitor clade A member 1	matrix metallopeptidase-9	GAMFLEAI
gi|189163542|ref|NP_001121179.1| serine proteinase inhibitor clade A member 1	matrix metallopeptidase-9	EAIPMSIP
gi|189163542|ref|NP_001121179.1| serine proteinase inhibitor clade A member 1	matrix metallopeptidase-11	AAGAMFLE
gi|189163542|ref|NP_001121179.1| serine proteinase inhibitor clade A member 1	matrix metallopeptidase-7	EAIPMSIP
gi|189163542|ref|NP_001121179.1| serine proteinase inhibitor clade A member 1	matrix metallopeptidase-12	RPFEVKDT
gi|189163542|ref|NP_001121179.1| serine proteinase inhibitor clade A member 1	matrix metallopeptidase-12	GAMFLEAI
gi|189163542|ref|NP_001121179.1| serine proteinase inhibitor clade A member 1	matrix metallopeptidase-12	EAIPMSIP
gi|189163542|ref|NP_001121179.1| serine proteinase inhibitor clade A member 1	membrane-type matrix metallopeptidase-6	GAMFLEAI
gi|189163542|ref|NP_001121179.1| serine proteinase inhibitor clade A member 1	membrane-type matrix metallopeptidase-6	EAIPMSIP
gi|189163542|ref|NP_001121179.1| serine proteinase inhibitor clade A member 1	matrix metallopeptidase-26	GAMFLEAI
gi|189163542|ref|NP_001121179.1| serine proteinase inhibitor clade A member 1	matrix metallopeptidase-26	EAIPMSIP

gi|189163542|ref|NP_001121179.1| serine proteinase inhibitor clade A member 1	matrix metallopeptidase-3	EAIPMSIP
gi|4504615|ref|NP_000587.1| insulin-like growth factor binding protein 1 precursor	matrix metallopeptidase-26	KALHVTNI
gi|4504615|ref|NP_000587.1| insulin-like growth factor binding protein 1 precursor	matrix metallopeptidase-11	KALHVTNI
gi|48255937|ref|NP_001001389.1| CD44 antigen isoform 2 precursor	membrane-type matrix metallopeptidase-1	GEYRTNPE
gi|48255937|ref|NP_001001389.1| CD44 antigen isoform 2 precursor	membrane-type matrix metallopeptidase-1	SSERSSTS
gi|10835159|ref|NP_000593.1| plasminogen activator inhibitor-1	matrix metallopeptidase-3	TVASSSTA
gi|10835159|ref|NP_000593.1| plasminogen activator inhibitor-1	matrix metallopeptidase-3	STAVIVSA
gi|7705753|ref|NP_057075.1| complement component 1 q subcomponent A chain precursor	matrix metallopeptidase-2	GIQGLKGD
gi|7705753|ref|NP_057075.1| complement component 1 q subcomponent A chain precursor	matrix metallopeptidase-2	GPLGARGI
gi|7705753|ref|NP_057075.1| complement component 1 q subcomponent A chain precursor	matrix metallopeptidase-9	GPLGARGI
gi|7705753|ref|NP_057075.1| complement component 1 q subcomponent A chain precursor	matrix metallopeptidase-3	GPLGARGI
gi|4506849|ref|NP_002985.1| chemokine (C-X-C motif) ligand 5 precursor	matrix metallopeptidase-9	PAAAVLRE
gi|91206462|ref|NP_001035147.1| secreted phosphoprotein 1 isoform a	matrix metallopeptidase-3	VVYGLRSK
gi|91206462|ref|NP_001035147.1| secreted phosphoprotein 1 isoform a	matrix metallopeptidase-7	VAQDLNAP
gi|110611235|ref|NP_085059.2| alpha 1 type XVIII collagen isoform 1 precursor	matrix metallopeptidase-7	RADDILAS

gi|110611235|ref|NP_085059.2| alpha 1 type XVIII collagen isoform 1 precursor	matrix metallopeptidase-9	TARPWRAD
gi|110611235|ref|NP_085059.2| alpha 1 type XVIII collagen isoform 1 precursor	matrix metallopeptidase-3	PPAHSHRD
gi|110611235|ref|NP_085059.2| alpha 1 type XVIII collagen isoform 1 precursor	matrix metallopeptidase-3	HHSSYVHL
gi|110611235|ref|NP_085059.2| alpha 1 type XVIII collagen isoform 1 precursor	matrix metallopeptidase-13	APHHSSYV
gi|110611235|ref|NP_085059.2| alpha 1 type XVIII collagen isoform 1 precursor	matrix metallopeptidase-13	HHSSYVHL
gi|110611235|ref|NP_085059.2| alpha 1 type XVIII collagen isoform 1 precursor	matrix metallopeptidase-20	HHSSYVHL
gi|110611235|ref|NP_085059.2| alpha 1 type XVIII collagen isoform 1 precursor	membrane-type matrix metallopeptidase-1	HHSSYVHL
gi|157419140|ref|NP_061486.2| laminin gamma 2 isoform b precursor	membrane-type matrix metallopeptidase-1	GYFGDPLA
gi|5454114|ref|NP_006278.1| tissue factor pathway inhibitor isoform a precursor	matrix metallopeptidase-7	PPLKLMHS
gi|5454114|ref|NP_006278.1| tissue factor pathway inhibitor isoform a precursor	matrix metallopeptidase-9	PPLKLMHS
gi|4502167|ref|NP_000475.1| amyloid beta A4 protein precursor isoform a	matrix metallopeptidase-9	NKGAIIGL
gi|4502167|ref|NP_000475.1| amyloid beta A4 protein precursor isoform a	matrix metallopeptidase-9	AIIGLMVG
gi|4502167|ref|NP_000475.1| amyloid beta A4 protein precursor isoform a	matrix metallopeptidase-9	IIGLMVGG
gi|4502167|ref|NP_000475.1| amyloid beta A4 protein precursor isoform a	matrix metallopeptidase-9	LVFFAEDV
gi|4502167|ref|NP_000475.1| amyloid beta A4 protein precursor isoform a	matrix metallopeptidase-9	FAEDVGSN

gi|4502167|ref|NP_000475.1| amyloid beta A4 protein precursor isoform a	membrane-type matrix metallopeptidase-3	RVEAMLND
gi|4502167|ref|NP_000475.1| amyloid beta A4 protein precursor isoform a	membrane-type matrix metallopeptidase-3	VLANMISE
gi|4502167|ref|NP_000475.1| amyloid beta A4 protein precursor isoform a	membrane-type matrix metallopeptidase-3	EVHHQKLV
gi|4502167|ref|NP_000475.1| amyloid beta A4 protein precursor isoform a	membrane-type matrix metallopeptidase-4	EVHHQKLV
gi|4502167|ref|NP_000475.1| amyloid beta A4 protein precursor isoform a	membrane-type matrix metallopeptidase-4	EVHHQKLV
gi|4502167|ref|NP_000475.1| amyloid beta A4 protein precursor isoform a	membrane-type matrix metallopeptidase-4	HHQKLVFF
gi|115583663|ref|NP_000925.2| alpha-2-plasmin inhibitor	matrix metallopeptidase-3	APRELKEQ
gi|115583663|ref|NP_000925.2| alpha-2-plasmin inhibitor	matrix metallopeptidase-3	SIAMSRMS
gi|115583663|ref|NP_000925.2| alpha-2-plasmin inhibitor	matrix metallopeptidase-3	FLQSLKGF
gi|50845428|ref|NP_001002841.1| atrial/embryonic alkali myosin light chain	matrix metallopeptidase-2	CINYEAFV
gi|4759166|ref|NP_000573.1| secreted phosphoprotein 1 isoform b	matrix metallopeptidase-3	VVYGLRSK
gi|4759166|ref|NP_000573.1| secreted phosphoprotein 1 isoform b	matrix metallopeptidase-7	VAQDLNAP
gi|4503689|ref|NP_000499.1| fibrinogen alpha polypeptide isoform alpha-E preproprotein	matrix metallopeptidase-12	MLGEFVSE
gi|4503689|ref|NP_000499.1| fibrinogen alpha polypeptide isoform alpha-E preproprotein	matrix metallopeptidase-12	HTEKLVTS

gi|4503689|ref|NP_000499.1| fibrinogen alpha polypeptide isoform alpha-E preproprotein	matrix metallopeptidase-13	GDKELRTG
gi|4503689|ref|NP_000499.1| fibrinogen alpha polypeptide isoform alpha-E preproprotein	membrane-type matrix metallopeptidase-1	HTEKLVTS
gi|4503689|ref|NP_000499.1| fibrinogen alpha polypeptide isoform alpha-E preproprotein	membrane-type matrix metallopeptidase-1	DSHSLTTN
gi|4503689|ref|NP_000499.1| fibrinogen alpha polypeptide isoform alpha-E preproprotein	matrix metallopeptidase-8	TAWTADSG
gi|4503689|ref|NP_000499.1| fibrinogen alpha polypeptide isoform alpha-E preproprotein	matrix metallopeptidase-12	TAWTADSG
gi|4503689|ref|NP_000499.1| fibrinogen alpha polypeptide isoform alpha-E preproprotein	matrix metallopeptidase-13	TAWTADSG
gi|4503689|ref|NP_000499.1| fibrinogen alpha polypeptide isoform alpha-E preproprotein	matrix metallopeptidase-26	GDKELRTG
gi|4503689|ref|NP_000499.1| fibrinogen alpha polypeptide isoform alpha-E preproprotein	matrix metallopeptidase-26	HTEKLVTS
gi|41406055|ref|NP_958816.1| amyloid beta A4 protein precursor isoform b	matrix metallopeptidase-9	NKGAIIGL
gi|41406055|ref|NP_958816.1| amyloid beta A4 protein precursor isoform b	matrix metallopeptidase-9	AIIGLMVG
gi|41406055|ref|NP_958816.1| amyloid beta A4 protein precursor isoform b	matrix metallopeptidase-9	IIGLMVGG
gi|41406055|ref|NP_958816.1| amyloid beta A4 protein precursor isoform b	matrix metallopeptidase-9	LVFFAEDV
gi|41406055|ref|NP_958816.1| amyloid beta A4 protein precursor isoform b	matrix metallopeptidase-9	FAEDVGSN
gi|41406055|ref|NP_958816.1| amyloid beta A4 protein precursor isoform b	membrane-type matrix metallopeptidase-3	RVEAMLND
gi|41406055|ref|NP_958816.1| amyloid beta A4 protein precursor isoform b	membrane-type matrix metallopeptidase-3	VLANMISE

gi|41406055|ref|NP_958816.1| amyloid beta A4 protein precursor isoform b	membrane-type matrix metallopeptidase-3	EVHHQKLV
gi|41406055|ref|NP_958816.1| amyloid beta A4 protein precursor isoform b	membrane-type matrix metallopeptidase-4	EVHHQKLV
gi|41406055|ref|NP_958816.1| amyloid beta A4 protein precursor isoform b	membrane-type matrix metallopeptidase-4	EVHHQKLV
gi|41406055|ref|NP_958816.1| amyloid beta A4 protein precursor isoform b	membrane-type matrix metallopeptidase-4	HHQKLVFF
gi|4503123|ref|NP_001892.1| connective tissue growth factor	matrix metallopeptidase-7	ALAAYRLE
gi|4503123|ref|NP_001892.1| connective tissue growth factor	matrix metallopeptidase-3	ALAAYRLE
gi|4503123|ref|NP_001892.1| connective tissue growth factor	matrix metallopeptidase-7	RANCLVQT
gi|4503123|ref|NP_001892.1| connective tissue growth factor	matrix metallopeptidase-13	RANCLVQT
gi|4503123|ref|NP_001892.1| connective tissue growth factor	membrane-type matrix metallopeptidase-1	ALAAYRLE
gi|115298674|ref|NP_002499.2| nidogen 1 precursor	matrix metallopeptidase-3	VAPPIHQG
gi|115298674|ref|NP_002499.2| nidogen 1 precursor	matrix metallopeptidase-3	EPTTIIRQ
gi|4885587|ref|NP_005399.1| small inducible cytokine A13 precursor	matrix metallopeptidase-3	AQPDALNV
gi|4885587|ref|NP_005399.1| small inducible cytokine A13 precursor	matrix metallopeptidase-3	QPDALNVP
gi|4505205|ref|NP_002416.1| matrix metalloproteinase 10 preproprotein	matrix metallopeptidase-13	GIQSLYGP

gi|4505205|ref|NP_002416.1| matrix metalloproteinase 10 preproprotein	matrix metallopeptidase-3	DTLEVMRK
gi|73760409|ref|NP_001027452.1| tissue factor pathway inhibitor isoform b precursor	matrix metallopeptidase-7	PPLKLMHS
gi|73760409|ref|NP_001027452.1| tissue factor pathway inhibitor isoform b precursor	matrix metallopeptidase-9	PPLKLMHS
gi|41406057|ref|NP_958817.1| amyloid beta A4 protein precursor isoform c	matrix metallopeptidase-9	NKGAIIGL
gi|41406057|ref|NP_958817.1| amyloid beta A4 protein precursor isoform c	matrix metallopeptidase-9	AIIGLMVG
gi|41406057|ref|NP_958817.1| amyloid beta A4 protein precursor isoform c	matrix metallopeptidase-9	IIGLMVGG
gi|41406057|ref|NP_958817.1| amyloid beta A4 protein precursor isoform c	matrix metallopeptidase-9	LVFFAEDV
gi|41406057|ref|NP_958817.1| amyloid beta A4 protein precursor isoform c	matrix metallopeptidase-9	FAEDVGSN
gi|41406057|ref|NP_958817.1| amyloid beta A4 protein precursor isoform c	membrane-type matrix metallopeptidase-3	RVEAMLND
gi|41406057|ref|NP_958817.1| amyloid beta A4 protein precursor isoform c	membrane-type matrix metallopeptidase-3	VLANMISE
gi|41406057|ref|NP_958817.1| amyloid beta A4 protein precursor isoform c	membrane-type matrix metallopeptidase-3	EVHHQKLV
gi|41406057|ref|NP_958817.1| amyloid beta A4 protein precursor isoform c	membrane-type matrix metallopeptidase-4	EVHHQKLV
gi|41406057|ref|NP_958817.1| amyloid beta A4 protein precursor isoform c	membrane-type matrix metallopeptidase-4	EVHHQKLV
gi|41406057|ref|NP_958817.1| amyloid beta A4 protein precursor isoform c	membrane-type matrix metallopeptidase-4	HHQKLVFF

gi|11761629|ref|NP_068657.1| fibrinogen alpha polypeptide isoform alpha preproprotein	matrix metallopeptidase-12	MLGEFVSE
gi|11761629|ref|NP_068657.1| fibrinogen alpha polypeptide isoform alpha preproprotein	matrix metallopeptidase-12	HTEKLVTS
gi|11761629|ref|NP_068657.1| fibrinogen alpha polypeptide isoform alpha preproprotein	matrix metallopeptidase-13	GDKELRTG
gi|11761629|ref|NP_068657.1| fibrinogen alpha polypeptide isoform alpha preproprotein	membrane-type matrix metallopeptidase-1	HTEKLVTS
gi|11761629|ref|NP_068657.1| fibrinogen alpha polypeptide isoform alpha preproprotein	membrane-type matrix metallopeptidase-1	DSHSLTTN
gi|11761629|ref|NP_068657.1| fibrinogen alpha polypeptide isoform alpha preproprotein	matrix metallopeptidase-8	TAWTADSG
gi|11761629|ref|NP_068657.1| fibrinogen alpha polypeptide isoform alpha preproprotein	matrix metallopeptidase-12	TAWTADSG
gi|11761629|ref|NP_068657.1| fibrinogen alpha polypeptide isoform alpha preproprotein	matrix metallopeptidase-13	TAWTADSG
gi|11761629|ref|NP_068657.1| fibrinogen alpha polypeptide isoform alpha preproprotein	matrix metallopeptidase-26	GDKELRTG
gi|11761629|ref|NP_068657.1| fibrinogen alpha polypeptide isoform alpha preproprotein	matrix metallopeptidase-26	HTEKLVTS
gi|91598939|ref|NP_001035149.1| secreted phosphoprotein 1 isoform c	matrix metallopeptidase-3	VVYGLRSK
gi|91598939|ref|NP_001035149.1| secreted phosphoprotein 1 isoform c	matrix metallopeptidase-7	VAQDLNAP
gi|189163540|ref|NP_001121178.1| serine proteinase inhibitor clade A member 1	matrix metallopeptidase-7	GAMFLEAI
gi|189163540|ref|NP_001121178.1| serine proteinase inhibitor clade A member 1	matrix metallopeptidase-8	GAMFLEAI
gi|189163540|ref|NP_001121178.1| serine proteinase inhibitor clade A member 1	matrix metallopeptidase-8	EAIPMSIP

gi|189163540|ref|NP_001121178.1| serine proteinase inhibitor clade A member 1	matrix metallopeptidase-9	GAMFLEAI
gi|189163540|ref|NP_001121178.1| serine proteinase inhibitor clade A member 1	matrix metallopeptidase-9	EAIPMSIP
gi|189163540|ref|NP_001121178.1| serine proteinase inhibitor clade A member 1	matrix metallopeptidase-11	AAGAMFLE
gi|189163540|ref|NP_001121178.1| serine proteinase inhibitor clade A member 1	matrix metallopeptidase-7	EAIPMSIP
gi|189163540|ref|NP_001121178.1| serine proteinase inhibitor clade A member 1	matrix metallopeptidase-12	RPFEVKDT
gi|189163540|ref|NP_001121178.1| serine proteinase inhibitor clade A member 1	matrix metallopeptidase-12	GAMFLEAI
gi|189163540|ref|NP_001121178.1| serine proteinase inhibitor clade A member 1	matrix metallopeptidase-12	EAIPMSIP
gi|189163540|ref|NP_001121178.1| serine proteinase inhibitor clade A member 1	membrane-type matrix metallopeptidase-6	GAMFLEAI
gi|189163540|ref|NP_001121178.1| serine proteinase inhibitor clade A member 1	membrane-type matrix metallopeptidase-6	EAIPMSIP
gi|189163540|ref|NP_001121178.1| serine proteinase inhibitor clade A member 1	matrix metallopeptidase-26	GAMFLEAI
gi|189163540|ref|NP_001121178.1| serine proteinase inhibitor clade A member 1	matrix metallopeptidase-26	EAIPMSIP
gi|189163540|ref|NP_001121178.1| serine proteinase inhibitor clade A member 1	matrix metallopeptidase-3	EAIPMSIP
gi|4557739|ref|NP_000233.1| soluble mannose-binding lectin precursor	matrix metallopeptidase-2	GPPGKLGP
gi|4557739|ref|NP_000233.1| soluble mannose-binding lectin precursor	matrix metallopeptidase-2	GPKGQKGD
gi|4557739|ref|NP_000233.1| soluble mannose-binding lectin precursor	matrix metallopeptidase-9	GPKGQKGD

gi|4557739|ref|NP_000233.1| soluble mannose-binding lectin precursor	membrane-type matrix metallopeptidase-1	GLRGLQGP
gi|4505863|ref|NP_002649.1| urokinase plasminogen activator preproprotein	matrix metallopeptidase-3	PPEELKFQ
gi|4505863|ref|NP_002649.1| urokinase plasminogen activator preproprotein	matrix metallopeptidase-7	PPEELKFQ
gi|17986275|ref|NP_524146.1| fast skeletal myosin alkali light chain 1 isoform 3f	matrix metallopeptidase-2	CINYEAFV
gi|109148522|ref|NP_001035835.1| insulin- insulin-like growth factor 2	matrix metallopeptidase-9	LVEALYLV
gi|109148522|ref|NP_001035835.1| insulin- insulin-like growth factor 2	matrix metallopeptidase-9	GERGFFYT
gi|109148522|ref|NP_001035835.1| insulin- insulin-like growth factor 2	matrix metallopeptidase-9	VNQHLCGS
gi|109148522|ref|NP_001035835.1| insulin- insulin-like growth factor 2	matrix metallopeptidase-9	EALYLVCG
gi|109148522|ref|NP_001035835.1| insulin- insulin-like growth factor 2	matrix metallopeptidase-3	LVEALYLV
gi|109148522|ref|NP_001035835.1| insulin- insulin-like growth factor 2	matrix metallopeptidase-7	LVEALYLV
gi|110611233|ref|NP_569712.2| alpha 1 type XVIII collagen isoform 2 precursor	matrix metallopeptidase-7	RADDILAS
gi|110611233|ref|NP_569712.2| alpha 1 type XVIII collagen isoform 2 precursor	matrix metallopeptidase-9	TARPWRAD
gi|110611233|ref|NP_569712.2| alpha 1 type XVIII collagen isoform 2 precursor	matrix metallopeptidase-3	PPAHSHRD
gi|110611233|ref|NP_569712.2| alpha 1 type XVIII collagen isoform 2 precursor	matrix metallopeptidase-3	HHSSYVHL
gi|110611233|ref|NP_569712.2| alpha 1 type XVIII collagen isoform 2 precursor	matrix metallopeptidase-13	APHHSSYV
gi|110611233|ref|NP_569712.2| alpha 1 type XVIII collagen isoform 2 precursor	matrix metallopeptidase-13	HHSSYVHL

gi|110611233|ref|NP_569712.2| alpha 1 type XVIII collagen isoform 2 precursor	matrix metallopeptidase-20	HHSSYVHL
gi|110611233|ref|NP_569712.2| alpha 1 type XVIII collagen isoform 2 precursor	membrane-type matrix metallopeptidase-1	HHSSYVHL
gi|189163530|ref|NP_001121173.1| serine proteinase inhibitor clade A member 1	matrix metallopeptidase-7	GAMFLEAI
gi|189163530|ref|NP_001121173.1| serine proteinase inhibitor clade A member 1	matrix metallopeptidase-8	GAMFLEAI
gi|189163530|ref|NP_001121173.1| serine proteinase inhibitor clade A member 1	matrix metallopeptidase-8	EAIPMSIP
gi|189163530|ref|NP_001121173.1| serine proteinase inhibitor clade A member 1	matrix metallopeptidase-9	GAMFLEAI
gi|189163530|ref|NP_001121173.1| serine proteinase inhibitor clade A member 1	matrix metallopeptidase-9	EAIPMSIP
gi|189163530|ref|NP_001121173.1| serine proteinase inhibitor clade A member 1	matrix metallopeptidase-11	AAGAMFLE
gi|189163530|ref|NP_001121173.1| serine proteinase inhibitor clade A member 1	matrix metallopeptidase-7	EAIPMSIP
gi|189163530|ref|NP_001121173.1| serine proteinase inhibitor clade A member 1	matrix metallopeptidase-12	RPFEVKDT
gi|189163530|ref|NP_001121173.1| serine proteinase inhibitor clade A member 1	matrix metallopeptidase-12	GAMFLEAI
gi|189163530|ref|NP_001121173.1| serine proteinase inhibitor clade A member 1	matrix metallopeptidase-12	EAIPMSIP
gi|189163530|ref|NP_001121173.1| serine proteinase inhibitor clade A member 1	membrane-type matrix metallopeptidase-6	GAMFLEAI
gi|189163530|ref|NP_001121173.1| serine proteinase inhibitor clade A member 1	membrane-type matrix metallopeptidase-6	EAIPMSIP
gi|189163530|ref|NP_001121173.1| serine proteinase inhibitor clade A member 1	matrix metallopeptidase-26	GAMFLEAI

gi|189163530|ref|NP_001121173.1| serine proteinase inhibitor clade A member 1	matrix metallopeptidase-26	EAIPMSIP
gi|189163530|ref|NP_001121173.1| serine proteinase inhibitor clade A member 1	matrix metallopeptidase-3	EAIPMSIP
gi|4504603|ref|NP_002167.1| interferon beta 1 fibroblast	matrix metallopeptidase-9	MSYNLLGF
gi|4504603|ref|NP_002167.1| interferon beta 1 fibroblast	matrix metallopeptidase-9	NVYHQINH
gi|62243248|ref|NP_001013416.1| insulin-like growth factor binding protein 3 isoform a precursor	matrix metallopeptidase-3	APGNASES
gi|62243248|ref|NP_001013416.1| insulin-like growth factor binding protein 3 isoform a precursor	matrix metallopeptidase-7	AVSRLRAY
gi|62243248|ref|NP_001013416.1| insulin-like growth factor binding protein 3 isoform a precursor	matrix metallopeptidase-1	HPLHSKII
gi|62243248|ref|NP_001013416.1| insulin-like growth factor binding protein 3 isoform a precursor	matrix metallopeptidase-2	SRLRAYLL
gi|62243248|ref|NP_001013416.1| insulin-like growth factor binding protein 3 isoform a precursor	matrix metallopeptidase-2	HPLHSKII
gi|62243248|ref|NP_001013416.1| insulin-like growth factor binding protein 3 isoform a precursor	matrix metallopeptidase-2	VSRLRAYL
gi|62243248|ref|NP_001013416.1| insulin-like growth factor binding protein 3 isoform a precursor	matrix metallopeptidase-2	FHPLHSKI
gi|17986273|ref|NP_524144.1| fast skeletal myosin alkali light chain 1 isoform 1f	matrix metallopeptidase-2	CINYEAFV
gi|110349772|ref|NP_000079.2| alpha 1 type I collagen preproprotein	matrix metallopeptidase-2	GPQGIAGQ
gi|110349772|ref|NP_000079.2| alpha 1 type I collagen preproprotein	membrane-type matrix metallopeptidase-1	GPQGIAGQ
gi|110349772|ref|NP_000079.2| alpha 1 type I collagen preproprotein	matrix metallopeptidase-9	GPAGEEGK
gi|4505981|ref|NP_002695.1| pro-platelet basic protein precursor	matrix metallopeptidase-9	HPKNIQSL

gi|4505981|ref|NP_002695.1| pro-platelet basic protein precursor	matrix metallopeptidase-9	VIATLKDG
gi|68509928|ref|NP_001020261.1| myelin basic protein isoform 3	matrix metallopeptidase-9	LSRFSWGA
gi|4505217|ref|NP_002413.1| matrix metalloproteinase 3 preproprotein	matrix metallopeptidase-13	GIQSLYGP
gi|4505217|ref|NP_002413.1| matrix metalloproteinase 3 preproprotein	matrix metallopeptidase-3	DVGHFRTF
gi|4505217|ref|NP_002413.1| matrix metalloproteinase 3 preproprotein	matrix metallopeptidase-3	DTLEVMRK
gi|4503413|ref|NP_001936.1| heparin-binding EGF-like growth factor	membrane-type matrix metallopeptidase-1	LSLPVENR
gi|4503413|ref|NP_001936.1| heparin-binding EGF-like growth factor	membrane-type matrix metallopeptidase-4	LPVENRLY
gi|4503413|ref|NP_001936.1| heparin-binding EGF-like growth factor	membrane-type matrix metallopeptidase-4	VENRLYTY
gi|4503413|ref|NP_001936.1| heparin-binding EGF-like growth factor	matrix metallopeptidase-3	LPVENRLY
gi|4503413|ref|NP_001936.1| heparin-binding EGF-like growth factor	membrane-type matrix metallopeptidase-1	LPVENRLY
gi|4503413|ref|NP_001936.1| heparin-binding EGF-like growth factor	membrane-type matrix metallopeptidase-4	VENRLYTY
gi|4503413|ref|NP_001936.1| heparin-binding EGF-like growth factor	membrane-type matrix metallopeptidase-4	LPVENRLY
gi|4557321|ref|NP_000030.1| apolipoprotein A-I preproprotein	matrix metallopeptidase-7	ALEDLRQG
gi|4557321|ref|NP_000030.1| apolipoprotein A-I preproprotein	matrix metallopeptidase-12	ALEDLRQG

gi|4557321|ref|NP_000030.1| apolipoprotein A-I preproprotein	matrix metallopeptidase-12	RLAEYHAK
gi|4557321|ref|NP_000030.1| apolipoprotein A-I preproprotein	matrix metallopeptidase-12	KVEPLRAE
gi|4557321|ref|NP_000030.1| apolipoprotein A-I preproprotein	matrix metallopeptidase-3	RLAEYHAK
gi|4557038|ref|NP_002467.1| atrial/embryonic alkali myosin light chain	matrix metallopeptidase-2	CINYEAFV
gi|6806898|ref|NP_009292.1| alpha-synuclein isoform NACP112	matrix metallopeptidase-3	TVAEKTKE
gi|6806898|ref|NP_009292.1| alpha-synuclein isoform NACP112	matrix metallopeptidase-3	GVATVAEK
gi|48255939|ref|NP_001001390.1| CD44 antigen isoform 3 precursor	membrane-type matrix metallopeptidase-1	GEYRTNPE
gi|48255939|ref|NP_001001390.1| CD44 antigen isoform 3 precursor	membrane-type matrix metallopeptidase-1	SSERSSTS
gi|32130518|ref|NP_000474.2| apolipoprotein C-II precursor	matrix metallopeptidase-7	AAQNLYEK
gi|32130518|ref|NP_000474.2| apolipoprotein C-II precursor	membrane-type matrix metallopeptidase-1	AAQNLYEK
gi|4557777|ref|NP_000249.1| slow skeletal ventricular myosin alkali light chain 3	matrix metallopeptidase-2	CINYEAFV
gi|4504153|ref|NP_001502.1| chemokine (C-X-C motif) ligand 1	matrix metallopeptidase-9	QSVNVKSP
gi|4504153|ref|NP_001502.1| chemokine (C-X-C motif) ligand 1	matrix metallopeptidase-9	VATELRCQ
gi|66346695|ref|NP_001990.2| fibrillin 2 precursor	matrix metallopeptidase-2	IITGLTIL
gi|66346695|ref|NP_001990.2| fibrillin 2 precursor	matrix metallopeptidase-2	ITGLTILN

gi|66346695|ref|NP_001990.2| fibrillin 2 precursor	matrix metallopeptidase-9	ITGLTILN
gi|66346695|ref|NP_001990.2| fibrillin 2 precursor	matrix metallopeptidase-12	ITGLTILN
gi|66346695|ref|NP_001990.2| fibrillin 2 precursor	matrix metallopeptidase-13	IITGLTIL
gi|89363017|ref|NP_000384.2| alpha 2 type V collagen preproprotein	matrix metallopeptidase-9	GHRGFTGL
gi|89363017|ref|NP_000384.2| alpha 2 type V collagen preproprotein	matrix metallopeptidase-9	GPPGLQGM
gi|68509930|ref|NP_001020252.1| myelin basic protein isoform 1	matrix metallopeptidase-9	LSRFSWGA
gi|4504763|ref|NP_002201.1| integrin alpha-V precursor	membrane-type matrix metallopeptidase-1	TKRDLALS
gi|100913220|ref|NP_001847.3| alpha 1 type XVI collagen precursor	matrix metallopeptidase-2	GPKGQKGD
gi|100913220|ref|NP_001847.3| alpha 1 type XVI collagen precursor	matrix metallopeptidase-9	GPKGQKGD
gi|4502261|ref|NP_000479.1| serine (or cysteine) proteinase inhibitor clade C (antithrombin) member 1	matrix metallopeptidase-3	IAGRSLNP
gi|98985806|ref|NP_001845.3| alpha 1 type XI collagen isoform A preproprotein	matrix metallopeptidase-9	GPGGVVGP
gi|122937273|ref|NP_001073909.1| hypothetical protein LOC131149	matrix metallopeptidase-9	GPPGVVGP
gi|98985808|ref|NP_542197.2| alpha 1 type XI collagen isoform C preproprotein	matrix metallopeptidase-9	GPGGVVGP
gi|19743854|ref|NP_598014.1| decorin isoform e precursor	matrix metallopeptidase-7	MLEDEASG
gi|40316924|ref|NP_954637.1| chemokine (C-X-C motif) ligand 12 (stromal cell-derived factor 1) isoform alpha	matrix metallopeptidase-9	KPVSLSYR
gi|47778921|ref|NP_001849.2| alpha 1 type XIX collagen precursor	matrix metallopeptidase-2	GPKGQKGE

gi|50363221|ref|NP_001002235.1| serine proteinase inhibitor clade A member 1	matrix metallopeptidase-7	GAMFLEAI
gi|50363221|ref|NP_001002235.1| serine proteinase inhibitor clade A member 1	matrix metallopeptidase-8	GAMFLEAI
gi|50363221|ref|NP_001002235.1| serine proteinase inhibitor clade A member 1	matrix metallopeptidase-8	EAIPMSIP
gi|50363221|ref|NP_001002235.1| serine proteinase inhibitor clade A member 1	matrix metallopeptidase-9	GAMFLEAI
gi|50363221|ref|NP_001002235.1| serine proteinase inhibitor clade A member 1	matrix metallopeptidase-9	EAIPMSIP
gi|50363221|ref|NP_001002235.1| serine proteinase inhibitor clade A member 1	matrix metallopeptidase-11	AAGAMFLE
gi|50363221|ref|NP_001002235.1| serine proteinase inhibitor clade A member 1	matrix metallopeptidase-7	EAIPMSIP
gi|50363221|ref|NP_001002235.1| serine proteinase inhibitor clade A member 1	matrix metallopeptidase-12	RPFEVKDT
gi|50363221|ref|NP_001002235.1| serine proteinase inhibitor clade A member 1	matrix metallopeptidase-12	GAMFLEAI
gi|50363221|ref|NP_001002235.1| serine proteinase inhibitor clade A member 1	matrix metallopeptidase-12	EAIPMSIP
gi|50363221|ref|NP_001002235.1| serine proteinase inhibitor clade A member 1	membrane-type matrix metallopeptidase-6	GAMFLEAI
gi|50363221|ref|NP_001002235.1| serine proteinase inhibitor clade A member 1	membrane-type matrix metallopeptidase-6	EAIPMSIP
gi|50363221|ref|NP_001002235.1| serine proteinase inhibitor clade A member 1	matrix metallopeptidase-26	GAMFLEAI
gi|50363221|ref|NP_001002235.1| serine proteinase inhibitor clade A member 1	matrix metallopeptidase-26	EAIPMSIP
gi|50363221|ref|NP_001002235.1| serine proteinase inhibitor clade A member 1	matrix metallopeptidase-3	EAIPMSIP

gi|4557325|ref|NP_000032.1| apolipoprotein E precursor	membrane-type matrix metallopeptidase-1	MDETMKEL
gi|4557325|ref|NP_000032.1| apolipoprotein E precursor	membrane-type matrix metallopeptidase-1	EQVAEVRA
gi|21264602|ref|NP_005551.3| laminin alpha 5	membrane-type matrix metallopeptidase-1	DPFRLQCT
gi|4505219|ref|NP_002414.1| matrix metalloproteinase 7 preproprotein	matrix metallopeptidase-3	DVAEYSLF
gi|4507341|ref|NP_003173.1| tachykinin 1 isoform beta precursor	matrix metallopeptidase-3	KPQQFFGL
gi|48255941|ref|NP_001001391.1| CD44 antigen isoform 4 precursor	membrane-type matrix metallopeptidase-1	GEYRTNPE
gi|48255941|ref|NP_001001391.1| CD44 antigen isoform 4 precursor	membrane-type matrix metallopeptidase-1	SSERSSTS
gi|66932947|ref|NP_000005.2| alpha-2-macroglobulin precursor	matrix metallopeptidase-2	GHARLVHV
gi|66932947|ref|NP_000005.2| alpha-2-macroglobulin precursor	matrix metallopeptidase-2	GPEGLRVG
gi|66932947|ref|NP_000005.2| alpha-2-macroglobulin precursor	matrix metallopeptidase-3	GPEGLRVG
gi|66932947|ref|NP_000005.2| alpha-2-macroglobulin precursor	matrix metallopeptidase-3	RVGFYESD
gi|19743852|ref|NP_598013.1| decorin isoform d precursor	matrix metallopeptidase-7	MLEDEASG
gi|7770079|ref|NP_054704.1| tachykinin 1 isoform delta precursor	matrix metallopeptidase-3	KPQQFFGL
gi|10834978|ref|NP_000575.1| interleukin 8 precursor	membrane-type matrix metallopeptidase-1	VLPRSAKE

gi|10834978|ref|NP_000575.1| interleukin 8 precursor	matrix metallopeptidase-9	LPRSAKEL
gi|76563933|ref|NP_001029058.1| chemokine (C-X-C motif) ligand 12 (stromal cell-derived factor 1) isoform gamma	matrix metallopeptidase-9	KPVSLSYR
gi|51873055|ref|NP_001989.2| fibulin 2 precursor isoform b	matrix metallopeptidase-3	PPAPVQAK
gi|4505881|ref|NP_000292.1| plasminogen	matrix metallopeptidase-3	CAAPSFDC
gi|4505881|ref|NP_000292.1| plasminogen	matrix metallopeptidase-9	VAPPPVVL
gi|4505881|ref|NP_000292.1| plasminogen	matrix metallopeptidase-7	APPPVVLL
gi|62243068|ref|NP_000589.2| insulin-like growth factor binding protein 3 isoform b precursor	matrix metallopeptidase-3	APGNASES
gi|62243068|ref|NP_000589.2| insulin-like growth factor binding protein 3 isoform b precursor	matrix metallopeptidase-7	AVSRLRAY
gi|62243068|ref|NP_000589.2| insulin-like growth factor binding protein 3 isoform b precursor	matrix metallopeptidase-1	HPLHSKII
gi|62243068|ref|NP_000589.2| insulin-like growth factor binding protein 3 isoform b precursor	matrix metallopeptidase-2	SRLRAYLL
gi|62243068|ref|NP_000589.2| insulin-like growth factor binding protein 3 isoform b precursor	matrix metallopeptidase-2	HPLHSKII
gi|62243068|ref|NP_000589.2| insulin-like growth factor binding protein 3 isoform b precursor	matrix metallopeptidase-2	VSRLRAYL
gi|62243068|ref|NP_000589.2| insulin-like growth factor binding protein 3 isoform b precursor	matrix metallopeptidase-2	FHPLHSKI
gi|4506851|ref|NP_002984.1| chemokine (C-X-C motif) ligand 6 (granulocyte chemotactic protein 2)	matrix metallopeptidase-9	GPVSAVLT
gi|19743850|ref|NP_598012.1| decorin isoform c precursor	matrix metallopeptidase-3	DAASLKGL
gi|19743850|ref|NP_598012.1| decorin isoform c precursor	matrix metallopeptidase-7	MLEDEASG

gi|19743850|ref|NP_598012.1| decorin isoform c precursor	matrix metallopeptidase-7	HLRELHLD
gi|10834988|ref|NP_000600.1| chemokine (C-X-C motif) ligand 12 (stromal cell-derived factor 1) isoform beta	matrix metallopeptidase-9	KPVSLSYR
gi|4507109|ref|NP_000336.1| alpha-synuclein isoform NACP140	matrix metallopeptidase-3	TVAEKTKE
gi|4507109|ref|NP_000336.1| alpha-synuclein isoform NACP140	matrix metallopeptidase-3	GVATVAEK
gi|73808266|ref|NP_068573.2| matrix metalloproteinase 26 preproprotein	matrix metallopeptidase-26	QMHALLHQ
gi|73808266|ref|NP_068573.2| matrix metalloproteinase 26 preproprotein	matrix metallopeptidase-26	SPLLTQET
gi|73808266|ref|NP_068573.2| matrix metalloproteinase 26 preproprotein	matrix metallopeptidase-26	QLLQQFHR
gi|50659080|ref|NP_001076.2| serpin peptidase inhibitor clade A member 3 precursor	matrix metallopeptidase-1	LLSALVET
gi|50659080|ref|NP_001076.2| serpin peptidase inhibitor clade A member 3 precursor	matrix metallopeptidase-3	LLSALVET
gi|50659080|ref|NP_001076.2| serpin peptidase inhibitor clade A member 3 precursor	matrix metallopeptidase-13	LLSALVET
gi|111118974|ref|NP_149162.2| collagen type II alpha 1 isoform 2 precursor	matrix metallopeptidase-7	EPGDIKDI
gi|111118976|ref|NP_001835.3| collagen type II alpha 1 isoform 1 precursor	matrix metallopeptidase-7	EPGDIKDI
gi|4504603|ref|NP_002167.1| interferon beta 1 fibroblast	matrix metallopeptidase-9	IVENLLAN
gi|6806898|ref|NP_009292.1| alpha-synuclein isoform NACP112	matrix metallopeptidase-3	AVAQKTVE
gi|4507109|ref|NP_000336.1| alpha-synuclein isoform NACP140	matrix metallopeptidase-3	AVAQKTVE
gi|89276751|ref|NP_000084.3| alpha 1 type V collagen preproprotein	procollagen C-peptidase	QLLDDGNG
gi|89276751|ref|NP_000084.3| alpha 1 type V collagen preproprotein	procollagen C-peptidase	TPQSQDPN

gi|15451844|ref|NP_150377.1| ADAM metallopeptidase domain 19 isoform 2 preproprotein	adamalysin-19	RPLESNAV
gi|60498997|ref|NP_001012530.1| gastrin-releasing peptide isoform 2 preproprotein	meprin beta subunit	MYPRGNHW
gi|19743848|ref|NP_598011.1| decorin isoform b precursor	procollagen C-peptidase	FMLEDEAS
gi|111118974|ref|NP_149162.2| collagen type II alpha 1 isoform 2 precursor	procollagen I N-peptidase	NFAAQMAG
gi|111118974|ref|NP_149162.2| collagen type II alpha 1 isoform 2 precursor	procollagen C-peptidase	YMRADQAA
gi|48762934|ref|NP_000080.2| alpha 2 type I collagen	procollagen C-peptidase	FYRADQPR
gi|60498999|ref|NP_001012531.1| gastrin-releasing peptide isoform 3 preproprotein	meprin beta subunit	MYPRGNHW
gi|67782346|ref|NP_005567.2| lysyl oxidase-like 1 preproprotein	procollagen C-peptidase	VAVGDSTG
gi|4503271|ref|NP_001911.1| decorin isoform a preproprotein	procollagen C-peptidase	FMLEDEAS
gi|111118976|ref|NP_001835.3| collagen type II alpha 1 isoform 1 precursor	procollagen I N-peptidase	NFAAQMAG
gi|111118976|ref|NP_001835.3| collagen type II alpha 1 isoform 1 precursor	procollagen C-peptidase	YMRADQAA
gi|4557671|ref|NP_000198.1| proinsulin precursor	meprin beta subunit	LVEALYLV
gi|4557671|ref|NP_000198.1| proinsulin precursor	adamalysin-19	LVEALYLV
gi|19743846|ref|NP_598010.1| decorin isoform a preproprotein	procollagen C-peptidase	FMLEDEAS
gi|4503273|ref|NP_000780.1| angiotensin I converting enzyme isoform 1 precursor	ADAM17 peptidase	AGQRLATA
gi|4503273|ref|NP_000780.1| angiotensin I converting enzyme isoform 1 precursor	ADAM17 peptidase	NSARSEGP
gi|4507461|ref|NP_003227.1| transforming growth factor alpha isoform 1	ADAM17 peptidase	VAAAVVSH
gi|4507461|ref|NP_003227.1| transforming growth factor alpha isoform 1	ADAM17 peptidase	DLLAVVAA
gi|113415639|ref|XP_001125684.1| PREDICTED: similar to Amphiregulin precursor (AR) (Colorectum cell-derived growth factor) (CRDGF)	ADAM17 peptidase	RVEQVVKP
gi|113415639|ref|XP_001125684.1| PREDICTED: similar to Amphiregulin precursor (AR) (Colorectum cell-derived growth factor) (CRDGF)	ADAM17 peptidase	EYDNEPQI
gi|4758598|ref|NP_004624.1| interleukin 1 receptor type II precursor	ADAM8 peptidase	KEASSTFS
gi|30089980|ref|NP_003732.2| chordin	procollagen C-peptidase	PMQADGPR
gi|30089980|ref|NP_003732.2| chordin	procollagen C-peptidase	RSYSDRGE
gi|30089980|ref|NP_003732.2| chordin	mammalian tolloid-like 1 protein	PMQADGPR

gi|30089980|ref|NP_003732.2| chordin	mammalian tolloid-like 1 protein	RSYSDRGE
gi|153791414|ref|NP_001093161.1| transforming growth factor alpha isoform 2	ADAM17 peptidase	VAAAVVSH
gi|153791414|ref|NP_001093161.1| transforming growth factor alpha isoform 2	ADAM17 peptidase	DLLAVVAA
gi|21361116|ref|NP_004376.2| versican isoform 1	ADAMTS4 peptidase	EAAEARRG
gi|20149540|ref|NP_002308.2| lysyl oxidase preproprotein	procollagen C-peptidase	GMVGDDPY
gi|20149540|ref|NP_002308.2| lysyl oxidase preproprotein	mammalian tolloid-like 1 protein	GMVGDDPY
gi|89191868|ref|NP_000543.2| von Willebrand factor preproprotein	ADAMTS13 peptidase	NLVYMVTG
gi|27894334|ref|NP_775465.1| interleukin 1 receptor type II precursor	ADAM8 peptidase	KEASSTFS
gi|4503923|ref|NP_000796.1| gastrin preproprotein	meprin beta subunit	GPWLEEEE
gi|4503923|ref|NP_000796.1| gastrin preproprotein	meprin beta subunit	PWLEEEEE
gi|4503923|ref|NP_000796.1| gastrin preproprotein	meprin beta subunit	WLEEEEEA
gi|4503923|ref|NP_000796.1| gastrin preproprotein	meprin beta subunit	LEEEEEAY
gi|4505449|ref|NP_000896.1| neuropeptide Y	meprin beta subunit	NPGEDAPA
gi|4505449|ref|NP_000896.1| neuropeptide Y	meprin beta subunit	APAEDMAR
gi|15451842|ref|NP_075525.2| ADAM metallopeptidase domain 19 isoform 1 preproprotein	adamalysin-19	RPLESNAV
gi|4557433|ref|NP_000065.1| CD40 ligand	ADAM10 peptidase	SFEMQKGD
gi|4885259|ref|NP_005250.1| myostatin	procollagen C-peptidase	DVQRDDSS
gi|4885259|ref|NP_005250.1| myostatin	mammalian tolloid-like 1 protein	DVQRDDSS
gi|4502951|ref|NP_000081.1| collagen type III alpha 1 preproprotein	procollagen C-peptidase	PYYGDEPM
gi|126090883|ref|NP_001126.2| aggrecan isoform 1 precursor	ADAMTS4 peptidase	KEEEGLGS
gi|126090883|ref|NP_001126.2| aggrecan isoform 1 precursor	ADAMTS4 peptidase	TAQEAGEG
gi|126090883|ref|NP_001126.2| aggrecan isoform 1 precursor	ADAMTS4 peptidase	TEGEARGS
gi|126090883|ref|NP_001126.2| aggrecan isoform 1 precursor	ADAMTS1 peptidase	KEEEGLGS
gi|126090883|ref|NP_001126.2| aggrecan isoform 1 precursor	ADAMTS5 peptidase	TEGEARGS
gi|126090883|ref|NP_001126.2| aggrecan isoform 1 precursor	ADAMTS4 peptidase	IPENFFGV

gi|126090883|ref|NP_001126.2| aggrecan isoform 1 precursor	ADAMTS1 peptidase	SELEGRGT
gi|126090883|ref|NP_001126.2| aggrecan isoform 1 precursor	ADAMTS1 peptidase	ISQELGQR
gi|126090883|ref|NP_001126.2| aggrecan isoform 1 precursor	ADAMTS1 peptidase	TEGEARGS
gi|126090883|ref|NP_001126.2| aggrecan isoform 1 precursor	ADAMTS5 peptidase	TEGEARGS
gi|126090892|ref|NP_037359.2| aggrecan isoform 2 precursor	ADAMTS4 peptidase	KEEEGLGS
gi|126090892|ref|NP_037359.2| aggrecan isoform 2 precursor	ADAMTS4 peptidase	TAQEAGEG
gi|126090892|ref|NP_037359.2| aggrecan isoform 2 precursor	ADAMTS4 peptidase	TEGEARGS
gi|126090892|ref|NP_037359.2| aggrecan isoform 2 precursor	ADAMTS1 peptidase	KEEEGLGS
gi|126090892|ref|NP_037359.2| aggrecan isoform 2 precursor	ADAMTS5 peptidase	TEGEARGS
gi|126090892|ref|NP_037359.2| aggrecan isoform 2 precursor	ADAMTS4 peptidase	IPENFFGV
gi|126090892|ref|NP_037359.2| aggrecan isoform 2 precursor	ADAMTS1 peptidase	SELEGRGT
gi|126090892|ref|NP_037359.2| aggrecan isoform 2 precursor	ADAMTS1 peptidase	ISQELGQR
gi|126090892|ref|NP_037359.2| aggrecan isoform 2 precursor	ADAMTS1 peptidase	TEGEARGS
gi|126090892|ref|NP_037359.2| aggrecan isoform 2 precursor	ADAMTS5 peptidase	TEGEARGS
gi|25952111|ref|NP_000585.2| tumor necrosis factor alpha	ADAM17 peptidase	LAQAVRSS
gi|25952111|ref|NP_000585.2| tumor necrosis factor alpha	ADAM17 peptidase	LAQAVRSS
gi|25952111|ref|NP_000585.2| tumor necrosis factor alpha	ADAM10 peptidase	LAQAVRSS
gi|25952111|ref|NP_000585.2| tumor necrosis factor alpha	ADAM8 peptidase	SPLAQAVR
gi|25952111|ref|NP_000585.2| tumor necrosis factor alpha	ADAM8 peptidase	LAQAVRSS
gi|25952111|ref|NP_000585.2| tumor necrosis factor alpha	ADAM9 peptidase	SPLAQAVR
gi|25952111|ref|NP_000585.2| tumor necrosis factor alpha	ADAM9 peptidase	SPLAQAVR
gi|25952111|ref|NP_000585.2| tumor necrosis factor alpha	ADAM9 peptidase	AVRSSSRT
gi|25952111|ref|NP_000585.2| tumor necrosis factor alpha	ADAM10 peptidase	LAQAVRSS
gi|25952111|ref|NP_000585.2| tumor necrosis factor alpha	ADAM17 peptidase	LAQAVRSS

gi|25952111|ref|NP_000585.2| tumor necrosis factor alpha	ADAM8 peptidase	SPLAQAVR
gi|25952111|ref|NP_000585.2| tumor necrosis factor alpha	ADAM8 peptidase	LAQAVRSS
gi|14790152|ref|NP_143026.1| tumor necrosis factor ligand superfamily member 11 isoform 2	ADAM33 peptidase	GSQHIRAE
gi|4502961|ref|NP_000085.1| alpha 1 type VII collagen precursor	procollagen C-peptidase	SYAADTAG
gi|157419138|ref|NP_005553.2| laminin gamma 2 isoform a precursor	procollagen C-peptidase	CYSGDENP
gi|157419140|ref|NP_061486.2| laminin gamma 2 isoform b precursor	procollagen C-peptidase	CYSGDENP
gi|4502167|ref|NP_000475.1| amyloid beta A4 protein precursor isoform a	ADAM17 peptidase	HHQKLVFF
gi|4502167|ref|NP_000475.1| amyloid beta A4 protein precursor isoform a	ADAM8 peptidase	EVHHQKLV
gi|4502167|ref|NP_000475.1| amyloid beta A4 protein precursor isoform a	ADAM9 peptidase	EVHHQKLV
gi|4502167|ref|NP_000475.1| amyloid beta A4 protein precursor isoform a	ADAM10 peptidase	HHQKLVFF
gi|4502167|ref|NP_000475.1| amyloid beta A4 protein precursor isoform a	ADAM9 peptidase	EVHHQKLV
gi|4502167|ref|NP_000475.1| amyloid beta A4 protein precursor isoform a	ADAM33 peptidase	EVHHQKLV
gi|4507595|ref|NP_003692.1| tumor necrosis factor ligand superfamily member 11 isoform 1	ADAM33 peptidase	GSQHIRAE
gi|4505175|ref|NP_000890.1| KIT ligand isoform b precursor	ADAM8 peptidase	PVAASSLR
gi|4505175|ref|NP_000890.1| KIT ligand isoform b precursor	ADAM9 peptidase	PPVAASSL
gi|4505175|ref|NP_000890.1| KIT ligand isoform b precursor	ADAM9 peptidase	VAASSLRN
gi|4505175|ref|NP_000890.1| KIT ligand isoform b precursor	adamalysin-19	PVAASSLR
gi|4505175|ref|NP_000890.1| KIT ligand isoform b precursor	ADAM17 peptidase	PVAASSLR
gi|4505175|ref|NP_000890.1| KIT ligand isoform b precursor	ADAM33 peptidase	PVAASSLR
gi|41406055|ref|NP_958816.1| amyloid beta A4 protein precursor isoform b	ADAM17 peptidase	HHQKLVFF
gi|41406055|ref|NP_958816.1| amyloid beta A4 protein precursor isoform b	ADAM8 peptidase	EVHHQKLV
gi|41406055|ref|NP_958816.1| amyloid beta A4 protein precursor isoform b	ADAM9 peptidase	EVHHQKLV
gi|41406055|ref|NP_958816.1| amyloid beta A4 protein precursor isoform b	ADAM10 peptidase	HHQKLVFF
gi|41406055|ref|NP_958816.1| amyloid beta A4 protein precursor isoform b	ADAM9 peptidase	EVHHQKLV

gi|41406055|ref|NP_958816.1| amyloid beta A4 protein precursor isoform b	ADAM33 peptidase	EVHHQKLV
gi|41406057|ref|NP_958817.1| amyloid beta A4 protein precursor isoform c	ADAM17 peptidase	HHQKLVFF
gi|41406057|ref|NP_958817.1| amyloid beta A4 protein precursor isoform c	ADAM8 peptidase	EVHHQKLV
gi|41406057|ref|NP_958817.1| amyloid beta A4 protein precursor isoform c	ADAM9 peptidase	EVHHQKLV
gi|41406057|ref|NP_958817.1| amyloid beta A4 protein precursor isoform c	ADAM10 peptidase	HHQKLVFF
gi|41406057|ref|NP_958817.1| amyloid beta A4 protein precursor isoform c	ADAM9 peptidase	EVHHQKLV
gi|41406057|ref|NP_958817.1| amyloid beta A4 protein precursor isoform c	ADAM33 peptidase	EVHHQKLV
gi|109148522|ref|NP_001035835.1| insulin- insulin-like growth factor 2	meprin beta subunit	LVEALYLV
gi|109148522|ref|NP_001035835.1| insulin- insulin-like growth factor 2	adamalysin-19	LVEALYLV
gi|4507577|ref|NP_001057.1| tumor necrosis factor receptor 2 precursor	ADAM9 peptidase	GAVHLPQP
gi|110349772|ref|NP_000079.2| alpha 1 type I collagen preproprotein	procollagen I N-peptidase	NFAPQLSY
gi|110349772|ref|NP_000079.2| alpha 1 type I collagen preproprotein	procollagen C-peptidase	YYRADDAN
gi|110349772|ref|NP_000079.2| alpha 1 type I collagen preproprotein	procollagen C-peptidase	YYRADDAN
gi|5031613|ref|NP_005802.1| growth differentiation factor 11	procollagen C-peptidase	DFQGDALQ
gi|4503413|ref|NP_001936.1| heparin-binding EGF-like growth factor	ADAM10 peptidase	KKGKGLGK
gi|4503413|ref|NP_001936.1| heparin-binding EGF-like growth factor	ADAM17 peptidase	LSLPVENR
gi|50980307|ref|NP_055949.2| myosin phosphatase-Rho interacting protein isoform 1	ADAM9 peptidase	PPAASSLR
gi|50980301|ref|NP_958431.2| myosin phosphatase-Rho interacting protein isoform 2	ADAM9 peptidase	PPAASSLR
gi|157427675|ref|NP_005090.3| ADAM metallopeptidase with thrombospondin type 1 motif 4 preproprotein	ADAMTS4 peptidase	SFRKFRYG
gi|157427675|ref|NP_005090.3| ADAM metallopeptidase with thrombospondin type 1 motif 4 preproprotein	ADAMTS4 peptidase	SALTFREE
gi|4758172|ref|NP_004398.1| dentin matrix acidic phosphoprotein isoform 1 precursor	procollagen C-peptidase	GMQSDDPE
gi|89363017|ref|NP_000384.2| alpha 2 type V collagen preproprotein	procollagen C-peptidase	EFTEDQAA
gi|4502199|ref|NP_001648.1| amphiregulin preproprotein	ADAM17 peptidase	RVEQVVKP
gi|4502199|ref|NP_001648.1| amphiregulin preproprotein	ADAM17 peptidase	EYDNEPQI

gi|4503945|ref|NP_002045.1| glucagon preproprotein	meprin beta subunit	HSQGTFTS
gi|4503945|ref|NP_002045.1| glucagon preproprotein	meprin beta subunit	TFTSDYSK
gi|4503945|ref|NP_002045.1| glucagon preproprotein	meprin beta subunit	SKYLDSRR
gi|4503945|ref|NP_002045.1| glucagon preproprotein	meprin beta subunit	DSRRAQDF
gi|4503945|ref|NP_002045.1| glucagon preproprotein	meprin beta subunit	RRAQDFVQ
gi|31542860|ref|NP_002082.2| gastrin-releasing peptide isoform 1 preproprotein	meprin beta subunit	MYPRGNHW
gi|23238214|ref|NP_690043.1| angiotensin I converting enzyme isoform 2 precursor	ADAM17 peptidase	AGQRLATA
gi|23238214|ref|NP_690043.1| angiotensin I converting enzyme isoform 2 precursor	ADAM17 peptidase	NSARSEGP
gi|121256621|ref|NP_001073380.1| dentin matrix acidic phosphoprotein isoform 2 precursor	procollagen C-peptidase	GMQSDDPE
gi|19743854|ref|NP_598014.1| decorin isoform e precursor	procollagen C-peptidase	FMLEDEAS
gi|66932947|ref|NP_000005.2| alpha-2-macroglobulin precursor	procollagen C-peptidase	FYESDVMG
gi|66932947|ref|NP_000005.2| alpha-2-macroglobulin precursor	ADAMTS4 peptidase	SDVMGRGH
gi|66932947|ref|NP_000005.2| alpha-2-macroglobulin precursor	ADAMTS5 peptidase	SDVMGRGH
gi|4502403|ref|NP_001702.1| biglycan preproprotein	procollagen C-peptidase	FMMNDEEA
gi|4502403|ref|NP_001702.1| biglycan preproprotein	mammalian tolloid-like 1 protein	FMMNDEEA
gi|19743852|ref|NP_598013.1| decorin isoform d precursor	procollagen C-peptidase	FMLEDEAS
gi|19743850|ref|NP_598012.1| decorin isoform c precursor	procollagen C-peptidase	FMLEDEAS
gi|4507605|ref|NP_001243.1| tumor necrosis factor ligand superfamily member 7	ADAM8 peptidase	FAQAQQQL
gi|46370062|ref|NP_996916.1| endothelin 3 isoform 3 preproprotein	endothelin-converting enzyme 1	DIIWINTP
gi|46370062|ref|NP_996916.1| endothelin 3 isoform 3 preproprotein	Kell blood-group protein	DIIWINTP
gi|46370062|ref|NP_996916.1| endothelin 3 isoform 3 preproprotein	endothelin-converting enzyme 2	DIIWINTP
gi|7770077|ref|NP_054703.1| tachykinin 1 isoform gamma precursor	neprilysin	KPQQFFGL
gi|7770077|ref|NP_054703.1| tachykinin 1 isoform gamma precursor	neprilysin	PQQFFGLM

gi|7770077|ref|NP_054703.1| tachykinin 1 isoform gamma precursor	endothelin-converting enzyme 1	KPQQFFGL
gi|7770077|ref|NP_054703.1| tachykinin 1 isoform gamma precursor	endothelin-converting enzyme 1	PQQFFGLM
gi|46370064|ref|NP_996917.1| endothelin 3 isoform 1 preproprotein	endothelin-converting enzyme 1	DIIWINTP
gi|46370064|ref|NP_996917.1| endothelin 3 isoform 1 preproprotein	Kell blood-group protein	DIIWINTP
gi|46370064|ref|NP_996917.1| endothelin 3 isoform 1 preproprotein	endothelin-converting enzyme 2	DIIWINTP
gi|7770075|ref|NP_054702.1| tachykinin 1 isoform alpha precursor	neprilysin	KPQQFFGL
gi|7770075|ref|NP_054702.1| tachykinin 1 isoform alpha precursor	neprilysin	PQQFFGLM
gi|7770075|ref|NP_054702.1| tachykinin 1 isoform alpha precursor	endothelin-converting enzyme 1	KPQQFFGL
gi|7770075|ref|NP_054702.1| tachykinin 1 isoform alpha precursor	endothelin-converting enzyme 1	PQQFFGLM
gi|4557671|ref|NP_000198.1| proinsulin precursor	neprilysin	GSHLVEAL
gi|4557671|ref|NP_000198.1| proinsulin precursor	neprilysin	LVEALYLV
gi|4557671|ref|NP_000198.1| proinsulin precursor	neprilysin	GERGFFYT
gi|4557671|ref|NP_000198.1| proinsulin precursor	neprilysin	ERGFFYTP
gi|4557671|ref|NP_000198.1| proinsulin precursor	neprilysin	RGFFYTPK
gi|39995091|ref|NP_945316.1| parathyroid hormone-like hormone isoform 1 preproprotein	PHEX peptidase	DHLSDTST
gi|39995093|ref|NP_945317.1| parathyroid hormone-like hormone isoform 1 preproprotein	PHEX peptidase	DHLSDTST
gi|23510319|ref|NP_006163.1| natriuretic peptide precursor A	neprilysin	FGGRMDRI
gi|23510319|ref|NP_006163.1| natriuretic peptide precursor A	neprilysin	RMDRIGAQ
gi|23510319|ref|NP_006163.1| natriuretic peptide precursor A	neprilysin	SLRRSSCF
gi|23510319|ref|NP_006163.1| natriuretic peptide precursor A	neprilysin	RSSCFGGR
gi|23510319|ref|NP_006163.1| natriuretic peptide precursor A	neprilysin	DRIGAQSG
gi|23510319|ref|NP_006163.1| natriuretic peptide precursor A	neprilysin	AQSGLGCN
gi|154800437|ref|NP_001946.3| endothelin 1	endothelin-converting enzyme 1	DIIWVNTP
gi|154800437|ref|NP_001946.3| endothelin 1	neprilysin-2	DIIWVNTP

gi|154800437|ref|NP_001946.3| endothelin 1	Kell blood-group protein	DIIWVNTP
gi|154800437|ref|NP_001946.3| endothelin 1	endothelin-converting enzyme 1	DIIWVNTP
gi|154800437|ref|NP_001946.3| endothelin 1	Kell blood-group protein	DIIWVNTP
gi|154800437|ref|NP_001946.3| endothelin 1	endothelin-converting enzyme 1	CHLDIIWV
gi|154800437|ref|NP_001946.3| endothelin 1	endothelin-converting enzyme 1	YGLGSPRS
gi|154800437|ref|NP_001946.3| endothelin 1	endothelin-converting enzyme 1	TPEHVVPY
gi|154800437|ref|NP_001946.3| endothelin 1	endothelin-converting enzyme 1	HLDIIWVN
gi|154800437|ref|NP_001946.3| endothelin 1	endothelin-converting enzyme 1	CVYFCHLD
gi|154800437|ref|NP_001946.3| endothelin 1	endothelin-converting enzyme 1	SCSSLMDK
gi|154800437|ref|NP_001946.3| endothelin 1	endothelin-converting enzyme 1	ECVYFCHL
gi|154800437|ref|NP_001946.3| endothelin 1	endothelin-converting enzyme 2	DIIWVNTP
gi|4502167|ref|NP_000475.1| amyloid beta A4 protein precursor isoform a	neprilysin	QKLVFFAE
gi|4502167|ref|NP_000475.1| amyloid beta A4 protein precursor isoform a	endothelin-converting enzyme 1	HQKLVFFA
gi|4502167|ref|NP_000475.1| amyloid beta A4 protein precursor isoform a	endothelin-converting enzyme 1	HHQKLVFF
gi|4502167|ref|NP_000475.1| amyloid beta A4 protein precursor isoform a	endothelin-converting enzyme 1	KLVFFAED
gi|41406055|ref|NP_958816.1| amyloid beta A4 protein precursor isoform b	neprilysin	QKLVFFAE
gi|41406055|ref|NP_958816.1| amyloid beta A4 protein precursor isoform b	endothelin-converting enzyme 1	HQKLVFFA

gi|41406055|ref|NP_958816.1| amyloid beta A4 protein precursor isoform b	endothelin-converting enzyme 1	HHQKLVFF
gi|41406055|ref|NP_958816.1| amyloid beta A4 protein precursor isoform b	endothelin-converting enzyme 1	KLVFFAED
gi|41406057|ref|NP_958817.1| amyloid beta A4 protein precursor isoform c	neprilysin	QKLVFFAE
gi|41406057|ref|NP_958817.1| amyloid beta A4 protein precursor isoform c	endothelin-converting enzyme 1	HQKLVFFA
gi|41406057|ref|NP_958817.1| amyloid beta A4 protein precursor isoform c	endothelin-converting enzyme 1	HHQKLVFF
gi|41406057|ref|NP_958817.1| amyloid beta A4 protein precursor isoform c	endothelin-converting enzyme 1	KLVFFAED
gi|109148522|ref|NP_001035835.1| insulin- insulin-like growth factor 2	neprilysin	GSHLVEAL
gi|109148522|ref|NP_001035835.1| insulin- insulin-like growth factor 2	neprilysin	LVEALYLV
gi|109148522|ref|NP_001035835.1| insulin- insulin-like growth factor 2	neprilysin	GERGFFYT
gi|109148522|ref|NP_001035835.1| insulin- insulin-like growth factor 2	neprilysin	ERGFFYTP
gi|109148522|ref|NP_001035835.1| insulin- insulin-like growth factor 2	neprilysin	RGFFYTPK
gi|4502605|ref|NP_000720.1| cholecystokinin preproprotein	neprilysin	DYMGWMDF
gi|4501945|ref|NP_001115.1| adrenomedullin	neprilysin	VASEFRKK
gi|4501945|ref|NP_001115.1| adrenomedullin	neprilysin	FRKKWNKW
gi|4501945|ref|NP_001115.1| adrenomedullin	neprilysin	KWNKWALS
gi|4501945|ref|NP_001115.1| adrenomedullin	neprilysin	WNKWALSR
gi|4501945|ref|NP_001115.1| adrenomedullin	neprilysin	NKWALSRG
gi|4507341|ref|NP_003173.1| tachykinin 1 isoform beta precursor	neprilysin	KPQQFFGL
gi|4507341|ref|NP_003173.1| tachykinin 1 isoform beta precursor	neprilysin	PQQFFGLM
gi|4507341|ref|NP_003173.1| tachykinin 1 isoform beta precursor	endothelin-converting enzyme 1	KPQQFFGL
gi|4507341|ref|NP_003173.1| tachykinin 1 isoform beta precursor	endothelin-converting enzyme 1	PQQFFGLM
gi|4557545|ref|NP_000105.1| endothelin 3 isoform 1 preproprotein	endothelin-converting enzyme 1	DIIWINTP
gi|4557545|ref|NP_000105.1| endothelin 3 isoform 1 preproprotein	Kell blood-group protein	DIIWINTP

gi|4557545|ref|NP_000105.1| endothelin 3 isoform 1 preproprotein	endothelin-converting enzyme 2	DIIWINTP
gi|7770079|ref|NP_054704.1| tachykinin 1 isoform delta precursor	neprilysin	KPQQFFGL
gi|7770079|ref|NP_054704.1| tachykinin 1 isoform delta precursor	neprilysin	PQQFFGLM
gi|7770079|ref|NP_054704.1| tachykinin 1 isoform delta precursor	endothelin-converting enzyme 1	KPQQFFGL
gi|7770079|ref|NP_054704.1| tachykinin 1 isoform delta precursor	endothelin-converting enzyme 1	PQQFFGLM
gi|4506269|ref|NP_002811.1| parathyroid hormone-like hormone isoform 2 preproprotein	PHEX peptidase	DHLSDTST
gi|4503463|ref|NP_001947.1| endothelin 2	endothelin-converting enzyme 1	DIIWVNTP
gi|4503463|ref|NP_001947.1| endothelin 2	neprilysin-2	DIIWVNTP
gi|4503463|ref|NP_001947.1| endothelin 2	Kell blood-group protein	DIIWVNTP
gi|4503463|ref|NP_001947.1| endothelin 2	endothelin-converting enzyme 1	DIIWVNTP
gi|4503463|ref|NP_001947.1| endothelin 2	Kell blood-group protein	DIIWVNTP
gi|4503463|ref|NP_001947.1| endothelin 2	endothelin-converting enzyme 1	CHLDIIWV
gi|4503463|ref|NP_001947.1| endothelin 2	endothelin-converting enzyme 1	HLDIIWVN
gi|4503463|ref|NP_001947.1| endothelin 2	endothelin-converting enzyme 1	CVYFCHLD
gi|4503463|ref|NP_001947.1| endothelin 2	endothelin-converting enzyme 1	ECVYFCHL
gi|4503463|ref|NP_001947.1| endothelin 2	endothelin-converting enzyme 2	DIIWVNTP
gi|46370060|ref|NP_996915.1| endothelin 3 isoform 2 preproprotein	endothelin-converting enzyme 1	DIIWINTP
gi|46370060|ref|NP_996915.1| endothelin 3 isoform 2 preproprotein	Kell blood-group protein	DIIWINTP
gi|46370060|ref|NP_996915.1| endothelin 3 isoform 2 preproprotein	endothelin-converting enzyme 2	DIIWINTP

gi|39995089|ref|NP_945315.1| parathyroid hormone-like hormone isoform 2 preproprotein	PHEX peptidase	DHLSDTST
gi|5453816|ref|NP_006174.1| neurotensin/neuromedin N preproprotein	nardilysin	YILKRDSY
gi|4557671|ref|NP_000198.1| proinsulin precursor	insulysin	HLVEALYL
gi|21735621|ref|NP_005909.2| mitochondrial malate dehydrogenase precursor	mitochondrial processing peptidase beta-subunit	LRRSFSTS
gi|21735621|ref|NP_005909.2| mitochondrial malate dehydrogenase precursor	mitochondrial processing peptidase beta-subunit	LRRSFSTS
gi|23510319|ref|NP_006163.1| natriuretic peptide precursor A	insulysin	GRMDRIGA
gi|23510319|ref|NP_006163.1| natriuretic peptide precursor A	insulysin	RSSCFGGR
gi|4507897|ref|NP_003372.1| vasoactive intestinal peptide isoform 1 preproprotein	insulysin	YTRLRKQM
gi|31742516|ref|NP_852090.1| frataxin isoform 2 preproprotein	mitochondrial processing peptidase beta-subunit	PRRASSNQ
gi|31742516|ref|NP_852090.1| frataxin isoform 2 preproprotein	mitochondrial processing peptidase beta-subunit	GRRGLRTD
gi|37588853|ref|NP_919416.1| vasoactive intestinal peptide isoform 2 preproprotein	insulysin	YTRLRKQM
gi|38788445|ref|NP_000522.3| ornithine carbamoyltransferase precursor	mitochondrial processing peptidase beta-subunit	MVRNFRCG
gi|80861463|ref|NP_001030333.1| proopiomelanocortin preproprotein	insulysin	LVTLFKNA
gi|80861463|ref|NP_001030333.1| proopiomelanocortin preproprotein	insulysin	VTLFKNAI
gi|4507243|ref|NP_001039.1| somatostatin preproprotein	nardilysin	PRERKAGC
gi|4502167|ref|NP_000475.1| amyloid beta A4 protein precursor isoform a	insulysin	VHHQKLVF
gi|4502167|ref|NP_000475.1| amyloid beta A4 protein precursor isoform a	insulysin	YEVHHQKL
gi|4502167|ref|NP_000475.1| amyloid beta A4 protein precursor isoform a	insulysin	EVHHQKLV
gi|4502167|ref|NP_000475.1| amyloid beta A4 protein precursor isoform a	insulysin	HQKLVFFA
gi|4502167|ref|NP_000475.1| amyloid beta A4 protein precursor isoform a	insulysin	HHQKLVFF

gi|4502167|ref|NP_000475.1| amyloid beta A4 protein precursor isoform a	insulysin	GSNKGAII
gi|4502167|ref|NP_000475.1| amyloid beta A4 protein precursor isoform a	insulysin	DAEFRHDS
gi|4502167|ref|NP_000475.1| amyloid beta A4 protein precursor isoform a	insulysin	KLVFFAED
gi|4502167|ref|NP_000475.1| amyloid beta A4 protein precursor isoform a	insulysin	QKLVFFAE
gi|4502167|ref|NP_000475.1| amyloid beta A4 protein precursor isoform a	insulysin	EVHHQKLV
gi|4505949|ref|NP_000930.1| proopiomelanocortin preproprotein	insulysin	LVTLFKNA
gi|4505949|ref|NP_000930.1| proopiomelanocortin preproprotein	insulysin	VTLFKNAI
gi|41406055|ref|NP_958816.1| amyloid beta A4 protein precursor isoform b	insulysin	VHHQKLVF
gi|41406055|ref|NP_958816.1| amyloid beta A4 protein precursor isoform b	insulysin	YEVHHQKL
gi|41406055|ref|NP_958816.1| amyloid beta A4 protein precursor isoform b	insulysin	EVHHQKLV
gi|41406055|ref|NP_958816.1| amyloid beta A4 protein precursor isoform b	insulysin	HQKLVFFA
gi|41406055|ref|NP_958816.1| amyloid beta A4 protein precursor isoform b	insulysin	HHQKLVFF
gi|41406055|ref|NP_958816.1| amyloid beta A4 protein precursor isoform b	insulysin	GSNKGAII
gi|41406055|ref|NP_958816.1| amyloid beta A4 protein precursor isoform b	insulysin	DAEFRHDS
gi|41406055|ref|NP_958816.1| amyloid beta A4 protein precursor isoform b	insulysin	KLVFFAED
gi|41406055|ref|NP_958816.1| amyloid beta A4 protein precursor isoform b	insulysin	QKLVFFAE
gi|41406055|ref|NP_958816.1| amyloid beta A4 protein precursor isoform b	insulysin	EVHHQKLV
gi|41406057|ref|NP_958817.1| amyloid beta A4 protein precursor isoform c	insulysin	VHHQKLVF
gi|41406057|ref|NP_958817.1| amyloid beta A4 protein precursor isoform c	insulysin	YEVHHQKL
gi|41406057|ref|NP_958817.1| amyloid beta A4 protein precursor isoform c	insulysin	EVHHQKLV
gi|41406057|ref|NP_958817.1| amyloid beta A4 protein precursor isoform c	insulysin	HQKLVFFA
gi|41406057|ref|NP_958817.1| amyloid beta A4 protein precursor isoform c	insulysin	HHQKLVFF
gi|41406057|ref|NP_958817.1| amyloid beta A4 protein precursor isoform c	insulysin	GSNKGAII
gi|41406057|ref|NP_958817.1| amyloid beta A4 protein precursor isoform c	insulysin	DAEFRHDS

gi|41406057|ref|NP_958817.1| amyloid beta A4 protein precursor isoform c	insulysin	KLVFFAED
gi|41406057|ref|NP_958817.1| amyloid beta A4 protein precursor isoform c	insulysin	QKLVFFAE
gi|41406057|ref|NP_958817.1| amyloid beta A4 protein precursor isoform c	insulysin	EVHHQKLV
gi|13270473|ref|NP_077722.1| beta-neoendorphin-dynorphin preproprotein	insulysin	GFLRRIRP
gi|13270473|ref|NP_077722.1| beta-neoendorphin-dynorphin preproprotein	eupitrilysin	KVVTRSQE
gi|109148522|ref|NP_001035835.1| insulin- insulin-like growth factor 2	insulysin	HLVEALYL
gi|31077081|ref|NP_000135.2| frataxin isoform 1 preproprotein	mitochondrial processing peptidase beta-subunit	PRRASSNQ
gi|31077081|ref|NP_000135.2| frataxin isoform 1 preproprotein	mitochondrial processing peptidase beta-subunit	GRRGLRTD
gi|68509940|ref|NP_001020272.1| Golli-mbp isoform 1	angiotensin-converting enzyme peptidase unit 2	VVHFFKNI
gi|68509928|ref|NP_001020261.1| myelin basic protein isoform 3	angiotensin-converting enzyme peptidase unit 2	VVHFFKNI
gi|4505123|ref|NP_002376.1| myelin basic protein isoform 2	angiotensin-converting enzyme peptidase unit 2	VVHFFKNI
gi|68509930|ref|NP_001020252.1| myelin basic protein isoform 1	angiotensin-converting enzyme peptidase unit 2	VVHFFKNI
gi|68509932|ref|NP_001020263.1| myelin basic protein isoform 4	angiotensin-converting enzyme peptidase unit 2	VVHFFKNI
gi|5453816|ref|NP_006174.1| neurotensin/neuromedin N preproprotein	thimet oligopeptidase	NKPRRPYI
gi|156231037|ref|NP_001095886.1| kininogen 1 isoform 1	thimet oligopeptidase	PPGFSPFR
gi|156231037|ref|NP_001095886.1| kininogen 1 isoform 1	mitochondrial intermediate peptidase	PPGFSPFR
gi|4502167|ref|NP_000475.1| amyloid beta A4 protein precursor isoform a	thimet oligopeptidase	EVKMDAEF
gi|41406055|ref|NP_958816.1| amyloid beta A4 protein precursor isoform b	thimet oligopeptidase	EVKMDAEF
gi|41406057|ref|NP_958817.1| amyloid beta A4 protein precursor isoform c	thimet oligopeptidase	EVKMDAEF

gi|4504893|ref|NP_000884.1| kininogen 1 isoform 2	thimet oligopeptidase	PPGFSPFR
gi|4504893|ref|NP_000884.1| kininogen 1 isoform 2	mitochondrial intermediate peptidase	PPGFSPFR
gi|38045915|ref|NP_002572.2| pregnancy-associated plasma protein A preproprotein	pappalysin-1	YERFNFDG
gi|62243290|ref|NP_001543.2| insulin-like growth factor binding protein 4 precursor	pappalysin-1	GGKMKVNG
gi|10834982|ref|NP_000590.1| insulin-like growth factor binding protein 5	pappalysin-1	TQSKFVGG
gi|10834982|ref|NP_000590.1| insulin-like growth factor binding protein 5	pappalysin-1	LTQSKFVG
gi|10834982|ref|NP_000590.1| insulin-like growth factor binding protein 5	pappalysin-2	LTQSKFVG
gi|27477113|ref|NP_004590.2| sterol regulatory element-binding transcription factor 2	S2P peptidase	RILLCVLT
gi|27477113|ref|NP_004590.2| sterol regulatory element-binding transcription factor 2	S2P peptidase	LTFLCLSF

Serine Proteases

gi|126273569|ref|NP_001863.2| plasma carboxypeptidase B2 isoform a preproprotein	thrombin	VSPRASAS
gi|109148542|ref|NP_001596.2| alanyl-tRNA synthetase	granzyme B	VAPDRLRF
gi|4504875|ref|NP_002248.1| kallikrein 1 preproprotein	kallikrein-related peptidase 2	IQSRIVGG
gi|29568103|ref|NP_003080.2| U1 small nuclear ribonucleoprotein 70 kDa	granzyme B	LGNDSRDM
gi|50363217|ref|NP_000286.3| serine proteinase inhibitor clade A member 1	mesotrypsin	AAQKTDTS
gi|41872583|ref|NP_004841.2| Rho-associated coiled-coil containing protein kinase 2	granzyme B	IGLDSSSI
gi|4502027|ref|NP_000468.1| albumin preproprotein	chymase	RETYGEMA
gi|94721239|ref|NP_038203.2| isoleucyl-tRNA synthetase	granzyme B	VTPDQSMV
gi|14550407|ref|NP_000054.2| complement component 2 precursor	complement component activated C1s	SLGRKIQI
gi|14550407|ref|NP_000054.2| complement component 2 precursor	mannan-binding lectin-associated serine peptidase	SLGRKIQI
gi|14550407|ref|NP_000054.2| complement component 2 precursor	mannan-binding lectin-associated serine peptidase	SLGRKIQI
gi|14550407|ref|NP_000054.2| complement component 2 precursor	mannan-binding lectin-associated serine peptidase	SLGRKIQI
gi|78191798|ref|NP_000883.2| plasma kallikrein B1 precursor	plasma kallikrein	TFHKAEYR

gi|78191798|ref|NP_000883.2| plasma kallikrein B1 precursor	coagulation factor XIIa	TSTRIVGG
gi|4506115|ref|NP_000303.1| protein C (inactivator of coagulation factors Va and VIIIa)	thrombin	VDPRLIDG
gi|46409270|ref|NP_997195.1| tubulin alpha 3e	HtrA2 peptidase	VPRAVFVD
gi|46409270|ref|NP_997195.1| tubulin alpha 3e	HtrA2 peptidase	VFVDLEPT
gi|46409270|ref|NP_997195.1| tubulin alpha 3e	HtrA2 peptidase	HSDCAFMV
gi|46409270|ref|NP_997195.1| tubulin alpha 3e	HtrA2 peptidase	VAEITNAC
gi|46409270|ref|NP_997195.1| tubulin alpha 3e	HtrA2 peptidase	PEQLITGK
gi|46409270|ref|NP_997195.1| tubulin alpha 3e	HtrA2 peptidase	GEGMEEGE
gi|46409270|ref|NP_997195.1| tubulin alpha 3e	HtrA2 peptidase	VDLTEFQT
gi|46409270|ref|NP_997195.1| tubulin alpha 3e	HtrA2 peptidase	FSETGAGK
gi|46409270|ref|NP_997195.1| tubulin alpha 3e	HtrA2 peptidase	FVHWYVGE
gi|46409270|ref|NP_997195.1| tubulin alpha 3e	HtrA2 peptidase	AFMVDNEA
gi|46409270|ref|NP_997195.1| tubulin alpha 3e	HtrA2 peptidase	PKDVNAAI
gi|46409270|ref|NP_997195.1| tubulin alpha 3e	HtrA2 peptidase	GFKVGINY
gi|46409270|ref|NP_997195.1| tubulin alpha 3e	HtrA2 peptidase	PRAVFVDL
gi|46409270|ref|NP_997195.1| tubulin alpha 3e	HtrA2 peptidase	AVFVDLEP
gi|4505209|ref|NP_002418.1| matrix metalloproteinase 13 preproprotein	plasmin	KKPRCGVP
gi|4505209|ref|NP_002418.1| matrix metalloproteinase 13 preproprotein	plasmin	DVGEYNVF
gi|4505209|ref|NP_002418.1| matrix metalloproteinase 13 preproprotein	plasmin	GILKENAA
gi|4501881|ref|NP_001091.1| actin alpha 1 skeletal muscle	HtrA2 peptidase	PTLLTEAP
gi|4501881|ref|NP_001091.1| actin alpha 1 skeletal muscle	HtrA2 peptidase	TLLTEAPL
gi|4501881|ref|NP_001091.1| actin alpha 1 skeletal muscle	HtrA2 peptidase	SGLVKAGF
gi|4501881|ref|NP_001091.1| actin alpha 1 skeletal muscle	HtrA2 peptidase	LCYVALDF
gi|4501881|ref|NP_001091.1| actin alpha 1 skeletal muscle	elastase-2	HQGVMVGM
gi|113430845|ref|XP_001129414.1| PREDICTED: similar to DNA dependent protein kinase catalytic subunit	granzyme B	VGPDFGKK
gi|113430845|ref|XP_001129414.1| PREDICTED: similar to DNA dependent protein kinase catalytic subunit	granzyme B	DEVDNKVK
gi|42558279|ref|NP_817124.1| tubulin beta 8	HtrA2 peptidase	WEVISDEH
gi|42558279|ref|NP_817124.1| tubulin beta 8	HtrA2 peptidase	GFQLTHSL
gi|42558279|ref|NP_817124.1| tubulin beta 8	HtrA2 peptidase	THSLGGGT
gi|42558279|ref|NP_817124.1| tubulin beta 8	HtrA2 peptidase	TTCLRFPG
gi|42558279|ref|NP_817124.1| tubulin beta 8	HtrA2 peptidase	LRKLAVNM
gi|42558279|ref|NP_817124.1| tubulin beta 8	HtrA2 peptidase	FPRLHFFM
gi|42558279|ref|NP_817124.1| tubulin beta 8	HtrA2 peptidase	GHYTEGAE
gi|42558279|ref|NP_817124.1| tubulin beta 8	HtrA2 peptidase	GNNWAKGH
gi|42558279|ref|NP_817124.1| tubulin beta 8	HtrA2 peptidase	NHLVSATM
gi|42558279|ref|NP_817124.1| tubulin beta 8	HtrA2 peptidase	THSLGGGT
gi|42558279|ref|NP_817124.1| tubulin beta 8	HtrA2 peptidase	FQLTHSLG

gi|18375501|ref|NP_001632.2| APEX nuclease	granzyme A	TAAKKNDK
gi|74272287|ref|NP_004985.2| matrix metalloproteinase 9 preproprotein	chymase	FQTFEGDL
gi|74272287|ref|NP_004985.2| matrix metalloproteinase 9 preproprotein	trypsin-2	DLGRFQTF
gi|45359865|ref|NP_004762.2| matrix metalloproteinase 20 preproprotein	plasmin	KKPRCGVP
gi|189163532|ref|NP_001121174.1| serine proteinase inhibitor clade A member 1	mesotrypsin	AAQKTDTS
gi|50363237|ref|NP_001002231.1| kallikrein 2 prostatic isoform 2	kallikrein-related peptidase 2	IQSRIVGG
gi|4506155|ref|NP_002765.1| kallikrein-related peptidase 6 isoform A preproprotein	kallikrein-related peptidase 6	LRQRESSQ
gi|4557671|ref|NP_000198.1| proinsulin precursor	chymotrypsin C	SLYQLENY
gi|4557671|ref|NP_000198.1| proinsulin precursor	elastase-2	SHLVEALY
gi|4557671|ref|NP_000198.1| proinsulin precursor	elastase-2	LVEALYLV
gi|4557671|ref|NP_000198.1| proinsulin precursor	cathepsin G	GSHLVEAL
gi|4557671|ref|NP_000198.1| proinsulin precursor	elastase-1	SHLVEALY
gi|4557671|ref|NP_000198.1| proinsulin precursor	elastase-1	LVEALYLV
gi|4557671|ref|NP_000198.1| proinsulin precursor	elastase-1	GERGFFYT
gi|4557671|ref|NP_000198.1| proinsulin precursor	chymotrypsin C	ERGFFYTP
gi|4557671|ref|NP_000198.1| proinsulin precursor	chymotrypsin C	RGFFYTPK
gi|4557671|ref|NP_000198.1| proinsulin precursor	kallikrein-related peptidase 7	RGFFYTPK
gi|11225260|ref|NP_003277.1| DNA topoisomerase I	granzyme B	IEADFRLN
gi|4506879|ref|NP_002997.1| selectin P ligand	cathepsin G	EYEYLDYD
gi|119392081|ref|NP_000195.2| complement factor I preproprotein	plasmin	RRKRIVGG
gi|119392081|ref|NP_000195.2| complement factor I preproprotein	plasmin	ATNRRSFP
gi|119392081|ref|NP_000195.2| complement factor I preproprotein	plasmin	RRFKLSDL
gi|119392081|ref|NP_000195.2| complement factor I preproprotein	plasmin	LCCKACQG
gi|119392081|ref|NP_000195.2| complement factor I preproprotein	plasmin	RRIKSLLP
gi|119392081|ref|NP_000195.2| complement factor I preproprotein	plasmin	VGGKRAQL
gi|119392081|ref|NP_000195.2| complement factor I preproprotein	plasmin	CLAKKYTH
gi|119392081|ref|NP_000195.2| complement factor I preproprotein	plasmin	LAKKYTHL
gi|119392081|ref|NP_000195.2| complement factor I preproprotein	plasmin	PDLKRIVI
gi|119392081|ref|NP_000195.2| complement factor I preproprotein	plasmin	CQQKSLEC
gi|53829379|ref|NP_001005376.1| plasminogen activator urokinase receptor isoform 2 precursor	elastase-2	GRAVTYSR

gi|53829379|ref|NP_001005376.1| plasminogen activator urokinase receptor isoform 2 precursor	cathepsin G	AVTYSRSR
gi|53829379|ref|NP_001005376.1| plasminogen activator urokinase receptor isoform 2 precursor	cathepsin G	RSRYLECI
gi|53829379|ref|NP_001005376.1| plasminogen activator urokinase receptor isoform 2 precursor	u-plasminogen activator	NSGRAVTY
gi|53829379|ref|NP_001005376.1| plasminogen activator urokinase receptor isoform 2 precursor	u-plasminogen activator	TYSRSRYL
gi|169217303|ref|XP_001732908.1| PREDICTED: hypothetical LOC389901	granzyme B	ISSDRDLL
gi|68509940|ref|NP_001020272.1| Golli-mbp isoform 1	cathepsin G	VHFFKNIV
gi|68509940|ref|NP_001020272.1| Golli-mbp isoform 1	cathepsin G	LSRFSWGA
gi|14210536|ref|NP_115914.1| tubulin beta 6	HtrA2 peptidase	WEVISDEH
gi|14210536|ref|NP_115914.1| tubulin beta 6	HtrA2 peptidase	GFQLTHSL
gi|14210536|ref|NP_115914.1| tubulin beta 6	HtrA2 peptidase	THSLGGGT
gi|14210536|ref|NP_115914.1| tubulin beta 6	HtrA2 peptidase	TLKLTTPT
gi|14210536|ref|NP_115914.1| tubulin beta 6	HtrA2 peptidase	LRKLAVNM
gi|14210536|ref|NP_115914.1| tubulin beta 6	HtrA2 peptidase	FPRLHFFM
gi|14210536|ref|NP_115914.1| tubulin beta 6	HtrA2 peptidase	GHYTEGAE
gi|14210536|ref|NP_115914.1| tubulin beta 6	HtrA2 peptidase	TTPTYGDL
gi|14210536|ref|NP_115914.1| tubulin beta 6	HtrA2 peptidase	GNNWAKGH
gi|14210536|ref|NP_115914.1| tubulin beta 6	HtrA2 peptidase	NHLVSATM
gi|14210536|ref|NP_115914.1| tubulin beta 6	HtrA2 peptidase	THSLGGGT
gi|14210536|ref|NP_115914.1| tubulin beta 6	HtrA2 peptidase	FQLTHSLG
gi|14210536|ref|NP_115914.1| tubulin beta 6	HtrA2 peptidase	GLKMASTF
gi|61744424|ref|NP_001012982.1| kallikrein-related peptidase 6 isoform A preproprotein	kallikrein-related peptidase 6	LRQRESSQ
gi|145275213|ref|NP_000496.2| coagulation factor XII precursor	coagulation factor XIIa	SMTRVVGG
gi|145275213|ref|NP_000496.2| coagulation factor XII precursor	plasma kallikrein	SLTRNGPL
gi|145275213|ref|NP_000496.2| coagulation factor XII precursor	plasma kallikrein	SMTRVVGG
gi|145275213|ref|NP_000496.2| coagulation factor XII precursor	plasma kallikrein	CGQRLRKS
gi|4507065|ref|NP_003055.1| secretory leukocyte peptidase inhibitor precursor	kallikrein-related peptidase 3	QCLRYKKP
gi|4507065|ref|NP_003055.1| secretory leukocyte peptidase inhibitor precursor	kallikrein-related peptidase 3	QCLMLNPP
gi|126032350|ref|NP_001075109.1| protein kinase DNA-activated catalytic polypeptide isoform 2	granzyme B	VGPDFGKK
gi|126032350|ref|NP_001075109.1| protein kinase DNA-activated catalytic polypeptide isoform 2	granzyme B	DEVDNKVK
gi|51599156|ref|NP_001264.2| chromodomain helicase DNA binding protein 4	granzyme B	VDPDYWEK
gi|4557707|ref|NP_000416.1| L1 cell adhesion molecule isoform 1 precursor	plasmin	SQRKHSKR
gi|4557707|ref|NP_000416.1| L1 cell adhesion molecule isoform 1 precursor	plasmin	KHSKRHIH

gi|116292750|ref|NP_005568.2| lipoprotein Lp(a) precursor	elastase-2	PNVILAPS
gi|116292750|ref|NP_005568.2| lipoprotein Lp(a) precursor	elastase-2	PTVIQVPS
gi|116292750|ref|NP_005568.2| lipoprotein Lp(a) precursor	elastase-2	MESTLLTT
gi|116292750|ref|NP_005568.2| lipoprotein Lp(a) precursor	plasmin	GNGKGYRG
gi|14790119|ref|NP_004337.2| caspase 3 preproprotein	granzyme B	IETDSGVD
gi|5031829|ref|NP_005542.1| kallikrein 2 prostatic isoform 1	kallikrein-related peptidase 2	IQSRIVGG
gi|73858568|ref|NP_000053.2| complement component 1 inhibitor precursor	chymase	KMLFVEPI
gi|12408647|ref|NP_071801.1| X-ray repair cross complementing protein 4 isoform 2	granzyme B	VSKDDSII
gi|4503649|ref|NP_000124.1| coagulation factor IX preproprotein	coagulation factor XIa	KLTRAETV
gi|4503649|ref|NP_000124.1| coagulation factor IX preproprotein	coagulation factor XIa	DFTRVVGG
gi|4503649|ref|NP_000124.1| coagulation factor IX preproprotein	coagulation factor VIIa	KLTRAETV
gi|4503649|ref|NP_000124.1| coagulation factor IX preproprotein	coagulation factor VIIa	DFTRVVGG
gi|4503649|ref|NP_000124.1| coagulation factor IX preproprotein	coagulation factor XIa	KLTRAETV
gi|4503649|ref|NP_000124.1| coagulation factor IX preproprotein	coagulation factor XIa	DFTRVVGG
gi|189163536|ref|NP_001121176.1| serine proteinase inhibitor clade A member 1	mesotrypsin	AAQKTDTS
gi|4507945|ref|NP_003392.1| X-ray repair cross complementing protein 4 isoform 1	granzyme B	VSKDDSII
gi|12408649|ref|NP_072044.1| X-ray repair cross complementing protein 4 isoform 1	granzyme B	VSKDDSII
gi|14589961|ref|NP_116739.1| chemokine (C-C motif) ligand 14 isoform 1 precursor	u-plasminogen activator	SSSRGPYH
gi|73858570|ref|NP_001027466.1| complement component 1 inhibitor precursor	chymase	KMLFVEPI
gi|14790115|ref|NP_116786.1| caspase 3 preproprotein	granzyme B	IETDSGVD
gi|157739864|ref|NP_001099032.1| ubiquitination factor E4B isoform 1	granzyme B	VDVDSGIE
gi|196162702|ref|NP_001124494.1| X-ray repair cross complementing protein 4 isoform 3	granzyme B	VSKDDSII
gi|47132547|ref|NP_473375.2| fibronectin 1 isoform 7 preproprotein	kallikrein-related peptidase 2	TDVRAAVY
gi|47132547|ref|NP_473375.2| fibronectin 1 isoform 7 preproprotein	kallikrein-related peptidase 3	AAVYQPQP
gi|4505865|ref|NP_002650.1| plasminogen activator urokinase receptor isoform 1 precursor	elastase-2	GRAVTYSR

gi|4505865|ref|NP_002650.1| plasminogen activator urokinase receptor isoform 1 precursor	cathepsin G	AVTYSRSR
gi|4505865|ref|NP_002650.1| plasminogen activator urokinase receptor isoform 1 precursor	cathepsin G	RSRYLECI
gi|4505865|ref|NP_002650.1| plasminogen activator urokinase receptor isoform 1 precursor	u-plasminogen activator	NSGRAVTY
gi|4505865|ref|NP_002650.1| plasminogen activator urokinase receptor isoform 1 precursor	u-plasminogen activator	TYSRSRYL
gi|47132549|ref|NP_997639.1| fibronectin 1 isoform 6 preproprotein	kallikrein-related peptidase 2	TDVRAAVY
gi|47132549|ref|NP_997639.1| fibronectin 1 isoform 6 preproprotein	kallikrein-related peptidase 3	VSVYTVKD
gi|47132549|ref|NP_997639.1| fibronectin 1 isoform 6 preproprotein	kallikrein-related peptidase 3	AAVYQPQP
gi|47132549|ref|NP_997639.1| fibronectin 1 isoform 6 preproprotein	kallikrein-related peptidase 3	ITIYAVEE
gi|47132553|ref|NP_997641.1| fibronectin 1 isoform 5 preproprotein	kallikrein-related peptidase 2	GFRRTTPP
gi|47132553|ref|NP_997641.1| fibronectin 1 isoform 5 preproprotein	kallikrein-related peptidase 2	TDVRAAVY
gi|47132553|ref|NP_997641.1| fibronectin 1 isoform 5 preproprotein	kallikrein-related peptidase 3	VSVYTVKD
gi|47132553|ref|NP_997641.1| fibronectin 1 isoform 5 preproprotein	kallikrein-related peptidase 3	AAVYQPQP
gi|47132553|ref|NP_997641.1| fibronectin 1 isoform 5 preproprotein	kallikrein-related peptidase 3	ITIYAVEE
gi|158420731|ref|NP_001005271.2| chromodomain helicase DNA binding protein 3 isoform 3	granzyme B	VDPDYWEK
gi|7019477|ref|NP_037379.1| HtrA serine peptidase 2 isoform 1 preproprotein	HtrA2 peptidase	VRLLSGDT
gi|7019477|ref|NP_037379.1| HtrA serine peptidase 2 isoform 1 preproprotein	HtrA2 peptidase	PFALQNTI
gi|7019477|ref|NP_037379.1| HtrA serine peptidase 2 isoform 1 preproprotein	HtrA2 peptidase	LQNTITSG
gi|47132555|ref|NP_997643.1| fibronectin 1 isoform 4 preproprotein	kallikrein-related peptidase 2	GFRRTTPP
gi|47132555|ref|NP_997643.1| fibronectin 1 isoform 4 preproprotein	kallikrein-related peptidase 2	TDVRAAVY
gi|47132555|ref|NP_997643.1| fibronectin 1 isoform 4 preproprotein	kallikrein-related peptidase 3	VSVYTVKD
gi|47132555|ref|NP_997643.1| fibronectin 1 isoform 4 preproprotein	kallikrein-related peptidase 3	AAVYQPQP
gi|47132555|ref|NP_997643.1| fibronectin 1 isoform 4 preproprotein	kallikrein-related peptidase 3	ITIYAVEE
gi|38327034|ref|NP_006039.2| ubiquitination factor E4B isoform 2	granzyme B	VDVDSGIE
gi|4501889|ref|NP_001606.1| actin gamma 2 propeptide	HtrA2 peptidase	PTLLTEAP

gi|4501889|ref|NP_001606.1| actin gamma 2 propeptide	HtrA2 peptidase	TLLTEAPL
gi|4501889|ref|NP_001606.1| actin gamma 2 propeptide	HtrA2 peptidase	LCYVALDF
gi|4501889|ref|NP_001606.1| actin gamma 2 propeptide	elastase-2	HQGVMVGM
gi|53829381|ref|NP_001005377.1| plasminogen activator urokinase receptor isoform 3 precursor	elastase-2	GRAVTYSR
gi|53829381|ref|NP_001005377.1| plasminogen activator urokinase receptor isoform 3 precursor	cathepsin G	AVTYSRSR
gi|53829381|ref|NP_001005377.1| plasminogen activator urokinase receptor isoform 3 precursor	cathepsin G	RSRYLECI
gi|53829381|ref|NP_001005377.1| plasminogen activator urokinase receptor isoform 3 precursor	u-plasminogen activator	NSGRAVTY
gi|53829381|ref|NP_001005377.1| plasminogen activator urokinase receptor isoform 3 precursor	u-plasminogen activator	TYSRSRYL
gi|14389309|ref|NP_116093.1| tubulin alpha 6	HtrA2 peptidase	VPRAVFVD
gi|14389309|ref|NP_116093.1| tubulin alpha 6	HtrA2 peptidase	VFVDLEPT
gi|14389309|ref|NP_116093.1| tubulin alpha 6	HtrA2 peptidase	HSDCAFMV
gi|14389309|ref|NP_116093.1| tubulin alpha 6	HtrA2 peptidase	VAEITNAC
gi|14389309|ref|NP_116093.1| tubulin alpha 6	HtrA2 peptidase	PEQLITGK
gi|14389309|ref|NP_116093.1| tubulin alpha 6	HtrA2 peptidase	GEGMEEGE
gi|14389309|ref|NP_116093.1| tubulin alpha 6	HtrA2 peptidase	VDLTEFQT
gi|14389309|ref|NP_116093.1| tubulin alpha 6	HtrA2 peptidase	FSETGAGK
gi|14389309|ref|NP_116093.1| tubulin alpha 6	HtrA2 peptidase	FVHWYVGE
gi|14389309|ref|NP_116093.1| tubulin alpha 6	HtrA2 peptidase	GFLVFHSF
gi|14389309|ref|NP_116093.1| tubulin alpha 6	HtrA2 peptidase	AFMVDNEA
gi|14389309|ref|NP_116093.1| tubulin alpha 6	HtrA2 peptidase	PKDVNAAI
gi|14389309|ref|NP_116093.1| tubulin alpha 6	HtrA2 peptidase	GFKVGINY
gi|14389309|ref|NP_116093.1| tubulin alpha 6	HtrA2 peptidase	PRAVFVDL
gi|14389309|ref|NP_116093.1| tubulin alpha 6	HtrA2 peptidase	AVFVDLEP
gi|4503475|ref|NP_001949.1| eukaryotic translation elongation factor 1 alpha 2	HtrA2 peptidase	LQDVYKIG
gi|21614538|ref|NP_659540.1| HtrA serine peptidase 2 isoform 2	HtrA2 peptidase	VRLLSGDT
gi|4506883|ref|NP_002998.1| semenogelin I isoform a preproprotein	kallikrein-related peptidase 3	QSTNREQD
gi|4506883|ref|NP_002998.1| semenogelin I isoform a preproprotein	kallikrein-related peptidase 3	VQKDVSQS
gi|4506883|ref|NP_002998.1| semenogelin I isoform a preproprotein	kallikrein-related peptidase 3	VQKDVSQR
gi|4506883|ref|NP_002998.1| semenogelin I isoform a preproprotein	kallikrein-related peptidase 3	GHYQNVVE
gi|4506883|ref|NP_002998.1| semenogelin I isoform a preproprotein	kallikrein-related peptidase 3	SKVQTSLC
gi|4506883|ref|NP_002998.1| semenogelin I isoform a preproprotein	kallikrein-related peptidase 3	IPSQEQEH
gi|4506883|ref|NP_002998.1| semenogelin I isoform a preproprotein	kallikrein-related peptidase 3	QQLLHNKQ

gi|4506883|ref|NP_002998.1| semenogelin I isoform a preproprotein	kallikrein-related peptidase 3	SSQYSNTE
gi|4506883|ref|NP_002998.1| semenogelin I isoform a preproprotein	kallikrein-related peptidase 3	LLVYNKNQ
gi|4506883|ref|NP_002998.1| semenogelin I isoform a preproprotein	kallikrein-related peptidase 3	SSIYSQTE
gi|4506883|ref|NP_002998.1| semenogelin I isoform a preproprotein	kallikrein-related peptidase 3	RSIYSQTE
gi|4506883|ref|NP_002998.1| semenogelin I isoform a preproprotein	kallikrein-related peptidase 3	GQHYSGQK
gi|4506883|ref|NP_002998.1| semenogelin I isoform a preproprotein	kallikrein-related peptidase 3	SIQYTYHV
gi|4506883|ref|NP_002998.1| semenogelin I isoform a preproprotein	kallikrein-related peptidase 3	SQQYDLNA
gi|4506883|ref|NP_002998.1| semenogelin I isoform a preproprotein	kallikrein-related peptidase 2	KAHRGTQN
gi|4506883|ref|NP_002998.1| semenogelin I isoform a preproprotein	kallikrein-related peptidase 2	EERRLHYG
gi|4506883|ref|NP_002998.1| semenogelin I isoform a preproprotein	kallikrein-related peptidase 2	VSQRSIYS
gi|4506883|ref|NP_002998.1| semenogelin I isoform a preproprotein	kallikrein-related peptidase 3	ISYQSSST
gi|4506883|ref|NP_002998.1| semenogelin I isoform a preproprotein	kallikrein-related peptidase 3	ISYQSSST
gi|4506883|ref|NP_002998.1| semenogelin I isoform a preproprotein	kallikrein-related peptidase 3	RLHYGENG
gi|4506883|ref|NP_002998.1| semenogelin I isoform a preproprotein	kallikrein-related peptidase 3	RLHYGENG
gi|4506883|ref|NP_002998.1| semenogelin I isoform a preproprotein	kallikrein-related peptidase 3	ISYQSSST
gi|4506883|ref|NP_002998.1| semenogelin I isoform a preproprotein	kallikrein-related peptidase 3	QSSYVLQT
gi|4506883|ref|NP_002998.1| semenogelin I isoform a preproprotein	kallikrein-related peptidase 3	SQQYDLNA
gi|4506883|ref|NP_002998.1| semenogelin I isoform a preproprotein	kallikrein-related peptidase 3	ISYQSSST
gi|47132557|ref|NP_997647.1| fibronectin 1 isoform 1 preproprotein	kallikrein-related peptidase 2	GFRRTTPP
gi|47132557|ref|NP_997647.1| fibronectin 1 isoform 1 preproprotein	kallikrein-related peptidase 2	TDVRAAVY
gi|47132557|ref|NP_997647.1| fibronectin 1 isoform 1 preproprotein	kallikrein-related peptidase 3	VSVYTVKD
gi|47132557|ref|NP_997647.1| fibronectin 1 isoform 1 preproprotein	kallikrein-related peptidase 3	AAVYQPQP
gi|47132557|ref|NP_997647.1| fibronectin 1 isoform 1 preproprotein	kallikrein-related peptidase 3	ITIYAVEE
gi|16933542|ref|NP_002017.1| fibronectin 1 isoform 3 preproprotein	kallikrein-related peptidase 2	GFRRTTPP

gi|16933542|ref|NP_002017.1| fibronectin 1 isoform 3 preproprotein	kallikrein-related peptidase 2	TDVRAAVY
gi|16933542|ref|NP_002017.1| fibronectin 1 isoform 3 preproprotein	kallikrein-related peptidase 3	VSVYTVKD
gi|16933542|ref|NP_002017.1| fibronectin 1 isoform 3 preproprotein	kallikrein-related peptidase 3	AAVYQPQP
gi|16933542|ref|NP_002017.1| fibronectin 1 isoform 3 preproprotein	kallikrein-related peptidase 3	ITIYAVEE
gi|38016947|ref|NP_001726.2| complement component 5 preproprotein	complement component C2a	QLGRLHMK
gi|38016947|ref|NP_001726.2| complement component 5 preproprotein	complement factor B	QLGRLHMK
gi|169217813|ref|XP_001713833.1| PREDICTED: hypothetical protein	enteropeptidase	DDDKIVGG
gi|47132551|ref|NP_997640.1| fibronectin 1 isoform 2 preproprotein	kallikrein-related peptidase 2	GFRRTTPP
gi|47132551|ref|NP_997640.1| fibronectin 1 isoform 2 preproprotein	kallikrein-related peptidase 2	TDVRAAVY
gi|47132551|ref|NP_997640.1| fibronectin 1 isoform 2 preproprotein	kallikrein-related peptidase 3	VSVYTVKD
gi|47132551|ref|NP_997640.1| fibronectin 1 isoform 2 preproprotein	kallikrein-related peptidase 3	AAVYQPQP
gi|47132551|ref|NP_997640.1| fibronectin 1 isoform 2 preproprotein	kallikrein-related peptidase 3	ITIYAVEE
gi|67190748|ref|NP_009224.2| complement component 4A preproprotein	complement component activated C1s	GLQRALEI
gi|67190748|ref|NP_009224.2| complement component 4A preproprotein	mannan-binding lectin-associated serine peptidase	GLQRALEI
gi|67190748|ref|NP_009224.2| complement component 4A preproprotein	mannan-binding lectin-associated serine peptidase	GLQRALEI
gi|67190748|ref|NP_009224.2| complement component 4A preproprotein	mannan-binding lectin-associated serine peptidase	GLQRALEI
gi|67190748|ref|NP_009224.2| complement component 4A preproprotein	complement factor I	STGRNGFK
gi|67190748|ref|NP_009224.2| complement component 4A preproprotein	complement factor I	HRGRTLEI
gi|4505917|ref|NP_002676.1| exosome component 10 isoform 2	granzyme B	VEQDMFAH
gi|4502907|ref|NP_001827.1| chymase 1 mast cell preproprotein	chymase	PSQFNFVP
gi|5032179|ref|NP_005753.1| tripartite motif-containing 28 protein	HtrA2 peptidase	HQRVKYTK
gi|38049014|ref|NP_937782.1| semenogelin I isoform b preproprotein	kallikrein-related peptidase 3	QSTNREQD

gi|38049014|ref|NP_937782.1| semenogelin I isoform b preproprotein	kallikrein-related peptidase 3	VQKDVSQS
gi|38049014|ref|NP_937782.1| semenogelin I isoform b preproprotein	kallikrein-related peptidase 3	GHYQNVVE
gi|38049014|ref|NP_937782.1| semenogelin I isoform b preproprotein	kallikrein-related peptidase 3	SKVQTSLC
gi|38049014|ref|NP_937782.1| semenogelin I isoform b preproprotein	kallikrein-related peptidase 3	QQLLHNKQ
gi|38049014|ref|NP_937782.1| semenogelin I isoform b preproprotein	kallikrein-related peptidase 3	SSQYSNTE
gi|38049014|ref|NP_937782.1| semenogelin I isoform b preproprotein	kallikrein-related peptidase 3	LLVYNKNQ
gi|38049014|ref|NP_937782.1| semenogelin I isoform b preproprotein	kallikrein-related peptidase 3	SSIYSQTE
gi|38049014|ref|NP_937782.1| semenogelin I isoform b preproprotein	kallikrein-related peptidase 3	GQHYSGQK
gi|38049014|ref|NP_937782.1| semenogelin I isoform b preproprotein	kallikrein-related peptidase 3	SIQYTYHV
gi|38049014|ref|NP_937782.1| semenogelin I isoform b preproprotein	kallikrein-related peptidase 3	SQQYDLNA
gi|38049014|ref|NP_937782.1| semenogelin I isoform b preproprotein	kallikrein-related peptidase 2	KAHRGTQN
gi|38049014|ref|NP_937782.1| semenogelin I isoform b preproprotein	kallikrein-related peptidase 2	EERRLHYG
gi|38049014|ref|NP_937782.1| semenogelin I isoform b preproprotein	kallikrein-related peptidase 3	ISYQSSST
gi|38049014|ref|NP_937782.1| semenogelin I isoform b preproprotein	kallikrein-related peptidase 3	ISYQSSST
gi|38049014|ref|NP_937782.1| semenogelin I isoform b preproprotein	kallikrein-related peptidase 3	RLHYGENG
gi|38049014|ref|NP_937782.1| semenogelin I isoform b preproprotein	kallikrein-related peptidase 3	RLHYGENG
gi|38049014|ref|NP_937782.1| semenogelin I isoform b preproprotein	kallikrein-related peptidase 3	ISYQSSST
gi|38049014|ref|NP_937782.1| semenogelin I isoform b preproprotein	kallikrein-related peptidase 3	QSSYVLQT
gi|38049014|ref|NP_937782.1| semenogelin I isoform b preproprotein	kallikrein-related peptidase 3	SQQYDLNA
gi|38049014|ref|NP_937782.1| semenogelin I isoform b preproprotein	kallikrein-related peptidase 3	ISYQSSST
gi|71834853|ref|NP_001025218.1| prostate specific antigen isoform 3 preproprotein	kallikrein-related peptidase 15	ILSRIVGG
gi|71834853|ref|NP_001025218.1| prostate specific antigen isoform 3 preproprotein	kallikrein-related peptidase 2	ILSRIVGG
gi|156231037|ref|NP_001095886.1| kininogen 1 isoform 1	elastase-2	LGMISLMK
gi|156231037|ref|NP_001095886.1| kininogen 1 isoform 1	elastase-2	SSRIGEIK
gi|156231037|ref|NP_001095886.1| kininogen 1 isoform 1	kallikrein 1	SPFRSSRI
gi|156231037|ref|NP_001095886.1| kininogen 1 isoform 1	kallikrein 1	ISLMKRPP

gi|156231037|ref|NP_001095886.1| kininogen 1 isoform 1	kallikrein-related peptidase 2	SPFRSSRI
gi|156231037|ref|NP_001095886.1| kininogen 1 isoform 1	kallikrein-related peptidase 2	SLMKRPPG
gi|156231037|ref|NP_001095886.1| kininogen 1 isoform 1	plasma kallikrein	SPFRSSRI
gi|156231037|ref|NP_001095886.1| kininogen 1 isoform 1	plasma kallikrein	SLMKRPPG
gi|119395709|ref|NP_000120.2| coagulation factor XIII A1 subunit precursor	thrombin	VVPRGVNL
gi|189163528|ref|NP_001121172.1| serine proteinase inhibitor clade A member 1	mesotrypsin	AAQKTDTS
gi|4557361|ref|NP_001187.1| BH3 interacting domain death agonist isoform 2	granzyme B	IEADSESQ
gi|4505215|ref|NP_002412.1| matrix metalloproteinase 1 preproprotein	plasmin	DVAQFVLT
gi|4505215|ref|NP_002412.1| matrix metalloproteinase 1 preproprotein	chymase	QFVLTEGN
gi|189163534|ref|NP_001121175.1| serine proteinase inhibitor clade A member 1	mesotrypsin	AAQKTDTS
gi|4826898|ref|NP_005013.1| profilin 1	chymase	PSVWAAVP
gi|126090883|ref|NP_001126.2| aggrecan isoform 1 precursor	elastase-2	RGSVILTV
gi|126090883|ref|NP_001126.2| aggrecan isoform 1 precursor	u-plasminogen activator	RGSVILTV
gi|126090883|ref|NP_001126.2| aggrecan isoform 1 precursor	plasmin	GEARGSVI
gi|24432106|ref|NP_066997.3| p30 DBC protein	HtrA2 peptidase	LFQTSHTL
gi|24432106|ref|NP_066997.3| p30 DBC protein	HtrA2 peptidase	LLHVAALG
gi|71834855|ref|NP_001025219.1| prostate specific antigen isoform 4 preproprotein	kallikrein-related peptidase 15	ILSRIVGG
gi|71834855|ref|NP_001025219.1| prostate specific antigen isoform 4 preproprotein	kallikrein-related peptidase 2	ILSRIVGG
gi|4505221|ref|NP_002415.1| matrix metalloproteinase 8 preproprotein	plasmin	KKPRCGVP
gi|169218213|ref|XP_001719515.1| PREDICTED: hypothetical protein partial	cathepsin G	GLARSNLD
gi|169218213|ref|XP_001719515.1| PREDICTED: hypothetical protein partial	cathepsin G	RSNLDEDI
gi|169218213|ref|XP_001719515.1| PREDICTED: hypothetical protein partial	complement component C2a	GLARSNLD
gi|169218213|ref|XP_001719515.1| PREDICTED: hypothetical protein partial	complement factor B	GLARSNLD
gi|88953571|ref|XP_933678.1| PREDICTED: similar to protein expressed in prostate ovary testis and placenta 2 isoform 2	HtrA2 peptidase	LCYVALDF
gi|21361322|ref|NP_006078.2| tubulin beta 4	HtrA2 peptidase	WEVISDEH
gi|21361322|ref|NP_006078.2| tubulin beta 4	HtrA2 peptidase	GFQLTHSL
gi|21361322|ref|NP_006078.2| tubulin beta 4	HtrA2 peptidase	THSLGGGT
gi|21361322|ref|NP_006078.2| tubulin beta 4	HtrA2 peptidase	TLKLTTPT
gi|21361322|ref|NP_006078.2| tubulin beta 4	HtrA2 peptidase	TTCLRFPG

gi|21361322|ref|NP_006078.2| tubulin beta 4	HtrA2 peptidase	LRKLAVNM
gi|21361322|ref|NP_006078.2| tubulin beta 4	HtrA2 peptidase	FPRLHFFM
gi|21361322|ref|NP_006078.2| tubulin beta 4	HtrA2 peptidase	GHYTEGAE
gi|21361322|ref|NP_006078.2| tubulin beta 4	HtrA2 peptidase	TTPTYGDL
gi|21361322|ref|NP_006078.2| tubulin beta 4	HtrA2 peptidase	GNNWAKGH
gi|21361322|ref|NP_006078.2| tubulin beta 4	HtrA2 peptidase	NHLVSATM
gi|21361322|ref|NP_006078.2| tubulin beta 4	HtrA2 peptidase	THSLGGGT
gi|21361322|ref|NP_006078.2| tubulin beta 4	HtrA2 peptidase	FQLTHSLG
gi|25777728|ref|NP_733798.1| aldehyde dehydrogenase 1A2 isoform 3	HtrA2 peptidase	IDKIAFTG
gi|23510319|ref|NP_006163.1| natriuretic peptide precursor A	corin	TAPRSLRR
gi|11342666|ref|NP_004521.1| matrix metalloproteinase 2 isoform a preproprotein	trypsin-2	ETMRKPRC
gi|11342666|ref|NP_004521.1| matrix metalloproteinase 2 isoform a preproprotein	elastase-2	PETAMSTV
gi|11342666|ref|NP_004521.1| matrix metalloproteinase 2 isoform a preproprotein	elastase-2	ATTANYDD
gi|11342666|ref|NP_004521.1| matrix metalloproteinase 2 isoform a preproprotein	elastase-2	LGPVTPEI
gi|37574726|ref|NP_932070.1| BH3 interacting domain death agonist isoform 1	granzyme B	IEADSESQ
gi|113413200|ref|XP_934799.2| PREDICTED: similar to protein expressed in prostate ovary testis and placenta 2	HtrA2 peptidase	LCYVALDF
gi|4506145|ref|NP_002760.1| protease serine 1 preproprotein	enteropeptidase	DDDKIVGG
gi|169208743|ref|XP_001716889.1| PREDICTED: similar to hCG1647990	HtrA2 peptidase	LTQQVFDA
gi|29788785|ref|NP_821133.1| tubulin beta	HtrA2 peptidase	LTQQVFDA
gi|29788785|ref|NP_821133.1| tubulin beta	HtrA2 peptidase	WEVISDEH
gi|29788785|ref|NP_821133.1| tubulin beta	HtrA2 peptidase	LDRISVYY
gi|29788785|ref|NP_821133.1| tubulin beta	HtrA2 peptidase	GFQLTHSL
gi|29788785|ref|NP_821133.1| tubulin beta	HtrA2 peptidase	THSLGGGT
gi|29788785|ref|NP_821133.1| tubulin beta	HtrA2 peptidase	TLKLTTPT
gi|29788785|ref|NP_821133.1| tubulin beta	HtrA2 peptidase	TTCLRFPG
gi|29788785|ref|NP_821133.1| tubulin beta	HtrA2 peptidase	LRKLAVNM
gi|29788785|ref|NP_821133.1| tubulin beta	HtrA2 peptidase	FPRLHFFM
gi|29788785|ref|NP_821133.1| tubulin beta	HtrA2 peptidase	GHYTEGAE
gi|29788785|ref|NP_821133.1| tubulin beta	HtrA2 peptidase	TTPTYGDL
gi|29788785|ref|NP_821133.1| tubulin beta	HtrA2 peptidase	GNNWAKGH
gi|29788785|ref|NP_821133.1| tubulin beta	HtrA2 peptidase	NHLVSATM
gi|29788785|ref|NP_821133.1| tubulin beta	HtrA2 peptidase	AILVDLEP
gi|29788785|ref|NP_821133.1| tubulin beta	HtrA2 peptidase	THSLGGGT
gi|29788785|ref|NP_821133.1| tubulin beta	HtrA2 peptidase	FQLTHSLG
gi|70906435|ref|NP_005132.2| fibrinogen beta chain preproprotein	thrombin	FSARGHRP
gi|67089157|ref|NP_150243.2| promyelocytic leukemia protein isoform 2	elastase-2	PMAVVQSV

gi|67089157|ref|NP_150243.2| promyelocytic leukemia protein isoform 2	elastase-2	PVPVYAFS
gi|126090892|ref|NP_037359.2| aggrecan isoform 2 precursor	elastase-2	RGSVILTV
gi|126090892|ref|NP_037359.2| aggrecan isoform 2 precursor	u-plasminogen activator	RGSVILTV
gi|126090892|ref|NP_037359.2| aggrecan isoform 2 precursor	plasmin	GEARGSVI
gi|189163538|ref|NP_001121177.1| serine proteinase inhibitor clade A member 1	mesotrypsin	AAQKTDTS
gi|50592996|ref|NP_006077.2| tubulin beta 4	HtrA2 peptidase	WEVISDEH
gi|50592996|ref|NP_006077.2| tubulin beta 4	HtrA2 peptidase	GFQLTHSL
gi|50592996|ref|NP_006077.2| tubulin beta 4	HtrA2 peptidase	THSLGGGT
gi|50592996|ref|NP_006077.2| tubulin beta 4	HtrA2 peptidase	LRKLAVNM
gi|50592996|ref|NP_006077.2| tubulin beta 4	HtrA2 peptidase	FPRLHFFM
gi|50592996|ref|NP_006077.2| tubulin beta 4	HtrA2 peptidase	GHYTEGAE
gi|50592996|ref|NP_006077.2| tubulin beta 4	HtrA2 peptidase	GNNWAKGH
gi|50592996|ref|NP_006077.2| tubulin beta 4	HtrA2 peptidase	NHLVSATM
gi|50592996|ref|NP_006077.2| tubulin beta 4	HtrA2 peptidase	AILVDLEP
gi|50592996|ref|NP_006077.2| tubulin beta 4	HtrA2 peptidase	THSLGGGT
gi|50592996|ref|NP_006077.2| tubulin beta 4	HtrA2 peptidase	FQLTHSLG
gi|50301240|ref|NP_001001998.1| exosome component 10 isoform 1	granzyme B	VEQDMFAH
gi|115298678|ref|NP_000055.2| complement component 3 precursor	cathepsin G	GLARSNLD
gi|115298678|ref|NP_000055.2| complement component 3 precursor	cathepsin G	RSNLDEDI
gi|115298678|ref|NP_000055.2| complement component 3 precursor	complement component C2a	GLARSNLD
gi|115298678|ref|NP_000055.2| complement component 3 precursor	complement factor B	GLARSNLD
gi|115298678|ref|NP_000055.2| complement component 3 precursor	complement factor I	LPSRSSKI
gi|115298678|ref|NP_000055.2| complement component 3 precursor	complement factor I	SLLRSEET
gi|4503627|ref|NP_000119.1| platelet coagulation factor XI precursor	coagulation factor XIIa	IKPRIVGG
gi|4503627|ref|NP_000119.1| platelet coagulation factor XI precursor	coagulation factor XIa	IKPRIVGG
gi|4503627|ref|NP_000119.1| platelet coagulation factor XI precursor	thrombin	IKPRIVGG
gi|13027802|ref|NP_005932.2| matrix metalloproteinase 16 isoform 1 preproprotein	plasmin	KKPRCGVP
gi|4501885|ref|NP_001092.1| beta actin	HtrA2 peptidase	LCYVALDF
gi|4501885|ref|NP_001092.1| beta actin	elastase-2	HQGVMVGM
gi|25777724|ref|NP_003879.2| aldehyde dehydrogenase 1A2 isoform 1	HtrA2 peptidase	IDKIAFTG
gi|67089149|ref|NP_150241.2| promyelocytic leukemia protein isoform 1	elastase-2	PMAVVQSV

gi|67089149|ref|NP_150241.2| promyelocytic leukemia protein isoform 1	elastase-2	PVPVYAFS
gi|71834857|ref|NP_001025220.1| prostate specific antigen isoform 5 preproprotein	kallikrein-related peptidase 15	ILSRIVGG
gi|71834857|ref|NP_001025220.1| prostate specific antigen isoform 5 preproprotein	kallikrein-related peptidase 2	ILSRIVGG
gi|189217853|ref|NP_001121363.1| matrix metalloproteinase 2 isoform b	trypsin-2	ETMRKPRC
gi|189217853|ref|NP_001121363.1| matrix metalloproteinase 2 isoform b	elastase-2	PETAMSTV
gi|189217853|ref|NP_001121363.1| matrix metalloproteinase 2 isoform b	elastase-2	ATTANYDD
gi|189217853|ref|NP_001121363.1| matrix metalloproteinase 2 isoform b	elastase-2	LGPVTPEI
gi|4506475|ref|NP_000528.1| renin precursor	coagulation factor XIIa	PMKRLTLG
gi|17865802|ref|NP_004860.2| vacuolar protein sorting factor 4B	HtrA2 peptidase	ILPIKFPH
gi|57013276|ref|NP_006073.2| tubulin alpha ubiquitous	HtrA2 peptidase	VPRAVFVD
gi|57013276|ref|NP_006073.2| tubulin alpha ubiquitous	HtrA2 peptidase	VFVDLEPT
gi|57013276|ref|NP_006073.2| tubulin alpha ubiquitous	HtrA2 peptidase	HSDCAFMV
gi|57013276|ref|NP_006073.2| tubulin alpha ubiquitous	HtrA2 peptidase	VAEITNAC
gi|57013276|ref|NP_006073.2| tubulin alpha ubiquitous	HtrA2 peptidase	PEQLITGK
gi|57013276|ref|NP_006073.2| tubulin alpha ubiquitous	HtrA2 peptidase	GEGMEEGE
gi|57013276|ref|NP_006073.2| tubulin alpha ubiquitous	HtrA2 peptidase	VDLTEFQT
gi|57013276|ref|NP_006073.2| tubulin alpha ubiquitous	HtrA2 peptidase	FSETGAGK
gi|57013276|ref|NP_006073.2| tubulin alpha ubiquitous	HtrA2 peptidase	FVHWYVGE
gi|57013276|ref|NP_006073.2| tubulin alpha ubiquitous	HtrA2 peptidase	GFLVFHSF
gi|57013276|ref|NP_006073.2| tubulin alpha ubiquitous	HtrA2 peptidase	AFMVDNEA
gi|57013276|ref|NP_006073.2| tubulin alpha ubiquitous	HtrA2 peptidase	PKDVNAAI
gi|57013276|ref|NP_006073.2| tubulin alpha ubiquitous	HtrA2 peptidase	GFKVGINY
gi|57013276|ref|NP_006073.2| tubulin alpha ubiquitous	HtrA2 peptidase	PRAVFVDL
gi|57013276|ref|NP_006073.2| tubulin alpha ubiquitous	HtrA2 peptidase	AVFVDLEP
gi|62414289|ref|NP_003371.2| vimentin	HtrA2 peptidase	TYSLGSAL
gi|4503471|ref|NP_001393.1| eukaryotic translation elongation factor 1 alpha 1	HtrA2 peptidase	LQDVYKIG
gi|41327764|ref|NP_003680.2| aldo-keto reductase family 7 member A2	thrombin	FGLRFYAY
gi|88853069|ref|NP_000629.3| vitronectin precursor	plasmin	KGYRSQRG
gi|71834859|ref|NP_001025221.1| prostate specific antigen isoform 6 preproprotein	kallikrein-related peptidase 15	ILSRIVGG
gi|71834859|ref|NP_001025221.1| prostate specific antigen isoform 6 preproprotein	kallikrein-related peptidase 2	ILSRIVGG
gi|6996014|ref|NP_002100.2| histidyl-tRNA synthetase	granzyme B	LGPDESKQ
gi|73858566|ref|NP_000176.2| heparin cofactor II precursor	elastase-2	LEKIFSED
gi|73858566|ref|NP_000176.2| heparin cofactor II precursor	elastase-2	VTTVGFMP

gi|73858566|ref|NP_000176.2| heparin cofactor II precursor	cathepsin G	LEKIFSED
gi|73858566|ref|NP_000176.2| heparin cofactor II precursor	cathepsin G	VTTVGFMP
gi|13435353|ref|NP_076493.1| L1 cell adhesion molecule isoform 2 precursor	plasmin	SQRKHSKR
gi|13435353|ref|NP_076493.1| L1 cell adhesion molecule isoform 2 precursor	plasmin	KHSKRHIH
gi|50363219|ref|NP_001002236.1| serine proteinase inhibitor clade A member 1	mesotrypsin	AAQKTDTS
gi|34577052|ref|NP_005233.3| coagulation factor II (thrombin) receptor-like 1 precursor	elastase-2	GKLTTVFL
gi|34577052|ref|NP_005233.3| coagulation factor II (thrombin) receptor-like 1 precursor	elastase-2	IGKVDGTS
gi|34577052|ref|NP_005233.3| coagulation factor II (thrombin) receptor-like 1 precursor	elastase-2	TSHVTGKG
gi|34577052|ref|NP_005233.3| coagulation factor II (thrombin) receptor-like 1 precursor	elastase-2	GKGVTVET
gi|34577052|ref|NP_005233.3| coagulation factor II (thrombin) receptor-like 1 precursor	elastase-2	VETVFSVD
gi|34577052|ref|NP_005233.3| coagulation factor II (thrombin) receptor-like 1 precursor	elastase-2	LTTVFLPI
gi|34577052|ref|NP_005233.3| coagulation factor II (thrombin) receptor-like 1 precursor	cathepsin G	GRSLIGKV
gi|34577052|ref|NP_005233.3| coagulation factor II (thrombin) receptor-like 1 precursor	cathepsin G	ETVFSVDE
gi|34577052|ref|NP_005233.3| coagulation factor II (thrombin) receptor-like 1 precursor	cathepsin G	VDEFSASV
gi|34577052|ref|NP_005233.3| coagulation factor II (thrombin) receptor-like 1 precursor	myeloblastin	FSVDEFSA
gi|34577052|ref|NP_005233.3| coagulation factor II (thrombin) receptor-like 1 precursor	myeloblastin	LTGKLTTV
gi|34577052|ref|NP_005233.3| coagulation factor II (thrombin) receptor-like 1 precursor	myeloblastin	TVETVFSV
gi|34577052|ref|NP_005233.3| coagulation factor II (thrombin) receptor-like 1 precursor	myeloblastin	GKLTTVFL
gi|34577052|ref|NP_005233.3| coagulation factor II (thrombin) receptor-like 1 precursor	myeloblastin	KLTTVFLP
gi|34577052|ref|NP_005233.3| coagulation factor II (thrombin) receptor-like 1 precursor	myeloblastin	TSHVTGKG
gi|34577052|ref|NP_005233.3| coagulation factor II (thrombin) receptor-like 1 precursor	myeloblastin	GVTVETVF
gi|34577052|ref|NP_005233.3| coagulation factor II (thrombin) receptor-like 1 precursor	myeloblastin	VFSVDEFS
gi|34577052|ref|NP_005233.3| coagulation factor II (thrombin) receptor-like 1 precursor	myeloblastin	LTTVFLPI
gi|34577052|ref|NP_005233.3| coagulation factor II (thrombin) receptor-like 1 precursor	thrombin	SKGRSLIG

gi|34577052|ref|NP_005233.3| coagulation factor II (thrombin) receptor-like 1 precursor	plasmin	SKGRSLIG
gi|34577052|ref|NP_005233.3| coagulation factor II (thrombin) receptor-like 1 precursor	plasmin	RSSKGRSL
gi|4502173|ref|NP_001639.1| prostate specific antigen isoform 1 preproprotein	kallikrein-related peptidase 15	ILSRIVGG
gi|4502173|ref|NP_001639.1| prostate specific antigen isoform 1 preproprotein	kallikrein-related peptidase 2	ILSRIVGG
gi|166362740|ref|NP_001983.2| coagulation factor II receptor precursor	elastase-2	ATNATLDP
gi|166362740|ref|NP_001983.2| coagulation factor II receptor precursor	elastase-2	QLPAFISE
gi|166362740|ref|NP_001983.2| coagulation factor II receptor precursor	elastase-2	YRLVSINK
gi|166362740|ref|NP_001983.2| coagulation factor II receptor precursor	cathepsin G	LDPRSFLL
gi|166362740|ref|NP_001983.2| coagulation factor II receptor precursor	cathepsin G	PRSFLLRN
gi|166362740|ref|NP_001983.2| coagulation factor II receptor precursor	cathepsin G	YEPFWEDE
gi|166362740|ref|NP_001983.2| coagulation factor II receptor precursor	cathepsin G	LTEYRLVS
gi|166362740|ref|NP_001983.2| coagulation factor II receptor precursor	myeloblastin	ATNATLDP
gi|166362740|ref|NP_001983.2| coagulation factor II receptor precursor	myeloblastin	SEDASGYL
gi|166362740|ref|NP_001983.2| coagulation factor II receptor precursor	myeloblastin	LRNPNDKY
gi|166362740|ref|NP_001983.2| coagulation factor II receptor precursor	myeloblastin	KYEPFWED
gi|166362740|ref|NP_001983.2| coagulation factor II receptor precursor	myeloblastin	YRLVSINK
gi|166362740|ref|NP_001983.2| coagulation factor II receptor precursor	thrombin	ARTRARRP
gi|166362740|ref|NP_001983.2| coagulation factor II receptor precursor	thrombin	LDPRSFLL
gi|166362740|ref|NP_001983.2| coagulation factor II receptor precursor	thrombin	PRSFLLRN
gi|166362740|ref|NP_001983.2| coagulation factor II receptor precursor	plasmin	LDPRSFLL
gi|166362740|ref|NP_001983.2| coagulation factor II receptor precursor	plasmin	TEYRLVSI
gi|166362740|ref|NP_001983.2| coagulation factor II receptor precursor	plasmin	PESKATNA
gi|166362740|ref|NP_001983.2| coagulation factor II receptor precursor	plasmin	SINKSSPL
gi|55770868|ref|NP_064424.3| tubulin beta polypeptide 4 member Q	HtrA2 peptidase	WEVISDEH

gi|55770868|ref|NP_064424.3| tubulin beta polypeptide 4 member Q	HtrA2 peptidase	GFQLTHSL
gi|55770868|ref|NP_064424.3| tubulin beta polypeptide 4 member Q	HtrA2 peptidase	THSLGGGT
gi|55770868|ref|NP_064424.3| tubulin beta polypeptide 4 member Q	HtrA2 peptidase	FPRLHFFM
gi|55770868|ref|NP_064424.3| tubulin beta polypeptide 4 member Q	HtrA2 peptidase	NHLVSATM
gi|55770868|ref|NP_064424.3| tubulin beta polypeptide 4 member Q	HtrA2 peptidase	THSLGGGT
gi|55770868|ref|NP_064424.3| tubulin beta polypeptide 4 member Q	HtrA2 peptidase	FQLTHSLG
gi|189163542|ref|NP_001121179.1| serine proteinase inhibitor clade A member 1	mesotrypsin	AAQKTDTS
gi|7019569|ref|NP_037377.1| vacuolar protein sorting factor 4A	HtrA2 peptidase	ILPIKFPH
gi|10835159|ref|NP_000593.1| plasminogen activator inhibitor-1	matriptase-3	VSARMAPE
gi|153266841|ref|NP_000033.2| apolipoprotein H precursor	coagulation factor Xa	AFWKTDAS
gi|153266841|ref|NP_000033.2| apolipoprotein H precursor	plasmin	AFWKTDAS
gi|18375503|ref|NP_542379.1| APEX nuclease	granzyme A	TAAKKNDK
gi|4506147|ref|NP_002761.1| protease serine 2 preproprotein	enteropeptidase	DDDKIVGG
gi|4503625|ref|NP_000495.1| coagulation factor X preproprotein	cathepsin G	PFDLLDFN
gi|4503625|ref|NP_000495.1| coagulation factor X preproprotein	coagulation factor IXa	NLTRIVGG
gi|4503625|ref|NP_000495.1| coagulation factor X preproprotein	coagulation factor VIIa	NLTRIVGG
gi|4503625|ref|NP_000495.1| coagulation factor X preproprotein	plasmin	THEKGRQS
gi|4503625|ref|NP_000495.1| coagulation factor X preproprotein	plasmin	PKAKSHAP
gi|103471997|ref|NP_056240.2| polymerase (RNA) I polypeptide A 194kDa	granzyme B	ICPDMYIN
gi|4503645|ref|NP_000122.1| coagulation factor VII isoform a precursor	coagulation factor XIIa	PQGRIVGG
gi|4503645|ref|NP_000122.1| coagulation factor VII isoform a precursor	coagulation factor IXa	PQGRIVGG
gi|4503645|ref|NP_000122.1| coagulation factor VII isoform a precursor	coagulation factor VIIa	PQGRIVGG
gi|4503645|ref|NP_000122.1| coagulation factor VII isoform a precursor	coagulation factor Xa	PQGRIVGG
gi|4503645|ref|NP_000122.1| coagulation factor VII isoform a precursor	thrombin	PQGRIVGG
gi|4503645|ref|NP_000122.1| coagulation factor VII isoform a precursor	hepsin	PQGRIVGG

gi|154800437|ref|NP_001946.3| endothelin 1	chymase	VVPYGLGS
gi|154800437|ref|NP_001946.3| endothelin 1	cathepsin G	FCHLDIIW
gi|154800437|ref|NP_001946.3| endothelin 1	cathepsin G	CVYFCHLD
gi|154800437|ref|NP_001946.3| endothelin 1	cathepsin G	DIIWVNTP
gi|154800437|ref|NP_001946.3| endothelin 1	chymase	VVPYGLGS
gi|18375505|ref|NP_542380.1| APEX nuclease	granzyme A	TAAKKNDK
gi|91206462|ref|NP_001035147.1| secreted phosphoprotein 1 isoform a	thrombin	YGLRSKSK
gi|109638749|ref|NP_008878.3| signal recognition particle 72kDa	granzyme B	VTPDPERW
gi|134133226|ref|NP_001077007.1| protein expressed in prostate ovary testis and placenta 2	HtrA2 peptidase	LCYVALDF
gi|169167253|ref|XP_001713916.1| PREDICTED: similar to tubulin beta polypeptide 4 member Q	HtrA2 peptidase	WEVISDEH
gi|169167253|ref|XP_001713916.1| PREDICTED: similar to tubulin beta polypeptide 4 member Q	HtrA2 peptidase	GFQLTHSL
gi|169167253|ref|XP_001713916.1| PREDICTED: similar to tubulin beta polypeptide 4 member Q	HtrA2 peptidase	THSLGGGT
gi|169167253|ref|XP_001713916.1| PREDICTED: similar to tubulin beta polypeptide 4 member Q	HtrA2 peptidase	TTCLRFPG
gi|169167253|ref|XP_001713916.1| PREDICTED: similar to tubulin beta polypeptide 4 member Q	HtrA2 peptidase	LRKLAVNM
gi|169167253|ref|XP_001713916.1| PREDICTED: similar to tubulin beta polypeptide 4 member Q	HtrA2 peptidase	THSLGGGT
gi|169167253|ref|XP_001713916.1| PREDICTED: similar to tubulin beta polypeptide 4 member Q	HtrA2 peptidase	FQLTHSLG
gi|13562114|ref|NP_110400.1| beta tubulin 1 class VI	HtrA2 peptidase	TLKLTTPT
gi|13562114|ref|NP_110400.1| beta tubulin 1 class VI	HtrA2 peptidase	LRKLAVNM
gi|13562114|ref|NP_110400.1| beta tubulin 1 class VI	HtrA2 peptidase	FPRLHFFM
gi|13562114|ref|NP_110400.1| beta tubulin 1 class VI	HtrA2 peptidase	GHYTEGAE
gi|13562114|ref|NP_110400.1| beta tubulin 1 class VI	HtrA2 peptidase	TTPTYGDL
gi|13562114|ref|NP_110400.1| beta tubulin 1 class VI	HtrA2 peptidase	GNNWAKGH
gi|4502167|ref|NP_000475.1| amyloid beta A4 protein precursor isoform a	kallikrein-related peptidase 6	AEFRHDSG
gi|4502167|ref|NP_000475.1| amyloid beta A4 protein precursor isoform a	kallikrein-related peptidase 6	HHQKLVFF
gi|4502167|ref|NP_000475.1| amyloid beta A4 protein precursor isoform a	kallikrein-related peptidase 6	SEVKMDAE
gi|4502167|ref|NP_000475.1| amyloid beta A4 protein precursor isoform a	HtrA1 peptidase	MQQNGYEN
gi|4502167|ref|NP_000475.1| amyloid beta A4 protein precursor isoform a	HtrA1 peptidase	VKMDAEFR
gi|4502167|ref|NP_000475.1| amyloid beta A4 protein precursor isoform a	HtrA1 peptidase	VHHQKLVF
gi|4502167|ref|NP_000475.1| amyloid beta A4 protein precursor isoform a	HtrA1 peptidase	GYEVHHQK
gi|39753970|ref|NP_004336.2| cathelicidin antimicrobial peptide	myeloblastin	KRFALLGD
gi|115583663|ref|NP_000925.2| alpha-2-plasmin inhibitor	matriptase-3	AMSRMSLS

gi|115583663|ref|NP_000925.2| alpha-2-plasmin inhibitor	plasmin	AMSRMSLS
gi|4759166|ref|NP_000573.1| secreted phosphoprotein 1 isoform b	thrombin	YGLRSKSK
gi|190341074|ref|NP_055842.2| pyridoxal-dependent decarboxylase domain containing 1	HtrA2 peptidase	LLLVANAG
gi|4503689|ref|NP_000499.1| fibrinogen alpha polypeptide isoform alpha-E preproprotein	thrombin	GGVRGPRV
gi|41406055|ref|NP_958816.1| amyloid beta A4 protein precursor isoform b	kallikrein-related peptidase 6	AEFRHDSG
gi|41406055|ref|NP_958816.1| amyloid beta A4 protein precursor isoform b	kallikrein-related peptidase 6	HHQKLVFF
gi|41406055|ref|NP_958816.1| amyloid beta A4 protein precursor isoform b	kallikrein-related peptidase 6	SEVKMDAE
gi|41406055|ref|NP_958816.1| amyloid beta A4 protein precursor isoform b	HtrA1 peptidase	MQQNGYEN
gi|41406055|ref|NP_958816.1| amyloid beta A4 protein precursor isoform b	HtrA1 peptidase	VKMDAEFR
gi|41406055|ref|NP_958816.1| amyloid beta A4 protein precursor isoform b	HtrA1 peptidase	VHHQKLVF
gi|41406055|ref|NP_958816.1| amyloid beta A4 protein precursor isoform b	HtrA1 peptidase	GYEVHHQK
gi|4506885|ref|NP_002999.1| semenogelin II precursor	kallikrein-related peptidase 3	LLVYNKNQ
gi|4506885|ref|NP_002999.1| semenogelin II precursor	kallikrein-related peptidase 3	SQQYDLNA
gi|4506885|ref|NP_002999.1| semenogelin II precursor	kallikrein-related peptidase 3	ISYQSSST
gi|4506885|ref|NP_002999.1| semenogelin II precursor	kallikrein-related peptidase 3	ISYQSSST
gi|4506885|ref|NP_002999.1| semenogelin II precursor	kallikrein-related peptidase 3	RRLNSGEK
gi|4506885|ref|NP_002999.1| semenogelin II precursor	kallikrein-related peptidase 3	GHYQNVVD
gi|4506885|ref|NP_002999.1| semenogelin II precursor	kallikrein-related peptidase 3	SKLQTSLH
gi|4506885|ref|NP_002999.1| semenogelin II precursor	kallikrein-related peptidase 3	SQNQVTIH
gi|4506885|ref|NP_002999.1| semenogelin II precursor	kallikrein-related peptidase 3	IPSQAQEY
gi|4506885|ref|NP_002999.1| semenogelin II precursor	kallikrein-related peptidase 3	ISYQSSST
gi|4506885|ref|NP_002999.1| semenogelin II precursor	kallikrein-related peptidase 3	MSYQSSST
gi|4506885|ref|NP_002999.1| semenogelin II precursor	kallikrein-related peptidase 3	SKQHLGGS
gi|4506885|ref|NP_002999.1| semenogelin II precursor	kallikrein-related peptidase 3	QSSYVLQT
gi|4506885|ref|NP_002999.1| semenogelin II precursor	kallikrein-related peptidase 3	RLNYGGKS

gi|4506885|ref|NP_002999.1| semenogelin II precursor	kallikrein-related peptidase 3	SQQYDLNA
gi|4506885|ref|NP_002999.1| semenogelin II precursor	kallikrein-related peptidase 2	TEKRLWVH
gi|4506885|ref|NP_002999.1| semenogelin II precursor	kallikrein-related peptidase 2	HQDRLQHG
gi|4506885|ref|NP_002999.1| semenogelin II precursor	kallikrein-related peptidase 2	PSSRTEER
gi|4506885|ref|NP_002999.1| semenogelin II precursor	kallikrein-related peptidase 2	NQVRIPSQ
gi|4506885|ref|NP_002999.1| semenogelin II precursor	kallikrein-related peptidase 2	EERRLNYG
gi|4506885|ref|NP_002999.1| semenogelin II precursor	kallikrein-related peptidase 2	QKGRYKQE
gi|4506885|ref|NP_002999.1| semenogelin II precursor	kallikrein-related peptidase 2	GHFHMIVI
gi|4506885|ref|NP_002999.1| semenogelin II precursor	kallikrein-related peptidase 3	ISYQSSST
gi|4506885|ref|NP_002999.1| semenogelin II precursor	kallikrein-related peptidase 3	HPAHQDRL
gi|4506885|ref|NP_002999.1| semenogelin II precursor	kallikrein-related peptidase 3	RQLHHGEK
gi|4506885|ref|NP_002999.1| semenogelin II precursor	kallikrein-related peptidase 3	SIQHTYHV
gi|4506885|ref|NP_002999.1| semenogelin II precursor	kallikrein-related peptidase 3	GQHYFGQK
gi|24476013|ref|NP_722560.1| PTK2 protein tyrosine kinase 2 isoform a	granzyme B	VSWDSGGS
gi|4759070|ref|NP_004157.1| chemokine (C-C motif) ligand 14 isoform 1 precursor	u-plasminogen activator	SSSRGPYH
gi|41406057|ref|NP_958817.1| amyloid beta A4 protein precursor isoform c	kallikrein-related peptidase 6	AEFRHDSG
gi|41406057|ref|NP_958817.1| amyloid beta A4 protein precursor isoform c	kallikrein-related peptidase 6	HHQKLVFF
gi|41406057|ref|NP_958817.1| amyloid beta A4 protein precursor isoform c	kallikrein-related peptidase 6	SEVKMDAE
gi|41406057|ref|NP_958817.1| amyloid beta A4 protein precursor isoform c	HtrA1 peptidase	MQQNGYEN
gi|41406057|ref|NP_958817.1| amyloid beta A4 protein precursor isoform c	HtrA1 peptidase	VKMDAEFR
gi|41406057|ref|NP_958817.1| amyloid beta A4 protein precursor isoform c	HtrA1 peptidase	VHHQKLVF
gi|41406057|ref|NP_958817.1| amyloid beta A4 protein precursor isoform c	HtrA1 peptidase	GYEVHHQK
gi|21536452|ref|NP_002762.2| mesotrypsin isoform 2 preproprotein	enteropeptidase	DDDKIVGG
gi|178557739|ref|NP_001002029.3| complement component 4B preproprotein	complement component activated C1s	GLQRALEI

gi|178557739|ref|NP_001002029.3| complement component 4B preproprotein	mannan-binding lectin-associated serine peptidase	GLQRALEI
gi|178557739|ref|NP_001002029.3| complement component 4B preproprotein	mannan-binding lectin-associated serine peptidase	GLQRALEI
gi|178557739|ref|NP_001002029.3| complement component 4B preproprotein	mannan-binding lectin-associated serine peptidase	GLQRALEI
gi|178557739|ref|NP_001002029.3| complement component 4B preproprotein	complement factor I	STGRNGFK
gi|178557739|ref|NP_001002029.3| complement component 4B preproprotein	complement factor I	HRGRTLEI
gi|11761629|ref|NP_068657.1| fibrinogen alpha polypeptide isoform alpha preproprotein	thrombin	GGVRGPRV
gi|4885049|ref|NP_005150.1| cardiac muscle alpha actin 1 proprotein	HtrA2 peptidase	PTLLTEAP
gi|4885049|ref|NP_005150.1| cardiac muscle alpha actin 1 proprotein	HtrA2 peptidase	TLLTEAPL
gi|4885049|ref|NP_005150.1| cardiac muscle alpha actin 1 proprotein	HtrA2 peptidase	SGLVKAGF
gi|4885049|ref|NP_005150.1| cardiac muscle alpha actin 1 proprotein	HtrA2 peptidase	LCYVALDF
gi|4885049|ref|NP_005150.1| cardiac muscle alpha actin 1 proprotein	elastase-2	HQGVMVGM
gi|40548408|ref|NP_954675.1| p30 DBC protein	HtrA2 peptidase	LFQTSHTL
gi|40548408|ref|NP_954675.1| p30 DBC protein	HtrA2 peptidase	LLHVAALG
gi|12056465|ref|NP_001427.2| fibrillarin	granzyme B	VGPDGLVY
gi|52630322|ref|NP_005843.2| chromodomain helicase DNA binding protein 3 isoform 2	granzyme B	VDPDYWEK
gi|109637788|ref|NP_150247.2| promyelocytic leukemia protein isoform 5	elastase-2	PMAVVQSV
gi|109637788|ref|NP_150247.2| promyelocytic leukemia protein isoform 5	elastase-2	PVPVYAFS
gi|91598939|ref|NP_001035149.1| secreted phosphoprotein 1 isoform c	thrombin	YGLRSKSK
gi|189163540|ref|NP_001121178.1| serine proteinase inhibitor clade A member 1	mesotrypsin	AAQKTDTS
gi|170296790|ref|NP_031369.2| mesotrypsin isoform 1 preproprotein	enteropeptidase	DDDKIVGG
gi|4502495|ref|NP_001725.1| complement component 1 s subcomponent	complement component activated C1r	EKQRIIGG
gi|4505863|ref|NP_002649.1| urokinase plasminogen activator preproprotein	elastase-2	RFKIIGGE
gi|4505863|ref|NP_002649.1| urokinase plasminogen activator preproprotein	cathepsin G	PRFKIIGG
gi|4505863|ref|NP_002649.1| urokinase plasminogen activator preproprotein	kallikrein-related peptidase 2	PRFKIIGG

gi|4505863|ref|NP_002649.1| urokinase plasminogen activator preproprotein	kallikrein-related peptidase 3	PRFKIIGG
gi|4505863|ref|NP_002649.1| urokinase plasminogen activator preproprotein	plasma kallikrein	PRFKIIGG
gi|4505863|ref|NP_002649.1| urokinase plasminogen activator preproprotein	thrombin	LRPRFKII
gi|4505863|ref|NP_002649.1| urokinase plasminogen activator preproprotein	u-plasminogen activator	VSNKYFSN
gi|4505863|ref|NP_002649.1| urokinase plasminogen activator preproprotein	plasmin	ADGKKPSS
gi|4505863|ref|NP_002649.1| urokinase plasminogen activator preproprotein	plasmin	PRFKIIGG
gi|4505863|ref|NP_002649.1| urokinase plasminogen activator preproprotein	hepsin	PRFKIIGG
gi|41393602|ref|NP_958850.1| complement component 1 s subcomponent	complement component activated C1r	EKQRIIGG
gi|109148522|ref|NP_001035835.1| insulin- insulin-like growth factor 2	elastase-2	SHLVEALY
gi|109148522|ref|NP_001035835.1| insulin- insulin-like growth factor 2	elastase-2	LVEALYLV
gi|109148522|ref|NP_001035835.1| insulin- insulin-like growth factor 2	cathepsin G	GSHLVEAL
gi|109148522|ref|NP_001035835.1| insulin- insulin-like growth factor 2	elastase-1	SHLVEALY
gi|109148522|ref|NP_001035835.1| insulin- insulin-like growth factor 2	elastase-1	LVEALYLV
gi|109148522|ref|NP_001035835.1| insulin- insulin-like growth factor 2	elastase-1	GERGFFYT
gi|109148522|ref|NP_001035835.1| insulin- insulin-like growth factor 2	chymotrypsin C	ERGFFYTP
gi|109148522|ref|NP_001035835.1| insulin- insulin-like growth factor 2	chymotrypsin C	RGFFYTPK
gi|109148522|ref|NP_001035835.1| insulin- insulin-like growth factor 2	kallikrein-related peptidase 7	RGFFYTPK
gi|14602459|ref|NP_005647.2| transmembrane protease serine 2	epitheliasin	RQSRIVGG
gi|4504893|ref|NP_000884.1| kininogen 1 isoform 2	elastase-2	LGMISLMK
gi|4504893|ref|NP_000884.1| kininogen 1 isoform 2	elastase-2	SSRIGEIK
gi|4504893|ref|NP_000884.1| kininogen 1 isoform 2	kallikrein 1	SPFRSSRI
gi|4504893|ref|NP_000884.1| kininogen 1 isoform 2	kallikrein 1	ISLMKRPP
gi|4504893|ref|NP_000884.1| kininogen 1 isoform 2	kallikrein-related peptidase 2	SPFRSSRI
gi|4504893|ref|NP_000884.1| kininogen 1 isoform 2	kallikrein-related peptidase 2	SLMKRPPG
gi|4504893|ref|NP_000884.1| kininogen 1 isoform 2	plasma kallikrein	SPFRSSRI
gi|4504893|ref|NP_000884.1| kininogen 1 isoform 2	plasma kallikrein	SLMKRPPG
gi|4504619|ref|NP_001544.1| insulin-like growth factor binding protein 7	matriptase	RKGKAGAA

gi|189163530|ref|NP_001121173.1| serine proteinase inhibitor clade A member 1	mesotrypsin	AAQKTDTS
gi|62243248|ref|NP_001013416.1| insulin-like growth factor binding protein 3 isoform a precursor	kallikrein-related peptidase 3	SQRYKVDY
gi|62243248|ref|NP_001013416.1| insulin-like growth factor binding protein 3 isoform a precursor	thrombin	SRLRAYLL
gi|62243248|ref|NP_001013416.1| insulin-like growth factor binding protein 3 isoform a precursor	thrombin	LSPRGVHI
gi|62243248|ref|NP_001013416.1| insulin-like growth factor binding protein 3 isoform a precursor	plasmin	AVSRLRAY
gi|62243248|ref|NP_001013416.1| insulin-like growth factor binding protein 3 isoform a precursor	plasmin	DSQRYKVD
gi|62243248|ref|NP_001013416.1| insulin-like growth factor binding protein 3 isoform a precursor	plasmin	HPLHSKII
gi|62243248|ref|NP_001013416.1| insulin-like growth factor binding protein 3 isoform a precursor	plasmin	QRYKVDYE
gi|62243248|ref|NP_001013416.1| insulin-like growth factor binding protein 3 isoform a precursor	plasmin	NHLKFLNV
gi|62243248|ref|NP_001013416.1| insulin-like growth factor binding protein 3 isoform a precursor	plasmin	GFYKKKQC
gi|62243248|ref|NP_001013416.1| insulin-like growth factor binding protein 3 isoform a precursor	elastase-2	IIIIKKGH
gi|62243248|ref|NP_001013416.1| insulin-like growth factor binding protein 3 isoform a precursor	elastase-2	PRGVHIPN
gi|62243248|ref|NP_001013416.1| insulin-like growth factor binding protein 3 isoform a precursor	cathepsin G	FHPLHSKI
gi|62243248|ref|NP_001013416.1| insulin-like growth factor binding protein 3 isoform a precursor	cathepsin G	HLKFLNVL
gi|62243248|ref|NP_001013416.1| insulin-like growth factor binding protein 3 isoform a precursor	plasmin	SRLRAYLL
gi|194018472|ref|NP_000615.3| serine (or cysteine) proteinase inhibitor clade A (alpha-1 antiproteinase antitrypsin) member 5	matriptase-3	FTFRSARL
gi|15451765|ref|NP_150242.1| promyelocytic leukemia protein isoform 9	elastase-2	PMAVVQSV
gi|15451765|ref|NP_150242.1| promyelocytic leukemia protein isoform 9	elastase-2	PVPVYAFS
gi|17978495|ref|NP_510867.1| cyclin-dependent kinase inhibitor 1A	myeloblastin	YLPTGPRR
gi|4502581|ref|NP_001218.1| caspase 7 isoform alpha precursor	granzyme B	IQADSGPI
gi|38201623|ref|NP_937884.1| eukaryotic translation initiation factor 4 gamma 1 isoform 1	HtrA2 peptidase	PSQISYPA
gi|19923193|ref|NP_003923.2| heat shock 70kD protein binding protein	granzyme B	IEPDTDAP
gi|19923193|ref|NP_003923.2| heat shock 70kD protein binding protein	thrombin	VQPRAQKI
gi|11386203|ref|NP_000380.1| cyclin-dependent kinase inhibitor 1A	myeloblastin	YLPTGPRR

gi|110349772|ref|NP_000079.2| alpha 1 type I collagen preproprotein	chymase	LKSLSQQI
gi|9507215|ref|NP_061816.1| tubulin alpha 8	HtrA2 peptidase	HSDCAFMV
gi|9507215|ref|NP_061816.1| tubulin alpha 8	HtrA2 peptidase	PEQLITGK
gi|9507215|ref|NP_061816.1| tubulin alpha 8	HtrA2 peptidase	GEGMEEGE
gi|9507215|ref|NP_061816.1| tubulin alpha 8	HtrA2 peptidase	VDLTEFQT
gi|9507215|ref|NP_061816.1| tubulin alpha 8	HtrA2 peptidase	FVHWYVGE
gi|9507215|ref|NP_061816.1| tubulin alpha 8	HtrA2 peptidase	AFMVDNEA
gi|9507215|ref|NP_061816.1| tubulin alpha 8	HtrA2 peptidase	GFKVGINY
gi|15718700|ref|NP_203125.1| caspase 7 isoform alpha	granzyme B	IQADSGPI
gi|68509928|ref|NP_001020261.1| myelin basic protein isoform 3	cathepsin G	VHFFKNIV
gi|68509928|ref|NP_001020261.1| myelin basic protein isoform 3	cathepsin G	LSRFSWGA
gi|169217560|ref|XP_001722858.1| PREDICTED: similar to hCG2001591	complement factor I	STGRNGFK
gi|169216498|ref|XP_001732909.1| PREDICTED: hypothetical LOC389901	granzyme B	ISSDRDLL
gi|4557287|ref|NP_000020.1| angiotensinogen preproprotein	chymase	DRVYIHPF
gi|4557287|ref|NP_000020.1| angiotensinogen preproprotein	cathepsin G	DRVYIHPF
gi|4557287|ref|NP_000020.1| angiotensinogen preproprotein	elastase-2	FHLVIHNE
gi|4557287|ref|NP_000020.1| angiotensinogen preproprotein	cathepsin G	IHPFHLVI
gi|4557287|ref|NP_000020.1| angiotensinogen preproprotein	myeloblastin	FHLVIHNE
gi|4501887|ref|NP_001605.1| actin gamma 1 propeptide	HtrA2 peptidase	LCYVALDF
gi|4501887|ref|NP_001605.1| actin gamma 1 propeptide	elastase-2	HQGVMVGM
gi|66347875|ref|NP_001724.3| complement component 1 r subcomponent	complement component activated C1r	QRQRIIGG
gi|15718698|ref|NP_203124.1| caspase 7 isoform delta	granzyme B	IQADSGPI
gi|38201627|ref|NP_937887.1| eukaryotic translation initiation factor 4 gamma 1 isoform 2	HtrA2 peptidase	PSQISYPA
gi|52630326|ref|NP_001005273.1| chromodomain helicase DNA binding protein 3 isoform 1	granzyme B	VDPDYWEK
gi|13654237|ref|NP_008835.5| protein kinase DNA-activated catalytic polypeptide isoform 1	granzyme B	VGPDFGKK
gi|13654237|ref|NP_008835.5| protein kinase DNA-activated catalytic polypeptide isoform 1	granzyme B	DEVDNKVK
gi|150417978|ref|NP_000164.4| platelet glycoprotein Ib alpha polypeptide precursor	cathepsin G	DTDLYDYY
gi|4505123|ref|NP_002376.1| myelin basic protein isoform 2	cathepsin G	VHFFKNIV

gi|33859835|ref|NP_000592.3| hepatocyte growth factor isoform 1 preproprotein	coagulation factor XIa	KQLRVVNG
gi|33859835|ref|NP_000592.3| hepatocyte growth factor isoform 1 preproprotein	hepatocyte growth factor activator	KQLRVVNG
gi|33859835|ref|NP_000592.3| hepatocyte growth factor isoform 1 preproprotein	plasma kallikrein	DLHRHIFW
gi|33859835|ref|NP_000592.3| hepatocyte growth factor isoform 1 preproprotein	plasma kallikrein	KQLRVVNG
gi|33859835|ref|NP_000592.3| hepatocyte growth factor isoform 1 preproprotein	coagulation factor XIa	DLHRHIFW
gi|33859835|ref|NP_000592.3| hepatocyte growth factor isoform 1 preproprotein	hepsin	KQLRVVNG
gi|63055057|ref|NP_001017992.1| actin beta-like 2	HtrA2 peptidase	LCYVALDF
gi|63055057|ref|NP_001017992.1| actin beta-like 2	elastase-2	HQGVMVGM
gi|38201621|ref|NP_886553.2| eukaryotic translation initiation factor 4 gamma 1 isoform 1	HtrA2 peptidase	PSQISYPA
gi|5174735|ref|NP_006079.1| tubulin beta 2	HtrA2 peptidase	WEVISDEH
gi|5174735|ref|NP_006079.1| tubulin beta 2	HtrA2 peptidase	GFQLTHSL
gi|5174735|ref|NP_006079.1| tubulin beta 2	HtrA2 peptidase	THSLGGGT
gi|5174735|ref|NP_006079.1| tubulin beta 2	HtrA2 peptidase	TLKLTTPT
gi|5174735|ref|NP_006079.1| tubulin beta 2	HtrA2 peptidase	TTCLRFPG
gi|5174735|ref|NP_006079.1| tubulin beta 2	HtrA2 peptidase	LRKLAVNM
gi|5174735|ref|NP_006079.1| tubulin beta 2	HtrA2 peptidase	FPRLHFFM
gi|5174735|ref|NP_006079.1| tubulin beta 2	HtrA2 peptidase	GHYTEGAE
gi|5174735|ref|NP_006079.1| tubulin beta 2	HtrA2 peptidase	TTPTYGDL
gi|5174735|ref|NP_006079.1| tubulin beta 2	HtrA2 peptidase	GNNWAKGH
gi|5174735|ref|NP_006079.1| tubulin beta 2	HtrA2 peptidase	NHLVSATM
gi|5174735|ref|NP_006079.1| tubulin beta 2	HtrA2 peptidase	THSLGGGT
gi|5174735|ref|NP_006079.1| tubulin beta 2	HtrA2 peptidase	FQLTHSLG
gi|21735415|ref|NP_001801.1| centromere protein B	granzyme B	VDSDEEEE
gi|4503635|ref|NP_000497.1| coagulation factor II preproprotein	coagulation factor Xa	FNPRTFGS
gi|4503635|ref|NP_000497.1| coagulation factor II preproprotein	coagulation factor Xa	IDGRIVEG
gi|4503635|ref|NP_000497.1| coagulation factor II preproprotein	thrombin	MTPRSEGS
gi|4503635|ref|NP_000497.1| coagulation factor II preproprotein	thrombin	FNPRTFGS
gi|4503635|ref|NP_000497.1| coagulation factor II preproprotein	thrombin	IDGRIVEG
gi|4503635|ref|NP_000497.1| coagulation factor II preproprotein	coagulation factor Xa	IDGRIVEG
gi|4503635|ref|NP_000497.1| coagulation factor II preproprotein	coagulation factor Xa	IDGRIVEG
gi|192447438|ref|NP_000304.2| protein S alpha	thrombin	PDLRSCVN
gi|4501883|ref|NP_001604.1| alpha 2 actin	HtrA2 peptidase	PTLLTEAP
gi|4501883|ref|NP_001604.1| alpha 2 actin	HtrA2 peptidase	TLLTEAPL
gi|4501883|ref|NP_001604.1| alpha 2 actin	HtrA2 peptidase	LCYVALDF

gi|4501883|ref|NP_001604.1| alpha 2 actin	elastase-2	HQGVMVGM
gi|17921993|ref|NP_005992.1| tubulin alpha 3c	HtrA2 peptidase	VPRAVFVD
gi|17921993|ref|NP_005992.1| tubulin alpha 3c	HtrA2 peptidase	VFVDLEPT
gi|17921993|ref|NP_005992.1| tubulin alpha 3c	HtrA2 peptidase	HSDCAFMV
gi|17921993|ref|NP_005992.1| tubulin alpha 3c	HtrA2 peptidase	VAEITNAC
gi|17921993|ref|NP_005992.1| tubulin alpha 3c	HtrA2 peptidase	PEQLITGK
gi|17921993|ref|NP_005992.1| tubulin alpha 3c	HtrA2 peptidase	GEGMEEGE
gi|17921993|ref|NP_005992.1| tubulin alpha 3c	HtrA2 peptidase	VDLTEFQT
gi|17921993|ref|NP_005992.1| tubulin alpha 3c	HtrA2 peptidase	FSETGAGK
gi|17921993|ref|NP_005992.1| tubulin alpha 3c	HtrA2 peptidase	FVHWYVGE
gi|17921993|ref|NP_005992.1| tubulin alpha 3c	HtrA2 peptidase	AFMVDNEA
gi|17921993|ref|NP_005992.1| tubulin alpha 3c	HtrA2 peptidase	PKDVNAAI
gi|17921993|ref|NP_005992.1| tubulin alpha 3c	HtrA2 peptidase	GFKVGINY
gi|17921993|ref|NP_005992.1| tubulin alpha 3c	HtrA2 peptidase	PRAVFVDL
gi|17921993|ref|NP_005992.1| tubulin alpha 3c	HtrA2 peptidase	AVFVDLEP
gi|58533170|ref|NP_001010932.1| hepatocyte growth factor isoform 3 precursor	coagulation factor XIa	KQLRVVNG
gi|58533170|ref|NP_001010932.1| hepatocyte growth factor isoform 3 precursor	hepatocyte growth factor activator	KQLRVVNG
gi|58533170|ref|NP_001010932.1| hepatocyte growth factor isoform 3 precursor	plasma kallikrein	DLHRHIFW
gi|58533170|ref|NP_001010932.1| hepatocyte growth factor isoform 3 precursor	plasma kallikrein	KQLRVVNG
gi|58533170|ref|NP_001010932.1| hepatocyte growth factor isoform 3 precursor	coagulation factor XIa	DLHRHIFW
gi|58533170|ref|NP_001010932.1| hepatocyte growth factor isoform 3 precursor	hepsin	KQLRVVNG
gi|29788768|ref|NP_821080.1| tubulin beta 2B	HtrA2 peptidase	WEVISDEH
gi|29788768|ref|NP_821080.1| tubulin beta 2B	HtrA2 peptidase	GFQLTHSL
gi|29788768|ref|NP_821080.1| tubulin beta 2B	HtrA2 peptidase	THSLGGGT
gi|29788768|ref|NP_821080.1| tubulin beta 2B	HtrA2 peptidase	TLKLTTPT
gi|29788768|ref|NP_821080.1| tubulin beta 2B	HtrA2 peptidase	TTCLRFPG
gi|29788768|ref|NP_821080.1| tubulin beta 2B	HtrA2 peptidase	LRKLAVNM
gi|29788768|ref|NP_821080.1| tubulin beta 2B	HtrA2 peptidase	FPRLHFFM
gi|29788768|ref|NP_821080.1| tubulin beta 2B	HtrA2 peptidase	GHYTEGAE
gi|29788768|ref|NP_821080.1| tubulin beta 2B	HtrA2 peptidase	TTPTYGDL
gi|29788768|ref|NP_821080.1| tubulin beta 2B	HtrA2 peptidase	GNNWAKGH
gi|29788768|ref|NP_821080.1| tubulin beta 2B	HtrA2 peptidase	NHLVSATM
gi|29788768|ref|NP_821080.1| tubulin beta 2B	HtrA2 peptidase	AILVDLEP
gi|29788768|ref|NP_821080.1| tubulin beta 2B	HtrA2 peptidase	THSLGGGT
gi|29788768|ref|NP_821080.1| tubulin beta 2B	HtrA2 peptidase	FQLTHSLG
gi|5729929|ref|NP_006681.1| matrix metalloproteinase 24 preproprotein	plasmin	KKPRCGVP
gi|46430499|ref|NP_068810.2| v-rel reticuloendotheliosis viral oncogene homolog A	elastase-2	RSSASVPK
gi|46430499|ref|NP_068810.2| v-rel reticuloendotheliosis viral oncogene homolog A	myeloblastin	GKDCRDGF

gi|24308089|ref|NP_056372.1| chromodomain helicase DNA binding protein 5	granzyme B	VDPDYWEK
gi|10835067|ref|NP_003133.1| autoantigen La	granzyme B	LEEDAEMK
gi|68509930|ref|NP_001020252.1| myelin basic protein isoform 1	cathepsin G	VHFFKNIV
gi|68509930|ref|NP_001020252.1| myelin basic protein isoform 1	cathepsin G	LSRFSWGA
gi|169208124|ref|XP_001718698.1| PREDICTED: hypothetical protein	HtrA2 peptidase	LTQQVFDA
gi|17986283|ref|NP_006000.2| tubulin alpha 1a	HtrA2 peptidase	VPRAVFVD
gi|17986283|ref|NP_006000.2| tubulin alpha 1a	HtrA2 peptidase	VFVDLEPT
gi|17986283|ref|NP_006000.2| tubulin alpha 1a	HtrA2 peptidase	HSDCAFMV
gi|17986283|ref|NP_006000.2| tubulin alpha 1a	HtrA2 peptidase	VAEITNAC
gi|17986283|ref|NP_006000.2| tubulin alpha 1a	HtrA2 peptidase	PEQLITGK
gi|17986283|ref|NP_006000.2| tubulin alpha 1a	HtrA2 peptidase	GEGMEEGE
gi|17986283|ref|NP_006000.2| tubulin alpha 1a	HtrA2 peptidase	VDLTEFQT
gi|17986283|ref|NP_006000.2| tubulin alpha 1a	HtrA2 peptidase	FSETGAGK
gi|17986283|ref|NP_006000.2| tubulin alpha 1a	HtrA2 peptidase	FVHWYVGE
gi|17986283|ref|NP_006000.2| tubulin alpha 1a	HtrA2 peptidase	GFLVFHSF
gi|17986283|ref|NP_006000.2| tubulin alpha 1a	HtrA2 peptidase	AFMVDNEA
gi|17986283|ref|NP_006000.2| tubulin alpha 1a	HtrA2 peptidase	PKDVNAAI
gi|17986283|ref|NP_006000.2| tubulin alpha 1a	HtrA2 peptidase	GFKVGINY
gi|17986283|ref|NP_006000.2| tubulin alpha 1a	HtrA2 peptidase	PRAVFVDL
gi|17986283|ref|NP_006000.2| tubulin alpha 1a	HtrA2 peptidase	AVFVDLEP
gi|4505903|ref|NP_002666.1| promyelocytic leukemia protein isoform 6	elastase-2	PMAVVQSV
gi|4505903|ref|NP_002666.1| promyelocytic leukemia protein isoform 6	elastase-2	PVPVYAFS
gi|4502261|ref|NP_000479.1| serine (or cysteine) proteinase inhibitor clade C (antithrombin) member 1	matriptase-3	IAGRSLNP
gi|4502261|ref|NP_000479.1| serine (or cysteine) proteinase inhibitor clade C (antithrombin) member 1	elastase-2	AVVIAGRS
gi|4502261|ref|NP_000479.1| serine (or cysteine) proteinase inhibitor clade C (antithrombin) member 1	thrombin	IAGRSLNP
gi|156564363|ref|NP_525125.2| tubulin alpha 3d	HtrA2 peptidase	VPRAVFVD
gi|156564363|ref|NP_525125.2| tubulin alpha 3d	HtrA2 peptidase	VFVDLEPT
gi|156564363|ref|NP_525125.2| tubulin alpha 3d	HtrA2 peptidase	HSDCAFMV
gi|156564363|ref|NP_525125.2| tubulin alpha 3d	HtrA2 peptidase	VAEITNAC
gi|156564363|ref|NP_525125.2| tubulin alpha 3d	HtrA2 peptidase	PEQLITGK
gi|156564363|ref|NP_525125.2| tubulin alpha 3d	HtrA2 peptidase	GEGMEEGE
gi|156564363|ref|NP_525125.2| tubulin alpha 3d	HtrA2 peptidase	VDLTEFQT
gi|156564363|ref|NP_525125.2| tubulin alpha 3d	HtrA2 peptidase	FSETGAGK
gi|156564363|ref|NP_525125.2| tubulin alpha 3d	HtrA2 peptidase	FVHWYVGE
gi|156564363|ref|NP_525125.2| tubulin alpha 3d	HtrA2 peptidase	AFMVDNEA
gi|156564363|ref|NP_525125.2| tubulin alpha 3d	HtrA2 peptidase	PKDVNAAI
gi|156564363|ref|NP_525125.2| tubulin alpha 3d	HtrA2 peptidase	GFKVGINY
gi|156564363|ref|NP_525125.2| tubulin alpha 3d	HtrA2 peptidase	PRAVFVDL
gi|156564363|ref|NP_525125.2| tubulin alpha 3d	HtrA2 peptidase	AVFVDLEP

gi|10518503|ref|NP_062562.1| coagulation factor VII isoform b precursor	coagulation factor XIIa	PQGRIVGG
gi|10518503|ref|NP_062562.1| coagulation factor VII isoform b precursor	coagulation factor IXa	PQGRIVGG
gi|10518503|ref|NP_062562.1| coagulation factor VII isoform b precursor	coagulation factor VIIa	PQGRIVGG
gi|10518503|ref|NP_062562.1| coagulation factor VII isoform b precursor	coagulation factor Xa	PQGRIVGG
gi|10518503|ref|NP_062562.1| coagulation factor VII isoform b precursor	thrombin	PQGRIVGG
gi|10518503|ref|NP_062562.1| coagulation factor VII isoform b precursor	hepsin	PQGRIVGG
gi|40316924|ref|NP_954637.1| chemokine (C-X-C motif) ligand 12 (stromal cell-derived factor 1) isoform alpha	cathepsin G	PVSLSYRC
gi|50363221|ref|NP_001002235.1| serine proteinase inhibitor clade A member 1	mesotrypsin	AAQKTDTS
gi|4758504|ref|NP_004484.1| hydroxysteroid (17-beta) dehydrogenase 10 isoform 1	HtrA2 peptidase	GNNCVFAP
gi|156523968|ref|NP_001609.2| poly (ADP-ribose) polymerase family member 1	cathepsin G	RMKLTLKG
gi|156523968|ref|NP_001609.2| poly (ADP-ribose) polymerase family member 1	cathepsin G	EAVKKLTV
gi|83715985|ref|NP_001032900.1| hydroxysteroid (17-beta) dehydrogenase 10 isoform 2	HtrA2 peptidase	GNNCVFAP
gi|103472005|ref|NP_002408.3| antigen identified by monoclonal antibody Ki-67	granzyme B	VCTDKPTT
gi|4505939|ref|NP_000928.1| DNA directed RNA polymerase II polypeptide A	granzyme B	ITPDPNLS
gi|153791352|ref|NP_001093241.1| prostate ovary testis expressed protein on chromosome 2	HtrA2 peptidase	LCYVALDF
gi|94721241|ref|NP_002152.2| isoleucyl-tRNA synthetase	granzyme B	VTPDQSMV
gi|66932947|ref|NP_000005.2| alpha-2-macroglobulin precursor	chymase	RVGFYESD
gi|66932947|ref|NP_000005.2| alpha-2-macroglobulin precursor	chymase	VGFYESDV
gi|66932947|ref|NP_000005.2| alpha-2-macroglobulin precursor	elastase-1	LRVGFYES
gi|66932947|ref|NP_000005.2| alpha-2-macroglobulin precursor	elastase-1	GLRVGFYE
gi|66932947|ref|NP_000005.2| alpha-2-macroglobulin precursor	thrombin	GHARLVHV
gi|66932947|ref|NP_000005.2| alpha-2-macroglobulin precursor	plasmin	GHARLVHV
gi|169209397|ref|XP_001713955.1| PREDICTED: similar to hCG1647990	HtrA2 peptidase	LTQQVFDA
gi|169214179|ref|XP_001724196.1| PREDICTED: similar to complement component 3	complement factor I	LPSRSSKI
gi|169214179|ref|XP_001724196.1| PREDICTED: similar to complement component 3	complement factor I	SLLRSEET

gi|4503841|ref|NP_001460.1| ATP-dependent DNA helicase II 70 kDa subunit	granzyme B	ISSDRDLL
gi|67782358|ref|NP_001701.2| complement factor B preproprotein	complement factor D	QQKRKIVL
gi|27886593|ref|NP_005598.3| PTK2 protein tyrosine kinase 2 isoform b	granzyme B	VSWDSGGS
gi|76563933|ref|NP_001029058.1| chemokine (C-X-C motif) ligand 12 (stromal cell-derived factor 1) isoform gamma	cathepsin G	PVSLSYRC
gi|4507729|ref|NP_001060.1| tubulin beta 2	HtrA2 peptidase	WEVISDEH
gi|4507729|ref|NP_001060.1| tubulin beta 2	HtrA2 peptidase	GFQLTHSL
gi|4507729|ref|NP_001060.1| tubulin beta 2	HtrA2 peptidase	THSLGGGT
gi|4507729|ref|NP_001060.1| tubulin beta 2	HtrA2 peptidase	TLKLTTPT
gi|4507729|ref|NP_001060.1| tubulin beta 2	HtrA2 peptidase	TTCLRFPG
gi|4507729|ref|NP_001060.1| tubulin beta 2	HtrA2 peptidase	LRKLAVNM
gi|4507729|ref|NP_001060.1| tubulin beta 2	HtrA2 peptidase	FPRLHFFM
gi|4507729|ref|NP_001060.1| tubulin beta 2	HtrA2 peptidase	GHYTEGAE
gi|4507729|ref|NP_001060.1| tubulin beta 2	HtrA2 peptidase	TTPTYGDL
gi|4507729|ref|NP_001060.1| tubulin beta 2	HtrA2 peptidase	GNNWAKGH
gi|4507729|ref|NP_001060.1| tubulin beta 2	HtrA2 peptidase	NHLVSATM
gi|4507729|ref|NP_001060.1| tubulin beta 2	HtrA2 peptidase	AILVDLEP
gi|4507729|ref|NP_001060.1| tubulin beta 2	HtrA2 peptidase	THSLGGGT
gi|4507729|ref|NP_001060.1| tubulin beta 2	HtrA2 peptidase	FQLTHSLG
gi|10834982|ref|NP_000590.1| insulin-like growth factor binding protein 5	thrombin	AHPRIISA
gi|10834982|ref|NP_000590.1| insulin-like growth factor binding protein 5	thrombin	MVPRAVYL
gi|10834982|ref|NP_000590.1| insulin-like growth factor binding protein 5	thrombin	FRPKHTRI
gi|14702169|ref|NP_127509.1| plasminogen activator tissue type isoform 3 preproprotein	plasmin	PQFRIKGG
gi|18105007|ref|NP_004332.2| carbamoylphosphate synthetase 2/aspartate transcarbamylase/dihydroorotase	HtrA2 peptidase	AMNVAYTD
gi|4505881|ref|NP_000292.1| plasminogen	kallikrein-related peptidase 3	SGTEASVV
gi|4505881|ref|NP_000292.1| plasminogen	u-plasminogen activator	CPGRVVGG
gi|4505881|ref|NP_000292.1| plasminogen	t-plasminogen activator	CPGRVVGG
gi|4505881|ref|NP_000292.1| plasminogen	plasmin	GNGKGYRG
gi|4505881|ref|NP_000292.1| plasminogen	plasmin	LFEKKVYL
gi|62243068|ref|NP_000589.2| insulin-like growth factor binding protein 3 isoform b precursor	kallikrein-related peptidase 3	SQRYKVDY
gi|62243068|ref|NP_000589.2| insulin-like growth factor binding protein 3 isoform b precursor	thrombin	SRLRAYLL
gi|62243068|ref|NP_000589.2| insulin-like growth factor binding protein 3 isoform b precursor	thrombin	LSPRGVHI
gi|62243068|ref|NP_000589.2| insulin-like growth factor binding protein 3 isoform b precursor	plasmin	AVSRLRAY

gi|62243068|ref|NP_000589.2| insulin-like growth factor binding protein 3 isoform b precursor	plasmin	DSQRYKVD
gi|62243068|ref|NP_000589.2| insulin-like growth factor binding protein 3 isoform b precursor	plasmin	HPLHSKII
gi|62243068|ref|NP_000589.2| insulin-like growth factor binding protein 3 isoform b precursor	plasmin	QRYKVDYE
gi|62243068|ref|NP_000589.2| insulin-like growth factor binding protein 3 isoform b precursor	plasmin	NHLKFLNV
gi|62243068|ref|NP_000589.2| insulin-like growth factor binding protein 3 isoform b precursor	plasmin	GFYKKKQC
gi|62243068|ref|NP_000589.2| insulin-like growth factor binding protein 3 isoform b precursor	elastase-2	IIIIKKGH
gi|62243068|ref|NP_000589.2| insulin-like growth factor binding protein 3 isoform b precursor	elastase-2	PRGVHIPN
gi|62243068|ref|NP_000589.2| insulin-like growth factor binding protein 3 isoform b precursor	cathepsin G	FHPLHSKI
gi|62243068|ref|NP_000589.2| insulin-like growth factor binding protein 3 isoform b precursor	cathepsin G	HLKFLNVL
gi|62243068|ref|NP_000589.2| insulin-like growth factor binding protein 3 isoform b precursor	plasmin	SRLRAYLL
gi|126273559|ref|NP_057497.3| plasma carboxypeptidase B2 isoform b	thrombin	VSPRASAS
gi|4503647|ref|NP_000123.1| coagulation factor VIII isoform a precursor	thrombin	QSPRSFQK
gi|4503647|ref|NP_000123.1| coagulation factor VIII isoform a precursor	thrombin	IQIRSVAK
gi|4503647|ref|NP_000123.1| coagulation factor VIII isoform a precursor	thrombin	IEPRSFSQ
gi|4503647|ref|NP_000123.1| coagulation factor VIII isoform a precursor	protein C (activated)	PQLRMKNN
gi|4503647|ref|NP_000123.1| coagulation factor VIII isoform a precursor	protein C (activated)	VDQRGNQI
gi|4503647|ref|NP_000123.1| coagulation factor VIII isoform a precursor	protein C (activated)	IEPRSFSQ
gi|4503463|ref|NP_001947.1| endothelin 2	cathepsin G	FCHLDIIW
gi|4503463|ref|NP_001947.1| endothelin 2	cathepsin G	CVYFCHLD
gi|4503463|ref|NP_001947.1| endothelin 2	cathepsin G	DIIWVNTP
gi|4503463|ref|NP_001947.1| endothelin 2	chymase	TAPYGLGN
gi|105990535|ref|NP_000121.2| coagulation factor V precursor	elastase-2	VIPANMDK
gi|105990535|ref|NP_000121.2| coagulation factor V precursor	elastase-2	PLVIVGLS
gi|105990535|ref|NP_000121.2| coagulation factor V precursor	elastase-2	RRGIQRAA
gi|105990535|ref|NP_000121.2| coagulation factor V precursor	elastase-2	ALGIRSFR
gi|105990535|ref|NP_000121.2| coagulation factor V precursor	elastase-2	VTGIRLLS

gi|105990535|ref|NP_000121.2| coagulation factor V precursor	elastase-2	WRLTSSEM
gi|105990535|ref|NP_000121.2| coagulation factor V precursor	cathepsin G	SLLLQEFN
gi|105990535|ref|NP_000121.2| coagulation factor V precursor	cathepsin G	YNTFSERR
gi|105990535|ref|NP_000121.2| coagulation factor V precursor	cathepsin G	YVPYDDPY
gi|105990535|ref|NP_000121.2| coagulation factor V precursor	coagulation factor VIIa	LSPRTFHP
gi|105990535|ref|NP_000121.2| coagulation factor V precursor	coagulation factor VIIa	LIQRNLSP
gi|105990535|ref|NP_000121.2| coagulation factor V precursor	coagulation factor VIIa	MATRKMHD
gi|105990535|ref|NP_000121.2| coagulation factor V precursor	coagulation factor VIIa	LGIRSFRN
gi|105990535|ref|NP_000121.2| coagulation factor V precursor	thrombin	LSPRTFHP
gi|105990535|ref|NP_000121.2| coagulation factor V precursor	thrombin	WYLRSNNG
gi|105990535|ref|NP_000121.2| coagulation factor V precursor	thrombin	LGIRSFRN
gi|105990535|ref|NP_000121.2| coagulation factor V precursor	protein C (activated)	KKTRNLKK
gi|105990535|ref|NP_000121.2| coagulation factor V precursor	protein C (activated)	LDRRGIQR
gi|105990535|ref|NP_000121.2| coagulation factor V precursor	protein C (activated)	MATRKMHD
gi|105990535|ref|NP_000121.2| coagulation factor V precursor	protein C (activated)	RLKKSQFL
gi|149999365|ref|NP_072086.2| matrix metalloproteinase 16 isoform 2 preproprotein	plasmin	KKPRCGVP
gi|17921989|ref|NP_005991.1| tubulin alpha 4a	HtrA2 peptidase	VPRAVFVD
gi|17921989|ref|NP_005991.1| tubulin alpha 4a	HtrA2 peptidase	VFVDLEPT
gi|17921989|ref|NP_005991.1| tubulin alpha 4a	HtrA2 peptidase	HSDCAFMV
gi|17921989|ref|NP_005991.1| tubulin alpha 4a	HtrA2 peptidase	VAEITNAC
gi|17921989|ref|NP_005991.1| tubulin alpha 4a	HtrA2 peptidase	PEQLITGK
gi|17921989|ref|NP_005991.1| tubulin alpha 4a	HtrA2 peptidase	GEGMEEGE
gi|17921989|ref|NP_005991.1| tubulin alpha 4a	HtrA2 peptidase	VDLTEFQT
gi|17921989|ref|NP_005991.1| tubulin alpha 4a	HtrA2 peptidase	FVHWYVGE
gi|17921989|ref|NP_005991.1| tubulin alpha 4a	HtrA2 peptidase	GFLVFHSF
gi|17921989|ref|NP_005991.1| tubulin alpha 4a	HtrA2 peptidase	AFMVDNEA
gi|17921989|ref|NP_005991.1| tubulin alpha 4a	HtrA2 peptidase	PKDVNAAI
gi|17921989|ref|NP_005991.1| tubulin alpha 4a	HtrA2 peptidase	GFKVGINY
gi|17921989|ref|NP_005991.1| tubulin alpha 4a	HtrA2 peptidase	PRAVFVDL
gi|17921989|ref|NP_005991.1| tubulin alpha 4a	HtrA2 peptidase	AVFVDLEP
gi|10834988|ref|NP_000600.1| chemokine (C-X-C motif) ligand 12 (stromal cell-derived factor 1) isoform beta	cathepsin G	PVSLSYRC

gi|68509932|ref|NP_001020263.1| myelin basic protein isoform 4	cathepsin G	VHFFKNIV
gi|4505861|ref|NP_000921.1| plasminogen activator tissue type isoform 1 preproprotein	plasmin	PQFRIKGG
gi|50659080|ref|NP_001076.2| serpin peptidase inhibitor clade A member 3 precursor	elastase-2	VKITLLSA
gi|56243551|ref|NP_000340.2| steroidogenic acute regulatory protein isoform 1	PIM1 peptidase	RMEAMGEW
gi|56243551|ref|NP_000340.2| steroidogenic acute regulatory protein isoform 1	PIM1 peptidase	SIDLKGWL
gi|56243551|ref|NP_000340.2| steroidogenic acute regulatory protein isoform 1	PIM1 peptidase	NQVLSQTQ
gi|56117848|ref|NP_001007244.1| steroidogenic acute regulatory protein isoform 2	PIM1 peptidase	RMEAMGEW
gi|56117848|ref|NP_001007244.1| steroidogenic acute regulatory protein isoform 2	PIM1 peptidase	SIDLKGWL
gi|56117848|ref|NP_001007244.1| steroidogenic acute regulatory protein isoform 2	PIM1 peptidase	NQVLSQTQ
gi|21264365|ref|NP_057404.2| nucleoporin 98kD isoform 1	nucleoporin 145	VSHFSKYG
gi|56549643|ref|NP_005378.4| nucleoporin 98kD isoform 3	nucleoporin 145	VSHFSKYG
gi|56549645|ref|NP_624358.2| nucleoporin 98kD isoform 4	nucleoporin 145	VSHFSKYG
gi|21264369|ref|NP_624357.1| nucleoporin 98kD isoform 2	nucleoporin 145	VSHFSKYG
gi|23397681|ref|NP_038475.2| egf-like module containing mucin-like hormone receptor-like sequence 2 isoform a	EGF-like module containing mucin-like hormone rece	CTHLSSFA
gi|23397683|ref|NP_690880.1| egf-like module containing mucin-like hormone receptor-like sequence 2 isoform b	EGF-like module containing mucin-like hormone rece	CTHLSSFA
gi|23397685|ref|NP_690881.1| egf-like module containing mucin-like hormone receptor-like sequence 2 isoform c	EGF-like module containing mucin-like hormone rece	CTHLSSFA
gi|23397687|ref|NP_690882.1| egf-like module containing mucin-like hormone receptor-like sequence 2 isoform d	EGF-like module containing mucin-like hormone rece	CTHLSSFA
gi|23397689|ref|NP_690883.1| egf-like module containing mucin-like hormone receptor-like sequence 2 isoform e	EGF-like module containing mucin-like hormone rece	CTHLSSFA
gi|23397691|ref|NP_690884.1| egf-like module containing mucin-like hormone receptor-like sequence 2 isoform f	EGF-like module containing mucin-like hormone rece	CTHLSSFA
gi|23397693|ref|NP_690885.1| egf-like module containing mucin-like hormone receptor-like sequence 2 isoform g	EGF-like module containing mucin-like hormone rece	CTHLSSFA

gi|61742782|ref|NP_060964.3| leucine rich repeat and death domain containing protein isoform 2	PIDD auto-processing protein unit 1	VPHFSWFL
gi|61742784|ref|NP_665893.2| leucine rich repeat and death domain containing protein isoform 1	PIDD auto-processing protein unit 1	VPHFSWFL
gi|61742784|ref|NP_665893.2| leucine rich repeat and death domain containing protein isoform 1	PIDD auto-processing protein unit 1	VTHFSWYW
gi|61742786|ref|NP_665894.2| leucine rich repeat and death domain containing protein isoform 3	PIDD auto-processing protein unit 1	VPHFSWFL
gi|61742786|ref|NP_665894.2| leucine rich repeat and death domain containing protein isoform 3	PIDD auto-processing protein unit 1	VTHFSWYW
gi|15451786|ref|NP_148937.1| platelet-derived growth factor beta isoform 2 preproprotein	furin	RGRRSLGS
gi|15451786|ref|NP_148937.1| platelet-derived growth factor beta isoform 2 preproprotein	PACE4 proprotein convertase	RGRRSLGS
gi|15451786|ref|NP_148937.1| platelet-derived growth factor beta isoform 2 preproprotein	proprotein convertase 5	RGRRSLGS
gi|15451786|ref|NP_148937.1| platelet-derived growth factor beta isoform 2 preproprotein	proprotein convertase 7	RGRRSLGS
gi|4502027|ref|NP_000468.1| albumin preproprotein	furin	VFRRDAHK
gi|4502027|ref|NP_000468.1| albumin preproprotein	proprotein convertase 1	VFRRDAHK
gi|4506115|ref|NP_000303.1| protein C (inactivator of coagulation factors Va and VIIIa)	furin	HLKRDTED
gi|4506115|ref|NP_000303.1| protein C (inactivator of coagulation factors Va and VIIIa)	furin	IRKRANSF
gi|4557671|ref|NP_000198.1| proinsulin precursor	proprotein convertase 1	KTRREAED
gi|4557671|ref|NP_000198.1| proinsulin precursor	proprotein convertase 1	LQKRGIVE
gi|4557671|ref|NP_000198.1| proinsulin precursor	proprotein convertase 2	LQKRGIVE
gi|4826834|ref|NP_004986.1| matrix metalloproteinase 14 preproprotein	furin	RRKRYAIQ
gi|63025222|ref|NP_000651.3| transforming growth factor beta 1	furin	RHRRALDT
gi|4503649|ref|NP_000124.1| coagulation factor IX preproprotein	furin	RPKRYNSG
gi|4506917|ref|NP_003011.1| secretory granule neuroendocrine protein 1 (7B2 protein)	furin	RKRRSVNP
gi|4506917|ref|NP_003011.1| secretory granule neuroendocrine protein 1 (7B2 protein)	proprotein convertase 2	VAKKSVPH
gi|126091152|ref|NP_001072.2| cubilin	furin	RQKRSINL
gi|89191868|ref|NP_000543.2| von Willebrand factor preproprotein	furin	RSKRSLSC

gi|89191868|ref|NP_000543.2| von Willebrand factor preproprotein	PACE4 proprotein convertase	RSKRSLSC
gi|25306235|ref|NP_733927.1| brain-derived neurotrophic factor isoform b preproprotein	site-1 peptidase	RGLTSLAD
gi|157276593|ref|NP_001193.2| bone morphogenetic protein 4 preproprotein	furin	RAKRSPKH
gi|157276593|ref|NP_001193.2| bone morphogenetic protein 4 preproprotein	PACE4 proprotein convertase	RAKRSPKH
gi|157276593|ref|NP_001193.2| bone morphogenetic protein 4 preproprotein	proprotein convertase 5	RAKRSPKH
gi|157276593|ref|NP_001193.2| bone morphogenetic protein 4 preproprotein	proprotein convertase 7	RAKRSPKH
gi|157419150|ref|NP_002836.3| protein tyrosine phosphatase receptor type M isoform 2 precursor	proprotein convertase 5	RPRRTKKT
gi|25306253|ref|NP_733928.1| brain-derived neurotrophic factor isoform a preproprotein	site-1 peptidase	RGLTSLAD
gi|24430141|ref|NP_000129.2| fibrillin 1 precursor	furin	RKRRSTNE
gi|25306257|ref|NP_733929.1| brain-derived neurotrophic factor isoform a preproprotein	site-1 peptidase	RGLTSLAD
gi|157276595|ref|NP_570911.2| bone morphogenetic protein 4 preproprotein	furin	RAKRSPKH
gi|157276595|ref|NP_570911.2| bone morphogenetic protein 4 preproprotein	PACE4 proprotein convertase	RAKRSPKH
gi|157276595|ref|NP_570911.2| bone morphogenetic protein 4 preproprotein	proprotein convertase 5	RAKRSPKH
gi|157276595|ref|NP_570911.2| bone morphogenetic protein 4 preproprotein	proprotein convertase 7	RAKRSPKH
gi|58331148|ref|NP_005931.2| matrix metalloproteinase 11 preproprotein	furin	RQKRFVLS
gi|25306261|ref|NP_733930.1| brain-derived neurotrophic factor isoform c preproprotein	site-1 peptidase	RGLTSLAD
gi|4505469|ref|NP_002518.1| neurotrophin 3 isoform 2 preproprotein	furin	RRKRYAEH
gi|4505469|ref|NP_002518.1| neurotrophin 3 isoform 2 preproprotein	PACE4 proprotein convertase	RRKRYAEH
gi|25306264|ref|NP_733931.1| brain-derived neurotrophic factor isoform a preproprotein	site-1 peptidase	RGLTSLAD
gi|25306267|ref|NP_001700.2| brain-derived neurotrophic factor isoform a preproprotein	site-1 peptidase	RGLTSLAD
gi|157276597|ref|NP_570912.2| bone morphogenetic protein 4 preproprotein	furin	RAKRSPKH
gi|157276597|ref|NP_570912.2| bone morphogenetic protein 4 preproprotein	PACE4 proprotein convertase	RAKRSPKH
gi|157276597|ref|NP_570912.2| bone morphogenetic protein 4 preproprotein	proprotein convertase 5	RAKRSPKH
gi|157276597|ref|NP_570912.2| bone morphogenetic protein 4 preproprotein	proprotein convertase 7	RAKRSPKH
gi|27477113|ref|NP_004590.2| sterol regulatory element-binding transcription factor 2	site-1 peptidase	RSVLSFES

gi|11342666|ref|NP_004521.1| matrix metalloproteinase 2 isoform a preproprotein	furin	RKPRCGNP
gi|156630995|ref|NP_001096124.1| neurotrophin 3 isoform 1 preproprotein	furin	RRKRYAEH
gi|156630995|ref|NP_001096124.1| neurotrophin 3 isoform 1 preproprotein	PACE4 proprotein convertase	RRKRYAEH
gi|189217853|ref|NP_001121363.1| matrix metalloproteinase 2 isoform b	furin	RKPRCGNP
gi|4506475|ref|NP_000528.1| renin precursor	proprotein convertase 1	PMKRLTLG
gi|4506475|ref|NP_000528.1| renin precursor	proprotein convertase 5	PMKRLTLG
gi|157419152|ref|NP_001098714.1| protein tyrosine phosphatase receptor type M isoform 1 precursor	proprotein convertase 5	RPRRTKKT
gi|4885653|ref|NP_005420.1| vascular endothelial growth factor C preproprotein	furin	IIRRSLPA
gi|4885653|ref|NP_005420.1| vascular endothelial growth factor C preproprotein	proprotein convertase 5	IIRRSLPA
gi|4885653|ref|NP_005420.1| vascular endothelial growth factor C preproprotein	proprotein convertase 7	IIRRSLPA
gi|56786157|ref|NP_031374.2| activating transcription factor 6	site-1 peptidase	RHLLGFSA
gi|80861463|ref|NP_001030333.1| proopiomelanocortin preproprotein	proprotein convertase 1	KDKRYGGF
gi|80861463|ref|NP_001030333.1| proopiomelanocortin preproprotein	proprotein convertase 1	EGKRSYSM
gi|80861463|ref|NP_001030333.1| proopiomelanocortin preproprotein	proprotein convertase 2	GKKRRPVK
gi|80861463|ref|NP_001030333.1| proopiomelanocortin preproprotein	proprotein convertase 2	KDKRYGGF
gi|80861463|ref|NP_001030333.1| proopiomelanocortin preproprotein	proprotein convertase 2	EGKRSYSM
gi|80861463|ref|NP_001030333.1| proopiomelanocortin preproprotein	proprotein convertase 2	ENPRKYVM
gi|4507243|ref|NP_001039.1| somatostatin preproprotein	proprotein convertase 1	RERKAGCK
gi|4507243|ref|NP_001039.1| somatostatin preproprotein	proprotein convertase 2	RERKAGCK
gi|154800437|ref|NP_001946.3| endothelin 1	proprotein convertase 7	KIRRSSEE
gi|154800437|ref|NP_001946.3| endothelin 1	proprotein convertase 7	RSKRCSCS
gi|154800437|ref|NP_001946.3| endothelin 1	proprotein convertase 7	RSKRALEN
gi|154800437|ref|NP_001946.3| endothelin 1	furin	RENRCQCA
gi|154800437|ref|NP_001946.3| endothelin 1	furin	KIRRSSEE
gi|154800437|ref|NP_001946.3| endothelin 1	furin	RSKRCSCS
gi|154800437|ref|NP_001946.3| endothelin 1	furin	RSKRALEN

gi|4505949|ref|NP_000930.1| proopiomelanocortin preproprotein	proprotein convertase 1	KDKRYGGF
gi|4505949|ref|NP_000930.1| proopiomelanocortin preproprotein	proprotein convertase 1	EGKRSYSM
gi|4505949|ref|NP_000930.1| proopiomelanocortin preproprotein	proprotein convertase 2	GKKRRPVK
gi|4505949|ref|NP_000930.1| proopiomelanocortin preproprotein	proprotein convertase 2	KDKRYGGF
gi|4505949|ref|NP_000930.1| proopiomelanocortin preproprotein	proprotein convertase 2	EGKRSYSM
gi|4505949|ref|NP_000930.1| proopiomelanocortin preproprotein	proprotein convertase 2	ENPRKYVM
gi|4505433|ref|NP_002512.1| natriuretic peptide precursor B preproprotein	furin	RAPRSPKM
gi|13270473|ref|NP_077722.1| beta-neoendorphin-dynorphin preproprotein	proprotein convertase 2	RRIRPKLK
gi|13270473|ref|NP_077722.1| beta-neoendorphin-dynorphin preproprotein	proprotein convertase 1	LYKRYGGF
gi|13270473|ref|NP_077722.1| beta-neoendorphin-dynorphin preproprotein	proprotein convertase 2	LYKRYGGF
gi|13270473|ref|NP_077722.1| beta-neoendorphin-dynorphin preproprotein	proprotein convertase 2	NQKRYGGF
gi|109148522|ref|NP_001035835.1| insulin- insulin-like growth factor 2	proprotein convertase 1	KTRREAED
gi|5453579|ref|NP_006120.1| bone morphogenetic protein 1 isoform 3 precursor	furin	RSRRAATS
gi|4506267|ref|NP_000306.1| parathyroid hormone preproprotein	furin	VKKRSVSE
gi|4506267|ref|NP_000306.1| parathyroid hormone preproprotein	proprotein convertase 7	VKKRSVSE
gi|6005920|ref|NP_009048.1| thyrotropin-releasing hormone	proprotein convertase 1	QHKRQHPG
gi|6005920|ref|NP_009048.1| thyrotropin-releasing hormone	proprotein convertase 1	VPKRQHPG
gi|6005920|ref|NP_009048.1| thyrotropin-releasing hormone	proprotein convertase 1	PEKRQHPG
gi|6005920|ref|NP_009048.1| thyrotropin-releasing hormone	proprotein convertase 1	EEKRQHPG
gi|6005920|ref|NP_009048.1| thyrotropin-releasing hormone	proprotein convertase 1	LSKRQHPG
gi|157427675|ref|NP_005090.3| ADAM metallopeptidase with thrombospondin type 1 motif 4 preproprotein	furin	RAKRFASL
gi|5902808|ref|NP_006119.1| bone morphogenetic protein 1 isoform 2 precursor	furin	RSRRAATS
gi|4502421|ref|NP_001190.1| bone morphogenetic protein 1 isoform 1 precursor	furin	RSRRAATS
gi|4503945|ref|NP_002045.1| glucagon preproprotein	proprotein convertase 1	RGRRDFPE

gi|4503945|ref|NP_002045.1| glucagon preproprotein	proprotein convertase 1	RGRRDFPE
gi|4503945|ref|NP_002045.1| glucagon preproprotein	proprotein convertase 1	RGRRDFPE
gi|4503945|ref|NP_002045.1| glucagon preproprotein	proprotein convertase 2	EDKRHSQG
gi|4503945|ref|NP_002045.1| glucagon preproprotein	proprotein convertase 2	EDKRHSQG
gi|4503945|ref|NP_002045.1| glucagon preproprotein	proprotein convertase 2	NTKRNRNN
gi|4503945|ref|NP_002045.1| glucagon preproprotein	proprotein convertase 2	EDKRHSQG
gi|4506775|ref|NP_003782.1| membrane-bound transcription factor site-1 protease preproprotein	site-1 peptidase	QKLLSIDL
gi|4506775|ref|NP_003782.1| membrane-bound transcription factor site-1 protease preproprotein	site-1 peptidase	RRLLRAIP
gi|4506775|ref|NP_003782.1| membrane-bound transcription factor site-1 protease preproprotein	site-1 peptidase	RSLKYAES
gi|4506775|ref|NP_003782.1| membrane-bound transcription factor site-1 protease preproprotein	site-1 peptidase	RKVFRSLK
gi|4505681|ref|NP_002599.1| platelet-derived growth factor beta isoform 1 preproprotein	furin	RGRRSLGS
gi|4505681|ref|NP_002599.1| platelet-derived growth factor beta isoform 1 preproprotein	PACE4 proprotein convertase	RGRRSLGS
gi|4505681|ref|NP_002599.1| platelet-derived growth factor beta isoform 1 preproprotein	proprotein convertase 5	RGRRSLGS
gi|4505681|ref|NP_002599.1| platelet-derived growth factor beta isoform 1 preproprotein	proprotein convertase 7	RGRRSLGS
gi|133908610|ref|NP_000816.3| gonadotropin-releasing hormone 1 precursor	proprotein convertase 2	GGKRDAEN
gi|133908612|ref|NP_001076580.1| gonadotropin-releasing hormone 1 precursor	proprotein convertase 2	GGKRDAEN
gi|5453876|ref|NP_006202.1| proenkephalin	proprotein convertase 1	VSKRYGGF
gi|5453876|ref|NP_006202.1| proenkephalin	proprotein convertase 1	GLKRSPQL
gi|5453876|ref|NP_006202.1| proenkephalin	proprotein convertase 1	YQKRYGGF
gi|5453876|ref|NP_006202.1| proenkephalin	proprotein convertase 1	FLKRFAEA
gi|5453876|ref|NP_006202.1| proenkephalin	proprotein convertase 1	MEKRYGGF
gi|5453876|ref|NP_006202.1| proenkephalin	proprotein convertase 1	FMKKDAEE
gi|5453876|ref|NP_006202.1| proenkephalin	proprotein convertase 2	VSKRYGGF
gi|5453876|ref|NP_006202.1| proenkephalin	proprotein convertase 2	GLKRSPQL

gi|5453876|ref|NP_006202.1| proenkephalin	proprotein convertase 2	YQKRYGGF
gi|5453876|ref|NP_006202.1| proenkephalin	proprotein convertase 2	FLKRFAEA
gi|5453876|ref|NP_006202.1| proenkephalin	proprotein convertase 2	MEKRYGGF
gi|13270473|ref|NP_077722.1| beta-neoendorphin-dynorphin preproprotein	prolyl oligopeptidase	RIRPKLKW
