# Supplementary material for: A Sequence and Structure Based Method to Predict Putative Substrates, Functions and Regulatory Networks of Endo Proteases
Source: PLoS One. 2009 May 27;4(5):e5700. doi: 10.1371/journal.pone.0005700 (PMC2683571; doi:10.1371/journal.pone.0005700)
Supplement: Table S5 — Relative Solvent Accessibility (rSASA) Values of Natural Substrates of Proteases (0.07 MB PDF) [file pone.0005700.s006.pdf]

**S5 -Relative solvent accessibility (rSASA) values of natural substrates of proteases**

# Aspartate Protease

| **Protein Name (PDB ID)** | **Protease Name** | **Octapeptide cleavage sequence** | **rSASA** | **Tetrapeptide cleavage sequence** | **rSASA** |
|---|---|---|---|---|---|
| *ALPHA1-ANTICHYMOTRYPSIN SERPIN (1QMN)* | | LLSALVET | 0.82 | LSAL | 2.00 |
| | | ITLLSALV | 2 | TLLS | 2.00 |
| | cathepsin D* | LSALVETR | 1 | SALV | 0.65 |
| | | | | | |
| *PROLACTIN ANTAGONIST (2Q98)* | cathepsin D* | YPVWSGLP | 0.52 | PVWS | 0.34 |
| | cathepsin E** | NEIYPVWS | 0.74 | EIYP | 0.42 |

# Cysteine Protease

| **Protein Name (PDB ID)** | **Protease Name** | **Octapeptide cleavage sequence** | **rSASA** | **Tetrapeptide cleavage sequence** | **rSASA** |
|---|---|---|---|---|---|
| *PROCATHEPSIN L (1CS8)* | | KVFQEPLF | 2 | VFQE | 0.46 |
| | | LFYEAPRS | 2 | FYEA | 0.62 |
| | cathepsin L *** | PLFYEAPR | 2 | LFYE | 0.68 |
| *SMALL G-PROTEIN (1MH1)* | caspase-3 | DLRDDKDT | 0.75 | LRDD | 0.38 |
| *ALPHA-1-ANTITRYPSIN (1QLP)* | | MFLEAIPM | 2 | FLEA | 0.66 |
| | cathepsin L *** | AIPMSIPP | 2 | IPMS | 0.59 |
| *HUMAN BCL-XL (1R2D)* | calpain-2 | WHLADSPA | 2 | HLAD | 2.00 |
| | caspase-1 | HLADSPAV | 2 | LADS | 2.00 |
| | | HLADSPAV | 2 | LADS | 2.00 |
| | caspase-3 | SSLDAREV | 2 | SLDA | 2.00 |
| | calpain-1 | EGTESEME | 2 | GTES | 2.00 |
| *BETA B2 CRYSTALLIN (1YTQ)* | calpain-1 | HQTQAGKP | 2 | QTQA | 2.00 |
| *EGFR KINASE DOMAIN (2ITV)* | calpain-1 | LWIPEGEK | 0.91 | WIPE | 0.31 |
| | | DEEDMDDV | 1 | EEDM | 0.77 |
| | caspase-3 | DMDDVVDA | 0.73 | MDDV | 0.49 |
| *P53DBD (2PCX)* | caspase-3 | SDSDGLAP | 0.78 | DSDG | 0.65 |
| *BETA-CATENIN (2Z6H)* | | YPVDGLPD | 2 | PVDG | 2.00 |
| | caspase-3 | DLMDGLPP | 2 | LMDG | 2.00 |
| *PROCATHEPSIN B (3PBH)* | | YLKRLCGT | 2 | LKRL | 0.22 |
| | cathepsin B **** | KRLCGTFL | 2 | RLCG | 0.14 |

# Metallo Protease

| **Protein Name (PDB ID)** | **Protease Name** | **Octapeptide cleavage sequence** | **rSASA** | **Tetrapeptide cleavage sequence** | **rSASA** |
|---|---|---|---|---|---|
| *STROMELYSIN (1CIZ)* | matrix metallopeptidase-13 | GIQSLYGP | 0.29 | IQSL | 0.23 |
| *APOLIPOPROTEIN E4 (1GS9)* | membrane-type matrix metallopeptidase-1 | MDETMKEL | 0.42 | DETM | 0.24 |
| *MMP9 (GELATINASE B) (1L6J)* | matrix metallopeptidase-9 | TLKAMRTP | 0.65 | LKAM | 0.30 |
| | | DLGRFQTF | 0.63 | LGRF | 0.39 |
| | matrix metallopeptidase-3 | RVAEMRGE | 2 | VAEM | 2.00 |

| | | | | | |
|---|---|---|---|---|---|
| | matrix metallopeptidase-7 | RVAEMRGE | 2 | VAEM | 2.00 |
| | matrix metallopeptidase-26 | TLKAMRTP | 0.65 | LKAM | 0.30 |
| *PLASMINOGEN ACTIVATOR INHIBITOR-1 (1LJ5)* | matrix metallopeptidase-3 | STAVIVSA | 0.26 | TAVI | 0.11 |
| *ALPHA-1-ANTITRYPSIN (1QLP)* | matrix metallopeptidase-7 | GAMFLEAI | 0.98 | AMFL | 0.72 |
| | matrix metallopeptidase-8 | GAMFLEAI | 0.98 | AMFL | 0.72 |
| | | EAIPMSIP | 0.92 | AIPM | 0.57 |
| | matrix metallopeptidase-9 | GAMFLEAI | 0.98 | AMFL | 0.72 |
| | | EAIPMSIP | 0.92 | AIPM | 0.57 |
| | matrix metallopeptidase-11 | AAGAMFLE | 1 | AGAM | 0.79 |
| | matrix metallopeptidase-7 | EAIPMSIP | 0.92 | AIPM | 0.57 |
| | matrix metallopeptidase-12 | RPFEVKDT | 0.61 | PFEV | 0.31 |
| | | GAMFLEAI | 0.98 | AMFL | 0.72 |
| | | EAIPMSIP | 0.92 | AIPM | 0.57 |
| | membrane-type matrix metallopeptidase-6 | GAMFLEAI | 0.98 | AMFL | 0.72 |
| | | EAIPMSIP | 0.92 | AIPM | 0.57 |
| | matrix metallopeptidase-26 | GAMFLEAI | 0.98 | AMFL | 0.72 |
| | | EAIPMSIP | 0.92 | AIPM | 0.57 |
| | matrix metallopeptidase-3 | EAIPMSIP | 0.92 | AIPM | 0.57 |

# Serine Protease

| **Protein Name (PDB ID)** | **Protease Name** | **Octapeptide cleavage sequence** | **rSASA** | **Tetrapeptide cleavage sequence** | **rSASA** |
|---|---|---|---|---|---|
| *COMPLEMENT C1S PROTEASE (1ELV)* | complement component activated C1r | EKQRIIGG | 2 | KQRI | 2.00 |
| *GELATINASE A (1GEN)* | elastase-2 | LGPVTPEI | 0.9 | GPVT | 0.88 |
| *THROMBIN (1JWT)* | coagulation factor Xa | IDGRIVEG | 1 | DGRI | 0.39 |
| | thrombin | IDGRIVEG | 1 | DGRI | 0.39 |
| *MMP9 (GELATINASE B) (1L6J)* | chymase | FQTFEGDL | 0.64 | QTFE | 0.60 |
| | trypsin-2 | DLGRFQTF | 0.63 | LGRF | 0.39 |
| *HTRA protease (1LCY)* | HtrA2 peptidase | VRLLSGDT | 0.44 | RLLS | 0.34 |
| | | PFALQNTI | 0.55 | FALQ | 0.30 |
| | | LQNTITSG | 0.54 | QNTI | 0.39 |
| *PLASMINOGEN ACTIVATOR INHIBITOR-1 (1LJ5)* | matriptase-3 | VSARMAPE | 0.75 | SARM | 0.32 |
| *KALLIKREIN 6 (1LO6)* | kallikrein-related peptidase 6 | LRQRESSQ | 0.88 | RQRE | 0.63 |
| *COMPLEMENT PROTEASE C1R (1MD8)* | complement component activated C1r | QRQRIIGG | 2 | RQRI | 2.00 |
| *SERUM ALBUMIN COMPLEXED (1N5U)* | chymase | RETYGEMA | 0.53 | ETYG | 0.45 |
| *PRO-CHYMASE (1NN6)* | chymase | PSQFNFVP | 0.68 | SQFN | 0.68 |
| *PLATELET RECEPTOR GLYCOPROTEIN IB-ALPHA (1P9A)* | cathepsin G | DTDLYDYY | 2 | TDLY | 2.00 |
| *FACTOR B (1Q0P)* | complement factor D | QQKRKIVL | 2 | QKRK | 2.00 |
| *ALPHA-1-ANTITRYPSIN (1QLP)* | mesotrypsin | AAQKTDTS | 2 | AQKT | 2.00 |

| | | | | | |
|---|---|---|---|---|---|
| *ALPHA1-ANTICHYMOTRYPSIN SERPIN (1QMN)* | elastase-2 | VKITLLSA | 2 | KITL | 2.00 |
| *CASPASE-3 (1QX3)* | granzyme B | IETDSGVD | 2 | ETDS | 2.00 |
| *ABETA-BOUND HUMAN ABAD (1SO8)* | HtrA2 peptidase | GNNCVFAP | 0.42 | NNCV | 0.32 |
| *VPS4B (1XWI)* | HtrA2 peptidase | ILPIKFPH | 0.56 | LPIK | 0.30 |
| *FIBRILLARIN (2IPX)* | granzyme B | VGPDGLVY | 0.39 | GPDG | 0.32 |

*- Localized in Lysosome(primary) , Extracellular,Golgi,ER

** - Localised in Endosome (primary), ER, Plasma Membrane, Cytoplast, Nucleus

*** - Localised in Endosome , Lysosome

**** - Localised in Lysosome (primary), Cytoplasm, Plasma Membrane, Mitochondria, Extracellular
