# Supplementary material for: A Sequence and Structure Based Method to Predict Putative Substrates, Functions and Regulatory Networks of Endo Proteases
Source: PLoS One. 2009 May 27;4(5):e5700. doi: 10.1371/journal.pone.0005700 (PMC2683571; doi:10.1371/journal.pone.0005700)
Supplement: Table S6 — Potential Substrates of Serine Proteases from the PDB and DisProt (0.38 MB PDF) [file pone.0005700.s007.pdf]

Table S6 - Potential Substrates of Serine Proteases from the PDB and DisProt

PDB

PDB ID	Enzyme name in BOLD/ Protein name	Cleavage Sequence	rSASA
	**chymase**		
2ODT	STRUCTURE OF HUMAN INOSITOL 1_3_4-TRISPHOSPHATE 5/6-KINASE	FPF	0.10
1A7S	ATOMIC RESOLUTION STRUCTURE OF HBP	FPF	0.12
1L6J	CRYSTAL STRUCTURE OF HUMAN MATRIX METALLOPROTEINASE MMP9 (GELATINASE B).	FPF	0.13
2A1X	HUMAN PHYTANOYL-COA 2-HYDROXYLASE IN COMPLEX WITH IRON AND 2-OXOGLUTARATE	FPF	0.15
2OCB	CRYSTAL STRUCTURE OF HUMAN RAB9B IN COMPLEX WITH A GTP ANALOGUE	FPF	0.15
1L6J	CRYSTAL STRUCTURE OF HUMAN MATRIX METALLOPROTEINASE MMP9 (GELATINASE B).	FPF	0.16
2AD1	HUMAN SULFOTRANSFERASE SULT1C2	FPF	0.17
1L6J	CRYSTAL STRUCTURE OF HUMAN MATRIX METALLOPROTEINASE MMP9 (GELATINASE B).	FPF	0.19
2V5O	STRUCTURE OF HUMAN IGF2R DOMAINS 11-14	FPF	0.19
1IMF	STRUCTURAL STUDIES OF METAL BINDING BY INOSITOL MONOPHOSPHATASE: EVIDENCE FOR TWO-METAL ION CATALYSIS	FPF	0.25
2VR2	HUMAN DIHYDROPYRIMIDINASE	FPF	0.25
1TQN	CRYSTAL STRUCTURE OF HUMAN MICROSOMAL P450 3A4	FPF	0.29
2OEW	STRUCTURE OF ALIX/AIP1 BRO1 DOMAIN	FPF	0.33
1XK5	CRYSTAL STRUCTURE OF THE M3G-CAP-BINDING DOMAIN OF SNURPORTIN1 IN COMPLEX WITH A M3GPPPG-CAP DINUCLEOTIDE	FPF	0.44
2OHF	CRYSTAL STRUCTURE OF HUMAN OLA1 IN COMPLEX WITH AMPPCP	FPF	2.00
	**coagulation factor IXa**		
1YCK	CRYSTAL STRUCTURE OF HUMAN PEPTIDOGLYCAN RECOGNITION PROTEIN (PGRP-S)	EGR	0.18
2O3H	CRYSTAL STRUCTURE OF THE HUMAN C65A APE	EGR	0.20
2FY2	STRUCTURES OF LIGAND BOUND HUMAN CHOLINE ACETYLTRANSFERASE PROVIDE INSIGHT INTO REGULATION OF ACETYLCHOLINE SYNTHESIS	EGR	0.22
1LI4	HUMAN S-ADENOSYLHOMOCYSTEINE HYDROLASE COMPLEXED WITH NEPLANOCIN	EGR	0.23
2HQQ	CRYSTAL STRUCTURE OF HUMAN KETOHEXOKINASE COMPLEXED TO DIFFERENT SUGAR MOLECULES	EGR	0.24
2GY5	TIE2 LIGAND-BINDING DOMAIN CRYSTAL STRUCTURE	EGR	0.25
1KT0	STRUCTURE OF THE LARGE FKBP-LIKE PROTEIN_ FKBP51_ INVOLVED IN STEROID RECEPTOR COMPLEXES	EGR	0.25
2B5M	CRYSTAL STRUCTURE OF DDB1	EGR	0.27
2V9R	FIRST AND SECOND IG DOMAINS FROM HUMAN ROBO1 (FORM 2)	EGR	0.30
2B3X	STRUCTURE OF AN ORTHORHOMBIC CRYSTAL FORM OF HUMAN CYTOSOLIC ACONITASE (IRP1)	EGR	0.31
1X04	CRYSTAL STRUCTURE OF ENDOPHILIN BAR DOMAIN (MUTANT)	EGR	0.32
1X03	CRYSTAL STRUCTURE OF ENDOPHILIN BAR DOMAIN	EGR	0.32
1KMQ	CRYSTAL STRUCTURE OF A CONSTITUTIVELY ACTIVATED RHOA MUTANT (Q63L)	EGR	0.34
2A91	CRYSTAL STRUCTURE OF ERBB2 DOMAINS 1-3	EGR	0.36
3CBQ	CRYSTAL STRUCTURE OF THE HUMAN REM2 GTPASE WITH BOUND GDP	EGR	0.37
1KAO	CRYSTAL STRUCTURE OF THE SMALL G PROTEIN RAP2A WITH GDP	EGR	0.37
2G3Y	CRYSTAL STRUCTURE OF THE HUMAN SMALL GTPASE GEM	EGR	0.38
1ZSX	CRYSTAL STRUCTURE OF HUMAN POTASSIUM CHANNEL KV BETA-SUBUNIT (KCNAB2)	EGR	0.40

1CZA	MUTANT MONOMER OF RECOMBINANT HUMAN HEXOKINASE TYPE I COMPLEXED WITH GLUCOSE_ GLUCOSE-6-PHOSPHATE_ AND ADP	EGR	0.40
2EFK	CRYSTAL STRUCTURE OF THE EFC DOMAIN OF CDC42-INTERACTING PROTEIN 4	EGR	0.41
1MX3	CRYSTAL STRUCTURE OF CTBP DEHYDROGENASE CORE HOLO FORM	EGR	0.42
1CZA	MUTANT MONOMER OF RECOMBINANT HUMAN HEXOKINASE TYPE I COMPLEXED WITH GLUCOSE_ GLUCOSE-6-PHOSPHATE_ AND ADP	EGR	0.44
2DQ7	CRYSTAL STRUCTURE OF FYN KINASE DOMAIN COMPLEXED WITH STAUROSPORINE	EGR	0.44
1V4S	CRYSTAL STRUCTURE OF HUMAN GLUCOKINASE	EGR	0.45
1IAT	CRYSTAL STRUCTURE OF HUMAN PHOSPHOGLUCOSE ISOMERASE/NEUROLEUKIN/AUTOCRINE MOTILITY FACTOR/MATURATION FACTOR	EGR	0.45
1CB0	STRUCTURE OF HUMAN 5'-DEOXY-5'-METHYLTHIOADENOSINE PHOSPHORYLASE AT 1.7 A RESOLUTION	EGR	0.48
1MQ4	CRYSTAL STRUCTURE OF AURORA-A PROTEIN KINASE	EGR	0.49
1JDN	CRYSTAL STRUCTURE OF HORMONE RECEPTOR	EGR	0.50
2NSM	CRYSTAL STRUCTURE OF THE HUMAN CARBOXYPEPTIDASE N (KININASE I) CATALYTIC DOMAIN	EGR	0.51
1HDR	THE CRYSTALLOGRAPHIC STRUCTURE OF A HUMAN DIHYDROPTERIDINE REDUCTASE NADH BINARY COMPLEX EXPRESSED IN ESCHERICHIA COLI BY A CDNA CONSTRUCTED FROM ITS RAT HOMOLOGUE	EGR	0.54
2I7Q	CRYSTAL STRUCTURE OF HUMAN CHOLINE KINASE A	EGR	0.54
2BH9	X-RAY STRUCTURE OF A DELETION VARIANT OF HUMAN GLUCOSE 6-PHOSPHATE DEHYDROGENASE COMPLEXED WITH STRUCTURAL AND COENZYME NADP	EGR	0.54
1L6J	CRYSTAL STRUCTURE OF HUMAN MATRIX METALLOPROTEINASE MMP9 (GELATINASE B).	EGR	0.55
1JTV	CRYSTAL STRUCTURE OF 17BETA-HYDROXYSTEROID DEHYDROGENASE TYPE 1 COMPLEXED WITH TESTOSTERONE	EGR	0.58
1L6J	CRYSTAL STRUCTURE OF HUMAN MATRIX METALLOPROTEINASE MMP9 (GELATINASE B).	EGR	0.63
2IVV	CRYSTAL STRUCTURE OF PHOSPHORYLATED RET TYROSINE KINASE DOMAIN COMPLEXED WITH THE INHIBITOR PP1	EGR	0.64
1P6F	STRUCTURE OF THE HUMAN NATURAL CYTOTOXICITY RECEPTOR NKP46	EGR	0.64
1N6A	STRUCTURE OF SET7/9	EGR	0.65
1ZSQ	CRYSTAL STRUCTURE OF MTMR2 IN COMPLEX WITH PHOSPHATIDYLINOSITOL 3-PHOSPHATE	EGR	0.67
1ELV	CRYSTAL STRUCTURE OF THE CATALYTIC DOMAIN OF HUMAN COMPLEMENT C1S PROTEASE	EGR	0.74
1GSM	A REASSESSMENT OF THE MADCAM-1 STRUCTURE AND ITS ROLE IN INTEGRIN RECOGNITION.	EGR	0.74
2PET	LUTHERAN GLYCOPROTEIN_ N-TERMINAL DOMAINS 1 AND 2.	EGR	0.75
1LN1	CRYSTAL STRUCTURE OF HUMAN PHOSPHATIDYLCHOLINE TRANSFER PROTEIN IN COMPLEX WITH DILINOLEOYLPHOSPHATIDYLCHOLINE	EGR	0.77
2OI0	CRYSTAL STRUCTURE ANALYSIS 0F THE TNF-A COVERTING ENZYME (TACE) IN COMPLEXED WITH ARYL-SULFONAMIDE	EGR	0.79
2IWR	GTPASE LIKE DOMAIN OF CENTAURIN GAMMA 1 (HUMAN)	EGR	0.83
2O8T	CRYSTAL STRUCTURE AND BINDING EPITOPES OF UROKINASE-TYPE PLASMINOGEN ACTIVATOR (C122A/N145Q) IN COMPLEX WITH INHIBITORS	EGR	2.00
2Q3H	THE CRYSTAL STRUCTURE OF RHOUA IN THE GDP-BOUND STATE.	EGR	2.00
2V9K	CRYSTAL STRUCTURE OF HUMAN PUS10_ A NOVEL PSEUDOURIDINE SYNTHASE.	EGR	2.00
	**coagulation factor XIa**		
1OI1	CRYSTAL STRUCTURE OF THE MBT DOMAINS OF HUMAN SCML2	EAR	0.18
2RKU	STRUCTURE OF PLK1 IN COMPLEX WITH BI2536	EAR	0.20

2DW4	CRYSTAL STRUCTURE OF HUMAN LSD1 AT 2.3 A RESOLUTION	EAR	0.22
2HGS	HUMAN GLUTATHIONE SYNTHETASE	EAR	0.23
2I53	CRYSTAL STRUCTURE OF CYCLIN K	EAR	0.23
2Z5Y	CRYSTAL STRUCTURE OF HUMAN MONOAMINE OXIDASE A (G110A) WITH HARMINE	EAR	0.24
2UW2	CRYSTAL STRUCTURE OF HUMAN RIBONUCLEOTIDE REDUCTASE SUBUNIT R2	EAR	0.25
1R9O	CRYSTAL STRUCTURE OF P4502C9 WITH FLURBIPROFEN BOUND	EAR	0.25
3BKB	CRYSTAL STRUCTURE OF HUMAN FELINE SARCOMA VIRAL ONCOGENE HOMOLOGUE (V-FES)	EAR	0.28
2V7O	CRYSTAL STRUCTURE OF HUMAN CALCIUM-CALMODULIN-DEPENDENT PROTEIN KINASE II GAMMA	EAR	0.29
2H6D	PROTEIN KINASE DOMAIN OF THE HUMAN 5'-AMP-ACTIVATED PROTEIN KINASE CATALYTIC SUBUNIT ALPHA-2 (AMPK ALPHA-2 CHAIN)	EAR	0.29
2I4I	CRYSTAL STRUCTURE OF HUMAN DEAD-BOX RNA HELICASE DDX3X	EAR	0.29
2FY2	STRUCTURES OF LIGAND BOUND HUMAN CHOLINE ACETYLTRANSFERASE PROVIDE INSIGHT INTO REGULATION OF ACETYLCHOLINE SYNTHESIS	EAR	0.30
1P0I	CRYSTAL STRUCTURE OF HUMAN BUTYRYL CHOLINESTERASE	EAR	0.31
1E0S	SMALL G PROTEIN ARF6-GDP	EAR	0.31
1BY7	HUMAN PLASMINOGEN ACTIVATOR INHIBITOR-2. LOOP (66-98) DELETION MUTANT	EAR	0.31
2OC3	CRYSTAL STRUCTURE OF THE CATALYTIC DOMAIN OF HUMAN PROTEIN TYROSINE PHOSPHATASE NON-RECEPTOR TYPE 18	EAR	0.31
1K1B	CRYSTAL STRUCTURE OF THE ANKYRIN REPEAT DOMAIN OF BCL-3: A UNIQUE MEMBER OF THE IKAPPAB PROTEIN FAMILY	EAR	0.33
2QZ4	HUMAN PARAPLEGIN_ AAA DOMAIN IN COMPLEX WITH ADP	EAR	0.33
1X9D	CRYSTAL STRUCTURE OF HUMAN CLASS I ALPHA-1_2-MANNOSIDASE IN COMPLEX WITH THIO-DISACCHARIDE SUBSTRATE ANALOGUE	EAR	0.33
1IAT	CRYSTAL STRUCTURE OF HUMAN PHOSPHOGLUCOSE ISOMERASE/NEUROLEUKIN/AUTOCRINE MOTILITY FACTOR/MATURATION FACTOR	EAR	0.35
1E2S	CRYSTAL STRUCTURE OF AN ARYLSULFATASE A MUTANT C69A	EAR	0.35
1IAP	CRYSTAL STRUCTURE OF P115RHOGEF RGRGS DOMAIN	EAR	0.36
2I7V	STRUCTURE OF HUMAN CPSF-73	EAR	0.36
2F9L	3D STRUCTURE OF INACTIVE HUMAN RAB11B GTPASE	EAR	0.37
2NZ2	CRYSTAL STRUCTURE OF HUMAN ARGININOSUCCINATE SYNTHASE IN COMPLEX WITH ASPARTATE AND CITRULLINE	EAR	0.37
1WB0	SPECIFICITY AND AFFINITY OF NATURAL PRODUCT CYCLOPENTAPEPTIDE INHIBITOR ARGIFIN AGAINST HUMAN CHITINAS	EAR	0.37
2OIL	CRYSTAL STRUCTURE OF HUMAN RAB25 IN COMPLEX WITH GDP	EAR	0.38
1NUF	ROLE OF CALCIUM IONS IN THE ACTIVATION AND ACTIVITY OF THE TRANSGLUTAMINASE 3 ENZYME	EAR	0.38
2QQJ	CRYSTAL STRUCTURE OF THE B1B2 DOMAINS FROM HUMAN NEUROPILIN 2	EAR	0.43
1SK4	CRYSTAL STRUCTURE OF THE C-TERMINAL PEPTIDOGLYCAN-BINDING DOMAIN OF HUMAN PEPTIDOGLYCAN RECOGNITION PROTEIN IALPHA	EAR	0.44
1XA6	CRYSTAL STRUCTURE OF THE HUMAN BETA2-CHIMAERIN	EAR	0.44
2QLU	CRYSTAL STRUCTURE OF ACTIVIN RECEPTOR TYPE II KINASE DOMAIN FROM HUMAN	EAR	0.44
1ZIV	CATALYTIC DOMAIN OF HUMAN CALPAIN-9	EAR	0.45
1HDR	THE CRYSTALLOGRAPHIC STRUCTURE OF A HUMAN DIHYDROPTERIDINE REDUCTASE NADH BINARY COMPLEX EXPRESSED IN ESCHERICHIA COLI BY A CDNA CONSTRUCTED FROM ITS RAT HOMOLOGUE	EAR	0.48
2PNY	STRUCTURE OF HUMAN ISOPENTENYL-DIPHOSPHATE DELTA-ISOMERASE	EAR	0.50
2Q5I	CRYSTAL STRUCTURE OF APO S581L GLYCYL-TRNA SYNTHETASE MUTAN	EAR	0.50
1QCY	THE CRYSTAL STRUCTURE OF THE I-DOMAIN OF HUMAN INTEGRIN ALPHA1BETA1	EAR	0.52
2EW1	CRYSTAL STRUCTURE OF RAB30 IN COMPLEX WITH A GTP ANALOGUE	EAR	0.75

1IAT	CRYSTAL STRUCTURE OF HUMAN PHOSPHOGLUCOSE ISOMERASE/NEUROLEUKIN/AUTOCRINE MOTILITY FACTOR/MATURATION FACTOR	EAR	0.79
1R55	CRYSTAL STRUCTURE OF THE CATALYTIC DOMAIN OF HUMAN ADAM 33	EAR	2.00
3C8X	CRYSTAL STRUCTURE OF THE LIGAND BINDING DOMAIN OF HUMAN EPHRIN A2 (EPHA2) RECEPTOR PROTEIN KINASE	EAR	2.00
	**coagulation factor XIIa**		
3CHO	CRYSTAL STRUCTURE OF LEUKOTRIENE A4 HYDROLASE IN COMPLEX WITH 2-AMINO-N-[4-(PHENYLMETHOXY)PHENYL]-ACETAMIDE	QGR	0.19
2B5M	CRYSTAL STRUCTURE OF DDB1	QGR	0.19
1WMA	CRYSTAL STRUCTURE OF HUMAN CBR1 IN COMPLEX WITH HYDROXY-PP	QGR	0.20
1T7V	ZN-ALPHA-2-GLYCOPROTEIN; BACULO-ZAG PEG 200	QGR	0.22
2D7I	CRSYTAL STRUCTURE OF PP-GALNAC-T10 WITH UDP_ GALNAC AND MN2	QGR	0.26
3BGS	STRUCTURE OF HUMAN PURINE NUCLEOSIDE PHOSPHORYLASE WITH L-DADME-IMMH AND PHOSPHATE	QGR	0.27
2GJK	STRUCTURAL AND FUNCTIONAL INSIGHTS INTO THE HUMAN UPF1 HELICASE CORE	QGR	0.30
1F6W	STRUCTURE OF THE CATALYTIC DOMAIN OF HUMAN BILE SALT ACTIVATED LIPASE	QGR	0.31
2PET	LUTHERAN GLYCOPROTEIN_ N-TERMINAL DOMAINS 1 AND 2.	QGR	0.33
2OHF	CRYSTAL STRUCTURE OF HUMAN OLA1 IN COMPLEX WITH AMPPCP	QGR	0.34
2ZG1	CRYSTAL STRUCTURE OF TWO N-TERMINAL DOMAINS OF SIGLEC-5 IN COMPLEX WITH 6'-SIALYLLACTOSE	QGR	0.34
1X8B	STRUCTURE OF HUMAN WEE1A KINASE: KINASE DOMAIN COMPLEXED WITH INHIBITOR PD0407824	QGR	0.35
2I7Q	CRYSTAL STRUCTURE OF HUMAN CHOLINE KINASE A	QGR	0.35
2ESB	CRYSTAL STRUCTURE OF HUMAN DUSP18	QGR	0.36
2AXN	CRYSTAL STRUCTURE OF THE HUMAN INDUCIBLE FORM 6-PHOSPHOFRUCTO-2-KINASE/FRUCTOSE-2_6-BISPHOSPHATASE	QGR	0.40
1WB0	SPECIFICITY AND AFFINITY OF NATURAL PRODUCT CYCLOPENTAPEPTIDE INHIBITOR ARGIFIN AGAINST HUMAN CHITINAS	QGR	0.40
2QQ5	CRYSTAL STRUCTURE OF HUMAN SDR FAMILY MEMBER 1	QGR	0.41
1JTV	CRYSTAL STRUCTURE OF 17BETA-HYDROXYSTEROID DEHYDROGENASE TYPE 1 COMPLEXED WITH TESTOSTERONE	QGR	0.41
2B9E	HUMAN NSUN5 PROTEIN	QGR	0.43
1B0F	CRYSTAL STRUCTURE OF HUMAN NEUTROPHIL ELASTASE WITH MDL 101 146	QGR	0.43
2JDF	HUMAN GAMMA-B CRYSTALLIN	QGR	0.43
3BER	HUMAN DEAD-BOX RNA-HELICASE DDX47_ CONSERVED DOMAIN I IN COMPLEX WITH AMP	QGR	0.45
1BX4	STRUCTURE OF HUMAN ADENOSINE KINASE AT 1.50 ANGSTROMS	QGR	0.46
1Z32	STRUCTURE-FUNCTION RELATIONSHIPS IN HUMAN SALIVARY ALPHA-AMYLASE: ROLE OF AROMATIC RESIDUES	QGR	0.47
1ZGK	1.35 ANGSTROM STRUCTURE OF THE KELCH DOMAIN OF KEAP1	QGR	0.47
2B5M	CRYSTAL STRUCTURE OF DDB1	QGR	0.49
1D3G	HUMAN DIHYDROOROTATE DEHYDROGENASE COMPLEXED WITH BREQUINAR ANALOG	QGR	0.50
3CTZ	STRUCTURE OF HUMAN CYTOSOLIC X-PROLYL AMINOPEPTIDASE	QGR	0.52
1GWZ	CRYSTAL STRUCTURE OF THE CATALYTIC DOMAIN OF THE PROTEIN TYROSINE PHOSPHATASE SHP-1	QGR	0.54
1J72	CRYSTAL STRUCTURE OF MUTANT MACROPHAGE CAPPING PROTEIN (CAP G) WITH ACTIN-SEVERING ACTIVITY IN THE CA2+-FREE FORM	QGR	0.55
2HQQ	CRYSTAL STRUCTURE OF HUMAN KETOHEXOKINASE COMPLEXED TO DIFFERENT SUGAR MOLECULES	QGR	0.57
2IVV	CRYSTAL STRUCTURE OF PHOSPHORYLATED RET TYROSINE KINASE DOMAIN COMPLEXED WITH THE INHIBITOR PP1	QGR	0.58
1A7S	ATOMIC RESOLUTION STRUCTURE OF HBP	QGR	0.59

1D7P	CRYSTAL STRUCTURE OF THE C2 DOMAIN OF HUMAN FACTOR VIII AT 1.5 A RESOLUTION AT 1.5 A	QGR	0.63
2A8B	CRYSTAL STRUCTURE OF THE CATALYTIC DOMAIN OF HUMAN TYROSINE PHOSPHATASE RECEPTOR_ TYPE R	QGR	0.64
1R0P	CRYSTAL STRUCTURE OF THE TYROSINE KINASE DOMAIN OF THE HEPATOCYTE GROWTH FACTOR RECEPTOR C-MET IN COMPLEX WITH THE MICROBIAL ALKALOID K-252A	QGR	0.65
1CZT	CRYSTAL STRUCTURE OF THE C2 DOMAIN OF HUMAN COAGULATION FACTOR V	QGR	0.68
2O8T	CRYSTAL STRUCTURE AND BINDING EPITOPES OF UROKINASE-TYPE PLASMINOGEN ACTIVATOR (C122A/N145Q) IN COMPLEX WITH INHIBITORS	QGR	0.75
2NZ6	CRYSTAL STRUCTURE OF THE PTPRJ INACTIVATING MUTANT C1239S	QGR	2.00
	**complement component C2a**		
2NSM	CRYSTAL STRUCTURE OF THE HUMAN CARBOXYPEPTIDASE N (KININASE I) CATALYTIC DOMAIN	LGR	0.10
1W6K	STRUCTURE OF HUMAN OSC IN COMPLEX WITH LANOSTEROL	LGR	0.10
2O8T	CRYSTAL STRUCTURE AND BINDING EPITOPES OF UROKINASE-TYPE PLASMINOGEN ACTIVATOR (C122A/N145Q) IN COMPLEX WITH INHIBITORS	LGR	0.11
1SPJ	STRUCTURE OF MATURE HUMAN TISSUE KALLIKREIN (HUMAN KALLIKREIN 1 OR KLK1) AT 1.70 ANGSTROM RESOLUTION WITH VACANT ACTIVE SITE	LGR	0.12
2DW5	CRYSTAL STRUCTURE OF HUMAN PEPTIDYLARGININE DEIMINASE 4 IN COMPLEX WITH N-ALPHA-BENZOYL-N5-(2-FLUORO-1-IMINOETHYL)-L-ORNITHINE AMIDE	LGR	0.12
1OHC	STRUCTURE OF THE PROLINE DIRECTED PHOSPHATASE CDC14	LGR	0.13
2PCX	CRYSTAL STRUCTURE OF P53DBD(R282Q) AT 1.54-ANGSTROM RESOLUTION	LGR	0.15
1Q33	CRYSTAL STRUCTURE OF HUMAN ADP-RIBOSE PYROPHOSPHATASE NUDT9	LGR	0.16
1KHB	PEPCK COMPLEX WITH NONHYDROLYZABLE GTP ANALOG_ NATIVE DATA	LGR	0.16
1UOU	CRYSTAL STRUCTURE OF HUMAN THYMIDINE PHOSPHORYLASE IN COMPLEX WITH A SMALL MOLECULE INHIBITOR	LGR	0.16
1Z8D	CRYSTAL STRUCTURE OF HUMAN MUSCLE GLYCOGEN PHOSPHORYLASE A WITH AMP AND GLUCOSE	LGR	0.17
1NM8	STRUCTURE OF HUMAN CARNITINE ACETYLTRANSFERASE: MOLECULAR BASIS FOR FATTY ACYL TRANSFER	LGR	0.18
1M6I	CRYSTAL STRUCTURE OF APOPTOSIS INDUCING FACTOR (AIF)	LGR	0.19
2Q5I	CRYSTAL STRUCTURE OF APO S581L GLYCYL-TRNA SYNTHETASE MUTAN	LGR	0.19
2CY7	THE CRYSTAL STRUCTURE OF HUMAN ATG4B	LGR	0.19
1UOU	CRYSTAL STRUCTURE OF HUMAN THYMIDINE PHOSPHORYLASE IN COMPLEX WITH A SMALL MOLECULE INHIBITOR	LGR	0.19
1OZN	1.5A CRYSTAL STRUCTURE OF THE NOGO RECEPTOR LIGAND BINDING DOMAIN REVEALS A CONVERGENT RECOGNITION SCAFFOLD MEDIATING INHIBITION OF MYELINATION	LGR	0.19
1FA9	HUMAN LIVER GLYCOGEN PHOSPHORYLASE A COMPLEXED WITH AMP	LGR	0.19
1LF7	CRYSTAL STRUCTURE OF HUMAN COMPLEMENT PROTEIN C8GAMMA AT 1.2 A RESOLUTION	LGR	0.20
1RYO	HUMAN SERUM TRANSFERRIN_ N-LOBE BOUND WITH OXALATE	LGR	0.20
2PE4	STRUCTURE OF HUMAN HYALURONIDASE 1_ A HYALURONAN HYDROLYZING ENZYME INVOLVED IN TUMOR GROWTH AND ANGIOGENESIS	LGR	0.21
2I7V	STRUCTURE OF HUMAN CPSF-73	LGR	0.22
1OZN	1.5A CRYSTAL STRUCTURE OF THE NOGO RECEPTOR LIGAND BINDING DOMAIN REVEALS A CONVERGENT RECOGNITION SCAFFOLD MEDIATING INHIBITION OF MYELINATION	LGR	0.22

2V24	STRUCTURE OF THE HUMAN SPRY DOMAIN-CONTAINING SOCS BOX PROTEIN SSB-4	LGR	0.22
1SIQ	THE CRYSTAL STRUCTURE AND MECHANISM OF HUMAN GLUTARYL-COA DEHYDROGENASE	LGR	0.23
2HQ6	STRUCTURE OF THE CYCLOPHILIN_CECYP16-LIKE DOMAIN OF THE SEROLOGICALLY DEFINED COLON CANCER ANTIGEN 10 FROM HOMO SAPIENS	LGR	0.23
2HZ6	THE CRYSTAL STRUCTURE OF HUMAN IRE1-ALPHA LUMINAL DOMAIN	LGR	0.24
1XWI	CRYSTAL STRUCTURE OF VPS4B	LGR	0.24
1GS9	APOLIPOPROTEIN E4_ 22K DOMAIN	LGR	0.25
2CL3	CRYSTAL STRUCTURE OF HUMAN CLEAVAGE AND POLYADENYLATION SPECIFICITY FACTOR 5 (CPSF5)	LGR	0.26
2ILR	CRYSTAL STRUCTURE OF HUMAN FANCONI ANEMIA PROTEIN E C-TERMINAL DOMAIN	LGR	0.26
1LCY	CRYSTAL STRUCTURE OF THE MITOCHONDRIAL SERINE PROTEASE HTRA	LGR	0.27
2O36	CRYSTAL STRUCTURE OF ENGINEERED THIMET OLIGOPEPTIDASE WITH NEUROLYSIN SPECIFICITY IN NEUROTENSIN CLEAVAGE SITE	LGR	0.27
1W8M	ENZYMATIC AND STRUCTURAL CHARACTERISATION OF NON PEPTIDE LIGAND CYCLOPHILIN COMPLEXES	LGR	0.27
1SQW	CRYSTAL STRUCTURE OF KD93_ A NOVEL PROTEIN EXPRESSED IN THE HUMAN PRO	LGR	0.27
1MF7	INTEGRIN ALPHA M I DOMAIN	LGR	0.28
1SIQ	THE CRYSTAL STRUCTURE AND MECHANISM OF HUMAN GLUTARYL-COA DEHYDROGENASE	LGR	0.28
2BIT	CRYSTAL STRUCTURE OF HUMAN CYCLOPHILIN D AT 1.7 A RESOLUTIO	LGR	0.28
1ORE	HUMAN ADENINE PHOSPHORIBOSYLTRANSFERASE	LGR	0.28
1B0F	CRYSTAL STRUCTURE OF HUMAN NEUTROPHIL ELASTASE WITH MDL 101 146	LGR	0.28
2OPW	CRYSTAL STRUCTURE OF HUMAN PHYTANOYL-COA DIOXYGENASE PHYHD1 (APO)	LGR	0.28
1TA0	THREE-DIMENSIONAL STRUCTURE OF A RNA-POLYMERASE II BINDING PROTEIN WITH ASSOCIATED LIGAND.	LGR	0.28
1ZD3	HUMAN SOLUBLE EPOXIDE HYDROLASE 4-(3-CYCLOHEXYLURIEDO)-BUTYRIC ACID COMPLEX	LGR	0.29
1H30	C-TERMINAL LG DOMAIN PAIR OF HUMAN GAS6	LGR	0.29
1S35	CRYSTAL STRUCTURE OF REPEATS 8 AND 9 OF HUMAN ERYTHROID SPECTRIN	LGR	0.31
1MX3	CRYSTAL STRUCTURE OF CTBP DEHYDROGENASE CORE HOLO FORM	LGR	0.31
2CY7	THE CRYSTAL STRUCTURE OF HUMAN ATG4B	LGR	0.32
1W6K	STRUCTURE OF HUMAN OSC IN COMPLEX WITH LANOSTEROL	LGR	0.33
2GRY	CRYSTAL STRUCTURE OF THE HUMAN KIF2 MOTOR DOMAIN IN COMPLEX WITH ADP	LGR	0.34
1LI4	HUMAN S-ADENOSYLHOMOCYSTEINE HYDROLASE COMPLEXED WITH NEPLANOCIN	LGR	0.34
1OSH	A CHEMICAL_ GENETIC_ AND STRUCTURAL ANALYSIS OF THE NUCLEAR BILE ACID RECEPTOR FXR	LGR	0.35
2OYC	CRYSTAL STRUCTURE OF HUMAN PYRIDOXAL PHOSPHATE PHOSPHATASE	LGR	0.36
1ZC0	CRYSTAL STRUCTURE OF HUMAN HEMATOPOIETIC TYROSINE PHOSPHATASE (HEPTP) CATALYTIC DOMAIN	LGR	0.36
1DG6	CRYSTAL STRUCTURE OF APO2L/TRAIL	LGR	0.37
2NZ2	CRYSTAL STRUCTURE OF HUMAN ARGININOSUCCINATE SYNTHASE IN COMPLEX WITH ASPARTATE AND CITRULLINE	LGR	0.37
2OZU	CRYSTAL STRUCTURE OF HUMAN MYST HISTONE ACETYLTRANSFERASE 3 IN COMPLEX WITH ACETYLCOENZYME A	LGR	0.37
2IUW	CRYSTAL STRUCTURE OF HUMAN ABH3 IN COMPLEX WITH IRON ION AND 2-OXOGLUTARATE	LGR	0.37
2FVV	HUMAN DIPHOSPHOINOSITOL POLYPHOSPHATE PHOSPHOHYDROLASE 1	LGR	0.40

2O36	CRYSTAL STRUCTURE OF ENGINEERED THIMET OLIGOPEPTIDASE WITH NEUROLYSIN SPECIFICITY IN NEUROTENSIN CLEAVAGE SITE	LGR	0.40
1Q20	CRYSTAL STRUCTURE OF HUMAN CHOLESTEROL SULFOTRANSFERASE (SULT2B1B) IN THE PRESENCE OF PAP AND PREGNENOLONE	LGR	0.41
1EVS	CRYSTAL STRUCTURE OF HUMAN ONCOSTATIN M	LGR	0.41
1M6I	CRYSTAL STRUCTURE OF APOPTOSIS INDUCING FACTOR (AIF)	LGR	0.42
1UPV	CRYSTAL STRUCTURE OF THE HUMAN LIVER X RECEPTOR BETA LIGAND BINDING DOMAIN IN COMPLEX WITH A SYNTHETIC AGONIST	LGR	0.42
1L6J	CRYSTAL STRUCTURE OF HUMAN MATRIX METALLOPROTEINASE MMP9 (GELATINASE B).	LGR	0.43
1P0I	CRYSTAL STRUCTURE OF HUMAN BUTYRYL CHOLINESTERASE	LGR	0.43
1OHC	STRUCTURE OF THE PROLINE DIRECTED PHOSPHATASE CDC14	LGR	0.43
3BQC	HIGH PH-VALUE CRYSTAL STRUCTURE OF EMODIN IN COMPLEX WITH THE CATALYTIC SUBUNIT OF PROTEIN KINASE CK2	LGR	0.43
2OU2	ACETYLTRANSFERASE DOMAIN OF HUMAN HIV-1 TAT INTERACTING PROTEIN_ 60KDA_ ISOFORM 3	LGR	0.43
1MP8	CRYSTAL STRUCTURE OF FOCAL ADHESION KINASE (FAK)	LGR	0.43
1YWN	VEGFR2 IN COMPLEX WITH A NOVEL 4-AMINO-FURO[2_3-D]PYRIMIDIN	LGR	0.44
3BQC	HIGH PH-VALUE CRYSTAL STRUCTURE OF EMODIN IN COMPLEX WITH THE CATALYTIC SUBUNIT OF PROTEIN KINASE CK2	LGR	0.46
2V9K	CRYSTAL STRUCTURE OF HUMAN PUS10_ A NOVEL PSEUDOURIDINE SYNTHASE.	LGR	0.47
1Y6B	CRYSTAL STRUCTURE OF VEGFR2 IN COMPLEX WITH A 2-ANILINO-5- ARYL-OXAZOLE INHIBITOR	LGR	0.48
2P39	CRYSTAL STRUCTURE OF HUMAN FGF23	LGR	0.48
2DE0	CRYSTAL STRUCTURE OF HUMAN ALPHA 1_6-FUCOSYLTRANSFERASE_ FUT8	LGR	0.48
2A2C	X-RAY STRUCTURE OF HUMAN N-ACETYL GALACTOSAMINE KINASE COMPLEXED WITH MG-ADP AND N-ACETYL GALACTOSAMINE 1-PHOSPHATE	LGR	0.50
2UUI	CRYSTAL STRUCTURE OF HUMAN LEUKOTRIENE C4 SYNTHASE	LGR	0.50
1ZD3	HUMAN SOLUBLE EPOXIDE HYDROLASE 4-(3-CYCLOHEXYLURIEDO)-BUTYRIC ACID COMPLEX	LGR	0.53
1LCY	CRYSTAL STRUCTURE OF THE MITOCHONDRIAL SERINE PROTEASE HTRA	LGR	0.63
1JDW	CRYSTAL STRUCTURE AND MECHANISM OF L-ARGININE: GLYCINE AMIDINOTRANSFERASE: A MITOCHONDRIAL ENZYME INVOLVED IN CREATINE BIOSYNTHESIS	LGR	2.00
2AEX	THE 1.58A CRYSTAL STRUCTURE OF HUMAN COPROPORPHYRINOGEN OXIDASE REVEALS THE STRUCTURAL BASIS OF HEREDITARY COPROPORPHYRIA	LGR	2.00
2QOL	HUMAN EPHA3 KINASE AND JUXTAMEMBRANE REGION_ Y596:Y602:S768G TRIPLE MUTANT	LGR	2.00
	**DESC1 peptidase**		
2QQJ	CRYSTAL STRUCTURE OF THE B1B2 DOMAINS FROM HUMAN NEUROPILIN 2	FVR	0.12
2NR8	CRYSTAL STRUCTURE OF THE HUMAN KIF9 MOTOR DOMAIN IN COMPLEX WITH ADP	FVR	0.12
2B9E	HUMAN NSUN5 PROTEIN	FVR	0.12
2QQI	CRYSTAL STRUCTURE OF THE B1B2 DOMAINS FROM HUMAN NEUROPILIN 1	FVR	0.12
1SI5	PROTEASE-LIKE DOMAIN FROM 2-CHAIN HEPATOCYTE GROWTH FACTOR	FVR	0.12
2EC8	CRYSTAL STRUCTURE OF THE EXCTRACELLULAR DOMAIN OF THE RECEPTOR TYROSINE KINASE_ KIT	FVR	0.13
2QQJ	CRYSTAL STRUCTURE OF THE B1B2 DOMAINS FROM HUMAN NEUROPILIN 2	FVR	0.13
1A6Q	CRYSTAL STRUCTURE OF THE PROTEIN SERINE/THREONINE PHOSPHATASE 2C AT 2 A RESOLUTION	FVR	0.16
1MD8	MONOMERIC STRUCTURE OF THE ACTIVE CATALYTIC DOMAIN OF COMPLEMENT PROTEASE C1R	FVR	0.17

1NUF	ROLE OF CALCIUM IONS IN THE ACTIVATION AND ACTIVITY OF THE TRANSGLUTAMINASE 3 ENZYME	FVR	0.17
2BH9	X-RAY STRUCTURE OF A DELETION VARIANT OF HUMAN GLUCOSE 6-PHOSPHATE DEHYDROGENASE COMPLEXED WITH STRUCTURAL AND COENZYME NADP	FVR	0.17
2FY2	STRUCTURES OF LIGAND BOUND HUMAN CHOLINE ACETYLTRANSFERASE PROVIDE INSIGHT INTO REGULATION OF ACETYLCHOLINE SYNTHESIS	FVR	0.18
2PZ1	CRYSTAL STRUCTURE OF AUTO-INHIBITED ASEF	FVR	0.18
1YCK	CRYSTAL STRUCTURE OF HUMAN PEPTIDOGLYCAN RECOGNITION PROTEIN (PGRP-S)	EGR	0.18
2F1W	CRYSTAL STRUCTURE OF THE TRAF-LIKE DOMAIN OF HAUSP/USP7	FVR	0.18
2A4D	STRUCTURE OF THE HUMAN UBIQUITIN-CONJUGATING ENZYME E2 VARIANT 1 (UEV-1)	FVR	0.19
2O3H	CRYSTAL STRUCTURE OF THE HUMAN C65A APE	EGR	0.20
2HC1	ENGINEERED CATALYTIC DOMAIN OF PROTEIN TYROSINE PHOSPHATASE HPTPBETA.	FVR	0.21
1CZA	MUTANT MONOMER OF RECOMBINANT HUMAN HEXOKINASE TYPE I COMPLEXED WITH GLUCOSE_ GLUCOSE-6-PHOSPHATE_ AND ADP	FVR	0.22
2FY2	STRUCTURES OF LIGAND BOUND HUMAN CHOLINE ACETYLTRANSFERASE PROVIDE INSIGHT INTO REGULATION OF ACETYLCHOLINE SYNTHESIS	EGR	0.22
1XJD	CRYSTAL STRUCTURE OF PKC-THETA COMPLEXED WITH STAUROSPORINE AT 2A RESOLUTION	FVR	0.22
1HFC	1.56 ANGSTROM STRUCTURE OF MATURE TRUNCATED HUMAN FIBROBLAST COLLAGENASE	FVR	0.22
3C5H	CRYSTAL STRUCTURE OF THE RAS HOMOLOG DOMAIN OF HUMAN GRLF1 (P190RHOGAP)	FVR	0.22
2BKA	CC3(TIP30)CRYSTAL STRUCURE	FVR	0.22
2PBN	CRYSTAL STRUCTURE OF THE HUMAN TYROSINE RECEPTOR PHOSPHATE GAMMA	FVR	0.22
1N3Y	CRYSTAL STRUCTURE OF THE ALPHA-X BETA2 INTEGRIN I DOMAIN	FVR	0.22
2II0	CRYSTAL STRUCTURE OF CATALYTIC DOMAIN OF SON OF SEVENLESS (REM-CDC25) IN THE ABSENCE OF RAS	FVR	0.23
1LI4	HUMAN S-ADENOSYLHOMOCYSTEINE HYDROLASE COMPLEXED WITH NEPLANOCIN	EGR	0.23
2NZ2	CRYSTAL STRUCTURE OF HUMAN ARGININOSUCCINATE SYNTHASE IN COMPLEX WITH ASPARTATE AND CITRULLINE	FVR	0.24
1CZA	MUTANT MONOMER OF RECOMBINANT HUMAN HEXOKINASE TYPE I COMPLEXED WITH GLUCOSE_ GLUCOSE-6-PHOSPHATE_ AND ADP	FVR	0.24
2QTZ	CRYSTAL STRUCTURE OF THE NADP+-BOUND FAD-CONTAINING FNR- LIKE MODULE OF HUMAN METHIONINE SYNTHASE REDUCTASE	FVR	0.24
2HQQ	CRYSTAL STRUCTURE OF HUMAN KETOHEXOKINASE COMPLEXED TO DIFFERENT SUGAR MOLECULES	EGR	0.24
2GY5	TIE2 LIGAND-BINDING DOMAIN CRYSTAL STRUCTURE	EGR	0.25
1KT0	STRUCTURE OF THE LARGE FKBP-LIKE PROTEIN_ FKBP51_ INVOLVED IN STEROID RECEPTOR COMPLEXES	EGR	0.25
2B5M	CRYSTAL STRUCTURE OF DDB1	EGR	0.27
1B0F	CRYSTAL STRUCTURE OF HUMAN NEUTROPHIL ELASTASE WITH MDL 101 146	FVR	0.29
1IJB	THE VON WILLEBRAND FACTOR MUTANT (I546V) A1 DOMAIN	FVR	0.30
2V9R	FIRST AND SECOND IG DOMAINS FROM HUMAN ROBO1 (FORM 2)	EGR	0.30
2B3X	STRUCTURE OF AN ORTHORHOMBIC CRYSTAL FORM OF HUMAN CYTOSOLIC ACONITASE (IRP1)	EGR	0.31
2AA2	MINERALOCORTICOID RECEPTOR WITH BOUND ALDOSTERONE	FVR	0.31
1X04	CRYSTAL STRUCTURE OF ENDOPHILIN BAR DOMAIN (MUTANT)	EGR	0.32
1X03	CRYSTAL STRUCTURE OF ENDOPHILIN BAR DOMAIN	EGR	0.32
1KMQ	CRYSTAL STRUCTURE OF A CONSTITUTIVELY ACTIVATED RHOA MUTANT (Q63L)	EGR	0.34
2A91	CRYSTAL STRUCTURE OF ERBB2 DOMAINS 1-3	EGR	0.36

3CBQ	CRYSTAL STRUCTURE OF THE HUMAN REM2 GTPASE WITH BOUND GDP	EGR	0.37
1KAO	CRYSTAL STRUCTURE OF THE SMALL G PROTEIN RAP2A WITH GDP	EGR	0.37
2G3Y	CRYSTAL STRUCTURE OF THE HUMAN SMALL GTPASE GEM	EGR	0.38
1ZSX	CRYSTAL STRUCTURE OF HUMAN POTASSIUM CHANNEL KV BETA-SUBUNIT (KCNAB2)	EGR	0.40
1CZA	MUTANT MONOMER OF RECOMBINANT HUMAN HEXOKINASE TYPE I COMPLEXED WITH GLUCOSE_ GLUCOSE-6-PHOSPHATE_ AND ADP	EGR	0.40
2EFK	CRYSTAL STRUCTURE OF THE EFC DOMAIN OF CDC42-INTERACTING PROTEIN 4	EGR	0.41
1MX3	CRYSTAL STRUCTURE OF CTBP DEHYDROGENASE CORE HOLO FORM	EGR	0.42
1CZA	MUTANT MONOMER OF RECOMBINANT HUMAN HEXOKINASE TYPE I COMPLEXED WITH GLUCOSE_ GLUCOSE-6-PHOSPHATE_ AND ADP	EGR	0.44
2DQ7	CRYSTAL STRUCTURE OF FYN KINASE DOMAIN COMPLEXED WITH STAUROSPORINE	EGR	0.44
1V4S	CRYSTAL STRUCTURE OF HUMAN GLUCOKINASE	EGR	0.45
1IAT	CRYSTAL STRUCTURE OF HUMAN PHOSPHOGLUCOSE ISOMERASE/NEUROLEUKIN/AUTOCRINE MOTILITY FACTOR/MATURATION FACTOR	EGR	0.45
1CB0	STRUCTURE OF HUMAN 5'-DEOXY-5'-METHYLTHIOADENOSINE PHOSPHORYLASE AT 1.7 A RESOLUTION	EGR	0.48
1MQ4	CRYSTAL STRUCTURE OF AURORA-A PROTEIN KINASE	EGR	0.49
1JDN	CRYSTAL STRUCTURE OF HORMONE RECEPTOR	EGR	0.50
2NSM	CRYSTAL STRUCTURE OF THE HUMAN CARBOXYPEPTIDASE N (KININASE I) CATALYTIC DOMAIN	EGR	0.51
1HDR	THE CRYSTALLOGRAPHIC STRUCTURE OF A HUMAN DIHYDROPTERIDINE REDUCTASE NADH BINARY COMPLEX EXPRESSED IN ESCHERICHIA COLI BY A CDNA CONSTRUCTED FROM ITS RAT HOMOLOGUE	EGR	0.54
2I7Q	CRYSTAL STRUCTURE OF HUMAN CHOLINE KINASE A	EGR	0.54
2BH9	X-RAY STRUCTURE OF A DELETION VARIANT OF HUMAN GLUCOSE 6-PHOSPHATE DEHYDROGENASE COMPLEXED WITH STRUCTURAL AND COENZYME NADP	EGR	0.54
1L6J	CRYSTAL STRUCTURE OF HUMAN MATRIX METALLOPROTEINASE MMP9 (GELATINASE B).	EGR	0.55
1JTV	CRYSTAL STRUCTURE OF 17BETA-HYDROXYSTEROID DEHYDROGENASE TYPE 1 COMPLEXED WITH TESTOSTERONE	EGR	0.58
1L6J	CRYSTAL STRUCTURE OF HUMAN MATRIX METALLOPROTEINASE MMP9 (GELATINASE B).	EGR	0.63
2IVV	CRYSTAL STRUCTURE OF PHOSPHORYLATED RET TYROSINE KINASE DOMAIN COMPLEXED WITH THE INHIBITOR PP1	EGR	0.64
1P6F	STRUCTURE OF THE HUMAN NATURAL CYTOTOXICITY RECEPTOR NKP46	EGR	0.64
1N6A	STRUCTURE OF SET7/9	EGR	0.65
1ZSQ	CRYSTAL STRUCTURE OF MTMR2 IN COMPLEX WITH PHOSPHATIDYLINOSITOL 3-PHOSPHATE	EGR	0.67
1ELV	CRYSTAL STRUCTURE OF THE CATALYTIC DOMAIN OF HUMAN COMPLEMENT C1S PROTEASE	EGR	0.74
1GSM	A REASSESSMENT OF THE MADCAM-1 STRUCTURE AND ITS ROLE IN INTEGRIN RECOGNITION.	EGR	0.74
2PET	LUTHERAN GLYCOPROTEIN_ N-TERMINAL DOMAINS 1 AND 2.	EGR	0.75
1LN1	CRYSTAL STRUCTURE OF HUMAN PHOSPHATIDYLCHOLINE TRANSFER PROTEIN IN COMPLEX WITH DILINOLEOYLPHOSPHATIDYLCHOLINE	EGR	0.77
2OI0	CRYSTAL STRUCTURE ANALYSIS 0F THE TNF-A COVERTING ENZYME (TACE) IN COMPLEXED WITH ARYL-SULFONAMIDE	EGR	0.79
2IWR	GTPASE LIKE DOMAIN OF CENTAURIN GAMMA 1 (HUMAN)	EGR	0.83
2O8T	CRYSTAL STRUCTURE AND BINDING EPITOPES OF UROKINASE-TYPE PLASMINOGEN ACTIVATOR (C122A/N145Q) IN COMPLEX WITH INHIBITORS	EGR	2.00
2Q3H	THE CRYSTAL STRUCTURE OF RHOUA IN THE GDP-BOUND STATE.	EGR	2.00

2V9K	CRYSTAL STRUCTURE OF HUMAN PUS10_ A NOVEL PSEUDOURIDINE SYNTHASE.	EGR	2.00
	**elastase-1**		
1WL4	HUMAN CYTOSOLIC ACETOACETYL-COA THIOLASE COMPLEXED WITH COA	AAA	0.05
2UXW	CRYSTAL STRUCTURE OF HUMAN VERY LONG CHAIN ACYL-COA DEHYDROGENASE (ACADVL)	AAA	0.06
2J8Z	CRYSTAL STRUCTURE OF HUMAN P53 INDUCIBLE OXIDOREDUCTASE (TP53I3_PIG3)	AAA	0.07
1LI4	HUMAN S-ADENOSYLHOMOCYSTEINE HYDROLASE COMPLEXED WITH NEPLANOCIN	AAA	0.07
2CZK	CRYSTAL STRUCTURE OF HUMAN MYO-INOSITOL MONOPHOSPHATASE 2 (IMPA2) (TRIGONAL FORM)	AAA	0.07
2OAY	CRYSTAL STRUCTURE OF LATENT HUMAN C1-INHIBITOR	AAA	0.07
1LI4	HUMAN S-ADENOSYLHOMOCYSTEINE HYDROLASE COMPLEXED WITH NEPLANOCIN	AAA	0.08
1P5J	CRYSTAL STRUCTURE ANALYSIS OF HUMAN SERINE DEHYDRATASE	AAA	0.08
1UOU	CRYSTAL STRUCTURE OF HUMAN THYMIDINE PHOSPHORYLASE IN COMPLEX WITH A SMALL MOLECULE INHIBITOR	APA	0.08
2DW4	CRYSTAL STRUCTURE OF HUMAN LSD1 AT 2.3 A RESOLUTION	AAA	0.08
2A7V	HUMAN MITOCHONDRIAL SERINE HYDROXYMETHYLTRANSFERASE 2	APA	0.08
1XM9	STRUCTURE OF THE ARMADILLO REPEAT DOMAIN OF PLAKOPHILIN 1	AAA	0.08
2E9L	CRYSTAL STRUCTURE OF HUMAN CYTOSOLIC NEUTRAL BETA-GLYCOSYLCERAMIDASE (KLOTHO-RELATED PROTE:KLRP) COMPLEX WITH GLUCOSE AND FATTY ACIDS	AAA	0.09
2OAY	CRYSTAL STRUCTURE OF LATENT HUMAN C1-INHIBITOR	AAA	0.09
1KHB	PEPCK COMPLEX WITH NONHYDROLYZABLE GTP ANALOG_ NATIVE DATA	AAA	0.09
2E8A	CRYSTAL STRUCTURE OF THE HUMAN HSP70 ATPASE DOMAIN IN COMPLEX WITH AMP-PNP	AAA	0.09
2QYL	CRYSTAL STRUCTURE OF PDE4B2B IN COMPLEX WITH INHIBITOR NPV	AAA	0.09
1ZKL	MULTIPLE DETERMINANTS FOR INHIBITOR SELECTIVITY OF CYCLIC NUCLEOTIDE PHOSPHODIESTERASES	AAA	0.10
1TAZ	CATALYTIC DOMAIN OF HUMAN PHOSPHODIESTERASE 1B	AAA	0.10
1M6I	CRYSTAL STRUCTURE OF APOPTOSIS INDUCING FACTOR (AIF)	AAA	0.10
1R55	CRYSTAL STRUCTURE OF THE CATALYTIC DOMAIN OF HUMAN ADAM 33	AAA	0.10
1UOH	HUMAN GANKYRIN	AAA	0.11
1UOU	CRYSTAL STRUCTURE OF HUMAN THYMIDINE PHOSPHORYLASE IN COMPLEX WITH A SMALL MOLECULE INHIBITOR	AAA	0.11
2J51	CRYSTAL STRUCTURE OF HUMAN STE20-LIKE KINASE BOUND TO 5- AMINO-3-((4-(AMINOSULFONYL)PHENYL)AMINO)-N-(2_6- DIFLUOROPHENYL)-1H-1_2_4-TRIAZOLE-1-CARBOTHIOAMIDE	AAA	0.11
2QIS	CRYSTAL STRUCTURE OF HUMAN FARNESYL PYROPHOSPHATE SYNTHASE T210S MUTANT BOUND TO RISEDRONATE	AAA	0.11
2Q5I	CRYSTAL STRUCTURE OF APO S581L GLYCYL-TRNA SYNTHETASE MUTAN	AAA	0.12
1XKS	THE CRYSTAL STRUCTURE OF THE N-TERMINAL DOMAIN OF NUP133 REVEALS A BETA-PROPELLER FOLD COMMON TO SEVERAL NUCLEOPORINS	AAA	0.12
2J7T	CRYSTAL STRUCTURE OF HUMAN SERINE THREONINE KINASE-10 BOUND TO SU11274	AAA	0.12
1YZQ	GPPNHP-BOUND RAB6 GTPASE	AAA	0.12
1IMV	2.85 A CRYSTAL STRUCTURE OF PEDF	AAA	0.12
2IQ1	CRYSTAL STRUCTURE OF HUMAN PPM1K	AAA	0.12
2HJW	CRYSTAL STRUCTURE OF THE BC DOMAIN OF ACC2	APA	0.13
2OCG	CRYSTAL STRUCTURE OF HUMAN VALACYCLOVIR HYDROLASE	AAA	0.13
1T67	CRYSTAL STRUCTURE OF HUMAN HDAC8 COMPLEXED WITH MS-344	AAA	0.13
2D7I	CRSYTAL STRUCTURE OF PP-GALNAC-T10 WITH UDP_ GALNAC AND MN2	AAA	0.13
1I1N	HUMAN PROTEIN L-ISOASPARTATE O-METHYLTRANSFERASE WITH S-ADENOSYL HOMOCYSTEINE	AAA	0.13

1JDN	CRYSTAL STRUCTURE OF HORMONE RECEPTOR	APA	0.14
2IPX	HUMAN FIBRILLARIN	AAA	0.14
1JDN	CRYSTAL STRUCTURE OF HORMONE RECEPTOR	AAA	0.14
1HDR	THE CRYSTALLOGRAPHIC STRUCTURE OF A HUMAN DIHYDROPTERIDINE REDUCTASE NADH BINARY COMPLEX EXPRESSED IN ESCHERICHIA COLI BY A CDNA CONSTRUCTED FROM ITS RAT HOMOLOGUE	AAA	0.14
3BKB	CRYSTAL STRUCTURE OF HUMAN FELINE SARCOMA VIRAL ONCOGENE HOMOLOGUE (V-FES)	AAA	0.14
2FG5	CRYSTAL STRUCTURE OF HUMAN RAB31 IN COMPLEX WITH A GTP ANALOGUE	AAA	0.14
1BX4	STRUCTURE OF HUMAN ADENOSINE KINASE AT 1.50 ANGSTROMS	AAA	0.15
1BD8	STRUCTURE OF CDK INHIBITOR P19INK4D	AAA	0.15
2QMJ	CRYSTRAL STRUCTURE OF THE N-TERMINAL SUBUNIT OF HUMAN MALTASE-GLUCOAMYLASE IN COMPLEX WITH ACARBOSE	APA	0.15
1N1F	CRYSTAL STRUCTURE OF HUMAN INTERLEUKIN-19	AAA	0.16
1TXD	CRYSTAL STRUCTURE OF THE DH/PH DOMAINS OF LEUKEMIA-ASSOCIATED RHOGEF	AAA	0.16
2Z6H	CRYSTAL STRUCTURE OF BETA-CATENIN ARMADILLO REPEAT REGION AND ITS C-TERMINAL DOMAIN	AAA	0.16
2C9H	STRUCTURE OF MITOCHONDRIAL BETA-KETOACYL SYNTHASE	AAA	0.16
1SO8	ABETA-BOUND HUMAN ABAD STRUCTURE [ALSO KNOWN AS 3-HYDROXYACYL-COA DEHYDROGENASE TYPE II (TYPE II HADH)_ ENDOPLASMIC RETICULUM-ASSOCIATED AMYLOID BETA-PEPTIDE BINDING PROTEIN (ERAB)]	APA	0.17
1NW3	STRUCTURE OF THE CATALYTIC DOMAIN OF HUMAN DOT1L_ A NON-SET DOMAIN NUCLEOSOMAL HISTONE METHYLTRANSFERASE	AAA	0.17
1ZRH	CRYSTAL STRUCTURE OF HUMAN HEPARAN SULFATE GLUCOSAMINE 3-O-SULFOTRANSFERASE 1 IN COMPLEX WITH PAP	AAA	0.17
1NST	THE SULFOTRANSFERASE DOMAIN OF HUMAN HAPARIN SULFATE N-DEACETYLASE/N-SULFOTRANSFERASE	AAA	0.17
1JJ7	CRYSTAL STRUCTURE OF THE C-TERMINAL ATPASE DOMAIN OF HUMAN TAP1	AAA	0.18
2JI4	HUMAN PHOSPHORIBOSYLPYROPHOSPHATE SYNTHETASE- ASSOCIATED PROTEIN 41 (PAP41)	AAA	0.19
1H0C	THE CRYSTAL STRUCTURE OF HUMAN ALANINE:GLYOXYLATE AMINOTRANSFERASE	AAA	0.19
2OIT	CRYSTAL STRUCTURE OF THE N-TERMINAL DOMAIN OF THE HUMAN PROTO-ONCOGENE NUP214/CAN	AAA	0.19
3BI1	X-RAY STRUCTURE OF HUMAN GLUTAMATE CARBOXYPEPTIDASE II (GCPII) IN COMPLEX WITH A TRANSITION STATE ANALOG OF METHOTREXATE-GLU	AAA	0.19
2UUI	CRYSTAL STRUCTURE OF HUMAN LEUKOTRIENE C4 SYNTHASE	AAA	0.19
2NSM	CRYSTAL STRUCTURE OF THE HUMAN CARBOXYPEPTIDASE N (KININASE I) CATALYTIC DOMAIN	AAA	0.20
2Z5J	FREE TRANSPORTIN 1	AAA	0.21
1NM8	STRUCTURE OF HUMAN CARNITINE ACETYLTRANSFERASE: MOLECULAR BASIS FOR FATTY ACYL TRANSFER	AAA	0.21
1Z70	1.15A RESOLUTION STRUCTURE OF THE FORMYLGLYCINE GENERATING ENZYME FGE	APA	0.21
2UXW	CRYSTAL STRUCTURE OF HUMAN VERY LONG CHAIN ACYL-COA DEHYDROGENASE (ACADVL)	AAA	0.22
1WB0	SPECIFICITY AND AFFINITY OF NATURAL PRODUCT CYCLOPENTAPEPTIDE INHIBITOR ARGIFIN AGAINST HUMAN CHITINAS	APA	0.22
2J8Z	CRYSTAL STRUCTURE OF HUMAN P53 INDUCIBLE OXIDOREDUCTASE (TP53I3_PIG3)	AAA	0.23
3C5H	CRYSTAL STRUCTURE OF THE RAS HOMOLOG DOMAIN OF HUMAN GRLF1 (P190RHOGAP)	AAA	0.23
1LCY	CRYSTAL STRUCTURE OF THE MITOCHONDRIAL SERINE PROTEASE HTRA	APA	0.23

1ZSY	THE STRUCTURE OF HUMAN MITOCHONDRIAL 2-ENOYL THIOESTER REDUCTASE (CGI-63)	AAA	0.23
2ILA	STRUCTURE OF INTERLEUKIN 1ALPHA AT 2.7-ANGSTROMS RESOLUTION	AAA	0.24
1UOU	CRYSTAL STRUCTURE OF HUMAN THYMIDINE PHOSPHORYLASE IN COMPLEX WITH A SMALL MOLECULE INHIBITOR	AAA	0.24
1OZN	1.5A CRYSTAL STRUCTURE OF THE NOGO RECEPTOR LIGAND BINDING DOMAIN REVEALS A CONVERGENT RECOGNITION SCAFFOLD MEDIATING INHIBITION OF MYELINATION	AAA	0.24
1LJ5	1.8A RESOLUTION STRUCTURE OF LATENT PLASMINOGEN ACTIVATOR INHIBITOR-1(PAI-1)	APA	0.24
2G62	CRYSTAL STRUCTURE OF HUMAN PTPA	AAA	0.26
2EBC	MECHANISM UNDERLYING THE CRITICAL CONTRIBUTION OF A SWITCH II RESIDUE IN A HETEROTRIMERIC G-PROTEIN ALPHA SUBUNIT DURING C. ELEGANS ASYMMETRIC CELL DIVISION	AAA	0.26
1YHV	CRYSTAL STRUCTURE OF PAK1 KINASE DOMAIN WITH TWO POINT MUTATIONS (K299R_ T423E)	AAA	0.26
1ZV4	STRUCTURE OF THE REGULATOR OF G-PROTEIN SIGNALING 17 (RGSZ2	APA	0.27
2OYC	CRYSTAL STRUCTURE OF HUMAN PYRIDOXAL PHOSPHATE PHOSPHATASE	AAA	0.28
1J72	CRYSTAL STRUCTURE OF MUTANT MACROPHAGE CAPPING PROTEIN (CAP G) WITH ACTIN-SEVERING ACTIVITY IN THE CA2+-FREE FORM	AAA	0.28
3BI7	CRYSTAL STRUCTURE OF THE SRA DOMAIN OF E3 UBIQUITIN-PROTEIN LIGASE UHRF1	APA	0.29
3COU	CRYSTAL STRUCTURE OF HUMAN NUDIX MOTIF 16 (NUDT16)	AAA	0.29
1JTV	CRYSTAL STRUCTURE OF 17BETA-HYDROXYSTEROID DEHYDROGENASE TYPE 1 COMPLEXED WITH TESTOSTERONE	AAA	0.30
2B9E	HUMAN NSUN5 PROTEIN	APA	0.30
1ZED	ALKALINE PHOSPHATASE FROM HUMAN PLACENTA IN COMPLEX WITH P-NITROPHENYL-PHOSPHONATE	AAA	0.31
2OKK	THE X-RAY CRYSTAL STRUCTURE OF THE 65KDA ISOFORM OF GLUTAMIC ACID DECARBOXYLASE (GAD65)	AAA	0.31
2E8A	CRYSTAL STRUCTURE OF THE HUMAN HSP70 ATPASE DOMAIN IN COMPLEX WITH AMP-PNP	AAA	0.31
3BCH	CRYSTAL STRUCTURE OF THE HUMAN LAMININ RECEPTOR PRECURSOR	AAA	0.34
1SEN	ENDOPLASMIC RETICULUM PROTEIN RP19 O95881	AAA	0.34
2OEW	STRUCTURE OF ALIX/AIP1 BRO1 DOMAIN	AAA	0.34
1T46	STRUCTURAL BASIS FOR THE AUTOINHIBITION AND STI-571 INHIBITION OF C-KIT TYROSINE KINASE	APA	0.35
1SK4	CRYSTAL STRUCTURE OF THE C-TERMINAL PEPTIDOGLYCAN-BINDING DOMAIN OF HUMAN PEPTIDOGLYCAN RECOGNITION PROTEIN IALPHA	AAA	0.35
2E3N	CRYSTAL STRUCTURE OF CERT START DOMAIN IN COMPLEX WITH C6-CERAMIDE (P212121)	APA	0.35
2Q0Z	CRYSTAL STRUCTURE OF Q9P172/SEC63 FROM HOMO SAPIENS. NORTHEAST STRUCTURAL GENOMICS TARGET HR1979.	APA	0.35
2ALD	HUMAN MUSCLE ALDOLASE	AAA	0.37
1YZQ	GPPNHP-BOUND RAB6 GTPASE	AAA	0.37
1UOU	CRYSTAL STRUCTURE OF HUMAN THYMIDINE PHOSPHORYLASE IN COMPLEX WITH A SMALL MOLECULE INHIBITOR	APA	0.37
1IMJ	CRYSTAL STRUCTURE OF THE HUMAN CCG1/TAFII250-INTERACTING FACTOR B (CIB)	AAA	0.38
2RIP	STRUCTURE OF DPPIV IN COMPLEX WITH AN INHIBITOR	APA	0.39
3COU	CRYSTAL STRUCTURE OF HUMAN NUDIX MOTIF 16 (NUDT16)	AAA	0.39
1W6K	STRUCTURE OF HUMAN OSC IN COMPLEX WITH LANOSTEROL	APA	0.39
1QNT	X-RAY STRUCTURE OF HUMAN O6ALKYLGUANINE-DNA ALKYLTRANSFERAS	APA	0.40
1Z8D	CRYSTAL STRUCTURE OF HUMAN MUSCLE GLYCOGEN PHOSPHORYLASE A WITH AMP AND GLUCOSE	AAA	0.40
1ZXQ	THE CRYSTAL STRUCTURE OF ICAM-2	APA	0.42
1ZSY	THE STRUCTURE OF HUMAN MITOCHONDRIAL 2-ENOYL THIOESTER REDUCTASE (CGI-63)	APA	0.42

2OUD	CRYSTAL STRUCTURE OF THE CATALYTIC DOMAIN OF HUMAN MKP5	AAA	0.42
1IMJ	CRYSTAL STRUCTURE OF THE HUMAN CCG1/TAFII250-INTERACTING FACTOR B (CIB)	APA	0.43
2QMJ	CRYSTRAL STRUCTURE OF THE N-TERMINAL SUBUNIT OF HUMAN MALTASE-GLUCOAMYLASE IN COMPLEX WITH ACARBOSE	AAA	0.43
1WAR	RECOMBINANT HUMAN PURPLE ACID PHOSPHATASE EXPRESSED IN PICHIA PASTORIS	AAA	0.45
1MRQ	CRYSTAL STRUCTURE OF HUMAN 20ALPHA-HSD IN TERNARY COMPLEX WITH NADP AND 20ALPHA-HYDROXY-PROGESTERONE	APA	0.46
2H44	CRYSTAL STRUCTURE OF PDE5A1 IN COMPLEX WITH ICARISID II	AAA	0.49
1TBF	CATALYTIC DOMAIN OF HUMAN PHOSPHODIESTERASE 5A IN COMPLEX WITH SILDENAFIL	AAA	0.49
1H6O	DIMERISATION DOMAIN FROM HUMAN TRF1	AAA	0.50
1JDN	CRYSTAL STRUCTURE OF HORMONE RECEPTOR	AAA	0.50
2IMS	THE X-RAY STRUCTURE OF A BAK HOMODIMER REVEALS AN INHIBITORY ZINC BINDING SITE	APA	0.51
1R55	CRYSTAL STRUCTURE OF THE CATALYTIC DOMAIN OF HUMAN ADAM 33	AAA	0.51
1QMN	ALPHA1-ANTICHYMOTRYPSIN SERPIN IN THE DELTA CONFORMATION (PARTIAL LOOP INSERTION)	AAA	0.51
2DH2	CRYSTAL STRUCTURE OF HUMAN ED-4F2HC	AAA	0.51
3BO5	CRYSTAL STRUCTURE OF METHYLTRANSFERASE DOMAIN OF HUMAN HISTONE-LYSINE N-METHYLTRANSFERASE SETMAR	APA	0.52
1OXZ	CRYSTAL STRUCTURE OF THE HUMAN GGA1 GAT DOMAIN	AAA	0.52
1WB0	SPECIFICITY AND AFFINITY OF NATURAL PRODUCT CYCLOPENTAPEPTIDE INHIBITOR ARGIFIN AGAINST HUMAN CHITINAS	AAA	0.53
3C8X	CRYSTAL STRUCTURE OF THE LIGAND BINDING DOMAIN OF HUMAN EPHRIN A2 (EPHA2) RECEPTOR PROTEIN KINASE	AAA	0.56
1R55	CRYSTAL STRUCTURE OF THE CATALYTIC DOMAIN OF HUMAN ADAM 33	AAA	0.56
2A14	CRYSTAL STRUCTURE OF HUMAN INDOLETHYLAMINE N-METHYLTRANSFERASE WITH SAH	APA	0.58
1Z70	1.15A RESOLUTION STRUCTURE OF THE FORMYLGLYCINE GENERATING ENZYME FGE	AAA	0.59
1N5U	X-RAY STUDY OF HUMAN SERUM ALBUMIN COMPLEXED WITH HEME	AAA	0.65
2F4J	STRUCTURE OF THE KINASE DOMAIN OF AN IMATINIB-RESISTANT ABL MUTANT IN COMPLEX WITH THE AURORA KINASE INHIBITOR VX-680	APA	0.68
2FCB	HUMAN FC GAMMA RECEPTOR IIB ECTODOMAIN (CD32)	APA	0.75
2FY2	STRUCTURES OF LIGAND BOUND HUMAN CHOLINE ACETYLTRANSFERASE PROVIDE INSIGHT INTO REGULATION OF ACETYLCHOLINE SYNTHESIS	AAA	0.77
3CKK	CRYSTAL STRUCTURE OF HUMAN METHYLTRANSFERASE-LIKE PROTEIN 1	APA	0.79
1DHS	CRYSTAL STRUCTURE OF THE NAD COMPLEX OF HUMAN DEOXYHYPUSINE SYNTHASE	APA	0.97
1H30	C-TERMINAL LG DOMAIN PAIR OF HUMAN GAS6	AAA	1.33
1BY7	HUMAN PLASMINOGEN ACTIVATOR INHIBITOR-2. LOOP (66-98) DELETION MUTANT	AAA	2.00
1E8Y	STRUCTURE DETERMINANTS OF PHOSPHOINOSITIDE 3-KINASE INHIBITION BY WORTMANNIN_ LY294002_ QUERCETIN_ MYRICETIN AND STAUROSPORINE	APA	2.00
1FCG	ECTODOMAIN OF HUMAN FC GAMMA RECEPTOR_ FCGRIIA	AAA	2.00
1FSU	4-SULFATASE (HUMAN)	APA	2.00
1JJ7	CRYSTAL STRUCTURE OF THE C-TERMINAL ATPASE DOMAIN OF HUMAN TAP1	APA	2.00
1PJA	THE CRYSTAL STRUCTURE OF PALMITOYL PROTEIN THIOESTERASE-2 REVEALS THE BASIS FOR DIVERGENT SUBSTRATE SPECIFICITIES OF THE TWO LYSOSOMAL THIOESTERASES (PPT1 AND PPT2)	APA	2.00
1PME	STRUCTURE OF PENTA MUTANT HUMAN ERK2 MAP KINASE COMPLEXED WITH A SPECIFIC INHIBITOR OF HUMAN P38 MAP KINASE	AAA	2.00
1R2D	STRUCTURE OF HUMAN BCL-XL AT 1.95 ANGSTROMS	AAA	2.00

1SL4	CRYSTAL STRUCTURE OF DC-SIGN CARBOHYDRATE RECOGNITION DOMAIN COMPLEXED WITH MAN4	APA	2.00
1SO8	ABETA-BOUND HUMAN ABAD STRUCTURE [ALSO KNOWN AS 3-HYDROXYACYL-COA DEHYDROGENASE TYPE II (TYPE II HADH)_ ENDOPLASMIC RETICULUM-ASSOCIATED AMYLOID BETA-PEPTIDE BINDING PROTEIN (ERAB)]	AAA	2.00
1SZ7	CRYSTAL STRUCTURE OF HUMAN BET3	AAA	2.00
1TA0	THREE-DIMENSIONAL STRUCTURE OF A RNA-POLYMERASE II BINDING PROTEIN WITH ASSOCIATED LIGAND.	AAA	2.00
1WAK	X-RAY STRUCTURE OF SRPK1	APA	2.00
1YGS	CRYSTAL STRUCTURE OF THE SMAD4 TUMOR SUPPRESSOR C-TERMINAL DOMAIN	APA	2.00
1YGS	CRYSTAL STRUCTURE OF THE SMAD4 TUMOR SUPPRESSOR C-TERMINAL DOMAIN	AAA	2.00
1ZSQ	CRYSTAL STRUCTURE OF MTMR2 IN COMPLEX WITH PHOSPHATIDYLINOSITOL 3-PHOSPHATE	AAA	2.00
2AA2	MINERALOCORTICOID RECEPTOR WITH BOUND ALDOSTERONE	APA	2.00
2D7I	CRSYTAL STRUCTURE OF PP-GALNAC-T10 WITH UDP_ GALNAC AND MN2	APA	2.00
2QOL	HUMAN EPHA3 KINASE AND JUXTAMEMBRANE REGION_ Y596:Y602:S768G TRIPLE MUTANT	AAA	2.00
2QTZ	CRYSTAL STRUCTURE OF THE NADP+-BOUND FAD-CONTAINING FNR- LIKE MODULE OF HUMAN METHIONINE SYNTHASE REDUCTASE	APA	2.00
2REP	CRYSTAL STRUCTURE OF THE MOTOR DOMAIN OF HUMAN KINESIN FAMILY MEMBER C1	APA	2.00
2UW2	CRYSTAL STRUCTURE OF HUMAN RIBONUCLEOTIDE REDUCTASE SUBUNIT R2	AAA	2.00
2V9R	FIRST AND SECOND IG DOMAINS FROM HUMAN ROBO1 (FORM 2)	AAA	2.00
2VR2	HUMAN DIHYDROPYRIMIDINASE	APA	2.00
2ZMD	CRYSTAL STRUCTURE OF HUMAN MPS1 CATALYTIC DOMAIN T686A MUTANT IN COMPLEX WITH SP600125 INHIBITOR	AAA	2.00
	**elastase-2**		
2C9H	STRUCTURE OF MITOCHONDRIAL BETA-KETOACYL SYNTHASE	AAV	0.05
1WL4	HUMAN CYTOSOLIC ACETOACETYL-COA THIOLASE COMPLEXED WITH COA	AAA	0.05
1WL4	HUMAN CYTOSOLIC ACETOACETYL-COA THIOLASE COMPLEXED WITH COA	AAV	0.05
2UXW	CRYSTAL STRUCTURE OF HUMAN VERY LONG CHAIN ACYL-COA DEHYDROGENASE (ACADVL)	AAA	0.06
2J8Z	CRYSTAL STRUCTURE OF HUMAN P53 INDUCIBLE OXIDOREDUCTASE (TP53I3_PIG3)	AAA	0.07
1LI4	HUMAN S-ADENOSYLHOMOCYSTEINE HYDROLASE COMPLEXED WITH NEPLANOCIN	AAA	0.07
2E8A	CRYSTAL STRUCTURE OF THE HUMAN HSP70 ATPASE DOMAIN IN COMPLEX WITH AMP-PNP	AAV	0.07
1WL4	HUMAN CYTOSOLIC ACETOACETYL-COA THIOLASE COMPLEXED WITH COA	AAV	0.07
2CZK	CRYSTAL STRUCTURE OF HUMAN MYO-INOSITOL MONOPHOSPHATASE 2 (IMPA2) (TRIGONAL FORM)	AAA	0.07
2OAY	CRYSTAL STRUCTURE OF LATENT HUMAN C1-INHIBITOR	AAA	0.07
1LI4	HUMAN S-ADENOSYLHOMOCYSTEINE HYDROLASE COMPLEXED WITH NEPLANOCIN	AAA	0.08
1P5J	CRYSTAL STRUCTURE ANALYSIS OF HUMAN SERINE DEHYDRATASE	AAA	0.08
2DW4	CRYSTAL STRUCTURE OF HUMAN LSD1 AT 2.3 A RESOLUTION	AAA	0.08
1PEX	COLLAGENASE-3 (MMP-13) C-TERMINAL HEMOPEXIN-LIKE DOMAIN	AAV	0.08
1XM9	STRUCTURE OF THE ARMADILLO REPEAT DOMAIN OF PLAKOPHILIN 1	AAA	0.08
3BI1	X-RAY STRUCTURE OF HUMAN GLUTAMATE CARBOXYPEPTIDASE II (GCPII) IN COMPLEX WITH A TRANSITION STATE ANALOG OF METHOTREXATE-GLU	AAV	0.08

2E9L	CRYSTAL STRUCTURE OF HUMAN CYTOSOLIC NEUTRAL BETA-GLYCOSYLCERAMIDASE (KLOTHO-RELATED PROTE:KLRP) COMPLEX WITH GLUCOSE AND FATTY ACIDS	AAA	0.09
2OAY	CRYSTAL STRUCTURE OF LATENT HUMAN C1-INHIBITOR	AAA	0.09
1KHB	PEPCK COMPLEX WITH NONHYDROLYZABLE GTP ANALOG_ NATIVE DATA	AAA	0.09
2E8A	CRYSTAL STRUCTURE OF THE HUMAN HSP70 ATPASE DOMAIN IN COMPLEX WITH AMP-PNP	AAA	0.09
1YZQ	GPPNHP-BOUND RAB6 GTPASE	AAV	0.09
2QYL	CRYSTAL STRUCTURE OF PDE4B2B IN COMPLEX WITH INHIBITOR NPV	AAA	0.09
2V5O	STRUCTURE OF HUMAN IGF2R DOMAINS 11-14	AAV	0.09
2ICA	CD11A (LFA1) I-DOMAIN COMPLEXED WITH BMS-587101 AKA 5-[(5S_ 9R)-9-(4-CYANOPHENYL)-3-(3_5-DICHLOROPHENYL)-1-METHYL-2_4- DIOXO-1_3_7-TRIAZASPIRO [4.4]NON-7-YL]METHYL]-3- THIOPHENECARBOXYLICACID	AAV	0.09
2A7V	HUMAN MITOCHONDRIAL SERINE HYDROXYMETHYLTRANSFERASE 2	AAV	0.10
1ZKL	MULTIPLE DETERMINANTS FOR INHIBITOR SELECTIVITY OF CYCLIC NUCLEOTIDE PHOSPHODIESTERASES	AAA	0.10
1TAZ	CATALYTIC DOMAIN OF HUMAN PHOSPHODIESTERASE 1B	AAA	0.10
1WDY	CRYSTAL STRUCTURE OF RIBONUCLEASE	AAV	0.10
1M6I	CRYSTAL STRUCTURE OF APOPTOSIS INDUCING FACTOR (AIF)	AAA	0.10
1ZD3	HUMAN SOLUBLE EPOXIDE HYDROLASE 4-(3-CYCLOHEXYLURIEDO)-BUTYRIC ACID COMPLEX	AAV	0.10
1R55	CRYSTAL STRUCTURE OF THE CATALYTIC DOMAIN OF HUMAN ADAM 33	AAA	0.10
3GRS	REFINED STRUCTURE OF GLUTATHIONE REDUCTASE AT 1.54 ANGSTROMS RESOLUTION	AAV	0.10
1S31	CRYSTAL STRUCTURE ANALYSIS OF THE HUMAN TUB PROTEIN (ISOFORM A) SPANNING RESIDUES 289 THROUGH 561	AAV	0.10
2BH9	X-RAY STRUCTURE OF A DELETION VARIANT OF HUMAN GLUCOSE 6-PHOSPHATE DEHYDROGENASE COMPLEXED WITH STRUCTURAL AND COENZYME NADP	AAV	0.10
2FG5	CRYSTAL STRUCTURE OF HUMAN RAB31 IN COMPLEX WITH A GTP ANALOGUE	AAV	0.11
1UOH	HUMAN GANKYRIN	AAA	0.11
1UOU	CRYSTAL STRUCTURE OF HUMAN THYMIDINE PHOSPHORYLASE IN COMPLEX WITH A SMALL MOLECULE INHIBITOR	AAA	0.11
2J51	CRYSTAL STRUCTURE OF HUMAN STE20-LIKE KINASE BOUND TO 5- AMINO-3-((4-(AMINOSULFONYL)PHENYL)AMINO)-N-(2_6- DIFLUOROPHENYL)-1H-1_2_4-TRIAZOLE-1-CARBOTHIOAMIDE	AAA	0.11
1P5J	CRYSTAL STRUCTURE ANALYSIS OF HUMAN SERINE DEHYDRATASE	AAV	0.11
2QIS	CRYSTAL STRUCTURE OF HUMAN FARNESYL PYROPHOSPHATE SYNTHASE T210S MUTANT BOUND TO RISEDRONATE	AAA	0.11
2Q5I	CRYSTAL STRUCTURE OF APO S581L GLYCYL-TRNA SYNTHETASE MUTAN	AAA	0.12
2UW2	CRYSTAL STRUCTURE OF HUMAN RIBONUCLEOTIDE REDUCTASE SUBUNIT R2	AAV	0.12
2BIK	HUMAN PIM1 PHOSPHORYLATED ON SER261	AAV	0.12
1XKS	THE CRYSTAL STRUCTURE OF THE N-TERMINAL DOMAIN OF NUP133 REVEALS A BETA-PROPELLER FOLD COMMON TO SEVERAL NUCLEOPORINS	AAA	0.12
1W7L	CRYSTAL STRUCTURE OF HUMAN KYNURENINE AMINOTRANSFERASE I	AAV	0.12
2J7T	CRYSTAL STRUCTURE OF HUMAN SERINE THREONINE KINASE-10 BOUND TO SU11274	AAA	0.12
1R1H	STRUCTURAL ANALYSIS OF NEPRILYSIN WITH VARIOUS SPECIFIC AND POTENT INHIBITORS	AAV	0.12
1IMV	2.85 A CRYSTAL STRUCTURE OF PEDF	AAV	0.12
2FVV	HUMAN DIPHOSPHOINOSITOL POLYPHOSPHATE PHOSPHOHYDROLASE 1	AAV	0.12
1YZQ	GPPNHP-BOUND RAB6 GTPASE	AAA	0.12
1CZA	MUTANT MONOMER OF RECOMBINANT HUMAN HEXOKINASE TYPE I COMPLEXED WITH GLUCOSE_ GLUCOSE-6-PHOSPHATE_ AND ADP	AAV	0.12
1IMV	2.85 A CRYSTAL STRUCTURE OF PEDF	AAA	0.12

2J5W	CERULOPLASMIN REVISITED: STRUCTURAL AND FUNCTIONAL ROLES OF VARIOUS METAL CATION BINDING SITES	AAV	0.12
2IQ1	CRYSTAL STRUCTURE OF HUMAN PPM1K	AAA	0.12
2OGQ	MOLECULAR AND STRUCTURAL BASIS OF PLK1 SUBSTRATE RECOGNITION: IMPLICATIONS IN CENTROSOMAL LOCALIZATION	AAV	0.13
2J0I	CRYSTAL STRUCTURE OF THE HUMAN P21-ACTIVATED KINASE 4	AAV	0.13
2OCG	CRYSTAL STRUCTURE OF HUMAN VALACYCLOVIR HYDROLASE	AAA	0.13
1T67	CRYSTAL STRUCTURE OF HUMAN HDAC8 COMPLEXED WITH MS-344	AAA	0.13
2H79	CRYSTAL STRUCTURE OF HUMAN TR ALPHA BOUND T3 IN ORTHORHOMBIC SPACE GROUP	AAV	0.13
2H6W	THYROID HORMONE RECEPTOR BOUND TO T3	AAV	0.13
2D7I	CRSYTAL STRUCTURE OF PP-GALNAC-T10 WITH UDP_ GALNAC AND MN2	AAA	0.13
1YHV	CRYSTAL STRUCTURE OF PAK1 KINASE DOMAIN WITH TWO POINT MUTATIONS (K299R_ T423E)	AAV	0.13
1IMF	STRUCTURAL STUDIES OF METAL BINDING BY INOSITOL MONOPHOSPHATASE: EVIDENCE FOR TWO-METAL ION CATALYSIS	AAV	0.13
1I1N	HUMAN PROTEIN L-ISOASPARTATE O-METHYLTRANSFERASE WITH S-ADENOSYL HOMOCYSTEINE	AAA	0.13
2OC3	CRYSTAL STRUCTURE OF THE CATALYTIC DOMAIN OF HUMAN PROTEIN TYROSINE PHOSPHATASE NON-RECEPTOR TYPE 18	AAV	0.14
2IPX	HUMAN FIBRILLARIN	AAA	0.14
1JJ7	CRYSTAL STRUCTURE OF THE C-TERMINAL ATPASE DOMAIN OF HUMAN TAP1	AAV	0.14
1JDN	CRYSTAL STRUCTURE OF HORMONE RECEPTOR	AAA	0.14
1OW1	CRYSTAL STRUCTURE OF THE SPOC DOMAIN OF THE HUMAN TRANSCRIPTIONAL COREPRESSOR_ SHARP.	AAV	0.14
1HDR	THE CRYSTALLOGRAPHIC STRUCTURE OF A HUMAN DIHYDROPTERIDINE REDUCTASE NADH BINARY COMPLEX EXPRESSED IN ESCHERICHIA COLI BY A CDNA CONSTRUCTED FROM ITS RAT HOMOLOGUE	AAA	0.14
3BKB	CRYSTAL STRUCTURE OF HUMAN FELINE SARCOMA VIRAL ONCOGENE HOMOLOGUE (V-FES)	AAA	0.14
2FG5	CRYSTAL STRUCTURE OF HUMAN RAB31 IN COMPLEX WITH A GTP ANALOGUE	AAA	0.14
1YZG	STRUCTURE OF HUMAN ADP-RIBOSYLATION FACTOR-LIKE 8	AAV	0.14
1WB0	SPECIFICITY AND AFFINITY OF NATURAL PRODUCT CYCLOPENTAPEPTIDE INHIBITOR ARGIFIN AGAINST HUMAN CHITINAS	AAV	0.14
1BX4	STRUCTURE OF HUMAN ADENOSINE KINASE AT 1.50 ANGSTROMS	AAA	0.15
1JJ7	CRYSTAL STRUCTURE OF THE C-TERMINAL ATPASE DOMAIN OF HUMAN TAP1	AAV	0.15
1BD8	STRUCTURE OF CDK INHIBITOR P19INK4D	AAA	0.15
2Z6H	CRYSTAL STRUCTURE OF BETA-CATENIN ARMADILLO REPEAT REGION AND ITS C-TERMINAL DOMAIN	AAV	0.16
1N1F	CRYSTAL STRUCTURE OF HUMAN INTERLEUKIN-19	AAA	0.16
2HJW	CRYSTAL STRUCTURE OF THE BC DOMAIN OF ACC2	AAV	0.16
2QCF	CRYSTAL STRUCTURE OF THE OROTIDINE-5'-MONOPHOSPHATE DECARBOXYLASE DOMAIN (ASP312ASN MUTANT) OF HUMAN UMP SYNTHASE BOUND TO 5-FLUORO-UMP	AAV	0.16
1TXD	CRYSTAL STRUCTURE OF THE DH/PH DOMAINS OF LEUKEMIA-ASSOCIATED RHOGEF	AAA	0.16
2Z6H	CRYSTAL STRUCTURE OF BETA-CATENIN ARMADILLO REPEAT REGION AND ITS C-TERMINAL DOMAIN	AAA	0.16
2C9H	STRUCTURE OF MITOCHONDRIAL BETA-KETOACYL SYNTHASE	AAA	0.16
1WB0	SPECIFICITY AND AFFINITY OF NATURAL PRODUCT CYCLOPENTAPEPTIDE INHIBITOR ARGIFIN AGAINST HUMAN CHITINAS	AAV	0.16
2Z6H	CRYSTAL STRUCTURE OF BETA-CATENIN ARMADILLO REPEAT REGION AND ITS C-TERMINAL DOMAIN	AAV	0.17

2VIJ	HUMAN BACE-1 IN COMPLEX WITH 3-(1_1-DIOXIDOTETRAHYDRO-2H-1_ 2-THIAZIN-2-YL)-5-(ETHYLAMINO)-N-((1S_2R)-2-HYDROXY-1-(PHENYLMETHYL)-3-(1_2_3_4-TETRAHYDRO-1-NAPHTHALENYLAMINO) PROPYL)BENZAMIDE	AAV	0.17
1NW3	STRUCTURE OF THE CATALYTIC DOMAIN OF HUMAN DOT1L_ A NON-SET DOMAIN NUCLEOSOMAL HISTONE METHYLTRANSFERASE	AAA	0.17
2QDJ	CRYSTAL STRUCTURE OF THE RETINOBLASTOMA PROTEIN N-DOMAIN PROVIDES INSIGHT INTO TUMOR SUPPRESSION_ LIGAND INTERACTION AND HOLOPROTEIN ARCHITECTURE	AAV	0.17
1ZRH	CRYSTAL STRUCTURE OF HUMAN HEPARAN SULFATE GLUCOSAMINE 3-O-SULFOTRANSFERASE 1 IN COMPLEX WITH PAP	AAA	0.17
1NST	THE SULFOTRANSFERASE DOMAIN OF HUMAN HAPARIN SULFATE N-DEACETYLASE/N-SULFOTRANSFERASE	AAA	0.17
1M6I	CRYSTAL STRUCTURE OF APOPTOSIS INDUCING FACTOR (AIF)	AAV	0.18
1JJ7	CRYSTAL STRUCTURE OF THE C-TERMINAL ATPASE DOMAIN OF HUMAN TAP1	AAA	0.18
1R5L	CRYSTAL STRUCTURE OF HUMAN ALPHA-TOCOPHEROL TRANSFER PROTEIN BOUND TO ITS LIGAND	AAV	0.18
2BLE	STRUCTURE OF HUMAN GUANOSINE MONOPHOSPHATE REDUCTASE GMPR1 IN COMPLEX WITH GMP	AAV	0.18
2JI4	HUMAN PHOSPHORIBOSYLPYROPHOSPHATE SYNTHETASE- ASSOCIATED PROTEIN 41 (PAP41)	AAA	0.19
1H0C	THE CRYSTAL STRUCTURE OF HUMAN ALANINE:GLYOXYLATE AMINOTRANSFERASE	AAA	0.19
2OIT	CRYSTAL STRUCTURE OF THE N-TERMINAL DOMAIN OF THE HUMAN PROTO-ONCOGENE NUP214/CAN	AAA	0.19
3BI1	X-RAY STRUCTURE OF HUMAN GLUTAMATE CARBOXYPEPTIDASE II (GCPII) IN COMPLEX WITH A TRANSITION STATE ANALOG OF METHOTREXATE-GLU	AAA	0.19
2UUI	CRYSTAL STRUCTURE OF HUMAN LEUKOTRIENE C4 SYNTHASE	AAA	0.19
2OEW	STRUCTURE OF ALIX/AIP1 BRO1 DOMAIN	AAV	0.20
2NSM	CRYSTAL STRUCTURE OF THE HUMAN CARBOXYPEPTIDASE N (KININASE I) CATALYTIC DOMAIN	AAA	0.20
1F5N	HUMAN GUANYLATE BINDING PROTEIN-1 IN COMPLEX WITH THE GTP ANALOGUE_ GMPPNP.	AAV	0.20
2ALD	HUMAN MUSCLE ALDOLASE	AAV	0.20
1E8Y	STRUCTURE DETERMINANTS OF PHOSPHOINOSITIDE 3-KINASE INHIBITION BY WORTMANNIN_ LY294002_ QUERCETIN_ MYRICETIN AND STAUROSPORINE	AAV	0.20
2Z5J	FREE TRANSPORTIN 1	AAA	0.21
2G62	CRYSTAL STRUCTURE OF HUMAN PTPA	AAV	0.21
1NM8	STRUCTURE OF HUMAN CARNITINE ACETYLTRANSFERASE: MOLECULAR BASIS FOR FATTY ACYL TRANSFER	AAA	0.21
2CZK	CRYSTAL STRUCTURE OF HUMAN MYO-INOSITOL MONOPHOSPHATASE 2 (IMPA2) (TRIGONAL FORM)	AAV	0.21
1LCT	STRUCTURE OF THE RECOMBINANT N-TERMINAL LOBE OF HUMAN LACTOFERRIN AT 2.0 ANGSTROMS RESOLUTION	AAV	0.21
1E2S	CRYSTAL STRUCTURE OF AN ARYLSULFATASE A MUTANT C69A	AAV	0.21
2ALR	ALDEHYDE REDUCTASE	AAV	0.21
2HRB	CRYSTAL STRUCTURE OF HUMAN CARBONYL REDUCTASE 3_ COMPLEXED WITH NADP+	AAV	0.22
2UXW	CRYSTAL STRUCTURE OF HUMAN VERY LONG CHAIN ACYL-COA DEHYDROGENASE (ACADVL)	AAA	0.22
1WMA	CRYSTAL STRUCTURE OF HUMAN CBR1 IN COMPLEX WITH HYDROXY-PP	AAV	0.22
2J8Z	CRYSTAL STRUCTURE OF HUMAN P53 INDUCIBLE OXIDOREDUCTASE (TP53I3_PIG3)	AAA	0.23
3C5H	CRYSTAL STRUCTURE OF THE RAS HOMOLOG DOMAIN OF HUMAN GRLF1 (P190RHOGAP)	AAA	0.23

1NHZ	CRYSTAL STRUCTURE OF THE ANTAGONIST FORM OF GLUCOCORTICOID RECEPTOR	AAV	0.23
1ZSY	THE STRUCTURE OF HUMAN MITOCHONDRIAL 2-ENOYL THIOESTER REDUCTASE (CGI-63)	AAA	0.23
1UOU	CRYSTAL STRUCTURE OF HUMAN THYMIDINE PHOSPHORYLASE IN COMPLEX WITH A SMALL MOLECULE INHIBITOR	AAV	0.23
2ILA	STRUCTURE OF INTERLEUKIN 1ALPHA AT 2.7-ANGSTROMS RESOLUTION	AAA	0.24
1UOU	CRYSTAL STRUCTURE OF HUMAN THYMIDINE PHOSPHORYLASE IN COMPLEX WITH A SMALL MOLECULE INHIBITOR	AAA	0.24
1IAP	CRYSTAL STRUCTURE OF P115RHOGEF RGRGS DOMAIN	AAV	0.24
1OZN	1.5A CRYSTAL STRUCTURE OF THE NOGO RECEPTOR LIGAND BINDING DOMAIN REVEALS A CONVERGENT RECOGNITION SCAFFOLD MEDIATING INHIBITION OF MYELINATION	AAA	0.24
1IMJ	CRYSTAL STRUCTURE OF THE HUMAN CCG1/TAFII250-INTERACTING FACTOR B (CIB)	AAV	0.24
2FFX	STRUCTURE OF HUMAN FERRITIN L. CHAIN	AAV	0.24
2A14	CRYSTAL STRUCTURE OF HUMAN INDOLETHYLAMINE N-METHYLTRANSFERASE WITH SAH	AAV	0.25
2F4J	STRUCTURE OF THE KINASE DOMAIN OF AN IMATINIB-RESISTANT ABL MUTANT IN COMPLEX WITH THE AURORA KINASE INHIBITOR VX-680	AAV	0.25
2VIJ	HUMAN BACE-1 IN COMPLEX WITH 3-(1_1-DIOXIDOTETRAHYDRO-2H-1_ 2-THIAZIN-2-YL)-5-(ETHYLAMINO)-N-((1S_2R)-2-HYDROXY-1-(PHENYLMETHYL)-3-(1_2_3_4-TETRAHYDRO-1-NAPHTHALENYLAMINO) PROPYL)BENZAMIDE	AAV	0.26
2G62	CRYSTAL STRUCTURE OF HUMAN PTPA	AAA	0.26
2EBC	MECHANISM UNDERLYING THE CRITICAL CONTRIBUTION OF A SWITCH II RESIDUE IN A HETEROTRIMERIC G-PROTEIN ALPHA SUBUNIT DURING C. ELEGANS ASYMMETRIC CELL DIVISION	AAA	0.26
1YHV	CRYSTAL STRUCTURE OF PAK1 KINASE DOMAIN WITH TWO POINT MUTATIONS (K299R_ T423E)	AAA	0.26
2OIL	CRYSTAL STRUCTURE OF HUMAN RAB25 IN COMPLEX WITH GDP	AAV	0.26
1XBB	CRYSTAL STRUCTURE OF THE SYK TYROSINE KINASE DOMAIN WITH GLEEVEC	AAV	0.27
2V7O	CRYSTAL STRUCTURE OF HUMAN CALCIUM-CALMODULIN-DEPENDENT PROTEIN KINASE II GAMMA	AAV	0.27
1X0X	CO-STRUCTURE OF HOMO SAPIENS GLYCEROL-3-PHOSPHATE DEHYDROGENASE 1 COMPLEX WITH NAD	AAV	0.27
2OYC	CRYSTAL STRUCTURE OF HUMAN PYRIDOXAL PHOSPHATE PHOSPHATASE	AAA	0.28
1J72	CRYSTAL STRUCTURE OF MUTANT MACROPHAGE CAPPING PROTEIN (CAP G) WITH ACTIN-SEVERING ACTIVITY IN THE CA2+-FREE FORM	AAA	0.28
2OYC	CRYSTAL STRUCTURE OF HUMAN PYRIDOXAL PHOSPHATE PHOSPHATASE	AAV	0.29
2UUI	CRYSTAL STRUCTURE OF HUMAN LEUKOTRIENE C4 SYNTHASE	AAV	0.29
3COU	CRYSTAL STRUCTURE OF HUMAN NUDIX MOTIF 16 (NUDT16)	AAA	0.29
2HRB	CRYSTAL STRUCTURE OF HUMAN CARBONYL REDUCTASE 3_ COMPLEXED WITH NADP+	AAV	0.29
2GF9	CRYSTAL STRUCTURE OF HUMAN RAB3D IN COMPLEX WITH GDP	AAV	0.30
1JTV	CRYSTAL STRUCTURE OF 17BETA-HYDROXYSTEROID DEHYDROGENASE TYPE 1 COMPLEXED WITH TESTOSTERONE	AAA	0.30
1WL4	HUMAN CYTOSOLIC ACETOACETYL-COA THIOLASE COMPLEXED WITH COA	AAV	0.30
3CTZ	STRUCTURE OF HUMAN CYTOSOLIC X-PROLYL AMINOPEPTIDASE	AAV	0.31
1ZED	ALKALINE PHOSPHATASE FROM HUMAN PLACENTA IN COMPLEX WITH P-NITROPHENYL-PHOSPHONATE	AAA	0.31
2OKK	THE X-RAY CRYSTAL STRUCTURE OF THE 65KDA ISOFORM OF GLUTAMIC ACID DECARBOXYLASE (GAD65)	AAA	0.31
2E8A	CRYSTAL STRUCTURE OF THE HUMAN HSP70 ATPASE DOMAIN IN COMPLEX WITH AMP-PNP	AAA	0.31
3BCH	CRYSTAL STRUCTURE OF THE HUMAN LAMININ RECEPTOR PRECURSOR	AAA	0.34
1ZJH	STRUCTURE OF HUMAN MUSCLE PYRUVATE KINASE (PKM2)	AAV	0.34
1SEN	ENDOPLASMIC RETICULUM PROTEIN RP19 O95881	AAA	0.34

2OEW	STRUCTURE OF ALIX/AIP1 BRO1 DOMAIN	AAA	0.34
1SK4	CRYSTAL STRUCTURE OF THE C-TERMINAL PEPTIDOGLYCAN-BINDING DOMAIN OF HUMAN PEPTIDOGLYCAN RECOGNITION PROTEIN IALPHA	AAA	0.35
2ALD	HUMAN MUSCLE ALDOLASE	AAA	0.37
1YZQ	GPPNHP-BOUND RAB6 GTPASE	AAA	0.37
1BJ4	RECOMBINANT SERINE HYDROXYMETHYLTRANSFERASE (HUMAN)	AAV	0.37
2G3Y	CRYSTAL STRUCTURE OF THE HUMAN SMALL GTPASE GEM	AAV	0.37
1IMJ	CRYSTAL STRUCTURE OF THE HUMAN CCG1/TAFII250-INTERACTING FACTOR B (CIB)	AAA	0.38
1R2D	STRUCTURE OF HUMAN BCL-XL AT 1.95 ANGSTROMS	AAV	0.38
3COU	CRYSTAL STRUCTURE OF HUMAN NUDIX MOTIF 16 (NUDT16)	AAA	0.39
2RK3	STRUCTURE OF A104T DJ-1	AAV	0.39
1R4X	CRYSTAL STRUCTURE ANALYS OF THE GAMMA-COPI APPENDAGE DOMAIN	AAV	0.40
1Z8D	CRYSTAL STRUCTURE OF HUMAN MUSCLE GLYCOGEN PHOSPHORYLASE A WITH AMP AND GLUCOSE	AAA	0.40
1QNT	X-RAY STRUCTURE OF HUMAN O6ALKYLGUANINE-DNA ALKYLTRANSFERAS	AAV	0.41
2EBC	MECHANISM UNDERLYING THE CRITICAL CONTRIBUTION OF A SWITCH II RESIDUE IN A HETEROTRIMERIC G-PROTEIN ALPHA SUBUNIT DURING C. ELEGANS ASYMMETRIC CELL DIVISION	AAV	0.42
2OUD	CRYSTAL STRUCTURE OF THE CATALYTIC DOMAIN OF HUMAN MKP5	AAA	0.42
2QMJ	CRYSTRAL STRUCTURE OF THE N-TERMINAL SUBUNIT OF HUMAN MALTASE-GLUCOAMYLASE IN COMPLEX WITH ACARBOSE	AAA	0.43
1WAR	RECOMBINANT HUMAN PURPLE ACID PHOSPHATASE EXPRESSED IN PICHIA PASTORIS	AAA	0.45
1MEO	HUMAN GLYCINAMIDE RIBONUCLEOTIDE TRANSFORMYLASE AT PH 4.2	AAV	0.46
2P02	CRYSTAL STRUCTURE OF THE ALPHA SUBUNIT OF HUMAN S-ADENOSYLMETHIONINE SYNTHETASE 2	AAV	0.47
1LZJ	GLYCOSYLTRANSFERASE B + UDP + H ANTIGEN ACCEPTOR	AAV	0.49
2H44	CRYSTAL STRUCTURE OF PDE5A1 IN COMPLEX WITH ICARISID II	AAA	0.49
1TBF	CATALYTIC DOMAIN OF HUMAN PHOSPHODIESTERASE 5A IN COMPLEX WITH SILDENAFIL	AAA	0.49
1H6O	DIMERISATION DOMAIN FROM HUMAN TRF1	AAA	0.50
1ZSY	THE STRUCTURE OF HUMAN MITOCHONDRIAL 2-ENOYL THIOESTER REDUCTASE (CGI-63)	AAV	0.50
1JDN	CRYSTAL STRUCTURE OF HORMONE RECEPTOR	AAA	0.50
1R55	CRYSTAL STRUCTURE OF THE CATALYTIC DOMAIN OF HUMAN ADAM 33	AAA	0.51
1QMN	ALPHA1-ANTICHYMOTRYPSIN SERPIN IN THE DELTA CONFORMATION (PARTIAL LOOP INSERTION)	AAA	0.51
2DH2	CRYSTAL STRUCTURE OF HUMAN ED-4F2HC	AAA	0.51
1OXZ	CRYSTAL STRUCTURE OF THE HUMAN GGA1 GAT DOMAIN	AAA	0.52
1WB0	SPECIFICITY AND AFFINITY OF NATURAL PRODUCT CYCLOPENTAPEPTIDE INHIBITOR ARGIFIN AGAINST HUMAN CHITINAS	AAA	0.53
1E2S	CRYSTAL STRUCTURE OF AN ARYLSULFATASE A MUTANT C69A	AAV	0.55
2FY2	STRUCTURES OF LIGAND BOUND HUMAN CHOLINE ACETYLTRANSFERASE PROVIDE INSIGHT INTO REGULATION OF ACETYLCHOLINE SYNTHESIS	AAV	0.56
3C8X	CRYSTAL STRUCTURE OF THE LIGAND BINDING DOMAIN OF HUMAN EPHRIN A2 (EPHA2) RECEPTOR PROTEIN KINASE	AAA	0.56
1DHS	CRYSTAL STRUCTURE OF THE NAD COMPLEX OF HUMAN DEOXYHYPUSINE SYNTHASE	AAV	0.56
1R55	CRYSTAL STRUCTURE OF THE CATALYTIC DOMAIN OF HUMAN ADAM 33	AAA	0.56
1Z70	1.15A RESOLUTION STRUCTURE OF THE FORMYLGLYCINE GENERATING ENZYME FGE	AAA	0.59
2H44	CRYSTAL STRUCTURE OF PDE5A1 IN COMPLEX WITH ICARISID II	AAV	0.63
1TBF	CATALYTIC DOMAIN OF HUMAN PHOSPHODIESTERASE 5A IN COMPLEX WITH SILDENAFIL	AAV	0.63
1N5U	X-RAY STUDY OF HUMAN SERUM ALBUMIN COMPLEXED WITH HEME	AAA	0.65
1WDY	CRYSTAL STRUCTURE OF RIBONUCLEASE	AAV	0.75

2FY2	STRUCTURES OF LIGAND BOUND HUMAN CHOLINE ACETYLTRANSFERASE PROVIDE INSIGHT INTO REGULATION OF ACETYLCHOLINE SYNTHESIS	AAA	0.77
1H30	C-TERMINAL LG DOMAIN PAIR OF HUMAN GAS6	AAA	1.33
1BY7	HUMAN PLASMINOGEN ACTIVATOR INHIBITOR-2. LOOP (66-98) DELETION MUTANT	AAA	2.00
1FCG	ECTODOMAIN OF HUMAN FC GAMMA RECEPTOR_ FCGRIIA	AAA	2.00
1N7D	EXTRACELLULAR DOMAIN OF THE LDL RECEPTOR	AAV	2.00
1PME	STRUCTURE OF PENTA MUTANT HUMAN ERK2 MAP KINASE COMPLEXED WITH A SPECIFIC INHIBITOR OF HUMAN P38 MAP KINASE	AAA	2.00
1R2D	STRUCTURE OF HUMAN BCL-XL AT 1.95 ANGSTROMS	AAA	2.00
1SO8	ABETA-BOUND HUMAN ABAD STRUCTURE [ALSO KNOWN AS 3-HYDROXYACYL-COA DEHYDROGENASE TYPE II (TYPE II HADH)_ ENDOPLASMIC RETICULUM-ASSOCIATED AMYLOID BETA-PEPTIDE BINDING PROTEIN (ERAB)]	AAA	2.00
1SZ7	CRYSTAL STRUCTURE OF HUMAN BET3	AAA	2.00
1TA0	THREE-DIMENSIONAL STRUCTURE OF A RNA-POLYMERASE II BINDING PROTEIN WITH ASSOCIATED LIGAND.	AAA	2.00
1W1D	CRYSTAL STRUCTURE OF THE PDK1 PLECKSTRIN HOMOLOGY (PH) DOMAIN BOUND TO INOSITOL (1_3_4_5)-TETRAKISPHOSPHATE	AAV	2.00
1YGS	CRYSTAL STRUCTURE OF THE SMAD4 TUMOR SUPPRESSOR C-TERMINAL DOMAIN	AAV	2.00
1YGS	CRYSTAL STRUCTURE OF THE SMAD4 TUMOR SUPPRESSOR C-TERMINAL DOMAIN	AAA	2.00
1ZSQ	CRYSTAL STRUCTURE OF MTMR2 IN COMPLEX WITH PHOSPHATIDYLINOSITOL 3-PHOSPHATE	AAA	2.00
2D7I	CRSYTAL STRUCTURE OF PP-GALNAC-T10 WITH UDP_ GALNAC AND MN2	AAV	2.00
2QOL	HUMAN EPHA3 KINASE AND JUXTAMEMBRANE REGION_ Y596:Y602:S768G TRIPLE MUTANT	AAA	2.00
2UW2	CRYSTAL STRUCTURE OF HUMAN RIBONUCLEOTIDE REDUCTASE SUBUNIT R2	AAA	2.00
2V9R	FIRST AND SECOND IG DOMAINS FROM HUMAN ROBO1 (FORM 2)	AAA	2.00
2ZMD	CRYSTAL STRUCTURE OF HUMAN MPS1 CATALYTIC DOMAIN T686A MUTANT IN COMPLEX WITH SP600125 INHIBITOR	AAA	2.00
3C0I	CASK CAM-KINASE DOMAIN- 3'-AMP COMPLEX_ P212121 FORM	AAV	2.00
	**granzyme A**		
2A2K	CRYSTAL STRUCTURE OF AN ACTIVE SITE MUTANT_ C473S_ OF CDC25B PHOSPHATASE CATALYTIC DOMAIN	GPR	0.19
1F5N	HUMAN GUANYLATE BINDING PROTEIN-1 IN COMPLEX WITH THE GTP ANALOGUE_ GMPPNP.	GPR	0.19
1C25	HUMAN CDC25A CATALYTIC DOMAIN	GPR	0.21
1IAT	CRYSTAL STRUCTURE OF HUMAN PHOSPHOGLUCOSE ISOMERASE/NEUROLEUKIN/AUTOCRINE MOTILITY FACTOR/MATURATION FACTOR	GPR	0.27
1KT0	STRUCTURE OF THE LARGE FKBP-LIKE PROTEIN_ FKBP51_ INVOLVED IN STEROID RECEPTOR COMPLEXES	GPR	0.28
2B69	CRYSTAL STRUCTURE OF HUMAN UDP-GLUCORONIC ACID DECARBOXYLAS	GPR	0.28
2JHM	STRUCTURE OF GLOBULAR HEADS OF M-FICOLIN AT NEUTRAL PH	GPR	0.29
2Z6O	CRYSTAL STRUCTURE OF THE UFC1_ UFM1 CONJUGATING ENZYME 1	GPR	0.30
1KO9	NATIVE STRUCTURE OF THE HUMAN 8-OXOGUANINE DNA GLYCOSYLASE HOGG1	GPR	0.30
1TQN	CRYSTAL STRUCTURE OF HUMAN MICROSOMAL P450 3A4	GPR	0.34
1ZVD	REGULATION OF SMURF2 UBIQUITIN LIGASE ACTIVITY BY ANCHORING THE E2 TO THE HECT DOMAIN	GPR	0.34
2HI4	CRYSTAL STRUCTURE OF HUMAN MICROSOMAL P450 1A2 IN COMPLEX WITH ALPHA-NAPHTHOFLAVONE	GPR	0.35
1D2S	CRYSTAL STRUCTURE OF THE N-TERMINAL LAMININ G-LIKE DOMAIN OF SHBG IN COMPLEX WITH DIHYDROTESTOSTERONE	GPR	0.38

2B69	CRYSTAL STRUCTURE OF HUMAN UDP-GLUCORONIC ACID DECARBOXYLAS	GPR	0.40
1N26	CRYSTAL STRUCTURE OF THE EXTRA-CELLULAR DOMAINS OF HUMAN INTERLEUKIN-6 RECEPTOR ALPHA CHAIN	GPR	0.41
1S31	CRYSTAL STRUCTURE ANALYSIS OF THE HUMAN TUB PROTEIN (ISOFORM A) SPANNING RESIDUES 289 THROUGH 561	GPR	0.44
1HDO	HUMAN BILIVERDIN IX BETA REDUCTASE: NADP COMPLEX	GPR	0.45
3CHO	CRYSTAL STRUCTURE OF LEUKOTRIENE A4 HYDROLASE IN COMPLEX WITH 2-AMINO-N-[4-(PHENYLMETHOXY)PHENYL]-ACETAMIDE	GPR	0.48
1CB0	STRUCTURE OF HUMAN 5'-DEOXY-5'-METHYLTHIOADENOSINE PHOSPHORYLASE AT 1.7 A RESOLUTION	GPR	0.50
1Q33	CRYSTAL STRUCTURE OF HUMAN ADP-RIBOSE PYROPHOSPHATASE NUDT9	GPR	0.53
1OZ2	CRYSTAL STRUCTURE OF 3-MBT REPEATS OF LETHAL (3) MALIGNANT BRAIN TUMOR (NATIVE-II) AT 1.55 ANGSTROM	GPR	0.54
1TDH	CRYSTAL STRUCTURE OF HUMAN ENDONUCLEASE VIII-LIKE 1 (NEIL1)	GPR	0.56
1PME	STRUCTURE OF PENTA MUTANT HUMAN ERK2 MAP KINASE COMPLEXED WITH A SPECIFIC INHIBITOR OF HUMAN P38 MAP KINASE	GPR	0.60
1PI1	CRYSTAL STRUCTURE OF A HUMAN MOB1 PROTEIN; TOWARD UNDERSTANDING MOB-REGULATED CELL CYCLE PATHWAYS.	GPR	0.61
1VZO	THE STRUCTURE OF THE N-TERMINAL KINASE DOMAIN OF MSK1 REVEALS A NOVEL AUTOINHIBITORY CONFORMATION FOR A DUAL KINASE PROTEIN	GPR	0.62
3COU	CRYSTAL STRUCTURE OF HUMAN NUDIX MOTIF 16 (NUDT16)	GPR	0.73
1CZA	MUTANT MONOMER OF RECOMBINANT HUMAN HEXOKINASE TYPE I COMPLEXED WITH GLUCOSE_ GLUCOSE-6-PHOSPHATE_ AND ADP	GPR	0.74
2VPJ	CRYSTAL STRUCTURE OF THE KELCH DOMAIN OF HUMAN KLHL12	GPR	2.00
	**granzyme B**		
2O72	CRYSTAL STRUCTURE ANALYSIS OF HUMAN E-CADHERIN (1-213)	AAD	0.09
1JDW	CRYSTAL STRUCTURE AND MECHANISM OF L-ARGININE: GLYCINE AMIDINOTRANSFERASE: A MITOCHONDRIAL ENZYME INVOLVED IN CREATINE BIOSYNTHESIS	AAD	0.11
2QYM	CRYSTAL STRUCTURE OF UNLIGANDED PDE4C2	AAD	0.11
2QYL	CRYSTAL STRUCTURE OF PDE4B2B IN COMPLEX WITH INHIBITOR NPV	AAD	0.11
1Z8D	CRYSTAL STRUCTURE OF HUMAN MUSCLE GLYCOGEN PHOSPHORYLASE A WITH AMP AND GLUCOSE	AAD	0.12
1TAZ	CATALYTIC DOMAIN OF HUMAN PHOSPHODIESTERASE 1B	AAD	0.12
1ZKL	MULTIPLE DETERMINANTS FOR INHIBITOR SELECTIVITY OF CYCLIC NUCLEOTIDE PHOSPHODIESTERASES	AAD	0.13
2IQ1	CRYSTAL STRUCTURE OF HUMAN PPM1K	AAD	0.14
2OO8	SYNTHESIS_ STRUCTURAL ANALYSIS_ AND SAR STUDIES OF TRIAZINE DERIVATIVES AS POTENT_ SELECTIVE TIE-2 INHIBITORS	AAD	0.14
1H30	C-TERMINAL LG DOMAIN PAIR OF HUMAN GAS6	AAD	0.15
2OEW	STRUCTURE OF ALIX/AIP1 BRO1 DOMAIN	AAD	0.16
1Q33	CRYSTAL STRUCTURE OF HUMAN ADP-RIBOSE PYROPHOSPHATASE NUDT9	AAD	0.16
1TAZ	CATALYTIC DOMAIN OF HUMAN PHOSPHODIESTERASE 1B	AAD	0.17
1TXD	CRYSTAL STRUCTURE OF THE DH/PH DOMAINS OF LEUKEMIA-ASSOCIATED RHOGEF	AAD	0.18
2ALD	HUMAN MUSCLE ALDOLASE	AAD	0.18
2V40	HUMAN ADENYLOSUCCINATE SYNTHETASE ISOZYME 2 IN COMPLEX WITH GDP	AAD	0.19
1F6W	STRUCTURE OF THE CATALYTIC DOMAIN OF HUMAN BILE SALT ACTIVATED LIPASE	AAD	0.20
2E0T	CRYSTAL STRUCTURE OF CATALYTIC DOMAIN OF DUAL SPECIFICITY PHOSPHATASE 26_ MS0830 FROM HOMO SAPIENS	AAD	0.21
2CZK	CRYSTAL STRUCTURE OF HUMAN MYO-INOSITOL MONOPHOSPHATASE 2 (IMPA2) (TRIGONAL FORM)	AAD	0.21
3COI	CRYSTAL STRUCTURE OF P38DELTA KINASE	AAD	0.22

2OKK	THE X-RAY CRYSTAL STRUCTURE OF THE 65KDA ISOFORM OF GLUTAMIC ACID DECARBOXYLASE (GAD65)	AAD	0.22
1Z70	1.15A RESOLUTION STRUCTURE OF THE FORMYLGLYCINE GENERATING ENZYME FGE	AAD	0.24
1ZD9	STRUCTURE OF HUMAN ADP-RIBOSYLATION FACTOR-LIKE 10B	AAD	0.24
2QQ5	CRYSTAL STRUCTURE OF HUMAN SDR FAMILY MEMBER 1	AAD	0.26
1HH8	THE ACTIVE N-TERMINAL REGION OF P67PHOX: STRUCTURE AT 1.8 ANGSTROM RESOLUTION AND BIOCHEMICAL CHARACTERIZATIONS OF THE A128V MUTANT IMPLICATED IN CHRONIC GRANULOMATOUS DISEASE	AAD	0.27
3CTZ	STRUCTURE OF HUMAN CYTOSOLIC X-PROLYL AMINOPEPTIDASE	AAD	0.27
1VJY	CRYSTAL STRUCTURE OF A NAPHTHYRIDINE INHIBITOR OF HUMAN TGF BETA TYPE I RECEPTOR	AAD	0.29
1WRM	CRYSTAL STRUCTURE OF JSP-1	AAD	0.31
1LBD	LIGAND-BINDING DOMAIN OF THE HUMAN NUCLEAR RECEPTOR RXR-ALPHA	AAD	0.33
1LCY	CRYSTAL STRUCTURE OF THE MITOCHONDRIAL SERINE PROTEASE HTRA	AAD	0.34
1P49	STRUCTURE OF HUMAN PLACENTAL ESTRONE/DHEA SULFATASE	AAD	0.35
1HDO	HUMAN BILIVERDIN IX BETA REDUCTASE: NADP COMPLEX	AAD	0.35
2QTZ	CRYSTAL STRUCTURE OF THE NADP+-BOUND FAD-CONTAINING FNR- LIKE MODULE OF HUMAN METHIONINE SYNTHASE REDUCTASE	AAD	0.35
1TAZ	CATALYTIC DOMAIN OF HUMAN PHOSPHODIESTERASE 1B	AAD	0.36
2JC9	CRYSTAL STRUCTURE OF HUMAN CYTOSOLIC 5'-NUCLEOTIDASE II IN COMPLEX WITH ADENOSINE	AAD	0.37
2O8T	CRYSTAL STRUCTURE AND BINDING EPITOPES OF UROKINASE-TYPE PLASMINOGEN ACTIVATOR (C122A/N145Q) IN COMPLEX WITH INHIBITORS	AAD	0.38
2OIT	CRYSTAL STRUCTURE OF THE N-TERMINAL DOMAIN OF THE HUMAN PROTO-ONCOGENE NUP214/CAN	AAD	0.41
1N5U	X-RAY STUDY OF HUMAN SERUM ALBUMIN COMPLEXED WITH HEME	AAD	0.42
1LF7	CRYSTAL STRUCTURE OF HUMAN COMPLEMENT PROTEIN C8GAMMA AT 1.2 A RESOLUTION	AAD	0.45
1DT9	THE CRYSTAL STRUCTURE OF HUMAN EUKARYOTIC RELEASE FACTOR ERF1-MECHANISM OF STOP CODON RECOGNITION AND PEPTIDYL-TRNA HYDROLYSIS	AAD	0.46
2VAF	CRYSTAL STRUCTURE OF HUMAN CARDIAC CALSEQUESTRIN	AAD	0.47
2B58	SSAT WITH COA_SP_ SPERMINE DISORDERED_ K26R MUTANT	AAD	0.50
1TFF	STRUCTURE OF OTUBAIN-2	AAD	0.50
2GJK	STRUCTURAL AND FUNCTIONAL INSIGHTS INTO THE HUMAN UPF1 HELICASE CORE	AAD	0.52
2QLU	CRYSTAL STRUCTURE OF ACTIVIN RECEPTOR TYPE II KINASE DOMAIN FROM HUMAN	AAD	0.53
1WOJ	CRYSTAL STRUCTURE OF HUMAN PHOSPHODIESTERASE	AAD	0.53
2QCF	CRYSTAL STRUCTURE OF THE OROTIDINE-5'-MONOPHOSPHATE DECARBOXYLASE DOMAIN (ASP312ASN MUTANT) OF HUMAN UMP SYNTHASE BOUND TO 5-FLUORO-UMP	AAD	0.59
1HTJ	STRUCTURE OF THE RGS-LIKE DOMAIN FROM PDZ-RHOGEF	AAD	0.59
1N5U	X-RAY STUDY OF HUMAN SERUM ALBUMIN COMPLEXED WITH HEME	AAD	0.68
1N5U	X-RAY STUDY OF HUMAN SERUM ALBUMIN COMPLEXED WITH HEME	AAD	0.69
1BY7	HUMAN PLASMINOGEN ACTIVATOR INHIBITOR-2. LOOP (66-98) DELETION MUTANT	AAD	0.72
1BIO	HUMAN COMPLEMENT FACTOR D IN COMPLEX WITH ISATOIC ANHYDRIDE INHIBITOR	AAD	2.00
1QNT	X-RAY STRUCTURE OF HUMAN O6ALKYLGUANINE-DNA ALKYLTRANSFERAS	AAD	2.00
2OJ9	STRUCTURE OF IGF-1R KINASE DOMAIN COMPLEXED WITH A BENZIMIDAZOLE INHIBITOR	AAD	2.00
	**granzyme H**		

1ZED	ALKALINE PHOSPHATASE FROM HUMAN PLACENTA IN COMPLEX WITH P-NITROPHENYL-PHOSPHONATE	FLF	0.05
1GEN	C-TERMINAL DOMAIN OF GELATINASE A	AAF	0.06
3BER	HUMAN DEAD-BOX RNA-HELICASE DDX47_ CONSERVED DOMAIN I IN COMPLEX WITH AMP	FLF	0.06
2UXW	CRYSTAL STRUCTURE OF HUMAN VERY LONG CHAIN ACYL-COA DEHYDROGENASE (ACADVL)	AAF	0.07
2DW4	CRYSTAL STRUCTURE OF HUMAN LSD1 AT 2.3 A RESOLUTION	FLF	0.07
2ICA	CD11A (LFA1) I-DOMAIN COMPLEXED WITH BMS-587101 AKA 5-[(5S_ 9R)-9-(4-CYANOPHENYL)-3-(3_5-DICHLOROPHENYL)-1-METHYL-2_4- DIOXO-1_3_7-TRIAZASPIRO [4.4]NON-7-YL]METHYL]-3- THIOPHENECARBOXYLICACID	FLF	0.07
2HI4	CRYSTAL STRUCTURE OF HUMAN MICROSOMAL P450 1A2 IN COMPLEX WITH ALPHA-NAPHTHOFLAVONE	FLF	0.07
1SO7	MALTOSE-INDUCED STRUCTURE OF THE HUMAN CYTOLSOLIC SIALIDASE NEU2	FLF	0.07
1CZA	MUTANT MONOMER OF RECOMBINANT HUMAN HEXOKINASE TYPE I COMPLEXED WITH GLUCOSE_ GLUCOSE-6-PHOSPHATE_ AND ADP	AAM	0.07
2B3X	STRUCTURE OF AN ORTHORHOMBIC CRYSTAL FORM OF HUMAN CYTOSOLIC ACONITASE (IRP1)	AAF	0.07
1M6I	CRYSTAL STRUCTURE OF APOPTOSIS INDUCING FACTOR (AIF)	AAF	0.07
1P49	STRUCTURE OF HUMAN PLACENTAL ESTRONE/DHEA SULFATASE	AAF	0.07
2PZ1	CRYSTAL STRUCTURE OF AUTO-INHIBITED ASEF	FLF	0.08
1T32	A DUAL INHIBITOR OF THE LEUKOCYTE PROTEASES CATHEPSIN G AND CHYMASE WITH THERAPEUTIC EFFICACY IN ANIMALS MODELS OF INFLAMMATION	AAF	0.08
2I7V	STRUCTURE OF HUMAN CPSF-73	AAM	0.08
1PEX	COLLAGENASE-3 (MMP-13) C-TERMINAL HEMOPEXIN-LIKE DOMAIN	AAY	0.08
2NNJ	CYP2C8DH COMPLEXED WITH FELODIPINE	FLF	0.08
1R9O	CRYSTAL STRUCTURE OF P4502C9 WITH FLURBIPROFEN BOUND	FLF	0.08
1BY7	HUMAN PLASMINOGEN ACTIVATOR INHIBITOR-2. LOOP (66-98) DELETION MUTANT	FLF	0.08
1E8Y	STRUCTURE DETERMINANTS OF PHOSPHOINOSITIDE 3-KINASE INHIBITION BY WORTMANNIN_ LY294002_ QUERCETIN_ MYRICETIN AND STAUROSPORINE	FLF	0.08
1NTY	CRYSTAL STRUCTURE OF THE FIRST DH/PH DOMAIN OF TRIO TO 1.7	FLF	0.08
2DHO	CRYSTAL STRUCTURE OF HUMAN IPP ISOMERASE I IN SPACE GROUP P212121	FLF	0.09
1KHB	PEPCK COMPLEX WITH NONHYDROLYZABLE GTP ANALOG_ NATIVE DATA	AAF	0.09
2OU2	ACETYLTRANSFERASE DOMAIN OF HUMAN HIV-1 TAT INTERACTING PROTEIN_ 60KDA_ ISOFORM 3	FLF	0.09
2OAY	CRYSTAL STRUCTURE OF LATENT HUMAN C1-INHIBITOR	FLF	0.09
2OUD	CRYSTAL STRUCTURE OF THE CATALYTIC DOMAIN OF HUMAN MKP5	FLF	0.09
1ZXQ	THE CRYSTAL STRUCTURE OF ICAM-2	FLF	0.09
2R0B	CRYSTAL STRUCTURE OF HUMAN TYROSINE PHOSPHATASE-LIKE SERINE/THREONINE/TYROSINE-INTERACTING PROTEIN	AAF	0.10
2OZU	CRYSTAL STRUCTURE OF HUMAN MYST HISTONE ACETYLTRANSFERASE 3 IN COMPLEX WITH ACETYLCOENZYME A	FLF	0.10
2J5W	CERULOPLASMIN REVISITED: STRUCTURAL AND FUNCTIONAL ROLES OF VARIOUS METAL CATION BINDING SITES	AAF	0.10
1P5J	CRYSTAL STRUCTURE ANALYSIS OF HUMAN SERINE DEHYDRATASE	AAY	0.10
2OBD	CRYSTAL STRUCTURE OF CHOLESTERYL ESTER TRANSFER PROTEIN	FLF	0.10
2GF9	CRYSTAL STRUCTURE OF HUMAN RAB3D IN COMPLEX WITH GDP	FLF	0.10
2O36	CRYSTAL STRUCTURE OF ENGINEERED THIMET OLIGOPEPTIDASE WITH NEUROLYSIN SPECIFICITY IN NEUROTENSIN CLEAVAGE SITE	AAM	0.10
2QIS	CRYSTAL STRUCTURE OF HUMAN FARNESYL PYROPHOSPHATE SYNTHASE T210S MUTANT BOUND TO RISEDRONATE	AAM	0.10
1LJ5	1.8A RESOLUTION STRUCTURE OF LATENT PLASMINOGEN ACTIVATOR INHIBITOR-1(PAI-1)	FLF	0.11

2P02	CRYSTAL STRUCTURE OF THE ALPHA SUBUNIT OF HUMAN S-ADENOSYLMETHIONINE SYNTHETASE 2	AAY	0.11
2PPL	HUMAN PANCREATIC LIPASE-RELATED PROTEIN 1	AAY	0.11
2OBV	CRYSTAL STRUCTURE OF THE HUMAN S-ADENOSYLMETHIONINE SYNTHETASE 1 IN COMPLEX WITH THE PRODUCT	AAY	0.11
3CKK	CRYSTAL STRUCTURE OF HUMAN METHYLTRANSFERASE-LIKE PROTEIN 1	FLF	0.11
1E8Y	STRUCTURE DETERMINANTS OF PHOSPHOINOSITIDE 3-KINASE INHIBITION BY WORTMANNIN_ LY294002_ QUERCETIN_ MYRICETIN AND STAUROSPORINE	FLF	0.11
1XQJ	3.10 A CRYSTAL STRUCTURE OF MASPIN_ SPACE GROUP I 4 2 2	AAY	0.11
2DW4	CRYSTAL STRUCTURE OF HUMAN LSD1 AT 2.3 A RESOLUTION	FLF	0.12
2C9H	STRUCTURE OF MITOCHONDRIAL BETA-KETOACYL SYNTHASE	AAF	0.12
1P49	STRUCTURE OF HUMAN PLACENTAL ESTRONE/DHEA SULFATASE	FLF	0.12
1OHC	STRUCTURE OF THE PROLINE DIRECTED PHOSPHATASE CDC14	AAF	0.12
2B3X	STRUCTURE OF AN ORTHORHOMBIC CRYSTAL FORM OF HUMAN CYTOSOLIC ACONITASE (IRP1)	AAM	0.12
2BW0	CRYSTAL STRUCTURE OF THE HYDROLASE DOMAIN OF HUMAN 10-FORMYLTETRAHYDROFOLATE 2 DEHYDROGENASE	FLF	0.12
2FOZ	HUMAN ADP-RIBOSYLHYDROLASE 3	AAF	0.12
1CS8	CRYSTAL STRUCTURE OF PROCATHEPSIN L	FLF	0.13
3CTZ	STRUCTURE OF HUMAN CYTOSOLIC X-PROLYL AMINOPEPTIDASE	AAM	0.13
2DW4	CRYSTAL STRUCTURE OF HUMAN LSD1 AT 2.3 A RESOLUTION	AAF	0.13
1Q20	CRYSTAL STRUCTURE OF HUMAN CHOLESTEROL SULFOTRANSFERASE (SULT2B1B) IN THE PRESENCE OF PAP AND PREGNENOLONE	FLF	0.13
2C9H	STRUCTURE OF MITOCHONDRIAL BETA-KETOACYL SYNTHASE	AAY	0.13
2G62	CRYSTAL STRUCTURE OF HUMAN PTPA	AAF	0.13
1M13	CRYSTAL STRUCTURE OF THE HUMAN PREGANE X RECEPTOR LIGAND BINDING DOMAIN IN COMPLEX WITH HYPERFORIN_ A CONSTITUENT OF ST. JOHN'S WORT	AAF	0.13
2G62	CRYSTAL STRUCTURE OF HUMAN PTPA	AAF	0.13
1CZA	MUTANT MONOMER OF RECOMBINANT HUMAN HEXOKINASE TYPE I COMPLEXED WITH GLUCOSE_ GLUCOSE-6-PHOSPHATE_ AND ADP	FLF	0.13
2E9L	CRYSTAL STRUCTURE OF HUMAN CYTOSOLIC NEUTRAL BETA-GLYCOSYLCERAMIDASE (KLOTHO-RELATED PROTE:KLRP) COMPLEX WITH GLUCOSE AND FATTY ACIDS	AAY	0.13
1P49	STRUCTURE OF HUMAN PLACENTAL ESTRONE/DHEA SULFATASE	FLF	0.14
1Z8D	CRYSTAL STRUCTURE OF HUMAN MUSCLE GLYCOGEN PHOSPHORYLASE A WITH AMP AND GLUCOSE	AAY	0.14
1FA9	HUMAN LIVER GLYCOGEN PHOSPHORYLASE A COMPLEXED WITH AMP	AAY	0.14
2RJQ	CRYSTAL STRUCTURE OF ADAMTS5 WITH INHIBITOR BOUND	AAF	0.14
1KHB	PEPCK COMPLEX WITH NONHYDROLYZABLE GTP ANALOG_ NATIVE DATA	AAM	0.14
2I4I	CRYSTAL STRUCTURE OF HUMAN DEAD-BOX RNA HELICASE DDX3X	AAF	0.14
1KT0	STRUCTURE OF THE LARGE FKBP-LIKE PROTEIN_ FKBP51_ INVOLVED IN STEROID RECEPTOR COMPLEXES	AAF	0.15
2F71	PROTEIN TYROSINE PHOSPHATASE 1B WITH SULFAMIC ACID INHIBITORS	FLF	0.15
3BI1	X-RAY STRUCTURE OF HUMAN GLUTAMATE CARBOXYPEPTIDASE II (GCPII) IN COMPLEX WITH A TRANSITION STATE ANALOG OF METHOTREXATE-GLU	AAF	0.15
2C43	STRUCTURE OF AMINOADIPATE-SEMIALDEHYDE DEHYDROGENASE-PHOSPHOPANTETHEINYL TRANSFERASE IN COMPLEX WITH COENZYME A	AAM	0.15
1ORE	HUMAN ADENINE PHOSPHORIBOSYLTRANSFERASE	FLF	0.16
2FST	MITOGEN ACTIVATED PROTEIN KINASE P38ALPHA (D176A+F327L) ACTIVATING MUTANT	AAF	0.16
1XWI	CRYSTAL STRUCTURE OF VPS4B	AAM	0.16
3GRS	REFINED STRUCTURE OF GLUTATHIONE REDUCTASE AT 1.54 ANGSTROMS RESOLUTION	AAF	0.16
2B1P	INHIBITOR COMPLEX OF JNK3	AAY	0.17

1IAT	CRYSTAL STRUCTURE OF HUMAN PHOSPHOGLUCOSE ISOMERASE/NEUROLEUKIN/AUTOCRINE MOTILITY FACTOR/MATURATION FACTOR	AAY	0.17
1US0	HUMAN ALDOSE REDUCTASE IN COMPLEX WITH NADP+ AND THE INHIBITOR IDD594 AT 0.66 ANGSTROM	AAM	0.17
2P02	CRYSTAL STRUCTURE OF THE ALPHA SUBUNIT OF HUMAN S-ADENOSYLMETHIONINE SYNTHETASE 2	AAY	0.17
1XTQ	STRUCTURE OF SMALL GTPASE HUMAN RHEB IN COMPLEX WITH GDP	AAF	0.17
2V5O	STRUCTURE OF HUMAN IGF2R DOMAINS 11-14	AAY	0.17
1L8K	T CELL PROTEIN-TYROSINE PHOSPHATASE STRUCTURE	FLF	0.18
2GFO	STRUCTURE OF THE CATALYTIC DOMAIN OF HUMAN UBIQUITIN CARBOXYL-TERMINAL HYDROLASE 8	AAY	0.18
2BMD	HIGH RESOLUTION STRUCTURE OF GDP-BOUND HUMAN RAB4A	FLF	0.18
2EW1	CRYSTAL STRUCTURE OF RAB30 IN COMPLEX WITH A GTP ANALOGUE	FLF	0.18
1OZN	1.5A CRYSTAL STRUCTURE OF THE NOGO RECEPTOR LIGAND BINDING DOMAIN REVEALS A CONVERGENT RECOGNITION SCAFFOLD MEDIATING INHIBITION OF MYELINATION	AAF	0.19
1HVF	STRUCTURAL AND ELECTROPHYSIOLOGICAL ANALYSIS OF ANNEXIN V MUTANTS. MUTAGENESIS OF HUMAN ANNEXIN V_ AN IN VITRO VOLTAGE-GATED CALCIUM CHANNEL_ PROVIDES INFORMATION ABOUT THE STRUCTURAL FEATURES OF THE ION PATHWAY_ THE VOLTAGE SENSOR AND THE ION SELECTIVITY FILTER	AAF	0.19
1HDO	HUMAN BILIVERDIN IX BETA REDUCTASE: NADP COMPLEX	AAM	0.19
1ZKL	MULTIPLE DETERMINANTS FOR INHIBITOR SELECTIVITY OF CYCLIC NUCLEOTIDE PHOSPHODIESTERASES	FLF	0.20
1FEW	CRYSTAL STRUCTURE OF SMAC/DIABLO	AAY	0.20
2ILT	HUMAN 11-BETA-HYDROXYSTEROID DEHYDROGENASE (HSD1) WITH NADP AND ADAMANTANE SULFONE INHIBITOR	AAY	0.20
2REW	CRYSTAL STRUCTURE OF PPARALPHA LIGAND BINDING DOMAIN WITH BMS-631707	FLF	0.20
1M9I	CRYSTAL STRUCTURE OF PHOSPHORYLATION-MIMICKINGMUTANT T356D OF ANNEXIN VI	AAY	0.20
1M13	CRYSTAL STRUCTURE OF THE HUMAN PREGANE X RECEPTOR LIGAND BINDING DOMAIN IN COMPLEX WITH HYPERFORIN_ A CONSTITUENT OF ST. JOHN'S WORT	FLF	0.21
2GW2	CRYSTAL STRUCTURE OF THE PEPTIDYL-PROLYL ISOMERASE DOMAIN OF HUMAN CYCLOPHILIN G	AAF	0.21
1V5H	CRYSTAL STRUCTURE OF HUMAN CYTOGLOBIN (FERRIC FORM)	AAY	0.21
3CHO	CRYSTAL STRUCTURE OF LEUKOTRIENE A4 HYDROLASE IN COMPLEX WITH 2-AMINO-N-[4-(PHENYLMETHOXY)PHENYL]-ACETAMIDE	AAF	0.22
1L3K	UP1_ THE TWO RNA-RECOGNITION MOTIF DOMAIN OF HNRNP A1	AAM	0.22
1F6W	STRUCTURE OF THE CATALYTIC DOMAIN OF HUMAN BILE SALT ACTIVATED LIPASE	AAF	0.22
2UUR	N-TERMINAL NC4 DOMAIN OF COLLAGEN IX	AAF	0.23
2DHO	CRYSTAL STRUCTURE OF HUMAN IPP ISOMERASE I IN SPACE GROUP P212121	FLF	0.23
2NZL	CRYSTAL STRUCTURE OF HUMAN HYDROXYACID OXIDASE 1	AAF	0.23
1H0C	THE CRYSTAL STRUCTURE OF HUMAN ALANINE:GLYOXYLATE AMINOTRANSFERASE	AAY	0.23
2OVJ	THE CRYSTAL STRUCTURE OF THE HUMAN RAC GTPASE ACTIVATING PROTEIN 1 (RACGAP1) MGCRACGAP.	AAM	0.23
2POM	TAB1 WITH MANGANESE ION	AAM	0.24
1OW1	CRYSTAL STRUCTURE OF THE SPOC DOMAIN OF THE HUMAN TRANSCRIPTIONAL COREPRESSOR_ SHARP.	AAF	0.24
2P02	CRYSTAL STRUCTURE OF THE ALPHA SUBUNIT OF HUMAN S-ADENOSYLMETHIONINE SYNTHETASE 2	FLF	0.24
2IQC	CRYSTAL STRUCTURE OF HUMAN FANCF PROTEIN THAT FUNCTIONS IN THE ASSEMBLY OF A DNA DAMAGE SIGNALING COMPLEX	AAF	0.24

1AIN	CRYSTAL STRUCTURE OF HUMAN ANNEXIN I AT 2.5 ANGSTROMS RESOLUTION	AAY	0.24
2HFT	THE CRYSTAL STRUCTURE OF THE EXTRACELLULAR DOMAIN OF HUMAN TISSUE FACTOR AT 1.7 ANGSTROMS RESOLUTION	AAY	0.25
2FFX	STRUCTURE OF HUMAN FERRITIN L. CHAIN	AAM	0.25
2IHD	CRYSTAL STRUCTURE OF HUMAN REGULATOR OF G-PROTEIN SIGNALING 8_ RGS8	AAF	0.25
1P0I	CRYSTAL STRUCTURE OF HUMAN BUTYRYL CHOLINESTERASE	AAF	0.25
1N5U	X-RAY STUDY OF HUMAN SERUM ALBUMIN COMPLEXED WITH HEME	AAF	0.26
1Z8D	CRYSTAL STRUCTURE OF HUMAN MUSCLE GLYCOGEN PHOSPHORYLASE A WITH AMP AND GLUCOSE	AAY	0.26
2C9Y	STRUCTURE OF HUMAN ADENYLATE KINASE 2	AAF	0.26
1AIN	CRYSTAL STRUCTURE OF HUMAN ANNEXIN I AT 2.5 ANGSTROMS RESOLUTION	AAM	0.26
1LJ5	1.8A RESOLUTION STRUCTURE OF LATENT PLASMINOGEN ACTIVATOR INHIBITOR-1(PAI-1)	AAM	0.27
2JHM	STRUCTURE OF GLOBULAR HEADS OF M-FICOLIN AT NEUTRAL PH	AAY	0.28
2EBC	MECHANISM UNDERLYING THE CRITICAL CONTRIBUTION OF A SWITCH II RESIDUE IN A HETEROTRIMERIC G-PROTEIN ALPHA SUBUNIT DURING C. ELEGANS ASYMMETRIC CELL DIVISION	AAY	0.28
1R2Q	CRYSTAL STRUCTURE OF HUMAN RAB5A GTPASE DOMAIN AT 1.05 A RESOLUTION	AAF	0.28
2R2P	KINASE DOMAIN OF HUMAN EPHRIN TYPE-A RECEPTOR 5 (EPHA5)	AAY	0.28
2FUE	HUMAN ALPHA-PHOSPHOMANNOMUTASE 1 WITH D-MANNOSE 1-PHOSPHATE AND MG2+ COFACTOR BOUND	AAF	0.29
1M8Z	CRYSTAL STRUCTURE OF A PUMILIO-HOMOLOGY DOMAIN	AAY	0.29
2DYL	CRYSTAL STRUCTURE OF HUMAN MITOGEN-ACTIVATED PROTEIN KINASE KINASE 7 ACTIVATED MUTANT (S287D_ T291D)	AAY	0.29
2EBC	MECHANISM UNDERLYING THE CRITICAL CONTRIBUTION OF A SWITCH II RESIDUE IN A HETEROTRIMERIC G-PROTEIN ALPHA SUBUNIT DURING C. ELEGANS ASYMMETRIC CELL DIVISION	AAY	0.30
1EEM	GLUTATHIONE TRANSFERASE FROM HOMO SAPIENS	AAM	0.30
1ZS9	CRYSTAL STRUCTURE OF HUMAN ENOLASE-PHOSPHATASE E1	AAF	0.30
1N5U	X-RAY STUDY OF HUMAN SERUM ALBUMIN COMPLEXED WITH HEME	AAF	0.31
1LI4	HUMAN S-ADENOSYLHOMOCYSTEINE HYDROLASE COMPLEXED WITH NEPLANOCIN	AAM	0.31
1N3L	CRYSTAL STRUCTURE OF A HUMAN AMINOACYL-TRNA SYNTHETASE CYTOKINE	AAY	0.32
1TDH	CRYSTAL STRUCTURE OF HUMAN ENDONUCLEASE VIII-LIKE 1 (NEIL1)	AAF	0.32
1OHC	STRUCTURE OF THE PROLINE DIRECTED PHOSPHATASE CDC14	AAY	0.33
1ZJK	CRYSTAL STRUCTURE OF THE ZYMOGEN CATALYTIC REGION OF HUMAN MASP-2	AAY	0.34
1R1H	STRUCTURAL ANALYSIS OF NEPRILYSIN WITH VARIOUS SPECIFIC AND POTENT INHIBITORS	AAF	0.35
2AA2	MINERALOCORTICOID RECEPTOR WITH BOUND ALDOSTERONE	AAF	0.35
2Z5Y	CRYSTAL STRUCTURE OF HUMAN MONOAMINE OXIDASE A (G110A) WITH HARMINE	AAF	0.36
1T46	STRUCTURAL BASIS FOR THE AUTOINHIBITION AND STI-571 INHIBITION OF C-KIT TYROSINE KINASE	AAM	0.37
2Z5J	FREE TRANSPORTIN 1	AAF	0.37
1H30	C-TERMINAL LG DOMAIN PAIR OF HUMAN GAS6	AAY	0.37
2NZL	CRYSTAL STRUCTURE OF HUMAN HYDROXYACID OXIDASE 1	AAY	0.38
2HGS	HUMAN GLUTATHIONE SYNTHETASE	AAF	0.39
1Z8D	CRYSTAL STRUCTURE OF HUMAN MUSCLE GLYCOGEN PHOSPHORYLASE A WITH AMP AND GLUCOSE	AAF	0.42
1BYG	KINASE DOMAIN OF HUMAN C-TERMINAL SRC KINASE (CSK) IN COMPLEX WITH INHIBITOR STAUROSPORINE	AAM	0.42
3COU	CRYSTAL STRUCTURE OF HUMAN NUDIX MOTIF 16 (NUDT16)	AAF	0.44

2HZP	CRYSTAL STRUCTURE OF HOMO SAPIENS KYNURENINASE	AAY	0.46
1AXN	THE HIGH RESOLUTION STRUCTURE OF ANNEXIN III SHOWS DIFFERENCES WITH ANNEXIN V	AAY	0.46
1SO8	ABETA-BOUND HUMAN ABAD STRUCTURE [ALSO KNOWN AS 3-HYDROXYACYL-COA DEHYDROGENASE TYPE II (TYPE II HADH)_ ENDOPLASMIC RETICULUM-ASSOCIATED AMYLOID BETA-PEPTIDE BINDING PROTEIN (ERAB)]	AAF	0.49
1SO8	ABETA-BOUND HUMAN ABAD STRUCTURE [ALSO KNOWN AS 3-HYDROXYACYL-COA DEHYDROGENASE TYPE II (TYPE II HADH)_ ENDOPLASMIC RETICULUM-ASSOCIATED AMYLOID BETA-PEPTIDE BINDING PROTEIN (ERAB)]	AAY	0.50
2DE0	CRYSTAL STRUCTURE OF HUMAN ALPHA 1_6-FUCOSYLTRANSFERASE_ FUT8	AAF	0.64
3BCH	CRYSTAL STRUCTURE OF THE HUMAN LAMININ RECEPTOR PRECURSOR	AAF	0.76
1HTJ	STRUCTURE OF THE RGS-LIKE DOMAIN FROM PDZ-RHOGEF	AAM	2.00
1NRG	STRUCTURE AND PROPERTIES OF RECOMBINANT HUMAN PYRIDOXINE-5' PHOSPHATE OXIDASE	AAM	2.00
1UWY	CRYSTAL STRUCTURE OF HUMAN CARBOXYPEPTIDASE M	FLF	2.00
1ZJH	STRUCTURE OF HUMAN MUSCLE PYRUVATE KINASE (PKM2)	AAM	2.00
1ZKL	MULTIPLE DETERMINANTS FOR INHIBITOR SELECTIVITY OF CYCLIC NUCLEOTIDE PHOSPHODIESTERASES	AAF	2.00
2ADP	NITRATED HUMAN MANGANESE SUPEROXIDE DISMUTASE	AAY	2.00
2J5W	CERULOPLASMIN REVISITED: STRUCTURAL AND FUNCTIONAL ROLES OF VARIOUS METAL CATION BINDING SITES	FLF	2.00
2Q0Z	CRYSTAL STRUCTURE OF Q9P172/SEC63 FROM HOMO SAPIENS. NORTHEAST STRUCTURAL GENOMICS TARGET HR1979.	AAY	2.00
2Q0Z	CRYSTAL STRUCTURE OF Q9P172/SEC63 FROM HOMO SAPIENS. NORTHEAST STRUCTURAL GENOMICS TARGET HR1979.	AAM	2.00
2QOL	HUMAN EPHA3 KINASE AND JUXTAMEMBRANE REGION_ Y596:Y602:S768G TRIPLE MUTANT	AAY	2.00
2UW2	CRYSTAL STRUCTURE OF HUMAN RIBONUCLEOTIDE REDUCTASE SUBUNIT R2	FLF	2.00
	**hepsin**		
2QQJ	CRYSTAL STRUCTURE OF THE B1B2 DOMAINS FROM HUMAN NEUROPILIN 2	FVR	0.12
2NR8	CRYSTAL STRUCTURE OF THE HUMAN KIF9 MOTOR DOMAIN IN COMPLEX WITH ADP	FVR	0.12
2B9E	HUMAN NSUN5 PROTEIN	FVR	0.12
2QQI	CRYSTAL STRUCTURE OF THE B1B2 DOMAINS FROM HUMAN NEUROPILIN 1	FVR	0.12
1SI5	PROTEASE-LIKE DOMAIN FROM 2-CHAIN HEPATOCYTE GROWTH FACTOR	FVR	0.12
2UXW	CRYSTAL STRUCTURE OF HUMAN VERY LONG CHAIN ACYL-COA DEHYDROGENASE (ACADVL)	LSR	0.12
2EC8	CRYSTAL STRUCTURE OF THE EXCTRACELLULAR DOMAIN OF THE RECEPTOR TYROSINE KINASE_ KIT	FVR	0.13
2QQJ	CRYSTAL STRUCTURE OF THE B1B2 DOMAINS FROM HUMAN NEUROPILIN 2	FVR	0.13
2HRB	CRYSTAL STRUCTURE OF HUMAN CARBONYL REDUCTASE 3_ COMPLEXED WITH NADP+	LSR	0.14
1WMA	CRYSTAL STRUCTURE OF HUMAN CBR1 IN COMPLEX WITH HYDROXY-PP	LSR	0.14
2HI4	CRYSTAL STRUCTURE OF HUMAN MICROSOMAL P450 1A2 IN COMPLEX WITH ALPHA-NAPHTHOFLAVONE	LSR	0.14
2VKQ	CRYSTAL STRUCTURE OF HUMAN CYTOSOLIC 5'-NUCLEOTIDASE III (CN-III_ NT5C3) IN COMPLEX WITH BERYLLIUM TRIFLUORIDE	LSR	0.15
2QMJ	CRYSTRAL STRUCTURE OF THE N-TERMINAL SUBUNIT OF HUMAN MALTASE-GLUCOAMYLASE IN COMPLEX WITH ACARBOSE	LSR	0.15
2I7V	STRUCTURE OF HUMAN CPSF-73	LSR	0.15
2BU7	CRYSTAL STRUCTURES OF HUMAN PYRUVATE DEHYDROGENASE KINASE 2 CONTAINING PHYSIOLOGICAL AND SYNTHETIC LIGANDS	LSR	0.16
2OIL	CRYSTAL STRUCTURE OF HUMAN RAB25 IN COMPLEX WITH GDP	LSR	0.16

2PPL	HUMAN PANCREATIC LIPASE-RELATED PROTEIN 1	LSR	0.16
1A6Q	CRYSTAL STRUCTURE OF THE PROTEIN SERINE/THREONINE PHOSPHATASE 2C AT 2 A RESOLUTION	FVR	0.16
2F9L	3D STRUCTURE OF INACTIVE HUMAN RAB11B GTPASE	LSR	0.16
1MD8	MONOMERIC STRUCTURE OF THE ACTIVE CATALYTIC DOMAIN OF COMPLEMENT PROTEASE C1R	FVR	0.17
1P0I	CRYSTAL STRUCTURE OF HUMAN BUTYRYL CHOLINESTERASE	LSR	0.17
1NUF	ROLE OF CALCIUM IONS IN THE ACTIVATION AND ACTIVITY OF THE TRANSGLUTAMINASE 3 ENZYME	FVR	0.17
2BH9	X-RAY STRUCTURE OF A DELETION VARIANT OF HUMAN GLUCOSE 6-PHOSPHATE DEHYDROGENASE COMPLEXED WITH STRUCTURAL AND COENZYME NADP	FVR	0.17
2FY2	STRUCTURES OF LIGAND BOUND HUMAN CHOLINE ACETYLTRANSFERASE PROVIDE INSIGHT INTO REGULATION OF ACETYLCHOLINE SYNTHESIS	FVR	0.18
2PZ1	CRYSTAL STRUCTURE OF AUTO-INHIBITED ASEF	FVR	0.18
2F1W	CRYSTAL STRUCTURE OF THE TRAF-LIKE DOMAIN OF HAUSP/USP7	FVR	0.18
2FOZ	HUMAN ADP-RIBOSYLHYDROLASE 3	LSR	0.18
1ZSX	CRYSTAL STRUCTURE OF HUMAN POTASSIUM CHANNEL KV BETA-SUBUNIT (KCNAB2)	LSR	0.18
2A4D	STRUCTURE OF THE HUMAN UBIQUITIN-CONJUGATING ENZYME E2 VARIANT 1 (UEV-1)	FVR	0.19
2Z5J	FREE TRANSPORTIN 1	LSR	0.19
2O3H	CRYSTAL STRUCTURE OF THE HUMAN C65A APE	LSR	0.19
2CMW	STRUCTURE OF HUMAN CASEIN KINASE 1 GAMMA-1 IN COMPLEX WITH 2-(2-HYDROXYETHYLAMINO)-6-(3-CHLOROANILINO)-9- ISOPROPYLPURINE (CASP TARGET)	LSR	0.21
1NKR	INHIBITORY RECEPTOR (P58-CL42) FOR HUMAN NATURAL KILLER CELLS	LSR	0.21
2HC1	ENGINEERED CATALYTIC DOMAIN OF PROTEIN TYROSINE PHOSPHATASE HPTPBETA.	FVR	0.21
1CZA	MUTANT MONOMER OF RECOMBINANT HUMAN HEXOKINASE TYPE I COMPLEXED WITH GLUCOSE_ GLUCOSE-6-PHOSPHATE_ AND ADP	FVR	0.22
1XJD	CRYSTAL STRUCTURE OF PKC-THETA COMPLEXED WITH STAUROSPORINE AT 2A RESOLUTION	FVR	0.22
1HFC	1.56 ANGSTROM STRUCTURE OF MATURE TRUNCATED HUMAN FIBROBLAST COLLAGENASE	FVR	0.22
1M4K	CRYSTAL STRUCTURE OF THE HUMAN NATURAL KILLER CELL ACTIVATOR RECEPTOR KIR2DS2 (CD158J)	LSR	0.22
3C5H	CRYSTAL STRUCTURE OF THE RAS HOMOLOG DOMAIN OF HUMAN GRLF1 (P190RHOGAP)	FVR	0.22
2BKA	CC3(TIP30)CRYSTAL STRUCURE	FVR	0.22
2PBN	CRYSTAL STRUCTURE OF THE HUMAN TYROSINE RECEPTOR PHOSPHATE GAMMA	FVR	0.22
1ZD3	HUMAN SOLUBLE EPOXIDE HYDROLASE 4-(3-CYCLOHEXYLURIEDO)-BUTYRIC ACID COMPLEX	LSR	0.22
1N3Y	CRYSTAL STRUCTURE OF THE ALPHA-X BETA2 INTEGRIN I DOMAIN	FVR	0.22
1KMV	HUMAN DIHYDROFOLATE REDUCTASE COMPLEXED WITH NADPH AND (Z)- 6-(2-[2_5-DIMETHOXYPHENYL]ETHEN-1-YL)-2_4-DIAMINO-5-METHYLPYRIDO[2_3-D]PYRIMIDINE (SRI-9662)_ A LIPOPHILIC ANTIFOLATE	LSR	0.22
2II0	CRYSTAL STRUCTURE OF CATALYTIC DOMAIN OF SON OF SEVENLESS (REM-CDC25) IN THE ABSENCE OF RAS	FVR	0.23
1FSU	4-SULFATASE (HUMAN)	LSR	0.23
1X9D	CRYSTAL STRUCTURE OF HUMAN CLASS I ALPHA-1_2-MANNOSIDASE IN COMPLEX WITH THIO-DISACCHARIDE SUBSTRATE ANALOGUE	LSR	0.23
2NZ2	CRYSTAL STRUCTURE OF HUMAN ARGININOSUCCINATE SYNTHASE IN COMPLEX WITH ASPARTATE AND CITRULLINE	FVR	0.24
2IVV	CRYSTAL STRUCTURE OF PHOSPHORYLATED RET TYROSINE KINASE DOMAIN COMPLEXED WITH THE INHIBITOR PP1	LSR	0.24

2JC9	CRYSTAL STRUCTURE OF HUMAN CYTOSOLIC 5'-NUCLEOTIDASE II IN COMPLEX WITH ADENOSINE	LSR	0.24
1R1H	STRUCTURAL ANALYSIS OF NEPRILYSIN WITH VARIOUS SPECIFIC AND POTENT INHIBITORS	LSR	0.24
2HI4	CRYSTAL STRUCTURE OF HUMAN MICROSOMAL P450 1A2 IN COMPLEX WITH ALPHA-NAPHTHOFLAVONE	LSR	0.24
1CZA	MUTANT MONOMER OF RECOMBINANT HUMAN HEXOKINASE TYPE I COMPLEXED WITH GLUCOSE_ GLUCOSE-6-PHOSPHATE_ AND ADP	FVR	0.24
2QTZ	CRYSTAL STRUCTURE OF THE NADP+-BOUND FAD-CONTAINING FNR- LIKE MODULE OF HUMAN METHIONINE SYNTHASE REDUCTASE	FVR	0.24
1A7S	ATOMIC RESOLUTION STRUCTURE OF HBP	LSR	0.25
2ODT	STRUCTURE OF HUMAN INOSITOL 1_3_4-TRISPHOSPHATE 5/6-KINASE	LSR	0.25
1Z5V	CRYSTAL STRUCTURE OF HUMAN GAMMA-TUBULIN BOUND TO GTPGAMMAS	LSR	0.25
1P5Z	STRUCTURE OF HUMAN DCK COMPLEXED WITH CYTARABINE AND ADP-MG	LSR	0.26
2F4J	STRUCTURE OF THE KINASE DOMAIN OF AN IMATINIB-RESISTANT ABL MUTANT IN COMPLEX WITH THE AURORA KINASE INHIBITOR VX-680	LSR	0.27
1CZA	MUTANT MONOMER OF RECOMBINANT HUMAN HEXOKINASE TYPE I COMPLEXED WITH GLUCOSE_ GLUCOSE-6-PHOSPHATE_ AND ADP	LSR	0.27
1MZA	CRYSTAL STRUCTURE OF HUMAN PRO-GRANZYME K	LSR	0.27
2P39	CRYSTAL STRUCTURE OF HUMAN FGF23	LSR	0.29
2R2P	KINASE DOMAIN OF HUMAN EPHRIN TYPE-A RECEPTOR 5 (EPHA5)	LSR	0.29
1ZED	ALKALINE PHOSPHATASE FROM HUMAN PLACENTA IN COMPLEX WITH P-NITROPHENYL-PHOSPHONATE	LSR	0.29
1B0F	CRYSTAL STRUCTURE OF HUMAN NEUTROPHIL ELASTASE WITH MDL 101 146	FVR	0.29
3COI	CRYSTAL STRUCTURE OF P38DELTA KINASE	LSR	0.29
2BLE	STRUCTURE OF HUMAN GUANOSINE MONOPHOSPHATE REDUCTASE GMPR1 IN COMPLEX WITH GMP	LSR	0.29
2HXP	CRYSTAL STRUCTURE OF THE HUMAN PHOSPHATASE (DUSP9)	LSR	0.29
2HGS	HUMAN GLUTATHIONE SYNTHETASE	LSR	0.29
1IJB	THE VON WILLEBRAND FACTOR MUTANT (I546V) A1 DOMAIN	FVR	0.30
1T32	A DUAL INHIBITOR OF THE LEUKOCYTE PROTEASES CATHEPSIN G AND CHYMASE WITH THERAPEUTIC EFFICACY IN ANIMALS MODELS OF INFLAMMATION	LSR	0.30
2UXW	CRYSTAL STRUCTURE OF HUMAN VERY LONG CHAIN ACYL-COA DEHYDROGENASE (ACADVL)	LSR	0.30
1T5I	CRYSTAL STRUCTURE OF THE C-TERMINAL DOMAIN OF UAP56	LSR	0.30
1KMV	HUMAN DIHYDROFOLATE REDUCTASE COMPLEXED WITH NADPH AND (Z)- 6-(2-[2_5-DIMETHOXYPHENYL]ETHEN-1-YL)-2_4-DIAMINO-5-METHYLPYRIDO[2_3-D]PYRIMIDINE (SRI-9662)_ A LIPOPHILIC ANTIFOLATE	LSR	0.31
1FEW	CRYSTAL STRUCTURE OF SMAC/DIABLO	LSR	0.31
2AA2	MINERALOCORTICOID RECEPTOR WITH BOUND ALDOSTERONE	FVR	0.31
2B1P	INHIBITOR COMPLEX OF JNK3	LSR	0.31
1HUW	THE CRYSTAL STRUCTURE OF AFFINITY-MATURED HUMAN GROWTH HORMONE AT 2 ANGSTROMS RESOLUTION	LSR	0.32
1TA0	THREE-DIMENSIONAL STRUCTURE OF A RNA-POLYMERASE II BINDING PROTEIN WITH ASSOCIATED LIGAND.	LSR	0.32
2EVA	STRUCTURAL BASIS FOR THE INTERACTION OF TAK1 KINASE WITH ITS ACTIVATING PROTEIN TAB1	LSR	0.32
2A8B	CRYSTAL STRUCTURE OF THE CATALYTIC DOMAIN OF HUMAN TYROSINE PHOSPHATASE RECEPTOR_ TYPE R	LSR	0.32
1WER	RAS-GTPASE-ACTIVATING DOMAIN OF HUMAN P120GAP	LSR	0.32
1S31	CRYSTAL STRUCTURE ANALYSIS OF THE HUMAN TUB PROTEIN (ISOFORM A) SPANNING RESIDUES 289 THROUGH 561	LSR	0.32
1A3S	HUMAN UBC9	LSR	0.32
2JEO	CRYSTAL STRUCTURE OF HUMAN URIDINE-CYTIDINE KINASE 1	LSR	0.32

1TA0	THREE-DIMENSIONAL STRUCTURE OF A RNA-POLYMERASE II BINDING PROTEIN WITH ASSOCIATED LIGAND.	LSR	0.33
1OW1	CRYSTAL STRUCTURE OF THE SPOC DOMAIN OF THE HUMAN TRANSCRIPTIONAL COREPRESSOR_ SHARP.	LSR	0.33
1J1L	CRYSTAL STRUCTURE OF HUMAN PIRIN: A BCL-3 AND NUCLEAR FACTOR I INTERACTING PROTEIN AND A CUPIN SUPERFAMILY MEMBE	LSR	0.34
1FSU	4-SULFATASE (HUMAN)	LSR	0.34
2QCF	CRYSTAL STRUCTURE OF THE OROTIDINE-5'-MONOPHOSPHATE DECARBOXYLASE DOMAIN (ASP312ASN MUTANT) OF HUMAN UMP SYNTHASE BOUND TO 5-FLUORO-UMP	LSR	0.37
2FST	MITOGEN ACTIVATED PROTEIN KINASE P38ALPHA (D176A+F327L) ACTIVATING MUTANT	LSR	0.37
1Z7C	CRYSTAL STRUCTURE OF HUMAN PLACENTAL LACTOGEN	LSR	0.37
1CJM	HUMAN SULT1A3 WITH SULFATE BOUND	LSR	0.37
1WOJ	CRYSTAL STRUCTURE OF HUMAN PHOSPHODIESTERASE	LSR	0.37
1ZD3	HUMAN SOLUBLE EPOXIDE HYDROLASE 4-(3-CYCLOHEXYLURIEDO)-BUTYRIC ACID COMPLEX	LSR	0.37
2Q0Z	CRYSTAL STRUCTURE OF Q9P172/SEC63 FROM HOMO SAPIENS. NORTHEAST STRUCTURAL GENOMICS TARGET HR1979.	LSR	0.37
1GZK	MOLECULAR MECHANISM FOR THE REGULATION OF PROTEIN KINASE B/ AKT BY HYDROPHOBIC MOTIF PHOSPHORYLATION	LSR	0.37
1XJD	CRYSTAL STRUCTURE OF PKC-THETA COMPLEXED WITH STAUROSPORINE AT 2A RESOLUTION	LSR	0.38
2PZ1	CRYSTAL STRUCTURE OF AUTO-INHIBITED ASEF	LSR	0.39
1LS6	HUMAN SULT1A1 COMPLEXED WITH PAP AND P-NITROPHENOL	LSR	0.39
1W6K	STRUCTURE OF HUMAN OSC IN COMPLEX WITH LANOSTEROL	LSR	0.40
2CL3	CRYSTAL STRUCTURE OF HUMAN CLEAVAGE AND POLYADENYLATION SPECIFICITY FACTOR 5 (CPSF5)	LSR	0.40
3BOR	CRYSTAL STRUCTURE OF THE DEADC DOMAIN OF HUMAN TRANSLATION INITIATION FACTOR 4A-2	LSR	0.41
2RAJ	SO4 BOUND PX-BAR MEMBRANE REMODELING UNIT OF SORTING NEXIN	LSR	0.42
1HGU	HUMAN GROWTH HORMONE	LSR	0.42
1NUF	ROLE OF CALCIUM IONS IN THE ACTIVATION AND ACTIVITY OF THE TRANSGLUTAMINASE 3 ENZYME	LSR	0.43
1W0H	CRYSTALLOGRAPHIC STRUCTURE OF THE NUCLEASE DOMAIN OF 3'HEXO A DEDDH FAMILY MEMBER_ BOUND TO RAMP	LSR	0.43
2ODT	STRUCTURE OF HUMAN INOSITOL 1_3_4-TRISPHOSPHATE 5/6-KINASE	LSR	0.45
3CC6	CRYSTAL STRUCTURE OF KINASE DOMAIN OF PROTEIN TYROSINE KINASE 2 BETA (PTK2B)	LSR	0.45
2OEW	STRUCTURE OF ALIX/AIP1 BRO1 DOMAIN	LSR	0.46
1B0F	CRYSTAL STRUCTURE OF HUMAN NEUTROPHIL ELASTASE WITH MDL 101 14[illegible]	LSR	0.50
1AOA	N-TERMINAL ACTIN-CROSSLINKING DOMAIN FROM HUMAN FIMBRIN	LSR	0.51
1MFM	MONOMERIC HUMAN SOD MUTANT F50E/G51E/E133Q AT ATOMIC RESOLUTION	LSR	0.52
2REP	CRYSTAL STRUCTURE OF THE MOTOR DOMAIN OF HUMAN KINESIN FAMILY MEMBER C1	LSR	0.53
1J8U	CATALYTIC DOMAIN OF HUMAN PHENYLALANINE HYDROXYLASE FE(II) IN COMPLEX WITH TETRAHYDROBIOPTERIN	LSR	2.00
1KL9	CRYSTAL STRUCTURE OF THE N-TERMINAL SEGMENT OF HUMAN EUKARYOTIC INITIATION FACTOR 2ALPHA	LSR	2.00
1MP8	CRYSTAL STRUCTURE OF FOCAL ADHESION KINASE (FAK)	LSR	2.00
1P49	STRUCTURE OF HUMAN PLACENTAL ESTRONE/DHEA SULFATASE	LSR	2.00
1XA6	CRYSTAL STRUCTURE OF THE HUMAN BETA2-CHIMAERIN	LSR	2.00
2IQC	CRYSTAL STRUCTURE OF HUMAN FANCF PROTEIN THAT FUNCTIONS IN THE ASSEMBLY OF A DNA DAMAGE SIGNALING COMPLEX	LSR	2.00
2OO8	SYNTHESIS_ STRUCTURAL ANALYSIS_ AND SAR STUDIES OF TRIAZINE DERIVATIVES AS POTENT_ SELECTIVE TIE-2 INHIBITORS	LSR	2.00

2QV2	A ROLE OF THE LOWE SYNDROME PROTEIN OCRL IN EARLY STEPS OF THE ENDOCYTIC PATHWAY	LSR	2.00
2REI	KINASE DOMAIN OF HUMAN EPHRIN TYPE-A RECEPTOR 7 (EPHA7)	LSR	2.00
3BIY	CRYSTAL STRUCTURE OF P300 HISTONE ACETYLTRANSFERASE DOMAIN IN COMPLEX WITH A BISUBSTRATE INHIBITOR_ LYS-COA	LSR	2.00
	**kallikrein 1**		
1H0C	THE CRYSTAL STRUCTURE OF HUMAN ALANINE:GLYOXYLATE AMINOTRANSFERASE	VLR	0.11
1SZ7	CRYSTAL STRUCTURE OF HUMAN BET3	VLR	0.12
1RKB	THE STRUCTURE OF ADRENAL GLAND PROTEIN AD-004	VLR	0.13
2AXN	CRYSTAL STRUCTURE OF THE HUMAN INDUCIBLE FORM 6-PHOSPHOFRUCTO-2-KINASE/FRUCTOSE-2_6-BISPHOSPHATASE	VLR	0.13
1IMV	2.85 A CRYSTAL STRUCTURE OF PEDF	VLR	0.14
3CKK	CRYSTAL STRUCTURE OF HUMAN METHYLTRANSFERASE-LIKE PROTEIN 1	VLR	0.14
1CZT	CRYSTAL STRUCTURE OF THE C2 DOMAIN OF HUMAN COAGULATION FACTOR V	PFR	0.14
2QYL	CRYSTAL STRUCTURE OF PDE4B2B IN COMPLEX WITH INHIBITOR NPV	VLR	0.14
1E8Y	STRUCTURE DETERMINANTS OF PHOSPHOINOSITIDE 3-KINASE INHIBITION BY WORTMANNIN_ LY294002_ QUERCETIN_ MYRICETIN AND STAUROSPORINE	VLR	0.15
1SO7	MALTOSE-INDUCED STRUCTURE OF THE HUMAN CYTOLSOLIC SIALIDASE NEU2	VLR	0.15
2JAV	HUMAN KINASE WITH PYRROLE-INDOLINONE LIGAND	VLR	0.16
1R2Q	CRYSTAL STRUCTURE OF HUMAN RAB5A GTPASE DOMAIN AT 1.05 A RESOLUTION	VLR	0.16
1Z0I	GDP-BOUND RAB21 GTPASE	VLR	0.18
3BCH	CRYSTAL STRUCTURE OF THE HUMAN LAMININ RECEPTOR PRECURSOR	VLR	0.18
2REW	CRYSTAL STRUCTURE OF PPARALPHA LIGAND BINDING DOMAIN WITH BMS-631707	VLR	0.18
2GRY	CRYSTAL STRUCTURE OF THE HUMAN KIF2 MOTOR DOMAIN IN COMPLEX WITH ADP	VLR	0.18
2B5M	CRYSTAL STRUCTURE OF DDB1	VLR	0.18
2FY7	CRYSTAL STRUCTURE OF THE CATALYTIC DOMAIN OF THE HUMAN BETA1_4-GALACTOSYLTRANSFERASE MUTANT M339H IN APO FORM	PFR	0.18
2UXW	CRYSTAL STRUCTURE OF HUMAN VERY LONG CHAIN ACYL-COA DEHYDROGENASE (ACADVL)	VLR	0.19
2HEH	CRYSTAL STRUCTURE OF THE KIF2C MOTOR DOMAIN (CASP TARGET)	VLR	0.19
2H58	CRYSTAL STRUCTURE OF THE KIFC3 MOTOR DOMAIN IN COMPLEX WITH ADF	VLR	0.19
2Q8K	THE CRYSTAL STRUCTURE OF EBP1	VLR	0.19
2EBC	MECHANISM UNDERLYING THE CRITICAL CONTRIBUTION OF A SWITCH II RESIDUE IN A HETEROTRIMERIC G-PROTEIN ALPHA SUBUNIT DURING C. ELEGANS ASYMMETRIC CELL DIVISION	VLR	0.20
2A2C	X-RAY STRUCTURE OF HUMAN N-ACETYL GALACTOSAMINE KINASE COMPLEXED WITH MG-ADP AND N-ACETYL GALACTOSAMINE 1-PHOSPHATE	VLR	0.20
1RGP	GTPASE-ACTIVATION DOMAIN FROM RHOGAP	VLR	0.21
1N26	CRYSTAL STRUCTURE OF THE EXTRA-CELLULAR DOMAINS OF HUMAN INTERLEUKIN-6 RECEPTOR ALPHA CHAIN	VLR	0.21
2AEX	THE 1.58A CRYSTAL STRUCTURE OF HUMAN COPROPORPHYRINOGEN OXIDASE REVEALS THE STRUCTURAL BASIS OF HEREDITARY COPROPORPHYRIA	VLR	0.21
2QNK	CRYSTAL STRUCTURE OF HUMAN 3-HYDROXYANTHRANILATE 3_4-DIOXYGENASE	VLR	0.21
1OHC	STRUCTURE OF THE PROLINE DIRECTED PHOSPHATASE CDC14	PFR	0.21
2FCB	HUMAN FC GAMMA RECEPTOR IIB ECTODOMAIN (CD32)	VLR	0.21

1TXD	CRYSTAL STRUCTURE OF THE DH/PH DOMAINS OF LEUKEMIA-ASSOCIATED RHOGEF	VLR	0.21
2HE3	CRYSTAL STRUCTURE OF THE SELENOCYSTEINE TO CYSTEINE MUTANT OF HUMAN GLUTATHIONINE PEROXIDASE 2 (GPX2)	PFR	0.21
2R99	CRYSTAL STRUCTURE OF CYCLOPHILIN ABH-LIKE DOMAIN OF HUMAN PEPTIDYLPROLYL ISOMERASE E ISOFORM 1	VLR	0.21
1IMV	2.85 A CRYSTAL STRUCTURE OF PEDF	VLR	0.21
2A1X	HUMAN PHYTANOYL-COA 2-HYDROXYLASE IN COMPLEX WITH IRON AND 2-OXOGLUTARATE	PFR	0.22
1HDO	HUMAN BILIVERDIN IX BETA REDUCTASE: NADP COMPLEX	VLR	0.22
1UU3	STRUCTURE OF HUMAN PDK1 KINASE DOMAIN IN COMPLEX WITH LY333531	PFR	0.23
2VGE	CRYSTAL STRUCTURE OF THE C-TERMINAL REGION OF HUMAN IASPP	VLR	0.23
1XQJ	3.10 A CRYSTAL STRUCTURE OF MASPIN_ SPACE GROUP I 4 2 2	PFR	0.23
1BY7	HUMAN PLASMINOGEN ACTIVATOR INHIBITOR-2. LOOP (66-98) DELETION MUTANT	PFR	0.23
1RGP	GTPASE-ACTIVATION DOMAIN FROM RHOGAP	VLR	0.23
2OJ9	STRUCTURE OF IGF-1R KINASE DOMAIN COMPLEXED WITH A BENZIMIDAZOLE INHIBITOR	VLR	0.23
1IAP	CRYSTAL STRUCTURE OF P115RHOGEF RGRGS DOMAIN	VLR	0.24
1Z57	CRYSTAL STRUCTURE OF HUMAN CLK1 IN COMPLEX WITH 10Z-HYMENIALDISINE	PFR	0.24
1KKU	CRYSTAL STRUCTURE OF NUCLEAR HUMAN NICOTINAMIDE MONONUCLEOTIDE ADENYLYLTRANSFERASE	VLR	0.24
3GRS	REFINED STRUCTURE OF GLUTATHIONE REDUCTASE AT 1.54 ANGSTROMS RESOLUTION	VLR	0.24
3BI1	X-RAY STRUCTURE OF HUMAN GLUTAMATE CARBOXYPEPTIDASE II (GCPII) IN COMPLEX WITH A TRANSITION STATE ANALOG OF METHOTREXATE-GLU	VLR	0.24
2VPJ	CRYSTAL STRUCTURE OF THE KELCH DOMAIN OF HUMAN KLHL12	VLR	0.24
2PL3	HUMAN DEAD-BOX RNA HELICASE DDX10_ DEAD DOMAIN IN COMPLEX WITH ADP	VLR	0.25
3CTZ	STRUCTURE OF HUMAN CYTOSOLIC X-PROLYL AMINOPEPTIDASE	VLR	0.25
2JC9	CRYSTAL STRUCTURE OF HUMAN CYTOSOLIC 5'-NUCLEOTIDASE II IN COMPLEX WITH ADENOSINE	VLR	0.25
1WDY	CRYSTAL STRUCTURE OF RIBONUCLEASE	VLR	0.26
2E8A	CRYSTAL STRUCTURE OF THE HUMAN HSP70 ATPASE DOMAIN IN COMPLEX WITH AMP-PNP	VLR	0.26
2C30	CRYSTAL STRUCTURE OF THE HUMAN P21-ACTIVATED KINASE 6	VLR	0.26
2G62	CRYSTAL STRUCTURE OF HUMAN PTPA	VLR	0.26
2GRY	CRYSTAL STRUCTURE OF THE HUMAN KIF2 MOTOR DOMAIN IN COMPLEX WITH ADP	PFR	0.27
2HEH	CRYSTAL STRUCTURE OF THE KIF2C MOTOR DOMAIN (CASP TARGET)	PFR	0.27
1R5L	CRYSTAL STRUCTURE OF HUMAN ALPHA-TOCOPHEROL TRANSFER PROTEIN BOUND TO ITS LIGAND	VLR	0.28
2BH9	X-RAY STRUCTURE OF A DELETION VARIANT OF HUMAN GLUCOSE 6-PHOSPHATE DEHYDROGENASE COMPLEXED WITH STRUCTURAL AND COENZYME NADP	VLR	0.28
2V7O	CRYSTAL STRUCTURE OF HUMAN CALCIUM-CALMODULIN-DEPENDENT PROTEIN KINASE II GAMMA	VLR	0.28
2Z5Y	CRYSTAL STRUCTURE OF HUMAN MONOAMINE OXIDASE A (G110A) WITH HARMINE	PFR	0.28
2Z6H	CRYSTAL STRUCTURE OF BETA-CATENIN ARMADILLO REPEAT REGION AND ITS C-TERMINAL DOMAIN	VLR	0.28
1WCH	CRYSTAL STRUCTURE OF PTPL1 HUMAN TYROSINE PHOSPHATASE MUTATED IN COLORECTAL CANCER- EVIDENCE FOR A SECOND PHOSPHOTYROSINE SUBSTRATE RECOGNITION POCKET	VLR	0.29
2FOZ	HUMAN ADP-RIBOSYLHYDROLASE 3	VLR	0.29

2IUW	CRYSTAL STRUCTURE OF HUMAN ABH3 IN COMPLEX WITH IRON ION AND 2-OXOGLUTARATE	VLR	0.29
2IC1	CRYSTAL STRUCTURE OF HUMAN CYSTEINE DIOXYGENASE IN COMPLEX WITH SUBSTRATE CYSTEINE	VLR	0.29
1D2S	CRYSTAL STRUCTURE OF THE N-TERMINAL LAMININ G-LIKE DOMAIN OF SHBG IN COMPLEX WITH DIHYDROTESTOSTERONE	VLR	0.29
3BI1	X-RAY STRUCTURE OF HUMAN GLUTAMATE CARBOXYPEPTIDASE II (GCPII) IN COMPLEX WITH A TRANSITION STATE ANALOG OF METHOTREXATE-GLU	VLR	0.29
1KGD	CRYSTAL STRUCTURE OF THE GUANYLATE KINASE-LIKE DOMAIN OF HUMAN CASK	VLR	0.30
2HC1	ENGINEERED CATALYTIC DOMAIN OF PROTEIN TYROSINE PHOSPHATASE HPTPBETA.	VLR	0.30
2UZ9	HUMAN GUANINE DEAMINASE (GUAD) IN COMPLEX WITH ZINC AND ITS PRODUCT XANTHINE.	VLR	0.30
1TDH	CRYSTAL STRUCTURE OF HUMAN ENDONUCLEASE VIII-LIKE 1 (NEIL1)	VLR	0.30
2VPJ	CRYSTAL STRUCTURE OF THE KELCH DOMAIN OF HUMAN KLHL12	VLR	0.31
2E3N	CRYSTAL STRUCTURE OF CERT START DOMAIN IN COMPLEX WITH C6-CERAMIDE (P212121)	VLR	0.31
1BIO	HUMAN COMPLEMENT FACTOR D IN COMPLEX WITH ISATOIC ANHYDRIDE INHIBITOR	VLR	0.32
1Z32	STRUCTURE-FUNCTION RELATIONSHIPS IN HUMAN SALIVARY ALPHA-AMYLASE: ROLE OF AROMATIC RESIDUES	PFR	0.33
2H58	CRYSTAL STRUCTURE OF THE KIFC3 MOTOR DOMAIN IN COMPLEX WITH ADP	PFR	0.33
2CW9	CRYSTAL STRUCTURE OF HUMAN TIM44 C-TERMINAL DOMAIN	VLR	0.33
1JDN	CRYSTAL STRUCTURE OF HORMONE RECEPTOR	VLR	0.34
2IPX	HUMAN FIBRILLARIN	PFR	0.35
2I7Q	CRYSTAL STRUCTURE OF HUMAN CHOLINE KINASE A	VLR	0.36
2JEO	CRYSTAL STRUCTURE OF HUMAN URIDINE-CYTIDINE KINASE 1	VLR	0.37
2NR8	CRYSTAL STRUCTURE OF THE HUMAN KIF9 MOTOR DOMAIN IN COMPLEX WITH ADP	PFR	0.39
1TA0	THREE-DIMENSIONAL STRUCTURE OF A RNA-POLYMERASE II BINDING PROTEIN WITH ASSOCIATED LIGAND.	VLR	0.40
1LZJ	GLYCOSYLTRANSFERASE B + UDP + H ANTIGEN ACCEPTOR	VLR	0.40
2QTZ	CRYSTAL STRUCTURE OF THE NADP+-BOUND FAD-CONTAINING FNR- LIKE MODULE OF HUMAN METHIONINE SYNTHASE REDUCTASE	VLR	0.40
3CKK	CRYSTAL STRUCTURE OF HUMAN METHYLTRANSFERASE-LIKE PROTEIN 1	VLR	0.40
1B3J	STRUCTURE OF THE MHC CLASS I HOMOLOG MIC-A_ A GAMMADELTA T CELL LIGAND	VLR	0.41
3COI	CRYSTAL STRUCTURE OF P38DELTA KINASE	PFR	0.42
1HVF	STRUCTURAL AND ELECTROPHYSIOLOGICAL ANALYSIS OF ANNEXIN V MUTANTS. MUTAGENESIS OF HUMAN ANNEXIN V_ AN IN VITRO VOLTAGE-GATED CALCIUM CHANNEL_ PROVIDES INFORMATION ABOUT THE STRUCTURAL FEATURES OF THE ION PATHWAY_ THE VOLTAGE SENSOR AND THE ION SELECTIVITY FILTER	VLR	0.63
1QKM	HUMAN OESTROGEN RECEPTOR BETA LIGAND-BINDING DOMAIN IN COMPLEX WITH PARTIAL AGONIST GENISTEIN	VLR	2.00
1XJD	CRYSTAL STRUCTURE OF PKC-THETA COMPLEXED WITH STAUROSPORINE AT 2A RESOLUTION	PFR	2.00
	**kallikrein-related peptidase 11**		
1CZT	CRYSTAL STRUCTURE OF THE C2 DOMAIN OF HUMAN COAGULATION FACTOR V	PFR	0.14
2FY7	CRYSTAL STRUCTURE OF THE CATALYTIC DOMAIN OF THE HUMAN BETA1_4-GALACTOSYLTRANSFERASE MUTANT M339H IN APO FORM	PFR	0.18
1OHC	STRUCTURE OF THE PROLINE DIRECTED PHOSPHATASE CDC14	PFR	0.21

2HE3	CRYSTAL STRUCTURE OF THE SELENOCYSTEINE TO CYSTEINE MUTANT OF HUMAN GLUTATHIONINE PEROXIDASE 2 (GPX2)	PFR	0.21
2A1X	HUMAN PHYTANOYL-COA 2-HYDROXYLASE IN COMPLEX WITH IRON AND 2-OXOGLUTARATE	PFR	0.22
1UU3	STRUCTURE OF HUMAN PDK1 KINASE DOMAIN IN COMPLEX WITH LY333531	PFR	0.23
1XQJ	3.10 A CRYSTAL STRUCTURE OF MASPIN_ SPACE GROUP I 4 2 2	PFR	0.23
1BY7	HUMAN PLASMINOGEN ACTIVATOR INHIBITOR-2. LOOP (66-98) DELETION MUTANT	PFR	0.23
1Z57	CRYSTAL STRUCTURE OF HUMAN CLK1 IN COMPLEX WITH 10Z-HYMENIALDISINE	PFR	0.24
2GRY	CRYSTAL STRUCTURE OF THE HUMAN KIF2 MOTOR DOMAIN IN COMPLEX WITH ADP	PFR	0.27
2HEH	CRYSTAL STRUCTURE OF THE KIF2C MOTOR DOMAIN (CASP TARGET)	PFR	0.27
2Z5Y	CRYSTAL STRUCTURE OF HUMAN MONOAMINE OXIDASE A (G110A) WITH HARMINE	PFR	0.28
1Z32	STRUCTURE-FUNCTION RELATIONSHIPS IN HUMAN SALIVARY ALPHA-AMYLASE: ROLE OF AROMATIC RESIDUES	PFR	0.33
2H58	CRYSTAL STRUCTURE OF THE KIFC3 MOTOR DOMAIN IN COMPLEX WITH ADI	PFR	0.33
2IPX	HUMAN FIBRILLARIN	PFR	0.35
2NR8	CRYSTAL STRUCTURE OF THE HUMAN KIF9 MOTOR DOMAIN IN COMPLEX WITH ADP	PFR	0.39
3COI	CRYSTAL STRUCTURE OF P38DELTA KINASE	PFR	0.42
1XJD	CRYSTAL STRUCTURE OF PKC-THETA COMPLEXED WITH STAUROSPORINE AT 2A RESOLUTION	PFR	2.00
	**kallikrein-related peptidase 14**		
2VGE	CRYSTAL STRUCTURE OF THE C-TERMINAL REGION OF HUMAN IASPP	VPR	0.20
1TXU	CRYSTAL STRUCTURE OF THE VPS9 DOMAIN OF RABEX-5	VPR	0.20
1ZGK	1.35 ANGSTROM STRUCTURE OF THE KELCH DOMAIN OF KEAP1	VPR	0.20
1Z8D	CRYSTAL STRUCTURE OF HUMAN MUSCLE GLYCOGEN PHOSPHORYLASE A WITH AMP AND GLUCOSE	VPR	0.22
1FA9	HUMAN LIVER GLYCOGEN PHOSPHORYLASE A COMPLEXED WITH AMP	VPR	0.23
1ZGK	1.35 ANGSTROM STRUCTURE OF THE KELCH DOMAIN OF KEAP1	VPR	0.23
1ELV	CRYSTAL STRUCTURE OF THE CATALYTIC DOMAIN OF HUMAN COMPLEMENT C1S PROTEASE	VPR	0.24
2OCG	CRYSTAL STRUCTURE OF HUMAN VALACYCLOVIR HYDROLASE	VPR	0.27
2C9H	STRUCTURE OF MITOCHONDRIAL BETA-KETOACYL SYNTHASE	VPR	0.28
1YCK	CRYSTAL STRUCTURE OF HUMAN PEPTIDOGLYCAN RECOGNITION PROTEIN (PGRP-S)	VPR	0.30
1UCH	DEUBIQUITINATING ENZYME UCH-L3 (HUMAN) AT 1.8 ANGSTROM RESOLUTION	VPR	0.31
2OBV	CRYSTAL STRUCTURE OF THE HUMAN S-ADENOSYLMETHIONINE SYNTHETASE 1 IN COMPLEX WITH THE PRODUCT	VPR	0.32
1TDH	CRYSTAL STRUCTURE OF HUMAN ENDONUCLEASE VIII-LIKE 1 (NEIL1)	VPR	0.33
2ALR	ALDEHYDE REDUCTASE	VPR	0.34
2ALR	ALDEHYDE REDUCTASE	VPR	0.37
2GY5	TIE2 LIGAND-BINDING DOMAIN CRYSTAL STRUCTURE	VPR	0.38
1BYG	KINASE DOMAIN OF HUMAN C-TERMINAL SRC KINASE (CSK) IN COMPLEX WITH INHIBITOR STAUROSPORINE	VPR	0.38
2FN4	THE CRYSTAL STRUCTURE OF HUMAN RAS-RELATED PROTEIN_ RRAS_ IN THE GDP-BOUND STATE	VPR	0.38
1QPC	STRUCTURAL ANALYSIS OF THE LYMPHOCYTE-SPECIFIC KINASE LCK IN COMPLEX WITH NON-SELECTIVE AND SRC FAMILY SELECTIVE KINASE INHIBITORS	VPR	0.40
1WAK	X-RAY STRUCTURE OF SRPK1	VPR	0.41
2J0I	CRYSTAL STRUCTURE OF THE HUMAN P21-ACTIVATED KINASE 4	VPR	0.42

2O36	CRYSTAL STRUCTURE OF ENGINEERED THIMET OLIGOPEPTIDASE WITH NEUROLYSIN SPECIFICITY IN NEUROTENSIN CLEAVAGE SITE	VPR	0.43
2BH9	X-RAY STRUCTURE OF A DELETION VARIANT OF HUMAN GLUCOSE 6-PHOSPHATE DEHYDROGENASE COMPLEXED WITH STRUCTURAL AND COENZYME NADP	VPR	0.43
1LZJ	GLYCOSYLTRANSFERASE B + UDP + H ANTIGEN ACCEPTOR	VPR	0.46
2A4D	STRUCTURE OF THE HUMAN UBIQUITIN-CONJUGATING ENZYME E2 VARIANT 1 (UEV-1)	VPR	0.46
1W0H	CRYSTALLOGRAPHIC STRUCTURE OF THE NUCLEASE DOMAIN OF 3'HEXO A DEDDH FAMILY MEMBER_ BOUND TO RAMP	VPR	0.48
1S1P	CRYSTAL STRUCTURES OF PROSTAGLANDIN D2 11-KETOREDUCTASE (AKR1C3) IN COMPLEX WITH THE NON-STEROIDAL ANTI-INFLAMMATORY DRUGS FLUFENAMIC ACID AND INDOMETHACIN	VPR	0.51
2QYM	CRYSTAL STRUCTURE OF UNLIGANDED PDE4C2	VPR	0.51
1YO8	STRUCUTURE OF THE C-TERMINAL DOMAIN OF HUMAN THROMBOSPONDIN 2	VPR	0.54
1FNH	CRYSTAL STRUCTURE OF HEPARIN AND INTEGRIN BINDING SEGMENT OF HUMAN FIBRONECTIN	VPR	0.56
1UZE	COMPLEX OF THE ANTI-HYPERTENSIVE DRUG ENALAPRILAT AND THE HUMAN TESTICULAR ANGIOTENSIN I-CONVERTING ENZYME	VPR	0.57
2AWF	STRUCTURE OF HUMAN UBIQUITIN-CONJUGATING ENZYME E2 G1	VPR	0.57
2GF9	CRYSTAL STRUCTURE OF HUMAN RAB3D IN COMPLEX WITH GDP	VPR	0.58
2A5J	CRYSTAL STRUCTURE OF HUMAN RAB2B	VPR	0.66
2QT1	HUMAN NICOTINAMIDE RIBOSIDE KINASE 1 IN COMPLEX WITH NICOTINAMIDE RIBOSIDE	VPR	0.75
2PNY	STRUCTURE OF HUMAN ISOPENTENYL-DIPHOSPHATE DELTA-ISOMERASE	VPR	0.76
1YRV	NOVEL UBIQUITIN-CONJUGATING ENZYME	VPR	0.92
2HQ6	STRUCTURE OF THE CYCLOPHILIN_CECYP16-LIKE DOMAIN OF THE SEROLOGICALLY DEFINED COLON CANCER ANTIGEN 10 FROM HOMO SAPIENS	VPR	1.01
1LBD	LIGAND-BINDING DOMAIN OF THE HUMAN NUCLEAR RECEPTOR RXR-ALPHA	VPR	2.00
1MX3	CRYSTAL STRUCTURE OF CTBP DEHYDROGENASE CORE HOLO FORM	VPR	2.00
1P5J	CRYSTAL STRUCTURE ANALYSIS OF HUMAN SERINE DEHYDRATASE	VPR	2.00
1PME	STRUCTURE OF PENTA MUTANT HUMAN ERK2 MAP KINASE COMPLEXED WITH A SPECIFIC INHIBITOR OF HUMAN P38 MAP KINASE	VPR	2.00
1Q1C	CRYSTAL STRUCTURE OF N(1-260) OF HUMAN FKBP52	VPR	2.00
1TBF	CATALYTIC DOMAIN OF HUMAN PHOSPHODIESTERASE 5A IN COMPLEX WITH SILDENAFIL	VPR	2.00
1XAP	STRUCTURE OF THE LIGAND BINDING DOMAIN OF THE RETINOIC ACID RECEPTOR BETA	VPR	2.00
1ZD9	STRUCTURE OF HUMAN ADP-RIBOSYLATION FACTOR-LIKE 10B	VPR	2.00
1ZIV	CATALYTIC DOMAIN OF HUMAN CALPAIN-9	VPR	2.00
1ZJH	STRUCTURE OF HUMAN MUSCLE PYRUVATE KINASE (PKM2)	VPR	2.00
2B3H	CRYSTAL STRUCTURE OF HUMAN METHIONINE AMINOPEPTIDASE TYPE I WITH A THIRD COBALT IN THE ACTIVE SITE	VPR	2.00
2B6H	STRUCTURE OF HUMAN ADP-RIBOSYLATION FACTOR 5	VPR	2.00
2B9E	HUMAN NSUN5 PROTEIN	VPR	2.00
2EW1	CRYSTAL STRUCTURE OF RAB30 IN COMPLEX WITH A GTP ANALOGUE	VPR	2.00
2FAU	CRYSTAL STRUCTURE OF HUMAN VPS26	VPR	2.00
2FG5	CRYSTAL STRUCTURE OF HUMAN RAB31 IN COMPLEX WITH A GTP ANALOGUE	VPR	2.00
2FK9	HUMAN PROTEIN KINASE C_ ETA	VPR	2.00
2FOL	CRYSTAL STRUCTURE OF HUMAN RAB1A IN COMPLEX WITH GDP	VPR	2.00
2FYT	HUMAN HMT1 HNRNP METHYLTRANSFERASE-LIKE 3 (S. CEREVISIAE) PROTEIN	VPR	2.00
2GEE	CRYSTAL STRUCTURE OF HUMAN TYPE III FIBRONECTIN EXTRADOMAIN B AND DOMAIN 8	VPR	2.00

2GFO	STRUCTURE OF THE CATALYTIC DOMAIN OF HUMAN UBIQUITIN CARBOXYL-TERMINAL HYDROLASE 8	VPR	2.00
2GRY	CRYSTAL STRUCTURE OF THE HUMAN KIF2 MOTOR DOMAIN IN COMPLEX WITH ADP	VPR	2.00
2GW2	CRYSTAL STRUCTURE OF THE PEPTIDYL-PROLYL ISOMERASE DOMAIN OF HUMAN CYCLOPHILIN G	VPR	2.00
2H17	STRUCTURE OF HUMAN ADP-RIBOSYLATION FACTOR-LIKE 5 (ARL5) (CASP TARGET)	VPR	2.00
2HEH	CRYSTAL STRUCTURE OF THE KIF2C MOTOR DOMAIN (CASP TARGET)	VPR	2.00
2HZ6	THE CRYSTAL STRUCTURE OF HUMAN IRE1-ALPHA LUMINAL DOMAIN	VPR	2.00
2I7A	DOMAIN IV OF HUMAN CALPAIN 13	VPR	2.00
2I7Q	CRYSTAL STRUCTURE OF HUMAN CHOLINE KINASE A	VPR	2.00
2IL1	CRYSTAL STRUCTURE OF A PREDICTED HUMAN GTPASE IN COMPLEX WITH GDP	VPR	2.00
2IUW	CRYSTAL STRUCTURE OF HUMAN ABH3 IN COMPLEX WITH IRON ION AND 2-OXOGLUTARATE	VPR	2.00
2JC9	CRYSTAL STRUCTURE OF HUMAN CYTOSOLIC 5'-NUCLEOTIDASE II IN COMPLEX WITH ADENOSINE	VPR	2.00
2NR8	CRYSTAL STRUCTURE OF THE HUMAN KIF9 MOTOR DOMAIN IN COMPLEX WITH ADP	VPR	2.00
2OIL	CRYSTAL STRUCTURE OF HUMAN RAB25 IN COMPLEX WITH GDP	VPR	2.00
2QLU	CRYSTAL STRUCTURE OF ACTIVIN RECEPTOR TYPE II KINASE DOMAIN FROM HUMAN	VPR	2.00
2REP	CRYSTAL STRUCTURE OF THE MOTOR DOMAIN OF HUMAN KINESIN FAMILY MEMBER C1	VPR	2.00
2VKQ	CRYSTAL STRUCTURE OF HUMAN CYTOSOLIC 5'-NUCLEOTIDASE III (CN-III_ NT5C3) IN COMPLEX WITH BERYLLIUM TRIFLUORIDE	VPR	2.00
2ZMD	CRYSTAL STRUCTURE OF HUMAN MPS1 CATALYTIC DOMAIN T686A MUTANT IN COMPLEX WITH SP600125 INHIBITOR	VPR	2.00
3BCH	CRYSTAL STRUCTURE OF THE HUMAN LAMININ RECEPTOR PRECURSOR	VPR	2.00
3BD9	HUMAN 3-O-SULFOTRANSFERASE ISOFORM 5 WITH BOUND PAP	VPR	2.00
	**kallikrein-related peptidase 2**		
1CZT	CRYSTAL STRUCTURE OF THE C2 DOMAIN OF HUMAN COAGULATION FACTOR V	PFR	0.14
1N5U	X-RAY STUDY OF HUMAN SERUM ALBUMIN COMPLEXED WITH HEME	ARR	0.17
1VJY	CRYSTAL STRUCTURE OF A NAPHTHYRIDINE INHIBITOR OF HUMAN TGF BETA TYPE I RECEPTOR	ARR	0.17
1N5U	X-RAY STUDY OF HUMAN SERUM ALBUMIN COMPLEXED WITH HEME	ARR	0.17
2FY7	CRYSTAL STRUCTURE OF THE CATALYTIC DOMAIN OF THE HUMAN BETA1_4-GALACTOSYLTRANSFERASE MUTANT M339H IN APO FORM	PFR	0.18
3GRS	REFINED STRUCTURE OF GLUTATHIONE REDUCTASE AT 1.54 ANGSTROMS RESOLUTION	ARR	0.20
1OHC	STRUCTURE OF THE PROLINE DIRECTED PHOSPHATASE CDC14	PFR	0.21
2HE3	CRYSTAL STRUCTURE OF THE SELENOCYSTEINE TO CYSTEINE MUTANT OF HUMAN GLUTATHIONINE PEROXIDASE 2 (GPX2)	PFR	0.21
2A1X	HUMAN PHYTANOYL-COA 2-HYDROXYLASE IN COMPLEX WITH IRON AND 2-OXOGLUTARATE	PFR	0.22
1UU3	STRUCTURE OF HUMAN PDK1 KINASE DOMAIN IN COMPLEX WITH LY333531	PFR	0.23
3CFW	L-SELECTIN LECTIN AND EGF DOMAINS	ARR	0.23
1XQJ	3.10 A CRYSTAL STRUCTURE OF MASPIN_ SPACE GROUP I 4 2 2	PFR	0.23
1BY7	HUMAN PLASMINOGEN ACTIVATOR INHIBITOR-2. LOOP (66-98) DELETION MUTANT	PFR	0.23
1Z57	CRYSTAL STRUCTURE OF HUMAN CLK1 IN COMPLEX WITH 10Z-HYMENIALDISINE	PFR	0.24
2H6D	PROTEIN KINASE DOMAIN OF THE HUMAN 5'-AMP-ACTIVATED PROTEIN KINASE CATALYTIC SUBUNIT ALPHA-2 (AMPK ALPHA-2 CHAIN)	ARR	0.24

2Z5Y	CRYSTAL STRUCTURE OF HUMAN MONOAMINE OXIDASE A (G110A) WITH HARMINE	ARR	0.24
2HI4	CRYSTAL STRUCTURE OF HUMAN MICROSOMAL P450 1A2 IN COMPLEX WITH ALPHA-NAPHTHOFLAVONE	ARR	0.25
2Q8G	STRUCTURE OF PYRUVATE DEHYDROGENASE KINASE ISOFORM 1 IN COMPLEX WITH GLUCOSE-LOWERING DRUG AZD7545	ARR	0.26
1UPV	CRYSTAL STRUCTURE OF THE HUMAN LIVER X RECEPTOR BETA LIGAND BINDING DOMAIN IN COMPLEX WITH A SYNTHETIC AGONIST	ARR	0.27
2GRY	CRYSTAL STRUCTURE OF THE HUMAN KIF2 MOTOR DOMAIN IN COMPLEX WITH ADP	PFR	0.27
2HEH	CRYSTAL STRUCTURE OF THE KIF2C MOTOR DOMAIN (CASP TARGET)	PFR	0.27
2Z5Y	CRYSTAL STRUCTURE OF HUMAN MONOAMINE OXIDASE A (G110A) WITH HARMINE	PFR	0.28
1Z70	1.15A RESOLUTION STRUCTURE OF THE FORMYLGLYCINE GENERATING ENZYME FGE	ARR	0.28
2HRB	CRYSTAL STRUCTURE OF HUMAN CARBONYL REDUCTASE 3_ COMPLEXED WITH NADP+	ARR	0.29
1HDR	THE CRYSTALLOGRAPHIC STRUCTURE OF A HUMAN DIHYDROPTERIDINE REDUCTASE NADH BINARY COMPLEX EXPRESSED IN ESCHERICHIA COLI BY A CDNA CONSTRUCTED FROM ITS RAT HOMOLOGUE	ARR	0.30
1NF1	THE GAP RELATED DOMAIN OF NEUROFIBROMIN	ARR	0.31
1T32	A DUAL INHIBITOR OF THE LEUKOCYTE PROTEASES CATHEPSIN G AND CHYMASE WITH THERAPEUTIC EFFICACY IN ANIMALS MODELS OF INFLAMMATION	ARR	0.32
1Z32	STRUCTURE-FUNCTION RELATIONSHIPS IN HUMAN SALIVARY ALPHA-AMYLASE: ROLE OF AROMATIC RESIDUES	PFR	0.33
2JBO	PROTEIN KINASE MK2 IN COMPLEX WITH AN INHIBITOR (CRYSTAL FORM-1_ SOAKING)	ARR	0.33
2H58	CRYSTAL STRUCTURE OF THE KIFC3 MOTOR DOMAIN IN COMPLEX WITH ADI	PFR	0.33
1FCY	ISOTYPE SELECTIVITY OF THE HUMAN RETINOIC ACID NUCLEAR RECEPTOR HRAR: THE COMPLEX WITH THE RARBETA/GAMMA-SELECTIVE RETINOID CD564	ARR	0.33
2UUI	CRYSTAL STRUCTURE OF HUMAN LEUKOTRIENE C4 SYNTHASE	ARR	0.33
2HI4	CRYSTAL STRUCTURE OF HUMAN MICROSOMAL P450 1A2 IN COMPLEX WITH ALPHA-NAPHTHOFLAVONE	ARR	0.34
1J8U	CATALYTIC DOMAIN OF HUMAN PHENYLALANINE HYDROXYLASE FE(II) IN COMPLEX WITH TETRAHYDROBIOPTERIN	ARR	0.35
2IPX	HUMAN FIBRILLARIN	PFR	0.35
1W1D	CRYSTAL STRUCTURE OF THE PDK1 PLECKSTRIN HOMOLOGY (PH) DOMAIN BOUND TO INOSITOL (1_3_4_5)-TETRAKISPHOSPHATE	ARR	0.36
1XWI	CRYSTAL STRUCTURE OF VPS4B	ARR	0.37
2OC3	CRYSTAL STRUCTURE OF THE CATALYTIC DOMAIN OF HUMAN PROTEIN TYROSINE PHOSPHATASE NON-RECEPTOR TYPE 18	ARR	0.38
2VR2	HUMAN DIHYDROPYRIMIDINASE	ARR	0.38
2NR8	CRYSTAL STRUCTURE OF THE HUMAN KIF9 MOTOR DOMAIN IN COMPLEX WITH ADP	PFR	0.39
2GFO	STRUCTURE OF THE CATALYTIC DOMAIN OF HUMAN UBIQUITIN CARBOXYL-TERMINAL HYDROLASE 8	ARR	0.40
1N3Y	CRYSTAL STRUCTURE OF THE ALPHA-X BETA2 INTEGRIN I DOMAIN	ARR	0.40
1HU3	MIDDLE DOMAIN OF HUMAN EIF4GII	ARR	0.41
2DE0	CRYSTAL STRUCTURE OF HUMAN ALPHA 1_6-FUCOSYLTRANSFERASE_ FUT8	ARR	0.42
1OW1	CRYSTAL STRUCTURE OF THE SPOC DOMAIN OF THE HUMAN TRANSCRIPTIONAL COREPRESSOR_ SHARP.	ARR	0.42
3COI	CRYSTAL STRUCTURE OF P38DELTA KINASE	PFR	0.42
1QCY	THE CRYSTAL STRUCTURE OF THE I-DOMAIN OF HUMAN INTEGRIN ALPHA1BETA1	ARR	0.43

3COU	CRYSTAL STRUCTURE OF HUMAN NUDIX MOTIF 16 (NUDT16)	ARR	0.47
2V7O	CRYSTAL STRUCTURE OF HUMAN CALCIUM-CALMODULIN-DEPENDENT PROTEIN KINASE II GAMMA	ARR	0.48
1EK5	STRUCTURE OF HUMAN UDP-GALACTOSE 4-EPIMERASE IN COMPLEX WITH NAD+	ARR	0.49
1E8Y	STRUCTURE DETERMINANTS OF PHOSPHOINOSITIDE 3-KINASE INHIBITION BY WORTMANNIN_ LY294002_ QUERCETIN_ MYRICETIN AND STAUROSPORINE	ARR	0.50
1WLJ	HUMAN ISG20	ARR	0.52
1OEC	FGFR2 KINASE DOMAIN	ARR	0.52
2QQ5	CRYSTAL STRUCTURE OF HUMAN SDR FAMILY MEMBER 1	ARR	0.56
1W7L	CRYSTAL STRUCTURE OF HUMAN KYNURENINE AMINOTRANSFERASE I	ARR	0.58
3C0I	CASK CAM-KINASE DOMAIN- 3'-AMP COMPLEX_ P212121 FORM	ARR	0.60
1NTY	CRYSTAL STRUCTURE OF THE FIRST DH/PH DOMAIN OF TRIO TO 1.7	ARR	0.65
1FYV	CRYSTAL STRUCTURE OF THE TIR DOMAIN OF HUMAN TLR1	ARR	0.68
2VGE	CRYSTAL STRUCTURE OF THE C-TERMINAL REGION OF HUMAN IASPP	ARR	0.72
1NN5	CRYSTAL STRUCTURE OF HUMAN THYMIDYLATE KINASE WITH D4TMP + APPNHP	ARR	2.00
1NW3	STRUCTURE OF THE CATALYTIC DOMAIN OF HUMAN DOT1L_ A NON-SET DOMAIN NUCLEOSOMAL HISTONE METHYLTRANSFERASE	ARR	2.00
1R55	CRYSTAL STRUCTURE OF THE CATALYTIC DOMAIN OF HUMAN ADAM 33	ARR	2.00
1XJD	CRYSTAL STRUCTURE OF PKC-THETA COMPLEXED WITH STAUROSPORINE AT 2A RESOLUTION	PFR	2.00
2ANY	EXPRESSION_ CRYSTALLIZATION AND THE THREE-DIMENSIONAL STRUCTURE OF THE CATALYTIC DOMAIN OF HUMAN PLASMA KALLIKREIN: IMPLICATIONS FOR STRUCTURE-BASED DESIGN OF PROTEASE INHIBITORS	ARR	2.00
2FUE	HUMAN ALPHA-PHOSPHOMANNOMUTASE 1 WITH D-MANNOSE 1-PHOSPHATE AND MG2+ COFACTOR BOUND	ARR	2.00
	**kallikrein-related peptidase 3**		
1JTV	CRYSTAL STRUCTURE OF 17BETA-HYDROXYSTEROID DEHYDROGENASE TYPE 1 COMPLEXED WITH TESTOSTERONE	KVY	0.15
3PBH	REFINED CRYSTAL STRUCTURE OF HUMAN PROCATHEPSIN B AT 2.5 ANGSTROM RESOLUTION	RPY	0.16
2NSM	CRYSTAL STRUCTURE OF THE HUMAN CARBOXYPEPTIDASE N (KININASE I) CATALYTIC DOMAIN	KVY	0.19
1H4W	STRUCTURE OF HUMAN TRYPSIN IV (BRAIN TRYPSIN)	KVY	0.19
2E3N	CRYSTAL STRUCTURE OF CERT START DOMAIN IN COMPLEX WITH C6-CERAMIDE (P212121)	KVY	0.19
1LBD	LIGAND-BINDING DOMAIN OF THE HUMAN NUCLEAR RECEPTOR RXR-ALPHA	KVY	0.19
1T32	A DUAL INHIBITOR OF THE LEUKOCYTE PROTEASES CATHEPSIN G AND CHYMASE WITH THERAPEUTIC EFFICACY IN ANIMALS MODELS OF INFLAMMATION	RPY	0.19
1KT0	STRUCTURE OF THE LARGE FKBP-LIKE PROTEIN_ FKBP51_ INVOLVED IN STEROID RECEPTOR COMPLEXES	KVY	0.20
1BIO	HUMAN COMPLEMENT FACTOR D IN COMPLEX WITH ISATOIC ANHYDRIDE INHIBITOR	RPY	0.20
1IAM	STRUCTURE OF THE TWO AMINO-TERMINAL DOMAINS OF HUMAN INTERCELLULAR ADHESION MOLECULE-1_ ICAM-1	KVY	0.20
2CJZ	CRYSTAL STRUCTURE OF THE C472S MUTANT OF HUMAN PROTEIN TYROSINE PHOSPHATASE PTPN5 (STEP_ STRIATUM ENRICHED PHOSPHATASE) IN COMPLEX WITH PHOSPHOTYROSINE	KVY	0.21
2FOZ	HUMAN ADP-RIBOSYLHYDROLASE 3	RPY	0.21
1ZC0	CRYSTAL STRUCTURE OF HUMAN HEMATOPOIETIC TYROSINE PHOSPHATASE (HEPTP) CATALYTIC DOMAIN	KVY	0.21
1NN6	HUMAN PRO-CHYMASE	RPY	0.21

1DHS	CRYSTAL STRUCTURE OF THE NAD COMPLEX OF HUMAN DEOXYHYPUSINE SYNTHASE	KVY	0.22
2J5W	CERULOPLASMIN REVISITED: STRUCTURAL AND FUNCTIONAL ROLES OF VARIOUS METAL CATION BINDING SITES	KVY	0.22
2J5W	CERULOPLASMIN REVISITED: STRUCTURAL AND FUNCTIONAL ROLES OF VARIOUS METAL CATION BINDING SITES	KVY	0.22
1ZS9	CRYSTAL STRUCTURE OF HUMAN ENOLASE-PHOSPHATASE E1	KVY	0.23
2ZFY	CRYSTAL STRUCTURE OF HUMAN OTUBAIN 1	KVY	0.23
1XK5	CRYSTAL STRUCTURE OF THE M3G-CAP-BINDING DOMAIN OF SNURPORTIN1 IN COMPLEX WITH A M3GPPPG-CAP DINUCLEOTIDE	RPY	0.24
1UMK	THE STRUCTURE OF HUMAN ERYTHROCYTE NADH-CYTOCHROME B5 REDUCTASE	KVY	0.26
2J51	CRYSTAL STRUCTURE OF HUMAN STE20-LIKE KINASE BOUND TO 5- AMINO-3-((4-(AMINOSULFONYL)PHENYL)AMINO)-N-(2_6- DIFLUOROPHENYL)-1H-1_2_4-TRIAZOLE-1-CARBOTHIOAMIDE	KVY	0.26
2QOL	HUMAN EPHA3 KINASE AND JUXTAMEMBRANE REGION_ Y596:Y602:S768G TRIPLE MUTANT	RPY	0.26
1XA6	CRYSTAL STRUCTURE OF THE HUMAN BETA2-CHIMAERIN	KVY	0.27
2D7I	CRSYTAL STRUCTURE OF PP-GALNAC-T10 WITH UDP_ GALNAC AND MN2	RPY	0.27
2J5W	CERULOPLASMIN REVISITED: STRUCTURAL AND FUNCTIONAL ROLES OF VARIOUS METAL CATION BINDING SITES	RPY	0.27
1BJ4	RECOMBINANT SERINE HYDROXYMETHYLTRANSFERASE (HUMAN)	KVY	0.28
2J7T	CRYSTAL STRUCTURE OF HUMAN SERINE THREONINE KINASE-10 BOUND TO SU11274	KVY	0.28
2J5W	CERULOPLASMIN REVISITED: STRUCTURAL AND FUNCTIONAL ROLES OF VARIOUS METAL CATION BINDING SITES	RPY	0.29
2QTZ	CRYSTAL STRUCTURE OF THE NADP+-BOUND FAD-CONTAINING FNR- LIKE MODULE OF HUMAN METHIONINE SYNTHASE REDUCTASE	RPY	0.30
1XQJ	3.10 A CRYSTAL STRUCTURE OF MASPIN_ SPACE GROUP I 4 2 2	RPY	0.30
2QMJ	CRYSTRAL STRUCTURE OF THE N-TERMINAL SUBUNIT OF HUMAN MALTASE-GLUCOAMYLASE IN COMPLEX WITH ACARBOSE	KVY	0.30
2REI	KINASE DOMAIN OF HUMAN EPHRIN TYPE-A RECEPTOR 7 (EPHA7)	RPY	0.30
2R2P	KINASE DOMAIN OF HUMAN EPHRIN TYPE-A RECEPTOR 5 (EPHA5)	RPY	0.31
1UMK	THE STRUCTURE OF HUMAN ERYTHROCYTE NADH-CYTOCHROME B5 REDUCTASE	RPY	0.31
2F4J	STRUCTURE OF THE KINASE DOMAIN OF AN IMATINIB-RESISTANT ABL MUTANT IN COMPLEX WITH THE AURORA KINASE INHIBITOR VX-680	KVY	0.34
1NF1	THE GAP RELATED DOMAIN OF NEUROFIBROMIN	KVY	0.35
1MB8	CRYSTAL STRUCTURE OF THE ACTIN BINDING DOMAIN OF PLECTIN	KVY	0.36
2OSA	THE RHO-GAP DOMAIN OF HUMAN N-CHIMAERIN	KVY	0.37
2J51	CRYSTAL STRUCTURE OF HUMAN STE20-LIKE KINASE BOUND TO 5- AMINO-3-((4-(AMINOSULFONYL)PHENYL)AMINO)-N-(2_6- DIFLUOROPHENYL)-1H-1_2_4-TRIAZOLE-1-CARBOTHIOAMIDE	RPY	0.37
2I75	CRYSTAL STRUCTURE OF HUMAN PROTEIN TYROSINE PHOSPHATASE N4 (PTPN4)	KVY	0.42
1J1L	CRYSTAL STRUCTURE OF HUMAN PIRIN: A BCL-3 AND NUCLEAR FACTOR I INTERACTING PROTEIN AND A CUPIN SUPERFAMILY MEMBE	KVY	0.45
2QXI	HIGH RESOLUTION STRUCTURE OF HUMAN KALLIKREIN 7 IN COMPLEX WITH SUC-ALA-ALA-PRO-PHE-CHLOROMETHYLKETONE	KVY	0.45
2NNJ	CYP2C8DH COMPLEXED WITH FELODIPINE	KVY	0.47
1R9O	CRYSTAL STRUCTURE OF P4502C9 WITH FLURBIPROFEN BOUND	KVY	0.47
2UWN	CRYSTAL STRUCTURE OF HUMAN COMPLEMENT FACTOR H_ SCR DOMAINS 6-8 (H402 RISK VARIANT)_ IN COMPLEX WITH LIGAND.	RPY	0.51
1K04	CRYSTAL STRUCTURE OF THE FOCAL ADHESION TARGETING DOMAIN OF FOCAL ADHESION KINASE	KVY	0.52
3B6E	CRYSTAL STRUCTURE OF HUMAN DECH-BOX RNA HELICASE MDA5 (MELANOMA DIFFERENTIATION-ASSOCIATED PROTEIN 5)_ DECH- DOMAIN	RPY	0.58

2NSQ	CRYSTAL STRUCTURE OF THE C2 DOMAIN OF THE HUMAN E3 UBIQUITIN PROTEIN LIGASE NEDD4-LIKE PROTEIN	RPY	2.00
	**kallikrein-related peptidase 4**		
1H0C	THE CRYSTAL STRUCTURE OF HUMAN ALANINE:GLYOXYLATE AMINOTRANSFERASE	VLR	0.11
1C25	HUMAN CDC25A CATALYTIC DOMAIN	VLK	0.12
1SZ7	CRYSTAL STRUCTURE OF HUMAN BET3	VLR	0.12
1RKB	THE STRUCTURE OF ADRENAL GLAND PROTEIN AD-004	VLR	0.13
2NZL	CRYSTAL STRUCTURE OF HUMAN HYDROXYACID OXIDASE 1	VLK	0.13
2Z6H	CRYSTAL STRUCTURE OF BETA-CATENIN ARMADILLO REPEAT REGION AND ITS C-TERMINAL DOMAIN	VLK	0.13
2AXN	CRYSTAL STRUCTURE OF THE HUMAN INDUCIBLE FORM 6-PHOSPHOFRUCTO-2-KINASE/FRUCTOSE-2_6-BISPHOSPHATASE	VLR	0.13
1IMV	2.85 A CRYSTAL STRUCTURE OF PEDF	VLR	0.14
3CTZ	STRUCTURE OF HUMAN CYTOSOLIC X-PROLYL AMINOPEPTIDASE	VLK	0.14
1UV5	GLYCOGEN SYNTHASE KINASE 3 BETA COMPLEXED WITH 6-BROMOINDIRUBIN-3'-OXIME	VLK	0.14
3CKK	CRYSTAL STRUCTURE OF HUMAN METHYLTRANSFERASE-LIKE PROTEIN 1	VLR	0.14
1CZT	CRYSTAL STRUCTURE OF THE C2 DOMAIN OF HUMAN COAGULATION FACTOR V	PFR	0.14
2QYL	CRYSTAL STRUCTURE OF PDE4B2B IN COMPLEX WITH INHIBITOR NPV	VLR	0.14
1FA9	HUMAN LIVER GLYCOGEN PHOSPHORYLASE A COMPLEXED WITH AMP	VLK	0.14
1E8Y	STRUCTURE DETERMINANTS OF PHOSPHOINOSITIDE 3-KINASE INHIBITION BY WORTMANNIN_ LY294002_ QUERCETIN_ MYRICETIN AND STAUROSPORINE	VLR	0.15
2HGS	HUMAN GLUTATHIONE SYNTHETASE	VLK	0.15
1SO7	MALTOSE-INDUCED STRUCTURE OF THE HUMAN CYTOLSOLIC SIALIDASE NEU2	VLR	0.15
2EVA	STRUCTURAL BASIS FOR THE INTERACTION OF TAK1 KINASE WITH ITS ACTIVATING PROTEIN TAB1	VLK	0.15
1SQW	CRYSTAL STRUCTURE OF KD93_ A NOVEL PROTEIN EXPRESSED IN THE HUMAN PRO	VLK	0.16
3BKB	CRYSTAL STRUCTURE OF HUMAN FELINE SARCOMA VIRAL ONCOGENE HOMOLOGUE (V-FES)	VLK	0.16
1AYE	HUMAN PROCARBOXYPEPTIDASE A2	VLK	0.16
1Q1C	CRYSTAL STRUCTURE OF N(1-260) OF HUMAN FKBP52	VLK	0.16
2JAV	HUMAN KINASE WITH PYRROLE-INDOLINONE LIGAND	VLR	0.16
1EM2	STAR-RELATED LIPID TRANSPORT DOMAIN OF MLN64	VLK	0.16
1R2Q	CRYSTAL STRUCTURE OF HUMAN RAB5A GTPASE DOMAIN AT 1.05 A RESOLUTION	VLR	0.16
1TQN	CRYSTAL STRUCTURE OF HUMAN MICROSOMAL P450 3A4	VLK	0.17
1TA0	THREE-DIMENSIONAL STRUCTURE OF A RNA-POLYMERASE II BINDING PROTEIN WITH ASSOCIATED LIGAND.	VLK	0.17
2F1W	CRYSTAL STRUCTURE OF THE TRAF-LIKE DOMAIN OF HAUSP/USP7	VLK	0.17
1YCK	CRYSTAL STRUCTURE OF HUMAN PEPTIDOGLYCAN RECOGNITION PROTEIN (PGRP-S)	VLK	0.17
1IJB	THE VON WILLEBRAND FACTOR MUTANT (I546V) A1 DOMAIN	VLK	0.17
2A7V	HUMAN MITOCHONDRIAL SERINE HYDROXYMETHYLTRANSFERASE 2	VLK	0.17
1NTY	CRYSTAL STRUCTURE OF THE FIRST DH/PH DOMAIN OF TRIO TO 1.7	VLK	0.17
1LUG	FULL MATRIX ERROR ANALYSIS OF CARBONIC ANHYDRASE	VLK	0.17
3BCH	CRYSTAL STRUCTURE OF THE HUMAN LAMININ RECEPTOR PRECURSOR	VLK	0.17
1RZ4	CRYSTAL STRUCTURE OF HUMAN EIF3K	VLK	0.17
2UV4	CRYSTAL STRUCTURE OF A CBS DOMAIN PAIR FROM THE REGULATORY GAMMA1 SUBUNIT OF HUMAN AMPK IN COMPLEX WITH AMP	VLK	0.18
1Z0I	GDP-BOUND RAB21 GTPASE	VLR	0.18
3BCH	CRYSTAL STRUCTURE OF THE HUMAN LAMININ RECEPTOR PRECURSOR	VLR	0.18

2REW	CRYSTAL STRUCTURE OF PPARALPHA LIGAND BINDING DOMAIN WITH BMS-631707	VLR	0.18
2GRY	CRYSTAL STRUCTURE OF THE HUMAN KIF2 MOTOR DOMAIN IN COMPLEX WITH ADP	VLR	0.18
2A8B	CRYSTAL STRUCTURE OF THE CATALYTIC DOMAIN OF HUMAN TYROSINE PHOSPHATASE RECEPTOR_ TYPE R	VLK	0.18
2AXN	CRYSTAL STRUCTURE OF THE HUMAN INDUCIBLE FORM 6-PHOSPHOFRUCTO-2-KINASE/FRUCTOSE-2_6-BISPHOSPHATASE	VLK	0.18
2B5M	CRYSTAL STRUCTURE OF DDB1	VLR	0.18
2NZ2	CRYSTAL STRUCTURE OF HUMAN ARGININOSUCCINATE SYNTHASE IN COMPLEX WITH ASPARTATE AND CITRULLINE	VLK	0.18
1DR9	CRYSTAL STRUCTURE OF A SOLUBLE FORM OF B7-1 (CD80)	VLK	0.18
1XMJ	CRYSTAL STRUCTURE OF HUMAN DELTAF508 HUMAN NBD1 DOMAIN WITH ATP	VLK	0.18
2FY7	CRYSTAL STRUCTURE OF THE CATALYTIC DOMAIN OF THE HUMAN BETA1_4-GALACTOSYLTRANSFERASE MUTANT M339H IN APO FORM	PFR	0.18
1LUG	FULL MATRIX ERROR ANALYSIS OF CARBONIC ANHYDRASE	VLK	0.19
2CY7	THE CRYSTAL STRUCTURE OF HUMAN ATG4B	VLK	0.19
1WDY	CRYSTAL STRUCTURE OF RIBONUCLEASE	VLK	0.19
1HCB	ENZYME-SUBSTRATE INTERACTIONS: STRUCTURE OF HUMAN CARBONIC ANHYDRASE I COMPLEXED WITH BICARBONATE	VLK	0.19
2ALD	HUMAN MUSCLE ALDOLASE	VLK	0.19
2UXW	CRYSTAL STRUCTURE OF HUMAN VERY LONG CHAIN ACYL-COA DEHYDROGENASE (ACADVL)	VLR	0.19
2HEH	CRYSTAL STRUCTURE OF THE KIF2C MOTOR DOMAIN (CASP TARGET)	VLR	0.19
2H58	CRYSTAL STRUCTURE OF THE KIFC3 MOTOR DOMAIN IN COMPLEX WITH ADF	VLR	0.19
2Q8K	THE CRYSTAL STRUCTURE OF EBP1	VLR	0.19
2EBC	MECHANISM UNDERLYING THE CRITICAL CONTRIBUTION OF A SWITCH II RESIDUE IN A HETEROTRIMERIC G-PROTEIN ALPHA SUBUNIT DURING C. ELEGANS ASYMMETRIC CELL DIVISION	VLR	0.20
2VGE	CRYSTAL STRUCTURE OF THE C-TERMINAL REGION OF HUMAN IASPP	VPR	0.20
1YH2	UBIQUITIN-CONJUGATING ENZYME HSPC150	VLK	0.20
1ZRZ	CRYSTAL STRUCTURE OF THE CATALYTIC DOMAIN OF ATYPICAL PROTEIN KINASE C-IOTA	VLK	0.20
1TXU	CRYSTAL STRUCTURE OF THE VPS9 DOMAIN OF RABEX-5	VPR	0.20
1ZGK	1.35 ANGSTROM STRUCTURE OF THE KELCH DOMAIN OF KEAP1	VPR	0.20
1TXU	CRYSTAL STRUCTURE OF THE VPS9 DOMAIN OF RABEX-5	VLK	0.20
1IJB	THE VON WILLEBRAND FACTOR MUTANT (I546V) A1 DOMAIN	VLK	0.20
2A2C	X-RAY STRUCTURE OF HUMAN N-ACETYL GALACTOSAMINE KINASE COMPLEXED WITH MG-ADP AND N-ACETYL GALACTOSAMINE 1-PHOSPHATE	VLR	0.20
2OIL	CRYSTAL STRUCTURE OF HUMAN RAB25 IN COMPLEX WITH GDP	VLK	0.20
1R1H	STRUCTURAL ANALYSIS OF NEPRILYSIN WITH VARIOUS SPECIFIC AND POTENT INHIBITORS	VLK	0.20
1RGP	GTPASE-ACTIVATION DOMAIN FROM RHOGAP	VLR	0.21
3CEK	CRYSTAL STRUCTURE OF HUMAN DUAL SPECIFICITY PROTEIN KINASE (TTK)	VLK	0.21
1N26	CRYSTAL STRUCTURE OF THE EXTRA-CELLULAR DOMAINS OF HUMAN INTERLEUKIN-6 RECEPTOR ALPHA CHAIN	VLR	0.21
2AEX	THE 1.58A CRYSTAL STRUCTURE OF HUMAN COPROPORPHYRINOGEN OXIDASE REVEALS THE STRUCTURAL BASIS OF HEREDITARY COPROPORPHYRIA	VLR	0.21
1EEM	GLUTATHIONE TRANSFERASE FROM HOMO SAPIENS	VLK	0.21
2ONI	CATALYTIC DOMAIN OF THE HUMAN NEDD4-LIKE E3 LIGASE	VLK	0.21
2QNK	CRYSTAL STRUCTURE OF HUMAN 3-HYDROXYANTHRANILATE 3_4-DIOXYGENASE	VLR	0.21

2ZMD	CRYSTAL STRUCTURE OF HUMAN MPS1 CATALYTIC DOMAIN T686A MUTANT IN COMPLEX WITH SP600125 INHIBITOR	VLK	0.21
2BKA	CC3(TIP30)CRYSTAL STRUCURE	VLK	0.21
1OHC	STRUCTURE OF THE PROLINE DIRECTED PHOSPHATASE CDC14	PFR	0.21
2FCB	HUMAN FC GAMMA RECEPTOR IIB ECTODOMAIN (CD32)	VLR	0.21
1TXD	CRYSTAL STRUCTURE OF THE DH/PH DOMAINS OF LEUKEMIA-ASSOCIATED RHOGEF	VLR	0.21
2HE3	CRYSTAL STRUCTURE OF THE SELENOCYSTEINE TO CYSTEINE MUTANT OF HUMAN GLUTATHIONINE PEROXIDASE 2 (GPX2)	PFR	0.21
3GRS	REFINED STRUCTURE OF GLUTATHIONE REDUCTASE AT 1.54 ANGSTROMS RESOLUTION	VLK	0.21
2R99	CRYSTAL STRUCTURE OF CYCLOPHILIN ABH-LIKE DOMAIN OF HUMAN PEPTIDYLPROLYL ISOMERASE E ISOFORM 1	VLR	0.21
1IMV	2.85 A CRYSTAL STRUCTURE OF PEDF	VLR	0.21
2A1X	HUMAN PHYTANOYL-COA 2-HYDROXYLASE IN COMPLEX WITH IRON AND 2-OXOGLUTARATE	PFR	0.22
1MFM	MONOMERIC HUMAN SOD MUTANT F50E/G51E/E133Q AT ATOMIC RESOLUTION	VLK	0.22
1HDO	HUMAN BILIVERDIN IX BETA REDUCTASE: NADP COMPLEX	VLR	0.22
1WL4	HUMAN CYTOSOLIC ACETOACETYL-COA THIOLASE COMPLEXED WITH COA	VLK	0.22
1SMB	CRYSTAL STRUCTURE OF GOLGI-ASSOCIATED PR-1 PROTEIN	VLK	0.22
1WER	RAS-GTPASE-ACTIVATING DOMAIN OF HUMAN P120GAP	VLK	0.22
1VZO	THE STRUCTURE OF THE N-TERMINAL KINASE DOMAIN OF MSK1 REVEALS A NOVEL AUTOINHIBITORY CONFORMATION FOR A DUAL KINASE PROTEIN	VLK	0.22
1Z8D	CRYSTAL STRUCTURE OF HUMAN MUSCLE GLYCOGEN PHOSPHORYLASE A WITH AMP AND GLUCOSE	VPR	0.22
2QDJ	CRYSTAL STRUCTURE OF THE RETINOBLASTOMA PROTEIN N-DOMAIN PROVIDES INSIGHT INTO TUMOR SUPPRESSION_ LIGAND INTERACTION AND HOLOPROTEIN ARCHITECTURE	VLK	0.22
1FA9	HUMAN LIVER GLYCOGEN PHOSPHORYLASE A COMPLEXED WITH AMP	VPR	0.23
2OC3	CRYSTAL STRUCTURE OF THE CATALYTIC DOMAIN OF HUMAN PROTEIN TYROSINE PHOSPHATASE NON-RECEPTOR TYPE 18	VLK	0.23
1UU3	STRUCTURE OF HUMAN PDK1 KINASE DOMAIN IN COMPLEX WITH LY333531	PFR	0.23
2A14	CRYSTAL STRUCTURE OF HUMAN INDOLETHYLAMINE N-METHYLTRANSFERASE WITH SAH	VLK	0.23
1MS6	DIPEPTIDE NITRILE INHIBITOR BOUND TO CATHEPSIN S.	VLK	0.23
2VGE	CRYSTAL STRUCTURE OF THE C-TERMINAL REGION OF HUMAN IASPP	VLR	0.23
1XQJ	3.10 A CRYSTAL STRUCTURE OF MASPIN_ SPACE GROUP I 4 2 2	PFR	0.23
1BY7	HUMAN PLASMINOGEN ACTIVATOR INHIBITOR-2. LOOP (66-98) DELETION MUTANT	PFR	0.23
1RGP	GTPASE-ACTIVATION DOMAIN FROM RHOGAP	VLR	0.23
2OJ9	STRUCTURE OF IGF-1R KINASE DOMAIN COMPLEXED WITH A BENZIMIDAZOLE INHIBITOR	VLR	0.23
1ZGK	1.35 ANGSTROM STRUCTURE OF THE KELCH DOMAIN OF KEAP1	VPR	0.23
2BH9	X-RAY STRUCTURE OF A DELETION VARIANT OF HUMAN GLUCOSE 6-PHOSPHATE DEHYDROGENASE COMPLEXED WITH STRUCTURAL AND COENZYME NADP	VLK	0.23
2EU9	CRYSTAL STRUCTURE OF CLK3	VLK	0.23
2DW4	CRYSTAL STRUCTURE OF HUMAN LSD1 AT 2.3 A RESOLUTION	VLK	0.24
1IAP	CRYSTAL STRUCTURE OF P115RHOGEF RGRGS DOMAIN	VLR	0.24
1Z57	CRYSTAL STRUCTURE OF HUMAN CLK1 IN COMPLEX WITH 10Z-HYMENIALDISINE	PFR	0.24
1KKU	CRYSTAL STRUCTURE OF NUCLEAR HUMAN NICOTINAMIDE MONONUCLEOTIDE ADENYLYLTRANSFERASE	VLR	0.24
3GRS	REFINED STRUCTURE OF GLUTATHIONE REDUCTASE AT 1.54 ANGSTROMS RESOLUTION	VLR	0.24

1U59	CRYSTAL STRUCTURE OF THE ZAP-70 KINASE DOMAIN IN COMPLEX WITH STAUROSPORINE	VLK	0.24
3BI1	X-RAY STRUCTURE OF HUMAN GLUTAMATE CARBOXYPEPTIDASE II (GCPII) IN COMPLEX WITH A TRANSITION STATE ANALOG OF METHOTREXATE-GLU	VLR	0.24
1P14	CRYSTAL STRUCTURE OF A CATALYTIC-LOOP MUTANT OF THE INSULIN RECEPTOR TYROSINE KINASE	VLK	0.24
2VPJ	CRYSTAL STRUCTURE OF THE KELCH DOMAIN OF HUMAN KLHL12	VLR	0.24
1ELV	CRYSTAL STRUCTURE OF THE CATALYTIC DOMAIN OF HUMAN COMPLEMENT C1S PROTEASE	VPR	0.24
2VKQ	CRYSTAL STRUCTURE OF HUMAN CYTOSOLIC 5'-NUCLEOTIDASE III (CN-III_ NT5C3) IN COMPLEX WITH BERYLLIUM TRIFLUORIDE	VLK	0.24
2PL3	HUMAN DEAD-BOX RNA HELICASE DDX10_ DEAD DOMAIN IN COMPLEX WITH ADP	VLR	0.25
3CTZ	STRUCTURE OF HUMAN CYTOSOLIC X-PROLYL AMINOPEPTIDASE	VLR	0.25
2FCB	HUMAN FC GAMMA RECEPTOR IIB ECTODOMAIN (CD32)	VLK	0.25
1FCG	ECTODOMAIN OF HUMAN FC GAMMA RECEPTOR_ FCGRIIA	VLK	0.25
2JC9	CRYSTAL STRUCTURE OF HUMAN CYTOSOLIC 5'-NUCLEOTIDASE II IN COMPLEX WITH ADENOSINE	VLR	0.25
1WDY	CRYSTAL STRUCTURE OF RIBONUCLEASE	VLR	0.26
1T67	CRYSTAL STRUCTURE OF HUMAN HDAC8 COMPLEXED WITH MS-344	VLK	0.26
1LUG	FULL MATRIX ERROR ANALYSIS OF CARBONIC ANHYDRASE	VLK	0.26
2E8A	CRYSTAL STRUCTURE OF THE HUMAN HSP70 ATPASE DOMAIN IN COMPLEX WITH AMP-PNP	VLR	0.26
2C30	CRYSTAL STRUCTURE OF THE HUMAN P21-ACTIVATED KINASE 6	VLR	0.26
2G62	CRYSTAL STRUCTURE OF HUMAN PTPA	VLR	0.26
2IVV	CRYSTAL STRUCTURE OF PHOSPHORYLATED RET TYROSINE KINASE DOMAIN COMPLEXED WITH THE INHIBITOR PP1	VLK	0.26
1BX4	STRUCTURE OF HUMAN ADENOSINE KINASE AT 1.50 ANGSTROMS	VLK	0.26
1S9J	X-RAY STRUCTURE OF THE HUMAN MITOGEN-ACTIVATED PROTEIN KINASE KINASE 1 (MEK1) IN A COMPLEX WITH LIGAND AND MGATP	VLK	0.26
2Q5I	CRYSTAL STRUCTURE OF APO S581L GLYCYL-TRNA SYNTHETASE MUTAN	VLK	0.27
2IJA	HUMAN N-ACETYLTRANSFERASE 1 F125S MUTANT	VLK	0.27
1N11	D34 REGION OF HUMAN ANKYRIN-R AND LINKER	VLK	0.27
2GRY	CRYSTAL STRUCTURE OF THE HUMAN KIF2 MOTOR DOMAIN IN COMPLEX WITH ADP	PFR	0.27
2OCG	CRYSTAL STRUCTURE OF HUMAN VALACYCLOVIR HYDROLASE	VPR	0.27
2B1P	INHIBITOR COMPLEX OF JNK3	VLK	0.27
2GRY	CRYSTAL STRUCTURE OF THE HUMAN KIF2 MOTOR DOMAIN IN COMPLEX WITH ADP	VLK	0.27
2OBD	CRYSTAL STRUCTURE OF CHOLESTERYL ESTER TRANSFER PROTEIN	VLK	0.27
2HEH	CRYSTAL STRUCTURE OF THE KIF2C MOTOR DOMAIN (CASP TARGET)	PFR	0.27
1R5L	CRYSTAL STRUCTURE OF HUMAN ALPHA-TOCOPHEROL TRANSFER PROTEIN BOUND TO ITS LIGAND	VLR	0.28
2BH9	X-RAY STRUCTURE OF A DELETION VARIANT OF HUMAN GLUCOSE 6-PHOSPHATE DEHYDROGENASE COMPLEXED WITH STRUCTURAL AND COENZYME NADP	VLR	0.28
2V7O	CRYSTAL STRUCTURE OF HUMAN CALCIUM-CALMODULIN-DEPENDENT PROTEIN KINASE II GAMMA	VLR	0.28
2Z5Y	CRYSTAL STRUCTURE OF HUMAN MONOAMINE OXIDASE A (G110A) WITH HARMINE	PFR	0.28
2Z6H	CRYSTAL STRUCTURE OF BETA-CATENIN ARMADILLO REPEAT REGION AND ITS C-TERMINAL DOMAIN	VLR	0.28
2C9H	STRUCTURE OF MITOCHONDRIAL BETA-KETOACYL SYNTHASE	VPR	0.28
1WCH	CRYSTAL STRUCTURE OF PTPL1 HUMAN TYROSINE PHOSPHATASE MUTATED IN COLORECTAL CANCER- EVIDENCE FOR A SECOND PHOSPHOTYROSINE SUBSTRATE RECOGNITION POCKET	VLR	0.29

1W0H	CRYSTALLOGRAPHIC STRUCTURE OF THE NUCLEASE DOMAIN OF 3'HEXO A DEDDH FAMILY MEMBER_ BOUND TO RAMP	VLK	0.29
2FOZ	HUMAN ADP-RIBOSYLHYDROLASE 3	VLR	0.29
2IUW	CRYSTAL STRUCTURE OF HUMAN ABH3 IN COMPLEX WITH IRON ION AND 2-OXOGLUTARATE	VLR	0.29
2IC1	CRYSTAL STRUCTURE OF HUMAN CYSTEINE DIOXYGENASE IN COMPLEX WITH SUBSTRATE CYSTEINE	VLR	0.29
1D2S	CRYSTAL STRUCTURE OF THE N-TERMINAL LAMININ G-LIKE DOMAIN OF SHBG IN COMPLEX WITH DIHYDROTESTOSTERONE	VLR	0.29
2Z6O	CRYSTAL STRUCTURE OF THE UFC1_ UFM1 CONJUGATING ENZYME 1	VLK	0.29
3BI1	X-RAY STRUCTURE OF HUMAN GLUTAMATE CARBOXYPEPTIDASE II (GCPII) IN COMPLEX WITH A TRANSITION STATE ANALOG OF METHOTREXATE-GLU	VLR	0.29
1KGD	CRYSTAL STRUCTURE OF THE GUANYLATE KINASE-LIKE DOMAIN OF HUMAN CASK	VLR	0.30
2HC1	ENGINEERED CATALYTIC DOMAIN OF PROTEIN TYROSINE PHOSPHATASE HPTPBETA.	VLR	0.30
2II0	CRYSTAL STRUCTURE OF CATALYTIC DOMAIN OF SON OF SEVENLESS (REM-CDC25) IN THE ABSENCE OF RAS	VLK	0.30
1YCK	CRYSTAL STRUCTURE OF HUMAN PEPTIDOGLYCAN RECOGNITION PROTEIN (PGRP-S)	VPR	0.30
1DT9	THE CRYSTAL STRUCTURE OF HUMAN EUKARYOTIC RELEASE FACTOR ERF1-MECHANISM OF STOP CODON RECOGNITION AND PEPTIDYL-TRNA HYDROLYSIS	VLK	0.30
2UZ9	HUMAN GUANINE DEAMINASE (GUAD) IN COMPLEX WITH ZINC AND ITS PRODUCT XANTHINE.	VLR	0.30
2YV8	CRYSTAL STRUCTURE OF N-TERMINAL DOMAIN OF HUMAN GALECTIN-8	VLK	0.30
1KXU	CYCLIN H_ A POSITIVE REGULATORY SUBUNIT OF CDK ACTIVATING KINASE	VLK	0.30
1M8Z	CRYSTAL STRUCTURE OF A PUMILIO-HOMOLOGY DOMAIN	VLK	0.30
2NNJ	CYP2C8DH COMPLEXED WITH FELODIPINE	VLK	0.30
1TDH	CRYSTAL STRUCTURE OF HUMAN ENDONUCLEASE VIII-LIKE 1 (NEIL1)	VLR	0.30
2VPJ	CRYSTAL STRUCTURE OF THE KELCH DOMAIN OF HUMAN KLHL12	VLR	0.31
1CB0	STRUCTURE OF HUMAN 5'-DEOXY-5'-METHYLTHIOADENOSINE PHOSPHORYLASE AT 1.7 A RESOLUTION	VLK	0.31
2Z7R	CRYSTAL STRUCTURE OF THE N-TERMINAL KINASE DOMAIN OF HUMAN RSK1 BOUND TO STAUROSPORINE	VLK	0.31
1ORE	HUMAN ADENINE PHOSPHORIBOSYLTRANSFERASE	VLK	0.31
2JBO	PROTEIN KINASE MK2 IN COMPLEX WITH AN INHIBITOR (CRYSTAL FORM-1_ SOAKING)	VLK	0.31
2HE7	FERM DOMAIN OF EPB41L3 (DAL-1)	VLK	0.31
2E3N	CRYSTAL STRUCTURE OF CERT START DOMAIN IN COMPLEX WITH C6-CERAMIDE (P212121)	VLR	0.31
1UCH	DEUBIQUITINATING ENZYME UCH-L3 (HUMAN) AT 1.8 ANGSTROM RESOLUTION	VPR	0.31
2DYL	CRYSTAL STRUCTURE OF HUMAN MITOGEN-ACTIVATED PROTEIN KINASE KINASE 7 ACTIVATED MUTANT (S287D_ T291D)	VLK	0.31
2OBV	CRYSTAL STRUCTURE OF THE HUMAN S-ADENOSYLMETHIONINE SYNTHETASE 1 IN COMPLEX WITH THE PRODUCT	VPR	0.32
1M7B	CRYSTAL STRUCTURE OF RND3/RHOE: FUNCTIONAL IMPLICATIONS	VLK	0.32
1BIO	HUMAN COMPLEMENT FACTOR D IN COMPLEX WITH ISATOIC ANHYDRIDE INHIBITOR	VLR	0.32
1Z32	STRUCTURE-FUNCTION RELATIONSHIPS IN HUMAN SALIVARY ALPHA-AMYLASE: ROLE OF AROMATIC RESIDUES	PFR	0.33
2H58	CRYSTAL STRUCTURE OF THE KIFC3 MOTOR DOMAIN IN COMPLEX WITH ADP	PFR	0.33
2CW9	CRYSTAL STRUCTURE OF HUMAN TIM44 C-TERMINAL DOMAIN	VLR	0.33
1TDH	CRYSTAL STRUCTURE OF HUMAN ENDONUCLEASE VIII-LIKE 1 (NEIL1)	VPR	0.33

1RNE	THE CRYSTAL STRUCTURE OF RECOMBINANT GLYCOSYLATED HUMAN RENIN ALONE AND IN COMPLEX WITH A TRANSITION STATE ANALOG INHIBITOR	VLK	0.33
2ALR	ALDEHYDE REDUCTASE	VPR	0.34
1FW1	GLUTATHIONE TRANSFERASE ZETA/MALEYLACETOACETATE ISOMERASE	VLK	0.34
1JDN	CRYSTAL STRUCTURE OF HORMONE RECEPTOR	VLR	0.34
2UZ9	HUMAN GUANINE DEAMINASE (GUAD) IN COMPLEX WITH ZINC AND ITS PRODUCT XANTHINE.	VLK	0.34
1S1E	CRYSTAL STRUCTURE OF KV CHANNEL-INTERACTING PROTEIN 1 (KCHIP-1)	VLK	0.35
1KT0	STRUCTURE OF THE LARGE FKBP-LIKE PROTEIN_ FKBP51_ INVOLVED IN STEROID RECEPTOR COMPLEXES	VLK	0.35
3BCH	CRYSTAL STRUCTURE OF THE HUMAN LAMININ RECEPTOR PRECURSOR	VLK	0.35
2I0Y	CFMS TYROSINE KINASE (FGF KID) IN COMPLEX WITH AN ARYLAMIDE INHIBITOR	VLK	0.35
1EWF	THE 1.7 ANGSTROM CRYSTAL STRUCTURE OF BPI	VLK	0.35
2I1M	CFMS TYROSINE KINASE (TIE2 KID) IN COMPLEX WITH AN ARYLAMIDE INHIBITOR	VLK	0.35
2VAF	CRYSTAL STRUCTURE OF HUMAN CARDIAC CALSEQUESTRIN	VLK	0.35
2IPX	HUMAN FIBRILLARIN	PFR	0.35
2OGV	CRYSTAL STRUCTURE OF THE AUTOINHIBITED HUMAN C-FMS KINASE DOMAIN	VLK	0.36
1WOJ	CRYSTAL STRUCTURE OF HUMAN PHOSPHODIESTERASE	VLK	0.36
2I7Q	CRYSTAL STRUCTURE OF HUMAN CHOLINE KINASE A	VLR	0.36
1W1D	CRYSTAL STRUCTURE OF THE PDK1 PLECKSTRIN HOMOLOGY (PH) DOMAIN BOUND TO INOSITOL (1_3_4_5)-TETRAKISPHOSPHATE	VLK	0.36
2HGS	HUMAN GLUTATHIONE SYNTHETASE	VLK	0.37
2YT4	CRYSTAL STRUCTURE OF HUMAN DGCR8 CORE	VLK	0.37
2JEO	CRYSTAL STRUCTURE OF HUMAN URIDINE-CYTIDINE KINASE 1	VLR	0.37
2ALR	ALDEHYDE REDUCTASE	VPR	0.37
2GY5	TIE2 LIGAND-BINDING DOMAIN CRYSTAL STRUCTURE	VPR	0.38
1BYG	KINASE DOMAIN OF HUMAN C-TERMINAL SRC KINASE (CSK) IN COMPLEX WITH INHIBITOR STAUROSPORINE	VPR	0.38
2FN4	THE CRYSTAL STRUCTURE OF HUMAN RAS-RELATED PROTEIN_ RRAS_ IN THE GDP-BOUND STATE	VPR	0.38
2NR8	CRYSTAL STRUCTURE OF THE HUMAN KIF9 MOTOR DOMAIN IN COMPLEX WITH ADP	PFR	0.39
1QPC	STRUCTURAL ANALYSIS OF THE LYMPHOCYTE-SPECIFIC KINASE LCK IN COMPLEX WITH NON-SELECTIVE AND SRC FAMILY SELECTIVE KINASE INHIBITORS	VPR	0.40
1TA0	THREE-DIMENSIONAL STRUCTURE OF A RNA-POLYMERASE II BINDING PROTEIN WITH ASSOCIATED LIGAND.	VLR	0.40
1LZJ	GLYCOSYLTRANSFERASE B + UDP + H ANTIGEN ACCEPTOR	VLR	0.40
2QTZ	CRYSTAL STRUCTURE OF THE NADP+-BOUND FAD-CONTAINING FNR- LIKE MODULE OF HUMAN METHIONINE SYNTHASE REDUCTASE	VLR	0.40
3CKK	CRYSTAL STRUCTURE OF HUMAN METHYLTRANSFERASE-LIKE PROTEIN 1	VLR	0.40
1WAK	X-RAY STRUCTURE OF SRPK1	VPR	0.41
1L2H	CRYSTAL STRUCTURE OF INTERLEUKIN 1-BETA F42W/W120F MUTANT	VLK	0.41
1B3J	STRUCTURE OF THE MHC CLASS I HOMOLOG MIC-A_ A GAMMADELTA T CELL LIGAND	VLR	0.41
2OO8	SYNTHESIS_ STRUCTURAL ANALYSIS_ AND SAR STUDIES OF TRIAZINE DERIVATIVES AS POTENT_ SELECTIVE TIE-2 INHIBITORS	VLK	0.41
2J0I	CRYSTAL STRUCTURE OF THE HUMAN P21-ACTIVATED KINASE 4	VPR	0.42
3COI	CRYSTAL STRUCTURE OF P38DELTA KINASE	PFR	0.42
2O36	CRYSTAL STRUCTURE OF ENGINEERED THIMET OLIGOPEPTIDASE WITH NEUROLYSIN SPECIFICITY IN NEUROTENSIN CLEAVAGE SITE	VPR	0.43

2AEX	THE 1.58A CRYSTAL STRUCTURE OF HUMAN COPROPORPHYRINOGEN OXIDASE REVEALS THE STRUCTURAL BASIS OF HEREDITARY COPROPORPHYRIA	VLK	0.43
2BH9	X-RAY STRUCTURE OF A DELETION VARIANT OF HUMAN GLUCOSE 6-PHOSPHATE DEHYDROGENASE COMPLEXED WITH STRUCTURAL AND COENZYME NADP	VPR	0.43
1QMN	ALPHA1-ANTICHYMOTRYPSIN SERPIN IN THE DELTA CONFORMATION (PARTIAL LOOP INSERTION)	VLK	0.43
1ZED	ALKALINE PHOSPHATASE FROM HUMAN PLACENTA IN COMPLEX WITH P-NITROPHENYL-PHOSPHONATE	VLK	0.44
1LZJ	GLYCOSYLTRANSFERASE B + UDP + H ANTIGEN ACCEPTOR	VPR	0.46
2A4D	STRUCTURE OF THE HUMAN UBIQUITIN-CONJUGATING ENZYME E2 VARIANT 1 (UEV-1)	VPR	0.46
1W0H	CRYSTALLOGRAPHIC STRUCTURE OF THE NUCLEASE DOMAIN OF 3'HEXO A DEDDH FAMILY MEMBER_ BOUND TO RAMP	VPR	0.48
1S1P	CRYSTAL STRUCTURES OF PROSTAGLANDIN D2 11-KETOREDUCTASE (AKR1C3) IN COMPLEX WITH THE NON-STEROIDAL ANTI-INFLAMMATORY DRUGS FLUFENAMIC ACID AND INDOMETHACIN	VPR	0.51
2QYM	CRYSTAL STRUCTURE OF UNLIGANDED PDE4C2	VPR	0.51
2IC1	CRYSTAL STRUCTURE OF HUMAN CYSTEINE DIOXYGENASE IN COMPLEX WITH SUBSTRATE CYSTEINE	VLK	0.53
1YO8	STRUCUTURE OF THE C-TERMINAL DOMAIN OF HUMAN THROMBOSPONDIN 2	VPR	0.54
1FNH	CRYSTAL STRUCTURE OF HEPARIN AND INTEGRIN BINDING SEGMENT OF HUMAN FIBRONECTIN	VPR	0.56
1UZE	COMPLEX OF THE ANTI-HYPERTENSIVE DRUG ENALAPRILAT AND THE HUMAN TESTICULAR ANGIOTENSIN I-CONVERTING ENZYME	VPR	0.57
2AWF	STRUCTURE OF HUMAN UBIQUITIN-CONJUGATING ENZYME E2 G1	VPR	0.57
2GF9	CRYSTAL STRUCTURE OF HUMAN RAB3D IN COMPLEX WITH GDP	VPR	0.58
1NN6	HUMAN PRO-CHYMASE	VLK	0.58
1DHS	CRYSTAL STRUCTURE OF THE NAD COMPLEX OF HUMAN DEOXYHYPUSINE SYNTHASE	VLK	0.63
1HVF	STRUCTURAL AND ELECTROPHYSIOLOGICAL ANALYSIS OF ANNEXIN V MUTANTS. MUTAGENESIS OF HUMAN ANNEXIN V_ AN IN VITRO VOLTAGE-GATED CALCIUM CHANNEL_ PROVIDES INFORMATION ABOUT THE STRUCTURAL FEATURES OF THE ION PATHWAY_ THE VOLTAGE SENSOR AND THE ION SELECTIVITY FILTER	VLR	0.63
2A5J	CRYSTAL STRUCTURE OF HUMAN RAB2B	VPR	0.66
2QT1	HUMAN NICOTINAMIDE RIBOSIDE KINASE 1 IN COMPLEX WITH NICOTINAMIDE RIBOSIDE	VPR	0.75
2PNY	STRUCTURE OF HUMAN ISOPENTENYL-DIPHOSPHATE DELTA-ISOMERASE	VPR	0.76
2RK3	STRUCTURE OF A104T DJ-1	VLK	0.79
1YRV	NOVEL UBIQUITIN-CONJUGATING ENZYME	VPR	0.92
2HQ6	STRUCTURE OF THE CYCLOPHILIN_CECYP16-LIKE DOMAIN OF THE SEROLOGICALLY DEFINED COLON CANCER ANTIGEN 10 FROM HOMO SAPIENS	VPR	1.01
1LBD	LIGAND-BINDING DOMAIN OF THE HUMAN NUCLEAR RECEPTOR RXR-ALPHA	VPR	2.00
1MX3	CRYSTAL STRUCTURE OF CTBP DEHYDROGENASE CORE HOLO FORM	VPR	2.00
1P5J	CRYSTAL STRUCTURE ANALYSIS OF HUMAN SERINE DEHYDRATASE	VPR	2.00
1PME	STRUCTURE OF PENTA MUTANT HUMAN ERK2 MAP KINASE COMPLEXED WITH A SPECIFIC INHIBITOR OF HUMAN P38 MAP KINASE	VPR	2.00
1Q1C	CRYSTAL STRUCTURE OF N(1-260) OF HUMAN FKBP52	VPR	2.00
1QKM	HUMAN OESTROGEN RECEPTOR BETA LIGAND-BINDING DOMAIN IN COMPLEX WITH PARTIAL AGONIST GENISTEIN	VLR	2.00
1RGP	GTPASE-ACTIVATION DOMAIN FROM RHOGAP	VLK	2.00
1TBF	CATALYTIC DOMAIN OF HUMAN PHOSPHODIESTERASE 5A IN COMPLEX WITH SILDENAFIL	VPR	2.00

1XAP	STRUCTURE OF THE LIGAND BINDING DOMAIN OF THE RETINOIC ACID RECEPTOR BETA	VPR	2.00
1XJD	CRYSTAL STRUCTURE OF PKC-THETA COMPLEXED WITH STAUROSPORINE AT 2A RESOLUTION	PFR	2.00
1ZD9	STRUCTURE OF HUMAN ADP-RIBOSYLATION FACTOR-LIKE 10B	VPR	2.00
1ZIV	CATALYTIC DOMAIN OF HUMAN CALPAIN-9	VPR	2.00
1ZJH	STRUCTURE OF HUMAN MUSCLE PYRUVATE KINASE (PKM2)	VPR	2.00
2B3H	CRYSTAL STRUCTURE OF HUMAN METHIONINE AMINOPEPTIDASE TYPE I WITH A THIRD COBALT IN THE ACTIVE SITE	VPR	2.00
2B6H	STRUCTURE OF HUMAN ADP-RIBOSYLATION FACTOR 5	VPR	2.00
2B9E	HUMAN NSUN5 PROTEIN	VPR	2.00
2EW1	CRYSTAL STRUCTURE OF RAB30 IN COMPLEX WITH A GTP ANALOGUE	VPR	2.00
2FAU	CRYSTAL STRUCTURE OF HUMAN VPS26	VPR	2.00
2FG5	CRYSTAL STRUCTURE OF HUMAN RAB31 IN COMPLEX WITH A GTP ANALOGUE	VPR	2.00
2FK9	HUMAN PROTEIN KINASE C_ ETA	VPR	2.00
2FOL	CRYSTAL STRUCTURE OF HUMAN RAB1A IN COMPLEX WITH GDP	VPR	2.00
2FYT	HUMAN HMT1 HNRNP METHYLTRANSFERASE-LIKE 3 (S. CEREVISIAE) PROTEIN	VPR	2.00
2GEE	CRYSTAL STRUCTURE OF HUMAN TYPE III FIBRONECTIN EXTRADOMAIN B AND DOMAIN 8	VPR	2.00
2GFO	STRUCTURE OF THE CATALYTIC DOMAIN OF HUMAN UBIQUITIN CARBOXYL-TERMINAL HYDROLASE 8	VPR	2.00
2GRY	CRYSTAL STRUCTURE OF THE HUMAN KIF2 MOTOR DOMAIN IN COMPLEX WITH ADP	VPR	2.00
2GW2	CRYSTAL STRUCTURE OF THE PEPTIDYL-PROLYL ISOMERASE DOMAIN OF HUMAN CYCLOPHILIN G	VPR	2.00
2H17	STRUCTURE OF HUMAN ADP-RIBOSYLATION FACTOR-LIKE 5 (ARL5) (CASP TARGET)	VPR	2.00
2HEH	CRYSTAL STRUCTURE OF THE KIF2C MOTOR DOMAIN (CASP TARGET)	VPR	2.00
2HI4	CRYSTAL STRUCTURE OF HUMAN MICROSOMAL P450 1A2 IN COMPLEX WITH ALPHA-NAPHTHOFLAVONE	VLK	2.00
2HZ6	THE CRYSTAL STRUCTURE OF HUMAN IRE1-ALPHA LUMINAL DOMAIN	VPR	2.00
2I7A	DOMAIN IV OF HUMAN CALPAIN 13	VPR	2.00
2I7Q	CRYSTAL STRUCTURE OF HUMAN CHOLINE KINASE A	VPR	2.00
2IL1	CRYSTAL STRUCTURE OF A PREDICTED HUMAN GTPASE IN COMPLEX WITH GDP	VPR	2.00
2IUW	CRYSTAL STRUCTURE OF HUMAN ABH3 IN COMPLEX WITH IRON ION AND 2 OXOGLUTARATE	VPR	2.00
2JC9	CRYSTAL STRUCTURE OF HUMAN CYTOSOLIC 5'-NUCLEOTIDASE II IN COMPLEX WITH ADENOSINE	VPR	2.00
2NR8	CRYSTAL STRUCTURE OF THE HUMAN KIF9 MOTOR DOMAIN IN COMPLEX WITH ADP	VPR	2.00
2NR8	CRYSTAL STRUCTURE OF THE HUMAN KIF9 MOTOR DOMAIN IN COMPLEX WITH ADP	VLK	2.00
2OIL	CRYSTAL STRUCTURE OF HUMAN RAB25 IN COMPLEX WITH GDP	VPR	2.00
2QLU	CRYSTAL STRUCTURE OF ACTIVIN RECEPTOR TYPE II KINASE DOMAIN FROM HUMAN	VPR	2.00
2QQ5	CRYSTAL STRUCTURE OF HUMAN SDR FAMILY MEMBER 1	VLK	2.00
2QTZ	CRYSTAL STRUCTURE OF THE NADP+-BOUND FAD-CONTAINING FNR- LIKE MODULE OF HUMAN METHIONINE SYNTHASE REDUCTASE	VLK	2.00
2REP	CRYSTAL STRUCTURE OF THE MOTOR DOMAIN OF HUMAN KINESIN FAMILY MEMBER C1	VPR	2.00
2VKQ	CRYSTAL STRUCTURE OF HUMAN CYTOSOLIC 5'-NUCLEOTIDASE III (CN-III_ NT5C3) IN COMPLEX WITH BERYLLIUM TRIFLUORIDE	VPR	2.00
2ZMD	CRYSTAL STRUCTURE OF HUMAN MPS1 CATALYTIC DOMAIN T686A MUTANT IN COMPLEX WITH SP600125 INHIBITOR	VPR	2.00
3BCH	CRYSTAL STRUCTURE OF THE HUMAN LAMININ RECEPTOR PRECURSOR	VPR	2.00

3BD9	HUMAN 3-O-SULFOTRANSFERASE ISOFORM 5 WITH BOUND PAP	VPR	2.00
	**kallikrein-related peptidase 6**		
2QMJ	CRYSTRAL STRUCTURE OF THE N-TERMINAL SUBUNIT OF HUMAN MALTASE-GLUCOAMYLASE IN COMPLEX WITH ACARBOSE	FSR	0.11
1JYD	CRYSTAL STRUCTURE OF RECOMBINANT HUMAN SERUM RETINOL-BINDING PROTEIN AT 1.7 A RESOLUTION	FSR	0.15
2B3X	STRUCTURE OF AN ORTHORHOMBIC CRYSTAL FORM OF HUMAN CYTOSOLIC ACONITASE (IRP1)	GPK	0.15
2I7V	STRUCTURE OF HUMAN CPSF-73	FSR	0.15
1YVJ	CRYSTAL STRUCTURE OF THE JAK3 KINASE DOMAIN IN COMPLEX WITH A STAUROSPORINE ANALOGUE	FSR	0.16
1XKS	THE CRYSTAL STRUCTURE OF THE N-TERMINAL DOMAIN OF NUP133 REVEALS A BETA-PROPELLER FOLD COMMON TO SEVERAL NUCLEOPORINS	FSR	0.16
1JNX	CRYSTAL STRUCTURE OF THE BRCT REPEAT REGION FROM THE BREAST CANCER ASSOCIATED PROTEIN_ BRCA1	GPK	0.17
1R03	CRYSTAL STRUCTURE OF A HUMAN MITOCHONDRIAL FERRITIN	FSR	0.17
2A2K	CRYSTAL STRUCTURE OF AN ACTIVE SITE MUTANT_ C473S_ OF CDC25B PHOSPHATASE CATALYTIC DOMAIN	GPR	0.19
1F5N	HUMAN GUANYLATE BINDING PROTEIN-1 IN COMPLEX WITH THE GTP ANALOGUE_ GMPPNP.	GPR	0.19
2VGE	CRYSTAL STRUCTURE OF THE C-TERMINAL REGION OF HUMAN IASPP	VPR	0.20
1TXU	CRYSTAL STRUCTURE OF THE VPS9 DOMAIN OF RABEX-5	VPR	0.20
1ZGK	1.35 ANGSTROM STRUCTURE OF THE KELCH DOMAIN OF KEAP1	VPR	0.20
1SIQ	THE CRYSTAL STRUCTURE AND MECHANISM OF HUMAN GLUTARYL-COA DEHYDROGENASE	QAR	0.20
2VR2	HUMAN DIHYDROPYRIMIDINASE	FSR	0.20
1UPV	CRYSTAL STRUCTURE OF THE HUMAN LIVER X RECEPTOR BETA LIGAND BINDING DOMAIN IN COMPLEX WITH A SYNTHETIC AGONIST	FSR	0.20
1C25	HUMAN CDC25A CATALYTIC DOMAIN	GPR	0.21
1YHV	CRYSTAL STRUCTURE OF PAK1 KINASE DOMAIN WITH TWO POINT MUTATIONS (K299R_ T423E)	GPK	0.22
2I7Q	CRYSTAL STRUCTURE OF HUMAN CHOLINE KINASE A	QAR	0.22
1Z8D	CRYSTAL STRUCTURE OF HUMAN MUSCLE GLYCOGEN PHOSPHORYLASE A WITH AMP AND GLUCOSE	VPR	0.22
1FA9	HUMAN LIVER GLYCOGEN PHOSPHORYLASE A COMPLEXED WITH AMP	VPR	0.23
1YCK	CRYSTAL STRUCTURE OF HUMAN PEPTIDOGLYCAN RECOGNITION PROTEIN (PGRP-S)	QAR	0.23
1ZGK	1.35 ANGSTROM STRUCTURE OF THE KELCH DOMAIN OF KEAP1	VPR	0.23
2JC9	CRYSTAL STRUCTURE OF HUMAN CYTOSOLIC 5'-NUCLEOTIDASE II IN COMPLEX WITH ADENOSINE	GPK	0.23
1SO7	MALTOSE-INDUCED STRUCTURE OF THE HUMAN CYTOLSOLIC SIALIDASE NEU2	QAR	0.24
2I7Q	CRYSTAL STRUCTURE OF HUMAN CHOLINE KINASE A	GPK	0.24
1ELV	CRYSTAL STRUCTURE OF THE CATALYTIC DOMAIN OF HUMAN COMPLEMENT C1S PROTEASE	VPR	0.24
1UZE	COMPLEX OF THE ANTI-HYPERTENSIVE DRUG ENALAPRILAT AND THE HUMAN TESTICULAR ANGIOTENSIN I-CONVERTING ENZYME	FSR	0.24
2DH2	CRYSTAL STRUCTURE OF HUMAN ED-4F2HC	QAR	0.25
1CZA	MUTANT MONOMER OF RECOMBINANT HUMAN HEXOKINASE TYPE I COMPLEXED WITH GLUCOSE_ GLUCOSE-6-PHOSPHATE_ AND ADP	FSR	0.25
1LCT	STRUCTURE OF THE RECOMBINANT N-TERMINAL LOBE OF HUMAN LACTOFERRIN AT 2.0 ANGSTROMS RESOLUTION	FSR	0.26
2HI4	CRYSTAL STRUCTURE OF HUMAN MICROSOMAL P450 1A2 IN COMPLEX WITH ALPHA-NAPHTHOFLAVONE	QAR	0.26
1R03	CRYSTAL STRUCTURE OF A HUMAN MITOCHONDRIAL FERRITIN	FSR	0.27

1IAT	CRYSTAL STRUCTURE OF HUMAN PHOSPHOGLUCOSE ISOMERASE/NEUROLEUKIN/AUTOCRINE MOTILITY FACTOR/MATURATION FACTOR	GPR	0.27
1ALU	HUMAN INTERLEUKIN-6	QAR	0.27
2OCG	CRYSTAL STRUCTURE OF HUMAN VALACYCLOVIR HYDROLASE	VPR	0.27
2B1P	INHIBITOR COMPLEX OF JNK3	QAR	0.27
2JBO	PROTEIN KINASE MK2 IN COMPLEX WITH AN INHIBITOR (CRYSTAL FORM-1_ SOAKING)	FSR	0.27
1X9D	CRYSTAL STRUCTURE OF HUMAN CLASS I ALPHA-1_2-MANNOSIDASE IN COMPLEX WITH THIO-DISACCHARIDE SUBSTRATE ANALOGUE	FSR	0.28
1M6I	CRYSTAL STRUCTURE OF APOPTOSIS INDUCING FACTOR (AIF)	QAR	0.28
1KT0	STRUCTURE OF THE LARGE FKBP-LIKE PROTEIN_ FKBP51_ INVOLVED IN STEROID RECEPTOR COMPLEXES	GPR	0.28
2C9H	STRUCTURE OF MITOCHONDRIAL BETA-KETOACYL SYNTHASE	VPR	0.28
2B69	CRYSTAL STRUCTURE OF HUMAN UDP-GLUCORONIC ACID DECARBOXYLAS	GPR	0.28
1ND7	CONFORMATIONAL FLEXIBILITY UNDERLIES UBIQUITIN LIGATION MEDIATED BY THE WWP1 HECT DOMAIN E3 LIGASE	FSR	0.28
2JDF	HUMAN GAMMA-B CRYSTALLIN	FSR	0.29
2JHM	STRUCTURE OF GLOBULAR HEADS OF M-FICOLIN AT NEUTRAL PH	GPR	0.29
1YCK	CRYSTAL STRUCTURE OF HUMAN PEPTIDOGLYCAN RECOGNITION PROTEIN (PGRP-S)	VPR	0.30
1UOU	CRYSTAL STRUCTURE OF HUMAN THYMIDINE PHOSPHORYLASE IN COMPLEX WITH A SMALL MOLECULE INHIBITOR	QAR	0.30
2Z6O	CRYSTAL STRUCTURE OF THE UFC1_ UFM1 CONJUGATING ENZYME 1	GPR	0.30
1W7L	CRYSTAL STRUCTURE OF HUMAN KYNURENINE AMINOTRANSFERASE I	FSR	0.30
1KO9	NATIVE STRUCTURE OF THE HUMAN 8-OXOGUANINE DNA GLYCOSYLASE HOGG1	GPR	0.30
2B6H	STRUCTURE OF HUMAN ADP-RIBOSYLATION FACTOR 5	FSR	0.30
2QTZ	CRYSTAL STRUCTURE OF THE NADP+-BOUND FAD-CONTAINING FNR- LIKE MODULE OF HUMAN METHIONINE SYNTHASE REDUCTASE	FSR	0.31
2FUE	HUMAN ALPHA-PHOSPHOMANNOMUTASE 1 WITH D-MANNOSE 1-PHOSPHATE AND MG2+ COFACTOR BOUND	FSR	0.31
1UCH	DEUBIQUITINATING ENZYME UCH-L3 (HUMAN) AT 1.8 ANGSTROM RESOLUTION	VPR	0.31
1M9I	CRYSTAL STRUCTURE OF PHOSPHORYLATION-MIMICKINGMUTANT T356D OF ANNEXIN VI	QAR	0.31
2F1W	CRYSTAL STRUCTURE OF THE TRAF-LIKE DOMAIN OF HAUSP/USP7	FSR	0.32
2OBV	CRYSTAL STRUCTURE OF THE HUMAN S-ADENOSYLMETHIONINE SYNTHETASE 1 IN COMPLEX WITH THE PRODUCT	VPR	0.32
1GS9	APOLIPOPROTEIN E4_ 22K DOMAIN	QAR	0.32
1QIB	CRYSTAL STRUCTURE OF GELATINASE A CATALYTIC DOMAIN	FSR	0.33
2DW5	CRYSTAL STRUCTURE OF HUMAN PEPTIDYLARGININE DEIMINASE 4 IN COMPLEX WITH N-ALPHA-BENZOYL-N5-(2-FLUORO-1-IMINOETHYL)-L-ORNITHINE AMIDE	GPK	0.33
1TDH	CRYSTAL STRUCTURE OF HUMAN ENDONUCLEASE VIII-LIKE 1 (NEIL1)	VPR	0.33
1CIZ	X-RAY STRUCTURE OF HUMAN STROMELYSIN CATALYTIC DOMAIN COMPLEXES WITH NON-PEPTIDE INHIBITORS: IMPLICATION FOR INHIBITOR SELECTIVITY	FSR	0.33
3BZH	CRYSTAL STRUCTURE OF HUMAN UBIQUITIN-CONJUGATING ENZYME E2 E1	GPK	0.34
2ALR	ALDEHYDE REDUCTASE	VPR	0.34
1TQN	CRYSTAL STRUCTURE OF HUMAN MICROSOMAL P450 3A4	GPR	0.34
1YPV	STRUCTURE OF HUMAN THYMIDYLATE SYNTHASE AT LOW SALT CONDITIONS	QAR	0.34
1ZVD	REGULATION OF SMURF2 UBIQUITIN LIGASE ACTIVITY BY ANCHORING THE E2 TO THE HECT DOMAIN	GPR	0.34
1K04	CRYSTAL STRUCTURE OF THE FOCAL ADHESION TARGETING DOMAIN OF FOCAL ADHESION KINASE	QAR	0.34
2C9H	STRUCTURE OF MITOCHONDRIAL BETA-KETOACYL SYNTHASE	FSR	0.34

1MFM	MONOMERIC HUMAN SOD MUTANT F50E/G51E/E133Q AT ATOMIC RESOLUTION	GPK	0.34
1UZE	COMPLEX OF THE ANTI-HYPERTENSIVE DRUG ENALAPRILAT AND THE HUMAN TESTICULAR ANGIOTENSIN I-CONVERTING ENZYME	QAR	0.35
2HI4	CRYSTAL STRUCTURE OF HUMAN MICROSOMAL P450 1A2 IN COMPLEX WITH ALPHA-NAPHTHOFLAVONE	GPR	0.35
1ILK	INTERLEUKIN-10 CRYSTAL STRUCTURE REVEALS THE FUNCTIONAL DIMER WITH AN UNEXPECTED TOPOLOGICAL SIMILARITY TO INTERFERON GAMMA	FSR	0.35
1NUF	ROLE OF CALCIUM IONS IN THE ACTIVATION AND ACTIVITY OF THE TRANSGLUTAMINASE 3 ENZYME	GPK	0.35
1CJM	HUMAN SULT1A3 WITH SULFATE BOUND	QAR	0.37
2NZL	CRYSTAL STRUCTURE OF HUMAN HYDROXYACID OXIDASE 1	FSR	0.37
2CBZ	STRUCTURE OF THE HUMAN MULTIDRUG RESISTANCE PROTEIN 1 NUCLEOTIDE BINDING DOMAIN 1	GPK	0.37
2ALR	ALDEHYDE REDUCTASE	VPR	0.37
2F21	HUMAN PIN1 FIP MUTANT	FSR	0.37
2GY5	TIE2 LIGAND-BINDING DOMAIN CRYSTAL STRUCTURE	VPR	0.38
1D2S	CRYSTAL STRUCTURE OF THE N-TERMINAL LAMININ G-LIKE DOMAIN OF SHBG IN COMPLEX WITH DIHYDROTESTOSTERONE	GPR	0.38
1BYG	KINASE DOMAIN OF HUMAN C-TERMINAL SRC KINASE (CSK) IN COMPLEX WITH INHIBITOR STAUROSPORINE	VPR	0.38
2FN4	THE CRYSTAL STRUCTURE OF HUMAN RAS-RELATED PROTEIN_ RRAS_ IN THE GDP-BOUND STATE	VPR	0.38
2FCB	HUMAN FC GAMMA RECEPTOR IIB ECTODOMAIN (CD32)	FSR	0.39
1QPC	STRUCTURAL ANALYSIS OF THE LYMPHOCYTE-SPECIFIC KINASE LCK IN COMPLEX WITH NON-SELECTIVE AND SRC FAMILY SELECTIVE KINASE INHIBITORS	VPR	0.40
1QCY	THE CRYSTAL STRUCTURE OF THE I-DOMAIN OF HUMAN INTEGRIN ALPHA1BETA1	GPK	0.40
3CFW	L-SELECTIN LECTIN AND EGF DOMAINS	FSR	0.40
2B69	CRYSTAL STRUCTURE OF HUMAN UDP-GLUCORONIC ACID DECARBOXYLAS	GPR	0.40
2A91	CRYSTAL STRUCTURE OF ERBB2 DOMAINS 1-3	GPK	0.41
1N26	CRYSTAL STRUCTURE OF THE EXTRA-CELLULAR DOMAINS OF HUMAN INTERLEUKIN-6 RECEPTOR ALPHA CHAIN	GPR	0.41
2F1W	CRYSTAL STRUCTURE OF THE TRAF-LIKE DOMAIN OF HAUSP/USP7	FSR	0.41
1WAK	X-RAY STRUCTURE OF SRPK1	VPR	0.41
1LS6	HUMAN SULT1A1 COMPLEXED WITH PAP AND P-NITROPHENOL	QAR	0.41
2QXI	HIGH RESOLUTION STRUCTURE OF HUMAN KALLIKREIN 7 IN COMPLEX WITH SUC-ALA-ALA-PRO-PHE-CHLOROMETHYLKETONE	QAR	0.41
2J0I	CRYSTAL STRUCTURE OF THE HUMAN P21-ACTIVATED KINASE 4	VPR	0.42
2O36	CRYSTAL STRUCTURE OF ENGINEERED THIMET OLIGOPEPTIDASE WITH NEUROLYSIN SPECIFICITY IN NEUROTENSIN CLEAVAGE SITE	VPR	0.43
2BH9	X-RAY STRUCTURE OF A DELETION VARIANT OF HUMAN GLUCOSE 6-PHOSPHATE DEHYDROGENASE COMPLEXED WITH STRUCTURAL AND COENZYME NADP	VPR	0.43
1NM8	STRUCTURE OF HUMAN CARNITINE ACETYLTRANSFERASE: MOLECULAR BASIS FOR FATTY ACYL TRANSFER	GPK	0.43
1E5W	STRUCTURE OF ISOLATED FERM DOMAIN AND FIRST LONG HELIX OF MOESIN	QAR	0.44
1S31	CRYSTAL STRUCTURE ANALYSIS OF THE HUMAN TUB PROTEIN (ISOFORM A) SPANNING RESIDUES 289 THROUGH 561	GPR	0.44
1HDO	HUMAN BILIVERDIN IX BETA REDUCTASE: NADP COMPLEX	GPR	0.45
1EQF	CRYSTAL STRUCTURE OF THE DOUBLE BROMODOMAIN MODULE FROM HUMAN TAFII250	GPK	0.45
1MZA	CRYSTAL STRUCTURE OF HUMAN PRO-GRANZYME K	FSR	0.46
1LZJ	GLYCOSYLTRANSFERASE B + UDP + H ANTIGEN ACCEPTOR	VPR	0.46

2A4D	STRUCTURE OF THE HUMAN UBIQUITIN-CONJUGATING ENZYME E2 VARIANT 1 (UEV-1)	VPR	0.46
1IMJ	CRYSTAL STRUCTURE OF THE HUMAN CCG1/TAFII250-INTERACTING FACTOR B (CIB)	QAR	0.47
1W0H	CRYSTALLOGRAPHIC STRUCTURE OF THE NUCLEASE DOMAIN OF 3'HEXO A DEDDH FAMILY MEMBER_ BOUND TO RAMP	VPR	0.48
1Z1L	THE CRYSTAL STRUCTURE OF THE PHOSPHODIESTERASE 2A CATALYTIC DOMAIN	FSR	0.48
2A4D	STRUCTURE OF THE HUMAN UBIQUITIN-CONJUGATING ENZYME E2 VARIANT 1 (UEV-1)	GPK	0.48
3CHO	CRYSTAL STRUCTURE OF LEUKOTRIENE A4 HYDROLASE IN COMPLEX WITH 2-AMINO-N-[4-(PHENYLMETHOXY)PHENYL]-ACETAMIDE	GPR	0.48
2GFO	STRUCTURE OF THE CATALYTIC DOMAIN OF HUMAN UBIQUITIN CARBOXYL-TERMINAL HYDROLASE 8	GPK	0.49
1NKR	INHIBITORY RECEPTOR (P58-CL42) FOR HUMAN NATURAL KILLER CELLS	GPK	0.49
3PBH	REFINED CRYSTAL STRUCTURE OF HUMAN PROCATHEPSIN B AT 2.5 ANGSTROM RESOLUTION	GPK	0.50
1CB0	STRUCTURE OF HUMAN 5'-DEOXY-5'-METHYLTHIOADENOSINE PHOSPHORYLASE AT 1.7 A RESOLUTION	GPR	0.50
1S1P	CRYSTAL STRUCTURES OF PROSTAGLANDIN D2 11-KETOREDUCTASE (AKR1C3) IN COMPLEX WITH THE NON-STEROIDAL ANTI-INFLAMMATORY DRUGS FLUFENAMIC ACID AND INDOMETHACIN	VPR	0.51
2QYM	CRYSTAL STRUCTURE OF UNLIGANDED PDE4C2	VPR	0.51
1LF7	CRYSTAL STRUCTURE OF HUMAN COMPLEMENT PROTEIN C8GAMMA AT 1.2 A RESOLUTION	QAR	0.52
2ALR	ALDEHYDE REDUCTASE	QAR	0.53
1Q33	CRYSTAL STRUCTURE OF HUMAN ADP-RIBOSE PYROPHOSPHATASE NUDT9	GPR	0.53
1J72	CRYSTAL STRUCTURE OF MUTANT MACROPHAGE CAPPING PROTEIN (CAP G) WITH ACTIN-SEVERING ACTIVITY IN THE CA2+-FREE FORM	GPK	0.54
1YO8	STRUCUTURE OF THE C-TERMINAL DOMAIN OF HUMAN THROMBOSPONDIN 2	VPR	0.54
1OZ2	CRYSTAL STRUCTURE OF 3-MBT REPEATS OF LETHAL (3) MALIGNANT BRAIN TUMOR (NATIVE-II) AT 1.55 ANGSTROM	GPR	0.54
1FNH	CRYSTAL STRUCTURE OF HEPARIN AND INTEGRIN BINDING SEGMENT OF HUMAN FIBRONECTIN	VPR	0.56
1TDH	CRYSTAL STRUCTURE OF HUMAN ENDONUCLEASE VIII-LIKE 1 (NEIL1)	GPR	0.56
1UZE	COMPLEX OF THE ANTI-HYPERTENSIVE DRUG ENALAPRILAT AND THE HUMAN TESTICULAR ANGIOTENSIN I-CONVERTING ENZYME	VPR	0.57
2AWF	STRUCTURE OF HUMAN UBIQUITIN-CONJUGATING ENZYME E2 G1	VPR	0.57
2OIL	CRYSTAL STRUCTURE OF HUMAN RAB25 IN COMPLEX WITH GDP	QAR	0.58
2GF9	CRYSTAL STRUCTURE OF HUMAN RAB3D IN COMPLEX WITH GDP	VPR	0.58
1IAP	CRYSTAL STRUCTURE OF P115RHOGEF RGRGS DOMAIN	GPK	0.58
2CZK	CRYSTAL STRUCTURE OF HUMAN MYO-INOSITOL MONOPHOSPHATASE 2 (IMPA2) (TRIGONAL FORM)	GPK	0.58
1WMA	CRYSTAL STRUCTURE OF HUMAN CBR1 IN COMPLEX WITH HYDROXY-PP	GPK	0.59
1PME	STRUCTURE OF PENTA MUTANT HUMAN ERK2 MAP KINASE COMPLEXED WITH A SPECIFIC INHIBITOR OF HUMAN P38 MAP KINASE	GPR	0.60
1PI1	CRYSTAL STRUCTURE OF A HUMAN MOB1 PROTEIN; TOWARD UNDERSTANDING MOB-REGULATED CELL CYCLE PATHWAYS.	GPR	0.61
1VZO	THE STRUCTURE OF THE N-TERMINAL KINASE DOMAIN OF MSK1 REVEALS A NOVEL AUTOINHIBITORY CONFORMATION FOR A DUAL KINASE PROTEIN	GPR	0.62
2A5J	CRYSTAL STRUCTURE OF HUMAN RAB2B	VPR	0.66
1W7L	CRYSTAL STRUCTURE OF HUMAN KYNURENINE AMINOTRANSFERASE I	QAR	0.71
3COU	CRYSTAL STRUCTURE OF HUMAN NUDIX MOTIF 16 (NUDT16)	GPR	0.73
2DW5	CRYSTAL STRUCTURE OF HUMAN PEPTIDYLARGININE DEIMINASE 4 IN COMPLEX WITH N-ALPHA-BENZOYL-N5-(2-FLUORO-1-IMINOETHYL)-L-ORNITHINE AMIDE	GPK	0.74

1CZA	MUTANT MONOMER OF RECOMBINANT HUMAN HEXOKINASE TYPE I COMPLEXED WITH GLUCOSE_ GLUCOSE-6-PHOSPHATE_ AND ADP	GPR	0.74
2QT1	HUMAN NICOTINAMIDE RIBOSIDE KINASE 1 IN COMPLEX WITH NICOTINAMIDE RIBOSIDE	VPR	0.75
2PNY	STRUCTURE OF HUMAN ISOPENTENYL-DIPHOSPHATE DELTA-ISOMERASE	VPR	0.76
1M4K	CRYSTAL STRUCTURE OF THE HUMAN NATURAL KILLER CELL ACTIVATOR RECEPTOR KIR2DS2 (CD158J)	GPK	0.79
1YRV	NOVEL UBIQUITIN-CONJUGATING ENZYME	VPR	0.92
2HQ6	STRUCTURE OF THE CYCLOPHILIN_CECYP16-LIKE DOMAIN OF THE SEROLOGICALLY DEFINED COLON CANCER ANTIGEN 10 FROM HOMO SAPIENS	VPR	1.01
1A17	TETRATRICOPEPTIDE REPEATS OF PROTEIN PHOSPHATASE 5	GPK	2.00
1B6A	HUMAN METHIONINE AMINOPEPTIDASE 2 COMPLEXED WITH TNP-470	GPK	2.00
1HU3	MIDDLE DOMAIN OF HUMAN EIF4GII	GPK	2.00
1LBD	LIGAND-BINDING DOMAIN OF THE HUMAN NUCLEAR RECEPTOR RXR-ALPHA	VPR	2.00
1MX3	CRYSTAL STRUCTURE OF CTBP DEHYDROGENASE CORE HOLO FORM	VPR	2.00
1NG2	STRUCTURE OF AUTOINHIBITED P47PHOX	QAR	2.00
1P5J	CRYSTAL STRUCTURE ANALYSIS OF HUMAN SERINE DEHYDRATASE	VPR	2.00
1PME	STRUCTURE OF PENTA MUTANT HUMAN ERK2 MAP KINASE COMPLEXED WITH A SPECIFIC INHIBITOR OF HUMAN P38 MAP KINASE	VPR	2.00
1Q1C	CRYSTAL STRUCTURE OF N(1-260) OF HUMAN FKBP52	VPR	2.00
1TBF	CATALYTIC DOMAIN OF HUMAN PHOSPHODIESTERASE 5A IN COMPLEX WITH SILDENAFIL	VPR	2.00
1UU3	STRUCTURE OF HUMAN PDK1 KINASE DOMAIN IN COMPLEX WITH LY333531	QAR	2.00
1XAP	STRUCTURE OF THE LIGAND BINDING DOMAIN OF THE RETINOIC ACID RECEPTOR BETA	VPR	2.00
1ZD9	STRUCTURE OF HUMAN ADP-RIBOSYLATION FACTOR-LIKE 10B	VPR	2.00
1ZIV	CATALYTIC DOMAIN OF HUMAN CALPAIN-9	VPR	2.00
1ZJH	STRUCTURE OF HUMAN MUSCLE PYRUVATE KINASE (PKM2)	VPR	2.00
2AXN	CRYSTAL STRUCTURE OF THE HUMAN INDUCIBLE FORM 6-PHOSPHOFRUCTO-2-KINASE/FRUCTOSE-2_6-BISPHOSPHATASE	GPK	2.00
2B3H	CRYSTAL STRUCTURE OF HUMAN METHIONINE AMINOPEPTIDASE TYPE I WITH A THIRD COBALT IN THE ACTIVE SITE	VPR	2.00
2B6H	STRUCTURE OF HUMAN ADP-RIBOSYLATION FACTOR 5	VPR	2.00
2B9E	HUMAN NSUN5 PROTEIN	VPR	2.00
2EW1	CRYSTAL STRUCTURE OF RAB30 IN COMPLEX WITH A GTP ANALOGUE	VPR	2.00
2FAU	CRYSTAL STRUCTURE OF HUMAN VPS26	VPR	2.00
2FG5	CRYSTAL STRUCTURE OF HUMAN RAB31 IN COMPLEX WITH A GTP ANALOGUE	VPR	2.00
2FK9	HUMAN PROTEIN KINASE C_ ETA	VPR	2.00
2FOL	CRYSTAL STRUCTURE OF HUMAN RAB1A IN COMPLEX WITH GDP	VPR	2.00
2FYT	HUMAN HMT1 HNRNP METHYLTRANSFERASE-LIKE 3 (S. CEREVISIAE) PROTEIN	VPR	2.00
2GEE	CRYSTAL STRUCTURE OF HUMAN TYPE III FIBRONECTIN EXTRADOMAIN B AND DOMAIN 8	VPR	2.00
2GFO	STRUCTURE OF THE CATALYTIC DOMAIN OF HUMAN UBIQUITIN CARBOXYL-TERMINAL HYDROLASE 8	VPR	2.00
2GRY	CRYSTAL STRUCTURE OF THE HUMAN KIF2 MOTOR DOMAIN IN COMPLEX WITH ADP	VPR	2.00
2GW2	CRYSTAL STRUCTURE OF THE PEPTIDYL-PROLYL ISOMERASE DOMAIN OF HUMAN CYCLOPHILIN G	VPR	2.00
2H17	STRUCTURE OF HUMAN ADP-RIBOSYLATION FACTOR-LIKE 5 (ARL5) (CASP TARGET)	VPR	2.00
2HEH	CRYSTAL STRUCTURE OF THE KIF2C MOTOR DOMAIN (CASP TARGET)	VPR	2.00
2HZ6	THE CRYSTAL STRUCTURE OF HUMAN IRE1-ALPHA LUMINAL DOMAIN	VPR	2.00
2I7A	DOMAIN IV OF HUMAN CALPAIN 13	VPR	2.00

2I7Q	CRYSTAL STRUCTURE OF HUMAN CHOLINE KINASE A	VPR	2.00
2IL1	CRYSTAL STRUCTURE OF A PREDICTED HUMAN GTPASE IN COMPLEX WITH GDP	VPR	2.00
2IUW	CRYSTAL STRUCTURE OF HUMAN ABH3 IN COMPLEX WITH IRON ION AND 2-OXOGLUTARATE	VPR	2.00
2JC9	CRYSTAL STRUCTURE OF HUMAN CYTOSOLIC 5'-NUCLEOTIDASE II IN COMPLEX WITH ADENOSINE	VPR	2.00
2NR8	CRYSTAL STRUCTURE OF THE HUMAN KIF9 MOTOR DOMAIN IN COMPLEX WITH ADP	VPR	2.00
2NSM	CRYSTAL STRUCTURE OF THE HUMAN CARBOXYPEPTIDASE N (KININASE I) CATALYTIC DOMAIN	QAR	2.00
2OIL	CRYSTAL STRUCTURE OF HUMAN RAB25 IN COMPLEX WITH GDP	VPR	2.00
2QLU	CRYSTAL STRUCTURE OF ACTIVIN RECEPTOR TYPE II KINASE DOMAIN FROM HUMAN	VPR	2.00
2R3A	METHYLTRANSFERASE DOMAIN OF HUMAN SUPPRESSOR OF VARIEGATION 3-9 HOMOLOG 2	GPK	2.00
2REP	CRYSTAL STRUCTURE OF THE MOTOR DOMAIN OF HUMAN KINESIN FAMILY MEMBER C1	VPR	2.00
2VKQ	CRYSTAL STRUCTURE OF HUMAN CYTOSOLIC 5'-NUCLEOTIDASE III (CN-III_ NT5C3) IN COMPLEX WITH BERYLLIUM TRIFLUORIDE	VPR	2.00
2VPJ	CRYSTAL STRUCTURE OF THE KELCH DOMAIN OF HUMAN KLHL12	GPR	2.00
2ZMD	CRYSTAL STRUCTURE OF HUMAN MPS1 CATALYTIC DOMAIN T686A MUTANT IN COMPLEX WITH SP600125 INHIBITOR	VPR	2.00
3BCH	CRYSTAL STRUCTURE OF THE HUMAN LAMININ RECEPTOR PRECURSOR	VPR	2.00
3BD9	HUMAN 3-O-SULFOTRANSFERASE ISOFORM 5 WITH BOUND PAP	VPR	2.00
3BQC	HIGH PH-VALUE CRYSTAL STRUCTURE OF EMODIN IN COMPLEX WITH THE CATALYTIC SUBUNIT OF PROTEIN KINASE CK2	QAR	2.00
	**kallikrein-related peptidase 7**		
3PBH	REFINED CRYSTAL STRUCTURE OF HUMAN PROCATHEPSIN B AT 2.5 ANGSTROM RESOLUTION	RPY	0.16
1T32	A DUAL INHIBITOR OF THE LEUKOCYTE PROTEASES CATHEPSIN G AND CHYMASE WITH THERAPEUTIC EFFICACY IN ANIMALS MODELS OF INFLAMMATION	RPY	0.19
1BIO	HUMAN COMPLEMENT FACTOR D IN COMPLEX WITH ISATOIC ANHYDRIDE INHIBITOR	RPY	0.20
2FOZ	HUMAN ADP-RIBOSYLHYDROLASE 3	RPY	0.21
1NN6	HUMAN PRO-CHYMASE	RPY	0.21
1XK5	CRYSTAL STRUCTURE OF THE M3G-CAP-BINDING DOMAIN OF SNURPORTIN1 IN COMPLEX WITH A M3GPPPG-CAP DINUCLEOTIDE	RPY	0.24
2QOL	HUMAN EPHA3 KINASE AND JUXTAMEMBRANE REGION_ Y596:Y602:S768G TRIPLE MUTANT	RPY	0.26
2D7I	CRSYTAL STRUCTURE OF PP-GALNAC-T10 WITH UDP_ GALNAC AND MN2	RPY	0.27
2J5W	CERULOPLASMIN REVISITED: STRUCTURAL AND FUNCTIONAL ROLES OF VARIOUS METAL CATION BINDING SITES	RPY	0.27
2J5W	CERULOPLASMIN REVISITED: STRUCTURAL AND FUNCTIONAL ROLES OF VARIOUS METAL CATION BINDING SITES	RPY	0.29
2QTZ	CRYSTAL STRUCTURE OF THE NADP+-BOUND FAD-CONTAINING FNR- LIKE MODULE OF HUMAN METHIONINE SYNTHASE REDUCTASE	RPY	0.30
1XQJ	3.10 A CRYSTAL STRUCTURE OF MASPIN_ SPACE GROUP I 4 2 2	RPY	0.30
2REI	KINASE DOMAIN OF HUMAN EPHRIN TYPE-A RECEPTOR 7 (EPHA7)	RPY	0.30
2R2P	KINASE DOMAIN OF HUMAN EPHRIN TYPE-A RECEPTOR 5 (EPHA5)	RPY	0.31
1UMK	THE STRUCTURE OF HUMAN ERYTHROCYTE NADH-CYTOCHROME B5 REDUCTASE	RPY	0.31
2J51	CRYSTAL STRUCTURE OF HUMAN STE20-LIKE KINASE BOUND TO 5- AMINO-3-((4-(AMINOSULFONYL)PHENYL)AMINO)-N-(2_6- DIFLUOROPHENYL)-1H-1_2_4-TRIAZOLE-1-CARBOTHIOAMIDE	RPY	0.37

2UWN	CRYSTAL STRUCTURE OF HUMAN COMPLEMENT FACTOR H_ SCR DOMAINS 6-8 (H402 RISK VARIANT)_ IN COMPLEX WITH LIGAND.	RPY	0.51
3B6E	CRYSTAL STRUCTURE OF HUMAN DECH-BOX RNA HELICASE MDA5 (MELANOMA DIFFERENTIATION-ASSOCIATED PROTEIN 5)_ DECH- DOMAIN	RPY	0.58
2NSQ	CRYSTAL STRUCTURE OF THE C2 DOMAIN OF THE HUMAN E3 UBIQUITIN PROTEIN LIGASE NEDD4-LIKE PROTEIN	RPY	2.00
	**marapsin**		
1UWY	CRYSTAL STRUCTURE OF HUMAN CARBOXYPEPTIDASE M	VGR	0.10
2NZ6	CRYSTAL STRUCTURE OF THE PTPRJ INACTIVATING MUTANT C1239S	VGR	0.11
2I7V	STRUCTURE OF HUMAN CPSF-73	VGR	0.12
1R1H	STRUCTURAL ANALYSIS OF NEPRILYSIN WITH VARIOUS SPECIFIC AND POTENT INHIBITORS	VGR	0.14
1ZVD	REGULATION OF SMURF2 UBIQUITIN LIGASE ACTIVITY BY ANCHORING THE E2 TO THE HECT DOMAIN	VGR	0.14
2IMS	THE X-RAY STRUCTURE OF A BAK HOMODIMER REVEALS AN INHIBITORY ZINC BINDING SITE	VGR	0.15
2BZL	CRYSTAL STRUCTURE OF THE HUMAN PROTEIN TYROSINE PHOSPHATASE N14 AT 1.65 A RESOLUTION	VGR	0.15
1JDW	CRYSTAL STRUCTURE AND MECHANISM OF L-ARGININE: GLYCINE AMIDINOTRANSFERASE: A MITOCHONDRIAL ENZYME INVOLVED IN CREATINE BIOSYNTHESIS	VGR	0.15
2PBN	CRYSTAL STRUCTURE OF THE HUMAN TYROSINE RECEPTOR PHOSPHATE GAMMA	VGR	0.15
1Q33	CRYSTAL STRUCTURE OF HUMAN ADP-RIBOSE PYROPHOSPHATASE NUDT9	VGR	0.15
2FH7	CRYSTAL STRUCTURE OF THE PHOSPHATASE DOMAINS OF HUMAN PTP SIGMA	VGR	0.15
2FH7	CRYSTAL STRUCTURE OF THE PHOSPHATASE DOMAINS OF HUMAN PTP SIGMA	VGR	0.16
1X8B	STRUCTURE OF HUMAN WEE1A KINASE: KINASE DOMAIN COMPLEXED WITH INHIBITOR PD0407824	VGR	0.16
2NZ2	CRYSTAL STRUCTURE OF HUMAN ARGININOSUCCINATE SYNTHASE IN COMPLEX WITH ASPARTATE AND CITRULLINE	VGR	0.16
2FY2	STRUCTURES OF LIGAND BOUND HUMAN CHOLINE ACETYLTRANSFERASE PROVIDE INSIGHT INTO REGULATION OF ACETYLCHOLINE SYNTHESIS	VGR	0.16
1UWY	CRYSTAL STRUCTURE OF HUMAN CARBOXYPEPTIDASE M	VGR	0.16
1NUF	ROLE OF CALCIUM IONS IN THE ACTIVATION AND ACTIVITY OF THE TRANSGLUTAMINASE 3 ENZYME	VGR	0.16
2NSM	CRYSTAL STRUCTURE OF THE HUMAN CARBOXYPEPTIDASE N (KININASE I) CATALYTIC DOMAIN	VGR	0.16
2HC1	ENGINEERED CATALYTIC DOMAIN OF PROTEIN TYROSINE PHOSPHATASE HPTPBETA.	VGR	0.17
1FA9	HUMAN LIVER GLYCOGEN PHOSPHORYLASE A COMPLEXED WITH AMP	VGR	0.17
2NZL	CRYSTAL STRUCTURE OF HUMAN HYDROXYACID OXIDASE 1	VGR	0.19
2A2K	CRYSTAL STRUCTURE OF AN ACTIVE SITE MUTANT_ C473S_ OF CDC25B PHOSPHATASE CATALYTIC DOMAIN	GPR	0.19
1F5N	HUMAN GUANYLATE BINDING PROTEIN-1 IN COMPLEX WITH THE GTP ANALOGUE_ GMPPNP.	GPR	0.19
1XKI	CRYSTAL STRUCTURE OF HUMAN TEAR LIPOCALIN/VON EBNERS GLAND PROTEIN	VGR	0.19
1Z8D	CRYSTAL STRUCTURE OF HUMAN MUSCLE GLYCOGEN PHOSPHORYLASE A WITH AMP AND GLUCOSE	VGR	0.21
1C25	HUMAN CDC25A CATALYTIC DOMAIN	GPR	0.21
1EVS	CRYSTAL STRUCTURE OF HUMAN ONCOSTATIN M	VGR	0.21
2RIP	STRUCTURE OF DPPIV IN COMPLEX WITH AN INHIBITOR	VGR	0.23
2QCF	CRYSTAL STRUCTURE OF THE OROTIDINE-5'-MONOPHOSPHATE DECARBOXYLASE DOMAIN (ASP312ASN MUTANT) OF HUMAN UMP SYNTHASE BOUND TO 5-FLUORO-UMP	VGR	0.23

1KGD	CRYSTAL STRUCTURE OF THE GUANYLATE KINASE-LIKE DOMAIN OF HUMAN CASK	VGR	0.24
2BX6	CRYSTAL STRUCTURE OF THE HUMAN RETINITIS PIGMENTOSA PROTEIN 2 (RP2)	VGR	0.26
1IAT	CRYSTAL STRUCTURE OF HUMAN PHOSPHOGLUCOSE ISOMERASE/NEUROLEUKIN/AUTOCRINE MOTILITY FACTOR/MATURATION FACTOR	GPR	0.27
2B5M	CRYSTAL STRUCTURE OF DDB1	VGR	0.27
1S9J	X-RAY STRUCTURE OF THE HUMAN MITOGEN-ACTIVATED PROTEIN KINASE KINASE 1 (MEK1) IN A COMPLEX WITH LIGAND AND MGATP	VGR	0.28
1KT0	STRUCTURE OF THE LARGE FKBP-LIKE PROTEIN_ FKBP51_ INVOLVED IN STEROID RECEPTOR COMPLEXES	GPR	0.28
2B69	CRYSTAL STRUCTURE OF HUMAN UDP-GLUCORONIC ACID DECARBOXYLAS	GPR	0.28
1QOI	U4/U6 SNRNP-SPECIFIC CYCLOPHILIN SNUCYP-20	VGR	0.29
2JHM	STRUCTURE OF GLOBULAR HEADS OF M-FICOLIN AT NEUTRAL PH	GPR	0.29
2Z6O	CRYSTAL STRUCTURE OF THE UFC1_ UFM1 CONJUGATING ENZYME 1	GPR	0.30
1KO9	NATIVE STRUCTURE OF THE HUMAN 8-OXOGUANINE DNA GLYCOSYLASE HOGG1	GPR	0.30
1IAP	CRYSTAL STRUCTURE OF P115RHOGEF RGRGS DOMAIN	VGR	0.31
2AC3	STRUCTURE OF HUMAN MNK2 KINASE DOMAIN	VGR	0.32
2HZ6	THE CRYSTAL STRUCTURE OF HUMAN IRE1-ALPHA LUMINAL DOMAIN	VGR	0.32
2HC1	ENGINEERED CATALYTIC DOMAIN OF PROTEIN TYROSINE PHOSPHATASE HPTPBETA.	VGR	0.33
1TQN	CRYSTAL STRUCTURE OF HUMAN MICROSOMAL P450 3A4	GPR	0.34
1ZVD	REGULATION OF SMURF2 UBIQUITIN LIGASE ACTIVITY BY ANCHORING THE E2 TO THE HECT DOMAIN	GPR	0.34
2DW4	CRYSTAL STRUCTURE OF HUMAN LSD1 AT 2.3 A RESOLUTION	VGR	0.34
2HI4	CRYSTAL STRUCTURE OF HUMAN MICROSOMAL P450 1A2 IN COMPLEX WITH ALPHA-NAPHTHOFLAVONE	GPR	0.35
2CGO	FACTOR INHIBITING HIF-1 ALPHA WITH FUMARATE	VGR	0.35
2OEW	STRUCTURE OF ALIX/AIP1 BRO1 DOMAIN	VGR	0.37
1D2S	CRYSTAL STRUCTURE OF THE N-TERMINAL LAMININ G-LIKE DOMAIN OF SHBG IN COMPLEX WITH DIHYDROTESTOSTERONE	GPR	0.38
2AXN	CRYSTAL STRUCTURE OF THE HUMAN INDUCIBLE FORM 6-PHOSPHOFRUCTO-2-KINASE/FRUCTOSE-2_6-BISPHOSPHATASE	VGR	0.38
2V5O	STRUCTURE OF HUMAN IGF2R DOMAINS 11-14	VGR	0.38
2Z5Y	CRYSTAL STRUCTURE OF HUMAN MONOAMINE OXIDASE A (G110A) WITH HARMINE	VGR	0.39
1XWW	CRYSTAL STRUCTURE OF HUMAN B-FORM LOW MOLECULAR WEIGHT PHOSPHOTYROSYL PHOSPHATASE AT 1.6 ANGSTROM RESOLUTION	VGR	0.40
1H30	C-TERMINAL LG DOMAIN PAIR OF HUMAN GAS6	VGR	0.40
2B69	CRYSTAL STRUCTURE OF HUMAN UDP-GLUCORONIC ACID DECARBOXYLAS	GPR	0.40
1N26	CRYSTAL STRUCTURE OF THE EXTRA-CELLULAR DOMAINS OF HUMAN INTERLEUKIN-6 RECEPTOR ALPHA CHAIN	GPR	0.41
1HH8	THE ACTIVE N-TERMINAL REGION OF P67PHOX: STRUCTURE AT 1.8 ANGSTROM RESOLUTION AND BIOCHEMICAL CHARACTERIZATIONS OF THE A128V MUTANT IMPLICATED IN CHRONIC GRANULOMATOUS DISEASE	VGR	0.42
1S31	CRYSTAL STRUCTURE ANALYSIS OF THE HUMAN TUB PROTEIN (ISOFORM A) SPANNING RESIDUES 289 THROUGH 561	GPR	0.44
1HDO	HUMAN BILIVERDIN IX BETA REDUCTASE: NADP COMPLEX	GPR	0.45
1CJM	HUMAN SULT1A3 WITH SULFATE BOUND	VGR	0.46
2EVA	STRUCTURAL BASIS FOR THE INTERACTION OF TAK1 KINASE WITH ITS ACTIVATING PROTEIN TAB1	VGR	0.47
2I7A	DOMAIN IV OF HUMAN CALPAIN 13	VGR	0.47
1NTY	CRYSTAL STRUCTURE OF THE FIRST DH/PH DOMAIN OF TRIO TO 1.7	VGR	0.47
3CHO	CRYSTAL STRUCTURE OF LEUKOTRIENE A4 HYDROLASE IN COMPLEX WITH 2-AMINO-N-[4-(PHENYLMETHOXY)PHENYL]-ACETAMIDE	GPR	0.48

1ZRH	CRYSTAL STRUCTURE OF HUMAN HEPARAN SULFATE GLUCOSAMINE 3-O-SULFOTRANSFERASE 1 IN COMPLEX WITH PAP	VGR	0.49
1CB0	STRUCTURE OF HUMAN 5'-DEOXY-5'-METHYLTHIOADENOSINE PHOSPHORYLASE AT 1.7 A RESOLUTION	GPR	0.50
1Q33	CRYSTAL STRUCTURE OF HUMAN ADP-RIBOSE PYROPHOSPHATASE NUDT9	GPR	0.53
1OZ2	CRYSTAL STRUCTURE OF 3-MBT REPEATS OF LETHAL (3) MALIGNANT BRAIN TUMOR (NATIVE-II) AT 1.55 ANGSTROM	GPR	0.54
1TDH	CRYSTAL STRUCTURE OF HUMAN ENDONUCLEASE VIII-LIKE 1 (NEIL1)	GPR	0.56
1IAP	CRYSTAL STRUCTURE OF P115RHOGEF RGRGS DOMAIN	VGR	0.58
2CMW	STRUCTURE OF HUMAN CASEIN KINASE 1 GAMMA-1 IN COMPLEX WITH 2-(2-HYDROXYETHYLAMINO)-6-(3-CHLOROANILINO)-9- ISOPROPYLPURINE (CASP TARGET)	VGR	0.58
1PME	STRUCTURE OF PENTA MUTANT HUMAN ERK2 MAP KINASE COMPLEXED WITH A SPECIFIC INHIBITOR OF HUMAN P38 MAP KINASE	GPR	0.60
1PI1	CRYSTAL STRUCTURE OF A HUMAN MOB1 PROTEIN; TOWARD UNDERSTANDING MOB-REGULATED CELL CYCLE PATHWAYS.	GPR	0.61
1VZO	THE STRUCTURE OF THE N-TERMINAL KINASE DOMAIN OF MSK1 REVEALS A NOVEL AUTOINHIBITORY CONFORMATION FOR A DUAL KINASE PROTEIN	GPR	0.62
1ZGK	1.35 ANGSTROM STRUCTURE OF THE KELCH DOMAIN OF KEAP1	VGR	0.66
1SZ7	CRYSTAL STRUCTURE OF HUMAN BET3	VGR	0.70
3COU	CRYSTAL STRUCTURE OF HUMAN NUDIX MOTIF 16 (NUDT16)	GPR	0.73
2C7S	CRYSTAL STRUCTURE OF HUMAN PROTEIN TYROSINE PHOSPHATASE KAPPA AT 1.95A RESOLUTION	VGR	0.74
1CZA	MUTANT MONOMER OF RECOMBINANT HUMAN HEXOKINASE TYPE I COMPLEXED WITH GLUCOSE_ GLUCOSE-6-PHOSPHATE_ AND ADP	GPR	0.74
1NF1	THE GAP RELATED DOMAIN OF NEUROFIBROMIN	VGR	2.00
2I4I	CRYSTAL STRUCTURE OF HUMAN DEAD-BOX RNA HELICASE DDX3X	VGR	2.00
2VPJ	CRYSTAL STRUCTURE OF THE KELCH DOMAIN OF HUMAN KLHL12	GPR	2.00
	**matriptase**		
2NSM	CRYSTAL STRUCTURE OF THE HUMAN CARBOXYPEPTIDASE N (KININASE I) CATALYTIC DOMAIN	LGR	0.10
1W6K	STRUCTURE OF HUMAN OSC IN COMPLEX WITH LANOSTEROL	LGR	0.10
2O8T	CRYSTAL STRUCTURE AND BINDING EPITOPES OF UROKINASE-TYPE PLASMINOGEN ACTIVATOR (C122A/N145Q) IN COMPLEX WITH INHIBITORS	LGR	0.11
1SPJ	STRUCTURE OF MATURE HUMAN TISSUE KALLIKREIN (HUMAN KALLIKREIN 1 OR KLK1) AT 1.70 ANGSTROM RESOLUTION WITH VACANT ACTIVE SITE	LGR	0.12
2DW5	CRYSTAL STRUCTURE OF HUMAN PEPTIDYLARGININE DEIMINASE 4 IN COMPLEX WITH N-ALPHA-BENZOYL-N5-(2-FLUORO-1-IMINOETHYL)-L-ORNITHINE AMIDE	LGR	0.12
1OHC	STRUCTURE OF THE PROLINE DIRECTED PHOSPHATASE CDC14	LGR	0.13
2PCX	CRYSTAL STRUCTURE OF P53DBD(R282Q) AT 1.54-ANGSTROM RESOLUTION	LGR	0.15
1T32	A DUAL INHIBITOR OF THE LEUKOCYTE PROTEASES CATHEPSIN G AND CHYMASE WITH THERAPEUTIC EFFICACY IN ANIMALS MODELS OF INFLAMMATION	AFK	0.15
1LCT	STRUCTURE OF THE RECOMBINANT N-TERMINAL LOBE OF HUMAN LACTOFERRIN AT 2.0 ANGSTROMS RESOLUTION	AFK	0.16
1Q33	CRYSTAL STRUCTURE OF HUMAN ADP-RIBOSE PYROPHOSPHATASE NUDT9	LGR	0.16
1KHB	PEPCK COMPLEX WITH NONHYDROLYZABLE GTP ANALOG_ NATIVE DATA	LGR	0.16
1RYO	HUMAN SERUM TRANSFERRIN_ N-LOBE BOUND WITH OXALATE	AFK	0.16
1UOU	CRYSTAL STRUCTURE OF HUMAN THYMIDINE PHOSPHORYLASE IN COMPLEX WITH A SMALL MOLECULE INHIBITOR	LGR	0.16
1Z8D	CRYSTAL STRUCTURE OF HUMAN MUSCLE GLYCOGEN PHOSPHORYLASE A WITH AMP AND GLUCOSE	LGR	0.17

1OI1	CRYSTAL STRUCTURE OF THE MBT DOMAINS OF HUMAN SCML2	EAR	0.18
1YCK	CRYSTAL STRUCTURE OF HUMAN PEPTIDOGLYCAN RECOGNITION PROTEIN (PGRP-S)	EGR	0.18
1NM8	STRUCTURE OF HUMAN CARNITINE ACETYLTRANSFERASE: MOLECULAR BASIS FOR FATTY ACYL TRANSFER	LGR	0.18
2B5M	CRYSTAL STRUCTURE OF DDB1	AFK	0.18
1M6I	CRYSTAL STRUCTURE OF APOPTOSIS INDUCING FACTOR (AIF)	LGR	0.19
2Q5I	CRYSTAL STRUCTURE OF APO S581L GLYCYL-TRNA SYNTHETASE MUTAN	LGR	0.19
2CY7	THE CRYSTAL STRUCTURE OF HUMAN ATG4B	LGR	0.19
1UOU	CRYSTAL STRUCTURE OF HUMAN THYMIDINE PHOSPHORYLASE IN COMPLEX WITH A SMALL MOLECULE INHIBITOR	LGR	0.19
3CHO	CRYSTAL STRUCTURE OF LEUKOTRIENE A4 HYDROLASE IN COMPLEX WITH 2-AMINO-N-[4-(PHENYLMETHOXY)PHENYL]-ACETAMIDE	QGR	0.19
1OZN	1.5A CRYSTAL STRUCTURE OF THE NOGO RECEPTOR LIGAND BINDING DOMAIN REVEALS A CONVERGENT RECOGNITION SCAFFOLD MEDIATING INHIBITION OF MYELINATION	LGR	0.19
1FA9	HUMAN LIVER GLYCOGEN PHOSPHORYLASE A COMPLEXED WITH AMP	LGR	0.19
2B5M	CRYSTAL STRUCTURE OF DDB1	QGR	0.19
1LF7	CRYSTAL STRUCTURE OF HUMAN COMPLEMENT PROTEIN C8GAMMA AT 1.2 A RESOLUTION	LGR	0.20
2O3H	CRYSTAL STRUCTURE OF THE HUMAN C65A APE	EGR	0.20
1WMA	CRYSTAL STRUCTURE OF HUMAN CBR1 IN COMPLEX WITH HYDROXY-PP	QGR	0.20
1RYO	HUMAN SERUM TRANSFERRIN_ N-LOBE BOUND WITH OXALATE	LGR	0.20
2F9L	3D STRUCTURE OF INACTIVE HUMAN RAB11B GTPASE	AFK	0.20
1SIQ	THE CRYSTAL STRUCTURE AND MECHANISM OF HUMAN GLUTARYL-COA DEHYDROGENASE	QAR	0.20
2RKU	STRUCTURE OF PLK1 IN COMPLEX WITH BI2536	EAR	0.20
2PE4	STRUCTURE OF HUMAN HYALURONIDASE 1_ A HYALURONAN HYDROLYZING ENZYME INVOLVED IN TUMOR GROWTH AND ANGIOGENESIS	LGR	0.21
1HVF	STRUCTURAL AND ELECTROPHYSIOLOGICAL ANALYSIS OF ANNEXIN V MUTANTS. MUTAGENESIS OF HUMAN ANNEXIN V_ AN IN VITRO VOLTAGE-GATED CALCIUM CHANNEL_ PROVIDES INFORMATION ABOUT THE STRUCTURAL FEATURES OF THE ION PATHWAY_ THE VOLTAGE SENSOR AND THE ION SELECTIVITY FILTER	AFK	0.21
1T7V	ZN-ALPHA-2-GLYCOPROTEIN; BACULO-ZAG PEG 200	QGR	0.22
2I7V	STRUCTURE OF HUMAN CPSF-73	LGR	0.22
2FY2	STRUCTURES OF LIGAND BOUND HUMAN CHOLINE ACETYLTRANSFERASE PROVIDE INSIGHT INTO REGULATION OF ACETYLCHOLINE SYNTHESIS	EGR	0.22
1OZN	1.5A CRYSTAL STRUCTURE OF THE NOGO RECEPTOR LIGAND BINDING DOMAIN REVEALS A CONVERGENT RECOGNITION SCAFFOLD MEDIATING INHIBITION OF MYELINATION	LGR	0.22
2V24	STRUCTURE OF THE HUMAN SPRY DOMAIN-CONTAINING SOCS BOX PROTEIN SSB-4	LGR	0.22
2I7Q	CRYSTAL STRUCTURE OF HUMAN CHOLINE KINASE A	QAR	0.22
1KL9	CRYSTAL STRUCTURE OF THE N-TERMINAL SEGMENT OF HUMAN EUKARYOTIC INITIATION FACTOR 2ALPHA	AFK	0.22
2DW4	CRYSTAL STRUCTURE OF HUMAN LSD1 AT 2.3 A RESOLUTION	EAR	0.22
2G6B	CRYSTAL STRUCTURE OF HUMAN RAB26 IN COMPLEX WITH A GTP ANALOGUE	AFK	0.23
1LI4	HUMAN S-ADENOSYLHOMOCYSTEINE HYDROLASE COMPLEXED WITH NEPLANOCIN	EGR	0.23
2OEW	STRUCTURE OF ALIX/AIP1 BRO1 DOMAIN	AFK	0.23
1SIQ	THE CRYSTAL STRUCTURE AND MECHANISM OF HUMAN GLUTARYL-COA DEHYDROGENASE	LGR	0.23
2HGS	HUMAN GLUTATHIONE SYNTHETASE	EAR	0.23
1YCK	CRYSTAL STRUCTURE OF HUMAN PEPTIDOGLYCAN RECOGNITION PROTEIN (PGRP-S)	QAR	0.23

1MF7	INTEGRIN ALPHA M I DOMAIN	AFK	0.23
2HQ6	STRUCTURE OF THE CYCLOPHILIN_CECYP16-LIKE DOMAIN OF THE SEROLOGICALLY DEFINED COLON CANCER ANTIGEN 10 FROM HOMO SAPIENS	LGR	0.23
2I53	CRYSTAL STRUCTURE OF CYCLIN K	EAR	0.23
1N5U	X-RAY STUDY OF HUMAN SERUM ALBUMIN COMPLEXED WITH HEME	AFK	0.24
2Z5Y	CRYSTAL STRUCTURE OF HUMAN MONOAMINE OXIDASE A (G110A) WITH HARMINE	EAR	0.24
2HZ6	THE CRYSTAL STRUCTURE OF HUMAN IRE1-ALPHA LUMINAL DOMAIN	LGR	0.24
1XWI	CRYSTAL STRUCTURE OF VPS4B	LGR	0.24
1SZ7	CRYSTAL STRUCTURE OF HUMAN BET3	AFK	0.24
1SO7	MALTOSE-INDUCED STRUCTURE OF THE HUMAN CYTOLSOLIC SIALIDASE NEU2	QAR	0.24
2HQQ	CRYSTAL STRUCTURE OF HUMAN KETOHEXOKINASE COMPLEXED TO DIFFERENT SUGAR MOLECULES	EGR	0.24
2DH2	CRYSTAL STRUCTURE OF HUMAN ED-4F2HC	QAR	0.25
2GY5	TIE2 LIGAND-BINDING DOMAIN CRYSTAL STRUCTURE	EGR	0.25
1KT0	STRUCTURE OF THE LARGE FKBP-LIKE PROTEIN_ FKBP51_ INVOLVED IN STEROID RECEPTOR COMPLEXES	EGR	0.25
1GS9	APOLIPOPROTEIN E4_ 22K DOMAIN	LGR	0.25
2UW2	CRYSTAL STRUCTURE OF HUMAN RIBONUCLEOTIDE REDUCTASE SUBUNIT R2	EAR	0.25
1R9O	CRYSTAL STRUCTURE OF P4502C9 WITH FLURBIPROFEN BOUND	EAR	0.25
1ZIV	CATALYTIC DOMAIN OF HUMAN CALPAIN-9	AFK	0.26
2CL3	CRYSTAL STRUCTURE OF HUMAN CLEAVAGE AND POLYADENYLATION SPECIFICITY FACTOR 5 (CPSF5)	LGR	0.26
2D7I	CRSYTAL STRUCTURE OF PP-GALNAC-T10 WITH UDP_ GALNAC AND MN2	QGR	0.26
2ILR	CRYSTAL STRUCTURE OF HUMAN FANCONI ANEMIA PROTEIN E C-TERMINAL DOMAIN	LGR	0.26
2HI4	CRYSTAL STRUCTURE OF HUMAN MICROSOMAL P450 1A2 IN COMPLEX WITH ALPHA-NAPHTHOFLAVONE	QAR	0.26
1LCY	CRYSTAL STRUCTURE OF THE MITOCHONDRIAL SERINE PROTEASE HTRA	LGR	0.27
3BGS	STRUCTURE OF HUMAN PURINE NUCLEOSIDE PHOSPHORYLASE WITH L-DADME-IMMH AND PHOSPHATE	QGR	0.27
2O36	CRYSTAL STRUCTURE OF ENGINEERED THIMET OLIGOPEPTIDASE WITH NEUROLYSIN SPECIFICITY IN NEUROTENSIN CLEAVAGE SITE	LGR	0.27
1ALU	HUMAN INTERLEUKIN-6	QAR	0.27
2B5M	CRYSTAL STRUCTURE OF DDB1	EGR	0.27
1W8M	ENZYMATIC AND STRUCTURAL CHARACTERISATION OF NON PEPTIDE LIGAND CYCLOPHILIN COMPLEXES	LGR	0.27
2B1P	INHIBITOR COMPLEX OF JNK3	QAR	0.27
1SQW	CRYSTAL STRUCTURE OF KD93_ A NOVEL PROTEIN EXPRESSED IN THE HUMAN PRO	LGR	0.27
1K95	CRYSTAL STRUCTURE OF DES(1-52)GRANCALCIN WITH BOUND CALCIUM	AFK	0.27
1MF7	INTEGRIN ALPHA M I DOMAIN	LGR	0.28
1SIQ	THE CRYSTAL STRUCTURE AND MECHANISM OF HUMAN GLUTARYL-COA DEHYDROGENASE	LGR	0.28
3BKB	CRYSTAL STRUCTURE OF HUMAN FELINE SARCOMA VIRAL ONCOGENE HOMOLOGUE (V-FES)	EAR	0.28
2BIT	CRYSTAL STRUCTURE OF HUMAN CYCLOPHILIN D AT 1.7 A RESOLUTIO	LGR	0.28
1M6I	CRYSTAL STRUCTURE OF APOPTOSIS INDUCING FACTOR (AIF)	QAR	0.28
1ORE	HUMAN ADENINE PHOSPHORIBOSYLTRANSFERASE	LGR	0.28
1B0F	CRYSTAL STRUCTURE OF HUMAN NEUTROPHIL ELASTASE WITH MDL 101 146	LGR	0.28
2OPW	CRYSTAL STRUCTURE OF HUMAN PHYTANOYL-COA DIOXYGENASE PHYHD1 (APO)	LGR	0.28
1TA0	THREE-DIMENSIONAL STRUCTURE OF A RNA-POLYMERASE II BINDING PROTEIN WITH ASSOCIATED LIGAND.	LGR	0.28

2V7O	CRYSTAL STRUCTURE OF HUMAN CALCIUM-CALMODULIN-DEPENDENT PROTEIN KINASE II GAMMA	EAR	0.29
2H6D	PROTEIN KINASE DOMAIN OF THE HUMAN 5'-AMP-ACTIVATED PROTEIN KINASE CATALYTIC SUBUNIT ALPHA-2 (AMPK ALPHA-2 CHAIN)	EAR	0.29
1ZD3	HUMAN SOLUBLE EPOXIDE HYDROLASE 4-(3-CYCLOHEXYLURIEDO)-BUTYRIC ACID COMPLEX	LGR	0.29
1OZ2	CRYSTAL STRUCTURE OF 3-MBT REPEATS OF LETHAL (3) MALIGNANT BRAIN TUMOR (NATIVE-II) AT 1.55 ANGSTROM	AFK	0.29
2I4I	CRYSTAL STRUCTURE OF HUMAN DEAD-BOX RNA HELICASE DDX3X	EAR	0.29
2V5O	STRUCTURE OF HUMAN IGF2R DOMAINS 11-14	AFK	0.29
1H30	C-TERMINAL LG DOMAIN PAIR OF HUMAN GAS6	LGR	0.29
1WOJ	CRYSTAL STRUCTURE OF HUMAN PHOSPHODIESTERASE	AFK	0.30
1UOU	CRYSTAL STRUCTURE OF HUMAN THYMIDINE PHOSPHORYLASE IN COMPLEX WITH A SMALL MOLECULE INHIBITOR	QAR	0.30
2GJK	STRUCTURAL AND FUNCTIONAL INSIGHTS INTO THE HUMAN UPF1 HELICASE CORE	QGR	0.30
1NN6	HUMAN PRO-CHYMASE	AFK	0.30
2FY2	STRUCTURES OF LIGAND BOUND HUMAN CHOLINE ACETYLTRANSFERASE PROVIDE INSIGHT INTO REGULATION OF ACETYLCHOLINE SYNTHESIS	EAR	0.30
2V9R	FIRST AND SECOND IG DOMAINS FROM HUMAN ROBO1 (FORM 2)	EGR	0.30
1S35	CRYSTAL STRUCTURE OF REPEATS 8 AND 9 OF HUMAN ERYTHROID SPECTRIN	LGR	0.31
2B3X	STRUCTURE OF AN ORTHORHOMBIC CRYSTAL FORM OF HUMAN CYTOSOLIC ACONITASE (IRP1)	EGR	0.31
1MX3	CRYSTAL STRUCTURE OF CTBP DEHYDROGENASE CORE HOLO FORM	LGR	0.31
1P0I	CRYSTAL STRUCTURE OF HUMAN BUTYRYL CHOLINESTERASE	EAR	0.31
1E0S	SMALL G PROTEIN ARF6-GDP	EAR	0.31
1F6W	STRUCTURE OF THE CATALYTIC DOMAIN OF HUMAN BILE SALT ACTIVATED LIPASE	QGR	0.31
1N83	CRYSTAL STRUCTURE OF THE COMPLEX BETWEEN THE ORPHAN NUCLEAR HORMONE RECEPTOR ROR(ALPHA)-LBD AND CHOLESTEROL	AFK	0.31
1BY7	HUMAN PLASMINOGEN ACTIVATOR INHIBITOR-2. LOOP (66-98) DELETION MUTANT	EAR	0.31
1M9I	CRYSTAL STRUCTURE OF PHOSPHORYLATION-MIMICKINGMUTANT T356D OF ANNEXIN VI	QAR	0.31
2OC3	CRYSTAL STRUCTURE OF THE CATALYTIC DOMAIN OF HUMAN PROTEIN TYROSINE PHOSPHATASE NON-RECEPTOR TYPE 18	EAR	0.31
1X04	CRYSTAL STRUCTURE OF ENDOPHILIN BAR DOMAIN (MUTANT)	EGR	0.32
1GS9	APOLIPOPROTEIN E4_ 22K DOMAIN	QAR	0.32
1M8Z	CRYSTAL STRUCTURE OF A PUMILIO-HOMOLOGY DOMAIN	AFK	0.32
2CY7	THE CRYSTAL STRUCTURE OF HUMAN ATG4B	LGR	0.32
1X03	CRYSTAL STRUCTURE OF ENDOPHILIN BAR DOMAIN	EGR	0.32
2PET	LUTHERAN GLYCOPROTEIN_ N-TERMINAL DOMAINS 1 AND 2.	QGR	0.33
2FAU	CRYSTAL STRUCTURE OF HUMAN VPS26	AFK	0.33
1K1B	CRYSTAL STRUCTURE OF THE ANKYRIN REPEAT DOMAIN OF BCL-3: A UNIQUE MEMBER OF THE IKAPPAB PROTEIN FAMILY	EAR	0.33
1W6K	STRUCTURE OF HUMAN OSC IN COMPLEX WITH LANOSTEROL	LGR	0.33
2QZ4	HUMAN PARAPLEGIN_ AAA DOMAIN IN COMPLEX WITH ADP	EAR	0.33
1X9D	CRYSTAL STRUCTURE OF HUMAN CLASS I ALPHA-1_2-MANNOSIDASE IN COMPLEX WITH THIO-DISACCHARIDE SUBSTRATE ANALOGUE	EAR	0.33
1YPV	STRUCTURE OF HUMAN THYMIDYLATE SYNTHASE AT LOW SALT CONDITIONS	QAR	0.34
1KMQ	CRYSTAL STRUCTURE OF A CONSTITUTIVELY ACTIVATED RHOA MUTANT (Q63L)	EGR	0.34
2GRY	CRYSTAL STRUCTURE OF THE HUMAN KIF2 MOTOR DOMAIN IN COMPLEX WITH ADP	LGR	0.34
1K04	CRYSTAL STRUCTURE OF THE FOCAL ADHESION TARGETING DOMAIN OF FOCAL ADHESION KINASE	QAR	0.34

2OHF	CRYSTAL STRUCTURE OF HUMAN OLA1 IN COMPLEX WITH AMPPCP	QGR	0.34
2ZG1	CRYSTAL STRUCTURE OF TWO N-TERMINAL DOMAINS OF SIGLEC-5 IN COMPLEX WITH 6'-SIALYLLACTOSE	QGR	0.34
1LI4	HUMAN S-ADENOSYLHOMOCYSTEINE HYDROLASE COMPLEXED WITH NEPLANOCIN	LGR	0.34
1UZE	COMPLEX OF THE ANTI-HYPERTENSIVE DRUG ENALAPRILAT AND THE HUMAN TESTICULAR ANGIOTENSIN I-CONVERTING ENZYME	QAR	0.35
1OSH	A CHEMICAL_ GENETIC_ AND STRUCTURAL ANALYSIS OF THE NUCLEAR BILE ACID RECEPTOR FXR	LGR	0.35
1IAT	CRYSTAL STRUCTURE OF HUMAN PHOSPHOGLUCOSE ISOMERASE/NEUROLEUKIN/AUTOCRINE MOTILITY FACTOR/MATURATION FACTOR	EAR	0.35
1X8B	STRUCTURE OF HUMAN WEE1A KINASE: KINASE DOMAIN COMPLEXED WITH INHIBITOR PD0407824	QGR	0.35
2I7Q	CRYSTAL STRUCTURE OF HUMAN CHOLINE KINASE A	QGR	0.35
1E2S	CRYSTAL STRUCTURE OF AN ARYLSULFATASE A MUTANT C69A	EAR	0.35
1IAP	CRYSTAL STRUCTURE OF P115RHOGEF RGRGS DOMAIN	EAR	0.36
2I7V	STRUCTURE OF HUMAN CPSF-73	EAR	0.36
2ESB	CRYSTAL STRUCTURE OF HUMAN DUSP18	QGR	0.36
2OYC	CRYSTAL STRUCTURE OF HUMAN PYRIDOXAL PHOSPHATE PHOSPHATASE	LGR	0.36
1ZC0	CRYSTAL STRUCTURE OF HUMAN HEMATOPOIETIC TYROSINE PHOSPHATASE (HEPTP) CATALYTIC DOMAIN	LGR	0.36
2A91	CRYSTAL STRUCTURE OF ERBB2 DOMAINS 1-3	EGR	0.36
3CBQ	CRYSTAL STRUCTURE OF THE HUMAN REM2 GTPASE WITH BOUND GDP	EGR	0.37
1CJM	HUMAN SULT1A3 WITH SULFATE BOUND	QAR	0.37
1DG6	CRYSTAL STRUCTURE OF APO2L/TRAIL	LGR	0.37
2F9L	3D STRUCTURE OF INACTIVE HUMAN RAB11B GTPASE	EAR	0.37
2NZ2	CRYSTAL STRUCTURE OF HUMAN ARGININOSUCCINATE SYNTHASE IN COMPLEX WITH ASPARTATE AND CITRULLINE	EAR	0.37
1KAO	CRYSTAL STRUCTURE OF THE SMALL G PROTEIN RAP2A WITH GDP	EGR	0.37
1WB0	SPECIFICITY AND AFFINITY OF NATURAL PRODUCT CYCLOPENTAPEPTIDE INHIBITOR ARGIFIN AGAINST HUMAN CHITINAS	EAR	0.37
2NZ2	CRYSTAL STRUCTURE OF HUMAN ARGININOSUCCINATE SYNTHASE IN COMPLEX WITH ASPARTATE AND CITRULLINE	LGR	0.37
2OZU	CRYSTAL STRUCTURE OF HUMAN MYST HISTONE ACETYLTRANSFERASE 3 IN COMPLEX WITH ACETYLCOENZYME A	LGR	0.37
2IUW	CRYSTAL STRUCTURE OF HUMAN ABH3 IN COMPLEX WITH IRON ION AND 2-OXOGLUTARATE	LGR	0.37
2OIL	CRYSTAL STRUCTURE OF HUMAN RAB25 IN COMPLEX WITH GDP	EAR	0.38
1NUF	ROLE OF CALCIUM IONS IN THE ACTIVATION AND ACTIVITY OF THE TRANSGLUTAMINASE 3 ENZYME	EAR	0.38
2G3Y	CRYSTAL STRUCTURE OF THE HUMAN SMALL GTPASE GEM	EGR	0.38
2EC8	CRYSTAL STRUCTURE OF THE EXCTRACELLULAR DOMAIN OF THE RECEPTOR TYROSINE KINASE_ KIT	AFK	0.39
2FVV	HUMAN DIPHOSPHOINOSITOL POLYPHOSPHATE PHOSPHOHYDROLASE 1	LGR	0.40
2AXN	CRYSTAL STRUCTURE OF THE HUMAN INDUCIBLE FORM 6-PHOSPHOFRUCTO-2-KINASE/FRUCTOSE-2_6-BISPHOSPHATASE	QGR	0.40
2V40	HUMAN ADENYLOSUCCINATE SYNTHETASE ISOZYME 2 IN COMPLEX WITH GDP	AFK	0.40
2O36	CRYSTAL STRUCTURE OF ENGINEERED THIMET OLIGOPEPTIDASE WITH NEUROLYSIN SPECIFICITY IN NEUROTENSIN CLEAVAGE SITE	LGR	0.40
1ZSX	CRYSTAL STRUCTURE OF HUMAN POTASSIUM CHANNEL KV BETA-SUBUNIT (KCNAB2)	EGR	0.40
1CZA	MUTANT MONOMER OF RECOMBINANT HUMAN HEXOKINASE TYPE I COMPLEXED WITH GLUCOSE_ GLUCOSE-6-PHOSPHATE_ AND ADP	EGR	0.40
1WB0	SPECIFICITY AND AFFINITY OF NATURAL PRODUCT CYCLOPENTAPEPTIDE INHIBITOR ARGIFIN AGAINST HUMAN CHITINAS	QGR	0.40
2QQ5	CRYSTAL STRUCTURE OF HUMAN SDR FAMILY MEMBER 1	QGR	0.41

2EFK	CRYSTAL STRUCTURE OF THE EFC DOMAIN OF CDC42-INTERACTING PROTEIN 4	EGR	0.41
1JTV	CRYSTAL STRUCTURE OF 17BETA-HYDROXYSTEROID DEHYDROGENASE TYPE 1 COMPLEXED WITH TESTOSTERONE	QGR	0.41
1WMA	CRYSTAL STRUCTURE OF HUMAN CBR1 IN COMPLEX WITH HYDROXY-PP	AFK	0.41
1Q20	CRYSTAL STRUCTURE OF HUMAN CHOLESTEROL SULFOTRANSFERASE (SULT2B1B) IN THE PRESENCE OF PAP AND PREGNENOLONE	LGR	0.41
1LS6	HUMAN SULT1A1 COMPLEXED WITH PAP AND P-NITROPHENOL	QAR	0.41
1EVS	CRYSTAL STRUCTURE OF HUMAN ONCOSTATIN M	LGR	0.41
2QXI	HIGH RESOLUTION STRUCTURE OF HUMAN KALLIKREIN 7 IN COMPLEX WITH SUC-ALA-ALA-PRO-PHE-CHLOROMETHYLKETONE	QAR	0.41
1M6I	CRYSTAL STRUCTURE OF APOPTOSIS INDUCING FACTOR (AIF)	LGR	0.42
1UPV	CRYSTAL STRUCTURE OF THE HUMAN LIVER X RECEPTOR BETA LIGAND BINDING DOMAIN IN COMPLEX WITH A SYNTHETIC AGONIST	LGR	0.42
1DR9	CRYSTAL STRUCTURE OF A SOLUBLE FORM OF B7-1 (CD80)	AFK	0.42
1MX3	CRYSTAL STRUCTURE OF CTBP DEHYDROGENASE CORE HOLO FORM	EGR	0.42
1L6J	CRYSTAL STRUCTURE OF HUMAN MATRIX METALLOPROTEINASE MMP9 (GELATINASE B).	LGR	0.43
1P0I	CRYSTAL STRUCTURE OF HUMAN BUTYRYL CHOLINESTERASE	LGR	0.43
1OHC	STRUCTURE OF THE PROLINE DIRECTED PHOSPHATASE CDC14	LGR	0.43
2QQJ	CRYSTAL STRUCTURE OF THE B1B2 DOMAINS FROM HUMAN NEUROPILIN 2	EAR	0.43
2B9E	HUMAN NSUN5 PROTEIN	QGR	0.43
3BQC	HIGH PH-VALUE CRYSTAL STRUCTURE OF EMODIN IN COMPLEX WITH THE CATALYTIC SUBUNIT OF PROTEIN KINASE CK2	LGR	0.43
2OU2	ACETYLTRANSFERASE DOMAIN OF HUMAN HIV-1 TAT INTERACTING PROTEIN_ 60KDA_ ISOFORM 3	LGR	0.43
1MP8	CRYSTAL STRUCTURE OF FOCAL ADHESION KINASE (FAK)	LGR	0.43
1B0F	CRYSTAL STRUCTURE OF HUMAN NEUTROPHIL ELASTASE WITH MDL 101 146	QGR	0.43
2JDF	HUMAN GAMMA-B CRYSTALLIN	QGR	0.43
1E5W	STRUCTURE OF ISOLATED FERM DOMAIN AND FIRST LONG HELIX OF MOESIN	QAR	0.44
1CZA	MUTANT MONOMER OF RECOMBINANT HUMAN HEXOKINASE TYPE I COMPLEXED WITH GLUCOSE_ GLUCOSE-6-PHOSPHATE_ AND ADP	EGR	0.44
2DQ7	CRYSTAL STRUCTURE OF FYN KINASE DOMAIN COMPLEXED WITH STAUROSPORINE	EGR	0.44
1SK4	CRYSTAL STRUCTURE OF THE C-TERMINAL PEPTIDOGLYCAN-BINDING DOMAIN OF HUMAN PEPTIDOGLYCAN RECOGNITION PROTEIN IALPHA	EAR	0.44
1YWN	VEGFR2 IN COMPLEX WITH A NOVEL 4-AMINO-FURO[2_3-D]PYRIMIDIN	LGR	0.44
1XA6	CRYSTAL STRUCTURE OF THE HUMAN BETA2-CHIMAERIN	EAR	0.44
2QLU	CRYSTAL STRUCTURE OF ACTIVIN RECEPTOR TYPE II KINASE DOMAIN FROM HUMAN	EAR	0.44
1ZIV	CATALYTIC DOMAIN OF HUMAN CALPAIN-9	EAR	0.45
3BER	HUMAN DEAD-BOX RNA-HELICASE DDX47_ CONSERVED DOMAIN I IN COMPLEX WITH AMP	QGR	0.45
1V4S	CRYSTAL STRUCTURE OF HUMAN GLUCOKINASE	EGR	0.45
1IAT	CRYSTAL STRUCTURE OF HUMAN PHOSPHOGLUCOSE ISOMERASE/NEUROLEUKIN/AUTOCRINE MOTILITY FACTOR/MATURATION FACTOR	EGR	0.45
1BX4	STRUCTURE OF HUMAN ADENOSINE KINASE AT 1.50 ANGSTROMS	QGR	0.46
3BQC	HIGH PH-VALUE CRYSTAL STRUCTURE OF EMODIN IN COMPLEX WITH THE CATALYTIC SUBUNIT OF PROTEIN KINASE CK2	LGR	0.46
2A2K	CRYSTAL STRUCTURE OF AN ACTIVE SITE MUTANT_ C473S_ OF CDC25B PHOSPHATASE CATALYTIC DOMAIN	AFK	0.46
1IMJ	CRYSTAL STRUCTURE OF THE HUMAN CCG1/TAFII250-INTERACTING FACTOR B (CIB)	QAR	0.47
2HRB	CRYSTAL STRUCTURE OF HUMAN CARBONYL REDUCTASE 3_ COMPLEXED WITH NADP+	AFK	0.47

1Z32	STRUCTURE-FUNCTION RELATIONSHIPS IN HUMAN SALIVARY ALPHA-AMYLASE: ROLE OF AROMATIC RESIDUES	QGR	0.47
2V9K	CRYSTAL STRUCTURE OF HUMAN PUS10_ A NOVEL PSEUDOURIDINE SYNTHASE.	LGR	0.47
1ZGK	1.35 ANGSTROM STRUCTURE OF THE KELCH DOMAIN OF KEAP1	QGR	0.47
1CB0	STRUCTURE OF HUMAN 5'-DEOXY-5'-METHYLTHIOADENOSINE PHOSPHORYLASE AT 1.7 A RESOLUTION	EGR	0.48
1HDR	THE CRYSTALLOGRAPHIC STRUCTURE OF A HUMAN DIHYDROPTERIDINE REDUCTASE NADH BINARY COMPLEX EXPRESSED IN ESCHERICHIA COLI BY A CDNA CONSTRUCTED FROM ITS RAT HOMOLOGUE	EAR	0.48
2IVV	CRYSTAL STRUCTURE OF PHOSPHORYLATED RET TYROSINE KINASE DOMAIN COMPLEXED WITH THE INHIBITOR PP1	AFK	0.48
1Y6B	CRYSTAL STRUCTURE OF VEGFR2 IN COMPLEX WITH A 2-ANILINO-5- ARYL-OXAZOLE INHIBITOR	LGR	0.48
2P39	CRYSTAL STRUCTURE OF HUMAN FGF23	LGR	0.48
2DE0	CRYSTAL STRUCTURE OF HUMAN ALPHA 1_6-FUCOSYLTRANSFERASE_ FUT8	LGR	0.48
1MQ4	CRYSTAL STRUCTURE OF AURORA-A PROTEIN KINASE	EGR	0.49
2B5M	CRYSTAL STRUCTURE OF DDB1	QGR	0.49
1D3G	HUMAN DIHYDROOROTATE DEHYDROGENASE COMPLEXED WITH BREQUINAR ANALOG	QGR	0.50
2PNY	STRUCTURE OF HUMAN ISOPENTENYL-DIPHOSPHATE DELTA-ISOMERASE	EAR	0.50
1JDN	CRYSTAL STRUCTURE OF HORMONE RECEPTOR	EGR	0.50
2Q5I	CRYSTAL STRUCTURE OF APO S581L GLYCYL-TRNA SYNTHETASE MUTAN	EAR	0.50
2A2C	X-RAY STRUCTURE OF HUMAN N-ACETYL GALACTOSAMINE KINASE COMPLEXED WITH MG-ADP AND N-ACETYL GALACTOSAMINE 1-PHOSPHATE	LGR	0.50
2UUI	CRYSTAL STRUCTURE OF HUMAN LEUKOTRIENE C4 SYNTHASE	LGR	0.50
2NSM	CRYSTAL STRUCTURE OF THE HUMAN CARBOXYPEPTIDASE N (KININASE I) CATALYTIC DOMAIN	EGR	0.51
1QCY	THE CRYSTAL STRUCTURE OF THE I-DOMAIN OF HUMAN INTEGRIN ALPHA1BETA1	EAR	0.52
1LF7	CRYSTAL STRUCTURE OF HUMAN COMPLEMENT PROTEIN C8GAMMA AT 1.2 A RESOLUTION	QAR	0.52
3CTZ	STRUCTURE OF HUMAN CYTOSOLIC X-PROLYL AMINOPEPTIDASE	QGR	0.52
2ALR	ALDEHYDE REDUCTASE	QAR	0.53
1ZD3	HUMAN SOLUBLE EPOXIDE HYDROLASE 4-(3-CYCLOHEXYLURIEDO)-BUTYRIC ACID COMPLEX	LGR	0.53
1HDR	THE CRYSTALLOGRAPHIC STRUCTURE OF A HUMAN DIHYDROPTERIDINE REDUCTASE NADH BINARY COMPLEX EXPRESSED IN ESCHERICHIA COLI BY A CDNA CONSTRUCTED FROM ITS RAT HOMOLOGUE	EGR	0.54
2I7Q	CRYSTAL STRUCTURE OF HUMAN CHOLINE KINASE A	EGR	0.54
2BH9	X-RAY STRUCTURE OF A DELETION VARIANT OF HUMAN GLUCOSE 6-PHOSPHATE DEHYDROGENASE COMPLEXED WITH STRUCTURAL AND COENZYME NADP	EGR	0.54
1GWZ	CRYSTAL STRUCTURE OF THE CATALYTIC DOMAIN OF THE PROTEIN TYROSINE PHOSPHATASE SHP-1	QGR	0.54
1J72	CRYSTAL STRUCTURE OF MUTANT MACROPHAGE CAPPING PROTEIN (CAP G) WITH ACTIN-SEVERING ACTIVITY IN THE CA2+-FREE FORM	QGR	0.55
1L6J	CRYSTAL STRUCTURE OF HUMAN MATRIX METALLOPROTEINASE MMP9 (GELATINASE B).	EGR	0.55
2HQQ	CRYSTAL STRUCTURE OF HUMAN KETOHEXOKINASE COMPLEXED TO DIFFERENT SUGAR MOLECULES	QGR	0.57
1JTV	CRYSTAL STRUCTURE OF 17BETA-HYDROXYSTEROID DEHYDROGENASE TYPE 1 COMPLEXED WITH TESTOSTERONE	EGR	0.58
2OIL	CRYSTAL STRUCTURE OF HUMAN RAB25 IN COMPLEX WITH GDP	QAR	0.58
2IVV	CRYSTAL STRUCTURE OF PHOSPHORYLATED RET TYROSINE KINASE DOMAIN COMPLEXED WITH THE INHIBITOR PP1	QGR	0.58

1A7S	ATOMIC RESOLUTION STRUCTURE OF HBP	QGR	0.59
1L6J	CRYSTAL STRUCTURE OF HUMAN MATRIX METALLOPROTEINASE MMP9 (GELATINASE B).	EGR	0.63
1LCY	CRYSTAL STRUCTURE OF THE MITOCHONDRIAL SERINE PROTEASE HTRA	LGR	0.63
1D7P	CRYSTAL STRUCTURE OF THE C2 DOMAIN OF HUMAN FACTOR VIII AT 1.5 A RESOLUTION AT 1.5 A	QGR	0.63
2IVV	CRYSTAL STRUCTURE OF PHOSPHORYLATED RET TYROSINE KINASE DOMAIN COMPLEXED WITH THE INHIBITOR PP1	EGR	0.64
1P6F	STRUCTURE OF THE HUMAN NATURAL CYTOTOXICITY RECEPTOR NKP46	EGR	0.64
2A8B	CRYSTAL STRUCTURE OF THE CATALYTIC DOMAIN OF HUMAN TYROSINE PHOSPHATASE RECEPTOR_ TYPE R	QGR	0.64
1R0P	CRYSTAL STRUCTURE OF THE TYROSINE KINASE DOMAIN OF THE HEPATOCYTE GROWTH FACTOR RECEPTOR C-MET IN COMPLEX WITH THE MICROBIAL ALKALOID K-252A	QGR	0.65
1N6A	STRUCTURE OF SET7/9	EGR	0.65
2EC8	CRYSTAL STRUCTURE OF THE EXCTRACELLULAR DOMAIN OF THE RECEPTOR TYROSINE KINASE_ KIT	AFK	0.65
1ZSQ	CRYSTAL STRUCTURE OF MTMR2 IN COMPLEX WITH PHOSPHATIDYLINOSITOL 3-PHOSPHATE	EGR	0.67
1CZT	CRYSTAL STRUCTURE OF THE C2 DOMAIN OF HUMAN COAGULATION FACTOR V	QGR	0.68
1W7L	CRYSTAL STRUCTURE OF HUMAN KYNURENINE AMINOTRANSFERASE I	QAR	0.71
1UV5	GLYCOGEN SYNTHASE KINASE 3 BETA COMPLEXED WITH 6-BROMOINDIRUBIN-3'-OXIME	AFK	0.72
1ELV	CRYSTAL STRUCTURE OF THE CATALYTIC DOMAIN OF HUMAN COMPLEMENT C1S PROTEASE	EGR	0.74
1GSM	A REASSESSMENT OF THE MADCAM-1 STRUCTURE AND ITS ROLE IN INTEGRIN RECOGNITION.	EGR	0.74
2EW1	CRYSTAL STRUCTURE OF RAB30 IN COMPLEX WITH A GTP ANALOGUE	EAR	0.75
2PET	LUTHERAN GLYCOPROTEIN_ N-TERMINAL DOMAINS 1 AND 2.	EGR	0.75
2O8T	CRYSTAL STRUCTURE AND BINDING EPITOPES OF UROKINASE-TYPE PLASMINOGEN ACTIVATOR (C122A/N145Q) IN COMPLEX WITH INHIBITORS	QGR	0.75
1LN1	CRYSTAL STRUCTURE OF HUMAN PHOSPHATIDYLCHOLINE TRANSFER PROTEIN IN COMPLEX WITH DILINOLEOYLPHOSPHATIDYLCHOLINE	EGR	0.77
2OI0	CRYSTAL STRUCTURE ANALYSIS 0F THE TNF-A COVERTING ENZYME (TACE) IN COMPLEXED WITH ARYL-SULFONAMIDE	EGR	0.79
1IAT	CRYSTAL STRUCTURE OF HUMAN PHOSPHOGLUCOSE ISOMERASE/NEUROLEUKIN/AUTOCRINE MOTILITY FACTOR/MATURATION FACTOR	EAR	0.79
1HGU	HUMAN GROWTH HORMONE	AFK	0.79
2IWR	GTPASE LIKE DOMAIN OF CENTAURIN GAMMA 1 (HUMAN)	EGR	0.83
1E8Y	STRUCTURE DETERMINANTS OF PHOSPHOINOSITIDE 3-KINASE INHIBITION BY WORTMANNIN_ LY294002_ QUERCETIN_ MYRICETIN AND STAUROSPORINE	AFK	2.00
1JDW	CRYSTAL STRUCTURE AND MECHANISM OF L-ARGININE: GLYCINE AMIDINOTRANSFERASE: A MITOCHONDRIAL ENZYME INVOLVED IN CREATINE BIOSYNTHESIS	LGR	2.00
1NG2	STRUCTURE OF AUTOINHIBITED P47PHOX	QAR	2.00
1R55	CRYSTAL STRUCTURE OF THE CATALYTIC DOMAIN OF HUMAN ADAM 33	EAR	2.00
1UU3	STRUCTURE OF HUMAN PDK1 KINASE DOMAIN IN COMPLEX WITH LY333531	QAR	2.00
2AEX	THE 1.58A CRYSTAL STRUCTURE OF HUMAN COPROPORPHYRINOGEN OXIDASE REVEALS THE STRUCTURAL BASIS OF HEREDITARY COPROPORPHYRIA	LGR	2.00
2NSM	CRYSTAL STRUCTURE OF THE HUMAN CARBOXYPEPTIDASE N (KININASE I) CATALYTIC DOMAIN	QAR	2.00
2NZ6	CRYSTAL STRUCTURE OF THE PTPRJ INACTIVATING MUTANT C1239S	QGR	2.00

2O8T	CRYSTAL STRUCTURE AND BINDING EPITOPES OF UROKINASE-TYPE PLASMINOGEN ACTIVATOR (C122A/N145Q) IN COMPLEX WITH INHIBITORS	EGR	2.00
2Q3H	THE CRYSTAL STRUCTURE OF RHOUA IN THE GDP-BOUND STATE.	EGR	2.00
2QOL	HUMAN EPHA3 KINASE AND JUXTAMEMBRANE REGION_ Y596:Y602:S768G TRIPLE MUTANT	LGR	2.00
2V9K	CRYSTAL STRUCTURE OF HUMAN PUS10_ A NOVEL PSEUDOURIDINE SYNTHASE.	EGR	2.00
3BQC	HIGH PH-VALUE CRYSTAL STRUCTURE OF EMODIN IN COMPLEX WITH THE CATALYTIC SUBUNIT OF PROTEIN KINASE CK2	QAR	2.00
3C8X	CRYSTAL STRUCTURE OF THE LIGAND BINDING DOMAIN OF HUMAN EPHRIN A2 (EPHA2) RECEPTOR PROTEIN KINASE	EAR	2.00
	**matriptase-3**		
2QQJ	CRYSTAL STRUCTURE OF THE B1B2 DOMAINS FROM HUMAN NEUROPILIN 2	FVR	0.12
2NR8	CRYSTAL STRUCTURE OF THE HUMAN KIF9 MOTOR DOMAIN IN COMPLEX WITH ADP	FVR	0.12
2B9E	HUMAN NSUN5 PROTEIN	FVR	0.12
2QQI	CRYSTAL STRUCTURE OF THE B1B2 DOMAINS FROM HUMAN NEUROPILIN 1	FVR	0.12
1SI5	PROTEASE-LIKE DOMAIN FROM 2-CHAIN HEPATOCYTE GROWTH FACTOR	FVR	0.12
2EC8	CRYSTAL STRUCTURE OF THE EXCTRACELLULAR DOMAIN OF THE RECEPTOR TYROSINE KINASE_ KIT	FVR	0.13
2QQJ	CRYSTAL STRUCTURE OF THE B1B2 DOMAINS FROM HUMAN NEUROPILIN 2	FVR	0.13
1A6Q	CRYSTAL STRUCTURE OF THE PROTEIN SERINE/THREONINE PHOSPHATASE 2C AT 2 A RESOLUTION	FVR	0.16
1MD8	MONOMERIC STRUCTURE OF THE ACTIVE CATALYTIC DOMAIN OF COMPLEMENT PROTEASE C1R	FVR	0.17
1NUF	ROLE OF CALCIUM IONS IN THE ACTIVATION AND ACTIVITY OF THE TRANSGLUTAMINASE 3 ENZYME	FVR	0.17
2BH9	X-RAY STRUCTURE OF A DELETION VARIANT OF HUMAN GLUCOSE 6-PHOSPHATE DEHYDROGENASE COMPLEXED WITH STRUCTURAL AND COENZYME NADP	FVR	0.17
2FY2	STRUCTURES OF LIGAND BOUND HUMAN CHOLINE ACETYLTRANSFERASE PROVIDE INSIGHT INTO REGULATION OF ACETYLCHOLINE SYNTHESIS	FVR	0.18
2PZ1	CRYSTAL STRUCTURE OF AUTO-INHIBITED ASEF	FVR	0.18
2F1W	CRYSTAL STRUCTURE OF THE TRAF-LIKE DOMAIN OF HAUSP/USP7	FVR	0.18
2A4D	STRUCTURE OF THE HUMAN UBIQUITIN-CONJUGATING ENZYME E2 VARIANT 1 (UEV-1)	FVR	0.19
2HC1	ENGINEERED CATALYTIC DOMAIN OF PROTEIN TYROSINE PHOSPHATASE HPTPBETA.	FVR	0.21
1CZA	MUTANT MONOMER OF RECOMBINANT HUMAN HEXOKINASE TYPE I COMPLEXED WITH GLUCOSE_ GLUCOSE-6-PHOSPHATE_ AND ADP	FVR	0.22
1XJD	CRYSTAL STRUCTURE OF PKC-THETA COMPLEXED WITH STAUROSPORINE AT 2A RESOLUTION	FVR	0.22
1HFC	1.56 ANGSTROM STRUCTURE OF MATURE TRUNCATED HUMAN FIBROBLAST COLLAGENASE	FVR	0.22
3C5H	CRYSTAL STRUCTURE OF THE RAS HOMOLOG DOMAIN OF HUMAN GRLF1 (P190RHOGAP)	FVR	0.22
2BKA	CC3(TIP30)CRYSTAL STRUCURE	FVR	0.22
2PBN	CRYSTAL STRUCTURE OF THE HUMAN TYROSINE RECEPTOR PHOSPHATE GAMMA	FVR	0.22
1N3Y	CRYSTAL STRUCTURE OF THE ALPHA-X BETA2 INTEGRIN I DOMAIN	FVR	0.22
2II0	CRYSTAL STRUCTURE OF CATALYTIC DOMAIN OF SON OF SEVENLESS (REM-CDC25) IN THE ABSENCE OF RAS	FVR	0.23
2NZ2	CRYSTAL STRUCTURE OF HUMAN ARGININOSUCCINATE SYNTHASE IN COMPLEX WITH ASPARTATE AND CITRULLINE	FVR	0.24
1CZA	MUTANT MONOMER OF RECOMBINANT HUMAN HEXOKINASE TYPE I COMPLEXED WITH GLUCOSE_ GLUCOSE-6-PHOSPHATE_ AND ADP	FVR	0.24

2QTZ	CRYSTAL STRUCTURE OF THE NADP+-BOUND FAD-CONTAINING FNR- LIKE MODULE OF HUMAN METHIONINE SYNTHASE REDUCTASE	FVR	0.24
1B0F	CRYSTAL STRUCTURE OF HUMAN NEUTROPHIL ELASTASE WITH MDL 101 146	FVR	0.29
1IJB	THE VON WILLEBRAND FACTOR MUTANT (I546V) A1 DOMAIN	FVR	0.30
2AA2	MINERALOCORTICOID RECEPTOR WITH BOUND ALDOSTERONE	FVR	0.31
	**mesotrypsin**		
2A2K	CRYSTAL STRUCTURE OF AN ACTIVE SITE MUTANT_ C473S_ OF CDC25B PHOSPHATASE CATALYTIC DOMAIN	GPR	0.19
1F5N	HUMAN GUANYLATE BINDING PROTEIN-1 IN COMPLEX WITH THE GTP ANALOGUE_ GMPPNP.	GPR	0.19
1C25	HUMAN CDC25A CATALYTIC DOMAIN	GPR	0.21
1IAT	CRYSTAL STRUCTURE OF HUMAN PHOSPHOGLUCOSE ISOMERASE/NEUROLEUKIN/AUTOCRINE MOTILITY FACTOR/MATURATION FACTOR	GPR	0.27
1KT0	STRUCTURE OF THE LARGE FKBP-LIKE PROTEIN_ FKBP51_ INVOLVED IN STEROID RECEPTOR COMPLEXES	GPR	0.28
2B69	CRYSTAL STRUCTURE OF HUMAN UDP-GLUCORONIC ACID DECARBOXYLAS	GPR	0.28
2JHM	STRUCTURE OF GLOBULAR HEADS OF M-FICOLIN AT NEUTRAL PH	GPR	0.29
2Z6O	CRYSTAL STRUCTURE OF THE UFC1_ UFM1 CONJUGATING ENZYME 1	GPR	0.30
1KO9	NATIVE STRUCTURE OF THE HUMAN 8-OXOGUANINE DNA GLYCOSYLASE HOGG1	GPR	0.30
1TQN	CRYSTAL STRUCTURE OF HUMAN MICROSOMAL P450 3A4	GPR	0.34
1ZVD	REGULATION OF SMURF2 UBIQUITIN LIGASE ACTIVITY BY ANCHORING THE E2 TO THE HECT DOMAIN	GPR	0.34
2HI4	CRYSTAL STRUCTURE OF HUMAN MICROSOMAL P450 1A2 IN COMPLEX WITH ALPHA-NAPHTHOFLAVONE	GPR	0.35
1D2S	CRYSTAL STRUCTURE OF THE N-TERMINAL LAMININ G-LIKE DOMAIN OF SHBG IN COMPLEX WITH DIHYDROTESTOSTERONE	GPR	0.38
2B69	CRYSTAL STRUCTURE OF HUMAN UDP-GLUCORONIC ACID DECARBOXYLAS	GPR	0.40
1N26	CRYSTAL STRUCTURE OF THE EXTRA-CELLULAR DOMAINS OF HUMAN INTERLEUKIN-6 RECEPTOR ALPHA CHAIN	GPR	0.41
1S31	CRYSTAL STRUCTURE ANALYSIS OF THE HUMAN TUB PROTEIN (ISOFORM A) SPANNING RESIDUES 289 THROUGH 561	GPR	0.44
1HDO	HUMAN BILIVERDIN IX BETA REDUCTASE: NADP COMPLEX	GPR	0.45
3CHO	CRYSTAL STRUCTURE OF LEUKOTRIENE A4 HYDROLASE IN COMPLEX WITH 2-AMINO-N-[4-(PHENYLMETHOXY)PHENYL]-ACETAMIDE	GPR	0.48
1CB0	STRUCTURE OF HUMAN 5'-DEOXY-5'-METHYLTHIOADENOSINE PHOSPHORYLASE AT 1.7 A RESOLUTION	GPR	0.50
1Q33	CRYSTAL STRUCTURE OF HUMAN ADP-RIBOSE PYROPHOSPHATASE NUDT9	GPR	0.53
1OZ2	CRYSTAL STRUCTURE OF 3-MBT REPEATS OF LETHAL (3) MALIGNANT BRAIN TUMOR (NATIVE-II) AT 1.55 ANGSTROM	GPR	0.54
1TDH	CRYSTAL STRUCTURE OF HUMAN ENDONUCLEASE VIII-LIKE 1 (NEIL1)	GPR	0.56
1PME	STRUCTURE OF PENTA MUTANT HUMAN ERK2 MAP KINASE COMPLEXED WITH A SPECIFIC INHIBITOR OF HUMAN P38 MAP KINASE	GPR	0.60
1PI1	CRYSTAL STRUCTURE OF A HUMAN MOB1 PROTEIN; TOWARD UNDERSTANDING MOB-REGULATED CELL CYCLE PATHWAYS.	GPR	0.61
1VZO	THE STRUCTURE OF THE N-TERMINAL KINASE DOMAIN OF MSK1 REVEALS A NOVEL AUTOINHIBITORY CONFORMATION FOR A DUAL KINASE PROTEIN	GPR	0.62
3COU	CRYSTAL STRUCTURE OF HUMAN NUDIX MOTIF 16 (NUDT16)	GPR	0.73
1CZA	MUTANT MONOMER OF RECOMBINANT HUMAN HEXOKINASE TYPE I COMPLEXED WITH GLUCOSE_ GLUCOSE-6-PHOSPHATE_ AND ADP	GPR	0.74
2VPJ	CRYSTAL STRUCTURE OF THE KELCH DOMAIN OF HUMAN KLHL12	GPR	2.00
	**myeloblastin**		
2RIP	STRUCTURE OF DPPIV IN COMPLEX WITH AN INHIBITOR	APV	0.07

1B0F	CRYSTAL STRUCTURE OF HUMAN NEUTROPHIL ELASTASE WITH MDL 101 146	APV	0.09
2HZP	CRYSTAL STRUCTURE OF HOMO SAPIENS KYNURENINASE	APV	0.10
1IAT	CRYSTAL STRUCTURE OF HUMAN PHOSPHOGLUCOSE ISOMERASE/NEUROLEUKIN/AUTOCRINE MOTILITY FACTOR/MATURATION FACTOR	APV	0.11
2OIT	CRYSTAL STRUCTURE OF THE N-TERMINAL DOMAIN OF THE HUMAN PROTO-ONCOGENE NUP214/CAN	APV	0.12
1H30	C-TERMINAL LG DOMAIN PAIR OF HUMAN GAS6	APV	0.16
1PSN	THE CRYSTAL STRUCTURE OF HUMAN PEPSIN AND ITS COMPLEX WITH PEPSTATIN	APV	0.18
1Z70	1.15A RESOLUTION STRUCTURE OF THE FORMYLGLYCINE GENERATING ENZYME FGE	APV	0.18
1H4W	STRUCTURE OF HUMAN TRYPSIN IV (BRAIN TRYPSIN)	APV	0.19
1R55	CRYSTAL STRUCTURE OF THE CATALYTIC DOMAIN OF HUMAN ADAM 33	APV	0.20
1JDN	CRYSTAL STRUCTURE OF HORMONE RECEPTOR	APV	0.20
2DW5	CRYSTAL STRUCTURE OF HUMAN PEPTIDYLARGININE DEIMINASE 4 IN COMPLEX WITH N-ALPHA-BENZOYL-N5-(2-FLUORO-1-IMINOETHYL)-L-ORNITHINE AMIDE	APV	0.21
1JNX	CRYSTAL STRUCTURE OF THE BRCT REPEAT REGION FROM THE BREAST CANCER ASSOCIATED PROTEIN_ BRCA1	APV	0.21
2DH2	CRYSTAL STRUCTURE OF HUMAN ED-4F2HC	APV	0.22
2OKK	THE X-RAY CRYSTAL STRUCTURE OF THE 65KDA ISOFORM OF GLUTAMIC ACID DECARBOXYLASE (GAD65)	APV	0.23
3C0I	CASK CAM-KINASE DOMAIN- 3'-AMP COMPLEX_ P212121 FORM	APV	0.25
3BPT	CRYSTAL STRUCTURE OF HUMAN BETA-HYDROXYISOBUTYRYL-COA HYDROLASE IN COMPLEX WITH QUERCETIN	APV	0.28
2P0K	CRYSTAL STRUCTURE OF SCMH1	APV	0.29
2OKK	THE X-RAY CRYSTAL STRUCTURE OF THE 65KDA ISOFORM OF GLUTAMIC ACID DECARBOXYLASE (GAD65)	APV	0.30
1R3S	UROPORPHYRINOGEN DECARBOXYLASE SINGLE MUTANT D86G IN COMPLEX WITH COPROPORPHYRINOGEN-I	APV	0.30
1OZ2	CRYSTAL STRUCTURE OF 3-MBT REPEATS OF LETHAL (3) MALIGNANT BRAIN TUMOR (NATIVE-II) AT 1.55 ANGSTROM	APV	0.31
1I1N	HUMAN PROTEIN L-ISOASPARTATE O-METHYLTRANSFERASE WITH S-ADENOSYL HOMOCYSTEINE	APV	0.32
2GJK	STRUCTURAL AND FUNCTIONAL INSIGHTS INTO THE HUMAN UPF1 HELICASE CORE	APV	0.36
2VKT	HUMAN CTP SYNTHETASE 2 - GLUTAMINASE DOMAIN	APV	0.42
3CTZ	STRUCTURE OF HUMAN CYTOSOLIC X-PROLYL AMINOPEPTIDASE	APV	0.43
1ORE	HUMAN ADENINE PHOSPHORIBOSYLTRANSFERASE	APV	0.47
1LB4	TRAF6 APO STRUCTURE	APV	0.75
1ALU	HUMAN INTERLEUKIN-6	APV	2.00
1L2H	CRYSTAL STRUCTURE OF INTERLEUKIN 1-BETA F42W/W120F MUTANT	APV	2.00
1M6I	CRYSTAL STRUCTURE OF APOPTOSIS INDUCING FACTOR (AIF)	APV	2.00
1X9D	CRYSTAL STRUCTURE OF HUMAN CLASS I ALPHA-1_2-MANNOSIDASE IN COMPLEX WITH THIO-DISACCHARIDE SUBSTRATE ANALOGUE	APV	2.00
1XMJ	CRYSTAL STRUCTURE OF HUMAN DELTAF508 HUMAN NBD1 DOMAIN WITH ATP	APV	2.00
2FAU	CRYSTAL STRUCTURE OF HUMAN VPS26	APV	2.00
2OPW	CRYSTAL STRUCTURE OF HUMAN PHYTANOYL-COA DIOXYGENASE PHYHD1 (APO)	APV	2.00
2QTZ	CRYSTAL STRUCTURE OF THE NADP+-BOUND FAD-CONTAINING FNR- LIKE MODULE OF HUMAN METHIONINE SYNTHASE REDUCTASE	APV	2.00
	**plasma kallikrein**		
1CZT	CRYSTAL STRUCTURE OF THE C2 DOMAIN OF HUMAN COAGULATION FACTOR V	PFR	0.14

2FY7	CRYSTAL STRUCTURE OF THE CATALYTIC DOMAIN OF THE HUMAN BETA1_4-GALACTOSYLTRANSFERASE MUTANT M339H IN APO FORM	PFR	0.18
2JHM	STRUCTURE OF GLOBULAR HEADS OF M-FICOLIN AT NEUTRAL PH	QRR	0.20
3BIY	CRYSTAL STRUCTURE OF P300 HISTONE ACETYLTRANSFERASE DOMAIN IN COMPLEX WITH A BISUBSTRATE INHIBITOR_ LYS-COA	QRR	0.20
1OHC	STRUCTURE OF THE PROLINE DIRECTED PHOSPHATASE CDC14	PFR	0.21
2HE3	CRYSTAL STRUCTURE OF THE SELENOCYSTEINE TO CYSTEINE MUTANT OF HUMAN GLUTATHIONINE PEROXIDASE 2 (GPX2)	PFR	0.21
1D5R	CRYSTAL STRUCTURE OF THE PTEN TUMOR SUPPRESSOR	QRR	0.21
2A1X	HUMAN PHYTANOYL-COA 2-HYDROXYLASE IN COMPLEX WITH IRON AND 2-OXOGLUTARATE	PFR	0.22
1UU3	STRUCTURE OF HUMAN PDK1 KINASE DOMAIN IN COMPLEX WITH LY333531	PFR	0.23
1XQJ	3.10 A CRYSTAL STRUCTURE OF MASPIN_ SPACE GROUP I 4 2 2	PFR	0.23
1BY7	HUMAN PLASMINOGEN ACTIVATOR INHIBITOR-2. LOOP (66-98) DELETION MUTANT	PFR	0.23
1Z57	CRYSTAL STRUCTURE OF HUMAN CLK1 IN COMPLEX WITH 10Z-HYMENIALDISINE	PFR	0.24
2DHO	CRYSTAL STRUCTURE OF HUMAN IPP ISOMERASE I IN SPACE GROUP P212121	QRR	0.26
2PNY	STRUCTURE OF HUMAN ISOPENTENYL-DIPHOSPHATE DELTA-ISOMERASE	QRR	0.26
2GRY	CRYSTAL STRUCTURE OF THE HUMAN KIF2 MOTOR DOMAIN IN COMPLEX WITH ADP	PFR	0.27
2A7V	HUMAN MITOCHONDRIAL SERINE HYDROXYMETHYLTRANSFERASE 2	QRR	0.27
2HEH	CRYSTAL STRUCTURE OF THE KIF2C MOTOR DOMAIN (CASP TARGET)	PFR	0.27
2Z5Y	CRYSTAL STRUCTURE OF HUMAN MONOAMINE OXIDASE A (G110A) WITH HARMINE	PFR	0.28
1T5I	CRYSTAL STRUCTURE OF THE C-TERMINAL DOMAIN OF UAP56	QRR	0.31
1Z32	STRUCTURE-FUNCTION RELATIONSHIPS IN HUMAN SALIVARY ALPHA-AMYLASE: ROLE OF AROMATIC RESIDUES	PFR	0.33
2H58	CRYSTAL STRUCTURE OF THE KIFC3 MOTOR DOMAIN IN COMPLEX WITH ADP	PFR	0.33
2DE0	CRYSTAL STRUCTURE OF HUMAN ALPHA 1_6-FUCOSYLTRANSFERASE_ FUT8	QRR	0.35
2REI	KINASE DOMAIN OF HUMAN EPHRIN TYPE-A RECEPTOR 7 (EPHA7)	QRR	0.35
2IPX	HUMAN FIBRILLARIN	PFR	0.35
1E8Y	STRUCTURE DETERMINANTS OF PHOSPHOINOSITIDE 3-KINASE INHIBITION BY WORTMANNIN_ LY294002_ QUERCETIN_ MYRICETIN AND STAUROSPORINE	QRR	0.37
1IAP	CRYSTAL STRUCTURE OF P115RHOGEF RGRGS DOMAIN	QRR	0.38
1LN1	CRYSTAL STRUCTURE OF HUMAN PHOSPHATIDYLCHOLINE TRANSFER PROTEIN IN COMPLEX WITH DILINOLEOYLPHOSPHATIDYLCHOLINE	QRR	0.38
2NR8	CRYSTAL STRUCTURE OF THE HUMAN KIF9 MOTOR DOMAIN IN COMPLEX WITH ADP	PFR	0.39
1TXD	CRYSTAL STRUCTURE OF THE DH/PH DOMAINS OF LEUKEMIA-ASSOCIATED RHOGEF	QRR	0.39
1IPC	CRYSTAL STRUCTURE OF EUKARYOTIC INITIATION FACTOR 4E COMPLEXED WITH 7-METHYL GTP	QRR	0.39
2QOL	HUMAN EPHA3 KINASE AND JUXTAMEMBRANE REGION_ Y596:Y602:S768G TRIPLE MUTANT	QRR	0.41
2R2P	KINASE DOMAIN OF HUMAN EPHRIN TYPE-A RECEPTOR 5 (EPHA5)	QRR	0.42
3COI	CRYSTAL STRUCTURE OF P38DELTA KINASE	PFR	0.42
2HYV	HUMAN ANNEXIN A2 WITH HEPARIN HEXASACCHARIDE BOUND	QRR	0.42
3BO5	CRYSTAL STRUCTURE OF METHYLTRANSFERASE DOMAIN OF HUMAN HISTONE-LYSINE N-METHYLTRANSFERASE SETMAR	QRR	0.43
2D7I	CRSYTAL STRUCTURE OF PP-GALNAC-T10 WITH UDP_ GALNAC AND MN2	QRR	0.46
1N7D	EXTRACELLULAR DOMAIN OF THE LDL RECEPTOR	QRR	0.46

2H79	CRYSTAL STRUCTURE OF HUMAN TR ALPHA BOUND T3 IN ORTHORHOMBIC SPACE GROUP	QRR	0.51
1L8K	T CELL PROTEIN-TYROSINE PHOSPHATASE STRUCTURE	QRR	0.51
2J51	CRYSTAL STRUCTURE OF HUMAN STE20-LIKE KINASE BOUND TO 5- AMINO-3-((4-(AMINOSULFONYL)PHENYL)AMINO)-N-(2_6- DIFLUOROPHENYL)-1H-1_2_4-TRIAZOLE-1-CARBOTHIOAMIDE	QRR	0.53
2C30	CRYSTAL STRUCTURE OF THE HUMAN P21-ACTIVATED KINASE 6	QRR	0.53
2PQ8	MYST HISTONE ACETYLTRANSFERASE 1	QRR	0.54
2J0I	CRYSTAL STRUCTURE OF THE HUMAN P21-ACTIVATED KINASE 4	QRR	0.55
2HJW	CRYSTAL STRUCTURE OF THE BC DOMAIN OF ACC2	QRR	0.59
2OU2	ACETYLTRANSFERASE DOMAIN OF HUMAN HIV-1 TAT INTERACTING PROTEIN_ 60KDA_ ISOFORM 3	QRR	0.59
1T32	A DUAL INHIBITOR OF THE LEUKOCYTE PROTEASES CATHEPSIN G AND CHYMASE WITH THERAPEUTIC EFFICACY IN ANIMALS MODELS OF INFLAMMATION	QRR	0.62
2C9H	STRUCTURE OF MITOCHONDRIAL BETA-KETOACYL SYNTHASE	QRR	0.64
2JC9	CRYSTAL STRUCTURE OF HUMAN CYTOSOLIC 5'-NUCLEOTIDASE II IN COMPLEX WITH ADENOSINE	QRR	0.65
2Z6H	CRYSTAL STRUCTURE OF BETA-CATENIN ARMADILLO REPEAT REGION AND ITS C-TERMINAL DOMAIN	QRR	0.75
3B7O	CRYSTAL STRUCTURE OF THE HUMAN TYROSINE PHOSPHATASE SHP2 (PTPN11) WITH AN ACCESSIBLE ACTIVE SITE	QRR	0.76
1LCY	CRYSTAL STRUCTURE OF THE MITOCHONDRIAL SERINE PROTEASE HTRA	QRR	2.00
1NG2	STRUCTURE OF AUTOINHIBITED P47PHOX	QRR	2.00
1S1E	CRYSTAL STRUCTURE OF KV CHANNEL-INTERACTING PROTEIN 1 (KCHIP-1)	QRR	2.00
1XJD	CRYSTAL STRUCTURE OF PKC-THETA COMPLEXED WITH STAUROSPORINE AT 2A RESOLUTION	PFR	2.00
2POM	TAB1 WITH MANGANESE ION	QRR	2.00
	**plasmin**		
2B3X	STRUCTURE OF AN ORTHORHOMBIC CRYSTAL FORM OF HUMAN CYTOSOLIC ACONITASE (IRP1)	GPK	0.15
1JNX	CRYSTAL STRUCTURE OF THE BRCT REPEAT REGION FROM THE BREAST CANCER ASSOCIATED PROTEIN_ BRCA1	GPK	0.17
1YHV	CRYSTAL STRUCTURE OF PAK1 KINASE DOMAIN WITH TWO POINT MUTATIONS (K299R_ T423E)	GPK	0.22
2JC9	CRYSTAL STRUCTURE OF HUMAN CYTOSOLIC 5'-NUCLEOTIDASE II IN COMPLEX WITH ADENOSINE	GPK	0.23
2I7Q	CRYSTAL STRUCTURE OF HUMAN CHOLINE KINASE A	GPK	0.24
2DW5	CRYSTAL STRUCTURE OF HUMAN PEPTIDYLARGININE DEIMINASE 4 IN COMPLEX WITH N-ALPHA-BENZOYL-N5-(2-FLUORO-1-IMINOETHYL)-L-ORNITHINE AMIDE	GPK	0.33
3BZH	CRYSTAL STRUCTURE OF HUMAN UBIQUITIN-CONJUGATING ENZYME E2 E1	GPK	0.34
1MFM	MONOMERIC HUMAN SOD MUTANT F50E/G51E/E133Q AT ATOMIC RESOLUTION	GPK	0.34
1NUF	ROLE OF CALCIUM IONS IN THE ACTIVATION AND ACTIVITY OF THE TRANSGLUTAMINASE 3 ENZYME	GPK	0.35
2CBZ	STRUCTURE OF THE HUMAN MULTIDRUG RESISTANCE PROTEIN 1 NUCLEOTIDE BINDING DOMAIN 1	GPK	0.37
1QCY	THE CRYSTAL STRUCTURE OF THE I-DOMAIN OF HUMAN INTEGRIN ALPHA1BETA1	GPK	0.40
2A91	CRYSTAL STRUCTURE OF ERBB2 DOMAINS 1-3	GPK	0.41
1NM8	STRUCTURE OF HUMAN CARNITINE ACETYLTRANSFERASE: MOLECULAR BASIS FOR FATTY ACYL TRANSFER	GPK	0.43
1EQF	CRYSTAL STRUCTURE OF THE DOUBLE BROMODOMAIN MODULE FROM HUMAN TAFII250	GPK	0.45

2A4D	STRUCTURE OF THE HUMAN UBIQUITIN-CONJUGATING ENZYME E2 VARIANT 1 (UEV-1)	GPK	0.48
2GFO	STRUCTURE OF THE CATALYTIC DOMAIN OF HUMAN UBIQUITIN CARBOXYL-TERMINAL HYDROLASE 8	GPK	0.49
1NKR	INHIBITORY RECEPTOR (P58-CL42) FOR HUMAN NATURAL KILLER CELLS	GPK	0.49
3PBH	REFINED CRYSTAL STRUCTURE OF HUMAN PROCATHEPSIN B AT 2.5 ANGSTROM RESOLUTION	GPK	0.50
1J72	CRYSTAL STRUCTURE OF MUTANT MACROPHAGE CAPPING PROTEIN (CAP G) WITH ACTIN-SEVERING ACTIVITY IN THE CA2+-FREE FORM	GPK	0.54
1IAP	CRYSTAL STRUCTURE OF P115RHOGEF RGRGS DOMAIN	GPK	0.58
2CZK	CRYSTAL STRUCTURE OF HUMAN MYO-INOSITOL MONOPHOSPHATASE 2 (IMPA2) (TRIGONAL FORM)	GPK	0.58
1WMA	CRYSTAL STRUCTURE OF HUMAN CBR1 IN COMPLEX WITH HYDROXY-PP	GPK	0.59
2DW5	CRYSTAL STRUCTURE OF HUMAN PEPTIDYLARGININE DEIMINASE 4 IN COMPLEX WITH N-ALPHA-BENZOYL-N5-(2-FLUORO-1-IMINOETHYL)-L-ORNITHINE AMIDE	GPK	0.74
1M4K	CRYSTAL STRUCTURE OF THE HUMAN NATURAL KILLER CELL ACTIVATOR RECEPTOR KIR2DS2 (CD158J)	GPK	0.79
1A17	TETRATRICOPEPTIDE REPEATS OF PROTEIN PHOSPHATASE 5	GPK	2.00
1B6A	HUMAN METHIONINE AMINOPEPTIDASE 2 COMPLEXED WITH TNP-470	GPK	2.00
1HU3	MIDDLE DOMAIN OF HUMAN EIF4GII	GPK	2.00
2AXN	CRYSTAL STRUCTURE OF THE HUMAN INDUCIBLE FORM 6-PHOSPHOFRUCTO-2-KINASE/FRUCTOSE-2_6-BISPHOSPHATASE	GPK	2.00
2R3A	METHYLTRANSFERASE DOMAIN OF HUMAN SUPPRESSOR OF VARIEGATION 3-9 HOMOLOG 2	GPK	2.00
	**prostasin**		
2A2K	CRYSTAL STRUCTURE OF AN ACTIVE SITE MUTANT_ C473S_ OF CDC25B PHOSPHATASE CATALYTIC DOMAIN	GPR	0.19
1F5N	HUMAN GUANYLATE BINDING PROTEIN-1 IN COMPLEX WITH THE GTP ANALOGUE_ GMPPNP.	GPR	0.19
1C25	HUMAN CDC25A CATALYTIC DOMAIN	GPR	0.21
1IAT	CRYSTAL STRUCTURE OF HUMAN PHOSPHOGLUCOSE ISOMERASE/NEUROLEUKIN/AUTOCRINE MOTILITY FACTOR/MATURATION FACTOR	GPR	0.27
1KT0	STRUCTURE OF THE LARGE FKBP-LIKE PROTEIN_ FKBP51_ INVOLVED IN STEROID RECEPTOR COMPLEXES	GPR	0.28
2B69	CRYSTAL STRUCTURE OF HUMAN UDP-GLUCORONIC ACID DECARBOXYLAS	GPR	0.28
2JHM	STRUCTURE OF GLOBULAR HEADS OF M-FICOLIN AT NEUTRAL PH	GPR	0.29
2Z6O	CRYSTAL STRUCTURE OF THE UFC1_ UFM1 CONJUGATING ENZYME 1	GPR	0.30
1KO9	NATIVE STRUCTURE OF THE HUMAN 8-OXOGUANINE DNA GLYCOSYLASE HOGG1	GPR	0.30
1TQN	CRYSTAL STRUCTURE OF HUMAN MICROSOMAL P450 3A4	GPR	0.34
1ZVD	REGULATION OF SMURF2 UBIQUITIN LIGASE ACTIVITY BY ANCHORING THE E2 TO THE HECT DOMAIN	GPR	0.34
2HI4	CRYSTAL STRUCTURE OF HUMAN MICROSOMAL P450 1A2 IN COMPLEX WITH ALPHA-NAPHTHOFLAVONE	GPR	0.35
1D2S	CRYSTAL STRUCTURE OF THE N-TERMINAL LAMININ G-LIKE DOMAIN OF SHBG IN COMPLEX WITH DIHYDROTESTOSTERONE	GPR	0.38
2B69	CRYSTAL STRUCTURE OF HUMAN UDP-GLUCORONIC ACID DECARBOXYLAS	GPR	0.40
1N26	CRYSTAL STRUCTURE OF THE EXTRA-CELLULAR DOMAINS OF HUMAN INTERLEUKIN-6 RECEPTOR ALPHA CHAIN	GPR	0.41
1S31	CRYSTAL STRUCTURE ANALYSIS OF THE HUMAN TUB PROTEIN (ISOFORM A) SPANNING RESIDUES 289 THROUGH 561	GPR	0.44
1HDO	HUMAN BILIVERDIN IX BETA REDUCTASE: NADP COMPLEX	GPR	0.45
3CHO	CRYSTAL STRUCTURE OF LEUKOTRIENE A4 HYDROLASE IN COMPLEX WITH 2-AMINO-N-[4-(PHENYLMETHOXY)PHENYL]-ACETAMIDE	GPR	0.48

1CB0	STRUCTURE OF HUMAN 5'-DEOXY-5'-METHYLTHIOADENOSINE PHOSPHORYLASE AT 1.7 A RESOLUTION	GPR	0.50
1Q33	CRYSTAL STRUCTURE OF HUMAN ADP-RIBOSE PYROPHOSPHATASE NUDT9	GPR	0.53
1OZ2	CRYSTAL STRUCTURE OF 3-MBT REPEATS OF LETHAL (3) MALIGNANT BRAIN TUMOR (NATIVE-II) AT 1.55 ANGSTROM	GPR	0.54
1TDH	CRYSTAL STRUCTURE OF HUMAN ENDONUCLEASE VIII-LIKE 1 (NEIL1)	GPR	0.56
1PME	STRUCTURE OF PENTA MUTANT HUMAN ERK2 MAP KINASE COMPLEXED WITH A SPECIFIC INHIBITOR OF HUMAN P38 MAP KINASE	GPR	0.60
1PI1	CRYSTAL STRUCTURE OF A HUMAN MOB1 PROTEIN; TOWARD UNDERSTANDING MOB-REGULATED CELL CYCLE PATHWAYS.	GPR	0.61
1VZO	THE STRUCTURE OF THE N-TERMINAL KINASE DOMAIN OF MSK1 REVEALS A NOVEL AUTOINHIBITORY CONFORMATION FOR A DUAL KINASE PROTEIN	GPR	0.62
3COU	CRYSTAL STRUCTURE OF HUMAN NUDIX MOTIF 16 (NUDT16)	GPR	0.73
1CZA	MUTANT MONOMER OF RECOMBINANT HUMAN HEXOKINASE TYPE I COMPLEXED WITH GLUCOSE_ GLUCOSE-6-PHOSPHATE_ AND ADP	GPR	0.74
2VPJ	CRYSTAL STRUCTURE OF THE KELCH DOMAIN OF HUMAN KLHL12	GPR	2.00
	**protein C**		
2G62	CRYSTAL STRUCTURE OF HUMAN PTPA	EPR	0.28
2IUW	CRYSTAL STRUCTURE OF HUMAN ABH3 IN COMPLEX WITH IRON ION AND 2-OXOGLUTARATE	EPR	0.29
1X9D	CRYSTAL STRUCTURE OF HUMAN CLASS I ALPHA-1_2-MANNOSIDASE IN COMPLEX WITH THIO-DISACCHARIDE SUBSTRATE ANALOGUE	EPR	0.32
3BCH	CRYSTAL STRUCTURE OF THE HUMAN LAMININ RECEPTOR PRECURSOR	EPR	0.32
2I7Q	CRYSTAL STRUCTURE OF HUMAN CHOLINE KINASE A	EPR	0.37
2II0	CRYSTAL STRUCTURE OF CATALYTIC DOMAIN OF SON OF SEVENLESS (REM-CDC25) IN THE ABSENCE OF RAS	EPR	0.37
1YPV	STRUCTURE OF HUMAN THYMIDYLATE SYNTHASE AT LOW SALT CONDITIONS	EPR	0.38
1Z5V	CRYSTAL STRUCTURE OF HUMAN GAMMA-TUBULIN BOUND TO GTPGAMMAS	EPR	0.48
1RYO	HUMAN SERUM TRANSFERRIN_ N-LOBE BOUND WITH OXALATE	EPR	0.52
1F5N	HUMAN GUANYLATE BINDING PROTEIN-1 IN COMPLEX WITH THE GTP ANALOGUE_ GMPPNP.	EPR	0.54
2CGO	FACTOR INHIBITING HIF-1 ALPHA WITH FUMARATE	EPR	0.55
1AOA	N-TERMINAL ACTIN-CROSSLINKING DOMAIN FROM HUMAN FIMBRIN	EPR	0.56
1P49	STRUCTURE OF HUMAN PLACENTAL ESTRONE/DHEA SULFATASE	EPR	0.57
2I7Q	CRYSTAL STRUCTURE OF HUMAN CHOLINE KINASE A	EPR	0.64
2QIS	CRYSTAL STRUCTURE OF HUMAN FARNESYL PYROPHOSPHATE SYNTHASE T210S MUTANT BOUND TO RISEDRONATE	EPR	0.68
1HH8	THE ACTIVE N-TERMINAL REGION OF P67PHOX: STRUCTURE AT 1.8 ANGSTROM RESOLUTION AND BIOCHEMICAL CHARACTERIZATIONS OF THE A128V MUTANT IMPLICATED IN CHRONIC GRANULOMATOUS DISEASE	EPR	0.74
1UU3	STRUCTURE OF HUMAN PDK1 KINASE DOMAIN IN COMPLEX WITH LY333531	EPR	2.00
1WOJ	CRYSTAL STRUCTURE OF HUMAN PHOSPHODIESTERASE	EPR	2.00
1XA6	CRYSTAL STRUCTURE OF THE HUMAN BETA2-CHIMAERIN	EPR	2.00
2YU1	CRYSTAL STRUCTURE OF HJHDM1A COMPLEXED WITH A-KETOGLUTARATE	EPR	2.00
	**testisin**		
1JDN	CRYSTAL STRUCTURE OF HORMONE RECEPTOR	LTR	0.11
2QMJ	CRYSTRAL STRUCTURE OF THE N-TERMINAL SUBUNIT OF HUMAN MALTASE-GLUCOAMYLASE IN COMPLEX WITH ACARBOSE	FSR	0.11
2QMJ	CRYSTRAL STRUCTURE OF THE N-TERMINAL SUBUNIT OF HUMAN MALTASE-GLUCOAMYLASE IN COMPLEX WITH ACARBOSE	LTR	0.12
1JYD	CRYSTAL STRUCTURE OF RECOMBINANT HUMAN SERUM RETINOL-BINDING PROTEIN AT 1.7 A RESOLUTION	FSR	0.15

2GJK	STRUCTURAL AND FUNCTIONAL INSIGHTS INTO THE HUMAN UPF1 HELICASE CORE	LTR	0.15
2QQJ	CRYSTAL STRUCTURE OF THE B1B2 DOMAINS FROM HUMAN NEUROPILIN 2	LTR	0.15
2I7V	STRUCTURE OF HUMAN CPSF-73	FSR	0.15
2J8Z	CRYSTAL STRUCTURE OF HUMAN P53 INDUCIBLE OXIDOREDUCTASE (TP53I3_PIG3)	LTR	0.15
1JY1	CRYSTAL STRUCTURE OF HUMAN TYROSYL-DNA PHOSPHODIESTERASE (TDP1)	LTR	0.16
2OHF	CRYSTAL STRUCTURE OF HUMAN OLA1 IN COMPLEX WITH AMPPCP	LTR	0.16
1YVJ	CRYSTAL STRUCTURE OF THE JAK3 KINASE DOMAIN IN COMPLEX WITH A STAUROSPORINE ANALOGUE	FSR	0.16
1XKS	THE CRYSTAL STRUCTURE OF THE N-TERMINAL DOMAIN OF NUP133 REVEALS A BETA-PROPELLER FOLD COMMON TO SEVERAL NUCLEOPORINS	FSR	0.16
1R03	CRYSTAL STRUCTURE OF A HUMAN MITOCHONDRIAL FERRITIN	FSR	0.17
1R3S	UROPORPHYRINOGEN DECARBOXYLASE SINGLE MUTANT D86G IN COMPLEX WITH COPROPORPHYRINOGEN-I	LTR	0.17
1JDN	CRYSTAL STRUCTURE OF HORMONE RECEPTOR	LTR	0.17
2DHO	CRYSTAL STRUCTURE OF HUMAN IPP ISOMERASE I IN SPACE GROUP P212121	LTR	0.18
2ILR	CRYSTAL STRUCTURE OF HUMAN FANCONI ANEMIA PROTEIN E C-TERMINAL DOMAIN	LTR	0.18
2AXN	CRYSTAL STRUCTURE OF THE HUMAN INDUCIBLE FORM 6-PHOSPHOFRUCTO-2-KINASE/FRUCTOSE-2_6-BISPHOSPHATASE	LTR	0.19
2B3H	CRYSTAL STRUCTURE OF HUMAN METHIONINE AMINOPEPTIDASE TYPE I WITH A THIRD COBALT IN THE ACTIVE SITE	LTR	0.20
2PZ1	CRYSTAL STRUCTURE OF AUTO-INHIBITED ASEF	LTR	0.20
1H6O	DIMERISATION DOMAIN FROM HUMAN TRF1	LTR	0.20
1M9I	CRYSTAL STRUCTURE OF PHOSPHORYLATION-MIMICKINGMUTANT T356D OF ANNEXIN VI	LTR	0.20
2VR2	HUMAN DIHYDROPYRIMIDINASE	FSR	0.20
1UPV	CRYSTAL STRUCTURE OF THE HUMAN LIVER X RECEPTOR BETA LIGAND BINDING DOMAIN IN COMPLEX WITH A SYNTHETIC AGONIST	FSR	0.20
2A91	CRYSTAL STRUCTURE OF ERBB2 DOMAINS 1-3	LTR	0.21
1ZIW	HUMAN TOLL-LIKE RECEPTOR 3 EXTRACELLULAR DOMAIN STRUCTURE	LTR	0.21
1D7P	CRYSTAL STRUCTURE OF THE C2 DOMAIN OF HUMAN FACTOR VIII AT 1.5 A RESOLUTION AT 1.5 A	LTR	0.21
1QX3	CONFORMATIONAL RESTRICTIONS IN THE ACTIVE SITE OF UNLIGANDED HUMAN CASPASE-3	LTR	0.22
1ZC0	CRYSTAL STRUCTURE OF HUMAN HEMATOPOIETIC TYROSINE PHOSPHATASE (HEPTP) CATALYTIC DOMAIN	LTR	0.23
2A4D	STRUCTURE OF THE HUMAN UBIQUITIN-CONJUGATING ENZYME E2 VARIANT 1 (UEV-1)	LTR	0.24
1LJ5	1.8A RESOLUTION STRUCTURE OF LATENT PLASMINOGEN ACTIVATOR INHIBITOR-1(PAI-1)	LTR	0.24
1UZE	COMPLEX OF THE ANTI-HYPERTENSIVE DRUG ENALAPRILAT AND THE HUMAN TESTICULAR ANGIOTENSIN I-CONVERTING ENZYME	FSR	0.24
2QIS	CRYSTAL STRUCTURE OF HUMAN FARNESYL PYROPHOSPHATE SYNTHASE T210S MUTANT BOUND TO RISEDRONATE	LTR	0.24
1CZA	MUTANT MONOMER OF RECOMBINANT HUMAN HEXOKINASE TYPE I COMPLEXED WITH GLUCOSE_ GLUCOSE-6-PHOSPHATE_ AND ADP	FSR	0.25
1QX3	CONFORMATIONAL RESTRICTIONS IN THE ACTIVE SITE OF UNLIGANDED HUMAN CASPASE-3	LTR	0.26
1LCT	STRUCTURE OF THE RECOMBINANT N-TERMINAL LOBE OF HUMAN LACTOFERRIN AT 2.0 ANGSTROMS RESOLUTION	FSR	0.26
1R03	CRYSTAL STRUCTURE OF A HUMAN MITOCHONDRIAL FERRITIN	FSR	0.27
2JBO	PROTEIN KINASE MK2 IN COMPLEX WITH AN INHIBITOR (CRYSTAL FORM-1_ SOAKING)	FSR	0.27

2A8B	CRYSTAL STRUCTURE OF THE CATALYTIC DOMAIN OF HUMAN TYROSINE PHOSPHATASE RECEPTOR_ TYPE R	LTR	0.28
1X9D	CRYSTAL STRUCTURE OF HUMAN CLASS I ALPHA-1_2-MANNOSIDASE IN COMPLEX WITH THIO-DISACCHARIDE SUBSTRATE ANALOGUE	FSR	0.28
1ND7	CONFORMATIONAL FLEXIBILITY UNDERLIES UBIQUITIN LIGATION MEDIATED BY THE WWP1 HECT DOMAIN E3 LIGASE	FSR	0.28
2JDF	HUMAN GAMMA-B CRYSTALLIN	FSR	0.29
1KHX	CRYSTAL STRUCTURE OF A PHOSPHORYLATED SMAD2	LTR	0.30
1WCH	CRYSTAL STRUCTURE OF PTPL1 HUMAN TYROSINE PHOSPHATASE MUTATED IN COLORECTAL CANCER- EVIDENCE FOR A SECOND PHOSPHOTYROSINE SUBSTRATE RECOGNITION POCKET	LTR	0.30
1W7L	CRYSTAL STRUCTURE OF HUMAN KYNURENINE AMINOTRANSFERASE I	FSR	0.30
2B6H	STRUCTURE OF HUMAN ADP-RIBOSYLATION FACTOR 5	FSR	0.30
2NNJ	CYP2C8DH COMPLEXED WITH FELODIPINE	LTR	0.31
2QTZ	CRYSTAL STRUCTURE OF THE NADP+-BOUND FAD-CONTAINING FNR- LIKE MODULE OF HUMAN METHIONINE SYNTHASE REDUCTASE	FSR	0.31
1MX3	CRYSTAL STRUCTURE OF CTBP DEHYDROGENASE CORE HOLO FORM	LTR	0.31
2FUE	HUMAN ALPHA-PHOSPHOMANNOMUTASE 1 WITH D-MANNOSE 1-PHOSPHATE AND MG2+ COFACTOR BOUND	FSR	0.31
2QTZ	CRYSTAL STRUCTURE OF THE NADP+-BOUND FAD-CONTAINING FNR- LIKE MODULE OF HUMAN METHIONINE SYNTHASE REDUCTASE	LTR	0.31
1CZA	MUTANT MONOMER OF RECOMBINANT HUMAN HEXOKINASE TYPE I COMPLEXED WITH GLUCOSE_ GLUCOSE-6-PHOSPHATE_ AND ADP	LTR	0.31
2F1W	CRYSTAL STRUCTURE OF THE TRAF-LIKE DOMAIN OF HAUSP/USP7	FSR	0.32
1QIB	CRYSTAL STRUCTURE OF GELATINASE A CATALYTIC DOMAIN	FSR	0.33
1CIZ	X-RAY STRUCTURE OF HUMAN STROMELYSIN CATALYTIC DOMAIN COMPLEXES WITH NON-PEPTIDE INHIBITORS: IMPLICATION FOR INHIBITOR SELECTIVITY	FSR	0.33
1LZJ	GLYCOSYLTRANSFERASE B + UDP + H ANTIGEN ACCEPTOR	LTR	0.34
2C9H	STRUCTURE OF MITOCHONDRIAL BETA-KETOACYL SYNTHASE	FSR	0.34
1TAZ	CATALYTIC DOMAIN OF HUMAN PHOSPHODIESTERASE 1B	LTR	0.34
1ZGK	1.35 ANGSTROM STRUCTURE OF THE KELCH DOMAIN OF KEAP1	LTR	0.35
1R55	CRYSTAL STRUCTURE OF THE CATALYTIC DOMAIN OF HUMAN ADAM 33	LTR	0.35
1ILK	INTERLEUKIN-10 CRYSTAL STRUCTURE REVEALS THE FUNCTIONAL DIMER WITH AN UNEXPECTED TOPOLOGICAL SIMILARITY TO INTERFERON GAMMA	FSR	0.35
2Q5I	CRYSTAL STRUCTURE OF APO S581L GLYCYL-TRNA SYNTHETASE MUTAN	LTR	0.36
1WRM	CRYSTAL STRUCTURE OF JSP-1	LTR	0.37
1IAT	CRYSTAL STRUCTURE OF HUMAN PHOSPHOGLUCOSE ISOMERASE/NEUROLEUKIN/AUTOCRINE MOTILITY FACTOR/MATURATION FACTOR	LTR	0.37
2NZL	CRYSTAL STRUCTURE OF HUMAN HYDROXYACID OXIDASE 1	FSR	0.37
3C8X	CRYSTAL STRUCTURE OF THE LIGAND BINDING DOMAIN OF HUMAN EPHRIN A2 (EPHA2) RECEPTOR PROTEIN KINASE	LTR	0.37
2F21	HUMAN PIN1 FIP MUTANT	FSR	0.37
2FCB	HUMAN FC GAMMA RECEPTOR IIB ECTODOMAIN (CD32)	FSR	0.39
1CZA	MUTANT MONOMER OF RECOMBINANT HUMAN HEXOKINASE TYPE I COMPLEXED WITH GLUCOSE_ GLUCOSE-6-PHOSPHATE_ AND ADP	LTR	0.39
2B5M	CRYSTAL STRUCTURE OF DDB1	LTR	0.40
2EC8	CRYSTAL STRUCTURE OF THE EXCTRACELLULAR DOMAIN OF THE RECEPTOR TYROSINE KINASE_ KIT	LTR	0.40
3CFW	L-SELECTIN LECTIN AND EGF DOMAINS	FSR	0.40
1Q92	CRYSTAL STRUCTURE OF HUMAN MITOCHONDRIAL DEOXYRIBONUCLEOTIDASE IN COMPLEX WITH THE INHIBITOR PMCP-U	LTR	0.41
2F1W	CRYSTAL STRUCTURE OF THE TRAF-LIKE DOMAIN OF HAUSP/USP7	FSR	0.41
1AD6	DOMAIN A OF HUMAN RETINOBLASTOMA TUMOR SUPPRESSOR	LTR	0.42
1MZA	CRYSTAL STRUCTURE OF HUMAN PRO-GRANZYME K	FSR	0.46
1E0S	SMALL G PROTEIN ARF6-GDP	LTR	0.46

2HWX	STRUCTURE OF HUMAN SMG6 E1282C PIN DOMAIN MUTANT.	LTR	0.47
1Z1L	THE CRYSTAL STRUCTURE OF THE PHOSPHODIESTERASE 2A CATALYTIC DOMAIN	FSR	0.48
2I4I	CRYSTAL STRUCTURE OF HUMAN DEAD-BOX RNA HELICASE DDX3X	LTR	0.51
2GRY	CRYSTAL STRUCTURE OF THE HUMAN KIF2 MOTOR DOMAIN IN COMPLEX WITH ADP	LTR	0.56
1A7S	ATOMIC RESOLUTION STRUCTURE OF HBP	LTR	0.59
1CIZ	X-RAY STRUCTURE OF HUMAN STROMELYSIN CATALYTIC DOMAIN COMPLEXES WITH NON-PEPTIDE INHIBITORS: IMPLICATION FOR INHIBITOR SELECTIVITY	LTR	0.61
2NSQ	CRYSTAL STRUCTURE OF THE C2 DOMAIN OF THE HUMAN E3 UBIQUITIN PROTEIN LIGASE NEDD4-LIKE PROTEIN	LTR	0.74
2JC9	CRYSTAL STRUCTURE OF HUMAN CYTOSOLIC 5'-NUCLEOTIDASE II IN COMPLEX WITH ADENOSINE	LTR	2.00
	**thrombin**		
2QQJ	CRYSTAL STRUCTURE OF THE B1B2 DOMAINS FROM HUMAN NEUROPILIN 2	FVR	0.12
2NR8	CRYSTAL STRUCTURE OF THE HUMAN KIF9 MOTOR DOMAIN IN COMPLEX WITH ADP	FVR	0.12
2B9E	HUMAN NSUN5 PROTEIN	FVR	0.12
2QQI	CRYSTAL STRUCTURE OF THE B1B2 DOMAINS FROM HUMAN NEUROPILIN 1	FVR	0.12
1SI5	PROTEASE-LIKE DOMAIN FROM 2-CHAIN HEPATOCYTE GROWTH FACTOR	FVR	0.12
2EC8	CRYSTAL STRUCTURE OF THE EXCTRACELLULAR DOMAIN OF THE RECEPTOR TYROSINE KINASE_ KIT	FVR	0.13
2QQJ	CRYSTAL STRUCTURE OF THE B1B2 DOMAINS FROM HUMAN NEUROPILIN 2	FVR	0.13
1A6Q	CRYSTAL STRUCTURE OF THE PROTEIN SERINE/THREONINE PHOSPHATASE 2C AT 2 A RESOLUTION	FVR	0.16
1MD8	MONOMERIC STRUCTURE OF THE ACTIVE CATALYTIC DOMAIN OF COMPLEMENT PROTEASE C1R	FVR	0.17
1NUF	ROLE OF CALCIUM IONS IN THE ACTIVATION AND ACTIVITY OF THE TRANSGLUTAMINASE 3 ENZYME	FVR	0.17
2BH9	X-RAY STRUCTURE OF A DELETION VARIANT OF HUMAN GLUCOSE 6-PHOSPHATE DEHYDROGENASE COMPLEXED WITH STRUCTURAL AND COENZYME NADP	FVR	0.17
2FY2	STRUCTURES OF LIGAND BOUND HUMAN CHOLINE ACETYLTRANSFERASE PROVIDE INSIGHT INTO REGULATION OF ACETYLCHOLINE SYNTHESIS	FVR	0.18
2PZ1	CRYSTAL STRUCTURE OF AUTO-INHIBITED ASEF	FVR	0.18
2F1W	CRYSTAL STRUCTURE OF THE TRAF-LIKE DOMAIN OF HAUSP/USP7	FVR	0.18
2A2K	CRYSTAL STRUCTURE OF AN ACTIVE SITE MUTANT_ C473S_ OF CDC25B PHOSPHATASE CATALYTIC DOMAIN	GPR	0.19
2A4D	STRUCTURE OF THE HUMAN UBIQUITIN-CONJUGATING ENZYME E2 VARIANT 1 (UEV-1)	FVR	0.19
1F5N	HUMAN GUANYLATE BINDING PROTEIN-1 IN COMPLEX WITH THE GTP ANALOGUE_ GMPPNP.	GPR	0.19
2VGE	CRYSTAL STRUCTURE OF THE C-TERMINAL REGION OF HUMAN IASPP	VPR	0.20
1TXU	CRYSTAL STRUCTURE OF THE VPS9 DOMAIN OF RABEX-5	VPR	0.20
1ZGK	1.35 ANGSTROM STRUCTURE OF THE KELCH DOMAIN OF KEAP1	VPR	0.20
1C25	HUMAN CDC25A CATALYTIC DOMAIN	GPR	0.21
2HC1	ENGINEERED CATALYTIC DOMAIN OF PROTEIN TYROSINE PHOSPHATASE HPTPBETA.	FVR	0.21
1CZA	MUTANT MONOMER OF RECOMBINANT HUMAN HEXOKINASE TYPE I COMPLEXED WITH GLUCOSE_ GLUCOSE-6-PHOSPHATE_ AND ADP	FVR	0.22
1XJD	CRYSTAL STRUCTURE OF PKC-THETA COMPLEXED WITH STAUROSPORINE AT 2A RESOLUTION	FVR	0.22
1HFC	1.56 ANGSTROM STRUCTURE OF MATURE TRUNCATED HUMAN FIBROBLAST COLLAGENASE	FVR	0.22
3C5H	CRYSTAL STRUCTURE OF THE RAS HOMOLOG DOMAIN OF HUMAN GRLF1 (P190RHOGAP)	FVR	0.22

2BKA	CC3(TIP30)CRYSTAL STRUCURE	FVR	0.22
2PBN	CRYSTAL STRUCTURE OF THE HUMAN TYROSINE RECEPTOR PHOSPHATE GAMMA	FVR	0.22
1Z8D	CRYSTAL STRUCTURE OF HUMAN MUSCLE GLYCOGEN PHOSPHORYLASE A WITH AMP AND GLUCOSE	VPR	0.22
1N3Y	CRYSTAL STRUCTURE OF THE ALPHA-X BETA2 INTEGRIN I DOMAIN	FVR	0.22
2II0	CRYSTAL STRUCTURE OF CATALYTIC DOMAIN OF SON OF SEVENLESS (REM-CDC25) IN THE ABSENCE OF RAS	FVR	0.23
1FA9	HUMAN LIVER GLYCOGEN PHOSPHORYLASE A COMPLEXED WITH AMP	VPR	0.23
1ZGK	1.35 ANGSTROM STRUCTURE OF THE KELCH DOMAIN OF KEAP1	VPR	0.23
2NZ2	CRYSTAL STRUCTURE OF HUMAN ARGININOSUCCINATE SYNTHASE IN COMPLEX WITH ASPARTATE AND CITRULLINE	FVR	0.24
1CZA	MUTANT MONOMER OF RECOMBINANT HUMAN HEXOKINASE TYPE I COMPLEXED WITH GLUCOSE_ GLUCOSE-6-PHOSPHATE_ AND ADP	FVR	0.24
2QTZ	CRYSTAL STRUCTURE OF THE NADP+-BOUND FAD-CONTAINING FNR- LIKE MODULE OF HUMAN METHIONINE SYNTHASE REDUCTASE	FVR	0.24
1ELV	CRYSTAL STRUCTURE OF THE CATALYTIC DOMAIN OF HUMAN COMPLEMENT C1S PROTEASE	VPR	0.24
1IAT	CRYSTAL STRUCTURE OF HUMAN PHOSPHOGLUCOSE ISOMERASE/NEUROLEUKIN/AUTOCRINE MOTILITY FACTOR/MATURATION FACTOR	GPR	0.27
2OCG	CRYSTAL STRUCTURE OF HUMAN VALACYCLOVIR HYDROLASE	VPR	0.27
1KT0	STRUCTURE OF THE LARGE FKBP-LIKE PROTEIN_ FKBP51_ INVOLVED IN STEROID RECEPTOR COMPLEXES	GPR	0.28
2C9H	STRUCTURE OF MITOCHONDRIAL BETA-KETOACYL SYNTHASE	VPR	0.28
2B69	CRYSTAL STRUCTURE OF HUMAN UDP-GLUCORONIC ACID DECARBOXYLAS	GPR	0.28
1B0F	CRYSTAL STRUCTURE OF HUMAN NEUTROPHIL ELASTASE WITH MDL 101 146	FVR	0.29
2JHM	STRUCTURE OF GLOBULAR HEADS OF M-FICOLIN AT NEUTRAL PH	GPR	0.29
1IJB	THE VON WILLEBRAND FACTOR MUTANT (I546V) A1 DOMAIN	FVR	0.30
1YCK	CRYSTAL STRUCTURE OF HUMAN PEPTIDOGLYCAN RECOGNITION PROTEIN (PGRP-S)	VPR	0.30
2Z6O	CRYSTAL STRUCTURE OF THE UFC1_ UFM1 CONJUGATING ENZYME 1	GPR	0.30
1KO9	NATIVE STRUCTURE OF THE HUMAN 8-OXOGUANINE DNA GLYCOSYLASE HOGG1	GPR	0.30
1UCH	DEUBIQUITINATING ENZYME UCH-L3 (HUMAN) AT 1.8 ANGSTROM RESOLUTION	VPR	0.31
2AA2	MINERALOCORTICOID RECEPTOR WITH BOUND ALDOSTERONE	FVR	0.31
2OBV	CRYSTAL STRUCTURE OF THE HUMAN S-ADENOSYLMETHIONINE SYNTHETASE 1 IN COMPLEX WITH THE PRODUCT	VPR	0.32
1TDH	CRYSTAL STRUCTURE OF HUMAN ENDONUCLEASE VIII-LIKE 1 (NEIL1)	VPR	0.33
2ALR	ALDEHYDE REDUCTASE	VPR	0.34
1TQN	CRYSTAL STRUCTURE OF HUMAN MICROSOMAL P450 3A4	GPR	0.34
1ZVD	REGULATION OF SMURF2 UBIQUITIN LIGASE ACTIVITY BY ANCHORING THE E2 TO THE HECT DOMAIN	GPR	0.34
2HI4	CRYSTAL STRUCTURE OF HUMAN MICROSOMAL P450 1A2 IN COMPLEX WITH ALPHA-NAPHTHOFLAVONE	GPR	0.35
2ALR	ALDEHYDE REDUCTASE	VPR	0.37
2GY5	TIE2 LIGAND-BINDING DOMAIN CRYSTAL STRUCTURE	VPR	0.38
1D2S	CRYSTAL STRUCTURE OF THE N-TERMINAL LAMININ G-LIKE DOMAIN OF SHBG IN COMPLEX WITH DIHYDROTESTOSTERONE	GPR	0.38
1BYG	KINASE DOMAIN OF HUMAN C-TERMINAL SRC KINASE (CSK) IN COMPLEX WITH INHIBITOR STAUROSPORINE	VPR	0.38
2FN4	THE CRYSTAL STRUCTURE OF HUMAN RAS-RELATED PROTEIN_ RRAS_ IN THE GDP-BOUND STATE	VPR	0.38
1QPC	STRUCTURAL ANALYSIS OF THE LYMPHOCYTE-SPECIFIC KINASE LCK IN COMPLEX WITH NON-SELECTIVE AND SRC FAMILY SELECTIVE KINASE INHIBITORS	VPR	0.40

2B69	CRYSTAL STRUCTURE OF HUMAN UDP-GLUCORONIC ACID DECARBOXYLAS	GPR	0.40
1N26	CRYSTAL STRUCTURE OF THE EXTRA-CELLULAR DOMAINS OF HUMAN INTERLEUKIN-6 RECEPTOR ALPHA CHAIN	GPR	0.41
1WAK	X-RAY STRUCTURE OF SRPK1	VPR	0.41
2J0I	CRYSTAL STRUCTURE OF THE HUMAN P21-ACTIVATED KINASE 4	VPR	0.42
2O36	CRYSTAL STRUCTURE OF ENGINEERED THIMET OLIGOPEPTIDASE WITH NEUROLYSIN SPECIFICITY IN NEUROTENSIN CLEAVAGE SITE	VPR	0.43
2BH9	X-RAY STRUCTURE OF A DELETION VARIANT OF HUMAN GLUCOSE 6-PHOSPHATE DEHYDROGENASE COMPLEXED WITH STRUCTURAL AND COENZYME NADP	VPR	0.43
1S31	CRYSTAL STRUCTURE ANALYSIS OF THE HUMAN TUB PROTEIN (ISOFORM A) SPANNING RESIDUES 289 THROUGH 561	GPR	0.44
1HDO	HUMAN BILIVERDIN IX BETA REDUCTASE: NADP COMPLEX	GPR	0.45
1LZJ	GLYCOSYLTRANSFERASE B + UDP + H ANTIGEN ACCEPTOR	VPR	0.46
2A4D	STRUCTURE OF THE HUMAN UBIQUITIN-CONJUGATING ENZYME E2 VARIANT 1 (UEV-1)	VPR	0.46
1W0H	CRYSTALLOGRAPHIC STRUCTURE OF THE NUCLEASE DOMAIN OF 3'HEXO A DEDDH FAMILY MEMBER_ BOUND TO RAMP	VPR	0.48
3CHO	CRYSTAL STRUCTURE OF LEUKOTRIENE A4 HYDROLASE IN COMPLEX WITH 2-AMINO-N-[4-(PHENYLMETHOXY)PHENYL]-ACETAMIDE	GPR	0.48
1CB0	STRUCTURE OF HUMAN 5'-DEOXY-5'-METHYLTHIOADENOSINE PHOSPHORYLASE AT 1.7 A RESOLUTION	GPR	0.50
1S1P	CRYSTAL STRUCTURES OF PROSTAGLANDIN D2 11-KETOREDUCTASE (AKR1C3) IN COMPLEX WITH THE NON-STEROIDAL ANTI-INFLAMMATORY DRUGS FLUFENAMIC ACID AND INDOMETHACIN	VPR	0.51
2QYM	CRYSTAL STRUCTURE OF UNLIGANDED PDE4C2	VPR	0.51
1Q33	CRYSTAL STRUCTURE OF HUMAN ADP-RIBOSE PYROPHOSPHATASE NUDT9	GPR	0.53
1YO8	STRUCUTURE OF THE C-TERMINAL DOMAIN OF HUMAN THROMBOSPONDIN 2	VPR	0.54
1OZ2	CRYSTAL STRUCTURE OF 3-MBT REPEATS OF LETHAL (3) MALIGNANT BRAIN TUMOR (NATIVE-II) AT 1.55 ANGSTROM	GPR	0.54
1FNH	CRYSTAL STRUCTURE OF HEPARIN AND INTEGRIN BINDING SEGMENT OF HUMAN FIBRONECTIN	VPR	0.56
1TDH	CRYSTAL STRUCTURE OF HUMAN ENDONUCLEASE VIII-LIKE 1 (NEIL1)	GPR	0.56
1UZE	COMPLEX OF THE ANTI-HYPERTENSIVE DRUG ENALAPRILAT AND THE HUMAN TESTICULAR ANGIOTENSIN I-CONVERTING ENZYME	VPR	0.57
2AWF	STRUCTURE OF HUMAN UBIQUITIN-CONJUGATING ENZYME E2 G1	VPR	0.57
2GF9	CRYSTAL STRUCTURE OF HUMAN RAB3D IN COMPLEX WITH GDP	VPR	0.58
1PME	STRUCTURE OF PENTA MUTANT HUMAN ERK2 MAP KINASE COMPLEXED WITH A SPECIFIC INHIBITOR OF HUMAN P38 MAP KINASE	GPR	0.60
1PI1	CRYSTAL STRUCTURE OF A HUMAN MOB1 PROTEIN; TOWARD UNDERSTANDING MOB-REGULATED CELL CYCLE PATHWAYS.	GPR	0.61
1VZO	THE STRUCTURE OF THE N-TERMINAL KINASE DOMAIN OF MSK1 REVEALS A NOVEL AUTOINHIBITORY CONFORMATION FOR A DUAL KINASE PROTEIN	GPR	0.62
2A5J	CRYSTAL STRUCTURE OF HUMAN RAB2B	VPR	0.66
3COU	CRYSTAL STRUCTURE OF HUMAN NUDIX MOTIF 16 (NUDT16)	GPR	0.73
1CZA	MUTANT MONOMER OF RECOMBINANT HUMAN HEXOKINASE TYPE I COMPLEXED WITH GLUCOSE_ GLUCOSE-6-PHOSPHATE_ AND ADP	GPR	0.74
2QT1	HUMAN NICOTINAMIDE RIBOSIDE KINASE 1 IN COMPLEX WITH NICOTINAMIDE RIBOSIDE	VPR	0.75
2PNY	STRUCTURE OF HUMAN ISOPENTENYL-DIPHOSPHATE DELTA-ISOMERASE	VPR	0.76
1YRV	NOVEL UBIQUITIN-CONJUGATING ENZYME	VPR	0.92
2HQ6	STRUCTURE OF THE CYCLOPHILIN_CECYP16-LIKE DOMAIN OF THE SEROLOGICALLY DEFINED COLON CANCER ANTIGEN 10 FROM HOMO SAPIENS	VPR	1.01
1LBD	LIGAND-BINDING DOMAIN OF THE HUMAN NUCLEAR RECEPTOR RXR-ALPHA	VPR	2.00

1MX3	CRYSTAL STRUCTURE OF CTBP DEHYDROGENASE CORE HOLO FORM	VPR	2.00
1P5J	CRYSTAL STRUCTURE ANALYSIS OF HUMAN SERINE DEHYDRATASE	VPR	2.00
1PME	STRUCTURE OF PENTA MUTANT HUMAN ERK2 MAP KINASE COMPLEXED WITH A SPECIFIC INHIBITOR OF HUMAN P38 MAP KINASE	VPR	2.00
1Q1C	CRYSTAL STRUCTURE OF N(1-260) OF HUMAN FKBP52	VPR	2.00
1TBF	CATALYTIC DOMAIN OF HUMAN PHOSPHODIESTERASE 5A IN COMPLEX WITH SILDENAFIL	VPR	2.00
1XAP	STRUCTURE OF THE LIGAND BINDING DOMAIN OF THE RETINOIC ACID RECEPTOR BETA	VPR	2.00
1ZD9	STRUCTURE OF HUMAN ADP-RIBOSYLATION FACTOR-LIKE 10B	VPR	2.00
1ZIV	CATALYTIC DOMAIN OF HUMAN CALPAIN-9	VPR	2.00
1ZJH	STRUCTURE OF HUMAN MUSCLE PYRUVATE KINASE (PKM2)	VPR	2.00
2B3H	CRYSTAL STRUCTURE OF HUMAN METHIONINE AMINOPEPTIDASE TYPE I WITH A THIRD COBALT IN THE ACTIVE SITE	VPR	2.00
2B6H	STRUCTURE OF HUMAN ADP-RIBOSYLATION FACTOR 5	VPR	2.00
2B9E	HUMAN NSUN5 PROTEIN	VPR	2.00
2EW1	CRYSTAL STRUCTURE OF RAB30 IN COMPLEX WITH A GTP ANALOGUE	VPR	2.00
2FAU	CRYSTAL STRUCTURE OF HUMAN VPS26	VPR	2.00
2FG5	CRYSTAL STRUCTURE OF HUMAN RAB31 IN COMPLEX WITH A GTP ANALOGUE	VPR	2.00
2FK9	HUMAN PROTEIN KINASE C_ ETA	VPR	2.00
2FOL	CRYSTAL STRUCTURE OF HUMAN RAB1A IN COMPLEX WITH GDP	VPR	2.00
2FYT	HUMAN HMT1 HNRNP METHYLTRANSFERASE-LIKE 3 (S. CEREVISIAE) PROTEIN	VPR	2.00
2GEE	CRYSTAL STRUCTURE OF HUMAN TYPE III FIBRONECTIN EXTRADOMAIN B AND DOMAIN 8	VPR	2.00
2GFO	STRUCTURE OF THE CATALYTIC DOMAIN OF HUMAN UBIQUITIN CARBOXYL-TERMINAL HYDROLASE 8	VPR	2.00
2GRY	CRYSTAL STRUCTURE OF THE HUMAN KIF2 MOTOR DOMAIN IN COMPLEX WITH ADP	VPR	2.00
2GW2	CRYSTAL STRUCTURE OF THE PEPTIDYL-PROLYL ISOMERASE DOMAIN OF HUMAN CYCLOPHILIN G	VPR	2.00
2H17	STRUCTURE OF HUMAN ADP-RIBOSYLATION FACTOR-LIKE 5 (ARL5) (CASP TARGET)	VPR	2.00
2HEH	CRYSTAL STRUCTURE OF THE KIF2C MOTOR DOMAIN (CASP TARGET)	VPR	2.00
2HZ6	THE CRYSTAL STRUCTURE OF HUMAN IRE1-ALPHA LUMINAL DOMAIN	VPR	2.00
2I7A	DOMAIN IV OF HUMAN CALPAIN 13	VPR	2.00
2I7Q	CRYSTAL STRUCTURE OF HUMAN CHOLINE KINASE A	VPR	2.00
2IL1	CRYSTAL STRUCTURE OF A PREDICTED HUMAN GTPASE IN COMPLEX WITH GDP	VPR	2.00
2IUW	CRYSTAL STRUCTURE OF HUMAN ABH3 IN COMPLEX WITH IRON ION AND 2-OXOGLUTARATE	VPR	2.00
2JC9	CRYSTAL STRUCTURE OF HUMAN CYTOSOLIC 5'-NUCLEOTIDASE II IN COMPLEX WITH ADENOSINE	VPR	2.00
2NR8	CRYSTAL STRUCTURE OF THE HUMAN KIF9 MOTOR DOMAIN IN COMPLEX WITH ADP	VPR	2.00
2OIL	CRYSTAL STRUCTURE OF HUMAN RAB25 IN COMPLEX WITH GDP	VPR	2.00
2QLU	CRYSTAL STRUCTURE OF ACTIVIN RECEPTOR TYPE II KINASE DOMAIN FROM HUMAN	VPR	2.00
2REP	CRYSTAL STRUCTURE OF THE MOTOR DOMAIN OF HUMAN KINESIN FAMILY MEMBER C1	VPR	2.00
2VKQ	CRYSTAL STRUCTURE OF HUMAN CYTOSOLIC 5'-NUCLEOTIDASE III (CN-III_ NT5C3) IN COMPLEX WITH BERYLLIUM TRIFLUORIDE	VPR	2.00
2VPJ	CRYSTAL STRUCTURE OF THE KELCH DOMAIN OF HUMAN KLHL12	GPR	2.00
2ZMD	CRYSTAL STRUCTURE OF HUMAN MPS1 CATALYTIC DOMAIN T686A MUTANT IN COMPLEX WITH SP600125 INHIBITOR	VPR	2.00
3BCH	CRYSTAL STRUCTURE OF THE HUMAN LAMININ RECEPTOR PRECURSOR	VPR	2.00
3BD9	HUMAN 3-O-SULFOTRANSFERASE ISOFORM 5 WITH BOUND PAP	VPR	2.00

	**t-plasminogen activator**		
2NSM	CRYSTAL STRUCTURE OF THE HUMAN CARBOXYPEPTIDASE N (KININASE I) CATALYTIC DOMAIN	LGR	0.10
1W6K	STRUCTURE OF HUMAN OSC IN COMPLEX WITH LANOSTEROL	LGR	0.10
2O8T	CRYSTAL STRUCTURE AND BINDING EPITOPES OF UROKINASE-TYPE PLASMINOGEN ACTIVATOR (C122A/N145Q) IN COMPLEX WITH INHIBITORS	LGR	0.11
1SPJ	STRUCTURE OF MATURE HUMAN TISSUE KALLIKREIN (HUMAN KALLIKREIN 1 OR KLK1) AT 1.70 ANGSTROM RESOLUTION WITH VACANT ACTIVE SITE	LGR	0.12
2DW5	CRYSTAL STRUCTURE OF HUMAN PEPTIDYLARGININE DEIMINASE 4 IN COMPLEX WITH N-ALPHA-BENZOYL-N5-(2-FLUORO-1-IMINOETHYL)-L-ORNITHINE AMIDE	LGR	0.12
1OHC	STRUCTURE OF THE PROLINE DIRECTED PHOSPHATASE CDC14	LGR	0.13
2PCX	CRYSTAL STRUCTURE OF P53DBD(R282Q) AT 1.54-ANGSTROM RESOLUTION	LGR	0.15
1Q33	CRYSTAL STRUCTURE OF HUMAN ADP-RIBOSE PYROPHOSPHATASE NUDT9	LGR	0.16
1KHB	PEPCK COMPLEX WITH NONHYDROLYZABLE GTP ANALOG_ NATIVE DATA	LGR	0.16
1UOU	CRYSTAL STRUCTURE OF HUMAN THYMIDINE PHOSPHORYLASE IN COMPLEX WITH A SMALL MOLECULE INHIBITOR	LGR	0.16
1Z8D	CRYSTAL STRUCTURE OF HUMAN MUSCLE GLYCOGEN PHOSPHORYLASE A WITH AMP AND GLUCOSE	LGR	0.17
1NM8	STRUCTURE OF HUMAN CARNITINE ACETYLTRANSFERASE: MOLECULAR BASIS FOR FATTY ACYL TRANSFER	LGR	0.18
1M6I	CRYSTAL STRUCTURE OF APOPTOSIS INDUCING FACTOR (AIF)	LGR	0.19
2Q5I	CRYSTAL STRUCTURE OF APO S581L GLYCYL-TRNA SYNTHETASE MUTAN	LGR	0.19
2CY7	THE CRYSTAL STRUCTURE OF HUMAN ATG4B	LGR	0.19
1UOU	CRYSTAL STRUCTURE OF HUMAN THYMIDINE PHOSPHORYLASE IN COMPLEX WITH A SMALL MOLECULE INHIBITOR	LGR	0.19
1OZN	1.5A CRYSTAL STRUCTURE OF THE NOGO RECEPTOR LIGAND BINDING DOMAIN REVEALS A CONVERGENT RECOGNITION SCAFFOLD MEDIATING INHIBITION OF MYELINATION	LGR	0.19
1FA9	HUMAN LIVER GLYCOGEN PHOSPHORYLASE A COMPLEXED WITH AMP	LGR	0.19
1LF7	CRYSTAL STRUCTURE OF HUMAN COMPLEMENT PROTEIN C8GAMMA AT 1.2 A RESOLUTION	LGR	0.20
1RYO	HUMAN SERUM TRANSFERRIN_ N-LOBE BOUND WITH OXALATE	LGR	0.20
2PE4	STRUCTURE OF HUMAN HYALURONIDASE 1_ A HYALURONAN HYDROLYZING ENZYME INVOLVED IN TUMOR GROWTH AND ANGIOGENESIS	LGR	0.21
2I7V	STRUCTURE OF HUMAN CPSF-73	LGR	0.22
1OZN	1.5A CRYSTAL STRUCTURE OF THE NOGO RECEPTOR LIGAND BINDING DOMAIN REVEALS A CONVERGENT RECOGNITION SCAFFOLD MEDIATING INHIBITION OF MYELINATION	LGR	0.22
2V24	STRUCTURE OF THE HUMAN SPRY DOMAIN-CONTAINING SOCS BOX PROTEIN SSB-4	LGR	0.22
1SIQ	THE CRYSTAL STRUCTURE AND MECHANISM OF HUMAN GLUTARYL-COA DEHYDROGENASE	LGR	0.23
2HQ6	STRUCTURE OF THE CYCLOPHILIN_CECYP16-LIKE DOMAIN OF THE SEROLOGICALLY DEFINED COLON CANCER ANTIGEN 10 FROM HOMO SAPIENS	LGR	0.23
2HZ6	THE CRYSTAL STRUCTURE OF HUMAN IRE1-ALPHA LUMINAL DOMAIN	LGR	0.24
1XWI	CRYSTAL STRUCTURE OF VPS4B	LGR	0.24
1GS9	APOLIPOPROTEIN E4_ 22K DOMAIN	LGR	0.25
2CL3	CRYSTAL STRUCTURE OF HUMAN CLEAVAGE AND POLYADENYLATION SPECIFICITY FACTOR 5 (CPSF5)	LGR	0.26
2ILR	CRYSTAL STRUCTURE OF HUMAN FANCONI ANEMIA PROTEIN E C-TERMINAL DOMAIN	LGR	0.26

1LCY	CRYSTAL STRUCTURE OF THE MITOCHONDRIAL SERINE PROTEASE HTRA	LGR	0.27
2O36	CRYSTAL STRUCTURE OF ENGINEERED THIMET OLIGOPEPTIDASE WITH NEUROLYSIN SPECIFICITY IN NEUROTENSIN CLEAVAGE SITE	LGR	0.27
1W8M	ENZYMATIC AND STRUCTURAL CHARACTERISATION OF NON PEPTIDE LIGAND CYCLOPHILIN COMPLEXES	LGR	0.27
1SQW	CRYSTAL STRUCTURE OF KD93_ A NOVEL PROTEIN EXPRESSED IN THE HUMAN PRO	LGR	0.27
1MF7	INTEGRIN ALPHA M I DOMAIN	LGR	0.28
1SIQ	THE CRYSTAL STRUCTURE AND MECHANISM OF HUMAN GLUTARYL-COA DEHYDROGENASE	LGR	0.28
2BIT	CRYSTAL STRUCTURE OF HUMAN CYCLOPHILIN D AT 1.7 A RESOLUTIO	LGR	0.28
1ORE	HUMAN ADENINE PHOSPHORIBOSYLTRANSFERASE	LGR	0.28
1B0F	CRYSTAL STRUCTURE OF HUMAN NEUTROPHIL ELASTASE WITH MDL 101 146	LGR	0.28
2OPW	CRYSTAL STRUCTURE OF HUMAN PHYTANOYL-COA DIOXYGENASE PHYHD1 (APO)	LGR	0.28
1TA0	THREE-DIMENSIONAL STRUCTURE OF A RNA-POLYMERASE II BINDING PROTEIN WITH ASSOCIATED LIGAND.	LGR	0.28
1ZD3	HUMAN SOLUBLE EPOXIDE HYDROLASE 4-(3-CYCLOHEXYLURIEDO)-BUTYRIC ACID COMPLEX	LGR	0.29
1H30	C-TERMINAL LG DOMAIN PAIR OF HUMAN GAS6	LGR	0.29
1S35	CRYSTAL STRUCTURE OF REPEATS 8 AND 9 OF HUMAN ERYTHROID SPECTRIN	LGR	0.31
1MX3	CRYSTAL STRUCTURE OF CTBP DEHYDROGENASE CORE HOLO FORM	LGR	0.31
2CY7	THE CRYSTAL STRUCTURE OF HUMAN ATG4B	LGR	0.32
1W6K	STRUCTURE OF HUMAN OSC IN COMPLEX WITH LANOSTEROL	LGR	0.33
2GRY	CRYSTAL STRUCTURE OF THE HUMAN KIF2 MOTOR DOMAIN IN COMPLEX WITH ADP	LGR	0.34
1LI4	HUMAN S-ADENOSYLHOMOCYSTEINE HYDROLASE COMPLEXED WITH NEPLANOCIN	LGR	0.34
1OSH	A CHEMICAL_ GENETIC_ AND STRUCTURAL ANALYSIS OF THE NUCLEAR BILE ACID RECEPTOR FXR	LGR	0.35
2OYC	CRYSTAL STRUCTURE OF HUMAN PYRIDOXAL PHOSPHATE PHOSPHATASE	LGR	0.36
1ZC0	CRYSTAL STRUCTURE OF HUMAN HEMATOPOIETIC TYROSINE PHOSPHATASE (HEPTP) CATALYTIC DOMAIN	LGR	0.36
1DG6	CRYSTAL STRUCTURE OF APO2L/TRAIL	LGR	0.37
2NZ2	CRYSTAL STRUCTURE OF HUMAN ARGININOSUCCINATE SYNTHASE IN COMPLEX WITH ASPARTATE AND CITRULLINE	LGR	0.37
2OZU	CRYSTAL STRUCTURE OF HUMAN MYST HISTONE ACETYLTRANSFERASE 3 IN COMPLEX WITH ACETYLCOENZYME A	LGR	0.37
2IUW	CRYSTAL STRUCTURE OF HUMAN ABH3 IN COMPLEX WITH IRON ION AND 2-OXOGLUTARATE	LGR	0.37
2FVV	HUMAN DIPHOSPHOINOSITOL POLYPHOSPHATE PHOSPHOHYDROLASE 1	LGR	0.40
2O36	CRYSTAL STRUCTURE OF ENGINEERED THIMET OLIGOPEPTIDASE WITH NEUROLYSIN SPECIFICITY IN NEUROTENSIN CLEAVAGE SITE	LGR	0.40
1Q20	CRYSTAL STRUCTURE OF HUMAN CHOLESTEROL SULFOTRANSFERASE (SULT2B1B) IN THE PRESENCE OF PAP AND PREGNENOLONE	LGR	0.41
1EVS	CRYSTAL STRUCTURE OF HUMAN ONCOSTATIN M	LGR	0.41
1M6I	CRYSTAL STRUCTURE OF APOPTOSIS INDUCING FACTOR (AIF)	LGR	0.42
1UPV	CRYSTAL STRUCTURE OF THE HUMAN LIVER X RECEPTOR BETA LIGAND BINDING DOMAIN IN COMPLEX WITH A SYNTHETIC AGONIST	LGR	0.42
1L6J	CRYSTAL STRUCTURE OF HUMAN MATRIX METALLOPROTEINASE MMP9 (GELATINASE B).	LGR	0.43
1P0I	CRYSTAL STRUCTURE OF HUMAN BUTYRYL CHOLINESTERASE	LGR	0.43
1OHC	STRUCTURE OF THE PROLINE DIRECTED PHOSPHATASE CDC14	LGR	0.43
3BQC	HIGH PH-VALUE CRYSTAL STRUCTURE OF EMODIN IN COMPLEX WITH THE CATALYTIC SUBUNIT OF PROTEIN KINASE CK2	LGR	0.43

2OU2	ACETYLTRANSFERASE DOMAIN OF HUMAN HIV-1 TAT INTERACTING PROTEIN_ 60KDA_ ISOFORM 3	LGR	0.43
1MP8	CRYSTAL STRUCTURE OF FOCAL ADHESION KINASE (FAK)	LGR	0.43
1YWN	VEGFR2 IN COMPLEX WITH A NOVEL 4-AMINO-FURO[2_3-D]PYRIMIDIN	LGR	0.44
3BQC	HIGH PH-VALUE CRYSTAL STRUCTURE OF EMODIN IN COMPLEX WITH THE CATALYTIC SUBUNIT OF PROTEIN KINASE CK2	LGR	0.46
2V9K	CRYSTAL STRUCTURE OF HUMAN PUS10_ A NOVEL PSEUDOURIDINE SYNTHASE.	LGR	0.47
1Y6B	CRYSTAL STRUCTURE OF VEGFR2 IN COMPLEX WITH A 2-ANILINO-5- ARYL-OXAZOLE INHIBITOR	LGR	0.48
2P39	CRYSTAL STRUCTURE OF HUMAN FGF23	LGR	0.48
2DE0	CRYSTAL STRUCTURE OF HUMAN ALPHA 1_6-FUCOSYLTRANSFERASE_ FUT8	LGR	0.48
2A2C	X-RAY STRUCTURE OF HUMAN N-ACETYL GALACTOSAMINE KINASE COMPLEXED WITH MG-ADP AND N-ACETYL GALACTOSAMINE 1-PHOSPHATE	LGR	0.50
2UUI	CRYSTAL STRUCTURE OF HUMAN LEUKOTRIENE C4 SYNTHASE	LGR	0.50
1ZD3	HUMAN SOLUBLE EPOXIDE HYDROLASE 4-(3-CYCLOHEXYLURIEDO)-BUTYRIC ACID COMPLEX	LGR	0.53
1LCY	CRYSTAL STRUCTURE OF THE MITOCHONDRIAL SERINE PROTEASE HTRA	LGR	0.63
1JDW	CRYSTAL STRUCTURE AND MECHANISM OF L-ARGININE: GLYCINE AMIDINOTRANSFERASE: A MITOCHONDRIAL ENZYME INVOLVED IN CREATINE BIOSYNTHESIS	LGR	2.00
2AEX	THE 1.58A CRYSTAL STRUCTURE OF HUMAN COPROPORPHYRINOGEN OXIDASE REVEALS THE STRUCTURAL BASIS OF HEREDITARY COPROPORPHYRIA	LGR	2.00
2QOL	HUMAN EPHA3 KINASE AND JUXTAMEMBRANE REGION_ Y596:Y602:S768G TRIPLE MUTANT	LGR	2.00
	**tripeptidyl-peptidase** I		
1GEN	C-TERMINAL DOMAIN OF GELATINASE A	AAF	0.06
2UXW	CRYSTAL STRUCTURE OF HUMAN VERY LONG CHAIN ACYL-COA DEHYDROGENASE (ACADVL)	AAF	0.07
2B3X	STRUCTURE OF AN ORTHORHOMBIC CRYSTAL FORM OF HUMAN CYTOSOLIC ACONITASE (IRP1)	AAF	0.07
1M6I	CRYSTAL STRUCTURE OF APOPTOSIS INDUCING FACTOR (AIF)	AAF	0.07
1P49	STRUCTURE OF HUMAN PLACENTAL ESTRONE/DHEA SULFATASE	AAF	0.07
1T32	A DUAL INHIBITOR OF THE LEUKOCYTE PROTEASES CATHEPSIN G AND CHYMASE WITH THERAPEUTIC EFFICACY IN ANIMALS MODELS OF INFLAMMATION	AAF	0.08
2J5W	CERULOPLASMIN REVISITED: STRUCTURAL AND FUNCTIONAL ROLES OF VARIOUS METAL CATION BINDING SITES	FPA	0.08
2J5W	CERULOPLASMIN REVISITED: STRUCTURAL AND FUNCTIONAL ROLES OF VARIOUS METAL CATION BINDING SITES	GPM	0.09
1R1H	STRUCTURAL ANALYSIS OF NEPRILYSIN WITH VARIOUS SPECIFIC AND POTENT INHIBITORS	FPA	0.09
1KHB	PEPCK COMPLEX WITH NONHYDROLYZABLE GTP ANALOG_ NATIVE DATA	AAF	0.09
2YYO	CRYSTAL STURCTURE OF HUMAN SPRY DOMAIN	FPA	0.09
2R0B	CRYSTAL STRUCTURE OF HUMAN TYROSINE PHOSPHATASE-LIKE SERINE/THREONINE/TYROSINE-INTERACTING PROTEIN	AAF	0.10
2J5W	CERULOPLASMIN REVISITED: STRUCTURAL AND FUNCTIONAL ROLES OF VARIOUS METAL CATION BINDING SITES	AAF	0.10
2C9H	STRUCTURE OF MITOCHONDRIAL BETA-KETOACYL SYNTHASE	AAF	0.12
1OHC	STRUCTURE OF THE PROLINE DIRECTED PHOSPHATASE CDC14	AAF	0.12
2FOZ	HUMAN ADP-RIBOSYLHYDROLASE 3	AAF	0.12
2A8B	CRYSTAL STRUCTURE OF THE CATALYTIC DOMAIN OF HUMAN TYROSINE PHOSPHATASE RECEPTOR_ TYPE R	GPM	0.13
2DW4	CRYSTAL STRUCTURE OF HUMAN LSD1 AT 2.3 A RESOLUTION	AAF	0.13

2G62	CRYSTAL STRUCTURE OF HUMAN PTPA	AAF	0.13
1OI1	CRYSTAL STRUCTURE OF THE MBT DOMAINS OF HUMAN SCML2	FPA	0.13
1M13	CRYSTAL STRUCTURE OF THE HUMAN PREGANE X RECEPTOR LIGAND BINDING DOMAIN IN COMPLEX WITH HYPERFORIN_ A CONSTITUENT OF ST. JOHN'S WORT	AAF	0.13
2G62	CRYSTAL STRUCTURE OF HUMAN PTPA	AAF	0.13
1D5R	CRYSTAL STRUCTURE OF THE PTEN TUMOR SUPPRESSOR	FPA	0.13
2RJQ	CRYSTAL STRUCTURE OF ADAMTS5 WITH INHIBITOR BOUND	AAF	0.14
2I4I	CRYSTAL STRUCTURE OF HUMAN DEAD-BOX RNA HELICASE DDX3X	AAF	0.14
1KT0	STRUCTURE OF THE LARGE FKBP-LIKE PROTEIN_ FKBP51_ INVOLVED IN STEROID RECEPTOR COMPLEXES	AAF	0.15
3BI1	X-RAY STRUCTURE OF HUMAN GLUTAMATE CARBOXYPEPTIDASE II (GCPII) IN COMPLEX WITH A TRANSITION STATE ANALOG OF METHOTREXATE-GLU	AAF	0.15
2Q5I	CRYSTAL STRUCTURE OF APO S581L GLYCYL-TRNA SYNTHETASE MUTAN	FPA	0.15
2FST	MITOGEN ACTIVATED PROTEIN KINASE P38ALPHA (D176A+F327L) ACTIVATING MUTANT	AAF	0.16
2B9E	HUMAN NSUN5 PROTEIN	FPA	0.16
1MEO	HUMAN GLYCINAMIDE RIBONUCLEOTIDE TRANSFORMYLASE AT PH 4.2	FPA	0.16
3GRS	REFINED STRUCTURE OF GLUTATHIONE REDUCTASE AT 1.54 ANGSTROMS RESOLUTION	AAF	0.16
1NUF	ROLE OF CALCIUM IONS IN THE ACTIVATION AND ACTIVITY OF THE TRANSGLUTAMINASE 3 ENZYME	FPA	0.17
2PZ1	CRYSTAL STRUCTURE OF AUTO-INHIBITED ASEF	FPA	0.17
1ZC0	CRYSTAL STRUCTURE OF HUMAN HEMATOPOIETIC TYROSINE PHOSPHATASE (HEPTP) CATALYTIC DOMAIN	GPM	0.17
1XTQ	STRUCTURE OF SMALL GTPASE HUMAN RHEB IN COMPLEX WITH GDP	AAF	0.17
3CKK	CRYSTAL STRUCTURE OF HUMAN METHYLTRANSFERASE-LIKE PROTEIN 1	FPA	0.17
1T5I	CRYSTAL STRUCTURE OF THE C-TERMINAL DOMAIN OF UAP56	FPA	0.18
1OZN	1.5A CRYSTAL STRUCTURE OF THE NOGO RECEPTOR LIGAND BINDING DOMAIN REVEALS A CONVERGENT RECOGNITION SCAFFOLD MEDIATING INHIBITION OF MYELINATION	AAF	0.19
1HVF	STRUCTURAL AND ELECTROPHYSIOLOGICAL ANALYSIS OF ANNEXIN V MUTANTS. MUTAGENESIS OF HUMAN ANNEXIN V_ AN IN VITRO VOLTAGE-GATED CALCIUM CHANNEL_ PROVIDES INFORMATION ABOUT THE STRUCTURAL FEATURES OF THE ION PATHWAY_ THE VOLTAGE SENSOR AND THE ION SELECTIVITY FILTER	AAF	0.19
2RAJ	SO4 BOUND PX-BAR MEMBRANE REMODELING UNIT OF SORTING NEXIN	GPM	0.21
2GW2	CRYSTAL STRUCTURE OF THE PEPTIDYL-PROLYL ISOMERASE DOMAIN OF HUMAN CYCLOPHILIN G	AAF	0.21
3CHO	CRYSTAL STRUCTURE OF LEUKOTRIENE A4 HYDROLASE IN COMPLEX WITH 2-AMINO-N-[4-(PHENYLMETHOXY)PHENYL]-ACETAMIDE	AAF	0.22
1F6W	STRUCTURE OF THE CATALYTIC DOMAIN OF HUMAN BILE SALT ACTIVATED LIPASE	AAF	0.22
2UUR	N-TERMINAL NC4 DOMAIN OF COLLAGEN IX	AAF	0.23
2NZL	CRYSTAL STRUCTURE OF HUMAN HYDROXYACID OXIDASE 1	AAF	0.23
2UZ9	HUMAN GUANINE DEAMINASE (GUAD) IN COMPLEX WITH ZINC AND ITS PRODUCT XANTHINE.	FPA	0.23
1OW1	CRYSTAL STRUCTURE OF THE SPOC DOMAIN OF THE HUMAN TRANSCRIPTIONAL COREPRESSOR_ SHARP.	AAF	0.24
1SMB	CRYSTAL STRUCTURE OF GOLGI-ASSOCIATED PR-1 PROTEIN	FPA	0.24
2IQC	CRYSTAL STRUCTURE OF HUMAN FANCF PROTEIN THAT FUNCTIONS IN THE ASSEMBLY OF A DNA DAMAGE SIGNALING COMPLEX	AAF	0.24
2CY7	THE CRYSTAL STRUCTURE OF HUMAN ATG4B	FPA	0.25
2Q8K	THE CRYSTAL STRUCTURE OF EBP1	GPM	0.25
2IHD	CRYSTAL STRUCTURE OF HUMAN REGULATOR OF G-PROTEIN SIGNALING 8_ RGS8	AAF	0.25
1P0I	CRYSTAL STRUCTURE OF HUMAN BUTYRYL CHOLINESTERASE	AAF	0.25

1N5U	X-RAY STUDY OF HUMAN SERUM ALBUMIN COMPLEXED WITH HEME	AAF	0.26
2C9Y	STRUCTURE OF HUMAN ADENYLATE KINASE 2	AAF	0.26
1ZIV	CATALYTIC DOMAIN OF HUMAN CALPAIN-9	FPA	0.26
2UW2	CRYSTAL STRUCTURE OF HUMAN RIBONUCLEOTIDE REDUCTASE SUBUNIT R2	GPM	0.27
2OBI	CRYSTAL STRUCTURE OF THE SELENOCYSTEINE TO CYSTEINE MUTANT OF HUMAN PHOSPHOLIPID HYDROPEROXIDE GLUTATHIONE PEROXIDASE (GPX4)	GPM	0.28
1R2Q	CRYSTAL STRUCTURE OF HUMAN RAB5A GTPASE DOMAIN AT 1.05 A RESOLUTION	AAF	0.28
1Z32	STRUCTURE-FUNCTION RELATIONSHIPS IN HUMAN SALIVARY ALPHA-AMYLASE: ROLE OF AROMATIC RESIDUES	FPA	0.28
2FUE	HUMAN ALPHA-PHOSPHOMANNOMUTASE 1 WITH D-MANNOSE 1-PHOSPHATE AND MG2+ COFACTOR BOUND	AAF	0.29
1F5N	HUMAN GUANYLATE BINDING PROTEIN-1 IN COMPLEX WITH THE GTP ANALOGUE_ GMPPNP.	GPM	0.29
2AA2	MINERALOCORTICOID RECEPTOR WITH BOUND ALDOSTERONE	FPA	0.29
1ZS9	CRYSTAL STRUCTURE OF HUMAN ENOLASE-PHOSPHATASE E1	AAF	0.30
1N5U	X-RAY STUDY OF HUMAN SERUM ALBUMIN COMPLEXED WITH HEME	AAF	0.31
1M4K	CRYSTAL STRUCTURE OF THE HUMAN NATURAL KILLER CELL ACTIVATOR RECEPTOR KIR2DS2 (CD158J)	GPM	0.31
2GS3	CRYSTAL STRUCTURE OF THE SELENOCYSTEINE TO GLYCINE MUTANT OF HUMAN GLUTATHIONE PEROXIDASE 4(GPX4)	GPM	0.31
1TDH	CRYSTAL STRUCTURE OF HUMAN ENDONUCLEASE VIII-LIKE 1 (NEIL1)	AAF	0.32
2B1P	INHIBITOR COMPLEX OF JNK3	FPA	0.34
1D2S	CRYSTAL STRUCTURE OF THE N-TERMINAL LAMININ G-LIKE DOMAIN OF SHBG IN COMPLEX WITH DIHYDROTESTOSTERONE	FPA	0.35
1R1H	STRUCTURAL ANALYSIS OF NEPRILYSIN WITH VARIOUS SPECIFIC AND POTENT INHIBITORS	AAF	0.35
2AA2	MINERALOCORTICOID RECEPTOR WITH BOUND ALDOSTERONE	AAF	0.35
2FY7	CRYSTAL STRUCTURE OF THE CATALYTIC DOMAIN OF THE HUMAN BETA1_4-GALACTOSYLTRANSFERASE MUTANT M339H IN APO FORM	GPM	0.36
2Z5Y	CRYSTAL STRUCTURE OF HUMAN MONOAMINE OXIDASE A (G110A) WITH HARMINE	AAF	0.36
2QE3	CRYSTAL STRUCTURE OF HUMAN TL1A EXTRACELLULAR DOMAIN	FPA	0.37
2Z5J	FREE TRANSPORTIN 1	AAF	0.37
1EAX	CRYSTAL STRUCTURE OF MTSP1 (MATRIPTASE)	FPA	0.38
2OCG	CRYSTAL STRUCTURE OF HUMAN VALACYCLOVIR HYDROLASE	FPA	0.38
2HGS	HUMAN GLUTATHIONE SYNTHETASE	AAF	0.39
2HFX	STRUCTURAL AND KINETIC ANALYSIS OF PROTON SHUTTLE RESIDUES IN THE ACTIVE SITE OF HUMAN CARBONIC ANHYDRASE III	FPA	0.40
1Z8D	CRYSTAL STRUCTURE OF HUMAN MUSCLE GLYCOGEN PHOSPHORYLASE A WITH AMP AND GLUCOSE	AAF	0.42
1UMK	THE STRUCTURE OF HUMAN ERYTHROCYTE NADH-CYTOCHROME B5 REDUCTASE	FPA	0.42
2QQI	CRYSTAL STRUCTURE OF THE B1B2 DOMAINS FROM HUMAN NEUROPILIN 1	FPA	0.43
1FSU	4-SULFATASE (HUMAN)	FPA	0.43
3COU	CRYSTAL STRUCTURE OF HUMAN NUDIX MOTIF 16 (NUDT16)	AAF	0.44
1SO8	ABETA-BOUND HUMAN ABAD STRUCTURE [ALSO KNOWN AS 3-HYDROXYACYL-COA DEHYDROGENASE TYPE II (TYPE II HADH)_ ENDOPLASMIC RETICULUM-ASSOCIATED AMYLOID BETA-PEPTIDE BINDING PROTEIN (ERAB)]	AAF	0.49
3BGS	STRUCTURE OF HUMAN PURINE NUCLEOSIDE PHOSPHORYLASE WITH L-DADME-IMMH AND PHOSPHATE	FPA	0.49
2E9L	CRYSTAL STRUCTURE OF HUMAN CYTOSOLIC NEUTRAL BETA-GLYCOSYLCERAMIDASE (KLOTHO-RELATED PROTE:KLRP) COMPLEX WITH GLUCOSE AND FATTY ACIDS	FPA	0.50

1FCY	ISOTYPE SELECTIVITY OF THE HUMAN RETINOIC ACID NUCLEAR RECEPTOR HRAR: THE COMPLEX WITH THE RARBETA/GAMMA-SELECTIVE RETINOID CD564	GPM	0.51
1SO7	MALTOSE-INDUCED STRUCTURE OF THE HUMAN CYTOLSOLIC SIALIDASE NEU2	FPA	0.51
1FNH	CRYSTAL STRUCTURE OF HEPARIN AND INTEGRIN BINDING SEGMENT OF HUMAN FIBRONECTIN	GPM	0.52
1EWF	THE 1.7 ANGSTROM CRYSTAL STRUCTURE OF BPI	FPA	0.60
1WRM	CRYSTAL STRUCTURE OF JSP-1	GPM	0.62
2DE0	CRYSTAL STRUCTURE OF HUMAN ALPHA 1_6-FUCOSYLTRANSFERASE_ FUT8	AAF	0.64
1NRG	STRUCTURE AND PROPERTIES OF RECOMBINANT HUMAN PYRIDOXINE-5' PHOSPHATE OXIDASE	GPM	0.71
3BCH	CRYSTAL STRUCTURE OF THE HUMAN LAMININ RECEPTOR PRECURSOR	AAF	0.76
1P6F	STRUCTURE OF THE HUMAN NATURAL CYTOTOXICITY RECEPTOR NKP46	FPA	2.00
1ZKL	MULTIPLE DETERMINANTS FOR INHIBITOR SELECTIVITY OF CYCLIC NUCLEOTIDE PHOSPHODIESTERASES	AAF	2.00
2IQC	CRYSTAL STRUCTURE OF HUMAN FANCF PROTEIN THAT FUNCTIONS IN THE ASSEMBLY OF A DNA DAMAGE SIGNALING COMPLEX	GPM	2.00
	**tripeptidyl-peptidase II**		
1GEN	C-TERMINAL DOMAIN OF GELATINASE A	AAF	0.06
2UXW	CRYSTAL STRUCTURE OF HUMAN VERY LONG CHAIN ACYL-COA DEHYDROGENASE (ACADVL)	AAF	0.07
2B3X	STRUCTURE OF AN ORTHORHOMBIC CRYSTAL FORM OF HUMAN CYTOSOLIC ACONITASE (IRP1)	AAF	0.07
1M6I	CRYSTAL STRUCTURE OF APOPTOSIS INDUCING FACTOR (AIF)	AAF	0.07
1P49	STRUCTURE OF HUMAN PLACENTAL ESTRONE/DHEA SULFATASE	AAF	0.07
1T32	A DUAL INHIBITOR OF THE LEUKOCYTE PROTEASES CATHEPSIN G AND CHYMASE WITH THERAPEUTIC EFFICACY IN ANIMALS MODELS OF INFLAMMATION	AAF	0.08
1KHB	PEPCK COMPLEX WITH NONHYDROLYZABLE GTP ANALOG_ NATIVE DATA	AAF	0.09
2R0B	CRYSTAL STRUCTURE OF HUMAN TYROSINE PHOSPHATASE-LIKE SERINE/THREONINE/TYROSINE-INTERACTING PROTEIN	AAF	0.10
2J5W	CERULOPLASMIN REVISITED: STRUCTURAL AND FUNCTIONAL ROLES OF VARIOUS METAL CATION BINDING SITES	AAF	0.10
2C9H	STRUCTURE OF MITOCHONDRIAL BETA-KETOACYL SYNTHASE	AAF	0.12
1OHC	STRUCTURE OF THE PROLINE DIRECTED PHOSPHATASE CDC14	AAF	0.12
2FOZ	HUMAN ADP-RIBOSYLHYDROLASE 3	AAF	0.12
2DW4	CRYSTAL STRUCTURE OF HUMAN LSD1 AT 2.3 A RESOLUTION	AAF	0.13
2G62	CRYSTAL STRUCTURE OF HUMAN PTPA	AAF	0.13
1M13	CRYSTAL STRUCTURE OF THE HUMAN PREGANE X RECEPTOR LIGAND BINDING DOMAIN IN COMPLEX WITH HYPERFORIN_ A CONSTITUENT OF ST. JOHN'S WORT	AAF	0.13
2G62	CRYSTAL STRUCTURE OF HUMAN PTPA	AAF	0.13
2RJQ	CRYSTAL STRUCTURE OF ADAMTS5 WITH INHIBITOR BOUND	AAF	0.14
2I4I	CRYSTAL STRUCTURE OF HUMAN DEAD-BOX RNA HELICASE DDX3X	AAF	0.14
1KT0	STRUCTURE OF THE LARGE FKBP-LIKE PROTEIN_ FKBP51_ INVOLVED IN STEROID RECEPTOR COMPLEXES	AAF	0.15
3BI1	X-RAY STRUCTURE OF HUMAN GLUTAMATE CARBOXYPEPTIDASE II (GCPII) IN COMPLEX WITH A TRANSITION STATE ANALOG OF METHOTREXATE-GLU	AAF	0.15
2FST	MITOGEN ACTIVATED PROTEIN KINASE P38ALPHA (D176A+F327L) ACTIVATING MUTANT	AAF	0.16
3GRS	REFINED STRUCTURE OF GLUTATHIONE REDUCTASE AT 1.54 ANGSTROMS RESOLUTION	AAF	0.16
1XTQ	STRUCTURE OF SMALL GTPASE HUMAN RHEB IN COMPLEX WITH GDP	AAF	0.17

1OZN	1.5A CRYSTAL STRUCTURE OF THE NOGO RECEPTOR LIGAND BINDING DOMAIN REVEALS A CONVERGENT RECOGNITION SCAFFOLD MEDIATING INHIBITION OF MYELINATION	AAF	0.19
1HVF	STRUCTURAL AND ELECTROPHYSIOLOGICAL ANALYSIS OF ANNEXIN V MUTANTS. MUTAGENESIS OF HUMAN ANNEXIN V_ AN IN VITRO VOLTAGE-GATED CALCIUM CHANNEL_ PROVIDES INFORMATION ABOUT THE STRUCTURAL FEATURES OF THE ION PATHWAY_ THE VOLTAGE SENSOR AND THE ION SELECTIVITY FILTER	AAF	0.19
2GW2	CRYSTAL STRUCTURE OF THE PEPTIDYL-PROLYL ISOMERASE DOMAIN OF HUMAN CYCLOPHILIN G	AAF	0.21
3CHO	CRYSTAL STRUCTURE OF LEUKOTRIENE A4 HYDROLASE IN COMPLEX WITH 2-AMINO-N-[4-(PHENYLMETHOXY)PHENYL]-ACETAMIDE	AAF	0.22
1F6W	STRUCTURE OF THE CATALYTIC DOMAIN OF HUMAN BILE SALT ACTIVATED LIPASE	AAF	0.22
2UUR	N-TERMINAL NC4 DOMAIN OF COLLAGEN IX	AAF	0.23
2NZL	CRYSTAL STRUCTURE OF HUMAN HYDROXYACID OXIDASE 1	AAF	0.23
1OW1	CRYSTAL STRUCTURE OF THE SPOC DOMAIN OF THE HUMAN TRANSCRIPTIONAL COREPRESSOR_ SHARP.	AAF	0.24
2IQC	CRYSTAL STRUCTURE OF HUMAN FANCF PROTEIN THAT FUNCTIONS IN THE ASSEMBLY OF A DNA DAMAGE SIGNALING COMPLEX	AAF	0.24
2IHD	CRYSTAL STRUCTURE OF HUMAN REGULATOR OF G-PROTEIN SIGNALING 8_ RGS8	AAF	0.25
1P0I	CRYSTAL STRUCTURE OF HUMAN BUTYRYL CHOLINESTERASE	AAF	0.25
1N5U	X-RAY STUDY OF HUMAN SERUM ALBUMIN COMPLEXED WITH HEME	AAF	0.26
2C9Y	STRUCTURE OF HUMAN ADENYLATE KINASE 2	AAF	0.26
1R2Q	CRYSTAL STRUCTURE OF HUMAN RAB5A GTPASE DOMAIN AT 1.05 A RESOLUTION	AAF	0.28
2FUE	HUMAN ALPHA-PHOSPHOMANNOMUTASE 1 WITH D-MANNOSE 1-PHOSPHATE AND MG2+ COFACTOR BOUND	AAF	0.29
1ZS9	CRYSTAL STRUCTURE OF HUMAN ENOLASE-PHOSPHATASE E1	AAF	0.30
1N5U	X-RAY STUDY OF HUMAN SERUM ALBUMIN COMPLEXED WITH HEME	AAF	0.31
1TDH	CRYSTAL STRUCTURE OF HUMAN ENDONUCLEASE VIII-LIKE 1 (NEIL1)	AAF	0.32
1R1H	STRUCTURAL ANALYSIS OF NEPRILYSIN WITH VARIOUS SPECIFIC AND POTENT INHIBITORS	AAF	0.35
2AA2	MINERALOCORTICOID RECEPTOR WITH BOUND ALDOSTERONE	AAF	0.35
2Z5Y	CRYSTAL STRUCTURE OF HUMAN MONOAMINE OXIDASE A (G110A) WITH HARMINE	AAF	0.36
2Z5J	FREE TRANSPORTIN 1	AAF	0.37
2HGS	HUMAN GLUTATHIONE SYNTHETASE	AAF	0.39
1Z8D	CRYSTAL STRUCTURE OF HUMAN MUSCLE GLYCOGEN PHOSPHORYLASE A WITH AMP AND GLUCOSE	AAF	0.42
3COU	CRYSTAL STRUCTURE OF HUMAN NUDIX MOTIF 16 (NUDT16)	AAF	0.44
1SO8	ABETA-BOUND HUMAN ABAD STRUCTURE [ALSO KNOWN AS 3-HYDROXYACYL-COA DEHYDROGENASE TYPE II (TYPE II HADH)_ ENDOPLASMIC RETICULUM-ASSOCIATED AMYLOID BETA-PEPTIDE BINDING PROTEIN (ERAB)]	AAF	0.49
2DE0	CRYSTAL STRUCTURE OF HUMAN ALPHA 1_6-FUCOSYLTRANSFERASE_ FUT8	AAF	0.64
3BCH	CRYSTAL STRUCTURE OF THE HUMAN LAMININ RECEPTOR PRECURSOR	AAF	0.76
1ZKL	MULTIPLE DETERMINANTS FOR INHIBITOR SELECTIVITY OF CYCLIC NUCLEOTIDE PHOSPHODIESTERASES	AAF	2.00
	**trypsin-2**		
2A2K	CRYSTAL STRUCTURE OF AN ACTIVE SITE MUTANT_ C473S_ OF CDC25B PHOSPHATASE CATALYTIC DOMAIN	GPR	0.19
1F5N	HUMAN GUANYLATE BINDING PROTEIN-1 IN COMPLEX WITH THE GTP ANALOGUE_ GMPPNP.	GPR	0.19
1C25	HUMAN CDC25A CATALYTIC DOMAIN	GPR	0.21

1IAT	CRYSTAL STRUCTURE OF HUMAN PHOSPHOGLUCOSE ISOMERASE/NEUROLEUKIN/AUTOCRINE MOTILITY FACTOR/MATURATION FACTOR	GPR	0.27
1KT0	STRUCTURE OF THE LARGE FKBP-LIKE PROTEIN_ FKBP51_ INVOLVED IN STEROID RECEPTOR COMPLEXES	GPR	0.28
2B69	CRYSTAL STRUCTURE OF HUMAN UDP-GLUCORONIC ACID DECARBOXYLAS	GPR	0.28
2JHM	STRUCTURE OF GLOBULAR HEADS OF M-FICOLIN AT NEUTRAL PH	GPR	0.29
2Z6O	CRYSTAL STRUCTURE OF THE UFC1_ UFM1 CONJUGATING ENZYME 1	GPR	0.30
1KO9	NATIVE STRUCTURE OF THE HUMAN 8-OXOGUANINE DNA GLYCOSYLASE HOGG1	GPR	0.30
1TQN	CRYSTAL STRUCTURE OF HUMAN MICROSOMAL P450 3A4	GPR	0.34
1ZVD	REGULATION OF SMURF2 UBIQUITIN LIGASE ACTIVITY BY ANCHORING THE E2 TO THE HECT DOMAIN	GPR	0.34
2HI4	CRYSTAL STRUCTURE OF HUMAN MICROSOMAL P450 1A2 IN COMPLEX WITH ALPHA-NAPHTHOFLAVONE	GPR	0.35
1D2S	CRYSTAL STRUCTURE OF THE N-TERMINAL LAMININ G-LIKE DOMAIN OF SHBG IN COMPLEX WITH DIHYDROTESTOSTERONE	GPR	0.38
2B69	CRYSTAL STRUCTURE OF HUMAN UDP-GLUCORONIC ACID DECARBOXYLAS	GPR	0.40
1N26	CRYSTAL STRUCTURE OF THE EXTRA-CELLULAR DOMAINS OF HUMAN INTERLEUKIN-6 RECEPTOR ALPHA CHAIN	GPR	0.41
1S31	CRYSTAL STRUCTURE ANALYSIS OF THE HUMAN TUB PROTEIN (ISOFORM A) SPANNING RESIDUES 289 THROUGH 561	GPR	0.44
1HDO	HUMAN BILIVERDIN IX BETA REDUCTASE: NADP COMPLEX	GPR	0.45
3CHO	CRYSTAL STRUCTURE OF LEUKOTRIENE A4 HYDROLASE IN COMPLEX WITH 2-AMINO-N-[4-(PHENYLMETHOXY)PHENYL]-ACETAMIDE	GPR	0.48
1CB0	STRUCTURE OF HUMAN 5'-DEOXY-5'-METHYLTHIOADENOSINE PHOSPHORYLASE AT 1.7 A RESOLUTION	GPR	0.50
1Q33	CRYSTAL STRUCTURE OF HUMAN ADP-RIBOSE PYROPHOSPHATASE NUDT9	GPR	0.53
1OZ2	CRYSTAL STRUCTURE OF 3-MBT REPEATS OF LETHAL (3) MALIGNANT BRAIN TUMOR (NATIVE-II) AT 1.55 ANGSTROM	GPR	0.54
1TDH	CRYSTAL STRUCTURE OF HUMAN ENDONUCLEASE VIII-LIKE 1 (NEIL1)	GPR	0.56
1PME	STRUCTURE OF PENTA MUTANT HUMAN ERK2 MAP KINASE COMPLEXED WITH A SPECIFIC INHIBITOR OF HUMAN P38 MAP KINASE	GPR	0.60
1PI1	CRYSTAL STRUCTURE OF A HUMAN MOB1 PROTEIN; TOWARD UNDERSTANDING MOB-REGULATED CELL CYCLE PATHWAYS.	GPR	0.61
1VZO	THE STRUCTURE OF THE N-TERMINAL KINASE DOMAIN OF MSK1 REVEALS A NOVEL AUTOINHIBITORY CONFORMATION FOR A DUAL KINASE PROTEIN	GPR	0.62
3COU	CRYSTAL STRUCTURE OF HUMAN NUDIX MOTIF 16 (NUDT16)	GPR	0.73
1CZA	MUTANT MONOMER OF RECOMBINANT HUMAN HEXOKINASE TYPE I COMPLEXED WITH GLUCOSE_ GLUCOSE-6-PHOSPHATE_ AND ADP	GPR	0.74
2VPJ	CRYSTAL STRUCTURE OF THE KELCH DOMAIN OF HUMAN KLHL12	GPR	2.00
	**tryptase beta**		
1ZGK	1.35 ANGSTROM STRUCTURE OF THE KELCH DOMAIN OF KEAP1	RNR	0.13
2DW4	CRYSTAL STRUCTURE OF HUMAN LSD1 AT 2.3 A RESOLUTION	RNR	0.18
3COI	CRYSTAL STRUCTURE OF P38DELTA KINASE	AKR	0.20
1X0X	CO-STRUCTURE OF HOMO SAPIENS GLYCEROL-3-PHOSPHATE DEHYDROGENASE 1 COMPLEX WITH NAD	RNR	0.22
2FST	MITOGEN ACTIVATED PROTEIN KINASE P38ALPHA (D176A+F327L) ACTIVATING MUTANT	AKR	0.23
3C5E	CRYSTAL STRUCTURE OF HUMAN ACYL-COA SYNTHETASE MEDIUM-CHAIN FAMILY MEMBER 2A (L64P MUTATION) IN COMPLEX WITH ATP	KAK	0.23
2J7T	CRYSTAL STRUCTURE OF HUMAN SERINE THREONINE KINASE-10 BOUND TO SU11274	KAK	0.23
1R1H	STRUCTURAL ANALYSIS OF NEPRILYSIN WITH VARIOUS SPECIFIC AND POTENT INHIBITORS	KAK	0.24

2A14	CRYSTAL STRUCTURE OF HUMAN INDOLETHYLAMINE N-METHYLTRANSFERASE WITH SAH	RNR	0.25
2E9L	CRYSTAL STRUCTURE OF HUMAN CYTOSOLIC NEUTRAL BETA-GLYCOSYLCERAMIDASE (KLOTHO-RELATED PROTE:KLRP) COMPLEX WITH GLUCOSE AND FATTY ACIDS	AKR	0.25
2OAY	CRYSTAL STRUCTURE OF LATENT HUMAN C1-INHIBITOR	KAK	0.25
2HE7	FERM DOMAIN OF EPB41L3 (DAL-1)	AKR	0.26
2B1P	INHIBITOR COMPLEX OF JNK3	AKR	0.27
2EVA	STRUCTURAL BASIS FOR THE INTERACTION OF TAK1 KINASE WITH ITS ACTIVATING PROTEIN TAB1	KAK	0.27
1QMN	ALPHA1-ANTICHYMOTRYPSIN SERPIN IN THE DELTA CONFORMATION (PARTIAL LOOP INSERTION)	KAK	0.27
1X3S	CRYSTAL STRUCTURE OF HUMAN RAB18 IN COMPLEX WITH GPPNHP	KAK	0.28
1ZHR	CRYSTAL STRUCTURE OF THE CATALYTIC DOMAIN OF COAGULATION FACTOR XI IN COMPLEX WITH BENZAMIDINE (S434A-T475A-C482S- K437A MUTANT)	KAK	0.28
3C5E	CRYSTAL STRUCTURE OF HUMAN ACYL-COA SYNTHETASE MEDIUM-CHAIN FAMILY MEMBER 2A (L64P MUTATION) IN COMPLEX WITH ATP	KAK	0.28
2E8A	CRYSTAL STRUCTURE OF THE HUMAN HSP70 ATPASE DOMAIN IN COMPLEX WITH AMP-PNP	AKR	0.28
3BO5	CRYSTAL STRUCTURE OF METHYLTRANSFERASE DOMAIN OF HUMAN HISTONE-LYSINE N-METHYLTRANSFERASE SETMAR	RNR	0.29
2VFK	AKAP18 DELTA CENTRAL DOMAIN - AMP	AKR	0.29
1NM8	STRUCTURE OF HUMAN CARNITINE ACETYLTRANSFERASE: MOLECULAR BASIS FOR FATTY ACYL TRANSFER	KAK	0.29
2E8A	CRYSTAL STRUCTURE OF THE HUMAN HSP70 ATPASE DOMAIN IN COMPLEX WITH AMP-PNP	AKR	0.29
2F71	PROTEIN TYROSINE PHOSPHATASE 1B WITH SULFAMIC ACID INHIBITORS	RNR	0.29
1IAT	CRYSTAL STRUCTURE OF HUMAN PHOSPHOGLUCOSE ISOMERASE/NEUROLEUKIN/AUTOCRINE MOTILITY FACTOR/MATURATION FACTOR	RNR	0.29
3COU	CRYSTAL STRUCTURE OF HUMAN NUDIX MOTIF 16 (NUDT16)	AKR	0.29
1L8K	T CELL PROTEIN-TYROSINE PHOSPHATASE STRUCTURE	RNR	0.30
1R3S	UROPORPHYRINOGEN DECARBOXYLASE SINGLE MUTANT D86G IN COMPLEX WITH COPROPORPHYRINOGEN-I	AKR	0.30
1XPC	HUMAN ESTROGEN RECEPTOR ALPHA LIGAND-BINDING DOMAIN IN COMPLEX WITH COMPOUND 19	AKR	0.31
1Q20	CRYSTAL STRUCTURE OF HUMAN CHOLESTEROL SULFOTRANSFERASE (SULT2B1B) IN THE PRESENCE OF PAP AND PREGNENOLONE	KAK	0.31
2IJA	HUMAN N-ACETYLTRANSFERASE 1 F125S MUTANT	RNR	0.31
1LBD	LIGAND-BINDING DOMAIN OF THE HUMAN NUCLEAR RECEPTOR RXR-ALPHA	AKR	0.31
1P0I	CRYSTAL STRUCTURE OF HUMAN BUTYRYL CHOLINESTERASE	RNR	0.32
2QMJ	CRYSTRAL STRUCTURE OF THE N-TERMINAL SUBUNIT OF HUMAN MALTASE-GLUCOAMYLASE IN COMPLEX WITH ACARBOSE	RNR	0.32
1XAP	STRUCTURE OF THE LIGAND BINDING DOMAIN OF THE RETINOIC ACID RECEPTOR BETA	AKR	0.32
2REW	CRYSTAL STRUCTURE OF PPARALPHA LIGAND BINDING DOMAIN WITH BMS-631707	AKR	0.32
1N83	CRYSTAL STRUCTURE OF THE COMPLEX BETWEEN THE ORPHAN NUCLEAR HORMONE RECEPTOR ROR(ALPHA)-LBD AND CHOLESTEROL	AKR	0.33
1FCY	ISOTYPE SELECTIVITY OF THE HUMAN RETINOIC ACID NUCLEAR RECEPTOR HRAR: THE COMPLEX WITH THE RARBETA/GAMMA-SELECTIVE RETINOID CD564	AKR	0.33
1N11	D34 REGION OF HUMAN ANKYRIN-R AND LINKER	AKR	0.33

2DW5	CRYSTAL STRUCTURE OF HUMAN PEPTIDYLARGININE DEIMINASE 4 IN COMPLEX WITH N-ALPHA-BENZOYL-N5-(2-FLUORO-1-IMINOETHYL)-L-ORNITHINE AMIDE	RNR	0.33
2UWN	CRYSTAL STRUCTURE OF HUMAN COMPLEMENT FACTOR H_ SCR DOMAINS 6-8 (H402 RISK VARIANT)_ IN COMPLEX WITH LIGAND.	KAK	0.33
1CB0	STRUCTURE OF HUMAN 5'-DEOXY-5'-METHYLTHIOADENOSINE PHOSPHORYLASE AT 1.7 A RESOLUTION	KAK	0.33
1J99	CRYSTAL STRUCTURE OF HUMAN DEHYDROEPIANDROSTERONE SULFOTRANSFERASE IN COMPLEX WITH SUBSTRATE	KAK	0.34
1RW6	HUMAN APP CORE DOMAIN	KAK	0.34
1QUU	CRYSTAL STRUCTURE OF TWO CENTRAL SPECTRIN-LIKE REPEATS FROM ALPHA-ACTININ	AKR	0.34
1H0C	THE CRYSTAL STRUCTURE OF HUMAN ALANINE:GLYOXYLATE AMINOTRANSFERASE	KAK	0.35
1TFF	STRUCTURE OF OTUBAIN-2	RNR	0.35
1NST	THE SULFOTRANSFERASE DOMAIN OF HUMAN HAPARIN SULFATE N-DEACETYLASE/N-SULFOTRANSFERASE	KAK	0.35
2B3X	STRUCTURE OF AN ORTHORHOMBIC CRYSTAL FORM OF HUMAN CYTOSOLIC ACONITASE (IRP1)	RNR	0.36
1YZQ	GPPNHP-BOUND RAB6 GTPASE	KAK	0.36
1EEM	GLUTATHIONE TRANSFERASE FROM HOMO SAPIENS	KAK	0.36
2EFK	CRYSTAL STRUCTURE OF THE EFC DOMAIN OF CDC42-INTERACTING PROTEIN 4	KAK	0.36
1MD8	MONOMERIC STRUCTURE OF THE ACTIVE CATALYTIC DOMAIN OF COMPLEMENT PROTEASE C1R	KAK	0.37
1F6W	STRUCTURE OF THE CATALYTIC DOMAIN OF HUMAN BILE SALT ACTIVATED LIPASE	KAK	0.37
2JI4	HUMAN PHOSPHORIBOSYLPYROPHOSPHATE SYNTHETASE- ASSOCIATED PROTEIN 41 (PAP41)	AKR	0.37
1VZO	THE STRUCTURE OF THE N-TERMINAL KINASE DOMAIN OF MSK1 REVEALS A NOVEL AUTOINHIBITORY CONFORMATION FOR A DUAL KINASE PROTEIN	KAK	0.37
1QMN	ALPHA1-ANTICHYMOTRYPSIN SERPIN IN THE DELTA CONFORMATION (PARTIAL LOOP INSERTION)	AKR	0.37
2AHE	CRYSTAL STRUCTURE OF A SOLUBLE FORM OF CLIC4. INTERCELLULAR CHLORIDE ION CHANNEL	AKR	0.37
1N5U	X-RAY STUDY OF HUMAN SERUM ALBUMIN COMPLEXED WITH HEME	AKR	0.38
2B69	CRYSTAL STRUCTURE OF HUMAN UDP-GLUCORONIC ACID DECARBOXYLAS	KAK	0.38
2B69	CRYSTAL STRUCTURE OF HUMAN UDP-GLUCORONIC ACID DECARBOXYLAS	AKR	0.38
3BZH	CRYSTAL STRUCTURE OF HUMAN UBIQUITIN-CONJUGATING ENZYME E2 E1	AKR	0.38
1N5U	X-RAY STUDY OF HUMAN SERUM ALBUMIN COMPLEXED WITH HEME	AKR	0.39
2ALD	HUMAN MUSCLE ALDOLASE	AKR	0.39
1A6Q	CRYSTAL STRUCTURE OF THE PROTEIN SERINE/THREONINE PHOSPHATASE 2C AT 2 A RESOLUTION	RNR	0.39
2HJW	CRYSTAL STRUCTURE OF THE BC DOMAIN OF ACC2	AKR	0.39
2PNY	STRUCTURE OF HUMAN ISOPENTENYL-DIPHOSPHATE DELTA-ISOMERASE	KAK	0.39
1N11	D34 REGION OF HUMAN ANKYRIN-R AND LINKER	KAK	0.40
1N11	D34 REGION OF HUMAN ANKYRIN-R AND LINKER	KAK	0.40
2DE0	CRYSTAL STRUCTURE OF HUMAN ALPHA 1_6-FUCOSYLTRANSFERASE_ FUT8	KAK	0.40
3BKB	CRYSTAL STRUCTURE OF HUMAN FELINE SARCOMA VIRAL ONCOGENE HOMOLOGUE (V-FES)	KAK	0.40
2Q8G	STRUCTURE OF PYRUVATE DEHYDROGENASE KINASE ISOFORM 1 IN COMPLEX WITH GLUCOSE-LOWERING DRUG AZD7545	RNR	0.40
5PNT	CRYSTAL STRUCTURE OF A HUMAN LOW MOLECULAR WEIGHT PHOSPHOTYROSYL PHOSPHATASE. IMPLICATIONS FOR SUBSTRATE SPECIFICITY	KAK	0.40
1L8K	T CELL PROTEIN-TYROSINE PHOSPHATASE STRUCTURE	RNR	0.40

1XWW	CRYSTAL STRUCTURE OF HUMAN B-FORM LOW MOLECULAR WEIGHT PHOSPHOTYROSYL PHOSPHATASE AT 1.6 ANGSTROM RESOLUTION	KAK	0.40
2E3N	CRYSTAL STRUCTURE OF CERT START DOMAIN IN COMPLEX WITH C6-CERAMIDE (P212121)	AKR	0.41
1EMR	CRYSTAL STRUCTURE OF HUMAN LEUKEMIA INHIBITORY FACTOR (LIF)	KAK	0.41
2HEH	CRYSTAL STRUCTURE OF THE KIF2C MOTOR DOMAIN (CASP TARGET)	KAK	0.42
2BU7	CRYSTAL STRUCTURES OF HUMAN PYRUVATE DEHYDROGENASE KINASE 2 CONTAINING PHYSIOLOGICAL AND SYNTHETIC LIGANDS	RNR	0.42
3PRG	LIGAND BINDING DOMAIN OF HUMAN PEROXISOME PROLIFERATOR ACTIVATED RECEPTOR	KAK	0.42
1ZJK	CRYSTAL STRUCTURE OF THE ZYMOGEN CATALYTIC REGION OF HUMAN MASP-2	KAK	0.42
1KT0	STRUCTURE OF THE LARGE FKBP-LIKE PROTEIN_ FKBP51_ INVOLVED IN STEROID RECEPTOR COMPLEXES	KAK	0.43
2QQI	CRYSTAL STRUCTURE OF THE B1B2 DOMAINS FROM HUMAN NEUROPILIN 1	KAK	0.43
3BIY	CRYSTAL STRUCTURE OF P300 HISTONE ACETYLTRANSFERASE DOMAIN IN COMPLEX WITH A BISUBSTRATE INHIBITOR_ LYS-COA	AKR	0.43
2JEO	CRYSTAL STRUCTURE OF HUMAN URIDINE-CYTIDINE KINASE 1	KAK	0.44
2Q8K	THE CRYSTAL STRUCTURE OF EBP1	KAK	0.44
1JK7	CRYSTAL STRUCTURE OF THE TUMOR-PROMOTER OKADAIC ACID BOUND TO PROTEIN PHOSPHATASE-1	AKR	0.45
1ZSQ	CRYSTAL STRUCTURE OF MTMR2 IN COMPLEX WITH PHOSPHATIDYLINOSITOL 3-PHOSPHATE	KAK	0.45
1OHC	STRUCTURE OF THE PROLINE DIRECTED PHOSPHATASE CDC14	AKR	0.45
2D7I	CRSYTAL STRUCTURE OF PP-GALNAC-T10 WITH UDP_ GALNAC AND MN2	RNR	0.45
2Q0Z	CRYSTAL STRUCTURE OF Q9P172/SEC63 FROM HOMO SAPIENS. NORTHEAST STRUCTURAL GENOMICS TARGET HR1979.	KAK	0.46
2R3A	METHYLTRANSFERASE DOMAIN OF HUMAN SUPPRESSOR OF VARIEGATION 3-9 HOMOLOG 2	KAK	0.46
2RKU	STRUCTURE OF PLK1 IN COMPLEX WITH BI2536	RNR	0.46
2DW5	CRYSTAL STRUCTURE OF HUMAN PEPTIDYLARGININE DEIMINASE 4 IN COMPLEX WITH N-ALPHA-BENZOYL-N5-(2-FLUORO-1-IMINOETHYL)-L-ORNITHINE AMIDE	KAK	0.47
2P39	CRYSTAL STRUCTURE OF HUMAN FGF23	AKR	0.47
1R8M	SEC7 DOMAIN OF THE ARF EXCHANGE FACTOR ARNO WITH BREFELDIN A-SENSITIZING MUTATIONS	RNR	0.47
1KT0	STRUCTURE OF THE LARGE FKBP-LIKE PROTEIN_ FKBP51_ INVOLVED IN STEROID RECEPTOR COMPLEXES	KAK	0.48
1ALU	HUMAN INTERLEUKIN-6	KAK	0.49
2B1P	INHIBITOR COMPLEX OF JNK3	AKR	0.49
2FY7	CRYSTAL STRUCTURE OF THE CATALYTIC DOMAIN OF THE HUMAN BETA1_4-GALACTOSYLTRANSFERASE MUTANT M339H IN APO FORM	RNR	0.50
2V7O	CRYSTAL STRUCTURE OF HUMAN CALCIUM-CALMODULIN-DEPENDENT PROTEIN KINASE II GAMMA	AKR	0.50
1T46	STRUCTURAL BASIS FOR THE AUTOINHIBITION AND STI-571 INHIBITION OF C-KIT TYROSINE KINASE	RNR	0.51
2Z7R	CRYSTAL STRUCTURE OF THE N-TERMINAL KINASE DOMAIN OF HUMAN RSK1 BOUND TO STAUROSPORINE	KAK	0.51
2B9E	HUMAN NSUN5 PROTEIN	AKR	0.51
2NZL	CRYSTAL STRUCTURE OF HUMAN HYDROXYACID OXIDASE 1	RNR	0.51
1ZED	ALKALINE PHOSPHATASE FROM HUMAN PLACENTA IN COMPLEX WITH P-NITROPHENYL-PHOSPHONATE	AKR	0.52
1Z57	CRYSTAL STRUCTURE OF HUMAN CLK1 IN COMPLEX WITH 10Z-HYMENIALDISINE	AKR	0.52
2PBN	CRYSTAL STRUCTURE OF THE HUMAN TYROSINE RECEPTOR PHOSPHATE GAMMA	KAK	0.52
2NSM	CRYSTAL STRUCTURE OF THE HUMAN CARBOXYPEPTIDASE N (KININASE I) CATALYTIC DOMAIN	RNR	0.53

1T32	A DUAL INHIBITOR OF THE LEUKOCYTE PROTEASES CATHEPSIN G AND CHYMASE WITH THERAPEUTIC EFFICACY IN ANIMALS MODELS OF INFLAMMATION	RNR	0.53
1HD2	HUMAN PEROXIREDOXIN 5	KAK	0.53
1ZUA	CRYSTAL STRUCTURE OF AKR1B10 COMPLEXED WITH NADP+ AND TOLRESTAT	KAK	0.54
2VKQ	CRYSTAL STRUCTURE OF HUMAN CYTOSOLIC 5'-NUCLEOTIDASE III (CN-III_ NT5C3) IN COMPLEX WITH BERYLLIUM TRIFLUORIDE	KAK	0.56
1B0F	CRYSTAL STRUCTURE OF HUMAN NEUTROPHIL ELASTASE WITH MDL 101 146	RNR	0.56
1Q1C	CRYSTAL STRUCTURE OF N(1-260) OF HUMAN FKBP52	KAK	0.57
1HU3	MIDDLE DOMAIN OF HUMAN EIF4GII	KAK	0.59
1A17	TETRATRICOPEPTIDE REPEATS OF PROTEIN PHOSPHATASE 5	KAK	0.61
1R9O	CRYSTAL STRUCTURE OF P4502C9 WITH FLURBIPROFEN BOUND	RNR	0.64
1NN5	CRYSTAL STRUCTURE OF HUMAN THYMIDYLATE KINASE WITH D4TMP + APPNHP	AKR	0.70
3BPT	CRYSTAL STRUCTURE OF HUMAN BETA-HYDROXYISOBUTYRYL-COA HYDROLASE IN COMPLEX WITH QUERCETIN	KAK	0.75
1NG2	STRUCTURE OF AUTOINHIBITED P47PHOX	KAK	0.82
2HWX	STRUCTURE OF HUMAN SMG6 E1282C PIN DOMAIN MUTANT.	KAK	0.82
2QHN	CRYSTAL STRUCTURE OF CHEK1 IN COMPLEX WITH INHIBITOR 1A	AKR	0.94
1NG2	STRUCTURE OF AUTOINHIBITED P47PHOX	AKR	0.96
1KO9	NATIVE STRUCTURE OF THE HUMAN 8-OXOGUANINE DNA GLYCOSYLASE HOGG1	AKR	2.00
1TDH	CRYSTAL STRUCTURE OF HUMAN ENDONUCLEASE VIII-LIKE 1 (NEIL1)	AKR	2.00
1XGW	THE CRYSTAL STRUCTURE OF HUMAN ENTHOPROTIN N-TERMINAL DOMAI	KAK	2.00
1XMJ	CRYSTAL STRUCTURE OF HUMAN DELTAF508 HUMAN NBD1 DOMAIN WITH ATP	KAK	2.00
2DYL	CRYSTAL STRUCTURE OF HUMAN MITOGEN-ACTIVATED PROTEIN KINASE KINASE 7 ACTIVATED MUTANT (S287D_ T291D)	KAK	2.00
2JC9	CRYSTAL STRUCTURE OF HUMAN CYTOSOLIC 5'-NUCLEOTIDASE II IN COMPLEX WITH ADENOSINE	RNR	2.00
	**tryptase gamma 1**		
2A2K	CRYSTAL STRUCTURE OF AN ACTIVE SITE MUTANT_ C473S_ OF CDC25B PHOSPHATASE CATALYTIC DOMAIN	GPR	0.19
2ALD	HUMAN MUSCLE ALDOLASE	KWR	0.19
1F5N	HUMAN GUANYLATE BINDING PROTEIN-1 IN COMPLEX WITH THE GTP ANALOGUE_ GMPPNP.	GPR	0.19
1C25	HUMAN CDC25A CATALYTIC DOMAIN	GPR	0.21
1IAT	CRYSTAL STRUCTURE OF HUMAN PHOSPHOGLUCOSE ISOMERASE/NEUROLEUKIN/AUTOCRINE MOTILITY FACTOR/MATURATION FACTOR	GPR	0.27
1KT0	STRUCTURE OF THE LARGE FKBP-LIKE PROTEIN_ FKBP51_ INVOLVED IN STEROID RECEPTOR COMPLEXES	GPR	0.28
2B69	CRYSTAL STRUCTURE OF HUMAN UDP-GLUCORONIC ACID DECARBOXYLAS	GPR	0.28
2JHM	STRUCTURE OF GLOBULAR HEADS OF M-FICOLIN AT NEUTRAL PH	GPR	0.29
2Z6O	CRYSTAL STRUCTURE OF THE UFC1_ UFM1 CONJUGATING ENZYME 1	GPR	0.30
1R5L	CRYSTAL STRUCTURE OF HUMAN ALPHA-TOCOPHEROL TRANSFER PROTEIN BOUND TO ITS LIGAND	KWR	0.30
1KO9	NATIVE STRUCTURE OF THE HUMAN 8-OXOGUANINE DNA GLYCOSYLASE HOGG1	GPR	0.30
1ZS9	CRYSTAL STRUCTURE OF HUMAN ENOLASE-PHOSPHATASE E1	KWR	0.33
1TQN	CRYSTAL STRUCTURE OF HUMAN MICROSOMAL P450 3A4	GPR	0.34
1ZVD	REGULATION OF SMURF2 UBIQUITIN LIGASE ACTIVITY BY ANCHORING THE E2 TO THE HECT DOMAIN	GPR	0.34
2HI4	CRYSTAL STRUCTURE OF HUMAN MICROSOMAL P450 1A2 IN COMPLEX WITH ALPHA-NAPHTHOFLAVONE	GPR	0.35

1D2S	CRYSTAL STRUCTURE OF THE N-TERMINAL LAMININ G-LIKE DOMAIN OF SHBG IN COMPLEX WITH DIHYDROTESTOSTERONE	GPR	0.38
1CIZ	X-RAY STRUCTURE OF HUMAN STROMELYSIN CATALYTIC DOMAIN COMPLEXES WITH NON-PEPTIDE INHIBITORS: IMPLICATION FOR INHIBITOR SELECTIVITY	KWR	0.39
2EVA	STRUCTURAL BASIS FOR THE INTERACTION OF TAK1 KINASE WITH ITS ACTIVATING PROTEIN TAB1	KWR	0.40
1VJY	CRYSTAL STRUCTURE OF A NAPHTHYRIDINE INHIBITOR OF HUMAN TGF BETA TYPE I RECEPTOR	KWR	0.40
2B69	CRYSTAL STRUCTURE OF HUMAN UDP-GLUCORONIC ACID DECARBOXYLAS	GPR	0.40
1N26	CRYSTAL STRUCTURE OF THE EXTRA-CELLULAR DOMAINS OF HUMAN INTERLEUKIN-6 RECEPTOR ALPHA CHAIN	GPR	0.41
1S31	CRYSTAL STRUCTURE ANALYSIS OF THE HUMAN TUB PROTEIN (ISOFORM A) SPANNING RESIDUES 289 THROUGH 561	GPR	0.44
1HDO	HUMAN BILIVERDIN IX BETA REDUCTASE: NADP COMPLEX	GPR	0.45
3CHO	CRYSTAL STRUCTURE OF LEUKOTRIENE A4 HYDROLASE IN COMPLEX WITH 2-AMINO-N-[4-(PHENYLMETHOXY)PHENYL]-ACETAMIDE	GPR	0.48
1CB0	STRUCTURE OF HUMAN 5'-DEOXY-5'-METHYLTHIOADENOSINE PHOSPHORYLASE AT 1.7 A RESOLUTION	GPR	0.50
1Q33	CRYSTAL STRUCTURE OF HUMAN ADP-RIBOSE PYROPHOSPHATASE NUDT9	GPR	0.53
1OZ2	CRYSTAL STRUCTURE OF 3-MBT REPEATS OF LETHAL (3) MALIGNANT BRAIN TUMOR (NATIVE-II) AT 1.55 ANGSTROM	GPR	0.54
1TDH	CRYSTAL STRUCTURE OF HUMAN ENDONUCLEASE VIII-LIKE 1 (NEIL1)	GPR	0.56
1PME	STRUCTURE OF PENTA MUTANT HUMAN ERK2 MAP KINASE COMPLEXED WITH A SPECIFIC INHIBITOR OF HUMAN P38 MAP KINASE	GPR	0.60
1PI1	CRYSTAL STRUCTURE OF A HUMAN MOB1 PROTEIN; TOWARD UNDERSTANDING MOB-REGULATED CELL CYCLE PATHWAYS.	GPR	0.61
1VZO	THE STRUCTURE OF THE N-TERMINAL KINASE DOMAIN OF MSK1 REVEALS A NOVEL AUTOINHIBITORY CONFORMATION FOR A DUAL KINASE PROTEIN	GPR	0.62
3COU	CRYSTAL STRUCTURE OF HUMAN NUDIX MOTIF 16 (NUDT16)	GPR	0.73
1CZA	MUTANT MONOMER OF RECOMBINANT HUMAN HEXOKINASE TYPE I COMPLEXED WITH GLUCOSE_ GLUCOSE-6-PHOSPHATE_ AND ADP	GPR	0.74
2VPJ	CRYSTAL STRUCTURE OF THE KELCH DOMAIN OF HUMAN KLHL12	GPR	2.00
3CKK	CRYSTAL STRUCTURE OF HUMAN METHYLTRANSFERASE-LIKE PROTEIN 1	KWR	2.00
	**u-plasminogen activator**		
2NSM	CRYSTAL STRUCTURE OF THE HUMAN CARBOXYPEPTIDASE N (KININASE I) CATALYTIC DOMAIN	LGR	0.10
1W6K	STRUCTURE OF HUMAN OSC IN COMPLEX WITH LANOSTEROL	LGR	0.10
2O8T	CRYSTAL STRUCTURE AND BINDING EPITOPES OF UROKINASE-TYPE PLASMINOGEN ACTIVATOR (C122A/N145Q) IN COMPLEX WITH INHIBITORS	LGR	0.11
1SPJ	STRUCTURE OF MATURE HUMAN TISSUE KALLIKREIN (HUMAN KALLIKREIN 1 OR KLK1) AT 1.70 ANGSTROM RESOLUTION WITH VACANT ACTIVE SITE	LGR	0.12
2DW5	CRYSTAL STRUCTURE OF HUMAN PEPTIDYLARGININE DEIMINASE 4 IN COMPLEX WITH N-ALPHA-BENZOYL-N5-(2-FLUORO-1-IMINOETHYL)-L-ORNITHINE AMIDE	LGR	0.12
1OHC	STRUCTURE OF THE PROLINE DIRECTED PHOSPHATASE CDC14	LGR	0.13
2PCX	CRYSTAL STRUCTURE OF P53DBD(R282Q) AT 1.54-ANGSTROM RESOLUTION	LGR	0.15
1Q33	CRYSTAL STRUCTURE OF HUMAN ADP-RIBOSE PYROPHOSPHATASE NUDT9	LGR	0.16
1KHB	PEPCK COMPLEX WITH NONHYDROLYZABLE GTP ANALOG_ NATIVE DATA	LGR	0.16
1UOU	CRYSTAL STRUCTURE OF HUMAN THYMIDINE PHOSPHORYLASE IN COMPLEX WITH A SMALL MOLECULE INHIBITOR	LGR	0.16
1Z8D	CRYSTAL STRUCTURE OF HUMAN MUSCLE GLYCOGEN PHOSPHORYLASE A WITH AMP AND GLUCOSE	LGR	0.17

1NM8	STRUCTURE OF HUMAN CARNITINE ACETYLTRANSFERASE: MOLECULAR BASIS FOR FATTY ACYL TRANSFER	LGR	0.18
1M6I	CRYSTAL STRUCTURE OF APOPTOSIS INDUCING FACTOR (AIF)	LGR	0.19
2Q5I	CRYSTAL STRUCTURE OF APO S581L GLYCYL-TRNA SYNTHETASE MUTAN	LGR	0.19
2CY7	THE CRYSTAL STRUCTURE OF HUMAN ATG4B	LGR	0.19
1UOU	CRYSTAL STRUCTURE OF HUMAN THYMIDINE PHOSPHORYLASE IN COMPLEX WITH A SMALL MOLECULE INHIBITOR	LGR	0.19
1OZN	1.5A CRYSTAL STRUCTURE OF THE NOGO RECEPTOR LIGAND BINDING DOMAIN REVEALS A CONVERGENT RECOGNITION SCAFFOLD MEDIATING INHIBITION OF MYELINATION	LGR	0.19
1FA9	HUMAN LIVER GLYCOGEN PHOSPHORYLASE A COMPLEXED WITH AMP	LGR	0.19
1LF7	CRYSTAL STRUCTURE OF HUMAN COMPLEMENT PROTEIN C8GAMMA AT 1.2 A RESOLUTION	LGR	0.20
1RYO	HUMAN SERUM TRANSFERRIN_ N-LOBE BOUND WITH OXALATE	LGR	0.20
2PE4	STRUCTURE OF HUMAN HYALURONIDASE 1_ A HYALURONAN HYDROLYZING ENZYME INVOLVED IN TUMOR GROWTH AND ANGIOGENESIS	LGR	0.21
2I7V	STRUCTURE OF HUMAN CPSF-73	LGR	0.22
1OZN	1.5A CRYSTAL STRUCTURE OF THE NOGO RECEPTOR LIGAND BINDING DOMAIN REVEALS A CONVERGENT RECOGNITION SCAFFOLD MEDIATING INHIBITION OF MYELINATION	LGR	0.22
2V24	STRUCTURE OF THE HUMAN SPRY DOMAIN-CONTAINING SOCS BOX PROTEIN SSB-4	LGR	0.22
1SIQ	THE CRYSTAL STRUCTURE AND MECHANISM OF HUMAN GLUTARYL-COA DEHYDROGENASE	LGR	0.23
2HQ6	STRUCTURE OF THE CYCLOPHILIN_CECYP16-LIKE DOMAIN OF THE SEROLOGICALLY DEFINED COLON CANCER ANTIGEN 10 FROM HOMO SAPIENS	LGR	0.23
2HZ6	THE CRYSTAL STRUCTURE OF HUMAN IRE1-ALPHA LUMINAL DOMAIN	LGR	0.24
1XWI	CRYSTAL STRUCTURE OF VPS4B	LGR	0.24
1GS9	APOLIPOPROTEIN E4_ 22K DOMAIN	LGR	0.25
2CL3	CRYSTAL STRUCTURE OF HUMAN CLEAVAGE AND POLYADENYLATION SPECIFICITY FACTOR 5 (CPSF5)	LGR	0.26
2ILR	CRYSTAL STRUCTURE OF HUMAN FANCONI ANEMIA PROTEIN E C-TERMINAL DOMAIN	LGR	0.26
1LCY	CRYSTAL STRUCTURE OF THE MITOCHONDRIAL SERINE PROTEASE HTRA	LGR	0.27
2O36	CRYSTAL STRUCTURE OF ENGINEERED THIMET OLIGOPEPTIDASE WITH NEUROLYSIN SPECIFICITY IN NEUROTENSIN CLEAVAGE SITE	LGR	0.27
1W8M	ENZYMATIC AND STRUCTURAL CHARACTERISATION OF NON PEPTIDE LIGAND CYCLOPHILIN COMPLEXES	LGR	0.27
1SQW	CRYSTAL STRUCTURE OF KD93_ A NOVEL PROTEIN EXPRESSED IN THE HUMAN PRO	LGR	0.27
1MF7	INTEGRIN ALPHA M I DOMAIN	LGR	0.28
1SIQ	THE CRYSTAL STRUCTURE AND MECHANISM OF HUMAN GLUTARYL-COA DEHYDROGENASE	LGR	0.28
2BIT	CRYSTAL STRUCTURE OF HUMAN CYCLOPHILIN D AT 1.7 A RESOLUTIO	LGR	0.28
1ORE	HUMAN ADENINE PHOSPHORIBOSYLTRANSFERASE	LGR	0.28
1B0F	CRYSTAL STRUCTURE OF HUMAN NEUTROPHIL ELASTASE WITH MDL 101 146	LGR	0.28
2OPW	CRYSTAL STRUCTURE OF HUMAN PHYTANOYL-COA DIOXYGENASE PHYHD1 (APO)	LGR	0.28
1TA0	THREE-DIMENSIONAL STRUCTURE OF A RNA-POLYMERASE II BINDING PROTEIN WITH ASSOCIATED LIGAND.	LGR	0.28
1ZD3	HUMAN SOLUBLE EPOXIDE HYDROLASE 4-(3-CYCLOHEXYLURIEDO)-BUTYRIC ACID COMPLEX	LGR	0.29
1H30	C-TERMINAL LG DOMAIN PAIR OF HUMAN GAS6	LGR	0.29
1S35	CRYSTAL STRUCTURE OF REPEATS 8 AND 9 OF HUMAN ERYTHROID SPECTRIN	LGR	0.31

1MX3	CRYSTAL STRUCTURE OF CTBP DEHYDROGENASE CORE HOLO FORM	LGR	0.31
2CY7	THE CRYSTAL STRUCTURE OF HUMAN ATG4B	LGR	0.32
1W6K	STRUCTURE OF HUMAN OSC IN COMPLEX WITH LANOSTEROL	LGR	0.33
2GRY	CRYSTAL STRUCTURE OF THE HUMAN KIF2 MOTOR DOMAIN IN COMPLEX WITH ADP	LGR	0.34
1LI4	HUMAN S-ADENOSYLHOMOCYSTEINE HYDROLASE COMPLEXED WITH NEPLANOCIN	LGR	0.34
1OSH	A CHEMICAL_ GENETIC_ AND STRUCTURAL ANALYSIS OF THE NUCLEAR BILE ACID RECEPTOR FXR	LGR	0.35
2OYC	CRYSTAL STRUCTURE OF HUMAN PYRIDOXAL PHOSPHATE PHOSPHATASE	LGR	0.36
1ZC0	CRYSTAL STRUCTURE OF HUMAN HEMATOPOIETIC TYROSINE PHOSPHATASE (HEPTP) CATALYTIC DOMAIN	LGR	0.36
1DG6	CRYSTAL STRUCTURE OF APO2L/TRAIL	LGR	0.37
2NZ2	CRYSTAL STRUCTURE OF HUMAN ARGININOSUCCINATE SYNTHASE IN COMPLEX WITH ASPARTATE AND CITRULLINE	LGR	0.37
2OZU	CRYSTAL STRUCTURE OF HUMAN MYST HISTONE ACETYLTRANSFERASE 3 IN COMPLEX WITH ACETYLCOENZYME A	LGR	0.37
2IUW	CRYSTAL STRUCTURE OF HUMAN ABH3 IN COMPLEX WITH IRON ION AND 2-OXOGLUTARATE	LGR	0.37
2FVV	HUMAN DIPHOSPHOINOSITOL POLYPHOSPHATE PHOSPHOHYDROLASE 1	LGR	0.40
2O36	CRYSTAL STRUCTURE OF ENGINEERED THIMET OLIGOPEPTIDASE WITH NEUROLYSIN SPECIFICITY IN NEUROTENSIN CLEAVAGE SITE	LGR	0.40
1Q20	CRYSTAL STRUCTURE OF HUMAN CHOLESTEROL SULFOTRANSFERASE (SULT2B1B) IN THE PRESENCE OF PAP AND PREGNENOLONE	LGR	0.41
1EVS	CRYSTAL STRUCTURE OF HUMAN ONCOSTATIN M	LGR	0.41
1M6I	CRYSTAL STRUCTURE OF APOPTOSIS INDUCING FACTOR (AIF)	LGR	0.42
1UPV	CRYSTAL STRUCTURE OF THE HUMAN LIVER X RECEPTOR BETA LIGAND BINDING DOMAIN IN COMPLEX WITH A SYNTHETIC AGONIST	LGR	0.42
1L6J	CRYSTAL STRUCTURE OF HUMAN MATRIX METALLOPROTEINASE MMP9 (GELATINASE B).	LGR	0.43
1P0I	CRYSTAL STRUCTURE OF HUMAN BUTYRYL CHOLINESTERASE	LGR	0.43
1OHC	STRUCTURE OF THE PROLINE DIRECTED PHOSPHATASE CDC14	LGR	0.43
3BQC	HIGH PH-VALUE CRYSTAL STRUCTURE OF EMODIN IN COMPLEX WITH THE CATALYTIC SUBUNIT OF PROTEIN KINASE CK2	LGR	0.43
2OU2	ACETYLTRANSFERASE DOMAIN OF HUMAN HIV-1 TAT INTERACTING PROTEIN_ 60KDA_ ISOFORM 3	LGR	0.43
1MP8	CRYSTAL STRUCTURE OF FOCAL ADHESION KINASE (FAK)	LGR	0.43
1YWN	VEGFR2 IN COMPLEX WITH A NOVEL 4-AMINO-FURO[2_3-D]PYRIMIDIN	LGR	0.44
3BQC	HIGH PH-VALUE CRYSTAL STRUCTURE OF EMODIN IN COMPLEX WITH THE CATALYTIC SUBUNIT OF PROTEIN KINASE CK2	LGR	0.46
2V9K	CRYSTAL STRUCTURE OF HUMAN PUS10_ A NOVEL PSEUDOURIDINE SYNTHASE.	LGR	0.47
1Y6B	CRYSTAL STRUCTURE OF VEGFR2 IN COMPLEX WITH A 2-ANILINO-5- ARYL-OXAZOLE INHIBITOR	LGR	0.48
2P39	CRYSTAL STRUCTURE OF HUMAN FGF23	LGR	0.48
2DE0	CRYSTAL STRUCTURE OF HUMAN ALPHA 1_6-FUCOSYLTRANSFERASE_ FUT8	LGR	0.48
2A2C	X-RAY STRUCTURE OF HUMAN N-ACETYL GALACTOSAMINE KINASE COMPLEXED WITH MG-ADP AND N-ACETYL GALACTOSAMINE 1-PHOSPHATE	LGR	0.50
2UUI	CRYSTAL STRUCTURE OF HUMAN LEUKOTRIENE C4 SYNTHASE	LGR	0.50
1ZD3	HUMAN SOLUBLE EPOXIDE HYDROLASE 4-(3-CYCLOHEXYLURIEDO)-BUTYRIC ACID COMPLEX	LGR	0.53
1LCY	CRYSTAL STRUCTURE OF THE MITOCHONDRIAL SERINE PROTEASE HTRA	LGR	0.63
1JDW	CRYSTAL STRUCTURE AND MECHANISM OF L-ARGININE: GLYCINE AMIDINOTRANSFERASE: A MITOCHONDRIAL ENZYME INVOLVED IN CREATINE BIOSYNTHESIS	LGR	2.00

2AEX	THE 1.58A CRYSTAL STRUCTURE OF HUMAN COPROPORPHYRINOGEN OXIDASE REVEALS THE STRUCTURAL BASIS OF HEREDITARY COPROPORPHYRIA	LGR	2.00
2QOL	HUMAN EPHA3 KINASE AND JUXTAMEMBRANE REGION_ Y596:Y602:S768G TRIPLE MUTANT	LGR	2.00
	**DisProt**		
	**chymase**		
	Securin	FPF	
	**coagulation factor IXa**		
	Salivary proline-rich glycoprotein precursor PRB4	EGR	
	Troponin I_ cardiac muscle	EGR	
	Dynein intermediate chain 1_ cytosolic	EGR	
	20 kDa nuclear cap binding protein	EGR	
	Adenomatous polyposis coli	EGR	
	Naked2	EGR	
	**coagulation factor XIa**		
	Cyclin-dependent kinase inhibitor 1	EAR	
	Glucocorticoid receptor	EAR	
	Tubulin beta-1 chain	EAR	
	adducin 1 (alpha) isoform a	EAR	
	Ubiquitin-like 1-activating enzyme E1B	EAR	
	**coagulation factor XIIa**		
	Cyclin-dependent kinase inhibitor 1	QGR	
	Phosphatidylinositol-4-phosphate 5-kinase type II beta	QGR	
	Salivary proline-rich glycoprotein precursor PRB4	QGR	
	Apolipoprotein E [Precursor]	QGR	
	**complement component C2a**		
	T-cell surface glycoprotein CD3 zeta chain [Precursor]	LGR	
	Breast cancer type 1 susceptibility protein	LGR	
	Dynein intermediate chain 1_ cytosolic	LGR	
	**DESC1 peptidase**		
	Salivary proline-rich glycoprotein precursor PRB4	EGR	
	Troponin I_ cardiac muscle	EGR	
	Dynein intermediate chain 1_ cytosolic	EGR	
	20 kDa nuclear cap binding protein	EGR	
	Peroxisomal biogenesis factor 5	FVR	
	Adenomatous polyposis coli	EGR	
	Naked2	EGR	
	Nogo-B	FVR	
	**elastase-1**		
	Cyclin-dependent kinase inhibitor 1C	APA	
	Cyclin-dependent kinase inhibitor 1C	AAA	
	Nonhistone chromosomal protein HMG-17	APA	
	Replication protein A 70 kDa DNA-binding subunit	APA	
	Synaptobrevin 2	APA	
	Alpha-synuclein	AAA	

	elastic titin-skeletal [fragment]	APA	
	Estrogen receptor alpha	AAA	
	c-fos	AAA	
	Transcription factor p65	APA	
	Calcineurin	AAA	
	Microtubule-associated protein tau isoform Tau-F	AAA	
	POU domain class 2_ associating factor 1	APA	
	RNA polymerase II subunit A C-terminal domain phosphatase	APA	
	T-cell surface glycoprotein CD3 zeta chain [Precursor]	APA	
	G2/mitotic-specific cyclin B1	APA	
	POU domain_ class 2_ transcription factor 1	AAA	
	Breast cancer type 1 susceptibility protein	AAA	
	adducin 1 (alpha) isoform a	AAA	
	Beta adducin	APA	
	Myc proto-oncogene protein	AAA	
	Apoptosis regulator Bcl-2	APA	
	Apoptosis regulator Bcl-2	AAA	
	Apoptosis regulator Bcl-X	AAA	
	ADP-ribosylation factor binding protein GGA1	AAA	
	cyclin-dependent kinase inhibitor 2A	AAA	
	Dynein intermediate chain 1_ cytosolic	AAA	
	Mothers against decapentaplegic homolog 4	APA	
	Mothers against decapentaplegic homolog 4	AAA	
	Major prion protein	AAA	
	Peroxisomal biogenesis factor 5	APA	
	Androgen receptor	AAA	
	Nuclear protein 1	AAA	
	Naked2	AAA	
	Nogo-B	APA	
	Nogo-B	AAA	
	**elastase-2**		
	Cyclin-dependent kinase inhibitor 1C	AAA	
	Alpha-synuclein	AAA	
	elastic titin-skeletal [fragment]	AAV	
	Estrogen receptor alpha	AAA	
	c-fos	AAA	
	Calcineurin	AAA	
	Microtubule-associated protein tau isoform Tau-F	AAA	
	DNA repair protein XRCC4	AAV	
	POU domain_ class 2_ transcription factor 1	AAA	
	POU domain_ class 2_ transcription factor 1	AAV	
	Breast cancer type 1 susceptibility protein	AAA	
	adducin 1 (alpha) isoform a	AAA	
	Myc proto-oncogene protein	AAA	
	Apoptosis regulator Bcl-2	AAA	
	Apoptosis regulator Bcl-X	AAA	
	ADP-ribosylation factor binding protein GGA1	AAA	
	cyclin-dependent kinase inhibitor 2A	AAA	
	Dynein intermediate chain 1_ cytosolic	AAA	
	Mothers against decapentaplegic homolog 4	AAA	
	Mothers against decapentaplegic homolog 4	AAV	
	Major prion protein	AAA	
	Androgen receptor	AAA	
	Nuclear protein 1	AAA	
	Adenomatous polyposis coli	AAV	
	Naked2	AAA	
	Nogo-B	AAA	

	Nogo-B	AAV	
	**furin**		
	Cystic fibrosis transmembrane conductance regulator	RRR	
	Cyclin-dependent kinase inhibitor 1	RKR	
	Cyclin-dependent kinase inhibitor 1C	RKR	
	elastic titin-skeletal [fragment]	RKR	
	Max protein	RKR	
	CD4 glycoprotein (Precursor)	RRR	
	Fragile X mental retardation 1 protein	RRR	
	T-cell surface glycoprotein CD3 zeta chain [Precursor]	RRR	
	protein phosphatase 1_ regulatory (inhibitor) subunit 11 isoform 1	RRR	
	protein phosphatase 1_ regulatory (inhibitor) subunit 11 isoform 1	RKR	
	Breast cancer type 1 susceptibility protein	RKR	
	Interferon-induced guanylate-binding protein 1	RRR	
	Dynein intermediate chain 1_ cytosolic	RKR	
	80 kDa nuclear cap binding protein	RRR	
	Jagged-1	RKR	
	FH1/FH2 domain-containing protein	RKR	
	Ubiquitin-like 1-activating enzyme E1B	RKR	
	B-cell antigen receptor complex-associated protein alpha-chain [Precursor]	RKR	
	Naked2	RKR	
	**granzyme A**		
	Cyclin-dependent kinase inhibitor 1	GPR	
	RNA polymerase II subunit A C-terminal domain phosphatase	GPR	
	Thymidine kinase	GPR	
	NF-kappaB inhibitor alpha	GPR	
	Cryptochrome 2	GPR	
	Naked2	GPR	
	**granzyme B**		
	c-fos	AAD	
	Max protein	AAD	
	Glycine amidinotransferase_ mitochondrial [Precursor]	AAD	
	adducin 1 (alpha) isoform a	AAD	
	Ectodysplasin A	AAD	
	T-cell surface glycoprotein CD3 delta chain [Precursor]	AAD	
	**granzyme H**		
	Estrogen receptor alpha	AAF	
	Estrogen receptor alpha	AAY	
	c-fos	FLF	
	Pyridoxine-5'-phosphate oxidase	AAM	
	Breast cancer type 1 susceptibility protein	AAF	
	Ankyrin-2 (202 KDa splice form)	AAF	
	Androgen receptor	AAY	
	**hepsin**		
	Cyclin-dependent kinase inhibitor 1	LSR	
	Cyclin-dependent kinase inhibitor 1C	LSR	
	Human growth hormone binding protein	LSR	
	Parathyroid hormone-related protein precursor	LSR	
	POU domain_ class 2_ transcription factor 1	LSR	
	Myelin basic protein	LSR	
	Breast cancer type 1 susceptibility protein	LSR	
	Peroxisomal biogenesis factor 5	FVR	
	Cryptochrome 2	LSR	

	Nogo-B	FVR	
	**kallikrein 1**		
	elastic titin-skeletal [fragment]	VLR	
	Calcineurin	VLR	
	von Hippel-Lindau Tumor Suppressor	VLR	
	Adenomatous polyposis coli	PFR	
	Naked2	PFR	
	**kallikrein-related peptidase 11**		
	Adenomatous polyposis coli	PFR	
	Naked2	PFR	
	**kallikrein-related peptidase 14**		
	Antibacterial protein FALL-39 precursor	VPR	
	Cyclin-dependent kinase inhibitor 1	VPR	
	elastic titin-skeletal [fragment]	VPR	
	Fragile X mental retardation 1 protein	VPR	
	NF-kappaB inhibitor alpha	VPR	
	**kallikrein-related peptidase 2**		
	Cystic fibrosis transmembrane conductance regulator	ARR	
	DNA topoisomerase I	ARR	
	Myelin basic protein	ARR	
	Nuclear protein 1	ARR	
	Adenomatous polyposis coli	PFR	
	Naked2	PFR	
	**kallikrein-related peptidase 4**		
	Antibacterial protein FALL-39 precursor	VPR	
	Cyclin-dependent kinase inhibitor 1	VPR	
	elastic titin-skeletal [fragment]	VLK	
	elastic titin-skeletal [fragment]	VLR	
	elastic titin-skeletal [fragment]	VPR	
	Calcineurin	VLR	
	Fragile X mental retardation 1 protein	VPR	
	Smad anchor for receptor activation	VLK	
	Stathmin	VLK	
	Calpastatin	VLK	
	Beta casein [Precursor](/	VLK	
	Copper-transporting ATPase 1	VLK	
	von Hippel-Lindau Tumor Suppressor	VLR	
	Apolipoprotein A-I	VLK	
	Rho-GTPase-activating protein 1	VLK	
	Ankyrin-2 (202 KDa splice form)	VLK	
	NF-kappaB inhibitor alpha	VPR	
	Adenomatous polyposis coli	PFR	
	Naked2	PFR	
	**kallikrein-related peptidase 6**		
	Antibacterial protein FALL-39 precursor	VPR	
	Cystic fibrosis transmembrane conductance regulator	QAR	
	Cyclin-dependent kinase inhibitor 1	VPR	
	Cyclin-dependent kinase inhibitor 1	GPR	
	Cyclin-dependent kinase inhibitor 1C	QAR	
	elastic titin-skeletal [fragment]	VPR	
	Microtubule-associated protein tau isoform Tau-F	QAR	
	Fragile X mental retardation 1 protein	VPR	

	Cardiac phospholamban	QAR	
	RNA polymerase II subunit A C-terminal domain phosphatase	GPR	
	T-cell surface glycoprotein CD3 zeta chain [Precursor]	FSR	
	Breast cancer type 1 susceptibility protein	FSR	
	Breast cancer type 1 susceptibility protein	GPK	
	Thymidine kinase	GPR	
	NF-kappaB inhibitor alpha	VPR	
	NF-kappaB inhibitor alpha	GPR	
	Cryptochrome 2	GPR	
	Ubiquitin-like 1-activating enzyme E1B	GPK	
	Adenomatous polyposis coli	FSR	
	Adenomatous polyposis coli	GPK	
	Naked2	GPR	
	**marapsin**		
	Cyclin-dependent kinase inhibitor 1	GPR	
	Cyclin-dependent kinase inhibitor 1C	VGR	
	RNA polymerase II subunit A C-terminal domain phosphatase	GPR	
	Thymidine kinase	GPR	
	NF-kappaB inhibitor alpha	GPR	
	Cryptochrome 2	GPR	
	Naked2	GPR	
	**matriptase**		
	Cystic fibrosis transmembrane conductance regulator	QAR	
	Cyclin-dependent kinase inhibitor 1	EAR	
	Cyclin-dependent kinase inhibitor 1	QGR	
	Cyclin-dependent kinase inhibitor 1C	QAR	
	Glucocorticoid receptor	EAR	
	Phosphatidylinositol-4-phosphate 5-kinase type II beta	QGR	
	Salivary proline-rich glycoprotein precursor PRB4	EGR	
	Salivary proline-rich glycoprotein precursor PRB4	QGR	
	Microtubule-associated protein tau isoform Tau-F	QAR	
	Cardiac phospholamban	QAR	
	Troponin I_ cardiac muscle	EGR	
	Tubulin beta-1 chain	EAR	
	T-cell surface glycoprotein CD3 zeta chain [Precursor]	LGR	
	Breast cancer type 1 susceptibility protein	LGR	
	Breast cancer type 1 susceptibility protein	AFK	
	adducin 1 (alpha) isoform a	EAR	
	Apolipoprotein E [Precursor]	QGR	
	Dynein intermediate chain 1_ cytosolic	EGR	
	Dynein intermediate chain 1_ cytosolic	LGR	
	20 kDa nuclear cap binding protein	EGR	
	Ubiquitin-like 1-activating enzyme E1B	EAR	
	Adenomatous polyposis coli	EGR	
	Naked2	EGR	
	Nogo-B	AFK	
	**matriptase-3**		
	Peroxisomal biogenesis factor 5	FVR	
	Nogo-B	FVR	
	**mesotrypsin**		
	Cyclin-dependent kinase inhibitor 1	GPR	
	RNA polymerase II subunit A C-terminal domain phosphatase	GPR	
	Thymidine kinase	GPR	
	NF-kappaB inhibitor alpha	GPR	

	Cryptochrome 2	GPR	
	Naked2	GPR	
	Cyclin-dependent kinase inhibitor 1C	APV	
	elastic titin-skeletal [fragment]	APV	
	Microtubule-associated protein tau isoform Tau-F	APV	
	Beta casein [Precursor](/	APV	
	adducin 1 (alpha) isoform a	APV	
	Beta adducin	APV	
	Nogo-B	APV	
	**plasma kallikrein**		
	Glucocorticoid receptor	QRR	
	Nonhistone chromosomal protein HMG-17	QRR	
	Parathyroid hormone-related protein precursor	QRR	
	Smad anchor for receptor activation	QRR	
	T-cell surface glycoprotein CD3 zeta chain [Precursor]	QRR	
	Insulin-like growth factor binding protein 6	QRR	
	T-cell surface glycoprotein CD3 epsilon chain [Precursor]	QRR	
	Adenomatous polyposis coli	PFR	
	Naked2	PFR	
	**plasmin**		
	Breast cancer type 1 susceptibility protein	GPK	
	Ubiquitin-like 1-activating enzyme E1B	GPK	
	Adenomatous polyposis coli	GPK	
	**prostasin**		
	Cyclin-dependent kinase inhibitor 1	GPR	
	RNA polymerase II subunit A C-terminal domain phosphatase	GPR	
	Thymidine kinase	GPR	
	NF-kappaB inhibitor alpha	GPR	
	Cryptochrome 2	GPR	
	Naked2	GPR	
	**protein C (activated)**		
	elastic titin-skeletal [fragment]	EPR	
	Thymidylate synthase	EPR	
	Microtubule-associated protein tau isoform Tau-F	EPR	
	Bone sialoprotein	EPR	
	Sulfotransferase family cytosolic 2B member 1:Isoform II	EPR	
	Thymidylate synthase	EPR	
	Naked2	EPR	
	**testisin**		
	Cyclin-dependent kinase inhibitor 1B	LTR	
	Cardiac phospholamban	LTR	
	T-cell surface glycoprotein CD3 zeta chain [Precursor]	FSR	
	Breast cancer type 1 susceptibility protein	FSR	
	Dynein intermediate chain 1_ cytosolic	LTR	
	Adenomatous polyposis coli	FSR	
	**thrombin**		
	Antibacterial protein FALL-39 precursor	VPR	
	Cyclin-dependent kinase inhibitor 1	VPR	
	Cyclin-dependent kinase inhibitor 1	GPR	
	elastic titin-skeletal [fragment]	VPR	
	Fragile X mental retardation 1 protein	VPR	
	RNA polymerase II subunit A C-terminal domain phosphatase	GPR	

	Thymidine kinase	GPR	
	NF-kappaB inhibitor alpha	VPR	
	NF-kappaB inhibitor alpha	GPR	
	Peroxisomal biogenesis factor 5	FVR	
	Cryptochrome 2	GPR	
	Naked2	GPR	
	Nogo-B	FVR	
	**t-plasminogen activator**		
	T-cell surface glycoprotein CD3 zeta chain [Precursor]	LGR	
	Breast cancer type 1 susceptibility protein	LGR	
	Dynein intermediate chain 1_ cytosolic	LGR	
	**tripeptidyl-peptidase I**		
	Estrogen receptor alpha	AAF	
	c-fos	GPM	
	c-fos	FPA	
	Pyridoxine-5'-phosphate oxidase	GPM	
	protein phosphatase 1_ regulatory (inhibitor) subunit 11 isoform 1	GPM	
	Breast cancer type 1 susceptibility protein	AAF	
	Beta adducin	GPM	
	Ankyrin-2 (202 KDa splice form)	AAF	
	**tripeptidyl-peptidase II**		
	Estrogen receptor alpha	AAF	
	Breast cancer type 1 susceptibility protein	AAF	
	Ankyrin-2 (202 KDa splice form)	AAF	
	**trypsin-2**		
	Cyclin-dependent kinase inhibitor 1	GPR	
	RNA polymerase II subunit A C-terminal domain phosphatase	GPR	
	Thymidine kinase	GPR	
	NF-kappaB inhibitor alpha	GPR	
	Cryptochrome 2	GPR	
	Naked2	GPR	
	**tryptase beta**		
	Cyclin-dependent kinase inhibitor 1C	AKR	
	Nonhistone chromosomal protein HMG-17	KAK	
	Retinoic acid receptor RXR-alpha	RNR	
	Alpha-synuclein	KAK	
	Microtubule-associated protein tau isoform Tau-F	KAK	
	Microtubule-associated protein tau isoform Tau-F	KAK	
	Beta casein [Precursor](/	KAK	
	G2/mitotic-specific cyclin B1	KAK	
	G2/mitotic-specific cyclin B1	AKR	
	Breast cancer type 1 susceptibility protein	RNR	
	Breast cancer type 1 susceptibility protein	KAK	
	Protein-arginine deiminase type IV	RNR	
	Serine/threonine protein phosphatase 5	KAK	
	Thyroid cancer protein 1	KAK	
	Thyroid cancer protein 1	AKR	
	Peroxisomal biogenesis factor 5	AKR	
	Ubiquitin-like 1-activating enzyme E1B	AKR	
	T-cell surface glycoprotein CD3 epsilon chain [Precursor]	KAK	
	Adenomatous polyposis coli	KAK	
	Securin	KAK	

	**tryptase gamma 1**		
	Cyclin-dependent kinase inhibitor 1	GPR	
	RNA polymerase II subunit A C-terminal domain phosphatase	GPR	
	Thymidine kinase	GPR	
	NF-kappaB inhibitor alpha	GPR	
	Cryptochrome 2	GPR	
	Naked2	GPR	
	**u-plasminogen activator**		
	T-cell surface glycoprotein CD3 zeta chain [Precursor]	LGR	
	Breast cancer type 1 susceptibility protein	LGR	
	Dynein intermediate chain 1_ cytosolic	LGR	
