# Supplementary material for: A Sequence and Structure Based Method to Predict Putative Substrates, Functions and Regulatory Networks of Endo Proteases
Source: PLoS One. 2009 May 27;4(5):e5700. doi: 10.1371/journal.pone.0005700 (PMC2683571; doi:10.1371/journal.pone.0005700)
Supplement: Table S7 — Putative Substrates of Matriptase from PDB Rank Ordered According to rSASA (0.08 MB PDF) [file pone.0005700.s008.pdf]

Table S7 - Putative substrates of Matriptase from PDB rank ordered according to rSASA

PDB I D	Name of the Protein	Cleavage Sequence	rSASA
1W6K	STRUCTURE OF HUMAN OSC IN COMPLEX WITH LANOSTEROL	LGRF	0.09
2O8T	CRYSTAL STRUCTURE AND BINDING EPITOPES OF UROKINASE-TYPE PLASMINOGEN ACTIVATOR (C122A/N145Q) IN COMPLEX WITH INHIBITORS	LGRS	0.11
2DW5	HUMAN PEPTIDYLARGININE DEIMINASE 4 IN COMPLEX WITH N-ALPHA-BENZOYL-N5-(2-FLUORO-1-IMINOETHYL)-L- ORNITHINE AMIDE	LGRI	0.11
2NSM	HUMAN CARBOXYPEPTIDASE N (KININASE I) CATALYTIC DOMAIN	LGRE	0.12
1OHC	STRUCTURE OF THE PROLINE DIRECTED PHOSPHATASE CDC14	LGRT	0.12
1LCT	STRUCTURE OF THE RECOMBINANT N-TERMINAL LOBE OF HUMAN LACTOFERRIN AT 2.0 ANGSTROMS RESOLUTION	AFKC	0.14
1UOU	HUMAN THYMIDINE PHOSPHORYLASE IN COMPLEX WITH A SMALL MOLECULE INHIBITOR	LGRF	0.14
1SPJ	STRUCTURE OF MATURE HUMAN TISSUE KALLIKREIN (HUMAN KALLIKREIN 1 OR KLK1) AT 1.70 ANGSTROM RESOLUTION WITH VACANT ACTIVE SITE	LGRH	0.14
1Q33	HUMAN ADP-RIBOSE PYROPHOSPHATASE NUDT9	LGRW	0.14
1RYO	HUMAN SERUM TRANSFERRIN N-LOBE BOUND WITH OXALATE	AFKC	0.14
1KHB	PEPCK COMPLEX WITH NONHYDROLYZABLE GTP ANALOG NATIVE DATA	LGRW	0.14
1Z8D	HUMAN MUSCLE GLYCOGEN PHOSPHORYLASE A WITH AMP AND GLUCOSE	LGRL	0.16
1T32	A DUAL INHIBITOR OF THE LEUKOCYTE PROTEASES CATHEPSIN G AND CHYMASE WITH THERAPEUTIC EFFICACY IN ANIMALS MODELS OF INFLAMMATION	AFKG	0.16
2Q5I	APO S581L GLYCYL-TRNA SYNTHETASE MUTANT	LGRI	0.16
2PCX	P53DBD(R282Q) AT 1.54-ANGSTROM RESOLUTION	LGRN	0.16
1OZN	1.5A NOGO RECEPTOR LIGAND BINDING DOMAIN REVEALS A CONVERGENT RECOGNITION SCAFFOLD MEDIATING INHIBITION OF MYELINATION	LGRL	0.16
1UOU	HUMAN THYMIDINE PHOSPHORYLASE IN COMPLEX WITH A SMALL MOLECULE INHIBITOR	LGRC	0.16
2O3H	HUMAN C65A APE	EGRV	0.16
2B5M	DDB1	QGRI	0.17
1WMA	HUMAN CBR1 IN COMPLEX WITH HYDROXY-PP	QGRV	0.17
1LF7	HUMAN COMPLEMENT PROTEIN C8GAMMA AT 1.2 A RESOLUTION	LGRF	0.17
1YCK	HUMAN PEPTIDOGLYCAN RECOGNITION PROTEIN (PGRP-S)	EGRG	0.17
1RYO	HUMAN SERUM TRANSFERRIN N-LOBE BOUND WITH OXALATE	LGRS	0.17
1FA9	HUMAN LIVER GLYCOGEN PHOSPHORYLASE A COMPLEXED WITH AMP	LGRL	0.18
3CHO	LEUKOTRIENE A4 HYDROLASE IN COMPLEX WITH 2-AMINO-N-[4-(PHENYLMETHOXY)PHENYL]-ACETAMIDE	QGRM	0.18
1OI1	MBT DOMAINS OF HUMAN SCML2	EARD	0.18
1OZN	1.5A NOGO RECEPTOR LIGAND BINDING DOMAIN REVEALS A CONVERGENT RECOGNITION SCAFFOLD MEDIATING INHIBITION OF MYELINATION	LGRL	0.18
1LI4	HUMAN S-ADENOSYLHOMOCYSTEINE HYDROLASE COMPLEXED WITH NEPLANOCIN	EGRL	0.19
2I7Q	HUMAN CHOLINE KINASE A	QARF	0.19
2G6B	HUMAN RAB26 IN COMPLEX WITH A GTP ANALOGUE	AFKV	0.19
2RKU	STRUCTURE OF PLK1 IN COMPLEX WITH BI2536	EARY	0.19
2B5M	DDB1	AFKE	0.19
2V24	STRUCTURE OF THE HUMAN SPRY DOMAIN-CONTAINING SOCS BOX PROTEIN SSB-4	LGRS	0.19
1NM8	STRUCTURE OF HUMAN CARNITINE ACETYLTRANSFERASE: MOLECULAR BASIS FOR FATTY ACYL TRANSFER	LGRT	0.19
1T7V	ZN-ALPHA-2-GLYCOPROTEIN; BACULO-ZAG PEG 200	QGRF	0.19

2FY2	STRUCTURES OF LIGAND BOUND HUMAN CHOLINE ACETYLTRANSFERASE PROVIDE INSIGHT INTO REGULATION OF ACETYLCHOLINE SYNTHESIS	EGRV	0.20
1MF7	INTEGRIN ALPHA M I DOMAIN	AFKI	0.20
2I7V	STRUCTURE OF HUMAN CPSF-73	LGRA	0.20
2HGS	HUMAN GLUTATHIONE SYNTHETASE	EARL	0.21
1GS9	APOLIPOPROTEIN E4 22K DOMAIN	LGRF	0.21
1N5U	X-RAY STUDY OF HUMAN SERUM ALBUMIN COMPLEXED WITH HEME	AFKA	0.21
2DH2	HUMAN ED-4F2HC	QARL	0.21
2B5M	DDB1	EGRL	0.22
2I53	CYCLIN K	EARY	0.22
2ILR	HUMAN FANCONI ANEMIA PROTEIN E C- TERMINAL DOMAIN	LGRI	0.22
1KT0	STRUCTURE OF THE LARGE FKBP-LIKE PROTEIN FKBP51 INVOLVED IN STEROID RECEPTOR COMPLEXES	EGRC	0.23
1SO7	MALTOSE-INDUCED STRUCTURE OF THE HUMAN CYTOLSOLIC SIALIDASE NEU2	QARL	0.23
2UW2	HUMAN RIBONUCLEOTIDE REDUCTASE SUBUNIT R2	EARC	0.23
2GY5	TIE2 LIGAND-BINDING DOMAIN CRYSTAL STRUCTURE	EGRV	0.23
2HQ6	STRUCTURE OF THE CYCLOPHILIN_CECYP16-LIKE DOMAIN OF THE SEROLOGICALLY DEFINED COLON CANCER ANTIGEN 10 FROM HOMO SAPIENS	LGRA	0.23
2OPW	HUMAN PHYTANOYL-COA DIOXYGENASE PHYHD1 (APO)	LGRV	0.23
2F9L	3D STRUCTURE OF INACTIVE HUMAN RAB11B GTPASE	AFKN	0.23
2HZ6	THE HUMAN IRE1-ALPHA LUMINAL DOMAIN	LGRT	0.23
3BGS	STRUCTURE OF HUMAN PURINE NUCLEOSIDE PHOSPHORYLASE WITH L- DADME-IMMH AND PHOSPHATE	QGRF	0.23
1SIQ	THE CRYSTAL STRUCTURE AND MECHANISM OF HUMAN GLUTARYL-COA DEHYDROGENASE	LGRL	0.23
1M6I	APOPTOSIS INDUCING FACTOR (AIF)	LGRK	0.23
1W8M	ENZYMATIC AND STRUCTURAL CHARACTERISATION OF NON PEPTIDE LIGAND CYCLOPHILIN COMPLEXES	LGRV	0.23
1R9O	P4502C9 WITH FLURBIPROFEN BOUND	EARC	0.23
2CY7	THE HUMAN ATG4B	LGRK	0.23
1LCY	MITOCHONDRIAL SERINE PROTEASE HTRA	LGRS	0.24
2BIT	HUMAN CYCLOPHILIN D AT 1.7 A RESOLUTIC	LGRV	0.24
1MF7	INTEGRIN ALPHA M I DOMAIN	LGRT	0.24
1SQW	KD93 A NOVEL PROTEIN EXPRESSED IN THE HUMAN PRO	LGRI	0.24
1SIQ	THE CRYSTAL STRUCTURE AND MECHANISM OF HUMAN GLUTARYL-COA DEHYDROGENASE	QARD	0.24
2PE4	STRUCTURE OF HUMAN HYALURONIDASE 1 A HYALURONAN HYDROLYZING ENZYME INVOLVED IN TUMOR GROWTH AND ANGIOGENESIS	LGRA	0.24
3BKB	HUMAN FELINE SARCOMA VIRAL ONCOGENE HOMOLOGUE (V-FES)	EARI	0.25
2V7O	HUMAN CALCIUM-CALMODULIN-DEPENDENT PROTEIN KINASE II GAMMA	EARI	0.25
1NN6	HUMAN PRO-CHYMASE	AFKG	0.25
1ORE	HUMAN ADENINE PHOSPHORIBOSYLTRANSFERASE	LGRL	0.25
2D7I	CRSYTAL STRUCTURE OF PP-GALNAC-T10 WITH UDP GALNAC AND MN2	QGRP	0.25
2B3X	STRUCTURE OF AN ORTHORHOMBIC CRYSTAL FORM OF HUMAN CYTOSOLIC ACONITASE (IRP1)	EGRV	0.25
1SZ7	HUMAN BET3	AFKM	0.26
1YCK	HUMAN PEPTIDOGLYCAN RECOGNITION PROTEIN (PGRP-S)	QARN	0.26
1MX3	CTBP DEHYDROGENASE CORE HOLO FORM	LGRV	0.26
1ALU	HUMAN INTERLEUKIN-6	QARA	0.26
1S35	REPEATS 8 AND 9 OF HUMAN ERYTHROID SPECTRIN	LGRM	0.27
1GS9	APOLIPOPROTEIN E4 22K DOMAIN	QARL	0.27
2HQQ	HUMAN KETOHEXOKINASE COMPLEXED TO DIFFERENT SUGAR MOLECULES	EGRN	0.27

2PET	LUTHERAN GLYCOPROTEIN N-TERMINAL DOMAINS 1 AND 2.	QGRL	0.27
1W6K	STRUCTURE OF HUMAN OSC IN COMPLEX WITH LANOSTEROL	LGRW	0.27
1KL9	N-TERMINAL SEGMENT OF HUMAN EUKARYOTIC INITIATION FACTOR 2ALPHA	AFKH	0.27
2DW4	HUMAN LSD1 AT 2.3 A RESOLUTION	EARD	0.27
2OEW	STRUCTURE OF ALIX/AIP1 BRO1 DOMAIN	AFKQ	0.28
2ZG1	TWO N-TERMINAL DOMAINS OF SIGLEC-5 IN COMPLEX WITH 6'-SIALYLLACTOSE	QGRF	0.28
1HVF	STRUCTURAL AND ELECTROPHYSIOLOGICAL ANALYSIS OF ANNEXIN V MUTANTS. MUTAGENESIS OF HUMAN ANNEXIN V AN IN VITRO VOLTAGE-GATED CALCIUM CHANNEL PROVIDES INFORMATION ABOUT THE STRUCTURAL FEATURES OF THE ION PATHWAY THE VOLTAGE SENSOR AND THE ION SELECTIVITY FILTER	AFKT	0.28
1ZD3	HUMAN SOLUBLE EPOXIDE HYDROLASE 4-(3-CYCLOHEXYLURIEDO)- BUTYRIC ACID COMPLEX	LGRT	0.28
1ZD3	HUMAN SOLUBLE EPOXIDE HYDROLASE 4-(3-CYCLOHEXYLURIEDO)- BUTYRIC ACID COMPLEX	LGRT	0.28
1ZIV	CATALYTIC DOMAIN OF HUMAN CALPAIN-9	AFKD	0.28
2B1P	INHIBITOR COMPLEX OF JNK3	QARD	0.28
2I7Q	HUMAN CHOLINE KINASE A	QGRL	0.28
1SIQ	THE CRYSTAL STRUCTURE AND MECHANISM OF HUMAN GLUTARYL-COA DEHYDROGENASE	LGRA	0.28
2ESB	HUMAN DUSP18	QGRT	0.29
2O36	ENGINEERED THIMET OLIGOPEPTIDASE WITH NEUROLYSIN SPECIFICITY IN NEUROTENSIN CLEAVAGE SITE	LGRR	0.29
2H6D	PROTEIN KINASE DOMAIN OF THE HUMAN 5'-AMP-ACTIVATED PROTEIN KINASE CATALYTIC SUBUNIT ALPHA-2 (AMPK ALPHA-2 CHAIN)	EARR	0.29
1YPV	STRUCTURE OF HUMAN THYMIDYLATE SYNTHASE AT LOW SALT CONDITIONS	QARY	0.29
2GJK	STRUCTURAL AND FUNCTIONAL INSIGHTS INTO THE HUMAN UPF1 HELICASE CORE	QGRE	0.30
1K1B	ANKYRIN REPEAT DOMAIN OF BCL-3: A UNIQUE MEMBER OF THE IKAPPAB PROTEIN FAMILY	EARN	0.30
2Z5Y	HUMAN MONOAMINE OXIDASE A (G110A) WITH HARMINE	EARD	0.30
2I7V	STRUCTURE OF HUMAN CPSF-73	EARF	0.30
1H30	C-TERMINAL LG DOMAIN PAIR OF HUMAN GAS6	LGRP	0.30
2I4I	HUMAN DEAD-BOX RNA HELICASE DDX3X	EARK	0.30
1X04	ENDOPHILIN BAR DOMAIN (MUTANT)	EGRR	0.30
1E2S	AN ARYLSULFATASE A MUTANT C69A	EARY	0.30
1X03	ENDOPHILIN BAR DOMAIN	EGRR	0.30
1XWI	VPS4B	LGRK	0.30
1X8B	STRUCTURE OF HUMAN WEE1A KINASE: KINASE DOMAIN COMPLEXED WITH INHIBITOR PD0407824	QGRL	0.30
1P0I	HUMAN BUTYRYL CHOLINESTERASE	EARN	0.31
1WB0	SPECIFICITY AND AFFINITY OF NATURAL PRODUCT CYCLOPENTAPEPTIDE INHIBITOR ARGIFIN AGAINST HUMAN CHITINAS	EARF	0.31
2A91	ERBB2 DOMAINS 1-3	EGRY	0.31
1OSH	A CHEMICAL GENETIC AND STRUCTURAL ANALYSIS OF THE NUCLEAR BILE ACID RECEPTOR FXR	LGRL	0.31
2OYC	HUMAN PYRIDOXAL PHOSPHATE PHOSPHATASE	LGRS	0.31
2HI4	HUMAN MICROSOMAL P450 1A2 IN COMPLEX WITH ALPHA-NAPHTHOFLAVONE	QARR	0.31
1IAP	P115RHOGEF RGRGS DOMAIN	EARE	0.31
1LI4	HUMAN S-ADENOSYLHOMOCYSTEINE HYDROLASE COMPLEXED WITH NEPLANOCIN	LGRH	0.32
1TA0	THREE-DIMENSIONAL STRUCTURE OF A RNA-POLYMERASE II BINDING PROTEIN WITH ASSOCIATED LIGAND.	LGRD	0.32
2IUW	HUMAN ABH3 IN COMPLEX WITH IRON ION AND 2-OXOGLUTARATE	LGRC	0.32
2V9R	FIRST AND SECOND IG DOMAINS FROM HUMAN ROBO1 (FORM 2)	EGRP	0.32

2FY2	STRUCTURES OF LIGAND BOUND HUMAN CHOLINE ACETYLTRANSFERASE PROVIDE INSIGHT INTO REGULATION OF ACETYLCHOLINE SYNTHESIS	EART	0.32
1NUF	ROLE OF CALCIUM IONS IN THE ACTIVATION AND ACTIVITY OF THE TRANSGLUTAMINASE 3 ENZYME	EARV	0.32
1OZ2	3-MBT REPEATS OF LETHAL (3) MALIGNANT BRAIN TUMOR (NATIVE-II) AT 1.55 ANGSTROM	AFKV	0.32
1UOU	HUMAN THYMIDINE PHOSPHORYLASE IN COMPLEX WITH A SMALL MOLECULE INHIBITOR	QARE	0.32
2QQ5	HUMAN SDR FAMILY MEMBER 1	QGRL	0.33
1K95	DES(1-52)GRANCALCIN WITH BOUND CALCIUM	AFKE	0.33
1E0S	SMALL G PROTEIN ARF6-GDP	EARQ	0.33
1JTV	17BETA-HYDROXYSTEROID DEHYDROGENASE TYPE 1 COMPLEXED WITH TESTOSTERONE	QGRL	0.33
1MX3	CTBP DEHYDROGENASE CORE HOLO FORM	EGRI	0.33
2AXN	HUMAN INDUCIBLE FORM 6- PHOSPHOFRUCTO-2-KINASE/FRUCTOSE-2 6-BISPHOSPHATASE	QGRI	0.33
1BY7	HUMAN PLASMINOGEN ACTIVATOR INHIBITOR-2. LOOP (66-98) DELETION MUTANT	EARK	0.34
1WB0	SPECIFICITY AND AFFINITY OF NATURAL PRODUCT CYCLOPENTAPEPTIDE INHIBITOR ARGIFIN AGAINST HUMAN CHITINAS	QGRY	0.34
2QQJ	B1B2 DOMAINS FROM HUMAN NEUROPILIN 2	EARS	0.34
1M9I	PHOSPHORYLATION-MIMICKING MUTANT T356D OF ANNEXIN VI	QARE	0.34
1B0F	HUMAN NEUTROPHIL ELASTASE WITH MDL 101 146	LGRN	0.34
2CL3	HUMAN CLEAVAGE AND POLYADENYLATION SPECIFICITY FACTOR 5 (CPSF5)	LGRQ	0.34
1K04	FOCAL ADHESION TARGETING DOMAIN OF FOCAL ADHESION KINASE	QARL	0.34
1CZA	MUTANT MONOMER OF RECOMBINANT HUMAN HEXOKINASE TYPE I COMPLEXED WITH GLUCOSE GLUCOSE-6-PHOSPHATE AND ADP	EGRI	0.35
1M6I	APOPTOSIS INDUCING FACTOR (AIF)	QARS	0.35
2OIL	HUMAN RAB25 IN COMPLEX WITH GDP	EARM	0.35
2F9L	3D STRUCTURE OF INACTIVE HUMAN RAB11B GTPASE	EARA	0.35
1KMQ	A CONSTITUTIVELY ACTIVATED RHOA MUTANT (Q63L)	EGRD	0.35
1N83	COMPLEX BETWEEN THE ORPHAN NUCLEAR HORMONE RECEPTOR ROR(ALPHA)-LBD AND CHOLESTEROL	AFKA	0.35
2QZ4	HUMAN PARAPLEGIN AAA DOMAIN IN COMPLEX WITH ADP	EARA	0.35
1CZA	MUTANT MONOMER OF RECOMBINANT HUMAN HEXOKINASE TYPE I COMPLEXED WITH GLUCOSE GLUCOSE-6-PHOSPHATE AND ADP	EGRM	0.35
1KAO	SMALL G PROTEIN RAP2A WITH GDP	EGRA	0.36
1X9D	HUMAN CLASS I ALPHA-1 2-MANNOSIDASE IN COMPLEX WITH THIO-DISACCHARIDE SUBSTRATE ANALOGUE	EARK	0.36
2V5O	STRUCTURE OF HUMAN IGF2R DOMAINS 11-14	AFKR	0.36
2GRY	HUMAN KIF2 MOTOR DOMAIN IN COMPLEX WITH ADP	LGRN	0.36
2FVV	HUMAN DIPHOSPHOINOSITOL POLYPHOSPHATE PHOSPHOHYDROLASE 1	LGRL	0.36
1IAT	HUMAN PHOSPHOGLUCOSE ISOMERASE/NEUROLEUKIN/AUTOCRINE MOTILITY FACTOR/MATURATION FACTOR	EGRA	0.36
1V4S	HUMAN GLUCOKINASE	EGRM	0.36
1DG6	APO2L/TRAIL	LGRK	0.36
2B9E	HUMAN NSUN5 PROTEIN	QGRA	0.36
2OZU	HUMAN MYST HISTONE ACETYLTRANSFERASE 3 IN COMPLEX WITH ACETYLCOENZYME A	LGRL	0.37
1P0I	HUMAN BUTYRYL CHOLINESTERASE	LGRL	0.37
3BER	HUMAN DEAD-BOX RNA-HELICASE DDX47 CONSERVED DOMAIN I IN COMPLEX WITH AMP	QGRD	0.37
1CJM	HUMAN SULT1A3 WITH SULFATE BOUND	QARP	0.37
2QLU	ACTIVIN RECEPTOR TYPE II KINASE DOMAIN FROM HUMAN	EARL	0.37
2JDF	HUMAN GAMMA-B CRYSTALLIN	QGRS	0.37
2NZ2	HUMAN ARGININOSUCCINATE SYNTHASE IN COMPLEX WITH ASPARTATE AND CITRULLINE	LGRE	0.37

3CBQ	HUMAN REM2 GTPASE WITH BOUND GDP	EGRH	0.38
1MP8	FOCAL ADHESION KINASE (FAK)	LGRC	0.38
2CY7	THE HUMAN ATG4B	LGRD	0.38
1ZGK	1.35 ANGSTROM STRUCTURE OF THE KELCH DOMAIN OF KEAP1	QGRI	0.38
2OC3	CATALYTIC DOMAIN OF HUMAN PROTEIN TYROSINE PHOSPHATASE NON-RECEPTOR TYPE 18	EARR	0.38
1M8Z	A PUMILIO-HOMOLOGY DOMAIN	AFKG	0.38
1XA6	HUMAN BETA2-CHIMAERIN	EARG	0.38
2OHF	HUMAN OLA1 IN COMPLEX WITH AMPPCP	QGRN	0.38
2G3Y	HUMAN SMALL GTPASE GEM	EGRA	0.38
2QXI	HIGH RESOLUTION STRUCTURE OF HUMAN KALLIKREIN 7 IN COMPLEX WITH SUC-ALA-ALA-PRO-PHE-CHLOROMETHYLKETONE	QARL	0.38
1L6J	HUMAN MATRIX METALLOPROTEINASE MMP9 (GELATINASE B).	LGRF	0.39
1IAT	HUMAN PHOSPHOGLUCOSE ISOMERASE/NEUROLEUKIN/AUTOCRINE MOTILITY FACTOR/MATURATION FACTOR	EARK	0.39
1WOJ	HUMAN PHOSPHODIESTERASE	AFKK	0.39
1OHC	STRUCTURE OF THE PROLINE DIRECTED PHOSPHATASE CDC14	LGRT	0.39
1M6I	APOPTOSIS INDUCING FACTOR (AIF)	LGRR	0.39
1Z32	STRUCTURE-FUNCTION RELATIONSHIPS IN HUMAN SALIVARY ALPHA- AMYLASE: ROLE OF AROMATIC RESIDUES	QGRT	0.40
1IMJ	HUMAN CCG1/TAFII250-INTERACTING FACTOR B (CIB)	QARF	0.40
2O36	ENGINEERED THIMET OLIGOPEPTIDASE WITH NEUROLYSIN SPECIFICITY IN NEUROTENSIN CLEAVAGE SITE	LGRD	0.40
2FAU	HUMAN VPS26	AFKQ	0.40
2NZ2	HUMAN ARGININOSUCCINATE SYNTHASE IN COMPLEX WITH ASPARTATE AND CITRULLINE	EARK	0.40
1JDN	HORMONE RECEPTOR	EGRF	0.40
1Q20	HUMAN CHOLESTEROL SULFOTRANSFERASE (SULT2B1B) IN THE PRESENCE OF PAP AND PREGNENOLONE	LGRP	0.40
1UZE	COMPLEX OF THE ANTI-HYPERTENSIVE DRUG ENALAPRILAT AND THE HUMAN TESTICULAR ANGIOTENSIN I-CONVERTING ENZYME	QARK	0.41
3BQC	HIGH PH-VALUE EMODIN IN COMPLEX WITH THE CATALYTIC SUBUNIT OF PROTEIN KINASE CK2	LGRH	0.41
1D3G	HUMAN DIHYDROOROTATE DEHYDROGENASE COMPLEXED WITH BREQUINAR ANALOG	QGRV	0.41
1LS6	HUMAN SULT1A1 COMPLEXED WITH PAP AND P-NITROPHENOL	QARP	0.41
1ZC0	HUMAN HEMATOPOIETIC TYROSINE PHOSPHATASE (HEPTP) CATALYTIC DOMAIN	LGRA	0.41
2B5M	DDB1	QGRH	0.42
1EVS	HUMAN ONCOSTATIN M	LGRR	0.42
1ZSX	HUMAN POTASSIUM CHANNEL KV BETA- SUBUNIT (KCNAB2)	EGRR	0.42
1HDR	HUMAN DIHYDROPTERIDINE REDUCTASE NADH BINARY COMPLEX EXPRESSED IN ESCHERICHIA COLI BY A CDNA CONSTRUCTED FROM ITS RAT HOMOLOGUE	EARR	0.42
2EC8	EXCTRACELLULAR DOMAIN OF THE RECEPTOR TYROSINE KINASE KIT	AFKA	0.42
1DR9	A SOLUBLE FORM OF B7-1 (CD80)	AFKR	0.43
2DQ7	FYN KINASE DOMAIN COMPLEXED WITH STAUROSPORINE	EGRA	0.43
1QCY	THE I-DOMAIN OF HUMAN INTEGRIN ALPHA1BETA1	EARG	0.43
2OU2	ACETYLTRANSFERASE DOMAIN OF HUMAN HIV-1 TAT INTERACTING PROTEIN 60KDA ISOFORM 3	LGRH	0.43
2DE0	HUMAN ALPHA 1 6-FUCOSYLTRANSFERASE FUT8	LGRT	0.43
2EFK	EFC DOMAIN OF CDC42-INTERACTING PROTEIN 4	EGRR	0.43
1UPV	HUMAN LIVER X RECEPTOR BETA LIGAND BINDING DOMAIN IN COMPLEX WITH A SYNTHETIC AGONIST	LGRE	0.44
1MQ4	AURORA-A PROTEIN KINASE	EGRM	0.44
2V40	HUMAN ADENYLOSUCCINATE SYNTHETASE ISOZYME 2 IN COMPLEX WITH GDP	AFKE	0.44

1ZIV	CATALYTIC DOMAIN OF HUMAN CALPAIN-9	EART	0.44
2NSM	HUMAN CARBOXYPEPTIDASE N (KININASE I) CATALYTIC DOMAIN	EGRH	0.44
2BH9	X-RAY STRUCTURE OF A DELETION VARIANT OF HUMAN GLUCOSE 6- PHOSPHATE DEHYDROGENASE COMPLEXED WITH STRUCTURAL AND COENZYME NADP	EGRG	0.44
2Q5I	APO S581L GLYCYL-TRNA SYNTHETASE MUTAN	EARY	0.45
1B0F	HUMAN NEUTROPHIL ELASTASE WITH MDL 101 146	QGRR	0.45
1E5W	STRUCTURE OF ISOLATED FERM DOMAIN AND FIRST LONG HELIX OF MOESIN	QARE	0.45
1BX4	STRUCTURE OF HUMAN ADENOSINE KINASE AT 1.50 ANGSTROMS	QGRD	0.45
2UUI	HUMAN LEUKOTRIENE C4 SYNTHASE	LGRL	0.46
1JTV	17BETA-HYDROXYSTEROID DEHYDROGENASE TYPE 1 COMPLEXED WITH TESTOSTERONE	EGRV	0.46
1CB0	STRUCTURE OF HUMAN 5'-DEOXY-5'-METHYLTHIOADENOSINE PHOSPHORYLASE AT 1.7 A RESOLUTION	EGRT	0.46
2ALR	ALDEHYDE REDUCTASE	QARG	0.47
1HDR	HUMAN DIHYDROPTERIDINE REDUCTASE NADH BINARY COMPLEX EXPRESSED IN ESCHERICHIA COLI BY A CDNA CONSTRUCTED FROM ITS RAT HOMOLOGUE	EGRT	0.47
1YWN	VEGFR2 IN COMPLEX WITH A NOVEL 4-AMINO-FURO[2 3-D]PYRIMIDIN	LGRG	0.47
3BQC	HIGH PH-VALUE EMODIN IN COMPLEX WITH THE CATALYTIC SUBUNIT OF PROTEIN KINASE CK2	LGRG	0.48
2A2C	X-RAY STRUCTURE OF HUMAN N-ACETYL GALACTOSAMINE KINASE COMPLEXED WITH MG-ADP AND N-ACETYL GALACTOSAMINE 1- PHOSPHATE	LGRN	0.49
2IVV	PHOSPHORYLATED RET TYROSINE KINASE DOMAIN COMPLEXED WITH THE INHIBITOR PP1	QGRI	0.50
1ZD3	HUMAN SOLUBLE EPOXIDE HYDROLASE 4-(3-CYCLOHEXYLURIEDO)- BUTYRIC ACID COMPLEX	LGRK	0.50
1ZD3	HUMAN SOLUBLE EPOXIDE HYDROLASE 4-(3-CYCLOHEXYLURIEDO)- BUTYRIC ACID COMPLEX	LGRK	0.50
1A7S	ATOMIC RESOLUTION STRUCTURE OF HBP	QGRH	0.50
3CTZ	STRUCTURE OF HUMAN CYTOSOLIC X-PROLYL AMINOPEPTIDASE	QGRQ	0.50
2PNY	STRUCTURE OF HUMAN ISOPENTENYL-DIPHOSPHATE DELTA-ISOMERASE	EARG	0.50
1J72	MUTANT MACROPHAGE CAPPING PROTEIN (CAP G) WITH ACTIN-SEVERING ACTIVITY IN THE CA2+-FREE FORM	QGRE	0.51
2A2K	AN ACTIVE SITE MUTANT C473S OF CDC25B PHOSPHATASE CATALYTIC DOMAIN	AFKD	0.51
2P39	HUMAN FGF23	LGRA	0.51
2A8B	CATALYTIC DOMAIN OF HUMAN TYROSINE PHOSPHATASE RECEPTOR TYPE R	QGRG	0.52
1SK4	C-TERMINAL PEPTIDOGLYCAN-BINDING DOMAIN OF HUMAN PEPTIDOGLYCAN RECOGNITION PROTEIN IALPHA	EARE	0.52
2I7Q	HUMAN CHOLINE KINASE A	EGRE	0.52
2HQQ	HUMAN KETOHEXOKINASE COMPLEXED TO DIFFERENT SUGAR MOLECULES	QGRS	0.53
1WMA	HUMAN CBR1 IN COMPLEX WITH HYDROXY-PP	AFKV	0.53
2V9K	HUMAN PUS10 A NOVEL PSEUDOURIDINE SYNTHASE	LGRD	0.53
1L6J	HUMAN MATRIX METALLOPROTEINASE MMP9 (GELATINASE B).	EGRS	0.54
2OIL	HUMAN RAB25 IN COMPLEX WITH GDP	QARE	0.54
1Y6B	VEGFR2 IN COMPLEX WITH A 2-ANILINO-5- ARYL-OXAZOLE INHIBITOR	LGRG	0.54
2HRB	HUMAN CARBONYL REDUCTASE 3 COMPLEXED WITH NADP+	AFKS	0.55
1GWZ	CATALYTIC DOMAIN OF THE PROTEIN TYROSINE PHOSPHATASE SHP-1	QGRD	0.55
2IVV	PHOSPHORYLATED RET TYROSINE KINASE DOMAIN COMPLEXED WITH THE INHIBITOR PP1	EGRK	0.56
1N6A	STRUCTURE OF SET7/9	EGRP	0.56
1CZT	C2 DOMAIN OF HUMAN COAGULATION FACTOR V	QGRV	0.57
1LCY	MITOCHONDRIAL SERINE PROTEASE HTRA	LGRE	0.59
1R0P	TYROSINE KINASE DOMAIN OF THE HEPATOCYTE GROWTH FACTOR RECEPTOR C-MET IN COMPLEX WITH THE MICROBIAL ALKALOID K-252A	QGRR	0.59
1L6J	HUMAN MATRIX METALLOPROTEINASE MMP9 (GELATINASE B).	EGRG	0.59

2IVV	PHOSPHORYLATED RET TYROSINE KINASE DOMAIN COMPLEXED WITH THE INHIBITOR PP1	AFKI	0.60
2O8T	CRYSTAL STRUCTURE AND BINDING EPITOPES OF UROKINASE-TYPE PLASMINOGEN ACTIVATOR (C122A/N145Q) IN COMPLEX WITH INHIBITORS	QGRM	0.62
1LF7	HUMAN COMPLEMENT PROTEIN C8GAMMA AT 1.2 A RESOLUTION	QARG	0.62
1UV5	GLYCOGEN SYNTHASE KINASE 3 BETA COMPLEXED WITH 6- BROMOINDIRUBIN-3'-OXIME	AFKN	0.63
1P6F	STRUCTURE OF THE HUMAN NATURAL CYTOTOXICITY RECEPTOR NKP46	EGRS	0.64
1W7L	HUMAN KYNURENINE AMINOTRANSFERASE I	QARR	0.65
2PET	LUTHERAN GLYCOPROTEIN N-TERMINAL DOMAINS 1 AND 2.	EGRH	0.68
1LN1	HUMAN PHOSPHATIDYLCHOLINE TRANSFER PROTEIN IN COMPLEX WITH DILINOLEOYLPHOSPHATIDYLCHOLINE	EGRK	0.68
2EC8	EXCTRACELLULAR DOMAIN OF THE RECEPTOR TYROSINE KINASE KIT	AFKH	0.74
2IWR	GTPASE LIKE DOMAIN OF CENTAURIN GAMMA 1 (HUMAN)	EGRG	0.80
1HGU	HUMAN GROWTH HORMONE	AFKQ	0.81
1IAT	HUMAN PHOSPHOGLUCOSE ISOMERASE/NEUROLEUKIN/AUTOCRINE MOTILITY FACTOR/MATURATION FACTOR	EARV	0.90
1E8Y	STRUCTURE DETERMINANTS OF PHOSPHOINOSITIDE 3-KINASE INHIBITION BY WORTMANNIN LY294002 QUERCETIN MYRICETIN AND STAUROSPORINE	AFKZ	2.00
1GSM	A REASSESSMENT OF THE MADCAM-1 STRUCTURE AND ITS ROLE IN INTEGRIN RECOGNITION.	EGR-	2.00
1JDW	CRYSTAL STRUCTURE AND MECHANISM OF L-ARGININE: GLYCINE AMIDINOTRANSFERASE: A MITOCHONDRIAL ENZYME INVOLVED IN CREATINE BIOSYNTHESIS	LGRZ	2.00
1NG2	STRUCTURE OF AUTOINHIBITED P47PHOX	QARZ	2.00
1R55	CATALYTIC DOMAIN OF HUMAN ADAM 33	EARZ	2.00
1UU3	STRUCTURE OF HUMAN PDK1 KINASE DOMAIN IN COMPLEX WITH LY333531	QARZ	2.00
2AEX	THE 1.58A HUMAN COPROPORPHYRINOGEN OXIDASE REVEALS THE STRUCTURAL BASIS OF HEREDITARY COPROPORPHYRIA	LGRZ	2.00
2EW1	RAB30 IN COMPLEX WITH A GTP ANALOGUE	EAR-	2.00
2NSM	HUMAN CARBOXYPEPTIDASE N (KININASE I) CATALYTIC DOMAIN	QARZ	2.00
2NZ6	PTPRJ INACTIVATING MUTANT C1239S	QGRZ	2.00
2O8T	CRYSTAL STRUCTURE AND BINDING EPITOPES OF UROKINASE-TYPE PLASMINOGEN ACTIVATOR (C122A/N145Q) IN COMPLEX WITH INHIBITORS	EGRZ	2.00
2OI0	CRYSTAL STRUCTURE ANALYSIS 0F THE TNF-A COVERTING ENZYME (TACE) IN COMPLEXED WITH ARYL SULFONAMIDE	EGR-	2.00
2Q3H	THE RHOUA IN THE GDP-BOUND STATE.	EGRZ	2.00
2QOL	HUMAN EPHA3 KINASE AND JUXTAMEMBRANE REGION Y596:Y602:S768G TRIPLE MUTAN	LGRZ	2.00
2V9K	HUMAN PUS10 A NOVEL PSEUDOURIDINE SYNTHASE	EGRZ	2.00
3BQC	HIGH PH-VALUE EMODIN IN COMPLEX WITH THE CATALYTIC SUBUNIT OF PROTEIN KINASE CK2	QARZ	2.00
3C8X	LIGAND BINDING DOMAIN OF HUMAN EPHRIN A2 (EPHA2) RECEPTOR PROTEIN KINASE	EARZ	2.00

Note: The query sequences were tripeptides of the type P3P2P1. Residues next to the P1 were included while calculating SASA values to account for amino acids at either side of the scissile bond.

Z' represents a disordered residue and the estimated SASA values may be inaccurate in such cases.
