# Supplementary material for: A Sequence and Structure Based Method to Predict Putative Substrates, Functions and Regulatory Networks of Endo Proteases
Source: PLoS One. 2009 May 27;4(5):e5700. doi: 10.1371/journal.pone.0005700 (PMC2683571; doi:10.1371/journal.pone.0005700)
Supplement: Table S8 — Putative Substrates of Matriptase (rSASA >0.4) Filtered for Membrane/Extracellular Localization. (0.06 MB PDF) [file pone.0005700.s009.pdf]

Table S8 - Putative substrates of matriptase with rSASA >0.4 filtered for membrane/extracellular localization.

Protein Name (PDB ID)	Cleavage sequence	rSASA	Comments
ADAM 33 (1R55) *	EARR	2.00	Cleavage site adjacent to the pro-peptide cleavage site; Start of the sequence in PDB; External to the membrane
Urokinase plasminogen activator (2O8T)	EGRC	2.00	Cleavage sequence within the peptidase domain followed immediately by a disulfide bond; Secreted protein
Receptor-type tyrosine-protein phosphatase eta (2NZ6)	QGRZ	2.00	Cleavage On the Cytoplasmic Side
Carboxypeptidase N catalytic chain (2NSM)	QART	2.00	Secreted protein (traceable author statement); Cleavage between 442-444; protein sequence ends at 460.
3-Phosphoinositide dependent protein kinase- 1 (1UU3)	QARA	2.00	According to Uniprot this is a peripheral membrane protein; cleavage sequence present within the kinase domain therefore the site would be on the cytoplasmic face of the membrane
Ephrin type-A receptor 2 (3C8X)*	EARH	2.00	Transmembrane protein; Topology Unclear; By comparison with the Ephrin type-A receptor 3 (2QOL), the cleavage site is likely to be extracellular
Casein kinase II subunit alpha (3BQC)	QARH	2.00	In uniprot Cellular compartment described as plasma membrane (besides its location within nucleus) ; topology unclear; Sequence at the end of the structure; 58 residues away from C-terminus in the native protein sequence
Ephrin receptor (2QOL)	LGRV	2.00	The sequence in Uniprot is LSR and not LGR as in the PDB; LSR is located on the cytoplasmic side
Ras homolog gene family, member U (2Q3H)*	EGRG	2.00	According to Uniprot the protein is localized to focal adhesion sites; Topology unclear; the cleavage site is ~16 and 45 residues away from the from the N-terminus in the PDB and native protein sequence respectively
Human Growth Harmone (1HGU)	AFKQ	0.81	Sequence in uniprot is IFK and not AFK
Lutheran glycoprotein (2PET)*	EGRH	0.68	Cleavage site within the topologically defined extracelluar region
Rab30 in complex with a GTP analogue (2EW1)	EARZ	2.00	Cleavage on the cytoplasmic side of the protein
MADCAM-1 (1GSM)	EGRZ	2.00	Sequence is HSP in the corresponding uniprot sequence
Human complement c1S protease (1ELV)	EGRT	0.67	Extracellular by experimental observation. Topology unclear
Coagulation Factor V (1CZT)*	QGRV	0.57	Plasma membrane localization is inferred form experiment; Secreted protein (by similarity, Uniprot) cleavage with in the coagulation factor V light chain

Myotubularin-related protein 2 (1ZSQ)	EGRT	0.56	Peripheral membrane protein; Partly associated with membrane; topology unclear
Mast/stem cell growth factor receptor (2EC8)*	AFKA,AFKH	0.42,0.74	Link absent in PDB for unitprot' idependent query in uniprot fetches P10721; both the cleavage sequences are present in the extracellular region (localization in the extracellular meliu inferred from direct assay)
Hepatocyte growth factor receptor (1R0P)	QGRR	0.59	Cleavage site present in the cytoplasmic domain
Receptor-type tyrosine-protein phosphatase R (2A8B)	QGRG	0.52	Cleavage site present in the cytoplasmic domain
natural cytotoxicity triggering receptor 1 (1P6F)*	EGRS	0.64	Cleavage site within topologically defined extracellular region
Coagulation factor viii precursor (1D7P)	QGRS	0.53	Plasma membrane localization inferred from experiment (uniprot). Cleavage site within factor VIIIa light chain; topology unclear
Heparin binding protein (1A7S)	QGRH	0.50	Uniprot - Present in cytoplasmic granules
Proto-oncogene tyrosine-protein kinase receptor ret precursor (2IVV)	QGRI	0.50	Cleavage site within the cytoplasmic domain
Ras-related protein Rab-25 (2OIL)	QARE	0.54	Cleavage on the 'potential' cytoplasmic side
Matrix metalloproteinase-9 (1L6J)*	EGRS,EGRG	0.54,0.59	Secreted/extracellular protein. Cleavage sites within the 83kDa processed region
Complement Protein C8gamma (1LF7)*	QARG	0.62	Membrane attack complex ; Secreted protein QARD in the uniprot instead of QARG; cleavage site within the functional domain
I-Domain of Integrin Alpha1beta1 (1QCY)*	EARG	0.43	Cleavage sequence within the extracellular region (potential)
Atrial Natriuretic Peptide Clearance Receptor (1JDN)*	EGRF	0.40	Cleavage sequence within the extracellular region (potential)
Fibroblast growth factor 23 (2P39)*	LGRA	0.51	Secreted; cleavage within the fibroblast growth factor 23 N-terminal peptide
Vascular endothelial growth factor receptor 2 (1Y6B)	LGRG	0.54	Cleavage site within the cytoplasmic domain
Proto-oncogene tyrosine-protein kinase receptor ret precursor (2IVV)	AFKI	0.60	Cleavage site within the cytoplasmic domain
Salivary alpha-amylase (1Z32)*	QGRT	0.40	Secreted; cleavage site is located seventh residue after the signal peptide
Casein kinase II subunit alpha (3BQC)	LGRH,LGRG	0.41,0.48	Topology unsure;
Vascular endothelial growth factor receptor 2 (1YWN)	LGRG	0.47	Cleavage site within potential cytoplasmic domain
Proto-oncogene tyrosine-protein kinase Fyn (2DQ7)	EGRA	0.43	Membrane localization by direct experiment; cleavage site within kinase domain therefore is on the cytoplamic side
MOESIN (1E5W)*	QARE	0.45	Membrane to membrane docking
T Lymphocyte activation antigen (1DR9)*	AFKR	0.43	Cleavage within extracellular region
Angiotensin-1-converting enzyme (1UZE)*	QARK	0.41	Extracellular region/ outerside of plasma membrane; cleavge within the extracellular domain (Uniprot)
Bile salt activated lipase (1F6W)	QGRK	0.40	Extracellular region as defined by experiment (Uniport) -topology unclear; present in pancreas and mammary gland

Putative substrates of matriptase identified from PDB (rSASA value of >0.4) were verified for their subcellular localization. Those proteins localized to the membrane/extracellular region were classified as most likely substrates and are listed here. Only those proteins in which the cleavage site was present in the extracellular region as verified by uniprot or those proteins known to be secreted were shortlisted for assigning function to matriptase (see Figure 4).

*- Indicates that these proteins were used for functional assigment and network.
