# Supplementary material for: A Sequence and Structure Based Method to Predict Putative Substrates, Functions and Regulatory Networks of Endo Proteases
Source: PLoS One. 2009 May 27;4(5):e5700. doi: 10.1371/journal.pone.0005700 (PMC2683571; doi:10.1371/journal.pone.0005700)
Supplement: Table S9 — Substrates of Furin, Hepsin, Matriptase and Testisin used in Building the Network (0.03 MB PDF) [file pone.0005700.s010.pdf]

Table S9 - Substrates of Furin, Hepsin, Matriptase and Testisin used in building the network

Protease Name	PDB ID	Cleavage Sequence	rSASA
Furin	1LCT	RRRRZ	2.00
Furin	1LF7	RPRRZ	2.00
Furin	1OEC	RARRE	0.63
Furin	2YV8	RFKRA	0.51
Furin	2P39	RAKRA	0.48
Furin	1T46	RRKRD	0.47
Furin	1IHK	RTKRH	0.43
Furin	2UUR	RPRRE	0.43
Furin	1H30	RFKRL	0.39
Furin	1ILK	RLRRC	0.27
Furin	1EAX	RLKRI	0.25
Furin	3CFW	RARRF	0.25
hepsin	1A7S	LSRF	0.22
hepsin	1B0F	LSRR	0.56
hepsin	1BoF	FVRG	0.34
hepsin	1HFC	FVRG	0.23
hepsin	1HGU	LSRL	0.36
hepsin	1HUW	LSRL	0.28
hepsin	1IJB	FVRY	0.26
hepsin	1MD8	FVRL	0.15
hepsin	1MFM	LSRK	0.48
hepsin	1MZA	LSRK	0.34
hepsin	1N3Y	FVRA	0.24
hepsin	1P0I	LSRS	0.17
hepsin	1SI5	FVRV	0.11
hepsin	1T32	LSRR	0.33
hepsin	1Z7C	LSRL	0.31
hepsin	1ZED	LSRN	0.31
hepsin	2EC8	FVRD	0.17
hepsin	2P39	LSRR	0.32
hepsin	2PPL	LSRI	0.13
hepsin	2QQI	FVRI	0.11
Matriptase	1CZT	QGRV	0.57
Matriptase	1D7P	QGRS	0.53
Matriptase	1DR9	AFKR	0.43
Matriptase	1E5W	QARE	0.45
Matriptase	1ELV	EGRT	0.67
Matriptase	1F6W	QGRK	0.40
Matriptase	1JDN	EGRF	0.40
Matriptase	1L6J	EGRG	0.59
Matriptase	1LF7	QARG	0.62
Matriptase	1P6F	EGRS	0.64
Matriptase	1QCY	EARG	0.43
Matriptase	1R55	EARZ	2.00
Matriptase	1UZE	QARK	0.41
Matriptase	1Z32	QGRT	0.40

Matriptase	1ZSQ	EGRT	0.56
Matriptase	2EC8	AFKH	0.74
Matriptase	2P39	LGRA	0.51
Matriptase	2PET	EGRH	0.68
testisin	1A7S	LTRR	0.53
testisin	1CIZ	LTRF	0.53
testisin	1CiZ	FSRL	0.31
testisin	1D7P	LTRY	0.19
testisin	1ILK	FSRV	0.31
testisin	1JdN	LTRV	0.15
testisin	1JDN	LTRV	0.10
testisin	1LCT	FSRV	0.25
testisin	1LJ5	LTRL	0.20
testisin	1MZA	FSRV	0.53
testisin	1R55	LTRH	0.39
testisin	1UZE	FSRP	0.25
testisin	2EC8	LTRL	0.35
testisin	2QQJ	LTRF	0.13
testisin	1LCT	FSRV	0.25
testisin	3CFW	FSRS	0.38
testisin	1ILK	FSRV	0.31
